# Supplementary material for: Heterogeneity of Zika virus exposure and outcome ascertainment across cohorts of pregnant women, their infants and their children: a metadata survey
Source: BMJ Open. 2022 Nov 22;12(11):e064362. doi: 10.1136/bmjopen-2022-064362 (PMC9685007; doi:10.1136/bmjopen-2022-064362)
Supplement: Supplementary data [file bmjopen-2022-064362supp001.pdf]

Appendix 1.

Table of Contents

I. **Zika search strategy** ... 2
PICO CRITERIA: ... 2
Zika search, Medline (through Ovid): ... 2
Zika search, Embase (through Ovid): ... 2
II. **Screening, eligibility and selection of studies trhough systematic search** ... 4
Figure S.1. Flowchart for study participation trough systematic search. ... 4
III. **Supplementary tables with descriptive zikv-ipd-ma metada-related statisitcs and figures** ... 5
Table S1. Detailed description of ZIKV-IPD-MA studies participating in the metadata survey (Part 1). ... 5
Table S1. Detailed description of ZIKV-IPD-MA studies participating in the metadata survey (Part 2). ... 12
Table S2. Infant and child development assessment among ZIKV-IPD-MA study participants of the metadata survey ... 17
Figure S2. STORCH and other additional testing among ZIKV-IPD-MA study participants of the metadata survey ... 17
Table S3. Sociodemographic and behavioral factors measured among ZIKV-IPD-MA study participants of the metadata survey. ... 18
Table S4. Genetic testing, fetal ultrasounds, and MRIs among ZIKV-IPD-MA study participants of the metadata survey ... 19
Table S5a. Type of maternal ZIKV assessment among ZIKV-IPD-MA study participants of the metadata survey. ... 20
Table S5b. Type of fetal ZIKV assessment among ZIKV-IPD-MA study participants of the metadata survey. ... 21
Table S5c. Type of infant ZIKV assessment among ZIKV-IPD-MA study participants of the metadata survey. ... 22

Carabali M, *et al. BMJ Open* 2022; 12:e064362. doi: 10.1136/bmjopen-2022-064362

I. ZIKA SEARCH STRATEGY

PICO CRITERIA:

Population	Exposure	Comparator	Outcome (open)
Pregnant women and newborns	ZIKV infection during pregnancy	No ZIKV infection during pregnancy	Primary: microcephaly, miscarriage, fetal loss.<br>Secondary: early/late fetal death, stillbirth, ocular abnormalities, hearing loss, neuroimaging abnormalities, etc.

Date: Started June 12, 2018 – Ended July 8, 2018

Zika search, Medline (through Ovid):

1. exp Zika Virus Infection/ or exp ZIKA VIRUS/
2. (zika or ZIKV).ti,ab,kf.
3. 1 or 2
4. exp Pregnancy/ or exp Maternal Exposure/ or exp "Embryonic and Fetal Development"/ or exp "Congenital, Hereditary, and Neonatal Diseases and Abnormalities"/ or exp Infant/ or exp Child/
5. (pregnan* or matern* or gestation* or perinatal* or birth* or congenital* or newborn* or fetal or fetus* or foetal or foetus* or neonat* or infan* or toddler* or child*).ti,ab,kf.
6. 4 or 5
7. 3 and 6
8. 7 not (exp Animals/ not exp Humans/)

☐	1	exp Zika Virus Infection/ or exp ZIKA VIRUS/	2407
☐	2	(zika or ZIKV).ti,ab,kf.	4672
☐	3	1 or 2	4784
☐	4	exp Pregnancy/ or exp Maternal Exposure/ or exp "Embryonic and Fetal Development"/ or exp "Congenital, Hereditary, and Neonatal Diseases and Abnormalities"/ or exp Infant/ or exp Child/	3661254
☐	5	(pregnan* or matern* or gestation* or perinatal* or birth* or congenital* or newborn* or fetal or fetus* or foetal or foetus* or neonat* or infan* or toddler* or child*).ti,ab,kf.	2574479
☐	6	4 or 5	4373079
☐	7	3 and 6	1947
☐	8	7 not (exp Animals/ not exp Humans/)	1857

Zika search, Embase (through Ovid):

1. exp Zika virus/ or exp Zika fever/
2. (zika or ZIKV).ti,ab,kw.
3. 1 or 2
4. exp pregnancy/ or exp pregnancy outcome/ or exp high risk pregnancy/ or exp pregnancy complication/ or exp maternal exposure/ or exp fetus/ or exp "functions of embryonic, fetal and placental structures"/ or exp Infant/ or exp infant disease/ or exp child/ or exp childhood disease/
5. (pregnan* or matern* or gestation* or perinatal* or birth* or congenital* or newborn* or fetal or fetus* or foetal or foetus* or neonat* or infan* or toddler* or child*).ti,ab,kw.
6. 4 or 5

Carabali M, *et al. BMJ Open* 2022; 12:e064362. doi: 10.1136/bmjopen-2022-064362

7. 3 and 6
8. 7 not ((exp animal/ or exp nonhuman/) not exp human/)

# ▲	Searches	Results
1	exp Zika virus/ or exp Zika fever/	4757
2	(zika or ZIKV).ti,ab,kw.	5183
3	1 or 2	5788
4	exp pregnancy/ or exp pregnancy outcome/ or exp high risk pregnancy/ or exp pregnancy complication/ or exp maternal exposure/ or exp fetus/ or exp "functions of embryonic, fetal and placental structures"/ or exp Infant/ or exp infant disease/ or exp child/ or exp childhood disease/	4556497
5	(pregnan* or matern* or gestation* or perinatal* or birth* or congenital* or newborn* or fetal or fetus* or foetal or foetus* or neonat* or infan* or toddler* or child*).ti,ab,kw.	3374208
6	4 or 5	5355248
7	3 and 6	2675
8	7 not ((exp animal/ or exp nonhuman/) not exp human/)	2326

II. SCREENING, ELIGIBILITY AND SELECTION OF STUDIES TRHOUGH SYSTEMATIC SEARCH

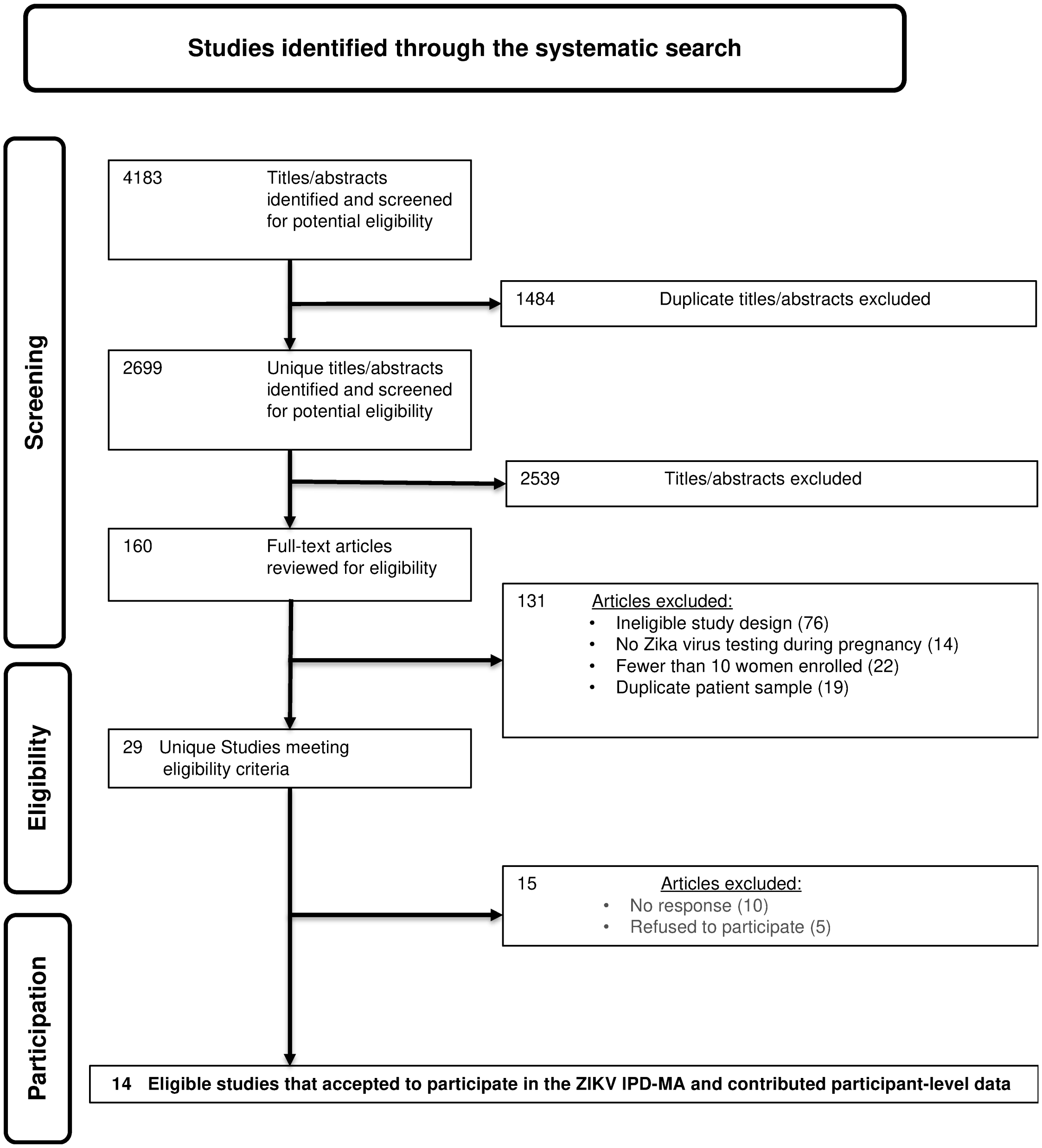

Figure S.1. Flowchart for study participation through systematic search.

III. SUPPLEMENTARY TABLES WITH DESCRIPTIVE ZIKV-IPD-MA METADA-RELATED STATISITCS AND FIGURES

Table S1. Detailed description of ZIKV-IPD-MA studies participating in the metadata survey (Part 1).

ID	Study Name	Country	Source population	Pregnancy status (Enrollment)	Enrollment and ZIKV status	Study Design	Planned follow-up pregnant women	Planned follow-up child	All Women	ZIKV (+) Women	Child ren	Funding
1	Bolivia_Roca_ZIKAlliance	Bolivia	Healthcare Facility	Pregnant	Unrelated to ZIKV	Cohort	9 visits conducted every month during pregnancy for both ZIKV+ and ZIKV- women	-	74	15	30	European Union
2	Brazil_Bahia_Ko_NIHNIAD	Brazil	Healthcare Facility	Pregnant	Unrelated to ZIKV	Cohort	9 visits in total for ZIKV+ and ZIKV- pregnant women, every 4 weeks during pregnancy, at delivery, and 6 weeks postpartum. Additional visits were also conducted for women with acute ZIKA-like symptoms.	4 visits at birth, 3, 6, and 12 months of age for infants of both ZIKV+ and ZIKV- pregnant women. The age window for the final visit could be extended up to 18 months.	600	0	509	NIH/NIAID and FIOCRUZ
3	Brazil_BahiaPaudaLima_Costa	Brazil	Community	Pregnant	Unrelated to ZIKV	Cohort	1 visit anytime during pregnancy for both ZIKV+ and ZIKV- women	1 visit anytime between 11-32 months of age for infants of both ZIKV+ and ZIKV- pregnant women	46	9	46	NIH/NIAID, Wellcome Trust, and Bahia State Research Support Foundation (FAPESB)
4	Brazil_BahiaSalvador_Costa	Brazil	Healthcare Facility	Delivered	Unrelated to ZIKV	Cohort	- (enrolled delivered women)	2 visits between 24-36 months of age and then between 30-42 months of age for infants of ZIKV+ and ZIKV- women	157	110	154	NIH/NIAID, Wellcome Trust, and Bahia State Research Support Foundation (FAPESB) ZIKA-FAPESB T.O. no PET0021/2016 (www.fapesb.ba.gov.br) and Coordination for the Improvement of Higher Education (CAPES) from Brazil (https://www.capes.gov.br/)
5	Brazil_BahiaSalvador_Siqueira_czs	Brazil	Healthcare Facility	Children only	Symptoms or presence of exposure	Surveillance	- (enrolled only infants)	One visit during the first week of life for infants of women with ZIKV symptoms during pregnancy	0	0	151	WHO, CNPq (Brazil), and Fapesb (Brazil)
6	Brazil_BahiaSalvador_Siqueira_zikvsymp	Brazil	Healthcare Facility	Pregnant and delivered	Symptoms or presence of exposure	Surveillance	-	One visit during the first week of life for infants of women with ZIKV symptoms during pregnancy	101	73	102	WHO, CNPq (Brazil), and Fapesb (Brazil)
7	Brazil_Brasil_ZIKAlliance	Brazil	Healthcare Facility	Pregnant	Unrelated to ZIKV	Cohort	9 visits conducted every month during pregnancy for both ZIKV+ and ZIKV- women	6 visits at week 4, and months 4, 9, 12, 18, and 24 (final age of folllow-up) for infants of both ZIKV+ and ZIKV- pregnant women	200	-	39	European Union

8	Brazil_Goias_Turchi	Brazil	Community & Healthcare Facility	Pregnant	Laboratory confirmation/ Symptoms or Presence of Exposure	Cohort	7 visits anytime during prenatal care and after birth for both ZIKV+ and ZIKV- women	6 visits at months 1, 3, 6, 12, 18 (final age of follow-up) for infants of both ZIKV+ and ZIKV- pregnant women	87	58	69	European Union and FAPEG - Fundacion de Amparo a Pesquisa do Estado de Goias
9	Brazil_Maranhao_Silva	Brazil	Healthcare Facility	Children only	Laboratory confirmation/ Symptoms or Presence of Exposure	Cohort	-	Two visits per year, at 6-month intervals, upto 3 years of age (originally planned upto 4 years of age) for infants of ZIKV+ pregnant women	0	0	110	CNPq (Brazilian Research Council), CAPES (Coordination for the Improvement of Higher Education Personnel, FAPEMA (Maranhao State Research Foundation) and Science and Technology Department, Ministry of Health, Brazil
10	Brazil_MatoGrosso_SchulerFaccini	Brazil	Community	Delivered	Laboratory confirmation/ Symptoms or Presence of Exposure	Case cohort	- (enrolled delivered women)	Visits at 6, 12, 18, 24 months and then once a year up to 5 years of age for infants of ZIKV+ pregnant women	50	50	47	National Institute of Population Medical Genetics (INaGeMP)
11	Brazil_Para_Oliveira	Brazil	Community & Healthcare Facility	Pregnant	Laboratory confirmation	Cohort	4 visits every month for ZIKV+ pregnant women. ZIKV- pregnant women not enrolled.	-	134	109	102	-
12	Brazil_Pernambuco_PerezCoelho	Brazil	Community & Healthcare Facility	Children only	Laboratory confirmation/ Symptoms or Presence of Exposure	Cohort	- (enrolled only infants)	-	0	0	130	-
13	Brazil_Pernambuco_Ximenes	Brazil	Community & Healthcare Facility	Pregnant	Laboratory confirmation/ Symptoms or Presence of Exposure	Cohort	3 visits during pregnancy and after a livebirth for ZIKV+ pregnant women. ZIKV- pregnant women not enrolled.	-	700	700	600	European Union, PAHO, NIH/NIAID, Wellcome Trust, CnPq; Capes; Decit; Facepe; Secretaria de Vigilancia em Saude - Ministerio da Saude - Brasil; ERAES; Medical Research Council.
14	Brazil_RdJ_Moreira_ZIP	Brazil	Healthcare Facility	Pregnant	Unrelated to ZIKV	Cohort	6 monthly visits throughout pregnancy for both ZIKV+ and ZIKV- pregnant women	4 visits at birth, 3, 6, and 12 months (final age of follow-up) for both infants of ZIKV+ and ZIKV- pregnant women	771	17	599	NIH/NIAID

15	Brazil_RibieroPrietro_Duarte	Brazil	Community & Healthcare Facility	Pregnant	Laboratory confirmation	Cohort	For ZIKV+ pregnant women, visits were conducted monthly, however the number of visits varied according to the gestational age of inclusion into the study. ZIKV- pregnant women not enrolled.	Visits at birth, and months 1, 3, 6, 12, 18, and 24 (final age of follow-up) for infants of ZIKV+ pregnant women	511	511	487	Mainly supported by Fundacio de Apoio ao Ensino Pesquisa e Assistencia do Hospital das Clinicas da Faculdade de Medicina de Ribeirio Preto da Universidade de Sao Paulo (FAEPA), Brasil, and received partial support from the CVE - Centro de Vigilancia Epidemiologica 'Prof. Alexandre Vranjac' da Secretaria da Saude do Estado de Sao Paulo (SES-SP).
16	Brazil_RiodeJaneiro_Brasil	Brazil	Community	Pregnant	Laboratory confirmation/ Symptoms or Presence of Exposure	Cohort	-	-	400	244	216	Brazilian Ministry of Health (DECIT), NIH and Fondation Merieux
17	Brazil_RiodeJaneiro_Cunha	Brazil	Healthcare Facility	Reproductive age	Laboratory confirmation/ Symptoms or Presence of Exposure	Cohort	8 visits, one per month during pregnancy, for both ZIKV+ and ZIKV- women	11 visits at months 1-6, 12, 18, 24, 30, 36 (final age of follow-up) for infants of ZIKV+ pregnant women. Infants of ZIKV- pregnant women were not followed.	118	118	54	FAPERJ
18	Brazil_RiodeJaneiro_Hofer	Brazil	Healthcare Facility	Pregnant	Laboratory confirmation/ Symptoms or Presence of Exposure	Cohort	1 visit every trimester for both ZIKV+ and ZIKV- women	12 visits at months 1-6, 8, 10, 12, 18, 24 for infants of both ZIKV+ and ZIKV- pregnant women	51	38	43	FAPERJ
19	Brazil_RiodeJaneiro_Joao	Brazil	Community	Pregnant	Laboratory confirmation	Cohort	6 prenatal visits for both ZIKV+ and ZIKV- pregnant women	1 visit at 6 months of age for infants of both ZIKV+ and ZIKV- pregnant women	219	34	221	No funding
20	Brazil_RiodeJaneiro_Moreira	Brazil	Healthcare Facility	Pregnant	Laboratory confirmation/ Symptoms or Presence of Exposure	Cohort	8 visits conducted every month for ZIKV+ pregnant women (also depends on time of ZIKV infection during pregnancy). 8 visits also conducted every month for ZIKV- pregnant women.	8 visits in total each at 3, 6, 9, 12, 18, 24, 30 and 36 months of age (excluding visit at birth). Further follow-up is planned from 36-60 months.	294	294	296	European Union, Wellcome Trust, CNPq and Faperj and PIP/Fiocruz (Brazilian governamental agencies)
21	Brazil_SaoPaulo_DuartePassos	Brazil	Healthcare Facility	Pregnant	Unrelated to ZIKV	Cohort	Unspecified number of visits* *According to medical judgement	Monthly up to 6 months of age; every 3 months up to 12 months of age; every 6 months up to 3 years of age	690	57	696	Brazilian Ministry of Health, London School of Hygiene and Tropical Medicine and FAPESP
22	Brazil_SaoPaulo_LacerdaNoguiera	Brazil	Healthcare Facility	Pregnant	Laboratory confirmation	Cohort	1 visit at enrollment and then 1 visit every trimester for ZIKV+ women. ZIKV- pregnant women not enrolled	-	54	54	54	FAPESP

23	Brazil_Segurado_ ZIKAlliance	Brazil	Healthcare Facility	Pregnant	Unrelated to ZIKV	Cohort	9 visits conducted every month during pregnancy for both ZIKV+ and ZIKV- women	6 visits at week 4, and months 4, 9, 12, 18, and 24 (final age of follow-up) for infants of both ZIKV+ and ZIKV- pregnant women	106	0	48	European Union
24	Brazil_SP_Mussi_ ZIP	Brazil	Community & Healthcare Facility	Pregnant	Unrelated to ZIKV	Cohort	On average 12 visits before delivery, 1 visit at delivery and 1 visit 6 weeks after delivery for both ZIKV+ and ZIKV- women	4 visits at birth, 3, 6, and 12 months (final age of follow-up) for both infants of ZIKV+ and ZIKV- pregnant women	554	0	418	NIH/NIAID
25	Colombia_Arias_ ZIP	Colombia	Healthcare Facility	Pregnant	Unrelated to ZIKV	Cohort	9 visits at enrollment, monthly thereafter, and six weeks post partum for both ZIKV+ and ZIKV- women	4 visits at birth, 3-5 months, 6-11 months, 12-18 months for both infants of ZIKV+ and ZIKV- pregnant women	435	3	206	NIH/NIAID
26	Colombia_Becerr a	Colombia	Healthcare Facility	Pregnant	Laboratory confirmation/ Symptoms or Presence of Exposure	Cohort	Monthly visits throughout pregnancy for both ZIKV+ and ZIKV- pregnant women	4 visits at months 6, 12, 18, 24 (final age of follow-up), for infants of both ZIKV+ and ZIKV- pregnant women	243	124	226	COLCIENCIAS Universidad Industrial De Santander Ascon Hospital Universitario De Santander
27	Colombia_Gilboa	Colombia	Healthcare Facility	Pregnant	Unrelated to ZIKV	Cohort	Both ZIKV+ and ZIKV- women had 14-16 visits if they were enrolled early in the first trimester and had a full-term pregnancy. If not, the visits were fewer. There was also one postpartum visit.	Some infants were only followed up until 6 months of age with visits at delivery, 10 days of age, and thereafter every two weeks. Other infants were followed up until 18 months with additional visits at 9, 12, and 18 months of age	1519	16	1239	US CDC, USAID, and Instituto Nacional de Salud (Colombia)
28	Colombia_Lopez Medina	Colombia	Community	Pregnant	Laboratory confirmation	Cohort	- (only followed up with infants)	Visits every 3-4 months until 18 months (final age of follow-up)	170	170	154	Thrasher Foundation
29	Colombia_Mattar	Colombia	Community	Delivered	Symptoms or presence of exposure	Surveil lance	8 visits during every trimester for both ZIKV+ and ZIKV- women	4 visits at 6, 12, 18, 24 months (final age of follow-up) for infants of both ZIKV+ and ZIKV- pregnant women	11	11	11	WHO
30	Colombia_Mercad oReyes	Colombia	Healthcare Facility	Pregnant	Laboratory confirmation/ Symptoms or Presence of Exposure	Surveil lance	14 visits across pregnancy for both ZIKV+ and ZIKV- women	Study did not dictate number of follow-ups, but data that were available in medical records were abstracted up to 2 years of age for infants of both ZIKV+ and ZIKv- pregnant women	1218	253	1185	US CDC
31	Colombia_Mulke y	Colombia	Community + Travelers	Pregnant	Symptoms or presence of exposure	Cohort	2 visits if the ZIKV+ pregnant woman is enrolled prior to 32 weeks of gestation. ZIKV- pregnant women not enrolled	2 visits at 6 months and between 12-18 months for infants of ZIKV+ pregnant women	82	82	80	Ikaria Fund; Thrasher Research Fund; NIH

Carabali M, *et al. BMJ Open* 2022; 12:e064362. doi: 10.1136/bmjopen-2022-064362

32	Colombia_SanzCortes	Colombia	Healthcare Facility	Pregnant	Laboratory confirmation/ Symptoms or Presence of Exposure	Cohort	An average of 5 monthly visits for ZIKV+ pregnant women. ZIKV- pregnant women not enrolled	An average of 5 monthly visits for infants of ZIKV+ pregnant women	17	17	17	No funding
33	Colombia_Villar_ZIKAlliance	Colombia	Healthcare Facility	Pregnant	Unrelated to ZIKV	Cohort	9 visits conducted every month during pregnancy for both ZIKV+ and ZIKV- women	6 visits at week 4, and months 4, 9, 12, 18, and 24 (final age of follow-up) for infants of both ZIKV+ and ZIKV- pregnant women	590	20	473	European Union
34	Ecuador_Soria_ZIKAlliance	Ecuador	Healthcare Facility	Pregnant	Unrelated to ZIKV	Cohort	9 visits conducted every month during pregnancy for both ZIKV+ and ZIKV- women	6 visits at week 4, and months 4, 9, 12, 18, and 24 (final age of folllow-up) for infants of both ZIKV+ and ZIKV- pregnant women	436	436	151	European Union
35	FrenchGuiana_Pomar	France	Healthcare Facility	Pregnant	Laboratory confirmation	Cohort	Monthly visits were conducted after inclusion for both ZIKV+ and ZIKV- pregnant women, capped at a maximum of 6 visits, as inclusion occured mainly after the results of the first serology performed during the first trimester	1 visit at 3 days post-partum for infants of both ZIKV+ and ZIKV- pregnant women	700	301	305	-
36	Grenada_LaBeaud	Grenada	Healthcare Facility	Pregnant and delivered	Symptoms or presence of exposure	Cohort	One prenatal and one postnatal visit for both ZIKV+ and ZIKV- women	Variable number of visits at variable ages upto a maximum of 30 months of age for both infants of ZIKV+ and ZIKV- pregnant women	384	113	388	NIH/NIAID, USAID, National Institutes of Health Eunice Kennedy Shriver, National Institute of Child Health and Human Development, Windward Islands Research and Education Foundation, Stanford Maternal Child Health Research Institute
37	Guadaloupe_Cabie	France	Healthcare Facility	Pregnant	Laboratory confirmation/ Symptoms or Presence of Exposure	Cohort	8 visits in total, one every month during pregnancy, for ZIKV+ women<br>4 visits in total, one every trimester and one at delivery, for ZIKV- women	5 visits at 2, 4, 9, 18, 24 (final age of follow-up) months for infants of both ZIKV+ and ZIKV- pregnant women	4959	1564	1180	French Ministry of Health
38	Guatemala_EspinosaBode_ZINC	Guatemala	Healthcare Facility	Pregnant	Unrelated to ZIKV	Cohort	15 visits at recruitment (if <20 weeks), 26, and 32 weeks, as well as at birth	2 visits at birth and at 3 months (final age of follow-up) for both infants of ZIKV+ and ZIKV- pregnant women	436	0	389	US CDC

39	Guatemala_Figueroa_ZIP	Guatemala	Community & Healthcare Facility	Pregnant	Unrelated to ZIKV	Cohort	10 monthly visits across pregnancy for both ZIKV+ and ZIKV- women	4 visits at birth, 3, 6, and 12 months (final age of follow-up) for both infants of ZIKV+ and ZIKV- pregnant women	998	0	722	NIH/NIAID
40	HaitiJamaica_ZIKAction	Haiti and Jamaica	Healthcare Facility	Pregnant	Unrelated to ZIKV	Cohort	4 visits at enrollment, 20, 28, and 36 weeks for both ZIKV+ and ZIKV- pregnant women. Extra visits were conducted if the woman was ill.	6 visits at 1, 4, 9, 12, 18, 24 (final age of follow-up) months for infants of both ZIKV+ and ZIKV- pregnant women	1147	-	-	European Union
41	Honduras_Alger	Honduras	Healthcare Facility	Pregnant	Unrelated to ZIKV	Cohort	1 visit upon enrollment for both ZIKV+ and ZIKV- pregnant women	1 visit for infants of both ZIKV+ and ZIKV- pregnant women	3995	7	645	US CDC and School of Public Health and Tropical Medicine, Tulane University
42	Kenya_Widdowson	Kenya	Healthcare Facility	Pregnant	Symptoms or presence of exposure	Cohort	10 monthly visits for ZIKV+ and ZIKV- pregnant women	-	2312	3	0	US CDC
43	Mexico_Alpuche_ZIKAlliance	Mexico	Healthcare Facility	Pregnant	Unrelated to ZIKV	Cohort	7 visits conducted every month during pregnancy for both ZIKV+ and ZIKV- women	6 visits at week 4, and months 4, 9, 12, 18, and 24 (final age of folllow-up) for infants of both ZIKV+ and ZIKV- pregnant women	-	-	-	European Union
44	Mexico_Borja_ZIKAlliance	Mexico	Healthcare Facility	Pregnant	Unrelated to ZIKV	Cohort	8 visits at 6-8, 10-13, 16-18, 22, 28, 32, 36, and 38-41 weeks for both ZIKV+ and ZIKV- pregnant women	4 visits at week 4, and months 4, 12, and 24 (final age of follow-up) for infants of both ZIKV+ and ZIKV- pregnant women	1144	63	657	European Union
45	Nicaragua_Balmaseda_ZIP	Nicaragua	Healthcare Facility	Pregnant	Unrelated to ZIKV	Cohort	On average 10 visits every month during pregnancy, 1 at delivery and 1 visit 6 weeks after delivery for both ZIKV+ and ZIKV- women	4 visits at birth, 3, 6, and 12 months (final age of follow-up) for both infants of ZIKV+ and ZIKV- pregnant women	722	0	-	NIH/NIAID
46	Peru_Gotuzzo_ZIKAlliance	Peru	Healthcare Facility	Pregnant	Unrelated to ZIKV	Cohort	9 visits conducted every month during pregnancy for both ZIKV+ and ZIKV- women	6 visits at week 4, and months 4, 9, 12, 18, and 24 (final age of folllow-up) for infants of both ZIKV+ and ZIKV- pregnant women	153	83	153	European Union
47	PuertoRico_ValenciaPrado_ZAPSS	Puerto Rico	Community & Healthcare Facility	Pregnant and delivered	Laboratory confirmation	Surveillance	-	-	4280	4280	4014	US CDC
48	Singapore_Chan	Singapore	Healthcare Facility	Pregnant	Laboratory confirmation/ Symptoms or Presence of Exposure	Cohort	7 visits every 4-6 weeks during pregnancy for ZIKV+ women. ZIKV- pregnant women were not enrolled.	-	15	15	18	Ministry of Health, Singapore KK Women's and Children's Hospital

49	Spain_Bardaji	Spain	Healthcare Facility	Pregnant	Laboratory confirmation/ Symptoms or Presence of Exposure	Surveil lance	Monthly follow-ups for ZIKV+ women throughout pregnancy. ZIKV- women were also followed monthly until a negative diganosis, after which they were referred to the closest healthcare centre for follow-up	7 visits at birth, and at months 1, 2, 6, 12, 18 and 24 (final age of follow up) for children of ZIKV+ pregnant women. Children of ZIKV- pregnant women were not followed	195	44	42	The Government of Spain, Fondo de Investigación en Salud (FIS), Instituto de Salud Carlos III.
50	Spain_Soriano	Spain	Travelers	Pregnant	Symptoms or presence of exposure	Cohort	At lesst one visit per trimester for ZIKV+ pregnant women. ZIKV- pregnant women not enrolled.	Visits scheduled at 1, 4, 9, 12, 18, 24 and 36 months of age for children of ZIKV+ pregnant women	186	186	213	European Union
51	Suriname_Juliana	Suriname	Healthcare Facility	Delivered	Laboratory confirmation/ Symptoms or Presence of Exposure	Surveil lance	2 visits for ZIKV+ women with one during pregnacy and another after. ZIKV- pregnant women were not enrolled	Visits at 3, 6, 9, and 12 months for infants with evidence of congential ZIKV syndrome. After this, children seen on an as-needed basis. Another complete assessment was conducted at 4 years of age	22	16	22	No funding
52	TrinidadTobago_ Sohan	Trinidad and Tobago	Healthcare Facility	Pregnant	Laboratory confirmation	Cohort	12 visits that occur every month until 26 weeks and then every 3 weeks until 36 weeks for ZIKV+ pregnant women. More visits can occur if abnormalities are detected<br>2 visits that occur during the trimester and then during the second or third trimester for ZIKV- pregnant women	-	100	100	0	-
53	USA_Mulkey	USA	Healthcare Facility	Pregnant	Symptoms or presence of exposure	Cohort	1 visit for ZIKV+ and ZIKV- pregnant women usually after 18 weeks of gestation	1-5 visits at birth and 3, 6, 12, and 18 months	72	29	42	Ikaria Fund; Thrasher Research Fund; NIH
54	USA_USZPIR	USA	Community + Travelers	Pregnant and delivered	Laboratory confirmation	Surveil lance	Medical records used for ZIKV+ pregnant women. Maternal data was collected at time of identification and then each trimester following. ZIKV- pregnant women not enrolled	Data on infants collected at birth and then at 2, 6, 12, 18, 24, 30, and 36 months	603	603	528	US CDC

ZIKV: Zika Virus; DENV: Dengue Virus; CHIKV: Chikungunya Virus; ZIKV(+): ZIKV positive individuals;

Note: Study Name convention: Country_City/PIName_StudyConsortium (if multisite)

Carabali M, *et al. BMJ Open* 2022; 12:e064362. doi: 10.1136/bmjopen-2022-064362

Table S1. Detailed description of ZIKV-IPD-MA studies participating in the metadata survey (Part 2).

ID	Study Name	Maternal ZIKV assessment	Fetal ZIKV assessment	Infant ZIKV assessment	Maternal PCR	Maternal PRNT	Fetal PCR	Prenatal microcephaly definition	Postnatal microcephaly assessment	Identification of CZS malformations	CZS Definition	DENV testing	CHIKV Testing
1	Bolivia_Roca_ZIK Alliance	Laboratory testing	Maternal clinical/laboratory criteria, fetal laboratory criteria	Infant clinical/laboratory criteria	Yes	No	Yes	-	No*	Peri/intrapartum and postpartum	WHO	Yes	Yes
2	Brazil_Bahia_Ko_NIHNIAD	Laboratory testing	-	Infant clinical/laboratory, imaging, and maternal clinical/laboratory criteria	Yes	No	No	2 SD below the mean	Yes	Antenatal and peri/intrapartum and postpartum	WHO	Yes	Yes
3	Brazil_BahiaPaudaLima_Costa	Laboratory testing	-	-	No	No	No	-	Yes	Postpartum	Study definition	No	No
4	Brazil_BahiaSalvador_Costa	Laboratory testing	-	Infant clinical/laboratory, imaging and maternal clinical/laboratory criteria	No	Yes	No	-	Yes	Postpartum	Study definition	No	No
5	Brazil_BahiaSalvador_Siqueira_czs	-	Placental laboratory criteria	Infant clinical/laboratory, imaging and maternal clinical/laboratory criteria	No	No	No	2 SD below the mean	Yes	Postpartum	Country	No	No
6	Brazil_BahiaSalvador_Siqueira_zikvsymp	Clinical case definition and/or Laboratory confirmation	Placental laboratory criteria	Infant clinical/laboratory, imaging and maternal clinical/laboratory criteria	No	No	No	2 SD below the mean	Yes	Postpartum	Country	Yes	Yes
7	Brazil_Brasil_ZIK Alliance	Laboratory testing	-	Infant clinical/laboratory, imaging and maternal clinical/laboratory criteria	Yes	Yes	No	2 SD below the mean	No*	-	-	Yes	Yes
8	Brazil_Goias_Turchi	Laboratory testing	Fetal imaging criteria	Infant clinical/laboratory criteria	Yes	No	No	2 SD below the mean	Yes	Postpartum	Country	Yes	Yes
9	Brazil_Maranhao_Silva	Clinical case definition	-	Infant clinical/laboratory, imaging and maternal clinical/laboratory criteria	No	No	No	-	Yes	Postpartum	Study definition	No	No
10	Brazil_MatoGrosso_SchulerFaccini	Clinical case definition and/or Laboratory confirmation	-	-	Yes	No	No	-	Yes	Peri/intrapartum	Country	No	No

Carabali M, *et al. BMJ Open* 2022; 12:e064362. doi: 10.1136/bmjopen-2022-064362

11	Brazil_Para_Oliveira	Laboratory testing	Maternal clinical/laboratory criteria and placental laboratory criteria	Maternal clinical/laboratory criteria	Yes	Yes	No	Below the 5th percentile	Yes	Peri/intrapartum and postpartum	Country	Yes	Yes
12	Brazil_Pernambuco_PerezCoelho	-	-	Infant clinical/laboratory and infant imaging criteria	No	No	No	-	Yes	Postpartum	Country	No	No
13	Brazil_Pernambuco_Ximenes	Laboratory testing	-	Infant clinical/laboratory, imaging and maternal clinical/laboratory criteria	Yes	Yes	No	-	Yes	Peri/intrapartum and postpartum	Study definition	Yes	Yes
14	Brazil_RdJ_Moreira_ZIP	Laboratory testing	Maternal clinical/laboratory, fetal imaging, laboratory and placental criteria	Infant clinical/laboratory, imaging and maternal clinical/laboratory criteria	Yes	No	Yes	2 SD below the mean	Yes	Antenatal and peri/intrapartum and postpartum	WHO	Yes	Yes
15	Brazil_RibieroPrietro_Duarte	Laboratory testing	Fetal imaging, laboratory and placental criteria	Infant clinical/laboratory criteria and maternal clinical/laboratory criteria	Yes	No	Yes	Below the 3rd centile	Yes	Postpartum	Country	No	No
16	Brazil_RiodeJaneiro_Brasil	Laboratory testing	Maternal clinical/laboratory, fetal imaging and laboratory criteria	Infant clinical/laboratory and infant imaging criteria	Yes	No	Yes	3 SD below the mean	Yes	Antenatal and postpartum	Study definition	Yes	Yes
17	Brazil_RiodeJaneiro_Cunha	Clinical case definition and/or Laboratory confirmation	Placental laboratory criteria	Infant clinical/laboratory, imaging and maternal clinical/laboratory criteria	Yes	No	No	2 SD below the mean	Yes	Peri/intrapartum	Country	Yes	Yes
18	Brazil_RiodeJaneiro_Hofer	Laboratory testing	-	Infant clinical/laboratory, imaging and maternal clinical/laboratory criteria	Yes	No	No	2 SD below the mean	Yes	Postpartum	Country	Yes	No
19	Brazil_RiodeJaneiro_Joao	Laboratory testing	-	-	Yes	Yes	No	-	Yes	Antenatal and peri/intrapartum and postpartum	Country	Yes	Yes
20	Brazil_RiodeJaneiro_Moreira	Clinical case definition and/or Laboratory confirmation	Maternal clinical/laboratory, fetal imaging, laboratory and placental criteria	Infant clinical/laboratory, imaging and maternal clinical/laboratory criteria	Yes	No	Yes	3 SD below the mean	Yes	Antenatal and peri/intrapartum and postpartum	WHO	Yes	Yes

Carabali M, *et al. BMJ Open* 2022; 12:e064362. doi: 10.1136/bmjopen-2022-064362

21	Brazil_SaoPaulo_DuartePassos	Laboratory testing	-	Infant clinical/laboratory and infant imaging criteria	Yes	No	No	2 SD below the mean	Yes	Antenatal and postpartum	CDC/WHO/Country	Yes	Yes
22	Brazil_SaoPaulo_LacerdaNoguiera	Laboratory testing	-	Infant clinical/laboratory criteria	Yes	No	No	-	Yes	Antenatal and postpartum	Study definition	Yes	No
23	Brazil_Segurado_ZIKAlliance	Laboratory testing	Maternal clinical/laboratory, fetal imaging, laboratory and placental criteria	-	Yes	No	Yes	3 SD below the mean	Yes	Postpartum	Study definition	No	Yes
24	Brazil_SP_Mussi_ZIP	Laboratory testing	-	-	Yes	No	No	2 SD below the mean	Yes	Antenatal and peri/intrapartum and postpartum	WHO	Yes	Yes
25	Colombia_Arias_ZIP	Laboratory testing	Fetal laboratory criteria	Infant clinical/laboratory criteria	Yes	No	Yes	3 SD below the mean	Yes	-	-	Yes	Yes
26	Colombia_Becerra	Laboratory testing	Maternal clinical/laboratory, fetal imaging, laboratory and placental criteria	Infant clinical/laboratory criteria and maternal clinical/laboratory criteria	Yes	No	Yes	2 SD below the mean	Yes	Antenatal and peri/intrapartum and postpartum	WHO	Yes	No
27	Colombia_Gilboa	Laboratory testing	Fetal laboratory criteria	Infant clinical/laboratory criteria	Yes	No	Yes	Other	Yes	Antenatal and postpartum	CDC	Yes	Yes
28	Colombia_Lopez Medina	Laboratory testing	-	-	Yes	No	No	2 SD below the mean	Yes	-	-	No	No
29	Colombia_Mattar	Laboratory testing	-	-	Yes	No	No	2 SD below the mean	Yes	Antenatal and peri/intrapartum	Country	No	Yes
30	Colombia_MercadoReyes	Clinical case definition and/or Laboratory confirmation	Maternal clinical/laboratory, fetal imaging, laboratory and placental criteria	Infant clinical/laboratory, imaging and maternal clinical/laboratory criteria	Yes	No	No	3 SD below the mean	Yes	Antenatal and peri/intrapartum and postpartum	Study definition	Yes	Yes
31	Colombia_Mulkey	Clinical case definition and/or Laboratory confirmation	-	Maternal clinical/laboratory criteria	Yes	Yes	No	3 SD below the mean	Yes	Antenatal and postpartum	Study definition	Yes	No

Carabali M, *et al. BMJ Open* 2022; 12:e064362. doi: 10.1136/bmjopen-2022-064362

32	Colombia_SanzCortes	Laboratory testing	Maternal clinical/laboratory, fetal imaging, laboratory and placental criteria	Infant clinical/laboratory, imaging and maternal clinical/laboratory criteria	Yes	No	Yes	3 SD below the mean	Yes	Antenatal and peri/intrapartum and postpartum	Country	No	No
33	Colombia_Villar_ZIKAlliance	Laboratory testing	-	-	Yes	No	No	-	Yes	Antenatal and peri/intrapartum and postpartum	WHO	Yes	Yes
34	Ecuador_Soria_ZIKAlliance	Laboratory testing	Fetal imaging, laboratory and placental criteria	Infant clinical/laboratory criteria	Yes	No	Yes	2 SD below the mean	Yes	Postpartum	Country	No	No
35	FrenchGuiana_Pomar	Laboratory testing	Fetal imaging, laboratory and placental criteria	Infant clinical/laboratory criteria	Yes	Yes	Yes	2 SD below the mean	No*	Antenatal and postpartum	Study definition	Yes	Yes
36	Grenada_LaBeaud	Laboratory testing	-	Infant clinical/laboratory criteria	Yes	No	No	-	Yes	Postpartum	WHO	Yes	Yes
37	Guadaloupe_Cabie	Laboratory testing	Placental laboratory criteria	Infant clinical/laboratory criteria and maternal clinical/laboratory criteria	Yes	No	No	2 SD below the mean	Yes	Antenatal and peri/intrapartum and postpartum	Study definition	No	No
38	Guatemala_EspinosaBode_ZINC	Clinical case definition and/or Laboratory confirmation	-	Infant clinical/laboratory criteria	Yes	No	No	2 SD below the mean	Yes	Postpartum	CDC	Yes	Yes
39	Guatemala_Figueroa_ZIP	Laboratory testing	Maternal clinical/laboratory criteria	Infant clinical/laboratory criteria	Yes	No	No	2 SD below the mean	Yes	-	-	Yes	No
40	HaitiJamaica_ZIKAction	Laboratory testing	-	Infant clinical/laboratory and infant imaging criteria	Yes	Yes	No	2 SD below the mean	No*	Antenatal and postpartum	Study definition	Yes	Yes
41	Honduras_Alger	Laboratory testing	-	-	Yes	No	No	-	Yes	-	-	Yes	-
42	Kenya_Widdowson	Clinical case definition and/or Laboratory confirmation	Maternal clinical/laboratory, fetal laboratory, and placental criteria	Infant clinical/laboratory criteria	Yes	Yes	Yes	-	No*	Postpartum	Intergrowth	Yes	Yes
43	Mexico_Alpuche_ZIKAlliance	Clinical case definition and/or Laboratory confirmation	-	-	Yes	No	No	-	Yes	Peri/intrapartum and postpartum	-	-	-
44	Mexico_Borja_ZIKAlliance	Laboratory testing	Fetal imaging, laboratory and	Infant clinical/laboratory and infant imaging criteria	Yes	No	Yes	2 SD below the mean	No*	Postpartum	Study definition	Yes	Yes

			placental criteria										
45	Nicaragua_Balmaseda_ZIP	Laboratory testing	-	-	Yes	No	-	-	-	-	-	-	-
46	Peru_Gotuzzo_ZIKAlliance	Laboratory testing	Fetal imaging, laboratory and placental criteria	Infant clinical/laboratory and infant imaging criteria	Yes	No	Yes	2 SD below the mean	Yes	Peri/intrapartum and postpartum	Country	No	Yes
47	PuertoRico_ValenciaPrado_ZAPSS	Laboratory testing	Placental laboratory criteria	Infant clinical/laboratory criteria	Yes	No	No	-	Yes	Antenatal and peri/intrapartum and postpartum	Study definition	Yes	Yes
48	Singapore_Chan	Laboratory testing	-	Infant clinical/laboratory criteria	Yes	No	No	2 SD below the mean	Yes	Antenatal and postpartum	Country	No	No
49	Spain_Bardaji	Laboratory testing	Maternal clinical/laboratory, fetal imaging, laboratory and placental criteria	Infant clinical/laboratory, imaging and maternal clinical/laboratory criteria	Yes	No	Yes	2 SD below the mean	Yes	Antenatal and peri/intrapartum and postpartum	WHO	Yes	Yes
50	Spain_Soriano	Clinical case definition and/or Laboratory confirmation	Maternal clinical/laboratory, fetal imaging, laboratory and placental criteria	Infant clinical/laboratory and infant imaging criteria	Yes	Yes	Yes	2 SD below the mean	Yes	Antenatal and peri/intrapartum and postpartum	Country	Yes	Yes
51	Suriname_Juliana	Clinical case definition and/or Laboratory confirmation	-	Infant clinical/laboratory, imaging and maternal clinical/laboratory criteria	Yes	No	No	-	Yes	Postpartum	WHO	No	No
52	TrinidadTobago_Sohan	Clinical case definition and/or Laboratory confirmation	-	-	Yes	No	No	Below the 5th percentile	Yes	-	-	Yes	Yes
53	USA_Mulkey	Clinical case definition and/or Laboratory confirmation	-	Maternal clinical/laboratory criteria	Yes	Yes	No	3 SD below the mean	Yes	Antenatal and peri/intrapartum and postpartum	Study definition	Yes	No
54	USA_USZPIR	Laboratory testing	Fetal laboratory and placental criteria	Infant clinical/laboratory criteria	Yes	Yes	Yes	3 SD below the mean	Yes	Antenatal and peri/intrapartum and postpartum	CDC	No	No

ZIKV: Zika Virus; DENV: Dengue Virus; CHIKV: Chikungunya Virus; ZIKV(+): ZIKV positive individuals;

Carabali M, *et al. BMJ Open* 2022; 12:e064362. doi: 10.1136/bmjopen-2022-064362

Table S2. Infant and child development assessment among ZIKV-IPD-MA study participants of the metadata survey.

**Characteristic**	N = 40[1]
**Age of Developmental Assessment (Months)**	
**Median (IQR)**	24 (18, 30)
**Range**	3, 60
**Developmental assessment tool**	
**ASQ-SE Tool**	21 / 40 (52%)
**BSID Tool**	30 / 40 (75%)
**WIDEA-FS Tool**	2 / 39 (5.1%)
**AIMS Tool**	4 / 40 (10%)
**Other Assessments***	18 / 40 (45%)

[1] N= Total studies; Frequency n/N (row) (%);

Bayley Scales of Infant Development (BSID); Ages & Stages Questionnaires® Social-Emotional, Second Edition (ASQ®-SE-2); Warner Initial Developmental Evaluation of Adaptive and Functional Skills (WIDEA) and four the Alberta Infant Motor Scale (AIMS). Note: developmental assessment was only reported by 40 studies, so the denominator is 40 studies. *Includes INTER-NDA and NEPSY-II

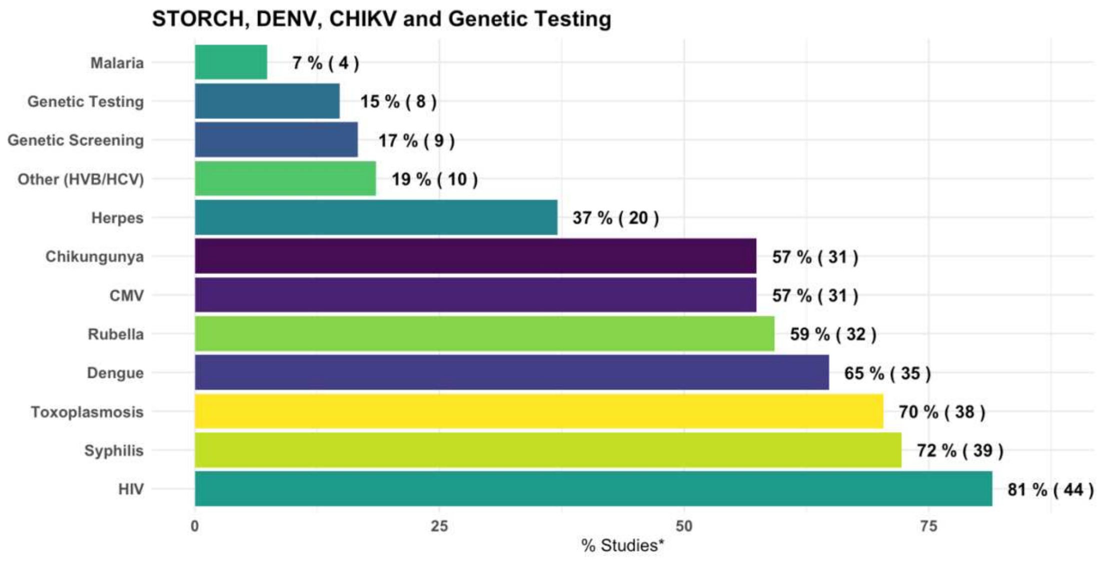

ZIKV: Zika Virus; DENV: Dengue Virus; CHIKV: Chikungunya Virus; HIV: Human Immunodeficiency Virus; CMV: Cytomegalovirus; HVB/HCV: Hepatitis B and Hepatitis C Virus.

Figure S2. STORCH and other additional testing among ZIKV-IPD-MA study participants of the metadata survey. The denominator is total number of studies (n=54)

Carabali M, *et al. BMJ Open* 2022; 12:e064362. doi: 10.1136/bmjopen-2022-064362

Table S3. Sociodemographic and behavioral factors measured among ZIKV-IPD-MA study participants of the metadata survey.

Characteristic	N = 52[1]
**Education**	43 / 52 (83%)
**Occupation**	32 / 52 (62%)
**Marital status**	40 / 52 (77%)
**Nutrition status**	15 / 51 (29%)
**Maternal exposure to workplace or environmental occupational teratogens**	17 / 51 (33%)
**Pre-pregnancy exposure to IPV**	4 / 52 (7.7%)
**Maternal exposure to IPV**	9 / 51 (18%)
**Infant/child exposure to IPV**	7 / 52 (13%)
**Maternal exposure to vaccination**	33 / 51 (65%)
**Maternal prescription drug use**	40 / 51 (78%)
**Maternal recreational drug use**	42 / 51 (82%)
**Maternal ethnic or minority status**	35 / 52 (67%)
**Household SES**	37 / 52 (71%)
**Mental health scales**	4 / 51 (7.8%)
**Maternal smoking**	42 / 51 (82%)
**Maternal alcohol consumption**	41 / 51 (80%)

[1] N= Total studies; Frequency n/N (row) (%)

Note: There were Two studies that did not provide any information about the sociodemographic metadata.

Table S4. Genetic testing, fetal ultrasounds, and MRIs among ZIKV-IPD-MA study participants of the metadata survey

**Characteristic**	**Overall N = 53[1]**	**Other Countries N = 30[1]**	**Brazil N = 23[1]**
**Genetic Screening**	9 / 53 (17%)	7 / 30 (23%)	2 / 23 (8.7%)
**Genetic Testing**	8 / 52 (15%)	6 / 29 (21%)	2 / 23 (8.7%)
**Fetal Ultrasounds**	39 / 53 (74%)	24 / 30 (80%)	15 / 23 (65%)
**Fetal Ultrasound Timing**			
Monthly	7 / 39 (18%)	6 / 24 (25%)	1 / 15 (6.7%)
Once per Trimester	17 / 39 (44%)	6 / 24 (25%)	11 / 15 (73%)
2nd & 3rd Trimester	3 / 39 (7.7%)	3 / 24 (12%)	0 / 15 (0%)
Twice per Pregnancy	3 / 39 (7.7%)	2 / 24 (8.3%)	1 / 15 (6.7%)
Unspecified Timing	9 / 39 (23%)	7 / 24 (29%)	2 / 15 (13%)
**Number of Ultrasounds per Woman**			
At least 3	25 / 39 (64%)	13 / 24 (54%)	12 / 15 (80%)
4 to 6	5 / 39 (13%)	3 / 24 (12%)	2 / 15 (13%)
More than 7	3 / 39 (7.7%)	3 / 24 (12%)	0 / 15 (0%)
Unspecified Number	6 / 39 (15%)	5 / 24 (21%)	1 / 15 (6.7%)
**Postnatal Ultrasound**	36 / 53 (68%)	23 / 30 (77%)	13 / 23 (57%)
**Fetal MRIs**	10 / 52 (19%)	7 / 29 (24%)	3 / 23 (13%)
**Postnatal MRI**	17 / 52 (33%)	9 / 29 (31%)	8 / 23 (35%)

[1] Frequency n/(N row) (%)

Table S5a. Type of maternal ZIKV assessment among ZIKV-IPD-MA study participants of the metadata survey.

Characteristic	N = 51[1]	Characteristic	N = 51[1]
**Type of Maternal ZIKV Assessment**		**Performed PRNT**	12 / 51 (24%)
Clinical and Laboratory Criteria	13 / 51 (25%)	**PRNT %**	
Laboratory Criteria	38 / 51 (75%)	PRNT50	2 / 12 (17%)
**Performed PCR**	48 / 51 (94%)	PRNT90	6 / 12 (50%)
**PCR Type**		Unknown	4 / 12 (33%)
Conventional PCR	5 / 48 (10%)	**PRNT Cut-off Titers**	
qRT-PCR	15 / 48 (31%)	PRNT>20	5 / 12 (42%)
RT-PCR	27 / 48 (56%)	PRNT≥10	5 / 12 (42%)
Unknown	1 / 48 (2.1%)	Unknown	2 / 12 (17%)
**Performed QC for PCR**		**Performed DENV/ZIKV PRNT**	
No	5 / 48 (10%)	No	8 / 13 (62%)
Unknown	8 / 48 (17%)	Unknown	3 / 13 (23%)
Yes	35 / 48 (73%)	Yes	2 / 13 (15%)
**Performed Immunoassays**	35 / 51 (69%)	**Performed QC for PRNT**	
**Performed ELISA IgM**	34 / 51 (67%)	No	10 / 12 (83%)
**Performed ELISA IgG**	18 / 51 (35%)	Unknown	1 / 12 (8.3%)
**Performed QC for ELISAs**		Yes	1 / 12 (8.3%)
No	1 / 35 (2.9%)		
Unknown	8 / 35 (23%)		
Yes	26 / 35 (74%)		

[1] Frequency n/(N row) (%)

Table S5b. Type of fetal ZIKV assessment among ZIKV-IPD-MA study participants of the metadata survey.

**Characteristic**	**N = 27**[1]
**Type of Fetal ZIKV Assessment**	
Fetal Imaging Criteria	1 / 27 (3.7%)
Fetal Laboratory Criteria	2 / 27 (7.4%)
Maternal & Fetal Laboratory Criteria	7 / 27 (26%)
Maternal & Fetal Laboratory Criteria, including Imaging	11 / 27 (41%)
Maternal Laboratory Criteria	6 / 27 (22%)
**Performed PCR**	18 / 27 (67%)
**PCR Type**	
Conventional PCR	1 / 18 (5.6%)
qRT-PCR	8 / 18 (44%)
RT-PCR	9 / 18 (50%)
**Performed QC for PCR**	
No	3 / 18 (17%)
Unknown	2 / 18 (11%)
Yes	13 / 18 (72%)
**Performed ELISA IgM**	8 / 27 (30%)
**Performed ELISA IgG**	4 / 27 (15%)
**Performed QC for ELISAs**	
No	1 / 9 (11%)
Unknown	1 / 9 (11%)
Yes	7 / 9 (78%)
**Performed PRNT**	1 / 27 (3.7%)

[1] Frequency n/(N row) (%)

Table S5c. Type of infant ZIKV assessment among ZIKV-IPD-MA study participants of the metadata survey.

**Characteristic**	**N = 41**[1]
**Type of Infant ZIKV Assessment**	
Infant Laboratory & Imaging Criteria	7 / 41(17%)
Infant Laboratory Criteria	14 / 41 (34%)
Maternal & Infant Laboratory Criteria	3 / 41 (7%)
Maternal & Infant Laboratory Criteria, including Imaging	15 / 41 (37%)
Maternal Clinical/Laboratory Criteria	2 / 41 (5%)
**Performed PCR**	33 / 41 (80%)
**PCR Type**	
Conventional PCR	5 / 33 (15%)
qRT-PCR	13 / 33 (39%)
RT-PCR	13 / 33 (39%)
Unknown	2 / 33 (6.1%)
**Performed QC for PCR**	
No	5 / 33 (15%)
Unknown	7 / 33 (21%)
Yes	21 / 33 (64%)
**Performed ELISA IgM**	24 / 41 (59%)
**Performed ELISA IgG**	14 / 41 (34%)
**Performed QC for ELISAs**	
No	2 / 25 (8.0%)
Unknown	5 / 25 (20%)
Yes	18 / 25 (72%)
**Performed PRNT**	5 / 41 (12%)

[1] Frequency n/(N row) (%)

Carabali M, *et al. BMJ Open* 2022; 12:e064362. doi: 10.1136/bmjopen-2022-064362
