# Supplementary material for: Heterogeneity of Zika virus exposure and outcome ascertainment across cohorts of pregnant women, their infants and their children: a metadata survey
Source: BMJ Open. 2022 Nov 22;12(11):e064362. doi: 10.1136/bmjopen-2022-064362 (PMC9685007; doi:10.1136/bmjopen-2022-064362)
Supplement: Supplementary data [file bmjopen-2022-064362supp002.pdf]

Confidential

Appendix 2

ZIKV IPD-MA Metadata survey

The metadata survey collects information from investigators about how their study or site measured ZIKV and DENV infection, how positive, negative, and indeterminate ZIKV tests were classified, and information on the measurement of confounders and outcomes of interest. These data will be used to appropriately account for study-level heterogeneity and measurement error in the statistical models developed for ZIKV IPD-MA Objectives 1-4.

The metadata survey may take up to one hour to complete and may require input from different members of your study team. Please contact Lauren Maxwell at maxwelll@who.int if you have questions while completing the metadata survey.

El mismo mensaje en español

La encuesta de metadatos recopila información de los investigadores sobre cómo su estudio o sitio midió la infección por ZIKV y DENV, cómo se clasificaron las pruebas de ZIKV positivas, negativas e indeterminadas, e información sobre la medición de factores de confusión y resultados de interés. Estos datos se utilizarán para explicar adecuadamente la heterogeneidad a nivel de estudio y el margin de error en los modelos estadísticos desarrollados para ZIKV IPD-MA Objetivos 1-4.

La encuesta de metadatos puede tardar hasta una hora en completarse y requerirá aportes de diferentes miembros de su equipo de investigacion. Por favor, póngase en contacto con Lauren Maxwell at maxwelll@who.int si tiene preguntas mientras completa la encuesta de metadatos.

A mesma mensagem em português

A pesquisa de metadados coleta informações de pesquisadores sobre como o estudo ou local mediu a infecção por ZIKV e DENV, como foram classificados os testes positivos, negativos e indeterminados de ZIKV e informações sobre como medir fatores de confusão e resultados de interesse. Esses dados serão utilizados para explicar adequadamente a heterogeneidade no nível do estudo e a margem de erro nos modelos estatísticos desenvolvidos para os objetivos 1-4 do ZIKV IPD-MA.

A pesquisa de metadados pode levar até uma hora para ser concluída e exigirá informações de diferentes membros da sua equipe de pesquisa. Entre em contato com Lauren Maxwell em maxwelll@who.int, se tiver dúvidas ao concluir a pesquisa de metadados.

Basic study information

Your first name ______________________

Your last name ______________________

Study name ____________________________

Carabali M, *et al. BMJ Open* 2022; 12:e064362. doi: 10.1136/bmjopen-2022-064362

Confidential

Study country	◯ Brazil<br>◯ Colombia<br>◯ Non-continental US<br>◯ Continental US<br>◯ Mexico<br>◯ Honduras<br>◯ Spain<br>◯ Thailand<br>◯ Nicaragua<br>◯ Philippines<br>◯ France<br>◯ Guyana<br>◯ Suriname<br>◯ Greece<br>◯ Grenada<br>◯ Italy<br>◯ UK<br>◯ Trinidad and Tobago<br>◯ Jamaica<br>◯ Singapore<br>◯ Kenya<br>◯ Other<br>◯ Multi-country study
For multi-country studies, specify the included countries	__________________________________
Specify other country	____________________________
Study state	____________________________<br>(Write N/A if you have a country-level study)
Study city	____________________________<br>(Write N/A if you have a state- or country-level study)
Number of study sites	____________________________
Provide city and state for each site, if relevant<br>Use a semi-colon to separate sites and a comma to separate the city and state<br><br>e.g. New York City, New York; San Francisco, California	__________________________________
Does your study agree, in principle, to share de-identified subject-level data for the ZIKV IPD Consortium IPD-MA?	◯ Yes<br>◯ No<br>◯ Considering
Study Type	◯ Cohort<br>◯ Active surveillance system<br>◯ Case cohort<br>◯ Other

Specify other study type	__________________________________
What is the source population for your study?<br><br>Select all that apply	☐ Hospital or health care center<br>☐ Community<br>☐ Travelers<br>☐ Other
What are the other source population(s) for your study?	__________________________________
Was your study funded by any of the following groups?<br><br>Select all that apply	☐ European Union<br>☐ GlOPID-R<br>☐ WHO<br>☐ PAHO<br>☐ Africa CDC<br>☐ E-CDC<br>☐ US-CDC<br>☐ Bill and Melinda Gates Foundation<br>☐ NIH/NIAID<br>☐ Wellcome Trust<br>☐ DfID<br>☐ USAID<br>☐ DFG<br>☐ Other<br>☐ Study did not receive funding from any of these groups
Which other group funded your study?	____
When did your study begin enrolling women?	__________________________________
When did your study finish enrolling women?	__________________________________<br>(Specify the date you estimate you will stop enrolling women if enrollment is ongoing)
How many women did your study PLAN to enroll?	__________________________________
How many women has your study ACTUALLY enrolled to date?	__________________________________
How many ZIKV+ pregnant women has your study enrolled to date?	__________________________________
How many infants or children did you study include?	__________________________________
For multi-site studies, have all sites applied the same laboratory and clinical protocols?	◯ Same laboratory protocol<br>◯ Same clinical protocol<br>◯ Same laboratory & clinical protocols<br>◯ Sites apply different laboratory and clinical protocols

Confidential

Describe cross-site differences in laboratory protocols	__________________
Describe cross-site differences in clinical protocols	__________________

Carabali M, *et al. BMJ Open* 2022; 12:e064362. doi: 10.1136/bmjopen-2022-064362

Confidential

Enrollment criteria & recruitment methods

Describe the inclusion criteria for the cohort

____

Describe the exclusion criteria for the cohort

____

Describe the inclusion criteria for the cohort from which the case-cohort was sampled

____

Describe the exclusion criteria for the cohort from which the case-cohort was sampled

____

What was the case definition used to identify cases for the case-cohort study?

____

How were women sampled from the cohort to participate in the case-cohort study?

____

Which groups were recruited to the study?

- ◯ pregnant women
- ◯ recently delivered women with infants with evidence of ZIKV infection
- ◯ recently delivered women, regardless of their infants' ZIKV status
- ◯ women of reproductive age, regardless of their pregnancy status
- ◯ other

Which other groups were recruited to the study?

____

How was the pregnancy identified?

Select all that apply

- ☐ Self-reported
- ☐ 1st obstetric visit (blood or urine test)
- ☐ Urine test (self-administered at home)
- ☐ Urine test (at health care facility)
- ☐ Blood test
- ☐ Ultrasound
- ☐ Varies at the participant level

How was gestational age determined?

Select all that apply

- ☐ LMP (last menstrual period)
- ☐ US (ultrasound)

Was ZIKV the original disease of interest for your cohort?

- ◯ Yes
- ◯ No, was originally a DENV study
- ◯ No, was originally an influenza study
- ◯ No, was formed for another purpose

What was the initial purpose of your cohort?

____

Confidential

If your study wasn't originally designed to measure ZIKV, which symptoms were used to decide whether to test women for ZIKV?<br><br>Select all that apply	☐ Fever<br>☐ Rash<br>☐ Joint pain<br>☐ Conjunctivitis<br>☐ Rash OR joint pain OR conjunctivitis OR fever<br>☐ Other<br>☐ Symptoms not required for testing
What other symptoms were used to decide whether to test women for ZIKV?	__________________________________
How was enrollment of women or infants into the study related to their ZIKV status?<br><br>Select all that apply	☐ women's ZIKV assay<br>☐ women's ZIKV symptoms<br>☐ women's ZIKV assay OR ZIKV symptoms<br>☐ infant's ZIKV assay<br>☐ infant's ZIKV symptoms<br>☐ infant's ZIKV assay OR ZIKV symptoms<br>☐ women's presumed exposure (travel or sexual)<br>☐ other<br>☐ enrollment not related to ZIKV status of women or infants
In what other way was enrollment of women or infants into the study related to their ZIKV status?	__________________________________
How was study enrollment of women related to their ZIKV assay result or their infant's ZIKV assay result?	◯ Study only enrolled ZIKV PCR pos (+) pregnant women<br>◯ Study only enrolled ZIKV PCR pos (+) or probable pregnant women<br>◯ Study only enrolled ZIKV PCR neg (-) pregnant women<br>◯ Study only enrolled women with ZIKV PCR pos (+) infants, irregardless of women's ZIKV infection status<br>◯ Study only enrolled women with ZIKV PCR pos (+) or ZIKV probable infants, irregardless of women's ZIKV infection status<br>◯ Study only enrolled recently delivered ZIKV PCR pos (+) women with ZIKV PCR pos (+) or ZIKV probable infants<br>◯ Other
Describe other ZIKV assay result-related inclusion criteria	__________________________________
What ZIKV-related symptoms did your study consider when enrolling women?	◯ Fever only<br>◯ Rash only<br>◯ Fever + rash<br>◯ Fever or rash<br>◯ Fever + rash + joint pain<br>◯ Fever + rash + joint pain + conjunctivitis<br>◯ Other<br>◯ Fever + rash + other
Describe other ZIKV symptom-related inclusion criteria	__________________________________

Confidential

What ZIKV-related symptoms in infants did your study consider when enrolling infants and/or their mothers?	◯ Fever only<br>◯ Rash only<br>◯ Fever + rash<br>◯ Fever or rash<br>◯ Fever + rash + joint pain<br>◯ Fever + rash + joint pain + conjunctivitis<br>◯ Other<br>◯ Fever + rash + other
What other ZIKV-related symptoms in infants did your study consider when enrolling participants?	__________________________________
Describe any additional ZIKV-related inclusion criteria	__________________________________
Describe any additional non-ZIKV related inclusion criteria (e.g. HIV co-infected, residence in a certain neighborhood, etc).	__________________________________
List any study exclusion criteria	__________________________________
What was your mechanism for identifying potential participants for your study?<br><br>Select all that apply	☐ Referrals from active surveillance system<br>☐ Referrals from hospital or clinic<br>☐ Preexisting cohort<br>☐ Other
What was your mechanism for identifying potential participants for your study?	___________________________
Were potential participants referred to your study using a surveillance definition?	◯ Yes<br>◯ No
What was the surveillance definition used to refer participants to the study or surveillance system?	__________________________________
Where did recruitment take place?<br><br>Select all that apply	☐ Primary healthcare facility<br>☐ Tertiary healthcare facility<br>☐ Any healthcare facility (not specified by the study)<br>☐ Community<br>☐ Other
Where else did recruitment take place?	__________________________________
When during pregnancy were women enrolled?	◯ Prior to pregnancy<br>◯ During 1st trimester only<br>◯ During 1st or 2nd trimester only<br>◯ Anytime during pregnancy<br>◯ First obstetric visit<br>◯ Other

Carabali M, *et al. BMJ Open* 2022; 12:e064362. doi: 10.1136/bmjopen-2022-064362

At what other time were women enrolled?	__________________________________
Who recruited potential participants?	◯ Health care facility staff<br>◯ Project interviewer (not health care facility staff member)<br>◯ Community health worker<br>◯ Other
Which other group enrolled research participants?	__________________________________

Confidential

Follow up schedules for pregnant women and infants

Was the number and timing of visits during pregnancy the same for ZIKV+ and ZIKV- pregnant women?	◯ Yes<br>◯ No<br>◯ Study only followed ZIKV + pregnant women<br>◯ Study did not follow any pregnant women
How many visits did the study plan to have with pregnant women?	____________________<br>(If there is a range for the number of visits, please indicate the upper limit of the range)
At what time during pregnancy was the visit/were the visits planned to occur?	____________________<br>(e.g. every trimester, every month, months 1, 6, 9, etc.)
How many visits did the study plan to have with ZIKV+ pregnant women?	____________________<br>(If there is a range for the number of visits, please indicate the upper limit of the range)
At what time during pregnancy was the visit/were the visits scheduled to occur with ZIKV+ pregnant women?	____________________<br>(e.g. every trimester, every month, months 1, 6, 9, etc.)
How many visits did the study plan to have with ZIKV- pregnant women?	____________________<br>(If there is a range for the number of visits, please indicate the upper limit of the range)
At what time during pregnancy was the visit/were the visits scheduled to occur with ZIKV- pregnant women?	____________________<br>(e.g. every trimester, every month, months 1, 6, 9, etc.)
Other than timing and duration of follow-up, describe any differences in the types of assessments conducted with ZIKV+ and ZIKV- pregnant women:	____________________
Did the study follow infants after birth?	◯ Yes<br>◯ No
Was the number and timing of follow-up visits the same for the infants/children of ZIKV+ and ZIKV- pregnant women?	◯ Yes<br>◯ No<br>◯ Study did not follow infants/children of ZIKV- pregnant women
How many visits did the study plan to have with infants/children?	____________________<br>(If there is a range for the number of visits, please indicate the upper limit of the range)
Up until what age (in months) did your study intend to follow infants/children?	____________________

At what ages was the visit/were the visits with infants/children scheduled to occur?	__________________ (Specify whether ages listed in months or years)
How many visits did the study plan to have with infants/children from ZIKV+ mothers?	__________________ (If there is a range for the number of visits, please indicate the upper limit of the range)
Up until what age (in months) did your study plan to follow infants/children of ZIKV+ mothers?	__________________
At what ages was the visit/were the visits with infants/children of ZIKV+ mothers scheduled to occur?	__________________ (Specify whether ages listed in months or years)
How many visits did the study plan to have with infants/children of ZIKV- mothers?	__________________ (If there is a range for the number of visits, please indicate the upper limit of the range)
Up until what age (in months) did your study plan to follow infants/children of ZIKV- mothers?	__________________
At what ages was the visit/were the visits with infants/children of ZIKV- mothers scheduled to occur?	__________________ (Specify whether ages listed in months or years)
Other than timing and duration of follow-up, describe any differences in the types of assessments conducted with infants/children of ZIKV+ and ZIKV- pregnant women:	__________________

ZIKV symptoms in pregnant women and infants

Which of the following were measured to determine symptomatic ZIKV infection in women?<br><br>Select all that apply	☐ Fever<br>☐ Rash<br>☐ Joint pain<br>☐ Conjunctivitis<br>☐ Rash OR joint pain OR conjunctivitis OR fever<br>☐ Other
What else was measured to determine symptomatic ZIKV infection in women?	______________________
How was fever measured in women?<br><br>Select all that apply	☐ Underarm<br>☐ Sublingual<br>☐ Otic<br>☐ Rectal<br>☐ Self reported or subjective (any site)
How did your study define fever in women? (degrees Celsius)	______________________<br>(Degrees Celsius)
How was fever measured in infants?<br><br>Select all that apply	☐ Underarm<br>☐ Sublingual<br>☐ Otic<br>☐ Rectal<br>☐ Self reported or subjective (any site)
How did your study define fever in infants? (degrees Celsius)	______________________<br>(Degrees Celsius)

Confidential

Ascertainment of maternal ZIKV infection

What methods were used to assess maternal ZIKV status?<br><br>Select all that apply	☐ Clinical case definition<br>☐ Molecular diagnosis (e.g. RT-PCR, other NAAT)<br>☐ Immunoassay (e.g. ELISA, IFA, PRNT, microneutralization)<br>☐ Rapid diagnostic test (RDT)<br>☐ Other
What other method was used to assess maternal ZIKV status?	__________________________
Did you use different assays, different brands of the same assay, or different versions of the same brand of assay at different study sites to ascertain maternal ZIKV status or over time within the study?<br><br>Select all that apply	☐ Yes, different versions of the same brand of assay over time<br>☐ Yes, different brands of the same type of assay over time<br>☐ Yes, different types of assays over time<br>☐ Yes, different versions of the same brand of assay at different sites<br>☐ Yes, different brands of the same type of assay at different sites<br>☐ Yes, different types of assays at different sites<br>☐ No, same tests applied throughout the project
Which different assay(s) did you use over time or across sites?	__________________________
Which different brands of the assay did you use over time or across sites?	__________________________
Which different versions of the brand of assay did you use over time or across sites?	__________________________

Confidential

Ascertainment of maternal ZIKV infection - Immunoassay

ZIKV immunoassay analyte<br><br>Select all that apply	☐ IgM<br>☐ IgG<br>☐ IgA<br>☐ neutralizing antibodies<br>☐ Other
Other ZIKV immunoassay analyte	__________________________________
ZIKV immunoassay type<br><br>Select all that apply	☐ ELISA<br>☐ IFA<br>☐ MIA<br>☐ PRNT<br>☐ Other
Specify other type(s) or ZIKV immunoassay(s) performed	__________________________________

Carabali M, *et al. BMJ Open* 2022; 12:e064362. doi: 10.1136/bmjopen-2022-064362

Confidential

ZIKV immunoassay brand name<br><br>Select all that apply	☐ In house-developed assay<br>☐ Zika MAC-ELISA (CDC)<br>☐ ZIKV Detect 2.0 IgM Capture ELISA Kit (InBios International Inc)<br>☐ Zika IgM ELISA (EUROIMMUN)<br>☐ Zika IgM + IgA ELISA (EUROIMMUN)<br>☐ Zika IgG ELISA (EUROIMMUN)<br>☐ ZIKV IgM IFA (EUROIMMUN)<br>☐ ZIKV IgG IFA (EUROIMMUN)<br>☐ Arbovirus Fever Mosaic 2 IgG + IgM IFA ZIKV-DENV-CHIKV (EUROIMMUN)<br>☐ ADVIA Centaur Zika Test (Siemens Healthcare Diagnostics)<br>☐ DPP Zika IgM Assay System (Chembio Diagnostic Systems Inc)<br>☐ DPP Zika IgM/IgG System (Chembio Diagnostic Systems Inc)<br>☐ DPP Zika/Chikungunya/Dengue IgM/IgG Combination Assay (Chembio Diagnostic Systems Inc)<br>☐ LIAISON XL Zika Capture IgM II assay (DiaSorin S.p.A.)<br>☐ STANDARD Q Zika IgM/IgG test (SD Biosensor)<br>☐ STANDARD E Zika IgM ELISA (SD Biosensor)<br>☐ STANDARD Q ZIKV/DENV/CHIKV Fast Quad (SD Biosensor)<br>☐ STANDARD Q ZIKV/DENV/CHIKV/YFV IgM Quad (SD Biosensor)<br>☐ Tell Me Fast Zika IgG/IgM Antibody Rapid Test (Biocan Diagnostics Inc)<br>☐ Tell Me Fast zika/dengue/chikungunya combination rapid tests (Biocan Diagnostics Inc)<br>☐ ZIKV-DENV-CHIKV IFA IgG (Vircell)<br>☐ ZIKV-DENV-CHIKV IFA IgM (Vircell)<br>☐ Human Anti-Zika ELISA Kits (Alpha Diagnostic International)<br>☐ anti-Zika virus IgM ELISA kit (µ-capture) (AbCam)<br>☐ anti-Zika virus IgG ELISA kit (AbCam)<br>☐ Zika Virus IgM ELISA Assay Kit (Eagle Biosciences)<br>☐ Zika Virus IgG ELISA Assay Kit (Eagle Biosciences)<br>☐ Human Anti-Zika Virus IgG ELISA Kit (R&D Systems)<br>☐ ZIKV IgG ELISA kit (DIA.PRO Diagnostic Bioprobes Srl)<br>☐ ZIKV IgM ELISA kit (DIA.PRO Diagnostic Bioprobes Srl)<br>☐ NovaLisa Zika Virus IgM µ-capture ELISA (NovaTec Immunodiagnostica GmbH)<br>☐ recomLine Tropical Fever IgG (MIKROGEN Diagnostik)<br>☐ recomLine Tropical Fever IgM (MIKROGEN Diagnostik)<br>☐ Other
Which other immunoassays were used to diagnose maternal ZIKV infection?	__________________________________
What type of assay is the house assay?<br><br>Select all that apply	☐ ELISA<br>☐ IFA<br>☐ MIA<br>☐ PRNT<br>☐ Other
What other type of assay is the in house assay?	__________________________________

What is the analyte measured by the in house assay?<br><br>Select all that apply	☐ IgM<br>☐ IgG<br>☐ IgA
What is the antigenic target for the in house assay?<br><br>Select all that apply	☐ NS1<br>☐ envelope protein<br>☐ NS5<br>☐ Other
What is the antigenic target for the in house assay?	______________________
For the in house ZIKV immunoassay, describe the testing procedure, including: incubation period, timing, type of cells, buffers, method to detect binding antibodies (e.g., optical density or secondary antibody detection), and methods to stop the enzymatic reaction	______________________
Sample used for ZIKV immunoassay<br><br>Select all that apply	☐ Serum/plasma<br>☐ Whole blood<br>☐ Other
Specify other sample used for ZIKV immunoassay	______________________
ZIKV ELISA tested in paired/subsequent samples	○ Yes<br>○ No
At what temperature was the ZIKV ELISA test stored? (degrees Celsius)	______________________
At what temperature(s) was/were the ZIKV ELISA sample(s) stored? (degrees Celsius)	______________________<br>(Include sample type for each temperature)
In what volume(s) was/were the ZIKV ELISA sample aliquot(s) stored? (mL)	______________________<br>(Include sample type with each volume)
Did your study deviate from the directions provided in the ZIKV ELISA commercial kit?	○ Yes<br>○ No<br>○ Study did not use a commercial ZIKV ELISA kit
Describe any deviations from the procedure recommended by the commercial ELISA kit, including: incubation period, timing, type of cells, buffers, method to detect bounding antibodies, and methods to stop the enzymatic reaction	______________________
Describe any additional conditions for ZIKV ELISA test administration (e.g. paired samples within 21 days of fever onset; count with more than 2ul of serum from the febrile phase, etc.)	______________________

Where was the ZIKV ELISA test performed?<br><br>Select all that apply	☐ Lab at the same health care facility where study is conducted<br>☐ Study-specific lab<br>☐ Ministry of Health reference lab<br>☐ Private external lab<br>☐ Other
Specify other lab where ZIKV ELISA was performed	__________________________________

Ascertainment of maternal ZIKV infection - ELISA

ZIKV ELISA cut-off point	__________________________________
Was a positive control from the outbreak included for the ZIKV ELISAs?	○ Yes<br>○ No
Was a negative control included with every run of ZIKV ELISAs?	○ Yes<br>○ No
Definition of POSITIVE ZIKV ELISA	____
Definition of NEGATIVE ZIKV ELISA	____
Definition of INCONCLUSIVE ZIKV ELISA	____
Source of definition used to interpret ZIKV ELISA test results<br><br>Select all that apply	☐ Manufacturer<br>☐ Ministry of Health reference lab<br>☐ WHO (global or local office e.g: PAHO)<br>☐ US-CDC<br>☐ Consensus/study researchers<br>☐ Other
Specify other source of definition used to interpret ZIKV ELISA test results	__________________________________
How many ZIKV ELISAs did the study plan to conduct in each pregnant woman?	__________________________________
Would your study be able to provide the P/N ratio, titer, or other quantitative readout for ZIKV ELISAs to inform the measurement error models?	○ Yes<br>○ No

Confidential

Ascertainment of maternal ZIKV infection - IFA

ZIKV IFA cut-off value	__________________________________
Was a positive control from the outbreak included for the ZIKV IFA?	◯ Yes<br>◯ No
Was a negative control included with every run of the ZIKV IFA?	◯ Yes<br>◯ No
Definition of POSITIVE ZIKV IFA	____
Definition of NEGATIVE ZIKV IFA	____
Definition of INCONCLUSIVE ZIKV IFA	____
Source of definition used to interpret ZIKV IFA test results<br><br>Select all that apply	☐ Manufacturer<br>☐ Ministry of Health reference lab<br>☐ WHO (global or local office e.g: PAHO)<br>☐ Consensus/study researchers<br>☐ Other
Specify other source of definition for ZIKV IFA test results	__________________________________
Specify any additional inclusion or exclusion criteria for determining which samples to test for ZIKV using IFA	____
Will your study be able to provide the titer or optical density for ZIKV IFAs to inform the measurement error model?	◯ Yes<br>◯ No

Ascertainment of maternal ZIKV infection - MIA

ZIKV MIA cut-off value	__________________
Was a positive control from the outbreak included for the ZIKV MIAs?	◯ Yes<br>◯ No
Was a negative control included with every run of ZIKV MIAs?	◯ Yes<br>◯ No
Definition of POSITIVE ZIKV MIA	__________________
Definition of NEGATIVE ZIKV MIA	__________________
Definition of INCONCLUSIVE ZIKV MIA	__________________
Source of definition used to interpret ZIKV MIA test results<br><br>Select all that apply	☐ Manufacturer<br>☐ Ministry of Health reference lab<br>☐ WHO (global or local office e.g: PAHO)<br>☐ Consensus/study researchers<br>☐ Other
Specify other source of definition for ZIKV MIA test results	__________________
Specify any additional inclusion or exclusion criteria for determining which samples to test for ZIKV using MIA	__________________
Will your study be able to provide the titer for ZIKV MIAs to inform the measurement error model?	◯ Yes<br>◯ No

Ascertainment of maternal ZIKV infection - PRNT

Question	Response
Did you use the CDC protocol for the PRNT?	◯ Yes<br>◯ No
Describe the other protocol used for the PRNT	__________________________
End-point PRNT titer	◯ PRNT50<br>◯ PRNT90<br>◯ Other
Which other end-point did you use for the PRNT titer?	____________________
Definition of a positive PRNT titer	◯ PRNT>10<br>◯ Other
Specify other definition of a positive PRNT titer	____________________
Source of definition used to interpret ZIKV PRNT test results<br><br>Select all that apply	☐ Manufacturer<br>☐ Ministry of Health reference lab<br>☐ WHO (global or local office e.g: PAHO)<br>☐ US-CDC<br>☐ Consensus/study researchers<br>☐ Other
Specify other source of definition used to interpret ZIKV PRNT test results	____________________
Definition of POSITIVE ZIKV PRNT	__________________________
Definition of NEGATIVE ZIKV PRNT	__________________________
Definition of INCONCLUSIVE PRNT (i.e. specific flavivirus could not be determined)	__________________________
Specify any additional inclusion or exclusion criteria for determining which samples to test for ZIKV using PRNT	__________________________
Where was the ZIKV PRNT test performed?<br><br>Select all that apply	☐ Lab at the same health care facility where study is conducted<br>☐ Study-specific lab<br>☐ Ministry of Health reference lab<br>☐ Private external lab<br>☐ Other
Specify other lab where ZIKV PRNT test was performed	____________________

BMJ Publishing Group Limited (BMJ) disclaims all liability and responsibility arising from any reliance placed on this supplemental material which has been supplied by the author(s)

How many ZIKV PRNTs were conducted with pregnant women?	__________________
Will your study be able to provide the titer for ZIKV PRNTs to inform the measurement error model?	◯ Yes<br>◯ No

Carabali M, *et al. BMJ Open* 2022; 12:e064362. doi: 10.1136/bmjopen-2022-064362

Ascertainment of maternal ZIKV infection - Molecular assay

Type of ZIKV molecular tests performed<br><br>Select all that apply	☐ conventional PCR<br>☐ rRT-PCR<br>☐ qRT-PCR<br>☐ Other NAAT
According to the study protocol, when were the ZIKV samples to be collected for molecular testing?<br><br>Select all that apply	☐ 3 days after fever onset<br>☐ 5 days after fever onset<br>☐ 7 days after fever onset<br>☐ 10 days after fever onset<br>☐ 14 days after fever onset<br>☐ Other
When else were the ZIKV samples to be collected for molecular testing?	________________________________
Sample used for ZIKV RT-PCR/PCR<br><br>Select all that apply	☐ Serum/plasma<br>☐ Whole blood<br>☐ Urine<br>☐ Saliva<br>☐ Breast milk<br>☐ Umbilical cord tissue<br>☐ Placental tissue<br>☐ Amniotic fluid<br>☐ Other
Specify other type of sample used for ZIKV RT-PCR/PCR	__________________________

Carabali M, *et al. BMJ Open* 2022; 12:e064362. doi: 10.1136/bmjopen-2022-064362

ZIKV RT-PCR/PCR brand name<br><br>Select all that apply	☐ In house technique<br>☐ Zika Virus RNA Qualitative Real-Time RT-PCR (Quest Diagnostics)<br>☐ RealStar Zika Virus RT-PCR Kit U.S. (altona Diagnostics GmbH)<br>☐ RealStar Zika Virus RT-PCR Kit 1.0 (altona Diagnostics GmbH)<br>☐ Aptima Zika Virus assay (Hologic Inc)<br>☐ Zika Virus Real-time RT-PCR (Viracor Eurofins)<br>☐ VERSANT Zika RNA 1.0 Assay (kPCR) (Siemens Healthcare Diagnostics Inc)<br>☐ xMAP MultiFLEX Zika RNA Assay (Luminex Corporation)<br>☐ Sentosa SA ZIKV RT-PCR Test (Vela Diagnostics Inc)<br>☐ Zika Virus Detection by RT-PCR (ARUP Laboratories)<br>☐ Abbott RealTime Zika (Abbott Molecular Inc)<br>☐ Zika ELITe MGB Kit U.S. (ELITechGroup Molecular Diagnostics)<br>☐ Gene-RADAR Zika Virus Test (Nanobiosym Diagnostics Inc)<br>☐ TaqPath Zika Virus Kit (Thermo Fisher Scientific)<br>☐ CII-ArboViroPlex rRT-PCR assay (Columbia University)<br>☐ Trioplex Real-Time RT-PCR Assay (CDC)<br>☐ Liferiver Zika Virus (ZIKV) Real Time RT-PCR Kit (Shanghai ZJ Bio-Tech Co Ltd)<br>☐ careGENE Zika Virus RT-PCR Kit (WELLS BIO Inc)<br>☐ Zika Virus - Single Check (Genekam Biotechnology AG)<br>☐ FTD Zika virus (Fast Track Diagnostics)<br>☐ VIASURE Zika Virus Real Time PCR Detection Kit (Certest Biotec)<br>☐ Zika Virus Real-TM (Sacace Biotechnologies)<br>☐ Logix Smart Zika Test Kit (Co-Diagnostics Inc)<br>☐ Genesig Kits for ZIKV (Primerdesign Ltd)<br>☐ Genesig Multiplex Kit for Dengue, Chikungunya and Zika Virus (Primerdesign Ltd)<br>☐ AccuPower ZIKV (DENV, CHIKV) Multiplex Real-Time RT-PCR Kit (Bioneer)<br>☐ VIASURE Zika, Dengue & Chikungunya Real Time PCR Detection Kit (Certest Biotec)<br>☐ FTD Zika/Dengue/Chik (Fast-Track Diagnostics)<br>☐ DiaPlexQ ZCD (ZIKV, CHIKV, DENV) Detection Kit (SolGent Co Ltd)<br>☐ TaqMan Zika Virus Triplex Kit (Thermo Fisher Scientific)<br>☐ GenoAmp Trioplex Real-Time RT-PCR Zika/Den/Chiku (Medical Innovation Ventures)<br>☐ Other
Describe procedure for in house ZIKV RT-PCR/PCR	______________________________
Specify other ZIKV RT-PCR/PCR brand name	______________________________
Did the study obtain a measure of ZIKV viral load?	◯ Yes<br>◯ No
ZIKV PCR/RT-PCR cut-off point	_________________________

Confidential

Was a negative control included with every run of ZIKV RT-PCR/PCRs?	◯ Yes<br>◯ No
Definition of POSITIVE ZIKV RT-PCR/PCR	__________________
Definition of NEGATIVE ZIKV RT-PCR/PCR	__________________
Definition of INCONCLUSIVE ZIKV RT-PCR/PCR	__________________
Source of definition used to interpret ZIKV RT-PCR/PCR test results<br><br>Select all that apply	☐ Manufacturer<br>☐ Ministry of Health reference lab<br>☐ WHO (global or local office e.g: PAHO)<br>☐ US-CDC<br>☐ Consensus/study researchers<br>☐ Other
Specify other source of definition used to interpret ZIKV RT-PCR/PCR test results	__________________
Where was the ZIKV RT-PCR/PCR test performed?<br><br>Select all that apply	☐ Lab at the same health care facility where study is conducted<br>☐ Study-specific lab<br>☐ Ministry of Health reference lab<br>☐ Private external lab<br>☐ Other
Specify other lab where ZIKV RT-PCR/PCR test was performed	__________________
Specify any additional inclusion or exclusion criteria for determining which samples to test for ZIKV using RT-PCR/PCR	__________________
How many ZIKV PCRs were conducted with pregnant women?	__________________
Would your study be able to provide a Ct value for ZIKV PCRs to inform the measurement error models?	◯ Yes<br>◯ No

Carabali M, *et al. BMJ Open* 2022; 12:e064362. doi: 10.1136/bmjopen-2022-064362

Confidential

ZIKV testing algorithm & test interpretation - Maternal ZIKV infection

Describe the testing algorithm for defining a confirmed ZIKV+ test in pregnant women
____

Describe the testing algorithm for defining a confirmed ZIKV- test in pregnant women
____

What tests were used to confirm ZIKV infection in pregnant women?

Select all that apply

- ☐ ZIKV IgM - 1 sample
- ☐ ZIKV IgM - paired samples (seroconversion or increased titers)
- ☐ ZIKV IgM (1 sample) + IgG
- ☐ ZIKV IgM (paired samples) + IgG
- ☐ RT-PCR or other NAAT
- ☐ PRNT > 10
- ☐ Other

Specify other test or combination of tests used to confirm ZIKV infection in pregnant women
____

Considering any relevant clinical and laboratory criteria, what was the study's definition of a CONFIRMED maternal ZIKV Infection
____

Considering any relevant clinical and laboratory criteria, what was the study's definition of a PROBABLE maternal ZIKV Infection
____

Considering any relevant clinical and laboratory criteria, what was the study's definition of a NEGATIVE/UNLIKELY maternal ZIKV Infection
____

Source of ZIKV test result categorization

Select all that apply

- ☐ Manufacturer
- ☐ Ministry of Health reference lab
- ☐ WHO (global or local office e.g: PAHO)
- ☐ Consensus/study researchers
- ☐ Other

Specify other source of ZIKV test result categorization
____

Changes in maternal ZIKV assessment over time

Which criteria did you use to infer maternal ZIKV status or fetal exposure to ZIKV?<br><br>Select all that apply	☐ Maternal clinical criteria<br>☐ Maternal laboratory criteria<br>☐ Infant clinical criteria<br>☐ Infant laboratory criteria
Which clinical criteria did you apply initially to diagnose maternal infection? (Clinical Definition 1)	________________________________
Did your clinical criteria for diagnosing maternal infection change over time?	◯ No<br>◯ Yes - changed 1x<br>◯ Yes - changed more than 1x
If your clinical criteria changed over time, which criteria did you apply subsequently? (Clinical Definition 2)	________________________________
When did you begin using the revised criteria? (Clinical Definition 2)	__________________________
What was the third set of criteria you applied? (Clinical Definition 3)	________________________________
When did you begin using the third set of clinical criteria? (Clinical Definition 3)	__________________________
What laboratory criteria was used initially to diagnose maternal ZIKV infection? (Laboratory Definition 1)	________________________________
Did your laboratory criteria for diagnosing maternal infection change over time?	◯ No<br>◯ Yes - changed 1x<br>◯ Yes - changed more than 1x
Which laboratory criteria did you apply subsequently? (Laboratory Definition 2)	________________________________
When did you begin using the revised lab criteria? (Laboratory Definition 2)	__________________________
What was the third set of laboratory criteria you applied? (Laboratory Definition 3)	________________________________
When did you begin using the third set of lab criteria? (Laboratory Definition 3)	__________________________
Were maternal samples retested using the new laboratory criteria?	◯ Yes<br>◯ No
If testing was performed for more than one arbovirus, including ZIKV, were they assessed simultaneously (i.e. multiplex testing) or separately?	◯ Yes, arboviruses tested simultaneously (multiplex)<br>◯ No, arboviruses tested separately

Confidential

Ascertainment of maternal DENV infection

Did the study assess pregnant women's DENV status?	○ Yes<br>○ No
What methods were used to assess maternal DENV status?<br><br>Select all that apply	☐ Clinical case definition<br>☐ Molecular diagnosis (e.g. RT-PCR, other NAAT)<br>☐ Immunoassay (e.g. ELISA, IFA, PRNT, microneutralization)<br>☐ Rapid diagnostic test (RDT)<br>☐ Other
What other method was used to assess maternal DENV status?	__________________________________
Were ZIKV and DENV assessed using separate tests?	○ DENV assessed separately from ZIKV<br>○ DENV and ZIKV assessed using multiplex testing
At any time, did your study screen for DENV and only test DENV (-) participants for ZIKV?	○ Yes, at some point study screened for DENV first, only tested DENV negative participants for ZIKV<br>○ No, study always tested for DENV and ZIKV simultaneously
If yes, when did your study BEGIN only administering ZIKV tests to DENV- participants?	___________________________
If yes, when did your study STOP only administering ZIKV tests to DENV- participants?	___________________________

Confidential

Ascertainment of maternal DENV infection - Immunoassay

DENV immunoassay analyte<br><br>Select all that apply	☐ IgM<br>☐ IgG<br>☐ IgA<br>☐ neutralizing antibodies<br>☐ Other
Other DENV immunoassay analyte	__________________________________
DENV immunoassay type<br><br>Select all that apply	☐ ELISA<br>☐ IFA<br>☐ MIA<br>☐ PRNT<br>☐ Other
Specify other type(s) or DENV immunoassay(s) performed	__________________________________
DENV immunoassay brand name<br><br>Select all that apply	☐ In house technique<br>☐ MAC-ELISA<br>☐ DENV Detect ELISA IgM/IgG (InBios International, United States)<br>☐ Dengue DxSelect IgM/IgG (Focus Diagnostics, USA)<br>☐ PATHOZYME-DENGUE IgM/IgG (Omega Diagnostics, UK)<br>☐ Dengue IgM/IgG Capture ELISA (Panbio Diagnostics, Australia)<br>☐ Dengue IgM/IgG Capture ELISA (Standard Diagnostics, South Korea)<br>☐ Platelia Dengue IgM/IgG ELISA" (Bio-Rad, France)<br>☐ SD Bioline Dengue IgM/IgG (Alere, United States)<br>☐ Arbovirus Fever Mosaic 2 IgG + IgM IFA ZIKV-DENV-CHIKV (EUROIMMUN)<br>☐ recomLine Tropical Fever IgG (MIKROGEN Diagnostik)<br>☐ recomLine Tropical Fever IgM (MIKROGEN Diagnostik)<br>☐ DPP Zika/Chikungunya/Dengue IgM/IgG Combination Assay (Chembio Diagnostic Systems Inc)<br>☐ STANDARD Q ZIKV/DENV/CHIKV Fast Quad (SD Biosensor)<br>☐ STANDARD Q ZIKV/DENV/CHIKV/YFV IgM Quad (SD Biosensor)<br>☐ Tell Me Fast zika/dengue/chikungunya combination rapid tests (Biocan Diagnostics Inc)<br>☐ Other
Which other immunoassays were used to diagnose maternal DENV infection?	__________________________________
What type of assay is the in house assay?<br><br>Select all that apply	☐ ELISA<br>☐ IFA<br>☐ MIA<br>☐ PRNT<br>☐ Other
What type of assay is the in house assay?	____

Confidential

What is the analyte measured by the in house assay?<br><br>Select all that apply	☐ IgM<br>☐ IgG<br>☐ IgA
What is the antigenic target for the in house assay<br><br>Select all that apply	☐ NS1<br>☐ envelope protein<br>☐ NS5<br>☐ Other
What is the antigenic target for the in house assay	__________________________________
For the in house DENV immunoassay, describe the testing procedure, including: incubation period, timing, type of cells, buffers, method to detect binding antibodies (e.g., optical density or secondary antibody detection), and methods to stop the enzymatic reaction	__________________________________
Sample used for DENV immunoassay<br><br>Select all that apply	☐ Serum/plasma<br>☐ Whole blood<br>☐ Urine<br>☐ Saliva<br>☐ Breast milk<br>☐ Umbilical cord tissue<br>☐ Placental tissue<br>☐ Amniotic fluid<br>☐ Other
Specify other type of sample used for DENV immunoassay	____________________________

Ascertainment of maternal DENV infection - ELISA

DENV ELISA cut-off value	__________________
Was a positive control from the outbreak included for the DENV ELISAs?	◯ Yes<br>◯ No
Was a negative control included with every run of DENV ELISAs?	◯ Yes<br>◯ No
Definition of POSITIVE DENV ELISA	______________________
Definition of NEGATIVE DENV ELISA	______________________
Definition of INCONCLUSIVE DENV ELISA	______________________
Source of definition used to interpret DENV ELISA test results<br><br>Select all that apply	☐ Manufacturer<br>☐ Ministry of Health reference lab<br>☐ WHO (global or local office e.g: PAHO)<br>☐ Consensus/study researchers<br>☐ Other
Specify other source of definition for DENV ELISA test results	__________________
Specify any additional inclusion or exclusion criteria for determining which samples to test for DENV using ELISA	______________________
Would your study be able to provide the P/N ratio, titer, or other quantitative readout for DENV ELISAs to inform the measurement error models?	◯ Yes<br>◯ No

Confidential

Ascertainment of maternal DENV infection - IFA

DENV IFA cut-off value	__________________
Was a positive control from the outbreak included for the DENV IFA?	◯ Yes<br>◯ No
Was a negative control included with every run of DENV IFA?	◯ Yes<br>◯ No
Definition of POSITIVE DENV IFA	__________________
Definition of NEGATIVE DENV IFA	__________________
Definition of INCONCLUSIVE DENV IFA	__________________
Source of definition used to interpret DENV IFA test results<br><br>Select all that apply	☐ Manufacturer<br>☐ Ministry of Health reference lab<br>☐ WHO (global or local office e.g: PAHO)<br>☐ Consensus/study researchers<br>☐ Other
Specify other source of definition for DENV IFA test results	__________________
Specify any additional inclusion or exclusion criteria for determining which samples to test for DENV using IFA	__________________
Will your study be able to provide the titer or optical density for DENV IFAs to inform the measurement error model?	◯ Yes<br>◯ No

Confidential

Ascertainment of maternal DENV infection - MIA

DENV MIA cut-off value	__________________
Was a positive control from the outbreak included for the DENV MIAs?	◯ Yes<br>◯ No
Was a negative control included with every run of DENV MIAs?	◯ Yes<br>◯ No
Definition of POSITIVE DENV MIA	__________________
Definition of NEGATIVE DENV MIA	__________________
Definition of INCONCLUSIVE DENV MIA	__________________
Source of definition used to interpret DENV MIA test results<br><br>Select all that apply	☐ Manufacturer<br>☐ Ministry of Health reference lab<br>☐ WHO (global or local office e.g: PAHO)<br>☐ Consensus/study researchers<br>☐ Other
Specify other source of definition for DENV MIA test results	__________________
Specify any additional inclusion or exclusion criteria for determining which samples to test for DENV using MIA	__________________
Will your study be able to provide the titer for DENV MIAs to inform the measurement error model?	◯ Yes<br>◯ No

Ascertainment of maternal DENV infection - Immunoassay - PRNT

Did you use the CDC protocol for the DENV PRNT?	◯ Yes<br>◯ No
Describe the other protocol used for the DENV PRNT?	__________________________________
Did your study simultaneously perform PRNT for ZIKV and DENV?	◯ Yes<br>◯ No
End-point PRNT titer	◯ PRNT50<br>◯ PRNT90<br>◯ Other
Which other end-point did you use for the PRNT titer?	___________________________
Definition of a positive PRNT titer	◯ PRNT>10<br>◯ Other
Other definition of a positive PRNT titer	___________________________
Definition of POSITIVE DENV PRNT	__________________________________
Definition of NEGATIVE DENV PRNT	__________________________________
Definition of INCONCLUSIVE DENV PRNT	__________________________________
Source of definition used to interpret DENV PRNT test results<br><br>Select all that apply	☐ Manufacturer<br>☐ Ministry of Health reference lab<br>☐ WHO (global or local office e.g: PAHO)<br>☐ US-CDC<br>☐ Consensus/study researchers<br>☐ Other
Specify other source of definition used to interpret DENV PRNT test results	___________________________
Specify any additional inclusion or exclusion criteria for determining which samples to test for DENV using PRNT	__________________________________
Where was the DENV PRNT test performed?<br><br>Select all that apply	☐ Lab at the same health care facility where study is conducted<br>☐ Study-specific lab<br>☐ Ministry of Health reference lab<br>☐ Private external lab<br>☐ Other

Confidential

Specify other lab where DENV PRNT test was performed	__________________________________
Will your study be able to provide the titer for DENV PRNTs to inform the measurement error model?	◯ Yes<br>◯ No
Can your study provide the full differences between the ZIKV and DENV titers or information about the magnitude of the difference? (e.g. is the difference 4 fold or greater? Is the difference 1 or less than 1?)	◯ Yes<br>◯ No<br>◯ Unsure

Ascertainment of maternal DENV infection - Molecular assay

Type of DENV PCR/RT-PCR performed<br><br>Select all that apply	☐ PCR (conventional or standard)<br>☐ qRT-PCR<br>☐ rRT-PCR<br>☐ No DENV PCR performed
DENV PCR/RT-PCR brand name<br><br>Select all that apply	☐ In house procedure<br>☐ CDC DENV-1-4 rRT-PCR Multiplex and Trioplex rRT-PCR Assays (CDC-USA)<br>☐ CDC DENV-1-4 Real-Time RT-PCR Multiplex Assay (CDC-USA)<br>☐ CDC Trioplex rRT-PCR assay (CDC-USA)<br>☐ Dengue Fever Virus & Chikungunya Virus Real Time RT-PCR Kit (Creative Biogene)<br>☐ Dengue Virus General-type Real Time RT-PCR Kit (Creative Biogene)<br>☐ RealStar Dengue RT-PCR (Altona Diagnostics-Hamburg,Germany)<br>☐ GenoAmp Trioplex Real-Time RT-PCR Zika/Den/Chiku (Medical Innovation Ventures)<br>☐ GenoAmp Real-Time RT-PCR Dengue (Serotyping of Dengue 1-Dengue 4) (Medical Innovation Ventures)<br>☐ RT-PCR kit (RealArt; artus/Qiagen-Germany)<br>☐ SimplexaTMdengue RT-PCR assay (Focus Diagnostics-Cypress, CA)<br>☐ Dengue virus general type real-time RT-PCR kit LiferiverTM (Shanghai ZJ Bio-Tech Co-China)<br>☐ Geno-Sen's dengue 1-4 real-time RT-PCR kit (Genome Diagnostics Pvt-New Delhi, India)<br>☐ CII-ArboViroPlex rRT-PCR assay (Columbia University)<br>☐ AccuPower ZIKV (DENV, CHIKV) Multiplex Real-Time RT-PCR kit (Bioneer)<br>☐ VIASURE Zika, Dengue & Chikungunya Real Time PCR Detection Kit (Certest Biotec)<br>☐ FTD Zika/Dengue/Chik (Fast-Track Diagnostics)<br>☐ DiaPlexQ ZCD (ZIKV, CHIKV, DENV) Detection Kit (SolGent Co Ltd)<br>☐ TaqMan Zika Virus Triplex Kit (Thermo Fisher Scientific)<br>☐ Genesig Multiplex Kit for Dengue, Chikungunya and Zika Virus (Primerdesign Ltd)<br>☐ Other
Describe procedure for in house DENV PCR/RT-PCR	________________________________
Specify other DENV PCR/RT-PCR used	________________________________
Sample used for DENV PCR/RT-PCR<br><br>Select all that apply	☐ Serum/plasma<br>☐ Whole blood<br>☐ Urine<br>☐ Saliva<br>☐ Breast milk<br>☐ Umbilical cord tissue<br>☐ Placental tissue<br>☐ Amniotic fluid<br>☐ Other

Confidential

Specify other type of sample used for DENV PCR/RT-PCR	__________________________________
Did the study obtain a measure of DENV viral load?	◯ Yes<br>◯ No
DENV PCR/RT-PCR cut-off point	__________________________________
Was a negative control included with every run of DENV PCR/RT-PCRs?	◯ Yes<br>◯ No
Definition of POSITIVE DENV PCR/RT-PCR	__________________________________
Definition of NEGATIVE DENV PCR/RT-PCR	__________________________________
Definition of INCONCLUSIVE DENV PCR/RT-PCR	__________________________________
Source of definition used to interpret DENV PCR/RT-PCR test results<br><br>Select all that apply	☐ Manufacturer<br>☐ Ministry of Health reference lab<br>☐ WHO (global or local office e.g: PAHO)<br>☐ Consensus/study researchers<br>☐ Other
Specify other source of definition for DENV PCR/RT-PCR test results	__________________________________
Specify any additional inclusion or exclusion criteria for determining which samples to test for DENV using PCR/RT-PCR	__________________________________
Would your study be able to provide a Ct value for DENV PCR/RT-PCRs to inform the measurement error models?	◯ Yes<br>◯ No

Carabali M, *et al. BMJ Open* 2022; 12:e064362. doi: 10.1136/bmjopen-2022-064362

DENV testing algorithm & qualitative definitions

What assay was used to confirm maternal DENV infection?	◯ NAAT (PCR) only<br>◯ NS-1 only<br>◯ IgM only<br>◯ IgG increase in paired samples<br>◯ IgM seroconversion in paired samples<br>◯ PRNT + any serologic test (IgM, IgG, or both)<br>◯ PCR or any NAAT + any serologic test (IgM, IgG or both)<br>◯ Other combination of assays
Specify other combination of assays used to confirm maternal DENV infection	__________________________________
Considering any relevant clinical and laboratory criteria, what was the study's definition of a CONFIRMED maternal DENV infection	__________________________________
Considering any relevant clinical and laboratory criteria, what was the study's definition of a PROBABLE maternal DENV infection	__________________________________
Considering any relevant clinical and laboratory criteria, what was the study's definition of a NEGATIVE/UNLIKELY maternal DENV infection?	__________________________________

Confidential

Ascertainment of maternal CHIKV infection

Did the study conduct chikv-specific laboratory tests?	◯ Yes, chikv assessed separately from ZIKV<br>◯ Yes, chikv assessed using multiplex testing<br>◯ No, chikv not assessed
What methods were used to assess maternal CHIKV status?<br><br>Select all that apply	☐ Clinical case definition<br>☐ Molecular diagnosis (e.g. RT-PCR, other NAAT)<br>☐ Immunoassay (e.g. ELISA, IFA, PRNT, microneutralization)<br>☐ Rapid diagnostic test (RDT)<br>☐ Other
What other method was used to assess maternal CHIKV status?	__________________________________
CHIKV immunoassay analyte<br><br>Select all that apply	☐ IgM<br>☐ IgG<br>☐ IgA<br>☐ neutralizing antibodies<br>☐ Other
Other CHIKV immunoassay analyte	__________________________
CHIKV immunoassay type<br><br>Select all that apply	☐ ELISA<br>☐ IFA<br>☐ MIA<br>☐ PRNT<br>☐ Other
Specify other type(s) or CHIKV immunoassay(s) performed	__________________________

Carabali M, *et al. BMJ Open* 2022; 12:e064362. doi: 10.1136/bmjopen-2022-064362

Confidential

CHIKV immunoassay brand name<br><br>Select all that apply	☐ In house technique<br>☐ MAC-ELISA<br>☐ CHIK IgM micro-capture ELISA (IBL International -Germany)<br>☐ RecombiLISA CHIK IgM Test (CTK Biotech-USA/China)<br>☐ CHIKV IgM µ-capture ELISA (Genway-Germany)<br>☐ Anti-CHIKV IgM human ELISA kit (Abcam-Germany)<br>☐ CHIKa IgM ELISA (SD Diagnostics South Korea)<br>☐ Anti-CHIKV ELISA (IgM) (Euroimmun-Germany)<br>☐ CHIKjj Detect MAC-ELISA (Inbios-USA)<br>☐ anti-chikungunya virus ELISA (IgM) kit (Euroimmun US-Mountain Lakes, NJ)<br>☐ Chikungunya IgM µ-capture ELISA (IBL International-Hamburg, Germany)<br>☐ Chikungunya IgG Capture ELISA (IBL International-Hamburg, Germany)<br>☐ Anti-Chikungunya Virus ELISA IgG test (Euroimmun-Lübeck, Germany)<br>☐ Arbovirus Fever Mosaic 2 IgG + IgM IFA ZIKV-DENV-CHIKV (EUROIMMUN)<br>☐ recomLine Tropical Fever IgG (MIKROGEN Diagnostik)<br>☐ recomLine Tropical Fever IgM (MIKROGEN Diagnostik)<br>☐ DPP Zika/Chikungunya/Dengue IgM/IgG Combination Assay (Chembio Diagnostic Systems Inc)<br>☐ STANDARD Q ZIKV/DENV/CHIKV Fast Quad (SD Biosensor)<br>☐ STANDARD Q ZIKV/DENV/CHIKV/YFV IgM Quad (SD Biosensor)<br>☐ Tell Me Fast zika/dengue/chikungunya combination rapid tests (Biocan Diagnostics Inc<br>☐ Other
Which other immunoassays were used to diagnose maternal CHIKV infection?	__________________________________
For in house CHIKV immunoassay, describe the testing procedure, including: incubation period, timing, type of cells, buffers, method to detect binding antibodies, and methods to stop the enzymatic reaction	__________________________________
Sample used for CHIKV immunoassay<br><br>Select all that apply	☐ Serum/plasma<br>☐ Whole blood<br>☐ Urine<br>☐ Saliva<br>☐ Breast milk<br>☐ Umbilical cord tissue<br>☐ Placental tissue<br>☐ Amniotic fluid<br>☐ Other
Specify other type of sample used in CHIKV immunoassay	__________________________________

Ascertainment of maternal CHIKV infection - ELISA

CHIKV ELISA cut-off value	______________________
Was a positive control from the outbreak included for the CHIKV ELISAs?	○ Yes<br>○ No
Was a negative control included with every run of CHIKV ELISAs?	○ Yes<br>○ No
Definition of POSITIVE CHIKV ELISA	______________________
Definition of NEGATIVE CHIKV ELISA	______________________
Definition of INCONCLUSIVE CHIKV ELISA	______________________
Source of definition used to interpret CHIKV ELISA test results<br><br>Select all that apply	☐ Manufacturer<br>☐ Ministry of Health reference lab<br>☐ WHO (global or local office e.g: PAHO)<br>☐ Consensus/study researchers<br>☐ Other
Specify other source of definition for CHIKV ELISA test results	______________________
Specify any additional inclusion or exclusion criteria for determining which samples to test for CHIKV using ELISA	______________________
Would your study be able to provide the P/N ratio, titer, or other quantitative readout for CHIKV ELISAs to inform the measurement error models?	○ Yes<br>○ No

Ascertainment of maternal CHIKV infection - IFA

CHIKV IFA cut-off value

____

Was a positive control from the outbreak included for the CHIKV IFA?

◯ Yes
◯ No

Was a negative control included with every run of CHIKV IFA?

◯ Yes
◯ No

Definition of POSITIVE CHIKV IFA

____

Definition of NEGATIVE CHIKV IFA

____

Definition of INCONCLUSIVE CHIKV IFA

____

Source of definition used to interpret CHIKV IFA test results

Select all that apply

☐ Manufacturer
☐ Ministry of Health reference lab
☐ WHO (global or local office e.g: PAHO)
☐ Consensus/study researchers
☐ Other

Specify other source of definition for CHIKV IFA test results

____

Specify any additional inclusion or exclusion criteria for determining which samples to test for CHIKV using IFA

____

Will your study be able to provide the optical density or titer for CHIKV IFAs to inform the measurement error model?

◯ Yes
◯ No

Confidential

Ascertainment of maternal CHIKV infection - MIA

CHIKV MIA cut-off value

____

Was a positive control from the outbreak included for the CHIKV MIAs?

◯ Yes
◯ No

Was a negative control included with every run of CHIKV MIAs?

◯ Yes
◯ No

Definition of POSITIVE CHIKV MIA

____

Definition of NEGATIVE CHIKV MIA

____

Definition of INCONCLUSIVE CHIKV MIA

____

Source of definition used to interpret CHIKV MIA test results

Select all that apply

☐ Manufacturer
☐ Ministry of Health reference lab
☐ WHO (global or local office e.g: PAHO)
☐ Consensus/study researchers
☐ Other

Specify other source of definition for CHIKV MIA test results

____

Specify any additional inclusion or exclusion criteria for determining which samples to test for CHIKV using MIA

____

Will your study be able to provide the titer for CHIKV MIAs to inform the measurement error model?

◯ Yes
◯ No

Ascertainment of maternal CHIKV infection - Molecular assay

Type of CHIKV PCR/RT-PCR performed<br><br>Select all that apply	☐ PCR (standard or conventional)<br>☐ qRT-PCR<br>☐ rRT-PCR<br>☐ No CHIKV PCR performed
CHIKV PCR/RT-PCR brand name<br><br>Select all that apply	☐ In house procedure<br>☐ CHIKV CDC qRT-PCR (CDC-USA)<br>☐ CDC Trioplex rRT-PCR Assay (CDC-USA)<br>☐ RealStar Chikungunya RT-PCR Kit (Altona Diagnostics-Germany)<br>☐ Genesig Chikungunya Non structural protein 2 standard (nsp2) RT-PCR kit (Primerdesign-UK)<br>☐ CII-ArboViroPlex rRT-PCR assay (Columbia University)<br>☐ AccuPower ZIKV (DENV, CHIKV) Multiplex Real-Time RT-PCR kit (Bioneer)<br>☐ VIASURE Zika, Dengue & Chikungunya Real Time PCR Detection Kit (Certest Biotec)<br>☐ FTD Zika/Dengue/Chik (Fast-Track Diagnostics)<br>☐ DiaPlexQ ZCD (ZIKV, CHIKV, DENV) Detection Kit (SolGent Co Ltd)<br>☐ TaqMan Zika Virus Triplex Kit (Thermo Fisher Scientific)<br>☐ Dengue Fever Virus & Chikungunya Virus Real Time RT-PCR Kit (Creative Biogene)<br>☐ GenoAmp Trioplex Real-Time RT-PCR Zika/Den/Chiku (Medical Innovation Ventures)<br>☐ Genesig Multiplex Kit for Dengue, Chikungunya and Zika Virus (Primerdesign Ltd)<br>☐ Other
Describe procedure for in house PCR/RT-PCR	______________________________
Specify other CHIKV PCR/RT-PCR brand used	__________________________
Sample used for CHIKV PCR/RT-PCR<br><br>Select all that apply	☐ Serum/plasma<br>☐ Whole blood<br>☐ Urine<br>☐ Saliva<br>☐ Breast milk<br>☐ Umbilical cord tissue<br>☐ Placental tissue<br>☐ Amniotic fluid<br>☐ Other
Specify other type of sample used for CHIKV PCR/RT-PCR	__________________________
Did the study obtain a measure of CHIKV viral load?	◯ Yes<br>◯ No
CHIKV PCR/RT-PCR cut-off point	__________________________
Was a negative control included with every run of CHIKV PCR/RT-PCRs?	◯ Yes<br>◯ No

Definition of POSITIVE CHIKV PCR/RT-PCR	__________________________
Definition of NEGATIVE CHIKV PCR/RT-PCR	__________________________
Definition of INCONCLUSIVE CHIKV PCR/RT-PCR	__________________________
Source of the definition used to interpret CHIKV PCR/RT-PCR test results<br><br>Select all that apply	☐ Manufacturer<br>☐ Ministry of Health reference lab<br>☐ WHO (global or local office e.g: PAHO)<br>☐ US-CDC<br>☐ Consensus/study researchers<br>☐ Other
Specify other source of the definition used to interpret CHIKV PCR/RT-PCR test results	____________________
Specify any additional inclusion or exclusion criteria for determining which samples to test for CHIKV using PCR/RT-PCR	__________________________
Where was the CHIKV PCR/RT-PCR test performed?<br><br>Select all that apply	☐ Lab at the same health care facility where study is conducted<br>☐ Study-specific lab<br>☐ Ministry of Health reference lab<br>☐ Private external lab<br>☐ Other
Specify other lab where the CHIKV PCR/RT-PCR test was performed	____________________

CHIKV testing algorithm & qualitative definitions

What tests were used to confirm CHIKV infection in pregnant women?<br><br>Select all that apply	☐ CHIKV IgM - 1 sample<br>☐ CHIKV IgM - paired samples (seroconversion or increased titers)<br>☐ CHIKV IgM (1 sample) + IgG<br>☐ CHIKV IgM (paired samples) + IgG<br>☐ RT-PCR or other NAAT<br>☐ PRNT > 10<br>☐ Other
Specify other test or combination of tests used to confirm CHIKV infection	__________________________________
Considering any relevant clinical and laboratory criteria, what was the study's definition of a CONFIRMED maternal CHIKV infection	__________________________________
Considering any relevant clinical and laboratory criteria, what was the study's definition of a PROBABLE maternal CHIKV infection	__________________________________
Considering any relevant clinical and laboratory criteria, what was the study's definition of a NEGATIVE maternal CHIKV infection	__________________________________

Confidential

Ascertainment of maternal infection with other flavi/alphaviruses

Does the study capture information on maternal history of any other flavivirus/alphavirus infections?	◯ Yes, self-reported<br>◯ Yes, lab assessment<br>◯ Yes, both self-reported and lab assessment<br>◯ No
Does the study capture information on maternal history of yellow fever or Japanese encephalitis vaccination?	◯ Yes<br>◯ No
Was testing performed for other current maternal arbovirus infections? If so, which ones?<br><br>Select all that apply	☐ DENV<br>☐ CHIKV<br>☐ Japanese/Venezuelan encephalitis<br>☐ Mayaro virus<br>☐ West Nile virus<br>☐ Other arboviruses<br>☐ No, study did not test for concurrent arbovirus infections
Which other current maternal arboviral infections did the study test for?	______________________________

Ascertainment of placental or fetal ZIKV infection

Was placental ZIKV infection measured?	◯ Yes<br>◯ No
Was fetal ZIKV infection measured?	◯ Yes<br>◯ No
Which criteria did you use to infer fetal ZIKV status?<br><br>Select all that apply	☐ fetal clinical criteria<br>☐ fetal laboratory criteria<br>☐ fetal imaging criteria<br>☐ maternal clinical criteria<br>☐ maternal laboratory criteria<br>☐ placental clinical criteria<br>☐ placental laboratory criteria<br>☐ other criteria
In what other way was fetal ZIKV infection assessed?	______________________________
Which laboratory tests were used to assess fetal ZIKV status?<br><br>Select all that apply	☐ molecular diagnosis (e.g. RT-PCR, other NAAT)<br>☐ immunoassay (e.g. ELISA, IFA, PRNT, IHC, microneutralization)<br>☐ rapid diagnostic test (RDT)<br>☐ Other
Which other laboratory tests were used to assess fetal ZIKV status?	______________________________
Were the assays or the clinical criteria used to assess fetal ZIKV infection the same as the assays or clinical criteria used to assess placental ZIKV infection?	◯ Yes<br>◯ No
Please describe the differences between the clinical criteria or assays used to assess fetal and placental ZIKV infection	________________________
When was fetal ZIKV infection measured using laboratory criteria?<br><br>Select all that apply	☐ 1st trimester<br>☐ 2nd trimester<br>☐ 3rd trimester<br>☐ First antenatal visit<br>☐ Following confirmation of maternal ZIKV infection, irregardless of trimester<br>☐ Other
Type of sample(s) obtained to ascertain fetal ZIKV infection<br><br>Select all that apply	☐ Amniotic fluid<br>☐ Placental tissue (Including umbilical cord and fetal membranes)<br>☐ Fetal tissue<br>☐ Other
What other type(s) of samples were obtained to ascertain fetal ZIKV infection?	________________________

Supplemental material
BMJ Publishing Group Limited (BMJ) disclaims all liability and responsibility arising from any reliance placed on this supplemental material which has been supplied by the author(s)
BMJ Open

At what other time was fetal ZIKV infection measured?	__________________________________
Where was the fetal sample analyzed?<br><br>Select all that apply	☐ Lab at the same health care facility where study is conducted<br>☐ Study-specific lab<br>☐ Ministry of Health reference lab<br>☐ Private external lab<br>☐ Other
Specify other lab where the fetal sample was analyzed	____________________________
Did you use different assays, different brands of the same assay, or different versions of the same brand of assay at different study sites to ascertain fetal ZIKV status or over time within the study?<br><br>Select all that apply	☐ Yes, different versions of the same brand of assay over time<br>☐ Yes, different brands of the same type of assay over time<br>☐ Yes, different types of assays over time<br>☐ Yes, different versions of the same brand of assay at different sites<br>☐ Yes, different brands of the same type of assay at different sites<br>☐ Yes, different types of assays at different sites<br>☐ No, same tests applied throughout the project
Which different assay(s) did you use over time or across sites?	__________________________________
Which different brands of the assay did you use over time or across sites?	__________________________________
Which different versions of the brand of assay did you use over time or across sites?	__________________________________

Carabali M, *et al. BMJ Open* 2022; 12:e064362. doi: 10.1136/bmjopen-2022-064362

Ascertainment of fetal ZIKV infection - Immunoassay

ZIKV immunoassay analyte to ascertain fetal infection<br><br>Select all that apply	☐ IgM<br>☐ IgG<br>☐ IgA<br>☐ neutralizing antibodies<br>☐ Other
Other ZIKV immunoassay analyte to ascertain fetal infection	__________________________________
ZIKV immunoassay type to ascertain fetal infection<br><br>Select all that apply	☐ ELISA<br>☐ IFA<br>☐ MIA<br>☐ PRNT<br>☐ Other
Specify other type(s) or ZIKV immunoassay(s) performed to ascertain fetal infection	__________________________________

ZIKV immunoassay brand name<br><br>Select all that apply	☐ In house-developed assay<br>☐ Zika MAC-ELISA (CDC)<br>☐ ZIKV Detect 2.0 IgM Capture ELISA Kit (InBios International Inc)<br>☐ Zika IgM ELISA (EUROIMMUN)<br>☐ Zika IgM + IgA ELISA (EUROIMMUN)<br>☐ Zika IgG ELISA (EUROIMMUN)<br>☐ ZIKV IgM IFA (EUROIMMUN)<br>☐ ZIKV IgG IFA (EUROIMMUN)<br>☐ Arbovirus Fever Mosaic 2 IgG + IgM IFA ZIKV-DENV-CHIKV (EUROIMMUN)<br>☐ ADVIA Centaur Zika Test (Siemens Healthcare Diagnostics)<br>☐ DPP Zika IgM Assay System (Chembio Diagnostic Systems Inc)<br>☐ DPP Zika IgM/IgG System (Chembio Diagnostic Systems Inc)<br>☐ DPP Zika/Chikungunya/Dengue IgM/IgG Combination Assay (Chembio Diagnostic Systems Inc)<br>☐ LIAISON XL Zika Capture IgM II assay (DiaSorin S.p.A.)<br>☐ STANDARD Q Zika IgM/IgG test (SD Biosensor)<br>☐ STANDARD E Zika IgM ELISA (SD Biosensor)<br>☐ STANDARD Q ZIKV/DENV/CHIKV Fast Quad (SD Biosensor)<br>☐ STANDARD Q ZIKV/DENV/CHIKV/YFV IgM Quad (SD Biosensor)<br>☐ Tell Me Fast Zika IgG/IgM Antibody Rapid Test (Biocan Diagnostics Inc)<br>☐ Tell Me Fast zika/dengue/chikungunya combination rapid tests (Biocan Diagnostics Inc)<br>☐ ZIKV-DENV-CHIKV IFA IgG (Vircell)<br>☐ ZIKV-DENV-CHIKV IFA IgM (Vircell)<br>☐ Human Anti-Zika ELISA Kits (Alpha Diagnostic International)<br>☐ anti-Zika virus IgM ELISA kit (µ-capture) (AbCam)<br>☐ anti-Zika virus IgG ELISA kit (AbCam)<br>☐ Zika Virus IgM ELISA Assay Kit (Eagle Biosciences)<br>☐ Zika Virus IgG ELISA Assay Kit (Eagle Biosciences)<br>☐ Human Anti-Zika Virus IgG ELISA Kit (R&D Systems)<br>☐ ZIKV IgG ELISA kit (DIA.PRO Diagnostic Bioprobes Srl)<br>☐ ZIKV IgM ELISA kit (DIA.PRO Diagnostic Bioprobes Srl)<br>☐ NovaLisa Zika Virus IgM µ-capture ELISA (NovaTec Immunodiagnostica GmbH)<br>☐ recomLine Tropical Fever IgG (MIKROGEN Diagnostik)<br>☐ recomLine Tropical Fever IgM (MIKROGEN Diagnostik)<br>☐ Other
Which other immunoassays were used to diagnose fetal ZIKV infection?	__________________________________
What type of assay is the in house assay (to ascertain fetal infection)?<br><br>Select all that apply	☐ ELISA<br>☐ IFA<br>☐ MIA<br>☐ PRNT<br>☐ Other
What type of assay is the in house assay (to ascertain fetal infection)?	__________________________________

What is the analyte measured by the in house assay (to ascertain fetal infection)?<br><br>Select all that apply	☐ IgM<br>☐ IgG<br>☐ IgA
What is the antigenic target for the in house assay (to ascertain fetal infection)?<br><br>Select all that apply	☐ NS1<br>☐ envelope protein<br>☐ NS5<br>☐ Other
What is the antigenic target for the in house assay (to ascertain fetal infection)?	________________________
For the in house ZIKV immunoassay to ascertain fetal infection, describe the testing procedure, including: incubation period, timing, type of cells, buffers, method to detect binding antibodies (e.g., optical density or secondary antibody detection), and methods to stop the enzymatic reaction	________________________
Sample used for ZIKV immunoassay (to ascertain fetal infection)<br><br>Select all that apply	☐ Serum/plasma<br>☐ Whole blood<br>☐ cerebrospinal fuid (CSF)<br>☐ chord blood<br>☐ Other
Specify other sample used for ZIKV immunoassay (to ascertain fetal infection)	____________________
ZIKV ELISA tested in paired/subsequent samples for ascertaining fetal infection	○ Yes<br>○ No
At what temperature was the ZIKV ELISA test stored for fetal infection? (degrees Celsius)	____________________
At what temperature(s) was/were the ZIKV ELISA sample(s) stored for fetal infection? (degrees Celsius)	________________________<br>(Include sample type for each temperature)
In what volume(s) was/were the ZIKV ELISA sample aliquot(s) stored for fetal infection? (mL)	________________________<br>(Include sample type with each volume)
Did your study deviate from the directions provided in the ZIKV ELISA commercial kit for fetal infection?	○ Yes<br>○ No<br>○ Study did not use a commercial ZIKV ELISA kit
Describe any deviations from the procedure recommended by the commercial ELISA kit, including: incubation period, timing, type of cells, buffers, method to detect bounding antibodies, and methods to stop the enzymatic reaction	________________________

Describe any additional conditions for ZIKV ELISA test administration for ascertaining fetal infection (e.g. paired samples within 21 days of fever onset; count with more than 2ul of serum from the febrile phase, etc.)	__________________________________
Where was the ZIKV ELISA test performed?<br><br>Select all that apply	☐ Lab at the same health care facility where study is conducted<br>☐ Study-specific lab<br>☐ Ministry of Health reference lab<br>☐ Private external lab<br>☐ Other
Specify other lab where ZIKV ELISA was performed for fetal infection	___________________________

Ascertainment of fetal ZIKV infection - ELISA

ZIKV ELISA cut-off point for fetal infection ________________

Was a positive control from the outbreak included for the ZIKV ELISAs for fetal infection?
- ◯ Yes
- ◯ No

Was a negative control included with every run of ZIKV ELISAs for fetal infection?
- ◯ Yes
- ◯ No

Definition of POSITIVE ZIKV ELISA for fetal infection ________________

Definition of NEGATIVE ZIKV ELISA for fetal infection ________________

Definition of INCONCLUSIVE ZIKV ELISA for fetal infection ________________

Source of definition used to interpret ZIKV immunoassay test results

Select all that apply
- ☐ Manufacturer
- ☐ Ministry of Health reference lab
- ☐ WHO (global or local office e.g: PAHO)
- ☐ US-CDC
- ☐ Consensus/study researchers
- ☐ Other

Specify other source of definition used to interpret ZIKV immunoassay test results ________________

How many ZIKV ELISAs did the study plan to conduct in each fetus? ________________

Would your study be able to provide the P/N ratio, titer, or other quantitative readout for ZIKV ELISAs to inform the measurement error models?
- ◯ Yes
- ◯ No

Confidential

Ascertainment of fetal ZIKV infection - IFA

ZIKV IFA cut-off value for fetal infection

____

Was a positive control from the outbreak included for the ZIKV IFA for fetal infection?
- ◯ Yes
- ◯ No

Was a negative control included with every run of ZIKV IFA for fetal infection?
- ◯ Yes
- ◯ No

Definition of POSITIVE ZIKV IFA for fetal infection

____

Definition of NEGATIVE ZIKV IFA for fetal infection

____

Definition of INCONCLUSIVE ZIKV IFA for fetal infection

____

Source of definition for ZIKV IFA test results for fetal infection

Select all that apply
- ☐ Manufacturer
- ☐ Ministry of Health reference lab
- ☐ WHO (global or local office e.g: PAHO)
- ☐ Consensus/study researchers
- ☐ Other

Specify other source of definition for ZIKV IFA test results

____

Specify any additional inclusion or exclusion criteria for determining which samples to test for ZIKV using IFA

____

Will your study be able to provide the titer or optical density for ZIKV IFAs to inform the measurement error model?
- ◯ Yes
- ◯ No

Confidential

Ascertainment of fetal ZIKV infection - MIA

ZIKV MIA cut-off value for fetal infection ________________________

Was a positive control from the outbreak included for the ZIKV MIAs for fetal infection?
◯ Yes
◯ No

Was a negative control included with every run of ZIKV MIAs for fetal infection?
◯ Yes
◯ No

Definition of POSITIVE ZIKV MIA for fetal infection ________________________

Definition of NEGATIVE ZIKV MIA for fetal infection ________________________

Definition of INCONCLUSIVE ZIKV MIA for fetal infection ________________________

Source of definition for MIA ZIKV test results for fetal infection

Select all that apply

☐ Manufacturer
☐ Ministry of Health reference lab
☐ WHO (global or local office e.g: PAHO)
☐ Consensus/study researchers
☐ Other

Specify other source of definition for ZIKV MIA test results ________________________

Specify any additional inclusion or exclusion criteria for determining which samples to test for ZIKV using MIA for fetal infection ________________________

Will your study be able to provide the titer for ZIKV MIAs to inform the measurement error model?
◯ Yes
◯ No

Ascertainment of fetal ZIKV infection - Molecular assay

Type of ZIKV molecular tests performed on fetus<br><br>Select all that apply	☐ conventional PCR<br>☐ rRT-PCR<br>☐ qRT-PCR<br>☐ Other NAAT
According to the study protocol, when were the ZIKV samples to be collected for molecular testing?<br><br>Select all that apply	☐ within 24 hours after birth<br>☐ within first week after birth<br>☐ Other
When were the ZIKV molecular tests (RT-PCR, other NAAT) conducted on fetus?	__________________________________
Sample used for ZIKV RT-RCR/PCR in fetus<br><br>Select all that apply	☐ Serum/plasma<br>☐ Whole blood<br>☐ Urine<br>☐ Saliva<br>☐ Breast milk<br>☐ Umbilical cord tissue<br>☐ cerebrospinal fuid (CSF)<br>☐ chord blood<br>☐ Other
Specify other type of sample used for ZIKV RT-PCR/PCR on fetus	___________________________

ZIKV RT-PCR/PCR brand name used for testing fetus<br><br>Select all that apply	☐ In house technique<br>☐ Zika Virus RNA Qualitative Real-Time RT-PCR (Quest Diagnostics)<br>☐ RealStar Zika Virus RT-PCR Kit U.S. (altona Diagnostics GmbH)<br>☐ RealStar Zika Virus RT-PCR Kit 1.0 (altona Diagnostics GmbH)<br>☐ Aptima Zika Virus assay (Hologic Inc)<br>☐ Zika Virus Real-time RT-PCR (Viracor Eurofins)<br>☐ VERSANT Zika RNA 1.0 Assay (kPCR) (Siemens Healthcare Diagnostics Inc)<br>☐ xMAP MultiFLEX Zika RNA Assay (Luminex Corporation)<br>☐ Sentosa SA ZIKV RT-PCR Test (Vela Diagnostics Inc)<br>☐ Zika Virus Detection by RT-PCR (ARUP Laboratories)<br>☐ Abbott RealTime Zika (Abbott Molecular Inc)<br>☐ Zika ELITe MGB Kit U.S. (ELITechGroup Molecular Diagnostics)<br>☐ Gene-RADAR Zika Virus Test (Nanobiosym Diagnostics Inc)<br>☐ TaqPath Zika Virus Kit (Thermo Fisher Scientific)<br>☐ CII-ArboViroPlex rRT-PCR assay (Columbia University)<br>☐ Trioplex Real-Time RT-PCR Assay (CDC)<br>☐ Liferiver Zika Virus (ZIKV) Real Time RT-PCR Kit (Shanghai ZJ Bio-Tech Co Ltd)<br>☐ careGENE Zika Virus RT-PCR Kit (WELLS BIO Inc)<br>☐ Zika Virus - Single Check (Genekam Biotechnology AG)<br>☐ FTD Zika virus (Fast Track Diagnostics)<br>☐ VIASURE Zika Virus Real Time PCR Detection Kit (Certest Biotec)<br>☐ Zika Virus Real-TM (Sacace Biotechnologies)<br>☐ Logix Smart Zika Test Kit (Co-Diagnostics Inc)<br>☐ Genesig Kits for ZIKV (Primerdesign Ltd)<br>☐ Genesig Multiplex Kit for Dengue, Chikungunya and Zika Virus (Primerdesign Ltd)<br>☐ AccuPower ZIKV (DENV, CHIKV) Multiplex Real-Time RT-PCR Kit (Bioneer)<br>☐ VIASURE Zika, Dengue & Chikungunya Real Time PCR Detection Kit (Certest Biotec)<br>☐ FTD Zika/Dengue/Chik (Fast-Track Diagnostics)<br>☐ DiaPlexQ ZCD (ZIKV, CHIKV, DENV) Detection Kit (SolGent Co Ltd)<br>☐ TaqMan Zika Virus Triplex Kit (Thermo Fisher Scientific)<br>☐ GenoAmp Trioplex Real-Time RT-PCR Zika/Den/Chiku (Medical Innovation Ventures)<br>☐ Other
Describe procedure for in house ZIKV RT-PCR/PCR for fetal infection	__________________________________
Specify other ZIKV RT-PCR/PCR brand name	__________________________________
Did the study obtain a measure of ZIKV viral load for fetal infection?	◯ Yes<br>◯ No
ZIKV PCR/RT-PCR cut-off point for fetal infection	____________________________

Confidential

Was a negative control included with every run of ZIKV RT-PCR/PCRs for fetal infection?	◯ Yes<br>◯ No
Definition of POSITIVE ZIKV RT-PCR/PCR for fetal infection	__________________________________
Definition of NEGATIVE ZIKV RT-PCR/PCR for fetal infection	__________________________________
Definition of INCONCLUSIVE ZIKV RT-PCR/PCR for fetal infection	__________________________________
Source of definition used to interpret ZIKV RT-PCR/PCR test results for fetal infection<br><br>Select all that apply	☐ Manufacturer<br>☐ Ministry of Health reference lab<br>☐ WHO (global or local office e.g: PAHO)<br>☐ US-CDC<br>☐ Consensus/study researchers<br>☐ Other
Specify other source of definition used to interpret ZIKV RT-PCR/PCR test results	___________________________
Where was the ZIKV RT-PCR/PCR test performed for samples from fetus?<br><br>Select all that apply	☐ Lab at the same health care facility where study is conducted<br>☐ Study-specific lab<br>☐ Ministry of Health reference lab<br>☐ Private external lab<br>☐ Other
Specify other lab where ZIKV RT-PCR/PCR test was performed for fetal samples	___________________________
Specify any additional inclusion or exclusion criteria for determining which samples from fetus to test for ZIKV using RT-PCR/PCR	__________________________________
How many ZIKV PCRs were conducted with with each fetus?	___________________________
Would your study be able to provide a Ct value for fetal ZIKV PCRs to inform the measurement error models?	◯ Yes<br>◯ No

Confidential

Changes in diagnosis of fetal ZIKV infection over time

Which clinical criteria did you apply initially to diagnose fetal infection? (Clinical Definition 1) ________________________________

Did your clinical criteria for fetal infection change over time?
- ◯ No
- ◯ Yes - changed 1x
- ◯ Yes - changed more than 1x

If your clinical criteria changed over time, which criteria did you apply subsequently to assess fetal infection? (Clinical Definition 2) ________________________________

When did you begin using the revised clinical criteria for assessing fetal infection? (Clinical Definition 2) ________________________

What was the third set of criteria you applied to assess fetal infection? (Clinical Definition 3) ________________________________

When did you begin using the third set of clinical criteria? (Clinical Definition 3) ________________________

Which imaging criteria did you apply initially to diagnose fetal infection? (Clinical Definition 1) ________________________________

Did your imaging criteria for fetal infection change over time?
- ◯ No
- ◯ Yes - changed 1x
- ◯ Yes - changed more than 1x

If your imaging criteria changed over time, which criteria did you apply subsequently to assess fetal infection? (Clinical Definition 2) ________________________________

When did you begin using the revised imaging criteria for assessing fetal infection? (Clinical Definition 2) ________________________

What was the third set of imaging criteria you applied to assess fetal infection? (Clinical Definition 3) ________________________________

When did you begin using the third set of imaging criteria? (Clinical Definition 3) ________________________

What laboratory criteria was used initially to diagnose fetal ZIKV infection? (Laboratory Definition 1) ________________________________

Did your laboratory criteria for diagnosing fetal infection change over time?
- ◯ No
- ◯ Yes - changed 1x
- ◯ Yes - changed more than 1x

Carabali M, *et al. BMJ Open* 2022; 12:e064362. doi: 10.1136/bmjopen-2022-064362

Confidential

Which laboratory criteria did you apply subsequently? (Laboratory Definition 2)	________________________________
When did you begin using the revised lab criteria for fetal infection? (Laboratory Definition 2)	__________________________
What was the third set of laboratory criteria you applied for diagnosing fetal infection? (Laboratory Definition 3)	________________________________
When did you begin using the third set of lab criteria for fetal infection? (Laboratory Definition 3)	__________________________
Were fetal samples retested using the new laboratory criteria?	________________________________

ZIKV testing algorithm & test interpretation - fetal ZIKV infection

Describe the testing algorithm for defining a confirmed ZIKV+ test in fetal testing	__________________________________
Describe the testing algorithm for defining a confirmed ZIKV- test in fetal testing	__________________________________
What tests were used to confirm ZIKV infection in fetal testing?<br><br>Select all that apply	☐ ZIKV IgM - 1 sample<br>☐ ZIKV IgM - paired samples (seroconversion or increased titers)<br>☐ ZIKV IgM (1 sample) + IgG<br>☐ ZIKV IgM (paired samples) + IgG<br>☐ RT-PCR or other NAAT<br>☐ PRNT > 10<br>☐ Other
Specify other test or combination of tests used to confirm ZIKV infection in fetal testing	__________________________________
Considering any relevant clinical and laboratory criteria, what was the study's definition of a CONFIRMED fetal ZIKV Infection	__________________________________
Considering any relevant clinical and laboratory criteria, what was the study's definition of a PROBABLE fetal ZIKV Infection	__________________________________
Considering any relevant clinical and laboratory criteria, what was the study's definition of a NEGATIVE/UNLIKELY fetal ZIKV Infection	__________________________________
Source of ZIKV test result categorization for fetal testing<br><br>Select all that apply	☐ Manufacturer<br>☐ Ministry of Health reference lab<br>☐ WHO (global or local office e.g: PAHO)<br>☐ Consensus/study researchers<br>☐ Other
Specify other source of ZIKV test result categorization for fetal testing	___________________________

Fetal Imaging & Ascertainment of Prenatal Microcephaly

Was fetal ultrasound performed?	◯ Yes<br>◯ No
How many fetal ultrasounds did the study plan to conduct for each pregnant woman?	__________________________
When in pregnancy did the study plan to conduct the ultrasound(s)?	__________________________<br>(List all trimesters or weeks of gestation the study had planned to administer ultrasound)
Fetal ultrasound machine model(s)	__________________________
Where were fetal ultrasounds performed?<br><br>Select all that apply	☐ Same health care facility where study is taking place<br>☐ Study-specific site (not a health care facility)<br>☐ Private/external site
Training received by ultrasound technician	__________________________
Quality control for ultrasound evaluation of abnormalities/head circumference	__________________________
Criteria for considering a fetal ultrasound as "abnormal"	__________________________
Was fetal MRI performed?	◯ Yes<br>◯ No
How many fetal MRIs did the study plan to conduct for each pregnant woman?	__________________________
When in pregnancy did the study plan to conduct the fetal MRI(s)?	__________________________<br>(List all trimesters or weeks of gestation the study had planned to administer MRIs)
Fetal MRI machine model(s)	__________________________
Where were fetal MRIs performed?<br><br>Select all that apply	☐ Same health care facility where study is taking place<br>☐ Study-specific site (not a health care facility)<br>☐ Private/external site

Confidential

Training received by MRI technician

____

Quality control for MRI evaluation of abnormalities/head circumference

____

Criteria for considering a fetal MRI as "abnormal"

____

When was prenatal/fetal microcephaly diagnosed?

- ◯ Postnatally diagnosed (after birth) congenital microcephaly
- ◯ Prenatally diagnosed (in utero) congenital microcephaly
- ◯ Study did not measure prenatal/fetal microcephaly

Gestational age (in weeks) at which study first assessed prenatal microcephaly

____

Reference standards used to calculate fetal head circumference Z score

Select all that apply

- ☐ Intergrowth
- ☐ Ministry of Health standards
- ☐ WHO or PAHO standard
- ☐ Local reference standard
- ☐ Consensus/study researchers
- ☐ Other

Specify other reference standards used to calculate fetal head circumference Z score

____

How was prenatal microcephaly diagnosed?

Select all that apply

- ☐ Presence vs absence
- ☐ By grades (normocephaly vs microcephaly vs severe microcephaly)
- ☐ By percentiles
- ☐ By Z-score
- ☐ By diagnostic certainty criteria
- ☐ Other

Specify other type of diagnosis of prenatal microcephaly

____

How was prenatal microcephaly defined?

- ◯ >2 SD below the mean
- ◯ < 3 SD below the mean
- ◯ 3 SD below the mean
- ◯ >3 SD below the mean
- ◯ below the 5th percentile
- ◯ below the 10th percentile
- ◯ Other

Specify other definition of prenatal microcephaly

____

Confidential

How was prenatal microcephaly defined?	◯ Level 1A of diagnostic certainty<br>◯ Level 1B of diagnostic certainty<br>◯ Level 2 of diagnostic certainty<br>◯ Level 3A of diagnostic certainty<br>◯ Level 3B of diagnostic certainty<br>◯ Level 4 of diagnostic certainty<br>◯ Other
How else was prenatal microcephaly defined?	______________________________

Ascertainment of infant ZIKV infection - Immunoassay

Was infant ZIKV infection measured?

- ○ Yes
- ○ No

Which criteria did you use to infer infant ZIKV status?

Select all that apply

- ☐ infant clinical criteria
- ☐ infant laboratory criteria
- ☐ infant imaging criteria
- ☐ maternal clinical criteria
- ☐ maternal laboratory criteria
- ☐ other criteria

In what other way was infant ZIKV infection assessed?

____

When was infant ZIKV infection measured?

Select all that apply

- ☐ Immediately after birth
- ☐ 1 month after birth
- ☐ 6 months after birth
- ☐ Other

At what other time was infant ZIKV infection measured?

____

Type of laboratory test performed to ascertain infant ZIKV infection

Select all that apply

- ☐ molecular diagnosis (e.g. RT-PCR, other NAAT)
- ☐ immunoassay (e.g. ELISA, IFA, PRNT, IHC, microneutralization)
- ☐ rapid diagnostic test (RDT)
- ☐ Other

Specify other test performed to ascertain infant ZIKV infection

____

Did you use different assays, different brands of the same assay, or different versions of the same brand of assay at different study sites to ascertain infant ZIKV status or over time within the study?

Select all that apply

- ☐ Yes, different versions of the same brand of assay over time
- ☐ Yes, different brands of the same type of assay over time
- ☐ Yes, different types of assays over time
- ☐ Yes, different versions of the same brand of assay at different sites
- ☐ Yes, different brands of the same type of assay at different sites
- ☐ Yes, different types of assays at different sites
- ☐ No, same tests applied throughout the project

Which different assay(s) did you use over time or across sites?

____

Which different brands of the assay did you use over time or across sites?

____

Which different versions of the brand of assay did you use over time or across sites?

____

Confidential

ZIKV immunoassay analyte to ascertain infant infection<br><br>Select all that apply	☐ IgM<br>☐ IgG<br>☐ IgA<br>☐ neutralizing antibodies<br>☐ Other
Other ZIKV immunoassay analyte to ascertain infant infection	__________________________________
ZIKV immunoassay type to ascertain infant infection<br><br>Select all that apply	☐ ELISA<br>☐ IFA<br>☐ MIA<br>☐ PRNT<br>☐ Other
Specify other type(s) or ZIKV immunoassay(s) performed to ascertain infant infection	__________________________________

Carabali M, *et al. BMJ Open* 2022; 12:e064362. doi: 10.1136/bmjopen-2022-064362

ZIKV immunoassay brand name (to ascertain infant infection)<br><br>Select all that apply	☐ In house-developed assay<br>☐ Zika MAC-ELISA (CDC)<br>☐ ZIKV Detect 2.0 IgM Capture ELISA Kit (InBios International Inc)<br>☐ Zika IgM ELISA (EUROIMMUN)<br>☐ Zika IgM + IgA ELISA (EUROIMMUN)<br>☐ Zika IgG ELISA (EUROIMMUN)<br>☐ ZIKV IgM IFA (EUROIMMUN)<br>☐ ZIKV IgG IFA (EUROIMMUN)<br>☐ Arbovirus Fever Mosaic 2 IgG + IgM IFA ZIKV-DENV-CHIKV (EUROIMMUN)<br>☐ ADVIA Centaur Zika Test (Siemens Healthcare Diagnostics)<br>☐ DPP Zika IgM Assay System (Chembio Diagnostic Systems Inc)<br>☐ DPP Zika IgM/IgG System (Chembio Diagnostic Systems Inc)<br>☐ DPP Zika/Chikungunya/Dengue IgM/IgG Combination Assay (Chembio Diagnostic Systems Inc)<br>☐ LIAISON XL Zika Capture IgM II assay (DiaSorin S.p.A.)<br>☐ STANDARD Q Zika IgM/IgG test (SD Biosensor)<br>☐ STANDARD E Zika IgM ELISA (SD Biosensor)<br>☐ STANDARD Q ZIKV/DENV/CHIKV Fast Quad (SD Biosensor)<br>☐ STANDARD Q ZIKV/DENV/CHIKV/YFV IgM Quad (SD Biosensor)<br>☐ Tell Me Fast Zika IgG/IgM Antibody Rapid Test (Biocan Diagnostics Inc)<br>☐ Tell Me Fast zika/dengue/chikungunya combination rapid tests (Biocan Diagnostics Inc)<br>☐ ZIKV-DENV-CHIKV IFA IgG (Vircell)<br>☐ ZIKV-DENV-CHIKV IFA IgM (Vircell)<br>☐ Human Anti-Zika ELISA Kits (Alpha Diagnostic International)<br>☐ anti-Zika virus IgM ELISA kit (µ-capture) (AbCam)<br>☐ anti-Zika virus IgG ELISA kit (AbCam)<br>☐ Zika Virus IgM ELISA Assay Kit (Eagle Biosciences)<br>☐ Zika Virus IgG ELISA Assay Kit (Eagle Biosciences)<br>☐ Human Anti-Zika Virus IgG ELISA Kit (R&D Systems)<br>☐ ZIKV IgG ELISA kit (DIA.PRO Diagnostic Bioprobes Srl)<br>☐ ZIKV IgM ELISA kit (DIA.PRO Diagnostic Bioprobes Srl)<br>☐ NovaLisa Zika Virus IgM µ-capture ELISA (NovaTec Immunodiagnostica GmbH)<br>☐ recomLine Tropical Fever IgG (MIKROGEN Diagnostik)<br>☐ recomLine Tropical Fever IgM (MIKROGEN Diagnostik)<br>☐ Other
Which other immunoassays were used to diagnose infant ZIKV infection?	__________________________________
What type of assay is the in house assay? (to ascertain infant ZIKV infection)<br><br>Select all that apply	☐ ELISA<br>☐ IFA<br>☐ MIA<br>☐ PRNT<br>☐ Other
What type of assay is the in house assay? (to ascertain infant ZIKV infection)	__________________________________

What is the analyte measured by the in house assay? (to ascertain infant ZIKV infection)<br><br>Select all that apply	☐ IgM<br>☐ IgG<br>☐ IgA
What is the antigenic target for the in house assay (to ascertain infant ZIKV infection)<br><br>Select all that apply	☐ NS1<br>☐ envelope protein<br>☐ NS5<br>☐ Other
What is the antigenic target for the in house assay (to ascertain infant ZIKV infection)	__________________________________
For the in house ZIKV immunoassay to ascertain infant infection, describe the testing procedure, including: incubation period, timing, type of cells, buffers, method to detect binding antibodies (e.g., optical density or secondary antibody detection), and methods to stop the enzymatic reaction	__________________________________
Sample used for ZIKV immunoassay (to ascertain infant infection)<br><br>Select all that apply	☐ Serum/plasma<br>☐ Whole blood<br>☐ cerebrospinal fuid (CSF)<br>☐ chord blood<br>☐ Other
Specify other sample used for ZIKV immunoassay (to ascertain infant infection)	____________________________
ZIKV ELISA tested in paired/subsequent samples	◯ Yes<br>◯ No
At what temperature was the ZIKV ELISA test stored? (degrees Celsius)	____________________________
At what temperature(s) was/were the ZIKV ELISA sample(s) stored? (degrees Celsius)	__________________________________<br>(Include sample type for each temperature)
In what volume(s) was/were the ZIKV ELISA sample aliquot(s) stored? (mL)	__________________________________<br>(Include sample type with each volume)
Did your study deviate from the directions provided in the ZIKV ELISA commercial kit?	◯ Yes<br>◯ No<br>◯ Study did not use a commercial ZIKV ELISA kit
Describe any deviations from the procedure recommended by the commercial ELISA kit, including: incubation period, timing, type of cells, buffers, method to detect bounding antibodies, and methods to stop the enzymatic reaction	__________________________________

Describe any additional conditions for ZIKV ELISA test administration for ascertining infant infection (e.g. paired samples within 21 days of fever onset; count with more than 2ul of serum from the febrile phase, etc.)	__________________________________
Where was the ZIKV ELISA test performed for ascertining infant infection?<br><br>Select all that apply	☐ Lab at the same health care facility where study is conducted<br>☐ Study-specific lab<br>☐ Ministry of Health reference lab<br>☐ Private external lab<br>☐ Other
Specify other lab where ZIKV ELISA was performed	___________________________

Ascertainment of infant ZIKV infection - ELISA

ZIKV ELISA cut-off point for ascertining infant infection	__________________________
Was a positive control from the outbreak included for the ZIKV ELISAs for ascertining infant infection?	◯ Yes<br>◯ No
Was a negative control included with every run of ZIKV ELISAs for ascertining infant infection?	◯ Yes<br>◯ No
Definition of POSITIVE ZIKV ELISA for ascertining infant infection	________________________________
Definition of NEGATIVE ZIKV ELISA for ascertining infant infection	________________________________
Definition of INCONCLUSIVE ZIKV ELISA for ascertining infant infection	________________________________
Source of definition used to interpret ZIKV immunoassay test results for ascertining infant infection<br><br>Select all that apply	☐ Manufacturer<br>☐ Ministry of Health reference lab<br>☐ WHO (global or local office e.g: PAHO)<br>☐ US-CDC<br>☐ Consensus/study researchers<br>☐ Other
Specify other source of definition used to interpret ZIKV immunoassay test results	__________________________
How many ZIKV ELISAs did the study plan to conduct in each infant?	__________________________
Would your study be able to provide the P/N ratio, titer, or other quantitative readout for ZIKV ELISAs to inform the measurement error models?	◯ Yes<br>◯ No

Confidential

Ascertainment of infant ZIKV infection - IFA

ZIKV IFA cut-off value for ascertining infant infection	__________________
Was a positive control from the outbreak included for the ZIKV IFA for ascertining infant infection?	◯ Yes<br>◯ No
Was a negative control included with every run of ZIKV IFA for ascertining infant infection?	◯ Yes<br>◯ No
Definition of POSITIVE ZIKV IFA for ascertining infant infection	__________________
Definition of NEGATIVE ZIKV IFA for ascertining infant infection	__________________
Definition of INCONCLUSIVE ZIKV IFA for ascertining infant infection	__________________
Source of definition for ZIKV IFA test results for ascertining infant infection<br><br>Select all that apply	☐ Manufacturer<br>☐ Ministry of Health reference lab<br>☐ WHO (global or local office e.g: PAHO)<br>☐ Consensus/study researchers<br>☐ Other
Specify other source of definition for ZIKV IFA test results for ascertining infant infection	__________________
Specify any additional inclusion or exclusion criteria for determining which samples to test for ZIKV using IFA for ascertining infant infection	__________________
Will your study be able to provide the titer or optical density for ZIKV IFAs to inform the measurement error model?	◯ Yes<br>◯ No

Confidential

Ascertainment of infant ZIKV infection - MIA

ZIKV MIA cut-off value for ascertining infant infection	__________________
Was a positive control from the outbreak included for the ZIKV MIAs for ascertining infant infection?	◯ Yes<br>◯ No
Was a negative control included with every run of ZIKV MIAs for ascertining infant infection?	◯ Yes<br>◯ No
Definition of POSITIVE ZIKV MIA for ascertining infant infection	__________________
Definition of NEGATIVE ZIKV MIA for ascertining infant infection	__________________
Definition of INCONCLUSIVE ZIKV MIA for ascertining infant infection	__________________
Source of definition for MIA ZIKV test results for ascertining infant infection<br><br>Select all that apply	☐ Manufacturer<br>☐ Ministry of Health reference lab<br>☐ WHO (global or local office e.g: PAHO)<br>☐ Consensus/study researchers<br>☐ Other
Specify other source of definition for ZIKV MIA test results	__________________
Specify any additional inclusion or exclusion criteria for determining which samples to test for ZIKV using MIA for ascertining infant infection	__________________
Will your study be able to provide the titer for ZIKV MIAs to inform the measurement error model?	◯ Yes<br>◯ No

Ascertainment of infant ZIKV infection - Immunoassay - PRNT

Did you use the CDC protocol for the PRNT for ascertining infant infection?
◯ Yes
◯ No

Describe the other protocol used for the PRNT
____

End-point PRNT titer for infants
◯ PRNT50
◯ PRNT90
◯ Other

Which other end-point did you use for the PRNT titer for infant infection?
____

Definition of a positive PRNT titer for infants
◯ PRNT>10
◯ Other

Other definition of a positive PRNT titer for infants
____

Source of the definition used to interpret ZIKV PRNT test results for infants

Select all that apply
☐ Manufacturer
☐ Ministry of Health reference lab
☐ WHO (global or local office e.g: PAHO)
☐ US-CDC
☐ Consensus/study researchers
☐ Other

Specify other source of definition used to interpret ZIKV PRNT test results
____

Definition of POSITIVE ZIKV PRNT for infants
____

Definition of NEGATIVE ZIKV PRNT for infants
____

Definition of INCONCLUSIVE PRNT (i.e. specific flavivirus could not be determined) for infants
____

Specify any additional inclusion or exclusion criteria for determining which infant samples to test for ZIKV using PRNT
____

Where was the ZIKV PRNT test performed for samples from infants?

Select all that apply
☐ Lab at the same health care facility where study is conducted
☐ Study-specific lab
☐ Ministry of Health reference lab
☐ Private external lab
☐ Other

Specify other lab where ZIKV PRNT test was performed for samples from infants
____

How many ZIKV PRNTs were conducted with samples from infants?	________________________________
Will your study be able to provide the titer for ZIKV PRNTs from infants to inform the measurement error model?	◯ Yes<br>◯ No

Carabali M, *et al*. *BMJ Open* 2022; 12:e064362. doi: 10.1136/bmjopen-2022-064362

Confidential

Ascertainment of infant ZIKV infection - Molecular assay

Type of ZIKV molecular tests performed in infants<br><br>Select all that apply	☐ conventional PCR<br>☐ rRT-PCR<br>☐ qRT-PCR<br>☐ Other NAAT
According to the study protocol, when were the ZIKV samples to be collected for molecular testing?<br><br>Select all that apply	☐ within 24 hours after birth<br>☐ within first week after birth<br>☐ Other
When were the ZIKV molecular tests (RT-PCR, other NAAT) conducted in infants?	______________________________
Sample used for ZIKV RT-RCR/PCR in infants<br><br>Select all that apply	☐ Serum/plasma<br>☐ Whole blood<br>☐ Urine<br>☐ Saliva<br>☐ Breast milk<br>☐ Umbilical cord tissue<br>☐ cerebrospinal fuid (CSF)<br>☐ chord blood<br>☐ Other
Specify other type of sample used for ZIKV RT-PCR/PCR in infants	________________________

ZIKV RT-PCR/PCR brand name used for testing infants<br><br>Select all that apply	☐ In house technique<br>☐ Zika Virus RNA Qualitative Real-Time RT-PCR (Quest Diagnostics)<br>☐ RealStar Zika Virus RT-PCR Kit U.S. (altona Diagnostics GmbH)<br>☐ RealStar Zika Virus RT-PCR Kit 1.0 (altona Diagnostics GmbH)<br>☐ Aptima Zika Virus assay (Hologic Inc)<br>☐ Zika Virus Real-time RT-PCR (Viracor Eurofins)<br>☐ VERSANT Zika RNA 1.0 Assay (kPCR) (Siemens Healthcare Diagnostics Inc)<br>☐ xMAP MultiFLEX Zika RNA Assay (Luminex Corporation)<br>☐ Sentosa SA ZIKV RT-PCR Test (Vela Diagnostics Inc)<br>☐ Zika Virus Detection by RT-PCR (ARUP Laboratories)<br>☐ Abbott RealTime Zika (Abbott Molecular Inc)<br>☐ Zika ELITe MGB Kit U.S. (ELITechGroup Molecular Diagnostics)<br>☐ Gene-RADAR Zika Virus Test (Nanobiosym Diagnostics Inc)<br>☐ TaqPath Zika Virus Kit (Thermo Fisher Scientific)<br>☐ CII-ArboViroPlex rRT-PCR assay (Columbia University)<br>☐ Trioplex Real-Time RT-PCR Assay (CDC)<br>☐ Liferiver Zika Virus (ZIKV) Real Time RT-PCR Kit (Shanghai ZJ Bio-Tech Co Ltd)<br>☐ careGENE Zika Virus RT-PCR Kit (WELLS BIO Inc)<br>☐ Zika Virus - Single Check (Genekam Biotechnology AG)<br>☐ FTD Zika virus (Fast Track Diagnostics)<br>☐ VIASURE Zika Virus Real Time PCR Detection Kit (Certest Biotec)<br>☐ Zika Virus Real-TM (Sacace Biotechnologies)<br>☐ Logix Smart Zika Test Kit (Co-Diagnostics Inc)<br>☐ Genesig Kits for ZIKV (Primerdesign Ltd)<br>☐ Genesig Multiplex Kit for Dengue, Chikungunya and Zika Virus (Primerdesign Ltd)<br>☐ AccuPower ZIKV (DENV, CHIKV) Multiplex Real-Time RT-PCR Kit (Bioneer)<br>☐ VIASURE Zika, Dengue & Chikungunya Real Time PCR Detection Kit (Certest Biotec)<br>☐ FTD Zika/Dengue/Chik (Fast-Track Diagnostics)<br>☐ DiaPlexQ ZCD (ZIKV, CHIKV, DENV) Detection Kit (SolGent Co Ltd)<br>☐ TaqMan Zika Virus Triplex Kit (Thermo Fisher Scientific)<br>☐ GenoAmp Trioplex Real-Time RT-PCR Zika/Den/Chiku (Medical Innovation Ventures)<br>☐ Other
Describe procedure for in house ZIKV RT-PCR/PCR for ascertining infant infection	__________________________________
Specify other ZIKV RT-PCR/PCR brand name	__________________________________
Did the study obtain a measure of ZIKV viral load in infants?	◯ Yes<br>◯ No
ZIKV PCR/RT-PCR cut-off point in infants	____________________________

Confidential

Was a negative control included with every run of ZIKV RT-PCR/PCRs?	◯ Yes<br>◯ No
Definition of POSITIVE ZIKV RT-PCR/PCR in infants	__________________________________
Definition of NEGATIVE ZIKV RT-PCR/PCR in infants	__________________________________
Definition of INCONCLUSIVE ZIKV RT-PCR/PCR in infants	__________________________________
Source of definition used to interpret ZIKV RT-PCR/PCR test results<br><br>Select all that apply	☐ Manufacturer<br>☐ Ministry of Health reference lab<br>☐ WHO (global or local office e.g: PAHO)<br>☐ US-CDC<br>☐ Consensus/study researchers<br>☐ Other
Specify other source of definition used to interpret ZIKV RT-PCR/PCR test results	___________________________
Where was the ZIKV RT-PCR/PCR test performed for samples from infants?<br><br>Select all that apply	☐ Lab at the same health care facility where study is conducted<br>☐ Study-specific lab<br>☐ Ministry of Health reference lab<br>☐ Private external lab<br>☐ Other
Specify other lab where ZIKV RT-PCR/PCR test was performed for samples from infants	___________________________
Specify any additional inclusion or exclusion criteria for determining which samples from infants to test for ZIKV using RT-PCR/PCR	__________________________________
How many ZIKV PCRs were conducted with infants?	___________________________
Would your study be able to provide a Ct value for infant ZIKV PCRs to inform the measurement error models?	◯ Yes<br>◯ No

Confidential

Changes in diagnosis of infant ZIKV infection over time

Which clinical criteria did you apply initially to diagnose infant infection? (Clinical Definition 1)

____

Did your clinical criteria for infant infection change over time?

- ◯ No
- ◯ Yes - changed 1x
- ◯ Yes - changed more than 1x

If your clinical criteria changed over time, which criteria did you apply subsequently to assess infant infection? (Clinical Definition 2)

____

When did you begin using the revised clinical criteria for assessing infant infection? (Clinical Definition 2)

____

What was the third set of criteria you applied to assess infant infection? (Clinical Definition 3)

____

When did you begin using the third set of clinical criteria? (Clinical Definition 3)

____

Which imaging criteria did you apply initially to diagnose infant infection? (Clinical Definition 1)

____

Did your imaging criteria for infant infection change over time?

- ◯ No
- ◯ Yes - changed 1x
- ◯ Yes - changed more than 1x

If your imaging criteria changed over time, which criteria did you apply subsequently to assess infant infection? (Clinical Definition 2)

____

When did you begin using the revised imaging criteria for assessing infant infection? (Clinical Definition 2)

____

What was the third set of imaging criteria you applied to assess infant infection? (Clinical Definition 3)

____

When did you begin using the third set of imaging criteria? (Clinical Definition 3)

____

What laboratory criteria was used initially to diagnose infant ZIKV infection? (Laboratory Definition 1)

____

Did your laboratory criteria for diagnosing infant infection change over time?

- ◯ No
- ◯ Yes - changed 1x
- ◯ Yes - changed more than 1x

Confidential

Which laboratory criteria did you apply subsequently? (Laboratory Definition 2)	______________________________
When did you begin using the revised lab criteria for infant infection? (Laboratory Definition 2)	________________________
What was the third set of laboratory criteria you applied for diagnosing infant infection? (Laboratory Definition 3)	______________________________
When did you begin using the third set of lab criteria for infant infection? (Laboratory Definition 3)	________________________
Were infant samples retested using the new laboratory criteria?	______________________________

ZIKV testing algorithm & test interpretation - infant ZIKV infection

Describe the testing algorithm for defining a confirmed ZIKV+ test in an infant	______________________
Describe the testing algorithm for defining a confirmed ZIKV- test in an infant	______________________
What tests were used to confirm ZIKV infection in an infant?<br><br>Select all that apply	☐ ZIKV IgM - 1 sample<br>☐ ZIKV IgM - paired samples (seroconversion or increased titers)<br>☐ ZIKV IgM (1 sample) + IgG<br>☐ ZIKV IgM (paired samples) + IgG<br>☐ RT-PCR or other NAAT<br>☐ PRNT > 10<br>☐ Other
Specify other test or combination of tests used to confirm ZIKV infection in an infant	______________________
Considering any relevant clinical and laboratory criteria, what was the study's definition of a CONFIRMED infant ZIKV Infection	______________________
Considering any relevant clinical and laboratory criteria, what was the study's definition of a PROBABLE infant ZIKV Infection	______________________
Considering any relevant clinical and laboratory criteria, what was the study's definition of a NEGATIVE/UNLIKELY infant ZIKV Infection	______________________
Source of ZIKV test result categorization<br><br>Select all that apply	☐ Manufacturer<br>☐ Ministry of Health reference lab<br>☐ WHO (global or local office e.g: PAHO)<br>☐ Consensus/study researchers<br>☐ Other
Specify other source of ZIKV test result categorization	______________________

ZIKV Diagnostics external quality assessment (EQA)

Did your study conduct ZIKV test-related external quality assessment (EQA)?	◯ Yes<br>◯ No
Where was the EQA conducted?	◯ Lab at the same health care facility where study is conducted<br>◯ Study-specific lab<br>◯ Ministry of Health reference lab<br>◯ Private external lab<br>◯ Other
Where else was the EQA conducted?	__________________________
Which group conducted the EQA?	__________________________
Which tests were evaluated in the EQA?<br><br>Select all that apply	☐ ZIKV ELISA<br>☐ ZIKV PCR/RT-PCR<br>☐ Other
Which other tests were evaluated in the EQA?	__________________________
Which assay or assays was/were used as a comparator for the ELISA EQA?	__________________________
Sensitivity of ZIKV ELISA test	__________________________<br>(Provide as percentage)
Specificity of ZIKV ELISA test	__________________________<br>(Provide as percentage)
PPV of the ZIKV ELISA test	__________________________<br>(Provide as percentage)
ZIKV prevalence used to estimate the PPV	__________________________
Sample (N) for ELISA EQA	__________________________
How many ELISAs, either IgG or IgM, were included in the EQA?	__________________________
Which assay or assays was/were used as a comparator for the PCR/RT-PCR EQA?	__________________________

Sensitivity of ZIKV PCR/RT-PCR test	______________________ (Provide as percentage)
Specificity of ZIKV PCR/RT-PCR test	______________________ (Provide as percentage)
PPV of the ZIKV PCR/RT-PCR test	______________________ (Provide as percentage)
ZIKV prevalence used to estimate the PPV	______________________
How many molecular tests (PCR/RT-PCR) were included in the EQA?	______________________
Describe the procedure used to resolve inconsistencies in the EQA	______________________
Was a positive control from the outbreak included in the EQA?	◯ Yes<br>◯ No
Was a negative control from the outbreak included in the EQA?	◯ Yes<br>◯ No
Please describe any additional details of the EQA	______________________
List any publications related to the EQA described above	______________________

Confidential

Perinatal Outcomes

How did the study measure gestational age at birth?	◯ last menstrual period (LMP)<br>◯ ultrasound<br>◯ LMP if ultrasound unavailable, otherwise ultrasound<br>◯ Other<br>◯ Study did not measure gestational age at birth
Describe other means of ascertaining gestational age at birth	__________________________________
Did the study include a newborn maturity assessment?<br><br>Select all that apply	☐ Ballard<br>☐ Dubowitz<br>☐ Capurro<br>☐ Other<br>☐ No, study did not include newborn maturity assessment
Specify other maturity assessment of newborn	__________________________
How did the study determine preterm birth?<br><br>Select all that apply	☐ Gestational age - from ultrasound<br>☐ Gestational age - from LMP<br>☐ Gestational age from LMP if ultrasound not available, otherwise from ultrasound<br>☐ Maturity assessment<br>☐ Other<br>☐ Study did not assess preterm birth
Specify other determination of preterm birth	__________________________
How did the study define preterm birth?	__________________________________<br>((e.g. extremely preterm (< 28 weeks); very preterm (28-32 weeks), moderate to late preterm (32-37 weeks)))
How did the study determine termination of pregnancy (TOP)/induced abortion?	◯ Self report<br>◯ Medical records<br>◯ Other<br>◯ Study did not measure TOP/induced abortion
Specify other means of determining termination of pregnancy/induced abortion	__________________________________
Provide wording of question related to TOP/induced abortion for self report or provide directions for extracting TOP-related information from medical chart	__________________________________
How did the study define miscarriage?	◯ Pregnancy loss < 20 weeks regardless of weight<br>◯ Pregnancy loss < 20 weeks or weight < 500 gr<br>◯ Involuntary loss < 20 weeks or voluntary loss between 10-20 weeks (Cuban definition)<br>◯ Other<br>◯ Study did not measure miscarriage

Specify other definition of miscarriage	__________________
How did the study define fetal loss or fetal death?	◯ Any loss < 20 weeks regardless of weight<br>◯ Any loss < 22 weeks or weight < 500 gr<br>◯ Early (20-27 weeks) and late (>28 weeks) fetal loss regardless of weight<br>◯ Other<br>◯ Study did not measure fetal loss
Specify other definition of fetal loss or fetal death	__________________
Did the study document placental insufficiency as an outcome?	◯ Yes<br>◯ No
Definition of placental insufficiency	__________________
Did the study document intrauterine growth restriction (IGR) as an outcome?	◯ Yes<br>◯ No
Definition of intrauterine growth restriction	__________________
Did the study document low birth weight (LBW) as an outcome?	◯ Yes<br>◯ No
How did the study define low birthweight?	__________________
How did the study define very low birthweight?	__________________
How did the study define extremely low birthweight?	__________________

Postnatal ZIKV-related imaging & microcephaly

Were postnatal cranial ultrasounds performed?	◯ Yes<br>◯ No
How many postnatal cranial ultrasounds did the study plan to conduct with each infant/child?	__________________________
Age (months) when the study planned to conduct the postnatal cranial ultrasounds	__________________________<br>(e.g., 1, 3, 6 months)
Postnatal cranial ultrasound machine models (list all that apply)	__________________________<br>(Write "same as above" if ultrasound machine models are same as those described in prenatal ultrasound section.)
Place where postnatal cranial ultrasound(s) were performed<br><br>Select all that apply	☐ Same health care facility where study is taking place<br>☐ Study-specific site (not a health care facility)<br>☐ Private/external site<br>☐ Other
Other place that the postnatal cranial ultrasound(s) were performed	__________________________
Training received by ultrasound technician performing postnatal cranial ultrasounds	__________________________<br>(Write "same as above" if technician training same as those described in prenatal ultrasound section.)
Quality control procedures for ultrasound evaluation of abnormalities/head circumference	__________________________<br>(Write "same as above" if quality control measures same as those described in prenatal ultrasound section.)
What other types of postnatal ultrasound did the study conduct?<br><br>Select all that apply	☐ Thoracic<br>☐ Abdominal<br>☐ Pelvic<br>☐ Osteo muscular<br>☐ Other<br>☐ No other types of postnatal ultrasound
Specify other type of additional postnatal ultrasound	__________________________
Did the study perform postnatal brain/head/neck MRIs?	◯ Yes<br>◯ No
How many postnatal brain/head/neck MRIs did the study plan to conduct with each infant/child?	__________________________

What other types of postnatal MRI did the study conduct?<br><br>Select all that apply	☐ Thoracic<br>☐ Abdominal<br>☐ Pelvic<br>☐ Osteo muscular<br>☐ Other<br>☐ No other types of postnatal MRI
Specify other type of additional postnatal MRI	______________________________
Age (months) when the study planned to conduct any postnatal MRI	______________________________<br>(e.g. 1, 3, 6, 9 months)
Describe any conditions for application of any postnatal MRI	______________________________
Place where postnatal MRI was performed	◯ Same health care facility where study is taking place<br>◯ Study-specific site (not a health care facility)<br>◯ Private/external site
Training received by postnatal MRI technician	______________________________<br>(Write "same as above" if technician training same as that described in prenatal MRI section.)
Quality control for evaluation of postnatal MRI	______________________________<br>(Write "same as above" if quality control measures same as those described in prenatal MRI section.)
Was postnatal microcephaly an outcome in this study?	◯ Yes<br>◯ No
At what age(s) (in months) was postnatal microcephaly assessed?	______________________________<br>(e.g., 1, 3, 6 months)
How was postnatal microcephaly diagnosed?<br><br>Select all that apply	☐ Presence vs absence<br>☐ By grades (normocephaly vs microcephaly vs severe microcephaly)<br>☐ By percentiles<br>☐ By Z-score<br>☐ By diagnostic certainty criteria<br>☐ Other
Specify other postnatal microcephaly diagnosis type	______________________________

How did the study define postnatal microcephaly?	◯ >2 SD below the mean<br>◯ < 3 SD below the mean<br>◯ 3 SD below the mean<br>◯ >3 SD below the mean<br>◯ below the 5th percentile<br>◯ below the 10th percentile<br>◯ Other
Specify other definition of postnatal microcephaly	__________________________________
How did the study define postnatal microcephaly?	◯ Level 1 of diagnostic certainty<br>◯ Level 2A of diagnostic certainty<br>◯ Level 2B of diagnostic certainty<br>◯ Level 3A of diagnostic certainty<br>◯ Level 3B of diagnostic certainty<br>◯ Level 4 of diagnostic certainty<br>◯ Other
Specify other definition of postnatal microcephaly	___________________________
Did the study measure infant head circumference	◯ Yes<br>◯ No
Device used to measure infant head circumference<br><br>Select all that apply	☐ Flexible-non-stretchable measuring tape-marked by 0.1 cm increments<br>☐ Metal tape<br>☐ Paper tape<br>☐ Other<br>☐ Unknown
Specify other device used to measure infant head circumference	__________________________________
Reference standards used to calculate infant head circumference Z score<br><br>Select all that apply	☐ Intergrowth<br>☐ Ministry of Health standards<br>☐ WHO or PAHO standard<br>☐ Local reference standard<br>☐ Consensus/study researchers<br>☐ Other
Other reference standards used to calculate infant head circumference Z score	___________________________
Was epilepsy screening performed?	◯ Yes<br>◯ No
How did the study define epilepsy?	__________________________________
How many times did the study plan to screen the infant or child for epilepsy?	___________________________

Confidential

List ages (in months) at which the study planned to screen for epilepsy	__________________
Was postnatal electroencephalography (EEG) performed?	◯ Yes<br>◯ No
How many EEGs did the study plan to conduct with each infant or child?	__________________
Were the EEGs reviewed for abnormalities by epileptologists who were blinded to the ZIKV status of the mother?	◯ Yes<br>◯ No

Confidential

Ascertainment of congenital Zika syndrome (CZS)

Did the study measure congenital Zika syndrome (CZS)?	◯ Yes<br>◯ No
When did the study assess CZS?<br><br>Select all that apply	☐ Antenatal<br>☐ Peri/intrapartum<br>☐ Postpartum
When, during pregnancy, did the study assess antenatal CZS?<br><br>Select all that apply	☐ 1st trimester<br>☐ 2nd trimester<br>☐ 3rd trimester
Time of CZS assessment in hours after delivery for peri/intrapartum assessment	________________________<br>(Please list all times, separated by a comma)
Time of CZS assessment in days after delivery for postpartum diagnosis of CZS	________________________<br>(Please list all times, separated by a comma)
Which criteria were used to assess CZS?<br><br>Select all that apply	☐ maternal clinical<br>☐ maternal laboratory<br>☐ placental laboratory<br>☐ fetal clinical<br>☐ fetal laboratory<br>☐ fetal neuroimaging<br>☐ infant clinical<br>☐ infant laboratory<br>☐ infant neuroimaging<br>☐ developmental<br>☐ other
Which other criteria were used to assess CZS?	______________________________
Infant or fetal neurologic alterations used to assess CZS<br><br>Select all that apply	☐ Epilepsy<br>☐ Seizures<br>☐ Dysphagia<br>☐ Hypertonia<br>☐ Hypotonia<br>☐ Hyperreflexia<br>☐ Hyporeflexia<br>☐ Other neurologic abnormality
Which other infant or fetal neurological criteria were used to assess CZS?	______________________________
Infant or fetal structural abnormalities used to assess CZS<br><br>Select all that apply	☐ Cranial shape malformation<br>☐ Microcephaly<br>☐ Abnormalities of intracranial anatomy<br>☐ Musculoskeletal defects<br>☐ Eye defects<br>☐ Hearing (ENT) defects<br>☐ Other structural abnormality

Confidential

Which other infant or fetal structural criteria were considered in assessing CZS?	__________________________________
What type of microcephaly diagnosis was used to diagnose CZS?<br><br>Select all that apply	☐ Antenatal microcephaly<br>☐ Postnatal microcephaly<br>☐ Both antenatal and postnatal microcephaly<br>☐ Other
What other type of microcephaly diagnosis was used to diagnose CZS?	__________________________________
Which intracranial abnormalities were considered in assessing CZS?<br><br>Select all that apply	☐ Diffuse, primarily subcortical calcifications<br>☐ Increased fluid spaces - ventricular and extra-axial<br>☐ Marked cortical thinning with abnormal gyral patterns<br>☐ Hypoplasia or absence of corpus callosum<br>☐ Anencephaly<br>☐ Holoprosencephaly<br>☐ Decreased myelination<br>☐ Cerebellar or cerebellar vermis hypoplasia<br>☐ Calcifications in basal ganglion/brainstem<br>☐ Other
What other intracranial abnormalities were considered in assessing CZS?	__________________________________
Maternal clinical criteria used to assess CZS	◯ Fever only<br>◯ Rash only<br>◯ Fever + rash<br>◯ Fever or rash<br>◯ Fever + rash + joint pain<br>◯ Fever + rash + joint pain + conjunctivitis<br>◯ Other<br>◯ Fever + rash + other
Which other maternal clinical criterion or combination of criteria were used to assess CZS	__________________________________
Fetal clinical criteria used to assess CZS	◯ Fever only<br>◯ Rash only<br>◯ Fever + rash<br>◯ Fever or rash<br>◯ Fever + rash + joint pain<br>◯ Fever + rash + joint pain + conjunctivitis<br>◯ Other<br>◯ Fever + rash + other
Which other fetal clinical criterion or combination of criteria were used to assess CZS	__________________________________

Confidential

Infant clinical criteria used to assess CZS	◯ Fever only<br>◯ Rash only<br>◯ Fever + rash<br>◯ Fever or rash<br>◯ Fever + rash + joint pain<br>◯ Fever + rash + joint pain + conjunctivitis<br>◯ Other<br>◯ Fever + rash + other
Which other infant clinical criterion or combination of criteria were used to assess CZS	__________________________________
Maternal laboratory diagnostics used to assess CZS<br><br>Select all that apply	☐ Molecular diagnosis (e.g. RT-PCR, other NAAT)<br>☐ Antibody-based immunoassay (e.g. ELISA, IFA, PRNT, microneutralization)<br>☐ Antigen-based immunoassay (e.g. ELISA, IFA)<br>☐ Rapid diagnostic test (RDT)<br>☐ Other
Which other maternal laboratory diagnostics were used to assess CZS?	___________________________
Placental laboratory diagnostics used to assess CZS<br><br>Select all that apply	☐ Molecular diagnosis (e.g. RT-PCR, other NAAT)<br>☐ Antibody-based immunoassay (e.g. ELISA, IFA, PRNT, microneutralization)<br>☐ Antigen-based immunoassay (e.g. ELISA, IFA)<br>☐ Rapid diagnostic test (RDT)<br>☐ Other
Which other placental laboratory diagnostics were used to assess CZS?	___________________________
Fetal laboratory diagnostics used to assess CZS<br><br>Select all that apply	☐ Molecular diagnosis (e.g. RT-PCR, other NAAT)<br>☐ Antibody-based immunoassay (e.g. ELISA, IFA, PRNT, microneutralization)<br>☐ Antigen-based immunoassay (e.g. ELISA, IFA)<br>☐ Rapid diagnostic test (RDT)<br>☐ Other
Which other fetal laboratory diagnostics were used to assess CZS?	___________________________
Infant laboratory diagnostics used to assess CZS<br><br>Select all that apply	☐ Molecular diagnosis (e.g. RT-PCR, other NAAT)<br>☐ Antibody-based immunoassay (e.g. ELISA, IFA, PRNT, microneutralization)<br>☐ Antigen-based immunoassay (e.g. ELISA, IFA)<br>☐ Rapid diagnostic test (RDT)<br>☐ Other
Which other infant laboratory diagnostics used to assess CZS?	___________________________
Fetal neuroimaging used to assess CZS<br><br>Select all that apply	☐ Ultrasound<br>☐ MRI<br>☐ CT scan<br>☐ Other

Carabali M, *et al. BMJ Open* 2022; 12:e064362. doi: 10.1136/bmjopen-2022-064362

Confidential

Specify other neuroimaging used to assess CZS in fetus	__________________________________
Infant neuroimaging used to assess CZS<br><br>Select all that apply	☐ Ultrasound<br>☐ MRI<br>☐ CT scan<br>☐ Other
Specify other neuroimaging used to assess CZS in infant	__________________________________
Which types of developmental delays or impairments were included in the CZS assessment?<br><br>Select all that apply	☐ motor<br>☐ cognitive<br>☐ speech<br>☐ other
Which other types of developmental delays or impairments were included in the CZS assessment?	__________________________________
Did your study apply the following criteria for CZS phenotype?<br><br>Severe microcephaly with partially collapsed skull Thin cerebral cortices with subcortical calcifications Macular scarring and focal pigmentary retinal mottling Congenital contractures Marked early hypertonia and symptoms of extrapyramidal involvement	◯ Yes<br>◯ No
Reference standard used to define CZS	◯ WHO guidelines 2015<br>◯ WHO guidelines 2016<br>◯ WHO guidelines 2017<br>◯ Current country guidelines (Ministry of Health)<br>◯ Study definition<br>◯ Other
Specify other reference standards used to define CZS	___________________________
Specify study definition used to define CZS	__________________________________
Study's definition of a CONFIRMED case of CZS	__________________________________
Study's definition of a PROBABLE case of CZS	__________________________________
Study's definition of an UNLIKELY case of CZS	__________________________________

Carabali M, *et al. BMJ Open* 2022; 12:e064362. doi: 10.1136/bmjopen-2022-064362

Confidential

Genetic abnormalities

Did the study conduct prenatal genetic screening or testing?	◯ Yes<br>◯ No
Did the study conduct genetic testing on products of conception in cases of termination or fetal loss?	◯ Yes<br>◯ No
How was prenatal genetic screening conducted?<br><br>Select all that apply	☐ FTS (first trimester screening, standard or enhanced, maternal blood screen + ultrasound)<br>☐ only blood screening (maternal serum screening)<br>☐ only ultrasound findings (including Nuchal Translucency Ultrasound)<br>☐ cfDNA/NIPT (Non-invasive prenatal testing)<br>☐ Amniocentesis<br>☐ chorionic villus sampling<br>☐ Triple screen (with AFP)<br>☐ Quad screen (with AFP)<br>☐ other
What other type of genetic screening was conducted?	______________________________
What type of prenatal genetic testing was conducted?<br><br>Select all that apply	☐ cfDNA/NIPT<br>☐ FISH<br>☐ karyotype<br>☐ chromosomal microarray<br>☐ single gene<br>☐ gene panel<br>☐ other
What other type of genetic testing was conducted?	______________________________

Confidential

Maternal and infant C reactive protein

Did the study collect a measure of maternal C-reactive protein	◯ Yes<br>◯ No
Was C-reactive protein in the mother reported as a binary or continuous measure?	◯ binary<br>◯ continuous
What was the cut off for the binary measure of C-reactive protein in the mother?	__________________________
How many times did the study plan to measure C-reactive protein for each pregnant woman?	________________________________
Did the study collect a measure of infant C-reactive protein?	◯ Yes<br>◯ No
Was C-reactive protein in the infant reported as a binary or continuous measure?	◯ binary<br>◯ continuous
What was the cut off for the binary C-reactive protein measure in the infant?	__________________________
How many times did the study plan to measure C-reactive protein for each infant?	________________________________
At what ages did the study plan to measure C-reactive protein for each infant?	________________________________
How was C-reactive protein measured in the infant?	◯ Immunoturbidimetric<br>◯ Nephelometry

Ophthalmological exams

Did the study measure ocular abnormalities?	◯ Yes<br>◯ No
How did the study measure ocular abnormalities?<br><br>Select all that apply	☐ fundoscopic exam<br>☐ RetCam<br>☐ other
What other exam did the study use to measure ocular abnormalities?	__________________________
Type of eye abnormalities measured in the study<br><br>Select all that apply	☐ Macular findings: Focal pigmentary mottling<br>☐ Macular findings: Chorioretinal atrophy<br>☐ Optic nerve findings: Optic nerve hypoplasia<br>☐ Optic nerve findings: Optic nerve cupping<br>☐ Optic nerve findings: Optic nerve atrophy<br>☐ Other retinal lesions: subretinal hemorrhage<br>☐ Hypopigmentation<br>☐ Vascular tortuosity<br>☐ Abnormal termination of the vessels<br>☐ Focal vascular dilation<br>☐ Congenital glaucoma<br>☐ Iris colobomas<br>☐ Microphthalmia<br>☐ Cataracts<br>☐ Subluxation of the lens<br>☐ Intraocular calcification<br>☐ Other
Specify other eye abnormalities measured by the study	____________________
Were any of the following exams performed?<br><br>Select all that apply	☐ Cardiff acuity test<br>☐ Cardiff visual contrast test<br>☐ Teller test<br>☐ Other
Specify any other visual acuity tests performed by the study	____________________
At what age(s) did the study plan to measure ophthalmological function?	____________________
Reference standard used for evaluation of fundoscopic exam<br><br>Select all that apply	☐ Provided by manufacturer<br>☐ In-hospital standard<br>☐ Regional or state standard<br>☐ Federal standard<br>☐ WHO or local branch<br>☐ Other
Specify other basis for evaluation of fundoscopic exam	____________________

Confidential

Reference standard used for evaluation of RetCam exam<br><br>Select all that apply	☐ Provided by manufacturer<br>☐ In-hospital standard<br>☐ Regional or state standard<br>☐ Federal standard<br>☐ WHO or local branch<br>☐ Other
Specify other basis for evaluation of RetCam opthamological exam	__________________________________

Carabali M, *et al. BMJ Open* 2022; 12:e064362. doi: 10.1136/bmjopen-2022-064362

Auditory abnormalities

Did the study measure auditory abnormalities?	◯ Yes<br>◯ No
How did the study measure auditory abnormalities?<br><br>Select all that apply	☐ impedance audiometry<br>☐ otoacoustic emissions (OAEs)<br>☐ other
How else did the study measure auditory abnormalities?	__________________________
Was impedance audiometry performed at birth or within the first 24-48 hours after birth?	◯ Yes<br>◯ No
Type of information used to assess auditory abnormalities	◯ Presence vs absence of alterations<br>◯ Severity of nerve damage<br>◯ Other
Specify other type of information used to assess auditory abnormalities	__________________________________
At what age(s) did the study plan to measure auditory function?	__________________________
Did the study perform an Auditory Brainstem Response (ABR) test?	◯ Yes<br>◯ No
How many times did the study plan to perform ABR on each infant/child?	__________________________
At what ages did the study perform ABR?	__________________________
Specify any other auditory tests performed by study	__________________________
Reference standard used for evaluation of impedance audiometry exam<br><br>Select all that apply	☐ Provided by manufacturer<br>☐ In-hospital standard<br>☐ Regional or state standard<br>☐ Federal standard<br>☐ WHO or local branch<br>☐ Other
Specify other reference standard used	__________________________________
Reference standard used for evaluation of OAE<br><br>Select all that apply	☐ Provided by manufacturer<br>☐ In-hospital standard<br>☐ Regional or state standard<br>☐ Federal standard<br>☐ WHO or local branch<br>☐ Other

Specify other reference standard used ____________________

Confidential

Additional abnormalities

In addition to the ocular and auditory outcomes described previously, which other infant outcomes were assessed by the study?<br><br>Select all that apply	☐ Motor abnormalities (hypotonia, hypertonia, hyperreflexia, spasticity, clonus, extrapyramidal symptoms)<br>☐ Congenital contractures (arthrogryposis, uni or bilateral clubfoot)<br>☐ Craniofacial disproportion<br>☐ Seizures, epilepsy<br>☐ Postnatal intraventricular hemorrhage<br>☐ Other non-neurologic congenital abnormalities
How were congenital contractures defined?	__________________________________
Which other non-neurologic congenital abnormalities were assessed by the study?	__________________________________

Developmental screening tools

Study administered the Bayley Scales of Infant Development (BSID)?	○ Yes<br>○ No
Who administered the Bayleys?	○ Pediatrician<br>○ Neuropediatrician<br>○ Other
Who else administered the Bayleys?	__________________
Describe the training of the person who administered the Bayleys	__________________
Was the person evaluating the Bayleys blinded to ZIKV status of the mother?	○ Blinded<br>○ Unblinded
How many times did the study plan to administer the Bayleys with each infant or child?	__________________
List ages (in months) at which the study planned to administer the Bayleys:	__________________
Did the study assess all 5 domains measured by the Bayleys? (i.e., receptive language, expressive language, fine motor, gross motor, and cognitive)	○ Yes<br>○ No
If all 5 domains were not assessed, which of the Bayleys scales were administered?<br><br>Select all that apply	☐ Receptive language<br>☐ Expressive language<br>☐ Fine motor<br>☐ Gross motor<br>☐ Cognitive
Study administered the Ages and Stages Questionnaire (ASQ)?	○ Yes<br>○ No
Was the person administering the ASQ blinded to the ZIKV status of the mother?	○ Blinded<br>○ Unblinded
How many times did the study plan to administer the ASQ with each infant or child?	__________________
List all ages (in months) at which the study planned to adminster the ASQ	__________________
Study administered the Warner Initial Developmental Evaluation of Adaptive and Functional Skills (WIDEA-FS)?	○ Yes<br>○ No
Was the person administering the WIDEA-FS blinded to the ZIKV status of the mother?	○ Blinded<br>○ Unblinded
How many times did the study plan to administer the WIDEA-FS with each child?	__________________

List all ages (in months) at which the study planned to adminster the WIDEA-FS	__________________
Study administered the Alberta Infant Motor Skills (AIMS) assessment?	◯ Yes<br>◯ No
Describe how the person evaluating the AIMS was trained	__________________
Who administered the AIMS?	◯ Pediatrician<br>◯ Neuropediatrician<br>◯ Other
Who else administered the AIMS?	__________________
Was the person administering the AIMS blinded to the ZIKV status of the mother?	◯ Blinded<br>◯ Unblinded
How many times did the study plan to administer the AIMS with each infant or child?	__________________
List all ages (in months) at which the study planned to adminster the AIMS	__________________
Study administered the Oxford Neurodevelopmental Assessment (Ox-NDA)?	◯ Yes<br>◯ No
Describe the training of the person administering the Ox-NDA	__________________
Was the person administering the Ox-NDA blinded to the ZIKV status of the mother?	◯ Blinded<br>◯ Unblinded
How many times did the study plan to administer the Ox-NDA with each infant or child?	__________________
List all ages (in months) at which the study planned to adminster the Ox-NDA	__________________
Study administered the Intergrowth-21st Neurodevelopmental Assessment (INTER-NDA)?	◯ Yes<br>◯ No
Describe the training of the person administering the INTER-NDA	__________________
Was the person administering the INTER-NDA blinded to the ZIKV status of the mother?	◯ Blinded<br>◯ Unblinded
List all ages (in months) at which the study planned to adminster the INTER-NDA	__________________

Carabali M, *et al. BMJ Open* 2022; 12:e064362. doi: 10.1136/bmjopen-2022-064362

Confidential

In addition to the instruments mentioned above, which of the following developmental screening tools did the project administer?<br><br>Select all that apply	☐ BDI (Batelle Developmental Inventory) 0-7 yrs<br>☐ BITSEA/ITSEA (Brief) Infant-Toddler Social and Emotional Assessment 1-3 yrs<br>☐ CDI (MacArthur-Bates Communication Development Inventory), 8-30 months<br>☐ DDST or DENVER II (Denver Developmental Screening Test) 0-6 yrs<br>☐ DECA (Devereaux Early Childhood Assessment) 1 months-6 years<br>☐ GMFCS (Gross Motor Function Classification System) 0-18 yrs<br>☐ Greenspan Social-Emotional Growth Chart 0-42 months<br>☐ HELP (Hawaii Early Learning Profile) 0-3 yrs<br>☐ HOME (Home Observation Measurement of the Environment) 0 and up<br>☐ MSEL (Mullen Scales of Early Learning) 0-7 yrs<br>☐ VABS (Vineland Adaptive Behavior Scales) 0 and up<br>☐ PDDST (Pervasive Developmental Disorders Screening Test) 1-4 yrs<br>☐ PEDI (Pediatric Evaluation of Disability Inventory) 6 months-7.5 yrs<br>☐ Other<br>☐ No additional infant development screening tools were administered by the project
Specify other infant developmental screening tools administered by project	__________________________________
Did the project administer any of the following development screening tools for toddlers and older?<br><br>Select all that apply	☐ BSRA (Bracken School Readiness Assessment) 2.6 years and up<br>☐ CBCL (Achenbach Child Behavior Checklist) 6-18 years<br>☐ SDQ (Strengths and Difficulties Questionnaire) 3-16 years<br>☐ Other<br>☐ No other toddler-and-above development screening tools were administered by the project than those mentioned previously
Specify other toddler developmental screening tools administered by project	__________________________________

Confidential

Sociodemographic variables and maternal mental health

Did the study measure maternal ethnicity or minority status?	◯ Yes, self defined<br>◯ Yes, interviewer or study defined<br>◯ Yes, as reported in participant medical record<br>◯ No, not measured
Did the study measure household SES?	◯ Yes, household income (self reported)<br>◯ Yes, country-level measure of SES (self reported)<br>◯ Yes, country-level measure of SES (verified)<br>◯ Other<br>◯ No, not measured
Specify other measurement of household SES	__________________________
Did the study measure maternal education?	◯ Yes<br>◯ No
Did the study measure maternal occupation?	◯ Yes<br>◯ No
Did the study measure maternal marital status?	◯ Yes<br>◯ No
List any maternal mental health scales that were administered by the study<br><br>Select all that apply	☐ BAI (Beck Anxiety Inventory)<br>☐ BDI (Beck Depression Inventory)<br>☐ BDI-II (Beck Depression Inventory II)<br>☐ CBS (Caregiver Burden Scale)<br>☐ CES-D (Center for Epidemiologic Studies Depression Scale)<br>☐ DASS-21 (Depression, Anxiety, and Stress Scale - Short Form)<br>☐ Patient Health Questionnaire-9 (PHQ-9)<br>☐ Patient Health Questionnaire-2 (PHQ-2)<br>☐ Edinburgh Postnatal Depression Scale (EPDS)<br>☐ MSPSS (Multidimensional Scale of Perceived Social Support)<br>☐ PSI (Parenting Stress Index)<br>☐ PSI-SF (Parenting Stress Index - Short Form)<br>☐ WHOQOL-BREF (WHO Quality of Life)<br>☐ Other<br>☐ No maternal mental health or parenting stress scales were administered by the project
Specify other maternal mental health screening tools administered by the study	________________________________

Pre-pregnancy-related covariates

Question	Options
Did the study measure pre-pregnancy maternal exposure to clinically suspected arboviruses, such as dengue and chikungunya, with specific or proxy dates?	◯ Yes, self reported<br>◯ Yes, medical/clinical chart<br>◯ No, not measured
Did the study measure pre-pregnancy maternal exposure to workplace teratogens?	◯ Yes, self reported<br>◯ Yes, medical/clinical chart<br>◯ No, not measured
Did the study measure pre-pregnancy exposure to intimate partner violence?	◯ Yes, self reported<br>◯ Yes, medical/clinical chart<br>◯ No, not measured
Did the study measure number of previous live births (parity)?	◯ Yes, self reported<br>◯ Yes, medical/clinical chart<br>◯ No, not measured
Did the study measure the number of previous total pregnancies (gravidity)?	◯ Yes, self reported<br>◯ Yes, medical/clinical chart<br>◯ No, not measured
Did the study measure the number of previous pregnancies with any complications	◯ Yes, self reported<br>◯ Yes, medical/clinical chart<br>◯ No, not measured
Did the study measure the number of previous term pregnancies?	◯ Yes, self reported<br>◯ Yes, medical/clinical chart<br>◯ No, not measured
Did the study measure the number of previous preterm pregnancies?	◯ Yes, self reported<br>◯ Yes, medical/clinical chart<br>◯ No, not measured
Did the study measure the number of previous induced or spontaneous abortions?	◯ Yes, self reported<br>◯ Yes, medical/clinical chart<br>◯ No, not measured
Did the study measure the number of previous stillbirths?	◯ Yes, self reported<br>◯ Yes, medical/clinical chart<br>◯ No, not measured
Did the study measure the number of previous pregnancies with preterm labor?	◯ Yes, self reported<br>◯ Yes, medical/clinical chart<br>◯ No, not measured
Did the study measure the number of previous pregnancies with eclampsia?	◯ Yes, self reported<br>◯ Yes, medical/clinical chart<br>◯ No, not measured
Did the study measure the number of previous pregnancies with preeclampsia?	◯ Yes, self reported<br>◯ Yes, medical/clinical chart<br>◯ No, not measured
Did the study measure the number of previous pregnancies with gestational diabetes?	◯ Yes, self reported<br>◯ Yes, medical/clinical chart<br>◯ No, not measured

Supplemental material BMJ Publishing Group Limited (BMJ) disclaims all liability and responsibility arising from any reliance placed on this supplemental material which has been supplied by the author(s) BMJ Open

Did the study measure the number of previous pregnancies with birth defects?	◯ Yes, self reported<br>◯ Yes, medical/clinical chart<br>◯ No, not measured
Did the study measure history of pregnancies with fetus or infant affected by genetic disorders and/or congenital abnormalities?	◯ Yes, self reported<br>◯ Yes, medical/clinical chart<br>◯ No, not measured

Carabali M, *et al. BMJ Open* 2022; 12:e064362. doi: 10.1136/bmjopen-2022-064362

Current pregnancy

Did the study measure complications related to the current pregnancy	◯ Yes, self reported<br>◯ Yes, medical/clinical chart<br>◯ No, not measured
Did the study measure gestational diabetes?	◯ Yes<br>◯ No
Did the study measure maternal body mass index (BMI)?	◯ Yes<br>◯ No

Ascertainment of maternal exposure to other infectious diseases

How did the study determine maternal HIV status?

Select all that apply

- ☐ Self report
- ☐ Medical records
- ☐ Diagnostic test
- ☐ Other
- ☐ Not measured

Specify other means of determining maternal HIV status

____

When was maternal HIV status determined?

Select all that apply

- ☐ 1st term
- ☐ 2nd term
- ☐ 3rd term
- ☐ whenever symptoms were present (per clinical indication)
- ☐ other

At what other time was maternal HIV status determined?

____

What diagnostic tests were applied to assess maternal HIV infection status?

Select all that apply

- ☐ serology
- ☐ RT-PCR
- ☐ other NAAT
- ☐ rapid diagnostic test (RDT)
- ☐ other

Which other diagnostic tests were applied to assess maternal HIV infection status?

____

How did the study determine maternal malaria infection (fever, placental parasitemia)?

Select all that apply

- ☐ Self report
- ☐ Medical records
- ☐ Diagnostic test
- ☐ Other
- ☐ Not measured

Specify other means of determining malaria infection

____

When was maternal malaria status determined?

Select all that apply

- ☐ 1st term
- ☐ 2nd term
- ☐ 3rd term
- ☐ whenever symptoms were present (per clinical indication)
- ☐ other

At what other time was maternal malaria status determined?

____

How did the study determine maternal parvovirus infection?

Select all that apply

- ☐ Self report
- ☐ Medical records
- ☐ Diagnostic test
- ☐ Other
- ☐ Not measured

Specify other means of determining parvovirus infection

____

When was maternal parvovirus status determined?<br><br>Select all that apply	☐ 1st term<br>☐ 2nd term<br>☐ 3rd term<br>☐ whenever symptoms were present (per clinical indication)<br>☐ other
At what other time was maternal parvovirus status determined?	________________________
How did the study determine maternal listeria infection (fever, placental parasitemia)?<br><br>Select all that apply	☐ Self report<br>☐ Medical records<br>☐ Diagnostic test<br>☐ Other<br>☐ Not measured
Specify other means of determining listeria infection	________________________
When was maternal listeria status determined?<br><br>Select all that apply	☐ 1st term<br>☐ 2nd term<br>☐ 3rd term<br>☐ whenever symptoms were present (per clinical indication)<br>☐ other
At what other time was maternal listeria status determined?	________________________
Other than HIV and malaria, which STORCH pathogens were measured during pregnancy?<br><br>Select all that apply	☐ Syphilis<br>☐ Toxoplasmosis<br>☐ Other (varicella, parovirus B19)<br>☐ Rubella<br>☐ CMV<br>☐ Herpes
What other pathogens were measured during pregnancy?	________________________
How did the study determine maternal syphilis infection status?<br><br>Select all that apply	☐ Self report<br>☐ Medical records<br>☐ Diagnostic test<br>☐ Other
Specify other means of determining maternal syphilis infection status	________________________
Which diagnostic test(s) were applied to assess maternal syphilis infection status?<br><br>Select all that apply	☐ serology<br>☐ RT-PCR<br>☐ immunohistology<br>☐ darkfield microscopy<br>☐ other
What other diagnostic tests were applied to assess maternal syphilis infection status?	______________________________

Confidential

Which serologic assay(s) was/were applied to assess maternal syphilis infection status?<br><br>Select all that apply	☐ TPPA (Treponema pallidum particle agglutination assay)<br>☐ TPHA (Treponema pallidum hemagglutination assay)<br>☐ Microhemagglutination assay (MHA-TP)<br>☐ EIA IgG<br>☐ EIA IgM<br>☐ IgM Immunoblot<br>☐ FTA-ABS (Fluorescent treponemal antibody absorption test)<br>☐ 19S-IgM-FTA-ABS<br>☐ Venereal Disease Research Laboratory test (VDRL)<br>☐ Rapid-Plasma-Reagin (RPR)<br>☐ Other
Which other serologic assay(s) was/were applied to assess maternal syphilis infection status?	__________________________________
When during pregnancy did the study plan to diagnose syphilis infection?<br><br>Select all that apply	☐ 1st term<br>☐ 2nd term<br>☐ 3rd term<br>☐ whenever symptoms were present (per clinical indication)<br>☐ other
At what other time during pregnancy did the study plan to diagnose syphilis infection?	__________________________________
How did the study determine maternal toxoplasmosis infection status?<br><br>Select all that apply	☐ Self report<br>☐ Medical records<br>☐ Diagnostic test<br>☐ Other
Specify other means of determining maternal toxoplasmosis infection status	____________________________
Which diagnostic test(s) were applied to assess maternal toxoplasmosis infection status?<br><br>Select all that apply	☐ serology<br>☐ RT-PCR<br>☐ immunohistology<br>☐ darkfield microscopy<br>☐ other
What other diagnostic tests were applied to assess maternal toxoplasmosis infection status?	__________________________________
Which serologic assay(s) was/were applied to assess maternal toxoplasmosis infection status?<br><br>Select all that apply	☐ IgM<br>☐ IgG<br>☐ IgA<br>☐ Other
Which other serologic assay(s) was/were applied to assess maternal toxoplasmosis infection status?	__________________________________

Confidential

When during pregnancy did the study plan to diagnose toxoplasmosis infection?<br><br>Select all that apply	☐ 1st term<br>☐ 2nd term<br>☐ 3rd term<br>☐ whenever symptoms were present (per clinical indication)<br>☐ other
At what other time during pregnancy did the study plan to diagnose toxoplasmosis infection?	__________________________________
How did the study determine maternal rubella infection status?<br><br>Select all that apply	☐ Self report<br>☐ Medical records<br>☐ Diagnostic test<br>☐ Other
Specify other means of determining maternal rubella infection status	____________________________
Which diagnostic test(s) were applied to assess rubella infection status?<br><br>Select all that apply	☐ serology<br>☐ RT-PCR<br>☐ immunohistology<br>☐ darkfield microscopy<br>☐ other
What other diagnostic tests were applied to assess maternal rubella infection status?	__________________________________
Which serologic assay(s) was/were applied to assess maternal rubella infection status?<br><br>Select all that apply	☐ TPPA (Treponema pallidum particle agglutination assay)<br>☐ TPHA (Treponema pallidum hemagglutination assay)<br>☐ Microhemagglutination assay (MHA-TP)<br>☐ EIA IgG<br>☐ EIA IgM<br>☐ IgM Immunoblot<br>☐ FTA-ABS (Fluorescent treponemal antibody absorption test)<br>☐ 19S-IgM-FTA-ABS<br>☐ Venereal Disease Research Laboratory test (VDRL)<br>☐ Rapid-Plasma-Reagin (RPR)<br>☐ Other
Which other serologic assay(s) was/were applied to assess maternal rubella infection status?	__________________________________
When during pregnancy did the study plan to diagnose rubella infection?<br><br>Select all that apply	☐ 1st term<br>☐ 2nd term<br>☐ 3rd term<br>☐ whenever symptoms were present (per clinical indication)<br>☐ other
At what other time during pregnancy did the study plan to diagnose maternal rubella infection?	__________________________________

How did the study determine maternal CMV infection status?<br><br>Select all that apply	☐ Self report<br>☐ Medical records<br>☐ Diagnostic test<br>☐ Other
Specify other means of determining maternal CMV infection status	__________________________
Which diagnostic test(s) were applied to assess maternal CMV infection status?<br><br>Select all that apply	☐ serology<br>☐ RT-PCR<br>☐ immunohistology<br>☐ darkfield microscopy<br>☐ other
What other diagnostic tests were applied to assess maternal CMV infection status?	__________________________
Which serologic assay(s) was/were applied to assess maternal CMV infection status?<br><br>Select all that apply	☐ TPPA (Treponema pallidum particle agglutination assay)<br>☐ TPHA (Treponema pallidum hemagglutination assay)<br>☐ Microhemagglutination assay (MHA-TP)<br>☐ EIA IgG<br>☐ EIA IgM<br>☐ IgM Immunoblot<br>☐ FTA-ABS (Fluorescent treponemal antibody absorption test)<br>☐ 19S-IgM-FTA-ABS<br>☐ Venereal Disease Research Laboratory test (VDRL)<br>☐ Rapid-Plasma-Reagin (RPR)<br>☐ Other
Which other serologic assay(s) was/were applied to assess maternal CMV infection status?	__________________________
Did the study conduct avidity assays for IgG positive CMV?	◯ Yes<br>◯ No
When during pregnancy did the study plan to diagnose maternal CMV infection?<br><br>Select all that apply	☐ 1st term<br>☐ 2nd term<br>☐ 3rd term<br>☐ whenever symptoms were present (per clinical indication)<br>☐ other
At what other time during pregnancy did the study plan to diagnose CMV infection?	__________________________
How did the study determine maternal herpes infection status?<br><br>Select all that apply	☐ Self report<br>☐ Medical records<br>☐ Diagnostic test<br>☐ Other
Specify other means of determining maternal herpes infection status	__________________________

Which diagnostic test(s) were applied to assess maternal herpes infection status?<br><br>Select all that apply	☐ serology<br>☐ RT-PCR<br>☐ immunohistology<br>☐ darkfield microscopy<br>☐ other
What other diagnostic tests were applied to assess maternal herpes infection status?	__________________________________
Which serologic assay(s) was/were applied to assess maternal herpes infection status?<br><br>Select all that apply	☐ TPPA (Treponema pallidum particle agglutination assay)<br>☐ TPHA (Treponema pallidum hemagglutination assay)<br>☐ Microhemagglutination assay (MHA-TP)<br>☐ EIA IgG<br>☐ EIA IgM<br>☐ IgM Immunoblot<br>☐ FTA-ABS (Fluorescent treponemal antibody absorption test)<br>☐ 19S-IgM-FTA-ABS<br>☐ Venereal Disease Research Laboratory test (VDRL)<br>☐ Rapid-Plasma-Reagin (RPR)<br>☐ Other
Which other serologic assay(s) was/were applied to assess maternal herpes infection status?	__________________________________
When during pregnancy did the study plan to diagnose herpes infection?<br><br>Select all that apply	☐ 1st term<br>☐ 2nd term<br>☐ 3rd term<br>☐ whenever symptoms were present (per clinical indication)<br>☐ other
At what other time during pregnancy did the study plan to diagnose herpes infection?	__________________________________

Confidential

Additional pregnancy-level maternal exposures

How did the study determine maternal nutrition status?<br><br>Select all that apply	☐ Self report<br>☐ Medical records<br>☐ Empirical evaluation of nutrition status<br>☐ Other<br>☐ Not measured
Specify other means of determining maternal nutrition status	__________________________________
How did the study determine maternal smoking during pregnancy?<br><br>Select all that apply	☐ Self report<br>☐ Medical records<br>☐ Other<br>☐ Not measured
Specify other means of determining maternal smoking during pregnancy	__________________________________
How did the study determine maternal alcohol consumption?<br><br>Select all that apply	☐ Self report<br>☐ Medical records<br>☐ Other<br>☐ Not measured
Specify other means of determining maternal alcohol consumption	__________________________________
How did the study determine maternal recreational drug use?<br><br>Select all that apply	☐ Self report<br>☐ Medical records<br>☐ Other<br>☐ Not measured
Specify other means of determining maternal recreational drug use	__________________________________
How did the study determine prescription drug or other medication use during pregnancy?<br><br>Select all that apply	☐ Self report<br>☐ Medical records<br>☐ Other<br>☐ Not measured
Specify other means of determining maternal prescription drug or medication use	__________________________________
How were vaccinations during pregnancy determined?<br><br>Select all that apply	☐ Self report<br>☐ Medical records<br>☐ Other<br>☐ Not measured
Specify other means of determining vaccinations during pregnancy	__________________________________
How did the study determine exposure to workplace or environmental teratogens (glycol ethers, solvents, heavy metals, pesticides) during pregnancy?<br><br>Select all that apply	☐ Self report<br>☐ Medical records<br>☐ Other<br>☐ Not measured

Confidential

Specify other means of determining exposure to workplace or environmental teratogens	__________________________________
How was exposure to intimate partner violence during pregnancy determined?<br><br>Select all that apply	☐ Self report<br>☐ Medical records<br>☐ Other<br>☐ Not measured
Specify other means of determining exposure to intimate partner violence during pregnancy	__________________________________

Carabali M, *et al. BMJ Open* 2022; 12:e064362. doi: 10.1136/bmjopen-2022-064362

Other infant/child-level covariates

How was infant/child exposure to family violence determined?<br><br>Select all that apply	☐ Self report<br>☐ Medical records<br>☐ Other<br>☐ Not measured
Specify other means of determining infant/child exposure to family violence	__________________________
Did the study measure family structure after the birth of the index child?	◯ Yes<br>◯ No
Do infants or children in the study receive any developmental interventions (e.g. physical, speech, motor therapy) provided by the study or by the health system?	◯ Yes<br>◯ No<br>◯ Not measured
Please describe the physical therapy/motor therapy interventions received by infants or children in the study	__________________________
Please describe the speech/language therapy interventions received by infants or children in the study	__________________________
Please describe the cognitive therapy interventions received by infants or children in the study	__________________________
Please describe any other types of therapies or developmental interventions received by infants or children in the study	__________________________
