# Supplementary material for: Heterogeneity of Zika virus exposure and outcome ascertainment across cohorts of pregnant women, their infants and their children: a metadata survey
Source: BMJ Open. 2022 Nov 22;12(11):e064362. doi: 10.1136/bmjopen-2022-064362 (PMC9685007; doi:10.1136/bmjopen-2022-064362)
Supplement: Supplementary data [file bmjopen-2022-064362supp003.pdf]

## Appendix 3

## ZIKV-IPD-MA Consortium participants

**Pablo Aguilar Ticona, MD**
Institute of Collective Health
Federal University of Bahia
Salvador, Brazil

**Luiz Carlos Junior Alcantara, PhD**
Flavivirus Reference Laboratory (LABFLA) from Oswaldo Cruz Institution (IOC)
Oswaldo Cruz Foundation (FIOCRUZ)
Rio de Janeiro, Brazil

**Jackeline Alger, MD, PhD**
Facultad de Ciencias Médicas
Universidad Nacional Autónoma de Honduras
Tegucigalpa, Honduras

**Celia Alpuche Aranda, MD, PhD**
CISEI Centro de Investigación Sobre Enfermedades Infecciosas
Institution Instituto Nacional de Salud Publica de México
Cuernavaca, México

**Jonathan Altamirano, MS**
Department of Pediatrics
Division of Infectious Diseases
Stanford University School of Medicine
Stanford, California, USA

**Juan Arias, MD, PhD**
Infectious Diseases
St Jude's Children's Research Hospital
Memphis, Tennessee, USA

**Lumumba Arriaga-Nieto, MD**
Instituto Mexicano del Seguro Social
Mexico City, Mexico
**Melissa A. G. Avelino, PhD**
Department of Surgery of Goiás Federal University
Goiânia, Goiás, Brazil
**Angel Balmaseda, MD**
Department of Virology
Sustainable Sciences Institute
Managua, Nicaragua

**Azucena Bardají, MD, PhD**
ISGlobal
Hospital Clínic-Universitat de Barcelona
Barcelona, Spain

Consorcio de Investigación Biomédica en Red de Epidemiología y Salud Pública (CIBERESP)
Madrid, Spain

Centro de Investigação em Saúde de Manhiça (CISM)
Maputo, Mozambique

**Carlos Hernan Becerra Mojica, MD**
Departamento de Obstetricia y Ginecologia
Universidad Industrial de Santander

Bucaramanga, Colombia

**Mónica Benavides, MPH**
Dirección de Vigilancia y Análisis del Riesgo en Salud Publica
Grupo de enfermedades Inmunoprevenibles
Instituto Nacional de Salud
Bogotá, Colombia

**A.P. Bertozzi, MD, PhD**
Department of Pediatrics
Faculty of Medicine of Jundiaí
São Paulo, Brazil

**Karen Blackmon, PhD**
Department of Psychiatry and Psychology
Mayo Clinic
Jacksonville, FL, USA

**Victor Hugo Borja Aburto, MD**
Instituto Mexicano del Seguro Social
Mexico City, Mexico

**Patrícia Brasil, MD, PhD**
Laboratório de Pesquisa Clínica em Doenças Febris Agudas
Instituto Nacional de Infectologia Evandro Chagas - FIOCRUZ
Rio de Janeiro, Brazil

**William J Britt, MD**
Department of Pediatrics
Heersink School of Medicine
University of Alabama
Birmingham, AL

**Nathalie Broutet, MD, PhD**
UNDP/UNFPA/UNICEF/WHO/World Bank Special Programme of Research, Development and Research Training in Human Reproduction
Department of Sexual and Reproductive Health and Research
World Health Organization
Geneva, Switzerland

**Pierre Buekens, MD, PhD**
School of Public Health and Tropical Medicine
Tulane University
Louisiana, USA

**André Cabié, MD, PhD**
Infectious Diseases and Tropical Medicine
Université des Antilles, CHU de Martinique, INSERM CIC 1424, INSERM PCCEI UMR 1058
Fort-de-France, Martinique, France

**David Alejandro Cabrera-Gaytán, MD**
Instituto Mexicano del Seguro Social
Mexico City, Mexico

**Rodrigo Cachay Figueroa, MD**

Instituto de Medicina Tropical Alexander von Humboldt
Universidad Peruana Cayetano Heredia
Lima, Perú

**María Luisa Cafferata, MD**
Mother and Children Health Research Department
Institute for Clinical Effectiveness and Health Policy
Buenos Aires, Argentina

**Juan Ignacio Calcagno, MD**
Department of Hospital Epidemiology
Maternidade Professor José Maria de Magalhães Netto
Salvador, Bahia, Brazil

**Juan P. Calle, MD**
Department of Pediatrics, Universidad del Quindío
Centro de Estudios en Infectología Pediátrica
Cali, Colombia

**Mabel Carabali, MD, PhD**
Département de Médecine Sociale et Préventive, École de Santé Publique,
Université de Montréal,
Montreal, Quebec, Canada.

Department of Epidemiology, Dalla Lana School of Public Health,
University of Toronto.
Toronto, Ontario, Canada.

**Derrick WS Chan, MD**
Paediatric Neurology, Paediatric Medicine
KK Women's and Children's Hospital
Singapore

**Celia CD Christie, MBBS, DM Peds**
Department of Child and Adolescent Health
University of the West Indies, Mona Campus
Kingston, Jamaica

**Federico Costa, PhD**
Institute of Collective Health
Federal University of Bahia
Salvador, Brazil

**Antonio Jose Cunha, PhD**
Departamento de Pediatria
Federal University of Rio de Janeiro
Rio de Janeiro, Brazil

**Carlos Cure Cure, MD**
BIOMELAB SAS
Barranquilla, Colombia

**Johanna Antonia Adriana Damen, PhD**
Julius Center for Health Sciences and Primary Care
University Medical Center Utrecht
Utrecht University

Utrecht, Netherlands

**Marcela C. Daza, Médica Pediatra, MSc**
Grupo de Investigación en Salud Materna y Perinatal
Dirección de Investigación en Salud Pública
Instituto Nacional de Salud
Bogotá, Colombia

**Roberta L. DeBiasi, MD**
Departments of Pediatrics and Microbiology, Immunology and Tropical Medicine
The George Washington University School of Medicine
Children's National Hospital
Washington, DC, USA

**Thomas Debray, PhD**
Julius Center for Health Sciences and Primary Care
University Medical Center Utrecht
Utrecht University
Utrecht, Netherlands

**Valentijn M.T. de Jong, PhD**
Julius Center for Health Sciences and Primary Care
University Medical Center Utrecht
Utrecht University
Utrecht, Netherlands

Data Analytics and Methods Task Force
European Medicines Agency
Amsterdam, Netherlands
**Camille Delgado López, MPH**
Puerto Rico Department of Health
San Juan, Puerto Rico

**Leah deWilde, BS**
Epidemiology Division
Virgin Islands Department of Health
Christiansted, St. Croix, Virgin Islands, USA

**Alan Oliveira Duarte, MSc**
Instituto Gonçalo Moniz
Fundação Oswaldo Cruz
Salvador, Bahia, Brazil

**Geraldo Duarte, MD, PhD**
Ribeirão Preto Medical School
University of São Paulo
São Paulo, Brazil

**Valorie Eckert, MPH**
California Birth Defects Monitoring Program
Genetic Disease Screening Division
California Department of Public Health
Richmond, CA USA

**Esther M. Ellis, PhD**
Territorial Epidemiologist

Virgin Islands Department of Health
Christiansted, St. Croix, Virgin Islands, USA

**Andres Espinosa-Bode, MD**
Division of Global Health Protection, Central America Region Office
Centers for Disease Control and Prevention
Atlanta, GA, USA

**Cassia Fernanda Estofolete, MD, PhD**
Department of Dermatologic, Infectious and Parasititc Diseases
Faculdade de Medicina de São José do Rio Preto
São José do Rio Preto, Brazil

**Roberta Evans, MS**
Windward Islands and Research Education Foundation
St. George, Grenada

**Valéria Christina de Rezende Féres, PhD**
Laboratório BIOTEC
Faculdade de Farmácia - Universidade Federal de Goiás
Setor Leste Universitário, Goiânia, Brasil

**Fabíola Fiaccadori, PhD**
Virology and Cell Culture Laboratory
Institute of Tropical Pathology and Public Health
Federal University of Goiás
Goiânia, Goiás, Brazil

**Lester Fernando Figueroa Bolaños, MD**
Departamento de Planificacion
Instituto de Nutricion de Centroamerica y Panama (INCAP)
Guatemala City, Guatemala

**Olivier Fléchelles, MD, PhD**
Pediatric and Neonatal Intensive Care Unit
University Hospital of Martinique
Martinique, France

**Arnaud Fontanet, MD, DrPH**
Global Health
Institut Pasteur
Paris, France

**Victoria Fumadó, MD, PhD**
Infectious and Imported Diseases Unit
Department of Pediatrics, Sant Joan de Déu University
Hospital Research Foundation
Barcelona, Spain

**Anna L. Funk, PhD**
Department of Pediatrics, Cumming School of Medicine
University of Calgary
Calgary, Alberta, Canada

**Anna Gajewski, MPH**
Sustainable Sciences Institute

Managua, Nicaragua

**Rosa Margarita Gelvez Ramirez, MSc**
Centro de Atención y Diagnóstico de Enfermedades Infecciosas-CDI
Santander, Colombia

**Carlo Giaquinto, MD**
Dipartimento di Salute della Donna e del Bambino
Università degli Studi di Padova
Italy

**Luz Gibbons, MSc**
Institute for Clinical Effectiveness and Health Policy
Buenos Aires, Argentina

**Suzanne M. Gilboa, PhD**
National Center on Birth Defects and Developmental Disabilities
Centers for Disease Control and Prevention
Atlanta, GA, USA

**Maria Barbara Franco Gomes, MD**
Maternal Infant Hospital
Goiania, Brazil

**Anna Goncé, MD, PhD**
Barcelona Centre for Maternal-Fetal and Neonatal Medicine (Hospital Clínic and Hospital Sant Joan de Déu)

Institut d'Investigacions Biomèdiques August Pi i Sunyer (IDIBAPS)
Universitat de Barcelona

Centre for Biomedical Research on Rare Diseases (CIBER-ER)
Barcelona, Spain

**Cesar R Gonzalez-Bonilla, MD, PhD**
Coordinación de Investigación en Salud
Instituto Mexicano del Seguro Social
Mexico City, Mexico

**Eduardo Gotuzzo, MD**
Instituto de Medicina Tropical Alexander Von Humboldt
Universidad Peruana Cayetano Heredia
Lima, Peru

**Concepción Grajales-Muñiz, MD**
Instituto Mexicano del Seguro Social
Mexico City, Mexico

**Rebecca Grant, MSc, MPH**
Epidemiology of Emerging Diseases Unit
Institut Pasteur
Paris, France

**Elysse Grossi-Soyster, PhD(c)**
Sarnow and Schnieder Labs
Microbiology & Immunology Department
Stanford University School of Medicine

**Tahani Hamdan, MPH**
Houston Health Department
City of Houston
Houston, TX, USA

**Eva Harris, PhD**
Division of Infectious Diseases and Vaccinology, School of Public Health
University of California, Berkeley
Berkeley, CA, USA

**Cosme Harrison, MPH**
Division of Epidemiology
Virgin Islands Department of Health
Saint Thomas/Virgin Islands, USA

**Bruno Hoen, MD, PhD**
Infectious Diseases and Tropical Medicine
University hospital of Guadeloupe
Pointe-à-Pitre, Guadeloupe, France

**Cristina Hofer, PhD**
Instituto de Puericultura e Pediatria Martagão Gesteira
Universidade Federal do Rio de Janeiro
Rio de Janeiro, Brazil

**Natanael Holband, MD**
Academic Pediatric Center Suriname
Academic Hospital Paramaribo
Paramaribo, Suriname

**Ivonne Huerta, MD**
CISEI Centro de Investigación Sobre Enfermedades Infecciosas
Institution Instituto Nacional de Salud Publica de México
Cuernavaca, México

**Irene Inwani, MD**
Department of Paediatrics and Child Health
Kenyatta National Teaching and Referral Hospital
Nairobi, Kenya

**Thomas Jaenisch, MD, PhD**
Heidelberg Institute for Global Health
Heidelberg University Hospital
Heidelberg, Germany

Center for Global Health
Colorado School of Public Health
University of Colorado
Denver, CO, USA

**Esaú João, PhD**
Department of Infectious Diseases
Hospital Federal dos Servidores do Estado
Rio de Janeiro, Brazil

**Edna Kara, MD**
UNDP/UNFPA/UNICEF/WHO/World Bank Special Programme of Research, Development and Research Training in Human Reproduction
Department of Sexual and Reproductive Health and Research
World Health Organization
Geneva, Switzerland

**Salma Khuwaja, MD, DrPH**
Houston Health Department
City of Houston
Houston, TX, USA

**Caron Kim, MD**
UNDP/UNFPA/UNICEF/WHO/World Bank Special Programme of Research, Development and Research Training in Human Reproduction
Department of Sexual and Reproductive Health and Research
World Health Organization
Geneva, Switzerland

**Albert I. Ko, MD**
Department of Epidemiology of Microbial Diseases
Yale School of Public Health
New Haven, CT, USA

**Nancy F. Krebs, MD**
Department of Pediatrics, Section of Nutrition
University of Colorado School of Medicine
Aurora, CO, USA

**Angelle Desiree LaBeaud, MD**
Department of Pediatrics
Division of Infectious Diseases
Stanford University School of Medicine
Stanford, California, USA

**Heather Lake-Burger, MS, MPH**
Division of Community Health Promotion
Florida Department of Health
Tallahassee, FL, USA

**Ellen H. Lee, MD**
Bureau of Communicable Disease
New York City Department of Health and Mental Hygiene
New York, USA

**Vernon Lee, PhD**
Saw Swee Hock School of Public Health
National University of Singapore
Singapore

**Yee-Sin Leo, MD**
Executive Director's Office
National Centre for Infectious Diseases
Singapore

**Brooke Levis, PhD**
Centre for Clinical Epidemiology
Lady Davis Institute of Medical Research
Jewish General Hospital
Montreal, Quebec

Centre for Prognosis Research, School of Medicine,
Keele University,
Staffordshire, United Kindom

**Eduardo Lopez-Medina, MD**
Centro de Estudios en Infectología Pediátrica —CEIP—
Department of Pediatrics, Universidad del Valle
Clínica Imbanaco, Grupo Quironsalud
Cali, Valle, Colombia

**Anyela Lozano-Parra, MSc**
Facultad de Salud
Universidad Industrial de Santander
Santander, Colombia

**Elena Marbán-Castro, PhD**
ISGlobal
Hospital Clínic-Universitat de Barcelona
Barcelona, Spain

**Celina Maria Turchi Martelli, PhD**
The Research Centre Aggeu Magalhães
Fundação Oswaldo Cruz (Fiocruz)
Recife, Brazil

**Lauren Maxwell, PhD**
Heidelberg Institute for Global Health
Heidelberg University Hospital
Heidelberg, Germany

Department of Sexual and Reproductive Health and Research
Human Reproduction Programme
World Health Organization

**Clara Menéndez, MD, PhD**
ISGlobal
Hospital Clínic-Universitat de Barcelona
Barcelona, Spain

Consorcio de Investigación Biomédica en Red de Epidemiología y Salud Pública (CIBERESP)
Madrid, Spain

Centro de Investigação em Saúde de Manhiça (CISM)
Maputo, Mozambique

**Marcela Mercado Reyes, MSc**
Public Health Research Division
Instituto Nacional de Salud - Colombia
Bogotá, Colombia

**Conrado Milani Coutinho, MD, PhD**
Departmento de Ginecologia e Obstetrícia
Hospital das Clínicas da Faculdade de Medicina de Ribeirão Preto da Universidade de São Paulo
São Paulo, Brazil

**María Consuelo Miranda Montoya, MD**
Facultad de Salud Carrera
Universidad Industrial de Santander
Santander, Colombia

**Demócrito de Barros Miranda-Filho, PhD**
Faculty of Medical Sciences
Federal University of Pernambuco
Recife, Brazil

**Maria Elisabeth Moreira, PhD**
Department of Neonatology
Fundação Oswaldo Cruz (Fiocruz)
Rio de Janeiro, Brazil

**J. Glenn Morris, Jr., MD**
Emerging Pathogens Institute
University of Florida
Gainesville, FL, USA

**Sarah B. Mulkey, MD, PhD**
Departments of Neurology and Pediatrics
The George Washington University School of Medicine
Prenatal Pediatrics Institute
Children's National Hospital
Washington, DC, USA

**Johanna Munoz, PhD**
Julius Center for Health Sciences and Primary Care
University Medical Center Utrecht
Utrecht University
Utrecht, Netherlands

**José Esteban Muñoz-Medina, PhD**
Department of Epidemiology
Instituto Mexicano del Seguro Social
Mexico City, Mexico

**Peninah Munyua, PhD**
Division of Global Health Protection
US Centers for Disease Control and Prevention
Nairobi, Kenya

**Marisa Marcia Mussi-Pinhata, MD, PhD**
Departamento de Pediatria
Faculdade de Medicina de Ribeirão Preto da Universidade de São Paulo
São Paulo, Brazil

**Nivison Nery Jr., MSc**
Instituto de Saúde Coletiva

Federal University of Bahia
Salvador, Brazil

**M. Kariuki Njenga, PhD**
Paul Allen Center for Global Health
Washington State University
Nairobi, Kenya

**Mauricio L. Nogueira, MD, PhD**
Department of Dermatologic, Infectious and Parasititc Diseases
Faculdade de Medicina de São José do Rio Preto
São José do Rio Preto, Brazil

**Eric Osoro, MD**
Department of Global Health
Washington State University, Kenya
Nairobi, Kenya

**Miguel Parra-Saavedra, PhD**
Department of Gynecology and Obstetrics
Universidad Simon Bolivar, CEDIUL
Barranquilla, Colombia

**Saulo Passos, PhD**
Department of Pediatrics
Faculty of Medicine of Jundiaí
São Paulo, Brazil

**Bernadete Perez Coêlho MD, PhD**
Department of Public Health of the Medical Sciences Center
Federal University of Pernambuco
Recife, Pernambuco, Brasil

**Monika Piccardi RN, BSN, MS**
Office for and People with Special Healthcare Needs
Maryland Department of Health
Baltimore, MD, USA

**Léo Pomar, PhD**
Department of Obstetrics and Gynecology
Centre Hospitalier de l'Ouest Guyanais
Saint-Laurent du Maroni, French Guiana

Materno-fetal and Obstetrics Research Unit
Department Woman-mother-child
Lausanne University Hospital, Switzerland

School of Health Sciences (HESAV)
University of Applied Sciences and Arts Western Switzerland
Delémont, Switzerland

**Arnaldo Prata-Barbosa, PhD**
Department of Pediatrics
D'Or Institute for Research & Education
Rio de Janeiro, Brazil

**Ingrid Rabe, MBChB, MMed**
Emerging Diseases and Zoonoses Unit
Global Infectious Hazards
Health Emergencies Programme
World Health Organization
Geneva, Switzerland

**Mitermayer Reis, MD, PhD**
Laboratório de Patologia e Biologia Molecular
Fundação Oswaldo Cruz (Fiocruz)
Salvador, Brazil

**Hannah Rettler, MPH**
Bureau of Epidemiology
Utah Department of Health
Salt Lake City, USA

**Megan R. Reynolds, MPH**
National Center on Birth Defects and Developmental Disabilities
Centers for Disease Control and Prevention
Atlanta, GA, USA

**Ana Maria Rivera Casas, MD**
Maternal-Fetal Medicine
FUCS University
Bogota, Colombia

**Diana Patricia Rojas, MD, PhD**
WHE/GIH/EZD
Zika and Chikungunya
World Health Organization
Geneva, Switzerland

**Teresita Rojas-Mendoza, MD**
Instituto Mexicano del Seguro Social
Mexico City, Mexico

**Nicole Roth, MPH**
National Center on Birth Defects and Developmental Disabilities
Centers for Disease Control and Prevention
Atlanta, GA, USA

**Paola Mariela Saba Villarroel, PhD**
Unité des Virus Émergents
UVE: Aix-Marseille University-IRD 190-INSERM 1207-IHU Méditerranée Infection
Marseille, France

**Magda Sanz Cortes, MD, PhD**
Department of Obstetrics and Gynecology
Fetal Surgery and Intervention
Baylor College of Medicine
Houston, TX, USA

**Janet L. Sayers, MS**
UNDP/UNFPA/UNICEF/WHO/World Bank Special Programme of Research, Development and Research Training in Human Reproduction

Department of Sexual and Reproductive Health and Research
Human Reproduction Programme
World Health Organization
Geneva, Switzerland

**Deolinda Scalabrin, MD, PhD**
Instituto Gonçalo Moniz
Fundação Oswaldo Cruz (Fiocruz)
Salvador, Bahia, Brasil

**Lavinia Schuler-Faccini, MD, PhD**
Genetics Department
Hospital de Clinicas de Porto Alegre
Universidade Federal do Rio Grande do Sul
Porto Alegre, Rio Grande do Sul, Brazil

**Stacey Schultz-Cherry, PhD**
Department of Infectious Diseases
St Jude Graduate School
St. Jude Children's Research Hospital
Memphis, Tennessee, USA

**Kirstin Short, MPH**
Houston Health Department
City of Houston
Houston, Texas, USA

**Priya Shreedhar, MSc**
Heidelberg Institute of Global Health
Heidelberg University
Heidelberg, Germany

**Antônio Augusto Silva, PhD**
Department Public Health
Federal University of Maranhão
Sao Luis, Maranhão, Brazil

**Ronaldo Silva, PhD**
UNDP/UNFPA/UNICEF/WHO/World Bank Special Programme of Research, Development and Research Training in Human Reproduction
Department of Sexual and Reproductive Health and Research
Human Reproduction Programme

**Isadora Siqueira, MD, PhD**
Researcher in Public Health
Instituto Gonçalo Moniz-Fiocruz
Salvador-BA, Brasil

**Karen Sohan, MD**
Consultant Obstetrician & Gynaecologist
Diagnostic (Mother & Baby) Ltd
Trinidad & Tobago

**Carmen Soria-Segarra, MD PhD**
SOSECALI C. Ltda
Universidad Católica Santiago de Guayaquil

Guayaquil, Ecuador

**Antoni Soriano-Arandes, MD, PhD**
Paediatric Infectious Diseases and Immunodeficiencies Unit
Hospital Universitari Vall d'Hebron
Barcelona, Catalonia, Spain

**Patrícia da Silva Sousa, MD, PhD**
Child Neurology and Clinical Neurophysiology
Universidade Federal do Maranhão - UFMA
Sao Luis, Maranhão, Brazil

**Maria Benamor Teixeira, MD, PhD**
Department of Infectious Diseases
Hospital Federal dos Servidores do Estado
Rio de Janeiro, Brazil

**Claire Thorne, PhD**
Great Ormond Street Institute of Child Health
University College London
London, UK

**Soe Soe Thwin, PhD**
UNDP/UNFPA/UNICEF/WHO/World Bank Special Programme of Research, Development and Research Training in Human Reproduction
Department of Sexual and Reproductive Health and Research
Human Reproduction Programme
World Health Organization
Geneva, Switzerland

**Van T. Tong, MPH**
National Center on Birth Defects and Developmental Disabilities
Centers for Disease Control and Prevention
Atlanta, GA, USA

**Benoit Tressieres, MSc**
Centre d'Investigation Clinique Antilles Guyane, Inserm CIC 1424
CHU de la Guadeloupe
Pointe-à-Pitre, France

**Marília Dalva Turchi, PhD**
Institute of Tropical Pathology and Public Health
Federal University of Goias
Goiânia, Brazil

**Diana Valencia, MS**
National Center on Birth Defects and Developmental Disabilities
Centers for Disease Control and Prevention
Atlanta, GA, USA

**Miguel Valencia-Prado, MD**
Puerto Rico Department of Health
San Juan, Puerto Rico

**Alfonso Vallejos-Parás, PhD**
Instituto Mexicano del Seguro Social

Mexico City, Mexico

**Maria Van Kerkhove, PhD**
WHO/WHE/GIH Unit head Emerging and Zoonotic Diseases
World Health Organization
Geneva, Switzerland

**Luis Angel Villar, MD, PhD**
Facultad de Salud Carrera
Universidad Industrial de Santander
Santander, Colombia

**Carmen Viñuela Benéitez, MD**
Department of Obstetrics and Gynecology.
Gregorio Marañón University Hospital School of Medicine
Complutense University.
Madrid, Spain

**Manon Vouga, MD, PhD**
Department Woman-Mother-Child
Lausanne University Hospital,
Lausanne, Switzerland

**Randall Waechter, PhD**
Department of Neuroscience, Physiology, & Behavioral Science
School of Medicine, St. George's University
St. George, Grenada

**Yinghui Wei, PhD**
Centre for Mathematical Sciences
School of Engineering, Computing and Mathematics
University of Plymouth
Plymouth, UK

**Jamie Westcott, MS**
Section of Pediatric Nutrition
University of Colorado School of Medicine
Aurora, CO, USA

**Marc-Alain Widdowson, VetMB, MA, MSc**
Division of Global Health Protection
Centers for Disease Control and Prevention
Nairobi, Kenya

**George SH Yeo, MD**
Division of Obstetrics and Gynecology
KK Women's and Children's Hospital
Singapore

**Ricardo Arraes de Alencar Ximenes, MD, PhD**
Department of Tropical Medicine
Federal University of Pernambuco
Recife, Brazil
