# Supplementary material for: Heterogeneity of Zika virus exposure and outcome ascertainment across cohorts of pregnant women, their infants and their children: a metadata survey
Source: BMJ Open. 2022 Nov 22;12(11):e064362. doi: 10.1136/bmjopen-2022-064362 (PMC9685007; doi:10.1136/bmjopen-2022-064362)
Supplement: Author's manuscript [file bmjopen-2022-064362.draft_revisions.pdf]

BMJ Open

BMJ Open is committed to open peer review. As part of this commitment we make the peer review history of every article we publish publicly available.

When an article is published we post the peer reviewers' comments and the authors' responses online. We also post the versions of the paper that were used during peer review. These are the versions that the peer review comments apply to.

The versions of the paper that follow are the versions that were submitted during the peer review process. They are not the versions of record or the final published versions. They should not be cited or distributed as the published version of this manuscript.

BMJ Open is an open access journal and the full, final, typeset and author-corrected version of record of the manuscript is available on our site with no access controls, subscription charges or pay-per-view fees (http://bmjopen.bmj.com).

If you have any questions on BMJ Open's open peer review process please email info.bmjopen@bmj.com

BMJ Open

Heterogeneity of Zika Virus Exposure and Outcome Ascertainment Across Cohorts of Pregnant Women, their Infants, and their Children: A Metadata Survey

Journal:	*BMJ Open*
Manuscript ID	bmjopen-2022-064362
Article Type:	Original research
Date Submitted by the Author:	03-May-2022

Complete List of Authors:	Carabali, Mabel; Universite de Montreal, Médecine Sociale et Préventive ; University of Toronto Dalla Lana School of Public Health, Epidemiology<br>Maxwell, Lauren; World Health Organization, Department of Sexual and Reproductive Health and Research; UniversitätsKlinikum Heidelberg<br>Levis, Brooke; Keele University, Centre for Prognosis Research, School of Primary, Community and Social Care<br>Shreedhar, Priya; UniversitätsKlinikum Heidelberg

Keywords:	Epidemiology < TROPICAL MEDICINE, Public health < INFECTIOUS DISEASES, INFECTIOUS DISEASES

SCHOLARONE™
Manuscripts

I, the Submitting Author has the right to grant and does grant on behalf of all authors of the Work (as defined in the below author licence), an exclusive licence and/or a non-exclusive licence for contributions from authors who are: i) UK Crown employees; ii) where BMJ has agreed a CC-BY licence shall apply, and/or iii) in accordance with the terms applicable for US Federal Government officers or employees acting as part of their official duties; on a worldwide, perpetual, irrevocable, royalty-free basis to BMJ Publishing Group Ltd ("BMJ") its licensees and where the relevant Journal is co-owned by BMJ to the co-owners of the Journal, to publish the Work in this journal and any other BMJ products and to exploit all rights, as set out in our licence.

The Submitting Author accepts and understands that any supply made under these terms is made by BMJ to the Submitting Author unless you are acting as an employee on behalf of your employer or a postgraduate student of an affiliated institution which is paying any applicable article publishing charge ("APC") for Open Access articles. Where the Submitting Author wishes to make the Work available on an Open Access basis (and intends to pay the relevant APC), the terms of reuse of such Open Access shall be governed by a Creative Commons licence – details of these licences and which Creative Commons licence will apply to this Work are set out in our licence referred to above.

Other than as permitted in any relevant BMJ Author's Self Archiving Policies, I confirm this Work has not been accepted for publication elsewhere, is not being considered for publication elsewhere and does not duplicate material already published. I confirm all authors consent to publication of this Work and authorise the granting of this licence.

For peer review only

Heterogeneity of Zika Virus Exposure and Outcome Ascertainment Across Cohorts of Pregnant Women, their Infants, and their Children: A Metadata Survey

Authors: Mabel Carabali[1, 2], Lauren Maxwell[3, 4], Brooke Levis[5, 6], Priya Shreedhar[4], on behalf of the ZIKV IPD consortium members.

Affiliations

1. Département de Médecine Sociale et Préventive, École de Santé Publique, Université de Montréal, Montreal, Quebec, Canada.
2. Department of Epidemiology, Dalla Lana School of Public Health, University of Toronto. Toronto, Ontario, Canada.
3. Development and Research Training in Human Reproduction, Department of Sexual and Reproductive Health and Research, World Health Organization, Geneve, Switzerland.
4. Heidelberger Institut für Global Health, Universitätsklinikum Heidelberg, Heidelberg, Germany.
5. Centre for Clinical Epidemiology, Lady Davis Institute of Medical Research, Montreal, Quebec, Canada.
6. Centre for Prognosis Research, School of Medicine, Keele University, Staffordshire, United Kingdom.

Corresponding author:

Dr. Mabel Carabali: mabel.carabali@utoronto.ca

Département de Médecine Sociale et Préventive, École de Santé Publique, Université de Montréal

7101 Avenue du Parc, Bureau/Office: 3123, Montréal (QC), H3N 1X9

Tel: 514 343 6111 Ext 44021

For peer review only

The ZIKV-IPD-MA Consortium: Pablo Aguilar Ticona, Luiz Carlos Junior Alcantara, Jackeline Alger, Celia Alpuche Aranda, Jonathan Altamirano, Juan Arias, Lumumba Arriaga-Nieto, Melissa A. G. Avelino, Angel Balmaseda, Azucena Bardají, Carlos Hernan Becerra Mojica, Mónica Benavides, A.P. Bertozzi, Karen Blackmon, Victor Hugo Borja Aburto, *Patrícia Brasil, William J Britt,* Nathalie Broutet, Pierre Buekens, André Cabié, David Alejandro Cabrera-Gaytán, Rodrigo Cachay Figueroa, María Luisa Cafferata, Juan Ignacio Calcagno, Juan P. Calle, Mabel Carabali, Derrick WS Chan, Celia CD Christie, Federico Costa, Antonio Jose Cunha, Carlos Cure, Johanna Antonia Adriana Damen, Marcela C. Daza, Roberta L. DeBiasi, Thomas Debray, Valentijn M.T. de Jong, Camille Delgado López, Leah deWilde, Alan Oliveira Duarte, Geraldo Duarte, Valorie Eckert, Esther M. Ellis, Andres Espinosa-Bode, Cassia Fernanda Estofolete, Roberta Evans, Valéria Christina de Rezende Féres, Fabíola Fiaccadori, Lester Fernando Figueroa Bolaños, Olivier Fléchelles, Arnaud Fontanet, Victoria Fumadó, Anna L. Funk, Anna Gajewski, Rosa Margarita Gelvez Ramirez, Carlo Giaquinto, Luz Gibbons, Suzanne M. Gilboa, Maria Barbara Franco Gomes, Anna Goncé, Cesar R Gonzalez-Bonilla, Eduardo Gotuzzo, Concepción Grajales-Muñiz, Rebecca Grant, Elysse Grossi-Soyster, Tahani Hamdan, Eva Harris, Cosme Harrison, Bruno Hoen, Cristina Hofer, Natanael Holband, Ivonne Huerta, Irene Inwani, Thomas Jaenisch, Esaú João, Edna Kara, Salma Khuwaja, Caron Kim, Albert I. Ko, Nancy F. Krebs, Angelle Desiree LaBeaud, Heather Lake-Burger, Ellen H. Lee, Vernon Lee, Yee-Sin Leo, Brooke Levis, Eduardo Lopez-Medina, Anyela Lozano-Parra, Elena Marbán-Castro, Celina Maria Turchi Martelli, Lauren Maxwell, Clara Menéndez, Marcela Mercado Reyes, Conrado Milani Coutinho, María Consuelo Miranda Montoya, Demócrito de Barros Miranda-Filho, Maria Elisabeth Moreira, J. Glenn Morris, Jr., Sarah B. Mulkey, Johanna Munoz, José Esteban Muñoz-Medina, Peninah Munyua, Marisa Marcia Mussi-Pinhata, Nivison Nery Jr., M. Kariuki Njenga, Mauricio L. Nogueira, Eric Osoro, Miguel Parra-Saavedra, Saulo Passos, Bernadete Perez Coêlho, Monika Piccardi, Léo Pomar, Arnaldo Prata-Barbosa, Ingrid Rabe, Mitermayer Reis, Hannah Rettler, Megan R. Reynolds, Ana Maria Rivera Casas, Diana Patricia Rojas, Teresita Rojas-Mendoza, Nicole Roth, Paola Mariela Saba Villarroel, Magda Sanz Cortes, Janet L. Sayers, Deolinda Scalabrin, Lavinia Schuler-Faccini, Stacey Schultz-Cherry, Kirstin Short, Priya Shreedhar, Antônio Augusto Silva, Ronaldo Silva, Isadora Siqueira, Karen Sohan, Carmen Soria-Segarra, Antoni Soriano-Arandes, Patrícia da Silva Sousa, Maria Benamor Teixeira, Claire Thorne, Soe Soe Thwin, Van T. Tong, Benoit Tressieres, Marília Dalva Turchi, Diana Valencia, Miguel Valencia-Prado, Alfonso Vallejos-Parás, Maria Van Kerkhove, Luis Angel Villar, Carmen Viñuela Benéitez, Manon Vouga, Randall Waechter, Yinghui Wei, Jamie Westcott, Marc-Alain Widdowson, George SH Yeo, Ricardo Arraes de Alencar Ximenes. The full list of participants of the ZIKV-IPD-MA Consortium members is attached as supplementary material.

For peer review only - http://bmjopen.bmj.com/site/about/guidelines.xhtml

ABSTRACT (248 words)

Objectives: To support the Zika virus (ZIKV) Individual Participant Data (IPD) Consortium's efforts to harmonize and analyze IPD from ZIK-related prospective cohort studies and surveillance-based studies of pregnant women and their infants and children; we developed and disseminated a metadata survey among ZIKV-IPD meta-analysis (MA) study participants to identify and provide a comprehensive overview of study-level heterogeneity in exposure, outcome, and covariate ascertainment and definitions.

Setting: Cohort and surveillance studies that measured ZIKV infection during pregnancy or at birth and measured fetal, infant, or child outcomes were identified through a systematic search and consultations with ZIKV researchers and Ministries of Health from 20 countries or territories.

Participants: Fifty-four cohort or active surveillance studies shared de-identified data for the IPD-MA and completed the metadata survey, representing 33,061 women (11,020 with ZIKV) and 18,281 children.

Primary and secondary outcome measures: Study-level heterogeneity in exposure, outcome, and covariate ascertainment and definitions.

Results: Median study sample size was 268 (Interquartile range (IQR)=100, 698). Inclusion criteria, follow-up procedures, and exposure and outcome ascertainment were highly heterogenous, differing meaningfully across regions and multisite studies. Enrollment duration and follow-up for children after birth varied before and after the declaration of the Public Health Emergency of International Concern (PHEIC) and according to the type of funding received.

Conclusion: This work highlights the logistic and statistical challenges that must be addressed to account for the multiple sources of within- and between-study heterogeneity when conducting IPD-MAs of data collected in the research response to emergent pathogens like ZIKV.

For peer review only - http://bmjopen.bmj.com/site/about/guidelines.xhtml

Strengths and limitations

- We document the presence of cross- and within study-level heterogeneity across 54 different ZIKV-IPD-MA participant studies from 20 countries or territories. By the time of submission, our study includes one of the largest sample of women (n=33,061) and ZIKV confirmed cases (n=11,020).

- Study and laboratory protocols changed substantially over time, as knowledge about ZIKV evolved, resulting in important heterogeneity across studies. The in-depth evaluation of the cross-study heterogeneity in exposure, outcome, and covariate ascertainment and definitions, elucidate logistic and methodological challenges inherent to the conduction of IPD-MA of emergent pathogens.

- The level of heterogeneity across studies underlines the urgent need to develop statistical methods that account properly for the measurement error in both exposure and outcome ascertainment in the context of an IPD-MA of an emergent pathogen.

- This assessment could be used to appropriately separate measurement- versus clinically relevant sources of heterogeneity in the ZIKV IPD-MA.

- This appraisal can be used as a template for the transparent and comprehensive treatment of different sources of bias when harmonizing and analyzing IPD collected in the research response to other emerging pathogens.

For peer review only

INTRODUCTION

In 2016, the sharp surge in microcephaly incidence led to the declaration of Zika virus (ZIKV) as a public health emergency of international concern (PHEIC). Five years later, our understanding of the short and long-term effects of ZIKV exposure *in utero* is still incomplete[1-4]. ZIKV is principally transmitted by *Aedes* mosquitoes and can also be transmitted through unprotected sexual intercourse with a ZIKV-infected partner[5,6]. To date, 90 countries have reported autochthonous ZIKV transmission[7].

Diagnosis of ZIKV infection is challenging, expensive, and time consuming, especially in endemic areas where cross-reactivity and co-circulating arboviruses are important concerns[8-14]. Most ZIKV infections (75-85%) are asymptomatic.[1,5-7] If symptoms arise, the presence of conjunctivitis and rash are distinctive ZIKV characteristics from other self-limited febrile arboviral infections (e.g., dengue virus (DENV), chikungunya (CHIKV))[6,15,16]. The confirmation of ZIKV includes ruling out the presence of DENV and other arboviruses, and confirmation via real-time or standard Reverse Transcription Polymerase Chain Reaction (RT-PCR) or plaque reduction neutralization test (PRNT)[9]. The performance of both immunological and molecular ZIKV tests differs according to the sensitivity and specificity of the assay, the characteristics of the sample (e.g., sample type, sample maintenance), the timing between symptom onset and sample collection and between sample collection and testing, and the implementation and adherence to laboratory quality control (QC) procedures[8-10,17].

ZIKV infections during pregnancy have been associated with severe outcomes including fetal loss and congenital Zika syndrome (CZS), a spectrum of clinical conditions including central nervous system-related congenital malformations (e.g., microcephaly) as well as ophthalmologic and musculoskeletal abnormalities[1,3]. American and Asian ZIKV strains have been causally related to fetal and postnatal onset microcephaly and CZS; recent nonhuman experimental studies describe higher than expected fetal pathogenicity among recent ZIKV African strains[18]. There is no specific treatment for ZIKV other than management of symptoms and no ZIKV vaccine has yet been approved for use[6,7,19,20]. At the country level, ZIKV-related healthcare needs and employment loss cost an estimated USD 2.3 billion per year and caused average yearly loss of over 44,000 disability-adjusted life years (DALYs) [21-24].

The ZIKV Individual Participant Data (IPD) Consortium was created in 2017 to bring together ZIKV-focused clinical and social science researchers and other stakeholders to conduct an IPD meta-analysis (IPD-MA)[2,25] of ZIKV-related cohorts and surveillance systems that measured ZIKV exposure during pregnancy or at birth. By harmonizing existing IPD across related studies, the consortium will leverage

For peer review only - http://bmjopen.bmj.com/site/about/guidelines.xhtml

existing data to develop and evaluate prognostic models to inform health care providers, pregnant women, and couples planning a pregnancy, and to improve prevention and control measures through identifying groups at the highest risk of adverse consequences during future outbreaks. In addition to priorities for improved diagnosis, management, and prevention of ZIKV infection described in the ZIKV Research and Development Roadmap[26], the ZIKV IPD Consortium's research priorities are to: 1) identify modifiers of the relation between ZIKV infection during pregnancy and adverse short and long term fetal, infant, and child outcomes, 2) assess the role of measurement error and other sources of bias in study estimates, and 3) assess the long-term effects of congenital exposure for children with and without clinical or laboratory signs of congenital infection at birth, with consistent definitions of CZS[2]. The ZIKV-IPD Consortium IPD-MA currently includes 65 cohorts and local surveillance sites from 24 countries and territories in the Americas, Asia, Europe, and Africa[2].

The pooled analysis of retrospectively harmonized IPD from longitudinal studies in the ZIKV IPD-MA is complicated by several logistic and analytic challenges. Cross- and within-study assessment of the risk of adverse fetal, infant, and child ZIKV-related outcomes has been limited by: heterogeneity in study design, selection bias, differential ascertainment of important confounders or mediators, limitations in ascertainment of exposure (e.g., timely diagnosis or incomplete follow-up) and outcomes (e.g., pregnancy losses or terminations), and lack of standardization in definitions across studies, which were launched mostly during the acute phase of the ZIKV outbreak in 2015-2016[1-3,7,11]. Therefore, well characterized metadata are essential to appropriately account for study-level sources of heterogeneity in outcome or imputation models and to further improve the precision of subject-level inference in IPD-MA studies.

To support the ZIKV IPD Consortium's efforts to harmonize and analyze IPD from ZIKV- related prospective cohort studies and surveillance-based studies of pregnant women and their infants and children, we developed and disseminated a survey of study-level characteristics (i.e., metadata). Here we present the results from the ZIKV-IPD metadata survey to provide a comprehensive overview of study-level sources of heterogeneity related to IPD datasets that have or will be made available for the IPD-MA. The in-depth evaluation of cross-study heterogeneity in exposure, outcome, and covariate ascertainment and definitions will be used to appropriately separate measurement- versus clinically derived sources of heterogeneity in the ZIKV IPD-MA. This comprehensive metadata review provides an outlook of different sources of bias and heterogeneity in the analysis of ZIKV-related studies and serves as a template for future work to harmonize and analyze IPD collected in the research response to emerging pathogens, where changes to best practice, definitions, and study and laboratory protocols proscribe the use of traditional metadata extraction.

For peer review only - http://bmjopen.bmj.com/site/about/guidelines.xhtml

METHODS

Study selection

Eligible studies to participate in the IPD-MA and corresponding metadata survey were identified through a systematic search registered on PROSPERO (CRD42017068915) that was subsequently published elsewhere[2,25]. The systematic search, developed in consultation with an information scientist and initially conducted on July 8, 2018, applied MeSH and text terms to the Ovid (Medline and Embase) without date, geography, or language-related restrictions. Additional studies were identified through consultation with subject matter experts, Ministries of Health (MoH) and regional health authorities (WHO, PAHO), and through a monthly PubMed alert that applied the following text terms: Zika[Title] AND protocol[Title/Abstract] and Zika[Title] AND cohort[Title]. Longitudinal observational studies or local surveillance systems that (1) conducted laboratory testing for ZIKV during pregnancy or at birth; (2) measured fetal, infant, and/or child outcomes; and (3) included at least 10 individuals (women or children) were eligible. Details of the systematic search to identify eligible studies, including the search strategy, are presented in Appendix 1, Figure S1. Eligible studies were invited via e-mail to share data and to actively participate in the development and completion of the IPD-MA.

Metadata survey development and dissemination

To obtain key metadata information, specifically about procedures that could have potentially changed after studies' implementation, and to obtain information directly from investigators instead of relying on information included in published studies or protocols, we developed a comprehensive metadata survey of completed and ongoing ZIKV-related studies (Appendix 2). We programmed the survey using Research Electronic Data Capture (REDCap)[27] software and disseminated the survey to sites that agreed to share participant-level data for the IPD-MA. The metadata survey included 964 items related to study location, design, population, enrollment criteria, and sample size; maternal, placental, fetal, and infant ascertainment of ZIKV; ascertainment of microcephaly and CZS (clinical and laboratory criteria), other arboviruses, and STORCH pathogens; genetic testing, child development assessment, funding, and duration and timing of follow-up contacts. ZIKV IPD Consortium members formed expert panels that reviewed the exposure and outcome-related components of the metadata survey to ensure its comprehensiveness and utility to potential end users (e.g., other researchers, policy makers, and health care practitioners). The metadata survey was circulated to ZIKV IPD Consortium members in June of 2020 and closed to new responses in March 2021. Investigators received two monthly reminders to complete the survey and were contacted individually to resolve study-level missingness and to address inconsistencies between survey responses and published study reports.

For peer review only - http://bmjopen.bmj.com/site/about/guidelines.xhtml

Data analysis

Overall design, recruitment, exposure, outcome, and covariate ascertainment variables included in the resultant metadata dataset were described using median and interquartile range (IQR) for continuous variables and proportions for categorical variables using RStudio Team (2020; R version 4·0·5). The metadata were assessed for cross- and within-study heterogeneity in recruitment, exposure, outcome and covariate ascertainment and definitions. ZIKV ascertainment practices were assessed by the type (e.g., serological, or molecular testing), timing of the ascertainment (e.g., antenatal, perinatal, and postnatal), and type or source of guidelines used. We conducted a *post hoc* qualitative bias assessment that describes potential issues with design and exposure and outcome ascertainment.

Patient and Public Involvement statement

This study assesses already collected data from ongoing or already finished ZIKV- research studies conducted during the ZIKV epidemic and PHEIC. Our unit of analysis are studies, not individuals. For this study, we did not involve patients in the development of research questions or outcome measurement. However, the ZIKV-IPD-MA consortium aims to leverage existing data to develop and evaluate prognostic models to inform pregnant women and couples planning a pregnancy, and to improve prevention and control measures through identifying groups at the highest risk of adverse consequences during future outbreaks. The consortium is in constant communication with Principal Investigators (PIs), Co-PIS, and site coordinators of the participant studies. Each participant study has a study-specific plan to disseminate their results. IPD-MA research results are disseminated among PIS, Co-PIs a coordinator of the participant studies as part of the already stablished different monthly working group meetings and yearly consortium meetings.

Role of the funding source

The systematic review and metadata survey were drafted and revised with funding support provided by the Wellcome Trust to the WHO Department of Sexual and Reproductive Health and Research Human Reproduction Program, grant numbers 206532/Z/17/Z and 216002/Z/19/Z, respectively. The funder of the study had no role in study design, data collection, data analysis, data interpretation, or writing of the report.

RESULTS

From 160 eligible publications identified through the systematic search and 145 studies identified through consultation, 63 cohort study or surveillance sites, representing 59 unique datasets agreed to

For peer review only - http://bmjopen.bmj.com/site/about/guidelines.xhtml

share de-identified, participant-level data for the ZIKV IPD-MA (Figure 1A). The metadata survey was completed for 54 of these datasets (91·5%). Remaining studies did not initiate or complete the metadata survey after nine months of regular follow-up. The difference between the number of study sites and unique datasets is explained by the inclusion of studies using data from a single surveillance system or multisite studies for which the metadata (e.g., source population, ZIKV assays, and outcome ascertainment) did not vary across sites. For instance, the NIH/NIAID-funded International Prospective Observational Cohort Study of Zika in Infants and Pregnancy (ZIP) study[28] and the European Commission Horizon 2020-funded ZIKAlliance study[29,30] each standardized outcomes definitions and laboratory procedures across sites, but due to specific differences in the source population or follow-up procedures these studies are analyzed individually by study site here. In contrast, the United States Zika Pregnancy and Infant Registry (USZPR)[31,32] is an enhanced surveillance program including 50 states and other US territories with a shared standardized protocol contributing to a single metadata dataset. The Puerto Rico Zika Active Pregnancy Surveillance System (PR-ZAPSS) study[33], part of the overall US Zika registry program, has a slightly different protocol and is included as a separate dataset.

Recruitment

Table 1 presents basic characteristics of participating studies by geography. The 54 studies that completed the metadata survey included 33,061 women (non-pregnant, pregnant, and puerperal), 11,030 confirmed maternal ZIKV cases, and 18,281 infants and children from 20 countries or territories. Details presented in Table S1. Twenty-three studies (43%) were conducted in Brazil (Figure 1B). Thirty-seven studies (68%) recruited participants from healthcare facilities and three studies included travelers returning from endemic countries. Fifty studies enrolled pregnant and or puerperal women, three studies enrolled infants at birth and collected retrospective information on ZIKV symptoms during pregnancy, and one study included women of reproductive age regardless of pregnancy status. Figure 2 shows the duration of enrollment and infant and child follow-up by type of funding and Figure 3 shows the study size, enrollment start date and ZIKV positivity by study design. Duration of enrollment ranged from 1 to 60 months (median=18; IQR=11, 28 months) (Table 1). The most frequent study design was prospective cohort (83%). Surveillance studies (15%), with recruitment based on clinical or laboratory criteria (e.g., PCR or immunoassays), were by default case-only studies and often included larger sample sizes. Studies that limited enrollment to ZIKV positive women (32%) or had a higher percentage of ZIKV positive women often started enrollment before the declaration of the PHEIC in 2016 (Figure 3). Self-funded studies (n=7) and studies receiving local or national funding (n=12) generally initiated enrollment before the declaration of the PHEIC while studies receiving international funding mostly started enrolment months after (Figure 2).

For peer review only - http://bmjopen.bmj.com/site/about/guidelines.xhtml

Table 1. Summary table of study-level characteristics among participants of the ZIKV-IPD-MA metadata survey

Characteristic	Overall N = 54[1]	Study's Origin: Brazil N = 23 (43%)	Study's Origin: Other Countries N = 31(57%)
**No. Pregnant women**			
Median (IQR)	268 (100, 698)	178 (98, 522)	436 (113, 1,108)
**No. ZIKV (+)**			
Median (IQR)	68 (18, 182)	66 (47, 150)	72 (16, 182)
**% ZIKV (+) per study**			
Median (IQR)	47 (3, 100)	68 (7, 100)	36 (2, 100)
**No. Children**			
Median (IQR)	154 (54, 487)	130 (54, 357)	220 (52, 616)
**Study design**			
Case cohort	1 (2%)	1 (4%)	0 (0%)
Cohort	45 (83%)	20 (87%)	25 (81%)
Surveillance	8 (15%)	2 (9%)	6 (19%)
**Source population**			
Community	6 (11%)	4 (17%)	2 (6.6%)
Community & healthcare facility	8 (15%)	6 (26%)	2 (6.6%)
Community + travelers	2 (4%)	0 (0%)	2 (6.6%)
Healthcare facility	37 (68%)	13 (57%)	24 (77%)
Travelers	1 (2%)	0 (0%)	1 (3.2%)
**Enrollment criteria**			
Children only	3 (5%)	3 (13%)	0 (0%)
Not pregnant	1 (2%)	1 (4%)	0 (0%)
Pregnant and/or puerperal women	50 (93%)	19 (83%)	31 (100%)
**Inclusion limited to ZIKV(+) pregnant women**	17 (32%)	6 (26%)	11 (37%)
**Duration of enrollment (months)**			
Median (IQR)	18 (11, 28)	18 (11, 27)	18 (11, 27)
**Duration of follow-up (months)**			
Median (IQR)	24 (15, 29)	24 (12, 36)	24 (18, 24)
**Received funding**	47 (87%)	20 (87%)	27 (87%)
**Type of Funding Received**			
International funding	22 (41%)	4 (17%)	18 (58%)
Multiple sources	13 (24%)	10 (43%)	3 (9.7%)
National/local funding	12 (22%)	6 (26%)	6 (19%)
No/self-funding	7 (13%)	3 (13%)	4 (13%)
**Maternal ZIKV assessment**	51 (94%)	20 (87%)	31 (100%)
**Maternal immunoassay**	35/51 (69%)	13/20 (65%)	19/31 (61%)
**Maternal PCR**	48/51 (94%)	17/20 (85%)	31 (100%)
**Maternal PRNT**	12/51 (24%)	5/20 (25%)	7/31(23%)
**DENV testing**	35/51 (69%)	15/20 (75%)	20/31 (64%)
**CHIKV testing**	31/51 (61%)	14/20 (70%)	17/31 (61%)
**HIV testing**	44/52 (85%)	19/23(83%)	25/29 (86%)
**STORCH testing**	40/51 (78%)	19/23 (83%)	21/28 (75%)
**Fetal ZIKV assessment**	27/53 (51%)	10/22 (45%)	17/31 (55%)
**Infant ZIKV assessment**	41/53 (77%)	17/22 (77%)	24/31 (77%)
**Prenatal microcephaly assessment**	40 (74%)	16 (70%)	24 (77%)
**Pre & postnatal microcephaly assessment**	51 (94%)	23 (100%)	28 (90%)
**Fetal ultrasounds**	39/53 (74%)	15/23 (65%)	24/30 (80%)
**Postnatal ultrasound**	36/53 (68%)	13/23 (57%)	23/30 (77%)

		Study's Origin	
**Characteristic**	**Overall N = 54[1]**	**Brazil N = 23 (43%)**	**Other Countries N = 31(57%)**
**Fetal MRIs**	10/52 (19%)	3/23 (13%)	7/29 (24%)
**Postnatal MRI**	17/52 (33%)	8/23 (35%)	9/29 (31%)
**CZS assessment**	46 (85%)	22 (96%)	24 (77%)
**CZS identification criteria**			
Study definition	15/46 (33%)	7/22 (32%)	8/24 (33%)
WHO/CDC/MoH	31/46 (67%)	15/22 (68%)	16/24 (67%)
**Genetic screening**	9/53 (17%)	2/23 (8.7%)	7/30 (23%)
**Performed child developmental assessment**	40/53 (75%)	16/22 (73%)	24/31 (77%)
**Documented termination of pregnancy**	30 (55%)	9 (39%)	21(68%)

[1] Total number of studies, unless otherwise specified is (n= 54 for all studies; n=23 for Brazilian studies and n=31 for other countries). Interquartile Range (IQR); Confirmed ZIKV Positive (ZIKV (+); Polymerase Chain Reaction (PCR); plaque reduction neutralization test (PRNT); Dengue Virus (DENV); Chikungunya Virus (CHIKV); Human Immunodeficiency Virus (HIV); STORCH (Syphilis; Toxoplasmosis, Rubella, Cytomegalovirus and Herpes Testing); Magnetic Resonance Imaging (MRI); Congenital Zika Syndrome (CZS); World Health Organization (WHO); Center for Diseases Control (CDC); Ministry of Health (MoH).

Exposure ascertainment

Assessment of ZIKV infection in pregnant or puerperal women, and fetal and infant ascertainment is presented in Table 1 and Figures 4-5. Assessment of maternal ZIKV status (n=51) included laboratory confirmation alone in 38 studies (74%) and clinical definitions plus laboratory confirmation in 13 studies (25%). The median proportion of ZIKV-positive women per study was 47% (IQR=3, 100%). The laboratory assessment of ZIKV in pregnant women included PCR (94%), enzyme-linked immunosorbent assays (ELISA) for immunoglobulin M (IgM) or immunoglobulin G (IgG) (69%), an PRNTs (24%). From the studies performing PCR, 71% reported cycle threshold (Ct) values and 73% reported QC procedures; 74% of studies conducting ELISAs, reported QC procedures. Four surveillance-based studies where the testing was done at local or national surveillance laboratories did not provide details of laboratory techniques or QC procedures. Twenty-seven studies ascertained fetal ZIKV exposure using a combination of maternal and fetal laboratory criteria (e.g., diverse testing of fetal tissues or umbilical cord) and used imaging as supporting ascertainment tool. Forty-one studies ascertained ZIKV in infants using PCRs (80%), ELISAs (61%) and PRNTs (12%). Maternal testing for DENV and CHIKV was conducted in 69% (N=35) and 61% (N=31) studies, respectively (Figure 4).

Outcome ascertainment

Antenatal and postnatal ascertainment of microcephaly was completed in 51 studies (94%). Criteria used to define microcephaly followed national or international standards (i.e., WHO, Intergrowth, or MoH definitions) in 42 studies (81%); 10 studies (19%) used their own study-specific criteria. Congenital ZIKV exposure or CZS was assessed in 46 studies (85%), from which 31 (67%) used

For peer review only

international standard criteria and 15 (33%) study-specific definitions. Fetal ultrasounds were performed in 39 (74%) studies (range: 1 to 12 ultrasounds per pregnant woman) and 10 studies performed fetal MRIs. Postnatal cranial ultrasounds and brain MRIs were performed in 36 and 17 studies, respectively. Termination of the pregnancy was documented in 30 (55%) studies (Table 1). Duration of follow-up (median=24; IQR=15, 29 months), differed by the ZIKV status of pregnant women, microcephaly status of newborns, and funding source (Figure 2).

Infant and child development were assessed in 40 (75%) studies using several different international scales, some of which were adapted to local languages and contexts[34]. The most used scales were the Bayley Scales of Infant Development (BSID) (75%) and the Ages & Stages Questionnaires® Social-Emotional, Second Edition (ASQ®-SE-2) (52%). The age range for developmental assessment varied greatly (median age =24; IQR=18, 30, range = 3, 60 months). Two studies from the same research group reported applying the Warner Initial Developmental Evaluation of Adaptive and Functional Skills (WIDEA) and four the Alberta Infant Motor Scale (AIMS) to provide further information on developmental and motor delays not captured by the ASQ®-SE-2 and BSID[35] (Table S2). Five studies reported planning to follow children beyond age five to assess developmental delays that relate to school performance, using the INTERGROWTH-21st Neurodevelopment Assessment (INTER-NDA) or the neuropsychological tests NEPSY®–II.

Covariate ascertainment

Genetic screening and testing were performed in nine and eight studies, respectively. Figure S2 presents the distribution of genetic testing, maternal STORCH and another pathogen ascertainment. Human Immunodeficiency Virus (HIV) testing was conducted in 44 (81%) studies. Fifty-one studies tested for STORCH pathogens, including syphilis (72%), toxoplasmosis (70%), rubella (59%), Cytomegalovirus (CMV) (57%), and herpes (37%). Four studies tested for malaria. Sociodemographic and behavioral factors measured by studies included education (83%), occupation (62%), marital status (77%), household socioeconomic status (71%), maternal exposure to workplace teratogens (33%), exposure to intimate partner violence (18%), recreational drug use/abuse (82%), smoking (82%), alcohol consumption (80%), and ethnicity (67%) (Table S3). Covariate selection and ascertainment varied by study type (surveillance-based vs cohort), duration, timing related to declaration of PHEIC, location, and funding level and source.

Quality of data and risk of bias Assessment

The metadata dataset included information obtained directly from investigators and substantiated by published reports to ensure reliability and quality of the information. The potential sources of bias

For peer review only - http://bmjopen.bmj.com/site/about/guidelines.xhtml

identified across the participant studies relate to selection bias due to differential selection, as by the presence of symptoms or observable birth defects, into the study and follow-up, and measurement error due to exposure and outcome ascertainment which were differential by the presence of symptoms and by ZIKV assay results. The metadata survey demonstrated that study protocols had changed importantly over the course of study implementation to account for advances in the understanding of ZIKV presentation (e.g., the high percentage of asymptomatic cases), diagnostics (e.g., cross-reactivity with DENV), outcomes (e.g., development of adverse developmental outcomes that were not observed at birth), and to accommodate funding availability. The misclassification of ZIKV infection is foregrounded in our presentation of the diversity of methods, resources, definitions, and classifications applied in studies' ascertainment of maternal, fetal, and infant ZIKV infection (Table 1, Figure 4 and 5, Tables S4-S5).

DISCUSSION

We conducted a comprehensive metadata survey to collect study-level data on exposure, outcome, and covariate ascertainment to ensure the appropriate harmonization and analysis of participant-level data from ZIKV IPD-MA contributing studies. We found a high level of cross- and within-study heterogeneity in both maternal ZIKV exposure and outcome ascertainment and the analysis of the evolving spectrum of congenital malformations and post-natal onset developmental delays that define CZS.

The range and distribution of study-level metadata have important public health and epidemiological implications related to the: 1) accurate communication of individual- and population-level risk, 2) reliable identification of prognostic factors and effect measure modifiers for ZIKV-related outcomes, and 3) urgent need to develop statistical methods that account properly for the measurement error in both exposure and outcome ascertainment in the context of an IPD-MA. Below we discuss the advantages of our approach to metadata collection, key study-level sources of heterogeneity, and the potential implications of our findings for the analysis of completed or ongoing IPD-MAs and the design of future research response to emerging pathogens.

First, in the research response to emerging pathogens, stakeholder consultation is likely more effective than a traditional systematic review for identifying eligible studies and obtaining reliable metadata[36-40]. We initially tried to collect study-level metadata from publications or otherwise available protocols. However, we found important differences in design, definitions and ascertainment procedures between the available documentation and investigator self-reports. The conflicting or incomplete information

For peer review only - http://bmjopen.bmj.com/site/about/guidelines.xhtml

across information sources, especially as related to exposure ascertainment and QC procedures, led us to develop the metadata survey.

Second, our metadata survey identified sources of heterogeneity across and within studies that are not commonly observed in IPD-MAs of well characterized pathogens or in chronic diseases[41-43]. Study and laboratory protocols changed substantially over time, as knowledge about ZIKV evolved, resulting in important heterogeneity across studies[5,9,15]. For example, diagnostic algorithms and testing guidelines evolved from using serology and RT-PCR to include PRNT testing over the course of the epidemic[5,9,14,15,44,45]. The high ZIKV infection attack rates (17-55%) reported during the epidemic across the Americas and the causal link with microcephaly [4,5,17,46-49], prompted a rapid rollout of cohorts of pregnant women. Due to the natural course of the epidemic and to study inclusion criteria, studies initiated around the epidemic peak or immediately after the declaration of PHEIC had higher ZIKV positive rates among pregnant women compared to studies that initiated later during the epidemic. Enrollment (criteria and duration), the follow-up for pregnant women and their children, and the planned methodological approaches varied by the type of funding. Thus, the natural course of the epidemic, changes to understanding of ZIKV etiology, and funding mechanisms[50] impacted the quality and amount of information collected, further contributing to cross-study heterogeneity.

Third, ZIKV diagnosis and confirmation is challenging[3,9-12,14,15,17,45]. Our findings indicate that the diverse criteria, definitions, assays, and testing algorithms used for the ZIKV diagnosis are key sources of heterogeneity in ZIKV infection ascertainment. The main challenges for the confirmation of ZIKV infection in our survey were the 1) lack of standardized or consistent guidelines and algorithms for ZIKV diagnosis; 2) ascertainment of ZIKV infection in settings that are endemic for other arboviruses using assays that do not adequately account for cross-reactivity; and 3) lack of ascertainment of fetal ZIKV infection. Most studies indicated that ZIKV infection among pregnant women was ascertained using both clinical and laboratory criteria. However, the range and scope of the clinical criteria was as diverse as the number of studies and the definition of laboratory confirmation included several combinations of immunoassays and molecular techniques, each one with different standards of procedures (SOPs), variable or unknown cutoff points, and different or unknown approaches for QC. The urgency and limited knowledge about ZIKV called for the simultaneous, asynchronous development of diagnostic tests, where researchers converged over time to a somewhat smaller number of more accurate assays or testing algorithms[8,9,14,15,26,31]. This lack of standardization can be seen as both detrimental, if at early stages of the epidemic the testing was based only on the presence of symptoms or if the methods used were less accurate[11,13-16,31], and beneficial because it led to innovation in test development[9,10,12,15,17,26].

For peer review only - http://bmjopen.bmj.com/site/about/guidelines.xhtml

Fourth, smaller studies that were launched earlier in the epidemic, which were often the less resourced, are potentially affected by an outcome and exposure ascertainment that is differential by the probability of infection, which in turn may influence the estimation of the long- and short-term risk of ZIKV effects. Surveillance-based studies include mostly symptomatic individuals who are able to seek and receive healthcare attention (e.g., diagnosis and standard care)[51]. Given the high proportion of asymptomatic ZIKV-infections[6,7,47,52], some surveillance-based studies are likely affected by underreporting[15,46,52-54]. Neither studies launched early in the epidemic nor surveillance sites included comparable data on women who were not infected with ZIKV during pregnancy. In addition, health seeking behavior for symptomatic individuals depends also on socioeconomic determinants of access to health care[46,53], potentially leading to differential outcome ascertainment, representing another source of bias[17,53,55]. For instance, in endemic areas, the presence of symptoms was related to antenatal healthcare practices (e.g., number of antenatal visits, ZIKV testing, and ultrasounds and medical termination of the pregnancies) during the epidemic[46], leading to potential differential assessment of exposure and outcomes, including for miscarriages, induced and spontaneous fetal losses, and congenital malformations. Additionally, cultural sensitivity and restricted legal environments meant that only half of studies measured termination of pregnancy, which biases results differently across sites depending on abortion access[56]. Furthermore, a wide range of definitions of congenital Zika were implemented. Some studies used the WHO definition of CZS, others a modified version including isolated manifestations like ophthalmological abnormalities, and several the presence of microcephaly at birth to define CZS. Therefore, the few cohort studies with longer follow-up are valuable resources for understanding the longer-term consequences of congenital ZIKV infection among infants that were born without manifestations of congenital ZIKV, because studies with short or no follow-up for children would not have captured potential cognitive or developmental issues.[1,3,34,35]

These four issues pose both statistical and logistical challenges when harmonizing and analyzing data in the context of an IPD-MA of an emerging pathogen. The ZIKV-IPD Consortium recognized that existing methods for addressing the correlated sources of heterogeneity in the research response to ZIKV (e.g., dependency between timing of symptoms, test accuracy, and differential ascertainment for both exposure and outcome, etc.) are inadequate. In response to this gap, the Consortium launched a statistical working group to develop novel approaches to address heterogeneity and measurement error in the context of IPD-MAs of emerging pathogens.

Limitations and Strengths

For peer review only - http://bmjopen.bmj.com/site/about/guidelines.xhtml

The inclusion of ongoing studies presented important challenges in that key aspects of exposure and outcome ascertainment have continued to evolve as studies obtain additional funding to retest samples or to continue and expand follow-up[50]. In some cases, studies changed exposure ascertainment procedures or laboratory QC procedures and were not able to record when changes were made or what had been changed, which represents missing or misclassified metadata, further complicating the assessment of the accuracy of exposure ascertainment[2,9,17]. Although we conducted regular follow-ups over a nine-month period to obtain the information, some researchers did not respond, reasonably because they were on the frontlines of the COVID-19 pandemic response. This impacted the timelines for participant- and metadata acquisition and review for most studies. Nonetheless, the multiple interactions required with the investigators enriched the analytical process, ensured the robustness and quality of the data, and strengthened the cross-consortium communication.

The identification of the above-mentioned sources of heterogeneity constitutes the main strength of this study. This is the first study to assemble a comprehensive reporting of study-level heterogeneity of ZIKV-related cohorts of pregnant women and their infants and children, which could only be gathered through investigators' self-report. The final metadata and participant-level data sets will represent the largest resource of longitudinal, participant-level data on maternal-infant/child dyads for many of the countries where research teams have contributed data for the IPD-MA. Ensuring that participant-level data are accompanied by well characterized metadata, coupled with engaging the teams that collected the data and external subject matter experts, will help the ZIKV IPD Consortium ensure the appropriate estimation of outcome and imputation models. This detailed study-level metadata will facilitate the way that IPD can be harmonized and analyzed, including the identification of study-related sources of heterogeneity and clinically related participant-level sources of heterogeneity[36,37,40]. Given the limited understanding of the distribution of adverse fetal, infant, and child outcomes related to congenital exposures, the meta- and participant-level dataset will represent a tremendous cross- and within-country resource for social, medical, infectious, and other exposures, that goes beyond ZIKV.

Conclusion

This analysis provides a comprehensive overview of study-level heterogeneity related to participant-level data available from ZIKV-related cohorts and surveillance systems of pregnant women and their infants and children. The challenges confronted and addressed here include: 1) the need to consult with researchers and local and international health authorities early in the outbreak to identify related studies; 2) the need to engage directly with investigators to understand and document heterogeneity in study design, definitions, and implementation, which are inconsistently or incompletely reported in the published literature and include information according to the existing knowledge of the problem at that

For peer review only - http://bmjopen.bmj.com/site/about/guidelines.xhtml

given time; and 3) the need to consistently and regularly adjust research targets and timelines to account for continuing changes to study protocols, teams, agendas and funding availability, ensuring the quality of the studies. Our study will serve to inform ongoing and future collaborations that prospectively or retrospectively harmonize IPD and to drive the development of novel statistical methods that can address correlated sources of measurement error in IPD-MA of emerging pathogens.

Contributions

LM, MC, BL were responsible for the study conception and design. LM designed and conducted database searches to identify eligible studies. MC, LM, BL, and PS contributed to data extraction and coding for the metadata analysis. MC, LM, and BL contributed to the data analysis and interpretation. MC and LM wrote and BL, PS contributed to the first draft of the manuscript; ZIKV-IPD Consortium participants contributed to subsequent versions of the manuscript. All authors provided a critical review and approved the final manuscript. LM, MC, BL, PS are the guarantors; they have full access to all the data in the study and take responsibility for the integrity of the data and the accuracy of the data analyses.

Declaration of interests

All authors have completed the ICMJE uniform disclosure form and declare: no support from any organization for the submitted work; no financial relationships with any organizations that might have an interest in the submitted work in the previous three years for the main authors. No funder had any role in the design and conduct of this study; collection, management, analysis, and interpretation of the data; preparation, review, or approval of the manuscript; and decision to submit the manuscript for publication. Consortium and study PIs contributing data to the ZIKV-IPD-MA members have provided declarations of potential or perceived conflict of interests in Appendix 3.

Acknowledgements

The authors would like to acknowledge the valuable contributions of all researchers, fieldworkers, and participants of the contributing studies of the ZIKV-IPD-MA.

Funding

This systematic review and summary of metadata was drafted and revised with funding support provided by the Wellcome Trust to the WHO Department of Reproductive Health and Research Human Reproduction Program, grant numbers 206532/Z/17/Z and 216002/Z/19/Z, respectively. Dr. Levis was supported by a Fonds de Recherche du Québec – Santé Postdoctoral Training Award. No other main authors reported funding for primary studies or for their work on the present study. Funding sources

For peer review only - http://bmjopen.bmj.com/site/about/guidelines.xhtml

for each participating study in the ZIKV-IPD-MA are provided in the supplementary material. The funding source had no role in the drafting of the manuscript.

Data Sharing

Key metadata are presented in the tables in the manuscript and corresponding appendix. Requests for access to the full metadata dataset for the 54 contributing studies should be made to the corresponding author.

Ethics Statement

The IPD-MA protocol was deemed exempt from ethical review by the WHO Ethics Review Committee and the Emory University Institutional Review Board because the IPD-MA uses de-identified, participant-level data from studies that have received ethical approval for the same objectives as the original studies. In keeping with recommendations for the re-use of de-identified participant-level data in an IPD-MA, we conducted a qualitative study to assess source communities' perceptions regarding the reuse of their data for an IPD-MA and preferences for the communication of ZIKV-related risk in the presence of uncertainty in both the diagnosis and the probability of ZIKV-related adverse events, given a positive diagnosis.

Role of the funding source

The study funder had no role in study design, data collection, data analysis, data interpretation, or writing of the report. The corresponding author had full access to all data in the study and final responsibility for the decision to submit for publication.

Disclaimers

World Health Organization: The authors alone are responsible for the views expressed in this article and they do not necessarily represent the views, decisions, or policies of the institutions with which they are affiliated. United States Centers for Disease Control and Prevention: The findings and conclusions in this report are those of the authors and do not represent the official position of the Centers for Disease Control and Prevention (CDC).

For peer review only - http://bmjopen.bmj.com/site/about/guidelines.xhtml

REFERENCES

1. Martins MM, Alves da Cunha AJL, Robaina JR, Raymundo CE, Barbosa AP, Medronho RdA. Fetal, neonatal, and infant outcomes associated with maternal Zika virus infection during pregnancy: A systematic review and meta-analysis. *PLOS ONE* 2021; **16**(2): e0246643.

2. Zika Virus Individual Participant Data C. The Zika Virus Individual Participant Data Consortium: A Global Initiative to Estimate the Effects of Exposure to Zika Virus during Pregnancy on Adverse Fetal, Infant, and Child Health Outcomes. *Tropical medicine and infectious disease* 2020; **5**(4): 152.

3. Freitas DA, Souza-Santos R, Carvalho LMA, et al. Congenital Zika syndrome: A systematic review. *PLoS One* 2020; **15**(12): e0242367.

4. WHO. Emergency Committee on Zika virus and observed increase in neurological disorders and neonatal malformations. Geneva, Switzerland: WHO, 2016.

5. WHO. Zika virus infection: global update on epidemiology and potentially associated clinical manifestations. *Releve epidemiologique hebdomadaire* 2016; **91**(7): 73-81.

6. Alvarado MG, Schwartz DA. Zika Virus Infection in Pregnancy, Microcephaly, and Maternal and Fetal Health: What We Think, What We Know, and What We Think We Know. *Archives of pathology & laboratory medicine* 2016; **141**(1): 26-32.

7. World Health Organization (WHO). Zika epidemiology update. In: WHO, editor. Health Topics. July 2019 ed. Geneva, Switzerland; 2019. p. https://www.who.int/publications/m/item/zika-epidemiology-update.

8. Waggoner JJ, Gresh L, Vargas MJ, et al. Viremia and Clinical Presentation in Nicaraguan Patients Infected With Zika Virus, Chikungunya Virus, and Dengue Virus. *Clinical Infectious Diseases* 2016; **63**(12): 1584-90.

9. Waggoner JJ, Pinsky BA. Zika Virus: Diagnostics for an Emerging Pandemic Threat. *Journal of Clinical Microbiology* 2016; **54**(4): 860-7.

10. Fischer C, Pedroso C, Mendrone A, Jr., et al. External Quality Assessment for Zika Virus Molecular Diagnostic Testing, Brazil. *Emerging infectious diseases* 2018; **24**(5): 888-92.

11. Fritzell C, Rousset D, Adde A, Kazanji M, Van Kerkhove MD, Flamand C. Current challenges and implications for dengue, chikungunya and Zika seroprevalence studies worldwide: A scoping review. *PLOS Neglected Tropical Diseases* 2018; **12**(7): e0006533.

12. Voermans JJC, Pas SD, van der Linden A, et al. Whole-Blood Testing for Diagnosis of Acute Zika Virus Infections in Routine Diagnostic Setting. *Emerging infectious diseases* 2019; **25**(7): 1394-6.

13. Qiao L, Martelli CMT, Raja AI, et al. Epidemic preparedness: Prenatal Zika virus screening during the next epidemic. *BMJ Global Health* 2021; **6**(6): e005332.

14. Ximenes RAdA, Miranda-Filho DdB, Brickley EB, et al. Zika virus infection in pregnancy: Establishing a case definition for clinical research on pregnant women with rash in an active transmission setting. *PLOS Neglected Tropical Diseases* 2019; **13**(10): e0007763.

15. Pan American Health Organization (PAHO). Tool for the diagnosis and care of patients with suspected arboviral diseases. 1st Edition ed. Washington: PAHO; 2017.

16. Sanchez Clemente N, Brickley EB, Furquim de Almeida M, Witkin SS, Duarte Passos S, Group tJZC. Can Zika Virus Infection in High Risk Pregnant Women Be Differentiated on the Basis of Symptoms? *Viruses* 2020; **12**(11): 1263.

17. Simmons G, Stone M, Busch MP. Arbovirus Diagnostics: From Bad to Worse due to Expanding Dengue Virus Vaccination and Zika Virus Epidemics. *Clinical Infectious Diseases* 2017; **66**(8): 1181-3.

18. Aubry F, Jacobs S, Darmuzey M, et al. Recent African strains of Zika virus display higher transmissibility and fetal pathogenicity than Asian strains. *Nature Communications* 2021; **12**(1): 916.

For peer review only - http://bmjopen.bmj.com/site/about/guidelines.xhtml

19. Pierson TC, Graham BS. Zika Virus: Immunity and Vaccine Development. *Cell* 2016; **167**(3): 625-31.
20. Castanha PMS, Marques ETA. A Glimmer of Hope: Recent Updates and Future Challenges in Zika Vaccine Development. *Viruses* 2020; **12**(12): 1371.
21. Shewale JB, Ganduglia Cazaban CM, Waller DK, Mitchell LE, Langlois PH, Agopian AJ. Microcephaly inpatient hospitalization and potential Zika outbreak in Texas: A cost and predicted economic burden analysis. *Travel Medicine and Infectious Disease* 2019; **30**: 67-72.
22. Lee BY, Alfaro-Murillo JA, Parpia AS, et al. The potential economic burden of Zika in the continental United States. *PLOS Neglected Tropical Diseases* 2017; **11**(4): e0005531.
23. Thompson R, Martin Del Campo J, Constenla D. A review of the economic evidence of Aedes-borne arboviruses and Aedes-borne arboviral disease prevention and control strategies. *Expert Review of Vaccines* 2020; **19**(2): 143-62.
24. Puntasecca CJ, King CH, LaBeaud AD. Measuring the global burden of chikungunya and Zika viruses: A systematic review. *PLOS Neglected Tropical Diseases* 2021; **15**(3): e0009055.
25. Wilder-Smith A, Wei Y, Araújo TVBd, et al. Understanding the relation between Zika virus infection during pregnancy and adverse fetal, infant and child outcomes: a protocol for a systematic review and individual participant data meta-analysis of longitudinal studies of pregnant women and their infants and children. *BMJ Open* 2019; **9**(6): e026092.
26. World Health Organization (WHO). Zika Virus Research and Development (R&D) Roadmap. Geneva, Switzerland: World Health Organization, 2021.
27. Harris PA, Taylor R, Minor BL, et al. The REDCap consortium: Building an international community of software platform partners. *Journal of Biomedical Informatics* 2019; **95**: 103208.
28. Lebov JF, Arias JF, Balmaseda A, et al. International prospective observational cohort study of Zika in infants and pregnancy (ZIP study): study protocol. *BMC Pregnancy Childbirth* 2019; **19**(1): 282-.
29. Avelino-Silva VI, Mayaud P, Tami A, et al. Study protocol for the multicentre cohorts of Zika virus infection in pregnant women, infants, and acute clinical cases in Latin America and the Caribbean: the ZIKAlliance consortium. *BMC Infectious Diseases* 2019; **19**(1): 1081.
30. Ades AE, Brickley EB, Alexander N, et al. Zika virus infection in pregnancy: a protocol for the joint analysis of the prospective cohort studies of the ZIKAlliance, ZikaPLAN and ZIKAction consortia. *BMJ Open* 2020; **10**(12): e035307.
31. Reynolds MR, Jones AM, Petersen EE, et al. Vital Signs: Update on Zika Virus-Associated Birth Defects and Evaluation of All U.S. Infants with Congenital Zika Virus Exposure - U.S. Zika Pregnancy Registry, 2016. *MMWR Morb Mortal Wkly Rep* 2017; **66**(13): 366-73.
32. Center for Disease Control and Prevention (CDC). US Zika Pregnancy and Infant Registry. Zika and Pregnancy Home; Research and Tracking Atlanta, GA: Content source: National Center on Birth Defects and Developmental Disabilities, Centers for Disease Control and Prevention (CDC). 2016. p. https://www.cdc.gov/pregnancy/zika/research/registry.html.
33. Shapiro-Mendoza CK, Rice ME, Galang RR, et al. Pregnancy Outcomes After Maternal Zika Virus Infection During Pregnancy - U.S. Territories, January 1, 2016-April 25, 2017. *MMWR Morb Mortal Wkly Rep* 2017; **66**(23): 615-21.
34. K. Connery A, Berrios-Siervo G, Arroyave P, et al. Responding to the Zika Epidemic: Preparation of a Neurodevelopmental Testing Protocol to Evaluate Young Children in Rural Guatemala. *The American Journal of Tropical Medicine and Hygiene* 2019; **100**(2): 438-44.
35. Mulkey SB, Arroyave-Wessel M, Peyton C, et al. Neurodevelopmental Abnormalities in Children With In Utero Zika Virus Exposure Without Congenital Zika Syndrome. *JAMA Pediatrics* 2020; **174**(3): 269-76.
36. Volk CJ, Lucero Y, Barnas K. Why is Data Sharing in Collaborative Natural Resource Efforts so Hard and What can We Do to Improve it? *Environmental Management* 2014; **53**(5): 883-93.

For peer review only - http://bmjopen.bmj.com/site/about/guidelines.xhtml

37. Samuel S, König-Ries B. Understanding experiments and research practices for reproducibility: an exploratory study. *PeerJ* 2021; **9**: e11140.
38. Lancaster GA, McCray G, Kariger P, et al. Creation of the WHO Indicators of Infant and Young Child Development (IYCD): metadata synthesis across 10 countries. *BMJ Global Health* 2018; **3**(5): e000747.
39. Burkot TR, Farlow R, Min M, Espino E, Mnzava A, Russell TL. A global analysis of National Malaria Control Programme vector surveillance by elimination and control status in 2018. *Malaria Journal* 2019; **18**(1): 399.
40. Levis B, Hattle M, Riley RD. PRIME-IPD SERIES Part 2. Retrieving, checking, and harmonizing data are underappreciated challenges in individual participant data meta-analyses. *Journal of clinical epidemiology* 2021.
41. Salameh J-P, Bossuyt PM, McGrath TA, et al. Preferred reporting items for systematic review and meta-analysis of diagnostic test accuracy studies (PRISMA-DTA): explanation, elaboration, and checklist. *BMJ* 2020; **370**: m2632.
42. Riley RD, Lambert PC, Abo-Zaid G. Meta-analysis of individual participant data: rationale, conduct, and reporting. *BMJ* 2010; **340**: c221.
43. Harris PA, Taylor R, Thielke R, Payne J, Gonzalez N, Conde JG. Research electronic data capture (REDCap)—A metadata-driven methodology and workflow process for providing translational research informatics support. *Journal of Biomedical Informatics* 2009; **42**(2): 377-81.
44. Karwowski MP, Nelson JM, Staples JE, et al. Zika Virus Disease: A CDC Update for Pediatric Health Care Providers. *Pediatrics* 2016; **137**(5).
45. Ribeiro MRC, Khouri R, Sousa PS, et al. Plaque Reduction Neutralization Test (PRNT) in the Congenital Zika Syndrome: Positivity and Associations with Laboratory, Clinical, and Imaging Characteristics. *Viruses* 2020; **12**(11): 1244.
46. Lozier M, Adams L, Febo MF, et al. Incidence of Zika Virus Disease by Age and Sex — Puerto Rico, November 1, 2015–October 20, 2016. *Morbidity and Mortality Weekly Report* 2016; **65**(44): 1219-23.
47. Ellington SR, Devine O, Bertolli J, et al. EStimating the number of pregnant women infected with zika virus and expected infants with microcephaly following the zika virus outbreak in puerto rico, 2016. *JAMA Pediatrics* 2016; **170**(10): 940-5.
48. de Araújo TVB, Ximenes RAA, Miranda-Filho DB, et al. Association between microcephaly, Zika virus infection, and other risk factors in Brazil: final report of a case-control study. *Lancet Infect Dis* 2018; **18**(3): 328-36.
49. Brasil P, Pereira JP, Moreira ME, et al. Zika Virus Infection in Pregnant Women in Rio de Janeiro. *New England Journal of Medicine* 2016; **375**(24): 2321-34.
50. Koopmans M, de Lamballerie X, Jaenisch T, et al. Familiar barriers still unresolved—a perspective on the Zika virus outbreak research response. *The Lancet Infectious Diseases* 2019; **19**(2): e59-e62.
51. Gibbons CL, Mangen M-JJ, Plass D, et al. Measuring underreporting and under-ascertainment in infectious disease datasets: a comparison of methods. *BMC Public Health* 2014; **14**(1): 147.
52. Kucharski AJ, Funk S, Eggo RM, Mallet H-P, Edmunds WJ, Nilles EJ. Transmission Dynamics of Zika Virus in Island Populations: A Modelling Analysis of the 2013–14 French Polynesia Outbreak. *PLOS Neglected Tropical Diseases* 2016; **10**(5): e0004726.
53. Carabali M, Jaramillo-Ramirez GI, Rivera VA, Mina Possu N-J, Restrepo BN, Zinszer K. Assessing the reporting of Dengue, Chikungunya and Zika to the National Surveillance System in Colombia from 2014–2017: A Capture-recapture analysis accounting for misclassification of arboviral diagnostics. *PLOS Neglected Tropical Diseases* 2021; **15**(2): e0009014.

For peer review only - http://bmjopen.bmj.com/site/about/guidelines.xhtml

54. Hernández-Ávila JE, Palacio-Mejía LS, López-Gatell H, et al. Zika virus infection estimates, Mexico. *Bulletin of the World Health Organization* 2018; **96**(5): 306-13.
55. Oliveira Melo AS, Malinger G, Ximenes R, Szejnfeld PO, Alves Sampaio S, Bispo de Filippis AM. Zika virus intrauterine infection causes fetal brain abnormality and microcephaly: tip of the iceberg? *Ultrasound in Obstetrics & Gynecology* 2016; **47**(1): 6-7.
56. Carabali M, Austin N, King NB, Kaufman JS. The Zika epidemic and abortion in Latin America: a scoping review. *Global Health Research and Policy* 2018; **3**(1): 15.

Figure Legends:

Figure 1. Metadata Flowchart (1.A) and ZIKV-Metadata Countries & Territories Participants (1.B). *The difference between the number of study sites and unique datasets is explained by the inclusion of studies using data from a single surveillance system or multisite studies for which the metadata (e.g., source population, ZIKV assays, and outcome ascertainment) did not vary across sites.

Figure 2. Duration of Enrollment and Follow-up for Children by Type of Funding Among Participants of the ZIKV-IPD-MA Metadata Survey

Figure 3. ZIKV Positivity by Study's Start date, Sample Size, and Study Design Among Participants of the ZIKV-IPD-MA Metadata survey

Figure 4. Overview of ZIKV Ascertainment Among Participants of the ZIKV-IPD-MA Metadata Survey

Figure 5. ZIKV Ascertainment and Type of Laboratory Testing and Other Criteria Among Participants of the ZIKV-IPD-MA Metadata Survey

For peer review only - http://bmjopen.bmj.com/site/about/guidelines.xhtml

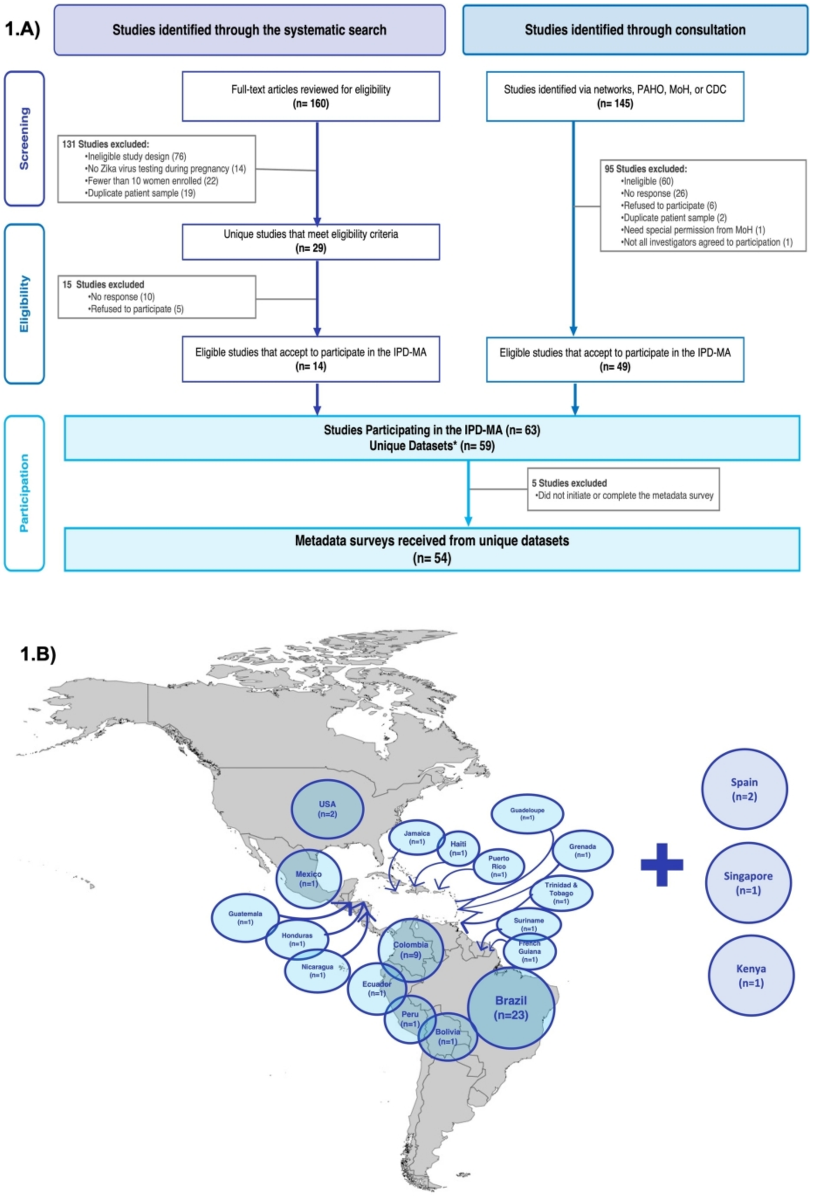

Figure 1. Metadata Flowchart (1.A) and ZIKV-Metadata Countries & Territories Participants (1.B).

179x262mm (300 x 300 DPI)

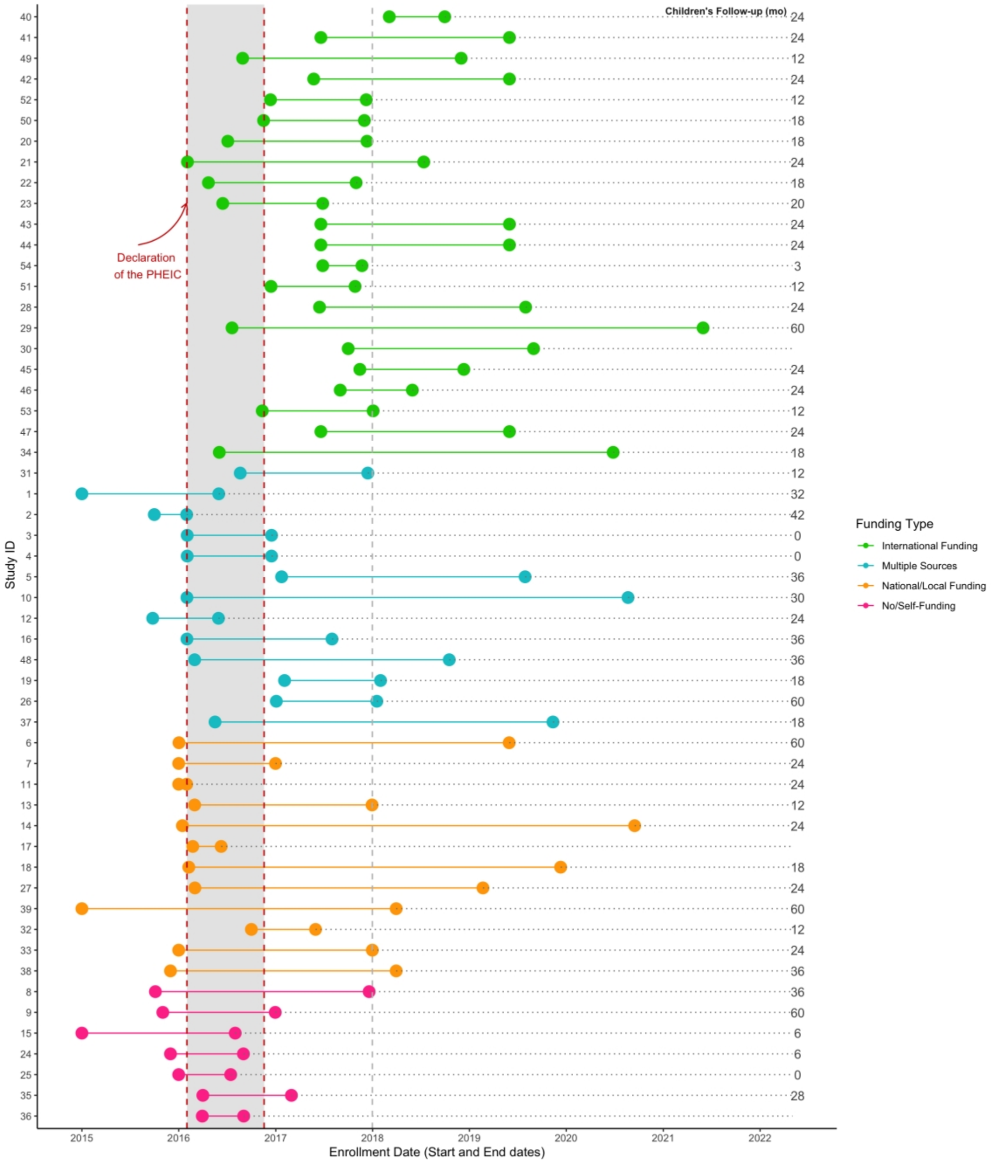

Figure 2. Duration of Enrollment and Follow-up for Children by Type of Funding Among Participants of the ZIKV-IPD-MA Metadata Survey

812x948mm (72 x 72 DPI)

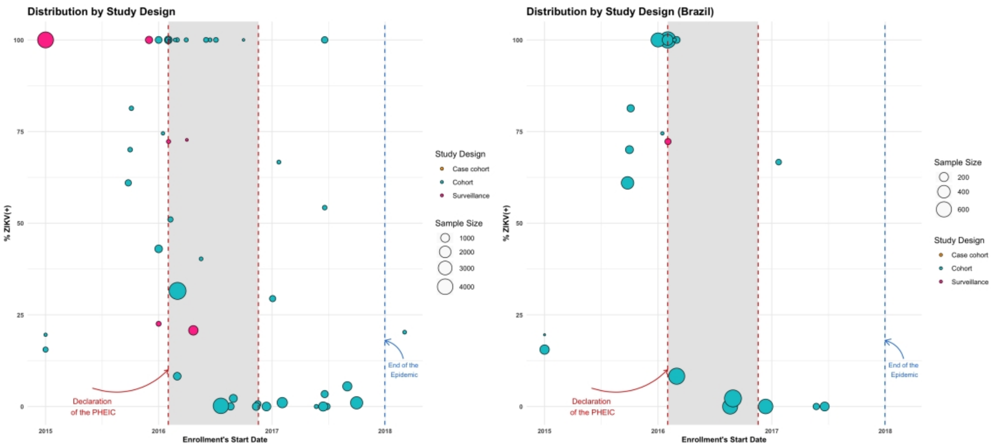

Figure 3. ZIKV Positivity by Study's Start date, Sample Size, and Study Design Among Participants of the ZIKV-IPD-MA Metadata survey

1219x541mm (72 x 72 DPI)

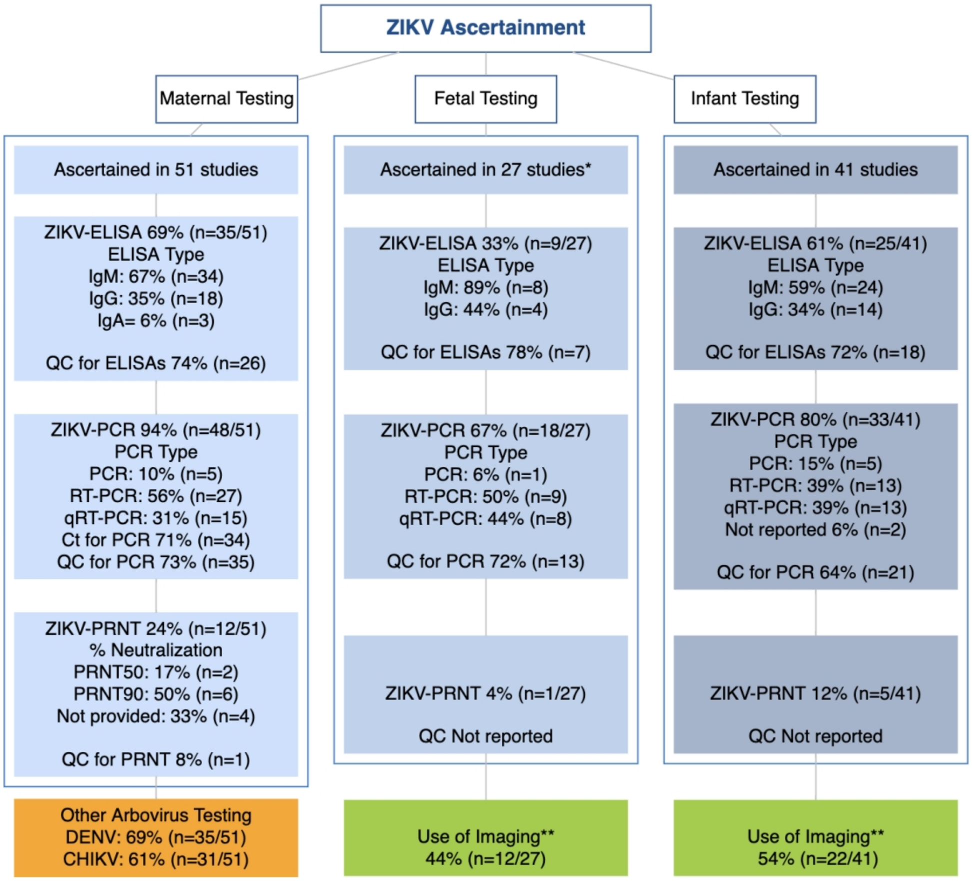

Figure 4. Overview of ZIKV Ascertainment Among Participants of the ZIKV-IPD-MA Metadata Survey

272x249mm (144 x 144 DPI)

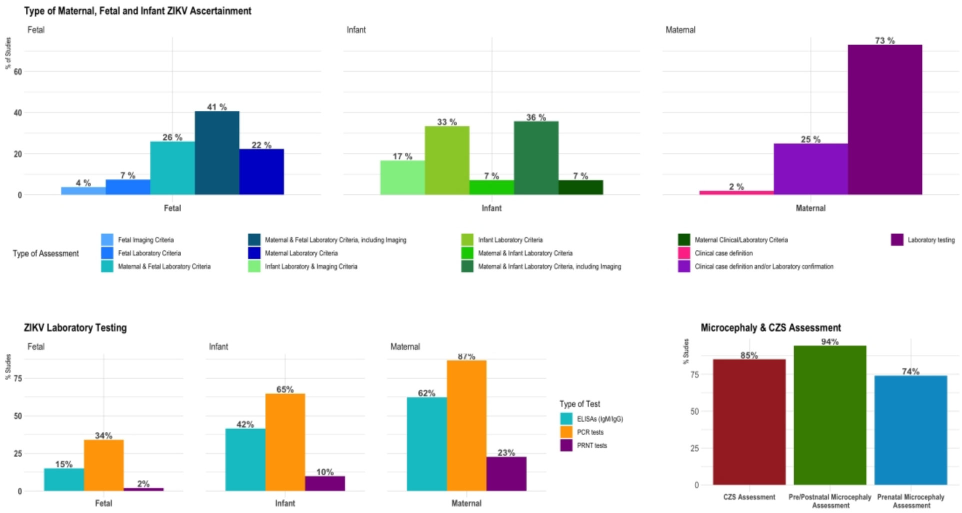

Figure 5. ZIKV Ascertainment and Type of Laboratory Testing and Other Criteria Among Participants of the ZIKV-IPD-MA Metadata Survey

1219x677mm (72 x 72 DPI)

Appendix 1.

Table of Contents

I. **Zika search strategy** ... 2
PICO CRITERIA: ... 2
Zika search, Medline (through Ovid): ... 2
Zika search, Embase (through Ovid): ... 2
II. **Screening, eligibility and selection of studies trhough systematic search** ... 4
Figure S.1. Flowchart for study participation trough systematic search. ... 4
III. **Supplementary tables with descriptive zikv-ipd-ma metada-related statisitcs and figures** ... 5
Table S1. Detailed description of ZIKV-IPD-MA studies participating in the metadata survey (Part 1). ... 5
Table S1. Detailed description of ZIKV-IPD-MA studies participating in the metadata survey (Part 2). ... 12
Table S2. Infant and child development assessment among ZIKV-IPD-MA study participants of the metadata survey... 17
Figure S2. STORCH and other additional testing among ZIKV-IPD-MA study participants of the metadata survey... 17
Table S3. Sociodemographic and behavioral factors measured among ZIKV-IPD-MA study participants of the metadata survey. ... 18
Table S4. Genetic testing, fetal ultrasounds, and MRIs among ZIKV-IPD-MA study participants of the metadata survey ... 19
Table S5a. Type of maternal ZIKV assessment among ZIKV-IPD-MA study participants of the metadata survey. ... 20
Table S5b. Type of fetal ZIKV assessment among ZIKV-IPD-MA study participants of the metadata survey. ... 21
Table S5c. Type of infant ZIKV assessment among ZIKV-IPD-MA study participants of the metadata survey. ... 22

For peer review only

I. ZIKA SEARCH STRATEGY

PICO CRITERIA:

Population	Exposure	Comparator	Outcome (open)
Pregnant women and newborns	ZIKV infection during pregnancy	No ZIKV infection during pregnancy	Primary: microcephaly, miscarriage, fetal loss. Secondary: early/late fetal death, stillbirth, ocular abnormalities, hearing loss, neuroimaging abnormalities, etc.

Date: Started June 12, 2018 – Ended July 8, 2018

Zika search, Medline (through Ovid):

1. exp Zika Virus Infection/ or exp ZIKA VIRUS/
2. (zika or ZIKV).ti,ab,kf.
3. 1 or 2
4. exp Pregnancy/ or exp Maternal Exposure/ or exp "Embryonic and Fetal Development"/ or exp "Congenital, Hereditary, and Neonatal Diseases and Abnormalities"/ or exp Infant/ or exp Child/
5. (pregnan* or matern* or gestation* or perinatal* or birth* or congenital* or newborn* or fetal or fetus* or foetal or foetus* or neonat* or infan* or toddler* or child*).ti,ab,kf.
6. 4 or 5
7. 3 and 6
8. 7 not (exp Animals/ not exp Humans/)

☐	1	exp Zika Virus Infection/ or exp ZIKA VIRUS/	2407
☐	2	(zika or ZIKV).ti,ab,kf.	4672
☐	3	1 or 2	4784
☐	4	exp Pregnancy/ or exp Maternal Exposure/ or exp "Embryonic and Fetal Development"/ or exp "Congenital, Hereditary, and Neonatal Diseases and Abnormalities"/ or exp Infant/ or exp Child/	3661254
☐	5	(pregnan* or matern* or gestation* or perinatal* or birth* or congenital* or newborn* or fetal or fetus* or foetal or foetus* or neonat* or infan* or toddler* or child*).ti,ab,kf.	2574479
☐	6	4 or 5	4373079
☐	7	3 and 6	1947
☐	8	7 not (exp Animals/ not exp Humans/)	1857

Zika search, Embase (through Ovid):

1. exp Zika virus/ or exp Zika fever/
2. (zika or ZIKV).ti,ab,kw.
3. 1 or 2
4. exp pregnancy/ or exp pregnancy outcome/ or exp high risk pregnancy/ or exp pregnancy complication/ or exp maternal exposure/ or exp fetus/ or exp "functions of embryonic, fetal and placental structures"/ or exp Infant/ or exp infant disease/ or exp child/ or exp childhood disease/
5. (pregnan* or matern* or gestation* or perinatal* or birth* or congenital* or newborn* or fetal or fetus* or foetal or foetus* or neonat* or infan* or toddler* or child*).ti,ab,kw.
6. 4 or 5

For peer review only - http://bmjopen.bmj.com/site/about/guidelines.xhtml

7. 3 and 6
8. 7 not ((exp animal/ or exp nonhuman/) not exp human/)

# ▲	Searches	Results
1	exp Zika virus/ or exp Zika fever/	4757
2	(zika or ZIKV).ti,ab,kw.	5183
3	1 or 2	5788
4	exp pregnancy/ or exp pregnancy outcome/ or exp high risk pregnancy/ or exp pregnancy complication/ or exp maternal exposure/ or exp fetus/ or exp "functions of embryonic, fetal and placental structures"/ or exp Infant/ or exp infant disease/ or exp child/ or exp childhood disease/	4556497
5	(pregnan* or matern* or gestation* or perinatal* or birth* or congenital* or newborn* or fetal or fetus* or foetal or foetus* or neonat* or infan* or toddler* or child*).ti,ab,kw.	3374208
6	4 or 5	5355248
7	3 and 6	2675
8	7 not ((exp animal/ or exp nonhuman/) not exp human/)	2326

review only

II. SCREENING, ELIGIBILITY AND SELECTION OF STUDIES TRHOUGH SYSTEMATIC SEARCH

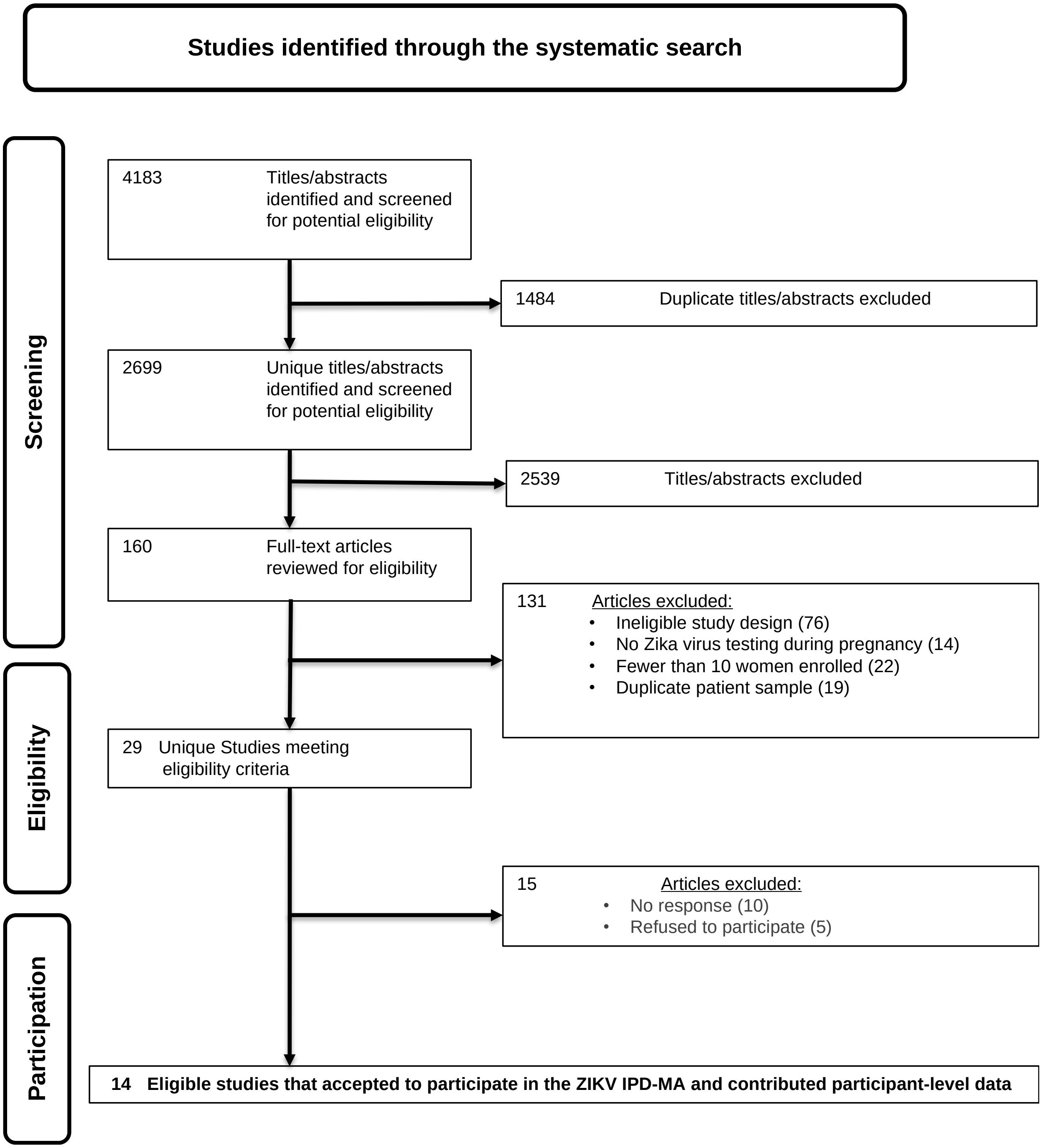

Figure S.1. Flowchart for study participation through systematic search.

For peer review only - http://bmjopen.bmj.com/site/about/guidelines.xhtml

III. SUPPLEMENTARY TABLES WITH DESCRIPTIVE ZIKV-IPD-MA METADA-RELATED STATISITCS AND FIGURES

Table S1. Detailed description of ZIKV-IPD-MA studies participating in the metadata survey (Part 1).

ID	Study Name	Country	Source population	Pregnancy status (Enrollment)	Enrollment and ZIKV status	Study Design	Planned follow-up pregnant women	Planned follow-up child	All Women	ZIKV (+) Women	Children	Funding
1	Bolivia_Roca_ZIKAlliance	Bolivia	Healthcare Facility	Pregnant	Unrelated to ZIKV	Cohort	9 visits conducted every month during pregnancy for both ZIKV+ and ZIKV- women	-	74	15	30	European Union
2	Brazil_Bahia_Ko_NIHNIAD	Brazil	Healthcare Facility	Pregnant	Unrelated to ZIKV	Cohort	9 visits in total for ZIKV+ and ZIKV- pregnant women, every 4 weeks during pregnancy, at delivery, and 6 weeks postpartum. Additional visits were also conducted for women with acute ZIKA-like symptoms.	4 visits at birth, 3, 6, and 12 months of age for infants of both ZIKV+ and ZIKV- pregnant women. The age window for the final visit could be extended up to 18 months.	600	0	509	NIH/NIAID and FIOCRUZ
3	Brazil_BahiaPaudaLima_Costa	Brazil	Community	Pregnant	Unrelated to ZIKV	Cohort	1 visit anytime during pregnancy for both ZIKV+ and ZIKV- women	1 visit anytime between 11-32 months of age for infants of both ZIKV+ and ZIKV- pregnant women	46	9	46	NIH/NIAID, Wellcome Trust, and Bahia State Research Support Foundation (FAPESB)
4	Brazil_BahiaSalvador_Costa	Brazil	Healthcare Facility	Delivered	Unrelated to ZIKV	Cohort	- (enrolled delivered women)	2 visits between 24-36 months of age and then between 30-42 months of age for infants of ZIKV+ and ZIKV- women	157	110	154	NIH/NIAID, Wellcome Trust, and Bahia State Research Support Foundation (FAPESB) ZIKA-FAPESB T.O. no PET0021/2016 (www.fapesb.ba.gov.br) and Coordination for the Improvement of Higher Education (CAPES) from Brazil (https://www.capes.gov.br/)
5	Brazil_BahiaSalvador_Siqueira_czs	Brazil	Healthcare Facility	Children only	Symptoms or presence of exposure	Surveillance	- (enrolled only infants)	One visit during the first week of life for infants of women with ZIKV symptoms during pregnancy	0	0	151	WHO, CNPq (Brazil), and Fapesb (Brazil)
6	Brazil_BahiaSalvador_Siqueira_zikvsymp	Brazil	Healthcare Facility	Pregnant and delivered	Symptoms or presence of exposure	Surveillance	-	One visit during the first week of life for infants of women with ZIKV symptoms during pregnancy	101	73	102	WHO, CNPq (Brazil), and Fapesb (Brazil)
7	Brazil_Brasil_ZIKAlliance	Brazil	Healthcare Facility	Pregnant	Unrelated to ZIKV	Cohort	9 visits conducted every month during pregnancy for both ZIKV+ and ZIKV- women	6 visits at week 4, and months 4, 9, 12, 18, and 24 (final age of folllow-up) for infants of both ZIKV+ and ZIKV- pregnant women	200	-	39	European Union

For peer review only - http://bmjopen.bmj.com/site/about/guidelines.xhtml

8	Brazil_Goias_Turchi	Brazil	Community & Healthcare Facility	Pregnant	Laboratory confirmation/ Symptoms or Presence of Exposure	Cohort	7 visits anytime during prenatal care and after birth for both ZIKV+ and ZIKV- women	6 visits at months 1, 3, 6, 12, 18 (final age of follow-up) for infants of both ZIKV+ and ZIKV- pregnant women	87	58	69	European Union and FAPEG - Fundacion de Amparo a Pesquisa do Estado de Goias
9	Brazil_Maranhao_Silva	Brazil	Healthcare Facility	Children only	Laboratory confirmation/ Symptoms or Presence of Exposure	Cohort	-	Two visits per year, at 6-month intervals, upto 3 years of age (originally planned upto 4 years of age) for infants of ZIKV+ pregnant women	0	0	110	CNPq (Brazilian Research Council), CAPES (Coordination for the Improvement of Higher Education Personnel, FAPEMA (Maranhao State Research Foundation) and Science and Technology Department, Ministry of Health, Brazil
10	Brazil_MatoGrosso_SchulerFaccini	Brazil	Community	Delivered	Laboratory confirmation/ Symptoms or Presence of Exposure	Case cohort	- (enrolled delivered women)	Visits at 6, 12, 18, 24 months and then once a year up to 5 years of age for infants of ZIKV+ pregnant women	50	50	47	National Institute of Population Medical Genetics (INaGeMP)
11	Brazil_Para_Oliveira	Brazil	Community & Healthcare Facility	Pregnant	Laboratory confirmation	Cohort	4 visits every month for ZIKV+ pregnant women. ZIKV- pregnant women not enrolled.	-	134	109	102	-
12	Brazil_Pernambuco_PerezCoelho	Brazil	Community & Healthcare Facility	Children only	Laboratory confirmation/ Symptoms or Presence of Exposure	Cohort	- (enrolled only infants)	-	0	0	130	-
13	Brazil_Pernambuco_Ximenes	Brazil	Community & Healthcare Facility	Pregnant	Laboratory confirmation/ Symptoms or Presence of Exposure	Cohort	3 visits during pregnancy and after a livebirth for ZIKV+ pregnant women. ZIKV- pregnant women not enrolled.	-	700	700	600	European Union, PAHO, NIH/NIAID, Wellcome Trust, CnPq; Capes; Decit; Facepe; Secretaria de Vigilancia em Saude - Ministerio da Saude - Brasil; ERAES; Medical Research Council.
14	Brazil_RdJ_Moreira_ZIP	Brazil	Healthcare Facility	Pregnant	Unrelated to ZIKV	Cohort	6 monthly visits throughout pregnancy for both ZIKV+ and ZIKV- pregnant women	4 visits at birth, 3, 6, and 12 months (final age of follow-up) for both infants of ZIKV+ and ZIKV- pregnant women	771	17	599	NIH/NIAID

For peer review only

15	Brazil_RibieroPrietro_Duarte	Brazil	Community & Healthcare Facility	Pregnant	Laboratory confirmation	Cohort	For ZIKV+ pregnant women, visits were conducted monthly, however the number of visits varied according to the gestational age of inclusion into the study. ZIKV- pregnant women not enrolled.	Visits at birth, and months 1, 3, 6, 12, 18, and 24 (final age of follow-up) for infants of ZIKV+ pregnant women	511	511	487	Mainly supported by Fundacio de Apoio ao Ensino Pesquisa e Assistencia do Hospital das Clinicas da Faculdade de Medicina de Ribeirio Preto da Universidade de Sao Paulo (FAEPA), Brasil, and received partial support from the CVE - Centro de Vigilancia Epidemiologica 'Prof. Alexandre Vranjac' da Secretaria da Saude do Estado de Sao Paulo (SES-SP).
16	Brazil_RiodeJaneiro_Brasil	Brazil	Community	Pregnant	Laboratory confirmation/ Symptoms or Presence of Exposure	Cohort	-	-	400	244	216	Brazilian Ministry of Health (DECIT), NIH and Fondation Merieux
17	Brazil_RiodeJaneiro_Cunha	Brazil	Healthcare Facility	Reproductive age	Laboratory confirmation/ Symptoms or Presence of Exposure	Cohort	8 visits, one per month during pregnancy, for both ZIKV+ and ZIKV- women	11 visits at months 1-6, 12, 18, 24, 30, 36 (final age of follow-up) for infants of ZIKV+ pregnant women. Infants of ZIKV- pregnant women were not followed.	118	118	54	FAPERJ
18	Brazil_RiodeJaneiro_Hofer	Brazil	Healthcare Facility	Pregnant	Laboratory confirmation/ Symptoms or Presence of Exposure	Cohort	1 visit every trimester for both ZIKV+ and ZIKV- women	12 visits at months 1-6, 8, 10, 12, 18, 24 for infants of both ZIKV+ and ZIKV- pregnant women	51	38	43	FAPERJ
19	Brazil_RiodeJaneiro_Joao	Brazil	Community	Pregnant	Laboratory confirmation	Cohort	6 prenatal visits for both ZIKV+ and ZIKV- pregnant women	1 visit at 6 months of age for infants of both ZIKV+ and ZIKV- pregnant women	219	34	221	No funding
20	Brazil_RiodeJaneiro_Moreira	Brazil	Healthcare Facility	Pregnant	Laboratory confirmation/ Symptoms or Presence of Exposure	Cohort	8 visits conducted every month for ZIKV+ pregnant women (also depends on time of ZIKV infection during pregnancy). 8 visits also conducted every month for ZIKV- pregnant women.	8 visits in total each at 3, 6, 9, 12, 18, 24, 30 and 36 months of age (excluding visit at birth). Further follow-up is planned from 36-60 months.	294	294	296	European Union, Wellcome Trust, CNPq and Faperj and PIP/Fiocruz (Brazilian governamental agencies)
21	Brazil_SaoPaulo_DuartePassos	Brazil	Healthcare Facility	Pregnant	Unrelated to ZIKV	Cohort	Unspecified number of visits* *According to medical judgement	Monthly up to 6 months of age; every 3 months up to 12 months of age; every 6 months up to 3 years of age	690	57	696	Brazilian Ministry of Health, London School of Hygiene and Tropical Medicine and FAPESP
22	Brazil_SaoPaulo_LacerdaNoguiera	Brazil	Healthcare Facility	Pregnant	Laboratory confirmation	Cohort	1 visit at enrollment and then 1 visit every trimester for ZIKV+ women. ZIKV- pregnant women not enrolled	-	54	54	54	FAPESP

For peer review only - http://bmjopen.bmj.com/site/about/guidelines.xhtml

23	Brazil_Segurado_ZIKAlliance	Brazil	Healthcare Facility	Pregnant	Unrelated to ZIKV	Cohort	9 visits conducted every month during pregnancy for both ZIKV+ and ZIKV- women	6 visits at week 4, and months 4, 9, 12, 18, and 24 (final age of follow-up) for infants of both ZIKV+ and ZIKV- pregnant women	106	0	48	European Union
24	Brazil_SP_Mussi_ZIP	Brazil	Community & Healthcare Facility	Pregnant	Unrelated to ZIKV	Cohort	On average 12 visits before delivery, 1 visit at delivery and 1 visit 6 weeks after delivery for both ZIKV+ and ZIKV- women	4 visits at birth, 3, 6, and 12 months (final age of follow-up) for both infants of ZIKV+ and ZIKV- pregnant women	554	0	418	NIH/NIAID
25	Colombia_Arias_ZIP	Colombia	Healthcare Facility	Pregnant	Unrelated to ZIKV	Cohort	9 visits at enrollment, monthly thereafter, and six weeks post partum for both ZIKV+ and ZIKV- women	4 visits at birth, 3-5 months, 6-11 months, 12-18 months for both infants of ZIKV+ and ZIKV- pregnant women	435	3	206	NIH/NIAID
26	Colombia_Becerra	Colombia	Healthcare Facility	Pregnant	Laboratory confirmation/ Symptoms or Presence of Exposure	Cohort	Monthly visits throughout pregnancy for both ZIKV+ and ZIKV- pregnant women	4 visits at months 6, 12, 18, 24 (final age of follow-up), for infants of both ZIKV+ and ZIKV- pregnant women	243	124	226	COLCIENCIAS Universidad Industrial De Santander Ascon Hospital Universitario De Santander
27	Colombia_Gilboa	Colombia	Healthcare Facility	Pregnant	Unrelated to ZIKV	Cohort	Both ZIKV+ and ZIKV- women had 14-16 visits if they were enrolled early in the first trimester and had a full-term pregnancy. If not, the visits were fewer. There was also one postpartum visit.	Some infants were only followed up until 6 months of age with visits at delivery, 10 days of age, and thereafter every two weeks. Other infants were followed up until 18 months with additional visits at 9, 12, and 18 months of age	1519	16	1239	US CDC, USAID, and Instituto Nacional de Salud (Colombia)
28	Colombia_Lopez Medina	Colombia	Community	Pregnant	Laboratory confirmation	Cohort	- (only followed up with infants)	Visits every 3-4 months until 18 months (final age of follow-up)	170	170	154	Thrasher Foundation
29	Colombia_Mattar	Colombia	Community	Delivered	Symptoms or presence of exposure	Surveillance	8 visits during every trimester for both ZIKV+ and ZIKV- women	4 visits at 6, 12, 18, 24 months (final age of follow-up) for infants of both ZIKV+ and ZIKV- pregnant women	11	11	11	WHO
30	Colombia_MercadoReyes	Colombia	Healthcare Facility	Pregnant	Laboratory confirmation/ Symptoms or Presence of Exposure	Surveillance	14 visits across pregnancy for both ZIKV+ and ZIKV- women	Study did not dictate number of follow-ups, but data that were available in medical records were abstracted up to 2 years of age for infants of both ZIKV+ and ZIKv- pregnant women	1218	253	1185	US CDC
31	Colombia_Mulkey	Colombia	Community + Travelers	Pregnant	Symptoms or presence of exposure	Cohort	2 visits if the ZIKV+ pregnant woman is enrolled prior to 32 weeks of gestation. ZIKV- pregnant women not enrolled	2 visits at 6 months and between 12-18 months for infants of ZIKV+ pregnant women	82	82	80	Ikaria Fund; Thrasher Research Fund; NIH

32	Colombia_SanzCortes	Colombia	Healthcare Facility	Pregnant	Laboratory confirmation/ Symptoms or Presence of Exposure	Cohort	An average of 5 monthly visits for ZIKV+ pregnant women. ZIKV- pregnant women not enrolled	An average of 5 monthly visits for infants of ZIKV+ pregnant women	17	17	17	No funding
33	Colombia_Villar_ZIKAlliance	Colombia	Healthcare Facility	Pregnant	Unrelated to ZIKV	Cohort	9 visits conducted every month during pregnancy for both ZIKV+ and ZIKV- women	6 visits at week 4, and months 4, 9, 12, 18, and 24 (final age of follow-up) for infants of both ZIKV+ and ZIKV- pregnant women	590	20	473	European Union
34	Ecuador_Soria_ZIKAlliance	Ecuador	Healthcare Facility	Pregnant	Unrelated to ZIKV	Cohort	9 visits conducted every month during pregnancy for both ZIKV+ and ZIKV- women	6 visits at week 4, and months 4, 9, 12, 18, and 24 (final age of folllow-up) for infants of both ZIKV+ and ZIKV- pregnant women	436	436	151	European Union
35	FrenchGuiana_Pomar	France	Healthcare Facility	Pregnant	Laboratory confirmation	Cohort	Monthly visits were conducted after inclusion for both ZIKV+ and ZIKV- pregnant women, capped at a maximum of 6 visits, as inclusion occured mainly after the results of the first serology performed during the first trimester	1 visit at 3 days post-partum for infants of both ZIKV+ and ZIKV- pregnant women	700	301	305	-
36	Grenada_LaBeaud	Grenada	Healthcare Facility	Pregnant and delivered	Symptoms or presence of exposure	Cohort	One prenatal and one postnatal visit for both ZIKV+ and ZIKV- women	Variable number of visits at variable ages upto a maximum of 30 months of age for both infants of ZIKV+ and ZIKV- pregnant women	384	113	388	NIH/NIAID, USAID, National Institutes of Health Eunice Kennedy Shriver, National Institute of Child Health and Human Development, Windward Islands Research and Education Foundation, Stanford Maternal Child Health Research Institute
37	Guadaloupe_Cabie	France	Healthcare Facility	Pregnant	Laboratory confirmation/ Symptoms or Presence of Exposure	Cohort	8 visits in total, one every month during pregnancy, for ZIKV+ women<br>4 visits in total, one every trimester and one at delivery, for ZIKV- women	5 visits at 2, 4, 9, 18, 24 (final age of follow-up) months for infants of both ZIKV+ and ZIKV- pregnant women	4959	1564	1180	French Ministry of Health
38	Guatemala_EspinosaBode_ZINC	Guatemala	Healthcare Facility	Pregnant	Unrelated to ZIKV	Cohort	15 visits at recruitment (if <20 weeks), 26, and 32 weeks, as well as at birth	2 visits at birth and at 3 months (final age of follow-up) for both infants of ZIKV+ and ZIKV- pregnant women	436	0	389	US CDC

For peer review only - http://bmjopen.bmj.com/site/about/guidelines.xhtml

39	Guatemala_Figueroa_ZIP	Guatemala	Community & Healthcare Facility	Pregnant	Unrelated to ZIKV	Cohort	10 monthly visits across pregnancy for both ZIKV+ and ZIKV- women	4 visits at birth, 3, 6, and 12 months (final age of follow-up) for both infants of ZIKV+ and ZIKV- pregnant women	998	0	722	NIH/NIAID
40	HaitiJamaica_ZIKAction	Haiti and Jamaica	Healthcare Facility	Pregnant	Unrelated to ZIKV	Cohort	4 visits at enrollment, 20, 28, and 36 weeks for both ZIKV+ and ZIKV- pregnant women. Extra visits were conducted if the woman was ill.	6 visits at 1, 4, 9, 12, 18, 24 (final age of follow-up) months for infants of both ZIKV+ and ZIKV- pregnant women	1147	-	-	European Union
41	Honduras_Alger	Honduras	Healthcare Facility	Pregnant	Unrelated to ZIKV	Cohort	1 visit upon enrollment for both ZIKV+ and ZIKV- pregnant women	1 visit for infants of both ZIKV+ and ZIKV- pregnant women	3995	7	645	US CDC and School of Public Health and Tropical Medicine, Tulane University
42	Kenya_Widdowson	Kenya	Healthcare Facility	Pregnant	Symptoms or presence of exposure	Cohort	10 monthly visits for ZIKV+ and ZIKV- pregnant women	-	2312	3	0	US CDC
43	Mexico_Alpuche_ZIKAlliance	Mexico	Healthcare Facility	Pregnant	Unrelated to ZIKV	Cohort	7 visits conducted every month during pregnancy for both ZIKV+ and ZIKV- women	6 visits at week 4, and months 4, 9, 12, 18, and 24 (final age of folllow-up) for infants of both ZIKV+ and ZIKV- pregnant women	-	-	-	European Union
44	Mexico_Borja_ZIKAlliance	Mexico	Healthcare Facility	Pregnant	Unrelated to ZIKV	Cohort	8 visits at 6-8, 10-13, 16-18, 22, 28, 32, 36, and 38-41 weeks for both ZIKV+ and ZIKV- pregnant women	4 visits at week 4, and months 4, 12, and 24 (final age of follow-up) for infants of both ZIKV+ and ZIKV- pregnant women	1144	63	657	European Union
45	Nicaragua_Balmaseda_ZIP	Nicaragua	Healthcare Facility	Pregnant	Unrelated to ZIKV	Cohort	On average 10 visits every month during pregnancy, 1 at delivery and 1 visit 6 weeks after delivery for both ZIKV+ and ZIKV- women	4 visits at birth, 3, 6, and 12 months (final age of follow-up) for both infants of ZIKV+ and ZIKV- pregnant women	722	0	-	NIH/NIAID
46	Peru_Gotuzzo_ZIKAlliance	Peru	Healthcare Facility	Pregnant	Unrelated to ZIKV	Cohort	9 visits conducted every month during pregnancy for both ZIKV+ and ZIKV- women	6 visits at week 4, and months 4, 9, 12, 18, and 24 (final age of folllow-up) for infants of both ZIKV+ and ZIKV- pregnant women	153	83	153	European Union
47	PuertoRico_ValenciaPrado_ZAPSS	Puerto Rico	Community & Healthcare Facility	Pregnant and delivered	Laboratory confirmation	Surveillance	-	-	4280	4280	4014	US CDC
48	Singapore_Chan	Singapore	Healthcare Facility	Pregnant	Laboratory confirmation/ Symptoms or Presence of Exposure	Cohort	7 visits every 4-6 weeks during pregnancy for ZIKV+ women. ZIKV- pregnant women were not enrolled.	-	15	15	18	Ministry of Health, Singapore KK Women's and Children's Hospital

For peer review only

49	Spain_Bardaji	Spain	Healthcare Facility	Pregnant	Laboratory confirmation/ Symptoms or Presence of Exposure	Surveillance	Monthly follow-ups for ZIKV+ women throughout pregnancy. ZIKV- women were also followed monthly until a negative diganosis, after which they were referred to the closest healthcare centre for follow-up	7 visits at birth, and at months 1, 2, 6, 12, 18 and 24 (final age of follow up) for children of ZIKV+ pregnant women. Children of ZIKV- pregnant women were not followed	195	44	42	The Government of Spain, Fondo de Investigación en Salud (FIS), Instituto de Salud Carlos III.
50	Spain_Soriano	Spain	Travelers	Pregnant	Symptoms or presence of exposure	Cohort	At lesst one visit per trimester for ZIKV+ pregnant women. ZIKV- pregnant women not enrolled.	Visits scheduled at 1, 4, 9, 12, 18, 24 and 36 months of age for children of ZIKV+ pregnant women	186	186	213	European Union
51	Suriname_Juliana	Suriname	Healthcare Facility	Delivered	Laboratory confirmation/ Symptoms or Presence of Exposure	Surveillance	2 visits for ZIKV+ women with one during pregnacy and another after. ZIKV- pregnant women were not enrolled	Visits at 3, 6, 9, and 12 months for infants with evidence of congential ZIKV syndrome. After this, children seen on an as-needed basis. Another complete assessment was conducted at 4 years of age	22	16	22	No funding
52	TrinidadTobago_ Sohan	Trinidad and Tobago	Healthcare Facility	Pregnant	Laboratory confirmation	Cohort	12 visits that occur every month until 26 weeks and then every 3 weeks until 36 weeks for ZIKV+ pregnant women. More visits can occur if abnormalities are detected<br>2 visits that occur during the trimester and then during the second or third trimester for ZIKV- pregnant women	-	100	100	0	-
53	USA_Mulkey	USA	Healthcare Facility	Pregnant	Symptoms or presence of exposure	Cohort	1 visit for ZIKV+ and ZIKV- pregnant women usually after 18 weeks of gestation	1-5 visits at birth and 3, 6, 12, and 18 months	72	29	42	Ikaria Fund; Thrasher Research Fund; NIH
54	USA_USZPIR	USA	Community + Travelers	Pregnant and delivered	Laboratory confirmation	Surveillance	Medical records used for ZIKV+ pregnant women. Maternal data was collected at time of identification and then each trimester following. ZIKV- pregnant women not enrolled	Data on infants collected at birth and then at 2, 6, 12, 18, 24, 30, and 36 months	603	603	528	US CDC

ZIKV: Zika Virus; DENV: Dengue Virus; CHIKV: Chikungunya Virus; ZIKV(+): ZIKV positive individuals;

Note: Study Name convention: Country_City/PIName_StudyConsortium (if multisite)

For peer review only - http://bmjopen.bmj.com/site/about/guidelines.xhtml

Table S1. Detailed description of ZIKV-IPD-MA studies participating in the metadata survey (Part 2).

ID	Study Name	Maternal ZIKV assessment	Fetal ZIKV assessment	Infant ZIKV assessment	Maternal PCR	Maternal PRNT	Fetal PCR	Prenatal microcephaly definition	Postnatal microcephaly assessment	Identification of CZS malformations	CZS Definition	DENV testing	CHIKV Testing
1	Bolivia_Roca_ZIKAlliance	Laboratory testing	Maternal clinical/laboratory criteria, fetal laboratory criteria	Infant clinical/laboratory criteria	Yes	No	Yes	-	No*	Peri/intrapartum and postpartum	WHO	Yes	Yes
2	Brazil_Bahia_Ko_NIHNIAD	Laboratory testing	-	Infant clinical/laboratory, imaging, and maternal clinical/laboratory criteria	Yes	No	No	2 SD below the mean	Yes	Antenatal and peri/intrapartum and postpartum	WHO	Yes	Yes
3	Brazil_BahiaPaudaLima_Costa	Laboratory testing	-	-	No	No	No	-	Yes	Postpartum	Study definition	No	No
4	Brazil_BahiaSalvador_Costa	Laboratory testing	-	Infant clinical/laboratory, imaging and maternal clinical/laboratory criteria	No	Yes	No	-	Yes	Postpartum	Study definition	No	No
5	Brazil_BahiaSalvador_Siqueira_czs	-	Placental laboratory criteria	Infant clinical/laboratory, imaging and maternal clinical/laboratory criteria	No	No	No	2 SD below the mean	Yes	Postpartum	Country	No	No
6	Brazil_BahiaSalvador_Siqueira_zikvsymp	Clinical case definition and/or Laboratory confirmation	Placental laboratory criteria	Infant clinical/laboratory, imaging and maternal clinical/laboratory criteria	No	No	No	2 SD below the mean	Yes	Postpartum	Country	Yes	Yes
7	Brazil_Brasil_ZIKAlliance	Laboratory testing	-	Infant clinical/laboratory, imaging and maternal clinical/laboratory criteria	Yes	Yes	No	2 SD below the mean	No*	-	-	Yes	Yes
8	Brazil_Goias_Turchi	Laboratory testing	Fetal imaging criteria	Infant clinical/laboratory criteria	Yes	No	No	2 SD below the mean	Yes	Postpartum	Country	Yes	Yes
9	Brazil_Maranhao_Silva	Clinical case definition	-	Infant clinical/laboratory, imaging and maternal clinical/laboratory criteria	No	No	No	-	Yes	Postpartum	Study definition	No	No
10	Brazil_MatoGrosso_SchulerFaccini	Clinical case definition and/or Laboratory confirmation	-	-	Yes	No	No	-	Yes	Peri/intrapartum	Country	No	No

For peer review only - http://bmjopen.bmj.com/site/about/guidelines.xhtml

11	Brazil_Para_Oliveira	Laboratory testing	Maternal clinical/laboratory criteria and placental laboratory criteria	Maternal clinical/laboratory criteria	Yes	Yes	No	Below the 5th percentile	Yes	Peri/intrapartum and postpartum	Country	Yes	Yes
12	Brazil_Pernambuco_PerezCoelho	-	-	Infant clinical/laboratory and infant imaging criteria	No	No	No	-	Yes	Postpartum	Country	No	No
13	Brazil_Pernambuco_Ximenes	Laboratory testing	-	Infant clinical/laboratory, imaging and maternal clinical/laboratory criteria	Yes	Yes	No	-	Yes	Peri/intrapartum and postpartum	Study definition	Yes	Yes
14	Brazil_RdJ_Moreira_ZIP	Laboratory testing	Maternal clinical/laboratory, fetal imaging, laboratory and placental criteria	Infant clinical/laboratory, imaging and maternal clinical/laboratory criteria	Yes	No	Yes	2 SD below the mean	Yes	Antenatal and peri/intrapartum and postpartum	WHO	Yes	Yes
15	Brazil_RibieroPrietro_Duarte	Laboratory testing	Fetal imaging, laboratory and placental criteria	Infant clinical/laboratory criteria and maternal clinical/laboratory criteria	Yes	No	Yes	Below the 3rd centile	Yes	Postpartum	Country	No	No
16	Brazil_RiodeJaneiro_Brasil	Laboratory testing	Maternal clinical/laboratory, fetal imaging and laboratory criteria	Infant clinical/laboratory and infant imaging criteria	Yes	No	Yes	3 SD below the mean	Yes	Antenatal and postpartum	Study definition	Yes	Yes
17	Brazil_RiodeJaneiro_Cunha	Clinical case definition and/or Laboratory confirmation	Placental laboratory criteria	Infant clinical/laboratory, imaging and maternal clinical/laboratory criteria	Yes	No	No	2 SD below the mean	Yes	Peri/intrapartum	Country	Yes	Yes
18	Brazil_RiodeJaneiro_Hofer	Laboratory testing	-	Infant clinical/laboratory, imaging and maternal clinical/laboratory criteria	Yes	No	No	2 SD below the mean	Yes	Postpartum	Country	Yes	No
19	Brazil_RiodeJaneiro_Joao	Laboratory testing	-	-	Yes	Yes	No	-	Yes	Antenatal and peri/intrapartum and postpartum	Country	Yes	Yes
20	Brazil_RiodeJaneiro_Moreira	Clinical case definition and/or Laboratory confirmation	Maternal clinical/laboratory, fetal imaging, laboratory and placental criteria	Infant clinical/laboratory, imaging and maternal clinical/laboratory criteria	Yes	No	Yes	3 SD below the mean	Yes	Antenatal and peri/intrapartum and postpartum	WHO	Yes	Yes

For peer review only

For peer review only - http://bmjopen.bmj.com/site/about/guidelines.xhtml

21	Brazil_SaoPaulo_DuartePassos	Laboratory testing	-	Infant clinical/laboratory and infant imaging criteria	Yes	No	No	2 SD below the mean	Yes	Antenatal and postpartum	CDC/WHO /Country	Yes	Yes
22	Brazil_SaoPaulo_LacerdaNoguiera	Laboratory testing	-	Infant clinical/laboratory criteria	Yes	No	No	-	Yes	Antenatal and postpartum	Study definition	Yes	No
23	Brazil_Segurado_ZIKAlliance	Laboratory testing	Maternal clinical/laboratory, fetal imaging, laboratory and placental criteria	-	Yes	No	Yes	3 SD below the mean	Yes	Postpartum	Study definition	No	Yes
24	Brazil_SP_Mussi_ZIP	Laboratory testing	-	-	Yes	No	No	2 SD below the mean	Yes	Antenatal and peri/intrapartum and postpartum	WHO	Yes	Yes
25	Colombia_Arias_ZIP	Laboratory testing	Fetal laboratory criteria	Infant clinical/laboratory criteria	Yes	No	Yes	3 SD below the mean	Yes	-	-	Yes	Yes
26	Colombia_Becerra	Laboratory testing	Maternal clinical/laboratory, fetal imaging, laboratory and placental criteria	Infant clinical/laboratory criteria and maternal clinical/laboratory criteria	Yes	No	Yes	2 SD below the mean	Yes	Antenatal and peri/intrapartum and postpartum	WHO	Yes	No
27	Colombia_Gilboa	Laboratory testing	Fetal laboratory criteria	Infant clinical/laboratory criteria	Yes	No	Yes	Other	Yes	Antenatal and postpartum	CDC	Yes	Yes
28	Colombia_Lopez Medina	Laboratory testing	-	-	Yes	No	No	2 SD below the mean	Yes	-	-	No	No
29	Colombia_Mattar	Laboratory testing	-	-	Yes	No	No	2 SD below the mean	Yes	Antenatal and peri/intrapartum	Country	No	Yes
30	Colombia_MercadoReyes	Clinical case definition and/or Laboratory confirmation	Maternal clinical/laboratory, fetal imaging, laboratory and placental criteria	Infant clinical/laboratory, imaging and maternal clinical/laboratory criteria	Yes	No	No	3 SD below the mean	Yes	Antenatal and peri/intrapartum and postpartum	Study definition	Yes	Yes
31	Colombia_Mulkey	Clinical case definition and/or Laboratory confirmation	-	Maternal clinical/laboratory criteria	Yes	Yes	No	3 SD below the mean	Yes	Antenatal and postpartum	Study definition	Yes	No

For peer review only - http://bmjopen.bmj.com/site/about/guidelines.xhtml

32	Colombia_SanzCortes	Laboratory testing	Maternal clinical/laboratory, fetal imaging, laboratory and placental criteria	Infant clinical/laboratory, imaging and maternal clinical/laboratory criteria	Yes	No	Yes	3 SD below the mean	Yes	Antenatal and peri/intrapartum and postpartum	Country	No	No
33	Colombia_Villar_ZIKAlliance	Laboratory testing	-	-	Yes	No	No	-	Yes	Antenatal and peri/intrapartum and postpartum	WHO	Yes	Yes
34	Ecuador_Soria_ZIKAlliance	Laboratory testing	Fetal imaging, laboratory and placental criteria	Infant clinical/laboratory criteria	Yes	No	Yes	2 SD below the mean	Yes	Postpartum	Country	No	No
35	FrenchGuiana_Pomar	Laboratory testing	Fetal imaging, laboratory and placental criteria	Infant clinical/laboratory criteria	Yes	Yes	Yes	2 SD below the mean	No*	Antenatal and postpartum	Study definition	Yes	Yes
36	Grenada_LaBeaud	Laboratory testing	-	Infant clinical/laboratory criteria	Yes	No	No	-	Yes	Postpartum	WHO	Yes	Yes
37	Guadaloupe_Cabie	Laboratory testing	Placental laboratory criteria	Infant clinical/laboratory criteria and maternal clinical/laboratory criteria	Yes	No	No	2 SD below the mean	Yes	Antenatal and peri/intrapartum and postpartum	Study definition	No	No
38	Guatemala_EspinosaBode_ZINC	Clinical case definition and/or Laboratory confirmation	-	Infant clinical/laboratory criteria	Yes	No	No	2 SD below the mean	Yes	Postpartum	CDC	Yes	Yes
39	Guatemala_Figueroa_ZIP	Laboratory testing	Maternal clinical/laboratory criteria	Infant clinical/laboratory criteria	Yes	No	No	2 SD below the mean	Yes	-	-	Yes	No
40	HaitiJamaica_ZIKAction	Laboratory testing	-	Infant clinical/laboratory and infant imaging criteria	Yes	Yes	No	2 SD below the mean	No*	Antenatal and postpartum	Study definition	Yes	Yes
41	Honduras_Alger	Laboratory testing	-	-	Yes	No	No	-	Yes	-	-	Yes	-
42	Kenya_Widdowson	Clinical case definition and/or Laboratory confirmation	Maternal clinical/laboratory, fetal laboratory, and placental criteria	Infant clinical/laboratory criteria	Yes	Yes	Yes	-	No*	Postpartum	Intergrowth	Yes	Yes
43	Mexico_Alpuche_ZIKAlliance	Clinical case definition and/or Laboratory confirmation	-	-	Yes	No	No	-	Yes	Peri/intrapartum and postpartum	-	-	-
44	Mexico_Borja_ZIKAlliance	Laboratory testing	Fetal imaging, laboratory and	Infant clinical/laboratory and infant imaging criteria	Yes	No	Yes	2 SD below the mean	No*	Postpartum	Study definition	Yes	Yes

For peer review only - http://bmjopen.bmj.com/site/about/guidelines.xhtml

			placental criteria										
45	Nicaragua_Balmaseda_ZIP	Laboratory testing	-	-	Yes	No	-	-	-	-	-	-	-
46	Peru_Gotuzzo_ZIKAlliance	Laboratory testing	Fetal imaging, laboratory and placental criteria	Infant clinical/laboratory and infant imaging criteria	Yes	No	Yes	2 SD below the mean	Yes	Peri/intrapartum and postpartum	Country	No	Yes
47	PuertoRico_ValenciaPrado_ZAPSS	Laboratory testing	Placental laboratory criteria	Infant clinical/laboratory criteria	Yes	No	No	-	Yes	Antenatal and peri/intrapartum and postpartum	Study definition	Yes	Yes
48	Singapore_Chan	Laboratory testing	-	Infant clinical/laboratory criteria	Yes	No	No	2 SD below the mean	Yes	Antenatal and postpartum	Country	No	No
49	Spain_Bardaji	Laboratory testing	Maternal clinical/laboratory, fetal imaging, laboratory and placental criteria	Infant clinical/laboratory, imaging and maternal clinical/laboratory criteria	Yes	No	Yes	2 SD below the mean	Yes	Antenatal and peri/intrapartum and postpartum	WHO	Yes	Yes
50	Spain_Soriano	Clinical case definition and/or Laboratory confirmation	Maternal clinical/laboratory, fetal imaging, laboratory and placental criteria	Infant clinical/laboratory and infant imaging criteria	Yes	Yes	Yes	2 SD below the mean	Yes	Antenatal and peri/intrapartum and postpartum	Country	Yes	Yes
51	Suriname_Juliana	Clinical case definition and/or Laboratory confirmation	-	Infant clinical/laboratory, imaging and maternal clinical/laboratory criteria	Yes	No	No	-	Yes	Postpartum	WHO	No	No
52	TrinidadTobago_Sohan	Clinical case definition and/or Laboratory confirmation	-	-	Yes	No	No	Below the 5th percentile	Yes	-	-	Yes	Yes
53	USA_Mulkey	Clinical case definition and/or Laboratory confirmation	-	Maternal clinical/laboratory criteria	Yes	Yes	No	3 SD below the mean	Yes	Antenatal and peri/intrapartum and postpartum	Study definition	Yes	No
54	USA_USZPIR	Laboratory testing	Fetal laboratory and placental criteria	Infant clinical/laboratory criteria	Yes	Yes	Yes	3 SD below the mean	Yes	Antenatal and peri/intrapartum and postpartum	CDC	No	No

ZIKV: Zika Virus; DENV: Dengue Virus; CHIKV: Chikungunya Virus; ZIKV(+): ZIKV positive individuals;

For peer review only

For peer review only - http://bmjopen.bmj.com/site/about/guidelines.xhtml

Table S2. Infant and child development assessment among ZIKV-IPD-MA study participants of the metadata survey.

**Characteristic**	N = 40[1]
**Age of Developmental Assessment (Months)**	
**Median (IQR)**	24 (18, 30)
**Range**	3, 60
**Developmental assessment tool**	
**ASQ-SE Tool**	21 / 40 (52%)
**BSID Tool**	30 / 40 (75%)
**WIDEA-FS Tool**	2 / 39 (5.1%)
**AIMS Tool**	4 / 40 (10%)
**Other Assessments***	18 / 40 (45%)

[1] N= Total studies; Frequency n/N (row) (%);

Bayley Scales of Infant Development (BSID); Ages & Stages Questionnaires® Social-Emotional, Second Edition (ASQ®-SE-2); Warner Initial Developmental Evaluation of Adaptive and Functional Skills (WIDEA) and four the Alberta Infant Motor Scale (AIMS). Note: developmental assessment was only reported by 40 studies, so the denominator is 40 studies. *Includes INTER-NDA and NEPSY-II

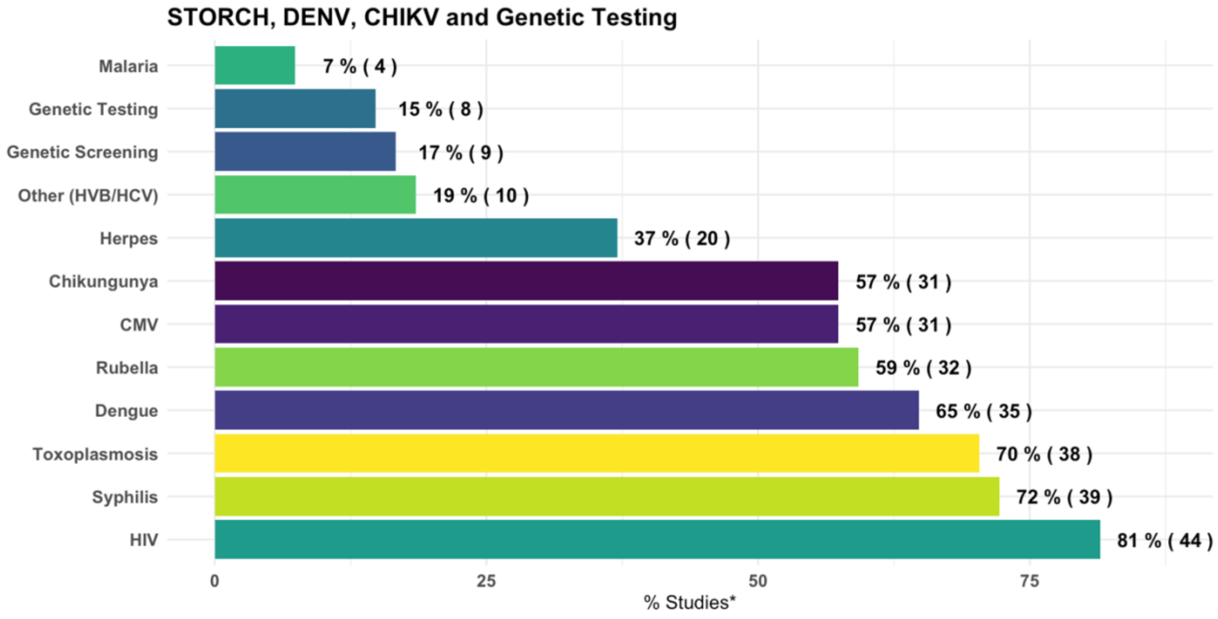

ZIKV: Zika Virus; DENV: Dengue Virus; CHIKV: Chikungunya Virus; HIV: Human Immunodeficiency Virus; CMV: Cytomegalovirus; HVB/HCV: Hepatitis B and Hepatitis C Virus.

Figure S2. STORCH and other additional testing among ZIKV-IPD-MA study participants of the metadata survey. The denominator is total number of studies (n=54)

For peer review only - http://bmjopen.bmj.com/site/about/guidelines.xhtml

Table S3. Sociodemographic and behavioral factors measured among ZIKV-IPD-MA study participants of the metadata survey.

Characteristic	N = 52[1]
**Education**	43 / 52 (83%)
**Occupation**	32 / 52 (62%)
**Marital status**	40 / 52 (77%)
**Nutrition status**	15 / 51 (29%)
**Maternal exposure to workplace or environmental occupational teratogens**	17 / 51 (33%)
**Pre-pregnancy exposure to IPV**	4 / 52 (7.7%)
**Maternal exposure to IPV**	9 / 51 (18%)
**Infant/child exposure to IPV**	7 / 52 (13%)
**Maternal exposure to vaccination**	33 / 51 (65%)
**Maternal prescription drug use**	40 / 51 (78%)
**Maternal recreational drug use**	42 / 51 (82%)
**Maternal ethnic or minority status**	35 / 52 (67%)
**Household SES**	37 / 52 (71%)
**Mental health scales**	4 / 51 (7.8%)
**Maternal smoking**	42 / 51 (82%)
**Maternal alcohol consumption**	41 / 51 (80%)

[1] N= Total studies; Frequency n/N (row) (%)

Note: There were Two studies that did not provide any information about the sociodemographic metadata.

For peer review only - http://bmjopen.bmj.com/site/about/guidelines.xhtml

Table S4. Genetic testing, fetal ultrasounds, and MRIs among ZIKV-IPD-MA study participants of the metadata survey

**Characteristic**	**Overall N = 53**[1]	**Other Countries N = 30**[1]	**Brazil N = 23**[1]
**Genetic Screening**	9 / 53 (17%)	7 / 30 (23%)	2 / 23 (8.7%)
**Genetic Testing**	8 / 52 (15%)	6 / 29 (21%)	2 / 23 (8.7%)
**Fetal Ultrasounds**	39 / 53 (74%)	24 / 30 (80%)	15 / 23 (65%)
**Fetal Ultrasound Timing**			
Monthly	7 / 39 (18%)	6 / 24 (25%)	1 / 15 (6.7%)
Once per Trimester	17 / 39 (44%)	6 / 24 (25%)	11 / 15 (73%)
2nd & 3rd Trimester	3 / 39 (7.7%)	3 / 24 (12%)	0 / 15 (0%)
Twice per Pregnancy	3 / 39 (7.7%)	2 / 24 (8.3%)	1 / 15 (6.7%)
Unspecified Timing	9 / 39 (23%)	7 / 24 (29%)	2 / 15 (13%)
**Number of Ultrasounds per Woman**			
At least 3	25 / 39 (64%)	13 / 24 (54%)	12 / 15 (80%)
4 to 6	5 / 39 (13%)	3 / 24 (12%)	2 / 15 (13%)
More than 7	3 / 39 (7.7%)	3 / 24 (12%)	0 / 15 (0%)
Unspecified Number	6 / 39 (15%)	5 / 24 (21%)	1 / 15 (6.7%)
**Postnatal Ultrasound**	36 / 53 (68%)	23 / 30 (77%)	13 / 23 (57%)
**Fetal MRIs**	10 / 52 (19%)	7 / 29 (24%)	3 / 23 (13%)
**Postnatal MRI**	17 / 52 (33%)	9 / 29 (31%)	8 / 23 (35%)

[1] Frequency n/(N row) (%)

Table S5a. Type of maternal ZIKV assessment among ZIKV-IPD-MA study participants of the metadata survey.

Characteristic	N = 51[1]	Characteristic	N = 51[1]
**Type of Maternal ZIKV Assessment**		**Performed PRNT**	12 / 51 (24%)
Clinical and Laboratory Criteria	13 / 51 (25%)	**PRNT %**	
Laboratory Criteria	38 / 51 (75%)	PRNT50	2 / 12 (17%)
**Performed PCR**	48 / 51 (94%)	PRNT90	6 / 12 (50%)
**PCR Type**		Unknown	4 / 12 (33%)
Conventional PCR	5 / 48 (10%)	**PRNT Cut-off Titers**	
qRT-PCR	15 / 48 (31%)	PRNT>20	5 / 12 (42%)
RT-PCR	27 / 48 (56%)	PRNT≥10	5 / 12 (42%)
Unknown	1 / 48 (2.1%)	Unknown	2 / 12 (17%)
**Performed QC for PCR**		**Performed DENV/ZIKV PRNT**	
No	5 / 48 (10%)	No	8 / 13 (62%)
Unknown	8 / 48 (17%)	Unknown	3 / 13 (23%)
Yes	35 / 48 (73%)	Yes	2 / 13 (15%)
**Performed Immunoassays**	35 / 51 (69%)	**Performed QC for PRNT**	
**Performed ELISA IgM**	34 / 51 (67%)	No	10 / 12 (83%)
**Performed ELISA IgG**	18 / 51 (35%)	Unknown	1 / 12 (8.3%)
**Performed QC for ELISAs**		Yes	1 / 12 (8.3%)
No	1 / 35 (2.9%)		
Unknown	8 / 35 (23%)		
Yes	26 / 35 (74%)		

[1] Frequency n/(N row) (%)

Table S5b. Type of fetal ZIKV assessment among ZIKV-IPD-MA study participants of the metadata survey.

**Characteristic**	**N = 27**[1]
**Type of Fetal ZIKV Assessment**	
Fetal Imaging Criteria	1 / 27 (3.7%)
Fetal Laboratory Criteria	2 / 27 (7.4%)
Maternal & Fetal Laboratory Criteria	7 / 27 (26%)
Maternal & Fetal Laboratory Criteria, including Imaging	11 / 27 (41%)
Maternal Laboratory Criteria	6 / 27 (22%)
**Performed PCR**	18 / 27 (67%)
**PCR Type**	
Conventional PCR	1 / 18 (5.6%)
qRT-PCR	8 / 18 (44%)
RT-PCR	9 / 18 (50%)
**Performed QC for PCR**	
No	3 / 18 (17%)
Unknown	2 / 18 (11%)
Yes	13 / 18 (72%)
**Performed ELISA IgM**	8 / 27 (30%)
**Performed ELISA IgG**	4 / 27 (15%)
**Performed QC for ELISAs**	
No	1 / 9 (11%)
Unknown	1 / 9 (11%)
Yes	7 / 9 (78%)
**Performed PRNT**	1 / 27 (3.7%)

[1] Frequency n/(N row) (%)

Table S5c. Type of infant ZIKV assessment among ZIKV-IPD-MA study participants of the metadata survey.

**Characteristic**	**N = 41**[1]
**Type of Infant ZIKV Assessment**	
Infant Laboratory & Imaging Criteria	7 / 41(17%)
Infant Laboratory Criteria	14 / 41 (34%)
Maternal & Infant Laboratory Criteria	3 / 41 (7%)
Maternal & Infant Laboratory Criteria, including Imaging	15 / 41 (37%)
Maternal Clinical/Laboratory Criteria	2 / 41 (5%)
**Performed PCR**	33 / 41 (80%)
**PCR Type**	
Conventional PCR	5 / 33 (15%)
qRT-PCR	13 / 33 (39%)
RT-PCR	13 / 33 (39%)
Unknown	2 / 33 (6.1%)
**Performed QC for PCR**	
No	5 / 33 (15%)
Unknown	7 / 33 (21%)
Yes	21 / 33 (64%)
**Performed ELISA IgM**	24 / 41 (59%)
**Performed ELISA IgG**	14 / 41 (34%)
**Performed QC for ELISAs**	
No	2 / 25 (8.0%)
Unknown	5 / 25 (20%)
Yes	18 / 25 (72%)
**Performed PRNT**	5 / 41 (12%)

[1] Frequency n/(N row) (%)

ZIKV IPD-MA Metadata survey

The metadata survey collects information from investigators about how their study or site measured ZIKV and DENV infection, how positive, negative, and indeterminate ZIKV tests were classified, and information on the measurement of confounders and outcomes of interest. These data will be used to appropriately account for study-level heterogeneity and measurement error in the statistical models developed for ZIKV IPD-MA Objectives 1-4.

The metadata survey may take up to one hour to complete and may require input from different members of your study team. Please contact Lauren Maxwell at maxwelll@who.int if you have questions while completing the metadata survey.

El mismo mensaje en español

La encuesta de metadatos recopila información de los investigadores sobre cómo su estudio o sitio midió la infección por ZIKV y DENV, cómo se clasificaron las pruebas de ZIKV positivas, negativas e indeterminadas, e información sobre la medición de factores de confusión y resultados de interés. Estos datos se utilizarán para explicar adecuadamente la heterogeneidad a nivel de estudio y el margin de error en los modelos estadísticos desarrollados para ZIKV IPD-MA Objetivos 1-4.

La encuesta de metadatos puede tardar hasta una hora en completarse y requerirá aportes de diferentes miembros de su equipo de investigacion. Por favor, póngase en contacto con Lauren Maxwell at maxwelll@who.int si tiene preguntas mientras completa la encuesta de metadatos.

A mesma mensagem em português

A pesquisa de metadados coleta informações de pesquisadores sobre como o estudo ou local mediu a infecção por ZIKV e DENV, como foram classificados os testes positivos, negativos e indeterminados de ZIKV e informações sobre como medir fatores de confusão e resultados de interesse. Esses dados serão utilizados para explicar adequadamente a heterogeneidade no nível do estudo e a margem de erro nos modelos estatísticos desenvolvidos para os objetivos 1-4 do ZIKV IPD-MA.

A pesquisa de metadados pode levar até uma hora para ser concluída e exigirá informações de diferentes membros da sua equipe de pesquisa. Entre em contato com Lauren Maxwell em maxwelll@who.int, se tiver dúvidas ao concluir a pesquisa de metadados.

Basic study information

Your first name

____

Your last name

____

Study name

____

For peer review only

Study country	◯ Brazil<br>◯ Colombia<br>◯ Non-continental US<br>◯ Continental US<br>◯ Mexico<br>◯ Honduras<br>◯ Spain<br>◯ Thailand<br>◯ Nicaragua<br>◯ Philippines<br>◯ France<br>◯ Guyana<br>◯ Suriname<br>◯ Greece<br>◯ Grenada<br>◯ Italy<br>◯ UK<br>◯ Trinidad and Tobago<br>◯ Jamaica<br>◯ Singapore<br>◯ Kenya<br>◯ Other<br>◯ Multi-country study
For multi-country studies, specify the included countries	__________________________________
Specify other country	___________________________
Study state	___________________________<br>(Write N/A if you have a country-level study)
Study city	___________________________<br>(Write N/A if you have a state- or country-level study)
Number of study sites	___________________________
Provide city and state for each site, if relevant<br>Use a semi-colon to separate sites and a comma to separate the city and state<br><br>e.g. New York City, New York; San Francisco, California	__________________________________
Does your study agree, in principle, to share de-identified subject-level data for the ZIKV IPD Consortium IPD-MA?	◯ Yes<br>◯ No<br>◯ Considering
Study Type	◯ Cohort<br>◯ Active surveillance system<br>◯ Case cohort<br>◯ Other

For peer review only

Specify other study type

____

What is the source population for your study?

Select all that apply

- ☐ Hospital or health care center
- ☐ Community
- ☐ Travelers
- ☐ Other

What are the other source population(s) for your study?

____

Was your study funded by any of the following groups?

Select all that apply

- ☐ European Union
- ☐ GlOPID-R
- ☐ WHO
- ☐ PAHO
- ☐ Africa CDC
- ☐ E-CDC
- ☐ US-CDC
- ☐ Bill and Melinda Gates Foundation
- ☐ NIH/NIAID
- ☐ Wellcome Trust
- ☐ DfID
- ☐ USAID
- ☐ DFG
- ☐ Other
- ☐ Study did not receive funding from any of these groups

Which other group funded your study?

____

When did your study begin enrolling women?

____

When did your study finish enrolling women?

____
(Specify the date you estimate you will stop enrolling women if enrollment is ongoing)

How many women did your study PLAN to enroll?

____

How many women has your study ACTUALLY enrolled to date?

____

How many ZIKV+ pregnant women has your study enrolled to date?

____

How many infants or children did you study include?

____

For multi-site studies, have all sites applied the same laboratory and clinical protocols?

- ◯ Same laboratory protocol
- ◯ Same clinical protocol
- ◯ Same laboratory & clinical protocols
- ◯ Sites apply different laboratory and clinical protocols

For peer review only

Describe cross-site differences in laboratory protocols

Describe cross-site differences in clinical protocols

For peer review only

Enrollment criteria & recruitment methods

Describe the inclusion criteria for the cohort
____

Describe the exclusion criteria for the cohort
____

Describe the inclusion criteria for the cohort from which the case-cohort was sampled
____

Describe the exclusion criteria for the cohort from which the case-cohort was sampled
____

What was the case definition used to identify cases for the case-cohort study?
____

How were women sampled from the cohort to participate in the case-cohort study?
____

Which groups were recruited to the study?

- ◯ pregnant women
- ◯ recently delivered women with infants with evidence of ZIKV infection
- ◯ recently delivered women, regardless of their infants' ZIKV status
- ◯ women of reproductive age, regardless of their pregnancy status
- ◯ other

Which other groups were recruited to the study?
____

How was the pregnancy identified?

Select all that apply

- ☐ Self-reported
- ☐ 1st obstetric visit (blood or urine test)
- ☐ Urine test (self-administered at home)
- ☐ Urine test (at health care facility)
- ☐ Blood test
- ☐ Ultrasound
- ☐ Varies at the participant level

How was gestational age determined?

Select all that apply

- ☐ LMP (last menstrual period)
- ☐ US (ultrasound)

Was ZIKV the original disease of interest for your cohort?

- ◯ Yes
- ◯ No, was originally a DENV study
- ◯ No, was originally an influenza study
- ◯ No, was formed for another purpose

What was the initial purpose of your cohort?
____

For peer review only - http://bmjopen.bmj.com/site/about/guidelines.xhtml

If your study wasn't originally designed to measure ZIKV, which symptoms were used to decide whether to test women for ZIKV?<br><br>Select all that apply	☐ Fever<br>☐ Rash<br>☐ Joint pain<br>☐ Conjunctivitis<br>☐ Rash OR joint pain OR conjunctivitis OR fever<br>☐ Other<br>☐ Symptoms not required for testing
What other symptoms were used to decide whether to test women for ZIKV?	__________________________________
How was enrollment of women or infants into the study related to their ZIKV status?<br><br>Select all that apply	☐ women's ZIKV assay<br>☐ women's ZIKV symptoms<br>☐ women's ZIKV assay OR ZIKV symptoms<br>☐ infant's ZIKV assay<br>☐ infant's ZIKV symptoms<br>☐ infant's ZIKV assay OR ZIKV symptoms<br>☐ women's presumed exposure (travel or sexual)<br>☐ other<br>☐ enrollment not related to ZIKV status of women or infants
In what other way was enrollment of women or infants into the study related to their ZIKV status?	__________________________________
How was study enrollment of women related to their ZIKV assay result or their infant's ZIKV assay result?	◯ Study only enrolled ZIKV PCR pos (+) pregnant women<br>◯ Study only enrolled ZIKV PCR pos (+) or probable pregnant women<br>◯ Study only enrolled ZIKV PCR neg (-) pregnant women<br>◯ Study only enrolled women with ZIKV PCR pos (+) infants, irregardless of women's ZIKV infection status<br>◯ Study only enrolled women with ZIKV PCR pos (+) or ZIKV probable infants, irregardless of women's ZIKV infection status<br>◯ Study only enrolled recently delivered ZIKV PCR pos (+) women with ZIKV PCR pos (+) or ZIKV probable infants<br>◯ Other
Describe other ZIKV assay result-related inclusion criteria	__________________________________
What ZIKV-related symptoms did your study consider when enrolling women?	◯ Fever only<br>◯ Rash only<br>◯ Fever + rash<br>◯ Fever or rash<br>◯ Fever + rash + joint pain<br>◯ Fever + rash + joint pain + conjunctivitis<br>◯ Other<br>◯ Fever + rash + other
Describe other ZIKV symptom-related inclusion criteria	__________________________________

For peer review only

What ZIKV-related symptoms in infants did your study consider when enrolling infants and/or their mothers?	◯ Fever only<br>◯ Rash only<br>◯ Fever + rash<br>◯ Fever or rash<br>◯ Fever + rash + joint pain<br>◯ Fever + rash + joint pain + conjunctivitis<br>◯ Other<br>◯ Fever + rash + other
What other ZIKV-related symptoms in infants did your study consider when enrolling participants?	__________________________________
Describe any additional ZIKV-related inclusion criteria	__________________________________
Describe any additional non-ZIKV related inclusion criteria (e.g. HIV co-infected, residence in a certain neighborhood, etc).	__________________________________
List any study exclusion criteria	__________________________________
What was your mechanism for identifying potential participants for your study?<br><br>Select all that apply	☐ Referrals from active surveillance system<br>☐ Referrals from hospital or clinic<br>☐ Preexisting cohort<br>☐ Other
What was your mechanism for identifying potential participants for your study?	__________________________________
Were potential participants referred to your study using a surveillance definition?	◯ Yes<br>◯ No
What was the surveillance definition used to refer participants to the study or surveillance system?	__________________________________
Where did recruitment take place?<br><br>Select all that apply	☐ Primary healthcare facility<br>☐ Tertiary healthcare facility<br>☐ Any healthcare facility (not specified by the study)<br>☐ Community<br>☐ Other
Where else did recruitment take place?	__________________________________
When during pregnancy were women enrolled?	◯ Prior to pregnancy<br>◯ During 1st trimester only<br>◯ During 1st or 2nd trimester only<br>◯ Anytime during pregnancy<br>◯ First obstetric visit<br>◯ Other

For peer review only

At what other time were women enrolled?	__________________________________
Who recruited potential participants?	◯ Health care facility staff<br>◯ Project interviewer (not health care facility staff member)<br>◯ Community health worker<br>◯ Other
Which other group enrolled research participants?	__________________________________

For peer review only

Follow up schedules for pregnant women and infants

Was the number and timing of visits during pregnancy the same for ZIKV+ and ZIKV- pregnant women?

◯ Yes
◯ No
◯ Study only followed ZIKV + pregnant women
◯ Study did not follow any pregnant women

How many visits did the study plan to have with pregnant women?

____
(If there is a range for the number of visits, please indicate the upper limit of the range)

At what time during pregnancy was the visit/were the visits planned to occur?

____
(e.g. every trimester, every month, months 1, 6, 9, etc.)

How many visits did the study plan to have with ZIKV+ pregnant women?

____
(If there is a range for the number of visits, please indicate the upper limit of the range)

At what time during pregnancy was the visit/were the visits scheduled to occur with ZIKV+ pregnant women?

____
(e.g. every trimester, every month, months 1, 6, 9, etc.)

How many visits did the study plan to have with ZIKV- pregnant women?

____
(If there is a range for the number of visits, please indicate the upper limit of the range)

At what time during pregnancy was the visit/were the visits scheduled to occur with ZIKV- pregnant women?

____
(e.g. every trimester, every month, months 1, 6, 9, etc.)

Other than timing and duration of follow-up, describe any differences in the types of assessments conducted with ZIKV+ and ZIKV- pregnant women:

____

Did the study follow infants after birth?

◯ Yes
◯ No

Was the number and timing of follow-up visits the same for the infants/children of ZIKV+ and ZIKV- pregnant women?

◯ Yes
◯ No
◯ Study did not follow infants/children of ZIKV- pregnant women

How many visits did the study plan to have with infants/children?

____
(If there is a range for the number of visits, please indicate the upper limit of the range)

Up until what age (in months) did your study intend to follow infants/children?

____

For peer review only

At what ages was the visit/were the visits with infants/children scheduled to occur?	______________________ (Specify whether ages listed in months or years)
How many visits did the study plan to have with infants/children from ZIKV+ mothers?	______________________ (If there is a range for the number of visits, please indicate the upper limit of the range)
Up until what age (in months) did your study plan to follow infants/children of ZIKV+ mothers?	______________________
At what ages was the visit/were the visits with infants/children of ZIKV+ mothers scheduled to occur?	______________________ (Specify whether ages listed in months or years)
How many visits did the study plan to have with infants/children of ZIKV- mothers?	______________________ (If there is a range for the number of visits, please indicate the upper limit of the range)
Up until what age (in months) did your study plan to follow infants/children of ZIKV- mothers?	______________________
At what ages was the visit/were the visits with infants/children of ZIKV- mothers scheduled to occur?	______________________ (Specify whether ages listed in months or years)
Other than timing and duration of follow-up, describe any differences in the types of assessments conducted with infants/children of ZIKV+ and ZIKV- pregnant women:	______________________

For peer review only

ZIKV symptoms in pregnant women and infants

Which of the following were measured to determine symptomatic ZIKV infection in women?

Select all that apply

- ☐ Fever
- ☐ Rash
- ☐ Joint pain
- ☐ Conjunctivitis
- ☐ Rash OR joint pain OR conjunctivitis OR fever
- ☐ Other

What else was measured to determine symptomatic ZIKV infection in women?

____

How was fever measured in women?

Select all that apply

- ☐ Underarm
- ☐ Sublingual
- ☐ Otic
- ☐ Rectal
- ☐ Self reported or subjective (any site)

How did your study define fever in women? (degrees Celsius)

____
(Degrees Celsius)

How was fever measured in infants?

Select all that apply

- ☐ Underarm
- ☐ Sublingual
- ☐ Otic
- ☐ Rectal
- ☐ Self reported or subjective (any site)

How did your study define fever in infants? (degrees Celsius)

____
(Degrees Celsius)

For peer review only

Ascertainment of maternal ZIKV infection

What methods were used to assess maternal ZIKV status?<br><br>Select all that apply	☐ Clinical case definition<br>☐ Molecular diagnosis (e.g. RT-PCR, other NAAT)<br>☐ Immunoassay (e.g. ELISA, IFA, PRNT, microneutralization)<br>☐ Rapid diagnostic test (RDT)<br>☐ Other
What other method was used to assess maternal ZIKV status?	__________________________________
Did you use different assays, different brands of the same assay, or different versions of the same brand of assay at different study sites to ascertain maternal ZIKV status or over time within the study?<br><br>Select all that apply	☐ Yes, different versions of the same brand of assay over time<br>☐ Yes, different brands of the same type of assay over time<br>☐ Yes, different types of assays over time<br>☐ Yes, different versions of the same brand of assay at different sites<br>☐ Yes, different brands of the same type of assay at different sites<br>☐ Yes, different types of assays at different sites<br>☐ No, same tests applied throughout the project
Which different assay(s) did you use over time or across sites?	__________________________________
Which different brands of the assay did you use over time or across sites?	__________________________________
Which different versions of the brand of assay did you use over time or across sites?	__________________________________

For peer review only

Ascertainment of maternal ZIKV infection - Immunoassay

ZIKV immunoassay analyte<br><br>Select all that apply	☐ IgM<br>☐ IgG<br>☐ IgA<br>☐ neutralizing antibodies<br>☐ Other
Other ZIKV immunoassay analyte	__________________________________
ZIKV immunoassay type<br><br>Select all that apply	☐ ELISA<br>☐ IFA<br>☐ MIA<br>☐ PRNT<br>☐ Other
Specify other type(s) or ZIKV immunoassay(s) performed	__________________________________

For peer review only

ZIKV immunoassay brand name<br><br>Select all that apply	☐ In house-developed assay<br>☐ Zika MAC-ELISA (CDC)<br>☐ ZIKV Detect 2.0 IgM Capture ELISA Kit (InBios International Inc)<br>☐ Zika IgM ELISA (EUROIMMUN)<br>☐ Zika IgM + IgA ELISA (EUROIMMUN)<br>☐ Zika IgG ELISA (EUROIMMUN)<br>☐ ZIKV IgM IFA (EUROIMMUN)<br>☐ ZIKV IgG IFA (EUROIMMUN)<br>☐ Arbovirus Fever Mosaic 2 IgG + IgM IFA ZIKV-DENV-CHIKV (EUROIMMUN)<br>☐ ADVIA Centaur Zika Test (Siemens Healthcare Diagnostics)<br>☐ DPP Zika IgM Assay System (Chembio Diagnostic Systems Inc)<br>☐ DPP Zika IgM/IgG System (Chembio Diagnostic Systems Inc)<br>☐ DPP Zika/Chikungunya/Dengue IgM/IgG Combination Assay (Chembio Diagnostic Systems Inc)<br>☐ LIAISON XL Zika Capture IgM II assay (DiaSorin S.p.A.)<br>☐ STANDARD Q Zika IgM/IgG test (SD Biosensor)<br>☐ STANDARD E Zika IgM ELISA (SD Biosensor)<br>☐ STANDARD Q ZIKV/DENV/CHIKV Fast Quad (SD Biosensor<br>☐ STANDARD Q ZIKV/DENV/CHIKV/YFV IgM Quad (SD Biosensor)<br>☐ Tell Me Fast Zika IgG/IgM Antibody Rapid Test (Biocan Diagnostics Inc)<br>☐ Tell Me Fast zika/dengue/chikungunya combination rapid tests (Biocan Diagnostics Inc)<br>☐ ZIKV-DENV-CHIKV IFA IgG (Vircell)<br>☐ ZIKV-DENV-CHIKV IFA IgM (Vircell)<br>☐ Human Anti-Zika ELISA Kits (Alpha Diagnostic International)<br>☐ anti-Zika virus IgM ELISA kit (µ-capture) (AbCam)<br>☐ anti-Zika virus IgG ELISA kit (AbCam)<br>☐ Zika Virus IgM ELISA Assay Kit (Eagle Biosciences)<br>☐ Zika Virus IgG ELISA Assay Kit (Eagle Biosciences)<br>☐ Human Anti-Zika Virus IgG ELISA Kit (R&D Systems)<br>☐ ZIKV IgG ELISA kit (DIA.PRO Diagnostic Bioprobes Srl)<br>☐ ZIKV IgM ELISA kit (DIA.PRO Diagnostic Bioprobes Srl)<br>☐ NovaLisa Zika Virus IgM µ-capture ELISA (NovaTec Immunodiagnostica GmbH)<br>☐ recomLine Tropical Fever IgG (MIKROGEN Diagnostik)<br>☐ recomLine Tropical Fever IgM (MIKROGEN Diagnostik)<br>☐ Other
Which other immunoassays were used to diagnose maternal ZIKV infection?	____________________________________
What type of assay is the house assay?<br><br>Select all that apply	☐ ELISA<br>☐ IFA<br>☐ MIA<br>☐ PRNT<br>☐ Other
What other type of assay is the in house assay?	____________________________________

For peer review only

What is the analyte measured by the in house assay?<br><br>Select all that apply	☐ IgM<br>☐ IgG<br>☐ IgA
What is the antigenic target for the in house assay?<br><br>Select all that apply	☐ NS1<br>☐ envelope protein<br>☐ NS5<br>☐ Other
What is the antigenic target for the in house assay?	__________________________________
For the in house ZIKV immunoassay, describe the testing procedure, including: incubation period, timing, type of cells, buffers, method to detect binding antibodies (e.g., optical density or secondary antibody detection), and methods to stop the enzymatic reaction	__________________________________
Sample used for ZIKV immunoassay<br><br>Select all that apply	☐ Serum/plasma<br>☐ Whole blood<br>☐ Other
Specify other sample used for ZIKV immunoassay	__________________________
ZIKV ELISA tested in paired/subsequent samples	◯ Yes<br>◯ No
At what temperature was the ZIKV ELISA test stored? (degrees Celsius)	__________________________
At what temperature(s) was/were the ZIKV ELISA sample(s) stored? (degrees Celsius)	__________________________________<br>(Include sample type for each temperature)
In what volume(s) was/were the ZIKV ELISA sample aliquot(s) stored? (mL)	__________________________________<br>(Include sample type with each volume)
Did your study deviate from the directions provided in the ZIKV ELISA commercial kit?	◯ Yes<br>◯ No<br>◯ Study did not use a commercial ZIKV ELISA kit
Describe any deviations from the procedure recommended by the commercial ELISA kit, including: incubation period, timing, type of cells, buffers, method to detect bounding antibodies, and methods to stop the enzymatic reaction	__________________________________
Describe any additional conditions for ZIKV ELISA test administration (e.g. paired samples within 21 days of fever onset; count with more than 2ul of serum from the febrile phase, etc.)	__________________________________

For peer review only

Where was the ZIKV ELISA test performed?<br><br>Select all that apply	☐ Lab at the same health care facility where study is conducted<br>☐ Study-specific lab<br>☐ Ministry of Health reference lab<br>☐ Private external lab<br>☐ Other
Specify other lab where ZIKV ELISA was performed	__________________________________

For peer review only

Ascertainment of maternal ZIKV infection - ELISA

ZIKV ELISA cut-off point
____

Was a positive control from the outbreak included for the ZIKV ELISAs?
◯ Yes
◯ No

Was a negative control included with every run of ZIKV ELISAs?
◯ Yes
◯ No

Definition of POSITIVE ZIKV ELISA
____

Definition of NEGATIVE ZIKV ELISA
____

Definition of INCONCLUSIVE ZIKV ELISA
____

Source of definition used to interpret ZIKV ELISA test results

Select all that apply
☐ Manufacturer
☐ Ministry of Health reference lab
☐ WHO (global or local office e.g: PAHO)
☐ US-CDC
☐ Consensus/study researchers
☐ Other

Specify other source of definition used to interpret ZIKV ELISA test results
____

How many ZIKV ELISAs did the study plan to conduct in each pregnant woman?
____

Would your study be able to provide the P/N ratio, titer, or other quantitative readout for ZIKV ELISAs to inform the measurement error models?
◯ Yes
◯ No

For peer review only

Ascertainment of maternal ZIKV infection - IFA

ZIKV IFA cut-off value

____

Was a positive control from the outbreak included for the ZIKV IFA?

◯ Yes
◯ No

Was a negative control included with every run of the ZIKV IFA?

◯ Yes
◯ No

Definition of POSITIVE ZIKV IFA

____

Definition of NEGATIVE ZIKV IFA

____

Definition of INCONCLUSIVE ZIKV IFA

____

Source of definition used to interpret ZIKV IFA test results

Select all that apply

☐ Manufacturer
☐ Ministry of Health reference lab
☐ WHO (global or local office e.g: PAHO)
☐ Consensus/study researchers
☐ Other

Specify other source of definition for ZIKV IFA test results

____

Specify any additional inclusion or exclusion criteria for determining which samples to test for ZIKV using IFA

____

Will your study be able to provide the titer or optical density for ZIKV IFAs to inform the measurement error model?

◯ Yes
◯ No

For peer review only

Ascertainment of maternal ZIKV infection - MIA

ZIKV MIA cut-off value ________________________________

Was a positive control from the outbreak included for the ZIKV MIAs?
◯ Yes
◯ No

Was a negative control included with every run of ZIKV MIAs?
◯ Yes
◯ No

Definition of POSITIVE ZIKV MIA ________________________________

Definition of NEGATIVE ZIKV MIA ________________________________

Definition of INCONCLUSIVE ZIKV MIA ________________________________

Source of definition used to interpret ZIKV MIA test results

Select all that apply
☐ Manufacturer
☐ Ministry of Health reference lab
☐ WHO (global or local office e.g: PAHO)
☐ Consensus/study researchers
☐ Other

Specify other source of definition for ZIKV MIA test results ________________________________

Specify any additional inclusion or exclusion criteria for determining which samples to test for ZIKV using MIA ________________________________

Will your study be able to provide the titer for ZIKV MIAs to inform the measurement error model?
◯ Yes
◯ No

For peer review only

Ascertainment of maternal ZIKV infection - PRNT

Did you use the CDC protocol for the PRNT?
- ◯ Yes
- ◯ No

Describe the other protocol used for the PRNT
____

End-point PRNT titer
- ◯ PRNT50
- ◯ PRNT90
- ◯ Other

Which other end-point did you use for the PRNT titer?
____

Definition of a positive PRNT titer
- ◯ PRNT>10
- ◯ Other

Specify other definition of a positive PRNT titer
____

Source of definition used to interpret ZIKV PRNT test results

Select all that apply
- ☐ Manufacturer
- ☐ Ministry of Health reference lab
- ☐ WHO (global or local office e.g: PAHO)
- ☐ US-CDC
- ☐ Consensus/study researchers
- ☐ Other

Specify other source of definition used to interpret ZIKV PRNT test results
____

Definition of POSITIVE ZIKV PRNT
____

Definition of NEGATIVE ZIKV PRNT
____

Definition of INCONCLUSIVE PRNT (i.e. specific flavivirus could not be determined)
____

Specify any additional inclusion or exclusion criteria for determining which samples to test for ZIKV using PRNT
____

Where was the ZIKV PRNT test performed?

Select all that apply
- ☐ Lab at the same health care facility where study is conducted
- ☐ Study-specific lab
- ☐ Ministry of Health reference lab
- ☐ Private external lab
- ☐ Other

Specify other lab where ZIKV PRNT test was performed
____

For peer review only

How many ZIKV PRNTs were conducted with pregnant women? __________________________________

Will your study be able to provide the titer for ZIKV PRNTs to inform the measurement error model?
◯ Yes
◯ No

For peer review only

Ascertainment of maternal ZIKV infection - Molecular assay

Type of ZIKV molecular tests performed<br><br>Select all that apply	☐ conventional PCR<br>☐ rRT-PCR<br>☐ qRT-PCR<br>☐ Other NAAT
According to the study protocol, when were the ZIKV samples to be collected for molecular testing?<br><br>Select all that apply	☐ 3 days after fever onset<br>☐ 5 days after fever onset<br>☐ 7 days after fever onset<br>☐ 10 days after fever onset<br>☐ 14 days after fever onset<br>☐ Other
When else were the ZIKV samples to be collected for molecular testing?	______________________________
Sample used for ZIKV RT-PCR/PCR<br><br>Select all that apply	☐ Serum/plasma<br>☐ Whole blood<br>☐ Urine<br>☐ Saliva<br>☐ Breast milk<br>☐ Umbilical cord tissue<br>☐ Placental tissue<br>☐ Amniotic fluid<br>☐ Other
Specify other type of sample used for ZIKV RT-PCR/PCR	______________________________

For peer review only

ZIKV RT-PCR/PCR brand name<br><br>Select all that apply	☐ In house technique<br>☐ Zika Virus RNA Qualitative Real-Time RT-PCR (Quest Diagnostics)<br>☐ RealStar Zika Virus RT-PCR Kit U.S. (altona Diagnostics GmbH)<br>☐ RealStar Zika Virus RT-PCR Kit 1.0 (altona Diagnostics GmbH)<br>☐ Aptima Zika Virus assay (Hologic Inc)<br>☐ Zika Virus Real-time RT-PCR (Viracor Eurofins)<br>☐ VERSANT Zika RNA 1.0 Assay (kPCR) (Siemens Healthcare Diagnostics Inc)<br>☐ xMAP MultiFLEX Zika RNA Assay (Luminex Corporation)<br>☐ Sentosa SA ZIKV RT-PCR Test (Vela Diagnostics Inc)<br>☐ Zika Virus Detection by RT-PCR (ARUP Laboratories)<br>☐ Abbott RealTime Zika (Abbott Molecular Inc)<br>☐ Zika ELITe MGB Kit U.S. (ELITechGroup Molecular Diagnostics)<br>☐ Gene-RADAR Zika Virus Test (Nanobiosym Diagnostics Inc)<br>☐ TaqPath Zika Virus Kit (Thermo Fisher Scientific)<br>☐ CII-ArboViroPlex rRT-PCR assay (Columbia University)<br>☐ Trioplex Real-Time RT-PCR Assay (CDC)<br>☐ Liferiver Zika Virus (ZIKV) Real Time RT-PCR Kit (Shanghai ZJ Bio-Tech Co Ltd)<br>☐ careGENE Zika Virus RT-PCR Kit (WELLS BIO Inc)<br>☐ Zika Virus - Single Check (Genekam Biotechnology AG)<br>☐ FTD Zika virus (Fast Track Diagnostics)<br>☐ VIASURE Zika Virus Real Time PCR Detection Kit (Certest Biotec)<br>☐ Zika Virus Real-TM (Sacace Biotechnologies)<br>☐ Logix Smart Zika Test Kit (Co-Diagnostics Inc)<br>☐ Genesig Kits for ZIKV (Primerdesign Ltd)<br>☐ Genesig Multiplex Kit for Dengue, Chikungunya and Zika Virus (Primerdesign Ltd)<br>☐ AccuPower ZIKV (DENV, CHIKV) Multiplex Real-Time RT-PCR Kit (Bioneer)<br>☐ VIASURE Zika, Dengue & Chikungunya Real Time PCR Detection Kit (Certest Biotec)<br>☐ FTD Zika/Dengue/Chik (Fast-Track Diagnostics)<br>☐ DiaPlexQ ZCD (ZIKV, CHIKV, DENV) Detection Kit (SolGent Co Ltd)<br>☐ TaqMan Zika Virus Triplex Kit (Thermo Fisher Scientific)<br>☐ GenoAmp Trioplex Real-Time RT-PCR Zika/Den/Chiku (Medical Innovation Ventures)<br>☐ Other
Describe procedure for in house ZIKV RT-PCR/PCR	__________________________________
Specify other ZIKV RT-PCR/PCR brand name	__________________________________
Did the study obtain a measure of ZIKV viral load?	○ Yes<br>○ No
ZIKV PCR/RT-PCR cut-off point	____________________________

For peer review only

Was a negative control included with every run of ZIKV RT-PCR/PCRs?	◯ Yes<br>◯ No
Definition of POSITIVE ZIKV RT-PCR/PCR	________________________________
Definition of NEGATIVE ZIKV RT-PCR/PCR	________________________________
Definition of INCONCLUSIVE ZIKV RT-PCR/PCR	________________________________
Source of definition used to interpret ZIKV RT-PCR/PCR test results<br><br>Select all that apply	☐ Manufacturer<br>☐ Ministry of Health reference lab<br>☐ WHO (global or local office e.g: PAHO)<br>☐ US-CDC<br>☐ Consensus/study researchers<br>☐ Other
Specify other source of definition used to interpret ZIKV RT-PCR/PCR test results	__________________________
Where was the ZIKV RT-PCR/PCR test performed?<br><br>Select all that apply	☐ Lab at the same health care facility where study is conducted<br>☐ Study-specific lab<br>☐ Ministry of Health reference lab<br>☐ Private external lab<br>☐ Other
Specify other lab where ZIKV RT-PCR/PCR test was performed	__________________________
Specify any additional inclusion or exclusion criteria for determining which samples to test for ZIKV using RT-PCR/PCR	________________________________
How many ZIKV PCRs were conducted with pregnant women?	__________________________
Would your study be able to provide a Ct value for ZIKV PCRs to inform the measurement error models?	◯ Yes<br>◯ No

For peer review only

ZIKV testing algorithm & test interpretation - Maternal ZIKV infection

Describe the testing algorithm for defining a confirmed ZIKV+ test in pregnant women	__________________________
Describe the testing algorithm for defining a confirmed ZIKV- test in pregnant women	__________________________
What tests were used to confirm ZIKV infection in pregnant women?<br><br>Select all that apply	☐ ZIKV IgM - 1 sample<br>☐ ZIKV IgM - paired samples (seroconversion or increased titers)<br>☐ ZIKV IgM (1 sample) + IgG<br>☐ ZIKV IgM (paired samples) + IgG<br>☐ RT-PCR or other NAAT<br>☐ PRNT > 10<br>☐ Other
Specify other test or combination of tests used to confirm ZIKV infection in pregnant women	__________________________
Considering any relevant clinical and laboratory criteria, what was the study's definition of a CONFIRMED maternal ZIKV Infection	__________________________
Considering any relevant clinical and laboratory criteria, what was the study's definition of a PROBABLE maternal ZIKV Infection	__________________________
Considering any relevant clinical and laboratory criteria, what was the study's definition of a NEGATIVE/UNLIKELY maternal ZIKV Infection	__________________________
Source of ZIKV test result categorization<br><br>Select all that apply	☐ Manufacturer<br>☐ Ministry of Health reference lab<br>☐ WHO (global or local office e.g: PAHO)<br>☐ Consensus/study researchers<br>☐ Other
Specify other source of ZIKV test result categorization	__________________________

For peer review only

Changes in maternal ZIKV assessment over time

Which criteria did you use to infer maternal ZIKV status or fetal exposure to ZIKV?<br><br>Select all that apply	☐ Maternal clinical criteria<br>☐ Maternal laboratory criteria<br>☐ Infant clinical criteria<br>☐ Infant laboratory criteria
Which clinical criteria did you apply initially to diagnose maternal infection? (Clinical Definition 1)	__________________________________
Did your clinical criteria for diagnosing maternal infection change over time?	◯ No<br>◯ Yes - changed 1x<br>◯ Yes - changed more than 1x
If your clinical criteria changed over time, which criteria did you apply subsequently? (Clinical Definition 2)	__________________________________
When did you begin using the revised criteria? (Clinical Definition 2)	___________________________
What was the third set of criteria you applied? (Clinical Definition 3)	__________________________________
When did you begin using the third set of clinical criteria? (Clinical Definition 3)	___________________________
What laboratory criteria was used initially to diagnose maternal ZIKV infection? (Laboratory Definition 1)	__________________________________
Did your laboratory criteria for diagnosing maternal infection change over time?	◯ No<br>◯ Yes - changed 1x<br>◯ Yes - changed more than 1x
Which laboratory criteria did you apply subsequently? (Laboratory Definition 2)	__________________________________
When did you begin using the revised lab criteria? (Laboratory Definition 2)	___________________________
What was the third set of laboratory criteria you applied? (Laboratory Definition 3)	__________________________________
When did you begin using the third set of lab criteria? (Laboratory Definition 3)	___________________________
Were maternal samples retested using the new laboratory criteria?	◯ Yes<br>◯ No
If testing was performed for more than one arbovirus, including ZIKV, were they assessed simultaneously (i.e. multiplex testing) or separately?	◯ Yes, arboviruses tested simultaneously (multiplex)<br>◯ No, arboviruses tested separately

For peer review only

Ascertainment of maternal DENV infection

Did the study assess pregnant women's DENV status?	◯ Yes<br>◯ No
What methods were used to assess maternal DENV status?<br><br>Select all that apply	☐ Clinical case definition<br>☐ Molecular diagnosis (e.g. RT-PCR, other NAAT)<br>☐ Immunoassay (e.g. ELISA, IFA, PRNT, microneutralization)<br>☐ Rapid diagnostic test (RDT)<br>☐ Other
What other method was used to assess maternal DENV status?	__________________________________
Were ZIKV and DENV assessed using separate tests?	◯ DENV assessed separately from ZIKV<br>◯ DENV and ZIKV assessed using multiplex testing
At any time, did your study screen for DENV and only test DENV (-) participants for ZIKV?	◯ Yes, at some point study screened for DENV first, only tested DENV negative participants for ZIKV<br>◯ No, study always tested for DENV and ZIKV simultaneously
If yes, when did your study BEGIN only administering ZIKV tests to DENV- participants?	___________________________
If yes, when did your study STOP only administering ZIKV tests to DENV- participants?	___________________________

For peer review only

Ascertainment of maternal DENV infection - Immunoassay

DENV immunoassay analyte<br><br>Select all that apply	☐ IgM<br>☐ IgG<br>☐ IgA<br>☐ neutralizing antibodies<br>☐ Other
Other DENV immunoassay analyte	__________________________________
DENV immunoassay type<br><br>Select all that apply	☐ ELISA<br>☐ IFA<br>☐ MIA<br>☐ PRNT<br>☐ Other
Specify other type(s) or DENV immunoassay(s) performed	__________________________________
DENV immunoassay brand name<br><br>Select all that apply	☐ In house technique<br>☐ MAC-ELISA<br>☐ DENV Detect ELISA IgM/IgG (InBios International, United States)<br>☐ Dengue DxSelect IgM/IgG (Focus Diagnostics, USA)<br>☐ PATHOZYME-DENGUE IgM/IgG (Omega Diagnostics, UK)<br>☐ Dengue IgM/IgG Capture ELISA (Panbio Diagnostics, Australia)<br>☐ Dengue IgM/IgG Capture ELISA (Standard Diagnostics, South Korea)<br>☐ Platelia Dengue IgM/IgG ELISA" (Bio-Rad, France)<br>☐ SD Bioline Dengue IgM/IgG (Alere, United States)<br>☐ Arbovirus Fever Mosaic 2 IgG + IgM IFA ZIKV-DENV-CHIKV (EUROIMMUN)<br>☐ recomLine Tropical Fever IgG (MIKROGEN Diagnostik)<br>☐ recomLine Tropical Fever IgM (MIKROGEN Diagnostik)<br>☐ DPP Zika/Chikungunya/Dengue IgM/IgG Combination Assay (Chembio Diagnostic Systems Inc)<br>☐ STANDARD Q ZIKV/DENV/CHIKV Fast Quad (SD Biosenso<br>☐ STANDARD Q ZIKV/DENV/CHIKV/YFV IgM Quad (SD Biosensor)<br>☐ Tell Me Fast zika/dengue/chikungunya combination rapid tests (Biocan Diagnostics Inc)<br>☐ Other
Which other immunoassays were used to diagnose maternal DENV infection?	__________________________________
What type of assay is the in house assay?<br><br>Select all that apply	☐ ELISA<br>☐ IFA<br>☐ MIA<br>☐ PRNT<br>☐ Other
What type of assay is the in house assay?	__________________________________

For peer review only

What is the analyte measured by the in house assay?<br><br>Select all that apply	☐ IgM<br>☐ IgG<br>☐ IgA
What is the antigenic target for the in house assay<br><br>Select all that apply	☐ NS1<br>☐ envelope protein<br>☐ NS5<br>☐ Other
What is the antigenic target for the in house assay	__________________________________
For the in house DENV immunoassay, describe the testing procedure, including: incubation period, timing, type of cells, buffers, method to detect binding antibodies (e.g., optical density or secondary antibody detection), and methods to stop the enzymatic reaction	__________________________________
Sample used for DENV immunoassay<br><br>Select all that apply	☐ Serum/plasma<br>☐ Whole blood<br>☐ Urine<br>☐ Saliva<br>☐ Breast milk<br>☐ Umbilical cord tissue<br>☐ Placental tissue<br>☐ Amniotic fluid<br>☐ Other
Specify other type of sample used for DENV immunoassay	____________________________

For peer review only

Ascertainment of maternal DENV infection - ELISA

DENV ELISA cut-off value	__________________________________
Was a positive control from the outbreak included for the DENV ELISAs?	◯ Yes<br>◯ No
Was a negative control included with every run of DENV ELISAs?	◯ Yes<br>◯ No
Definition of POSITIVE DENV ELISA	__________________________________
Definition of NEGATIVE DENV ELISA	__________________________________
Definition of INCONCLUSIVE DENV ELISA	__________________________________
Source of definition used to interpret DENV ELISA test results<br><br>Select all that apply	☐ Manufacturer<br>☐ Ministry of Health reference lab<br>☐ WHO (global or local office e.g: PAHO)<br>☐ Consensus/study researchers<br>☐ Other
Specify other source of definition for DENV ELISA test results	__________________________________
Specify any additional inclusion or exclusion criteria for determining which samples to test for DENV using ELISA	__________________________________
Would your study be able to provide the P/N ratio, titer, or other quantitative readout for DENV ELISAs to inform the measurement error models?	◯ Yes<br>◯ No

For peer review only

Ascertainment of maternal DENV infection - IFA

DENV IFA cut-off value ________________________

Was a positive control from the outbreak included for the DENV IFA?
- ◯ Yes
- ◯ No

Was a negative control included with every run of DENV IFA?
- ◯ Yes
- ◯ No

Definition of POSITIVE DENV IFA ________________________

Definition of NEGATIVE DENV IFA ________________________

Definition of INCONCLUSIVE DENV IFA ________________________

Source of definition used to interpret DENV IFA test results

Select all that apply
- ☐ Manufacturer
- ☐ Ministry of Health reference lab
- ☐ WHO (global or local office e.g: PAHO)
- ☐ Consensus/study researchers
- ☐ Other

Specify other source of definition for DENV IFA test results ________________________

Specify any additional inclusion or exclusion criteria for determining which samples to test for DENV using IFA ________________________

Will your study be able to provide the titer or optical density for DENV IFAs to inform the measurement error model?
- ◯ Yes
- ◯ No

For peer review only

Ascertainment of maternal DENV infection - MIA

DENV MIA cut-off value ____________________

Was a positive control from the outbreak included for the DENV MIAs?
- ◯ Yes
- ◯ No

Was a negative control included with every run of DENV MIAs?
- ◯ Yes
- ◯ No

Definition of POSITIVE DENV MIA ____________________

Definition of NEGATIVE DENV MIA ____________________

Definition of INCONCLUSIVE DENV MIA ____________________

Source of definition used to interpret DENV MIA test results

Select all that apply
- ☐ Manufacturer
- ☐ Ministry of Health reference lab
- ☐ WHO (global or local office e.g: PAHO)
- ☐ Consensus/study researchers
- ☐ Other

Specify other source of definition for DENV MIA test results ____________________

Specify any additional inclusion or exclusion criteria for determining which samples to test for DENV using MIA ____________________

Will your study be able to provide the titer for DENV MIAs to inform the measurement error model?
- ◯ Yes
- ◯ No

For peer review only

Ascertainment of maternal DENV infection - Immunoassay - PRNT

Did you use the CDC protocol for the DENV PRNT?	◯ Yes<br>◯ No
Describe the other protocol used for the DENV PRNT?	__________________________________
Did your study simultaneously perform PRNT for ZIKV and DENV?	◯ Yes<br>◯ No
End-point PRNT titer	◯ PRNT50<br>◯ PRNT90<br>◯ Other
Which other end-point did you use for the PRNT titer?	___________________________
Definition of a positive PRNT titer	◯ PRNT>10<br>◯ Other
Other definition of a positive PRNT titer	___________________________
Definition of POSITIVE DENV PRNT	__________________________________
Definition of NEGATIVE DENV PRNT	__________________________________
Definition of INCONCLUSIVE DENV PRNT	__________________________________
Source of definition used to interpret DENV PRNT test results<br><br>Select all that apply	☐ Manufacturer<br>☐ Ministry of Health reference lab<br>☐ WHO (global or local office e.g: PAHO)<br>☐ US-CDC<br>☐ Consensus/study researchers<br>☐ Other
Specify other source of definition used to interpret DENV PRNT test results	___________________________
Specify any additional inclusion or exclusion criteria for determining which samples to test for DENV using PRNT	__________________________________
Where was the DENV PRNT test performed?<br><br>Select all that apply	☐ Lab at the same health care facility where study is conducted<br>☐ Study-specific lab<br>☐ Ministry of Health reference lab<br>☐ Private external lab<br>☐ Other

For peer review only

Specify other lab where DENV PRNT test was performed	__________________________
Will your study be able to provide the titer for DENV PRNTs to inform the measurement error model?	◯ Yes<br>◯ No
Can your study provide the full differences between the ZIKV and DENV titers or information about the magnitude of the difference? (e.g. is the difference 4 fold or greater? Is the difference 1 or less than 1?)	◯ Yes<br>◯ No<br>◯ Unsure

For peer review only

Ascertainment of maternal DENV infection - Molecular assay

Type of DENV PCR/RT-PCR performed<br><br>Select all that apply	☐ PCR (conventional or standard)<br>☐ qRT-PCR<br>☐ rRT-PCR<br>☐ No DENV PCR performed
DENV PCR/RT-PCR brand name<br><br>Select all that apply	☐ In house procedure<br>☐ CDC DENV-1-4 rRT-PCR Multiplex and Trioplex rRT-PCR Assays (CDC-USA)<br>☐ CDC DENV-1-4 Real-Time RT-PCR Multiplex Assay (CDC-USA)<br>☐ CDC Trioplex rRT-PCR assay (CDC-USA)<br>☐ Dengue Fever Virus & Chikungunya Virus Real Time RT-PCR Kit (Creative Biogene)<br>☐ Dengue Virus General-type Real Time RT-PCR Kit (Creative Biogene)<br>☐ RealStar Dengue RT-PCR (Altona Diagnostics-Hamburg,Germany)<br>☐ GenoAmp Trioplex Real-Time RT-PCR Zika/Den/Chiku (Medical Innovation Ventures)<br>☐ GenoAmp Real-Time RT-PCR Dengue (Serotyping of Dengue 1-Dengue 4) (Medical Innovation Ventures)<br>☐ RT-PCR kit (RealArt; artus/Qiagen-Germany)<br>☐ SimplexaTMdengue RT-PCR assay (Focus Diagnostics-Cypress, CA)<br>☐ Dengue virus general type real-time RT-PCR kit LiferiverTM (Shanghai ZJ Bio-Tech Co-China)<br>☐ Geno-Sen's dengue 1-4 real-time RT-PCR kit (Genome Diagnostics Pvt-New Delhi, India)<br>☐ CII-ArboViroPlex rRT-PCR assay (Columbia University)<br>☐ AccuPower ZIKV (DENV, CHIKV) Multiplex Real-Time RT-PCR kit (Bioneer)<br>☐ VIASURE Zika, Dengue & Chikungunya Real Time PCR Detection Kit (Certest Biotec)<br>☐ FTD Zika/Dengue/Chik (Fast-Track Diagnostics)<br>☐ DiaPlexQ ZCD (ZIKV, CHIKV, DENV) Detection Kit (SolGent Co Ltd)<br>☐ TaqMan Zika Virus Triplex Kit (Thermo Fisher Scientific)<br>☐ Genesig Multiplex Kit for Dengue, Chikungunya and Zika Virus (Primerdesign Ltd)<br>☐ Other
Describe procedure for in house DENV PCR/RT-PCR	__________________________________
Specify other DENV PCR/RT-PCR used	__________________________________
Sample used for DENV PCR/RT-PCR<br><br>Select all that apply	☐ Serum/plasma<br>☐ Whole blood<br>☐ Urine<br>☐ Saliva<br>☐ Breast milk<br>☐ Umbilical cord tissue<br>☐ Placental tissue<br>☐ Amniotic fluid<br>☐ Other

For peer review only

Specify other type of sample used for DENV PCR/RT-PCR	__________________
Did the study obtain a measure of DENV viral load?	◯ Yes<br>◯ No
DENV PCR/RT-PCR cut-off point	__________________
Was a negative control included with every run of DENV PCR/RT-PCRs?	◯ Yes<br>◯ No
Definition of POSITIVE DENV PCR/RT-PCR	__________________
Definition of NEGATIVE DENV PCR/RT-PCR	__________________
Definition of INCONCLUSIVE DENV PCR/RT-PCR	__________________
Source of definition used to interpret DENV PCR/RT-PCR test results<br><br>Select all that apply	☐ Manufacturer<br>☐ Ministry of Health reference lab<br>☐ WHO (global or local office e.g: PAHO)<br>☐ Consensus/study researchers<br>☐ Other
Specify other source of definition for DENV PCR/RT-PCR test results	__________________
Specify any additional inclusion or exclusion criteria for determining which samples to test for DENV using PCR/RT-PCR	__________________
Would your study be able to provide a Ct value for DENV PCR/RT-PCRs to inform the measurement error models?	◯ Yes<br>◯ No

For peer review only

DENV testing algorithm & qualitative definitions

What assay was used to confirm maternal DENV infection?

- ◯ NAAT (PCR) only
- ◯ NS-1 only
- ◯ IgM only
- ◯ IgG increase in paired samples
- ◯ IgM seroconversion in paired samples
- ◯ PRNT + any serologic test (IgM, IgG, or both)
- ◯ PCR or any NAAT + any serologic test (IgM, IgG or both)
- ◯ Other combination of assays

Specify other combination of assays used to confirm maternal DENV infection

____

Considering any relevant clinical and laboratory criteria, what was the study's definition of a CONFIRMED maternal DENV infection

____

Considering any relevant clinical and laboratory criteria, what was the study's definition of a PROBABLE maternal DENV infection

____

Considering any relevant clinical and laboratory criteria, what was the study's definition of a NEGATIVE/UNLIKELY maternal DENV infection?

____

For peer review only

Ascertainment of maternal CHIKV infection

Did the study conduct chikv-specific laboratory tests?	◯ Yes, chikv assessed separately from ZIKV<br>◯ Yes, chikv assessed using multiplex testing<br>◯ No, chikv not assessed
What methods were used to assess maternal CHIKV status?<br><br>Select all that apply	☐ Clinical case definition<br>☐ Molecular diagnosis (e.g. RT-PCR, other NAAT)<br>☐ Immunoassay (e.g. ELISA, IFA, PRNT, microneutralization)<br>☐ Rapid diagnostic test (RDT)<br>☐ Other
What other method was used to assess maternal CHIKV status?	__________________________________
CHIKV immunoassay analyte<br><br>Select all that apply	☐ IgM<br>☐ IgG<br>☐ IgA<br>☐ neutralizing antibodies<br>☐ Other
Other CHIKV immunoassay analyte	____________________________
CHIKV immunoassay type<br><br>Select all that apply	☐ ELISA<br>☐ IFA<br>☐ MIA<br>☐ PRNT<br>☐ Other
Specify other type(s) or CHIKV immunoassay(s) performed	____________________________

For peer review only

CHIKV immunoassay brand name<br><br>Select all that apply	☐ In house technique<br>☐ MAC-ELISA<br>☐ CHIK IgM micro-capture ELISA (IBL International -Germany)<br>☐ RecombiLISA CHIK IgM Test (CTK Biotech-USA/China)<br>☐ CHIKV IgM µ-capture ELISA (Genway-Germany)<br>☐ Anti-CHIKV IgM human ELISA kit (Abcam-Germany)<br>☐ CHIKa IgM ELISA (SD Diagnostics South Korea)<br>☐ Anti-CHIKV ELISA (IgM) (Euroimmun-Germany)<br>☐ CHIKjj Detect MAC-ELISA (Inbios-USA)<br>☐ anti-chikungunya virus ELISA (IgM) kit (Euroimmun US-Mountain Lakes, NJ)<br>☐ Chikungunya IgM µ-capture ELISA (IBL International-Hamburg, Germany)<br>☐ Chikungunya IgG Capture ELISA (IBL International-Hamburg, Germany)<br>☐ Anti-Chikungunya Virus ELISA IgG test (Euroimmun-Lübeck, Germany)<br>☐ Arbovirus Fever Mosaic 2 IgG + IgM IFA ZIKV-DENV-CHIKV (EUROIMMUN)<br>☐ recomLine Tropical Fever IgG (MIKROGEN Diagnostik)<br>☐ recomLine Tropical Fever IgM (MIKROGEN Diagnostik)<br>☐ DPP Zika/Chikungunya/Dengue IgM/IgG Combination Assay (Chembio Diagnostic Systems Inc)<br>☐ STANDARD Q ZIKV/DENV/CHIKV Fast Quad (SD Biosensor<br>☐ STANDARD Q ZIKV/DENV/CHIKV/YFV IgM Quad (SD Biosensor)<br>☐ Tell Me Fast zika/dengue/chikungunya combination rapid tests (Biocan Diagnostics Inc<br>☐ Other
Which other immunoassays were used to diagnose maternal CHIKV infection?	__________________________________
For in house CHIKV immunoassay, describe the testing procedure, including: incubation period, timing, type of cells, buffers, method to detect binding antibodies, and methods to stop the enzymatic reaction	__________________________________
Sample used for CHIKV immunoassay<br><br>Select all that apply	☐ Serum/plasma<br>☐ Whole blood<br>☐ Urine<br>☐ Saliva<br>☐ Breast milk<br>☐ Umbilical cord tissue<br>☐ Placental tissue<br>☐ Amniotic fluid<br>☐ Other
Specify other type of sample used in CHIKV immunoassay	__________________________________

For peer review only

Ascertainment of maternal CHIKV infection - ELISA

CHIKV ELISA cut-off value

____

Was a positive control from the outbreak included for the CHIKV ELISAs?

◯ Yes
◯ No

Was a negative control included with every run of CHIKV ELISAs?

◯ Yes
◯ No

Definition of POSITIVE CHIKV ELISA

____

Definition of NEGATIVE CHIKV ELISA

____

Definition of INCONCLUSIVE CHIKV ELISA

____

Source of definition used to interpret CHIKV ELISA test results

Select all that apply

☐ Manufacturer
☐ Ministry of Health reference lab
☐ WHO (global or local office e.g: PAHO)
☐ Consensus/study researchers
☐ Other

Specify other source of definition for CHIKV ELISA test results

____

Specify any additional inclusion or exclusion criteria for determining which samples to test for CHIKV using ELISA

____

Would your study be able to provide the P/N ratio, titer, or other quantitative readout for CHIKV ELISAs to inform the measurement error models?

◯ Yes
◯ No

For peer review only

Ascertainment of maternal CHIKV infection - IFA

CHIKV IFA cut-off value

____

Was a positive control from the outbreak included for the CHIKV IFA?

◯ Yes
◯ No

Was a negative control included with every run of CHIKV IFA?

◯ Yes
◯ No

Definition of POSITIVE CHIKV IFA

____

Definition of NEGATIVE CHIKV IFA

____

Definition of INCONCLUSIVE CHIKV IFA

____

Source of definition used to interpret CHIKV IFA test results

Select all that apply

☐ Manufacturer
☐ Ministry of Health reference lab
☐ WHO (global or local office e.g: PAHO)
☐ Consensus/study researchers
☐ Other

Specify other source of definition for CHIKV IFA test results

____

Specify any additional inclusion or exclusion criteria for determining which samples to test for CHIKV using IFA

____

Will your study be able to provide the optical density or titer for CHIKV IFAs to inform the measurement error model?

◯ Yes
◯ No

For peer review only

Ascertainment of maternal CHIKV infection - MIA

CHIKV MIA cut-off value	__________________
Was a positive control from the outbreak included for the CHIKV MIAs?	◯ Yes<br>◯ No
Was a negative control included with every run of CHIKV MIAs?	◯ Yes<br>◯ No
Definition of POSITIVE CHIKV MIA	__________________
Definition of NEGATIVE CHIKV MIA	__________________
Definition of INCONCLUSIVE CHIKV MIA	__________________
Source of definition used to interpret CHIKV MIA test results<br><br>Select all that apply	☐ Manufacturer<br>☐ Ministry of Health reference lab<br>☐ WHO (global or local office e.g: PAHO)<br>☐ Consensus/study researchers<br>☐ Other
Specify other source of definition for CHIKV MIA test results	__________________
Specify any additional inclusion or exclusion criteria for determining which samples to test for CHIKV using MIA	__________________
Will your study be able to provide the titer for CHIKV MIAs to inform the measurement error model?	◯ Yes<br>◯ No

For peer review only

Ascertainment of maternal CHIKV infection - Molecular assay

Type of CHIKV PCR/RT-PCR performed<br><br>Select all that apply	☐ PCR (standard or conventional)<br>☐ qRT-PCR<br>☐ rRT-PCR<br>☐ No CHIKV PCR performed
CHIKV PCR/RT-PCR brand name<br><br>Select all that apply	☐ In house procedure<br>☐ CHIKV CDC qRT-PCR (CDC-USA)<br>☐ CDC Trioplex rRT-PCR Assay (CDC-USA)<br>☐ RealStar Chikungunya RT-PCR Kit (Altona Diagnostics-Germany)<br>☐ Genesig Chikungunya Non structural protein 2 standard (nsp2) RT-PCR kit (Primerdesign-UK)<br>☐ CII-ArboViroPlex rRT-PCR assay (Columbia University)<br>☐ AccuPower ZIKV (DENV, CHIKV) Multiplex Real-Time RT-PCR kit (Bioneer)<br>☐ VIASURE Zika, Dengue & Chikungunya Real Time PCR Detection Kit (Certest Biotec)<br>☐ FTD Zika/Dengue/Chik (Fast-Track Diagnostics)<br>☐ DiaPlexQ ZCD (ZIKV, CHIKV, DENV) Detection Kit (SolGent Co Ltd)<br>☐ TaqMan Zika Virus Triplex Kit (Thermo Fisher Scientific)<br>☐ Dengue Fever Virus & Chikungunya Virus Real Time RT-PCR Kit (Creative Biogene)<br>☐ GenoAmp Trioplex Real-Time RT-PCR Zika/Den/Chiku (Medical Innovation Ventures)<br>☐ Genesig Multiplex Kit for Dengue, Chikungunya and Zika Virus (Primerdesign Ltd)<br>☐ Other
Describe procedure for in house PCR/RT-PCR	__________________________________
Specify other CHIKV PCR/RT-PCR brand used	__________________________________
Sample used for CHIKV PCR/RT-PCR<br><br>Select all that apply	☐ Serum/plasma<br>☐ Whole blood<br>☐ Urine<br>☐ Saliva<br>☐ Breast milk<br>☐ Umbilical cord tissue<br>☐ Placental tissue<br>☐ Amniotic fluid<br>☐ Other
Specify other type of sample used for CHIKV PCR/RT-PCR	__________________________________
Did the study obtain a measure of CHIKV viral load?	◯ Yes<br>◯ No
CHIKV PCR/RT-PCR cut-off point	__________________________________
Was a negative control included with every run of CHIKV PCR/RT-PCRs?	◯ Yes<br>◯ No

For peer review only

Definition of POSITIVE CHIKV PCR/RT-PCR	__________
Definition of NEGATIVE CHIKV PCR/RT-PCR	__________
Definition of INCONCLUSIVE CHIKV PCR/RT-PCR	__________
Source of the definition used to interpret CHIKV PCR/RT-PCR test results<br><br>Select all that apply	☐ Manufacturer<br>☐ Ministry of Health reference lab<br>☐ WHO (global or local office e.g: PAHO)<br>☐ US-CDC<br>☐ Consensus/study researchers<br>☐ Other
Specify other source of the definition used to interpret CHIKV PCR/RT-PCR test results	__________
Specify any additional inclusion or exclusion criteria for determining which samples to test for CHIKV using PCR/RT-PCR	__________
Where was the CHIKV PCR/RT-PCR test performed?<br><br>Select all that apply	☐ Lab at the same health care facility where study is conducted<br>☐ Study-specific lab<br>☐ Ministry of Health reference lab<br>☐ Private external lab<br>☐ Other
Specify other lab where the CHIKV PCR/RT-PCR test was performed	__________

For peer review only

CHIKV testing algorithm & qualitative definitions

What tests were used to confirm CHIKV infection in pregnant women?<br><br>Select all that apply	☐ CHIKV IgM - 1 sample<br>☐ CHIKV IgM - paired samples (seroconversion or increased titers)<br>☐ CHIKV IgM (1 sample) + IgG<br>☐ CHIKV IgM (paired samples) + IgG<br>☐ RT-PCR or other NAAT<br>☐ PRNT > 10<br>☐ Other
Specify other test or combination of tests used to confirm CHIKV infection	__________________________________
Considering any relevant clinical and laboratory criteria, what was the study's definition of a CONFIRMED maternal CHIKV infection	__________________________________
Considering any relevant clinical and laboratory criteria, what was the study's definition of a PROBABLE maternal CHIKV infection	__________________________________
Considering any relevant clinical and laboratory criteria, what was the study's definition of a NEGATIVE maternal CHIKV infection	__________________________________

For peer review only

Ascertainment of maternal infection with other flavi/alphaviruses

Does the study capture information on maternal history of any other flavivirus/alphavirus infections?	◯ Yes, self-reported<br>◯ Yes, lab assessment<br>◯ Yes, both self-reported and lab assessment<br>◯ No
Does the study capture information on maternal history of yellow fever or Japanese encephalitis vaccination?	◯ Yes<br>◯ No
Was testing performed for other current maternal arbovirus infections? If so, which ones?<br><br>Select all that apply	☐ DENV<br>☐ CHIKV<br>☐ Japanese/Venezuelan encephalitis<br>☐ Mayaro virus<br>☐ West Nile virus<br>☐ Other arboviruses<br>☐ No, study did not test for concurrent arbovirus infections
Which other current maternal arboviral infections did the study test for?	______________________________

For peer review only

Ascertainment of placental or fetal ZIKV infection

Was placental ZIKV infection measured?	◯ Yes<br>◯ No
Was fetal ZIKV infection measured?	◯ Yes<br>◯ No
Which criteria did you use to infer fetal ZIKV status?<br><br>Select all that apply	☐ fetal clinical criteria<br>☐ fetal laboratory criteria<br>☐ fetal imaging criteria<br>☐ maternal clinical criteria<br>☐ maternal laboratory criteria<br>☐ placental clinical criteria<br>☐ placental laboratory criteria<br>☐ other criteria
In what other way was fetal ZIKV infection assessed?	__________________________________
Which laboratory tests were used to assess fetal ZIKV status?<br><br>Select all that apply	☐ molecular diagnosis (e.g. RT-PCR, other NAAT)<br>☐ immunoassay (e.g. ELISA, IFA, PRNT, IHC, microneutralization)<br>☐ rapid diagnostic test (RDT)<br>☐ Other
Which other laboratory tests were used to assess fetal ZIKV status?	__________________________________
Were the assays or the clinical criteria used to assess fetal ZIKV infection the same as the assays or clinical criteria used to assess placental ZIKV infection?	◯ Yes<br>◯ No
Please describe the differences between the clinical criteria or assays used to assess fetal and placental ZIKV infection	____________________________
When was fetal ZIKV infection measured using laboratory criteria?<br><br>Select all that apply	☐ 1st trimester<br>☐ 2nd trimester<br>☐ 3rd trimester<br>☐ First antenatal visit<br>☐ Following confirmation of maternal ZIKV infection, irregardless of trimester<br>☐ Other
Type of sample(s) obtained to ascertain fetal ZIKV infection<br><br>Select all that apply	☐ Amniotic fluid<br>☐ Placental tissue (Including umbilical cord and fetal membranes)<br>☐ Fetal tissue<br>☐ Other
What other type(s) of samples were obtained to ascertain fetal ZIKV infection?	____________________________

For peer review only

At what other time was fetal ZIKV infection measured?	__________________________________
Where was the fetal sample analyzed?<br><br>Select all that apply	☐ Lab at the same health care facility where study is conducted<br>☐ Study-specific lab<br>☐ Ministry of Health reference lab<br>☐ Private external lab<br>☐ Other
Specify other lab where the fetal sample was analyzed	___________________________
Did you use different assays, different brands of the same assay, or different versions of the same brand of assay at different study sites to ascertain fetal ZIKV status or over time within the study?<br><br>Select all that apply	☐ Yes, different versions of the same brand of assay over time<br>☐ Yes, different brands of the same type of assay over time<br>☐ Yes, different types of assays over time<br>☐ Yes, different versions of the same brand of assay at different sites<br>☐ Yes, different brands of the same type of assay at different sites<br>☐ Yes, different types of assays at different sites<br>☐ No, same tests applied throughout the project
Which different assay(s) did you use over time or across sites?	__________________________________
Which different brands of the assay did you use over time or across sites?	__________________________________
Which different versions of the brand of assay did you use over time or across sites?	__________________________________

For peer review only

Ascertainment of fetal ZIKV infection - Immunoassay

ZIKV immunoassay analyte to ascertain fetal infection<br><br>Select all that apply	☐ IgM<br>☐ IgG<br>☐ IgA<br>☐ neutralizing antibodies<br>☐ Other
Other ZIKV immunoassay analyte to ascertain fetal infection	__________________________________
ZIKV immunoassay type to ascertain fetal infection<br><br>Select all that apply	☐ ELISA<br>☐ IFA<br>☐ MIA<br>☐ PRNT<br>☐ Other
Specify other type(s) or ZIKV immunoassay(s) performed to ascertain fetal infection	__________________________________

For peer review only

ZIKV immunoassay brand name<br><br>Select all that apply	☐ In house-developed assay<br>☐ Zika MAC-ELISA (CDC)<br>☐ ZIKV Detect 2.0 IgM Capture ELISA Kit (InBios International Inc)<br>☐ Zika IgM ELISA (EUROIMMUN)<br>☐ Zika IgM + IgA ELISA (EUROIMMUN)<br>☐ Zika IgG ELISA (EUROIMMUN)<br>☐ ZIKV IgM IFA (EUROIMMUN)<br>☐ ZIKV IgG IFA (EUROIMMUN)<br>☐ Arbovirus Fever Mosaic 2 IgG + IgM IFA ZIKV-DENV-CHIKV (EUROIMMUN)<br>☐ ADVIA Centaur Zika Test (Siemens Healthcare Diagnostics)<br>☐ DPP Zika IgM Assay System (Chembio Diagnostic Systems Inc)<br>☐ DPP Zika IgM/IgG System (Chembio Diagnostic Systems Inc)<br>☐ DPP Zika/Chikungunya/Dengue IgM/IgG Combination Assay (Chembio Diagnostic Systems Inc)<br>☐ LIAISON XL Zika Capture IgM II assay (DiaSorin S.p.A.)<br>☐ STANDARD Q Zika IgM/IgG test (SD Biosensor)<br>☐ STANDARD E Zika IgM ELISA (SD Biosensor)<br>☐ STANDARD Q ZIKV/DENV/CHIKV Fast Quad (SD Biosenso<br>☐ STANDARD Q ZIKV/DENV/CHIKV/YFV IgM Quad (SD Biosensor)<br>☐ Tell Me Fast Zika IgG/IgM Antibody Rapid Test (Biocan Diagnostics Inc)<br>☐ Tell Me Fast zika/dengue/chikungunya combination rapid tests (Biocan Diagnostics Inc)<br>☐ ZIKV-DENV-CHIKV IFA IgG (Vircell)<br>☐ ZIKV-DENV-CHIKV IFA IgM (Vircell)<br>☐ Human Anti-Zika ELISA Kits (Alpha Diagnostic International)<br>☐ anti-Zika virus IgM ELISA kit (µ-capture) (AbCam)<br>☐ anti-Zika virus IgG ELISA kit (AbCam)<br>☐ Zika Virus IgM ELISA Assay Kit (Eagle Biosciences)<br>☐ Zika Virus IgG ELISA Assay Kit (Eagle Biosciences)<br>☐ Human Anti-Zika Virus IgG ELISA Kit (R&D Systems)<br>☐ ZIKV IgG ELISA kit (DIA.PRO Diagnostic Bioprobes Srl)<br>☐ ZIKV IgM ELISA kit (DIA.PRO Diagnostic Bioprobes Srl)<br>☐ NovaLisa Zika Virus IgM µ-capture ELISA (NovaTec Immunodiagnostica GmbH)<br>☐ recomLine Tropical Fever IgG (MIKROGEN Diagnostik)<br>☐ recomLine Tropical Fever IgM (MIKROGEN Diagnostik)<br>☐ Other
Which other immunoassays were used to diagnose fetal ZIKV infection?	__________________________________
What type of assay is the in house assay (to ascertain fetal infection)?<br><br>Select all that apply	☐ ELISA<br>☐ IFA<br>☐ MIA<br>☐ PRNT<br>☐ Other
What type of assay is the in house assay (to ascertain fetal infection)?	__________________________________

For peer review only

What is the analyte measured by the in house assay (to ascertain fetal infection)?<br><br>Select all that apply	☐ IgM<br>☐ IgG<br>☐ IgA
What is the antigenic target for the in house assay (to ascertain fetal infection)?<br><br>Select all that apply	☐ NS1<br>☐ envelope protein<br>☐ NS5<br>☐ Other
What is the antigenic target for the in house assay (to ascertain fetal infection)?	__________________________________
For the in house ZIKV immunoassay to ascertain fetal infection, describe the testing procedure, including: incubation period, timing, type of cells, buffers, method to detect binding antibodies (e.g., optical density or secondary antibody detection), and methods to stop the enzymatic reaction	__________________________________
Sample used for ZIKV immunoassay (to ascertain fetal infection)<br><br>Select all that apply	☐ Serum/plasma<br>☐ Whole blood<br>☐ cerebrospinal fuid (CSF)<br>☐ chord blood<br>☐ Other
Specify other sample used for ZIKV immunoassay (to ascertain fetal infection)	____________________________
ZIKV ELISA tested in paired/subsequent samples for ascertaining fetal infection	◯ Yes<br>◯ No
At what temperature was the ZIKV ELISA test stored for fetal infection? (degrees Celsius)	____________________________
At what temperature(s) was/were the ZIKV ELISA sample(s) stored for fetal infection? (degrees Celsius)	__________________________________<br>(Include sample type for each temperature)
In what volume(s) was/were the ZIKV ELISA sample aliquot(s) stored for fetal infection? (mL)	__________________________________<br>(Include sample type with each volume)
Did your study deviate from the directions provided in the ZIKV ELISA commercial kit for fetal infection?	◯ Yes<br>◯ No<br>◯ Study did not use a commercial ZIKV ELISA kit
Describe any deviations from the procedure recommended by the commercial ELISA kit, including: incubation period, timing, type of cells, buffers, method to detect bounding antibodies, and methods to stop the enzymatic reaction	__________________________________

For peer review only

Describe any additional conditions for ZIKV ELISA test administration for ascertaining fetal infection (e.g. paired samples within 21 days of fever onset; count with more than 2ul of serum from the febrile phase, etc.)	__________________________________
Where was the ZIKV ELISA test performed?<br><br>Select all that apply	☐ Lab at the same health care facility where study is conducted<br>☐ Study-specific lab<br>☐ Ministry of Health reference lab<br>☐ Private external lab<br>☐ Other
Specify other lab where ZIKV ELISA was performed for fetal infection	__________________________

For peer review only

Ascertainment of fetal ZIKV infection - ELISA

ZIKV ELISA cut-off point for fetal infection	__________________________
Was a positive control from the outbreak included for the ZIKV ELISAs for fetal infection?	◯ Yes<br>◯ No
Was a negative control included with every run of ZIKV ELISAs for fetal infection?	◯ Yes<br>◯ No
Definition of POSITIVE ZIKV ELISA for fetal infection	__________________________
Definition of NEGATIVE ZIKV ELISA for fetal infection	__________________________
Definition of INCONCLUSIVE ZIKV ELISA for fetal infection	__________________________
Source of definition used to interpret ZIKV immunoassay test results<br><br>Select all that apply	☐ Manufacturer<br>☐ Ministry of Health reference lab<br>☐ WHO (global or local office e.g: PAHO)<br>☐ US-CDC<br>☐ Consensus/study researchers<br>☐ Other
Specify other source of definition used to interpret ZIKV immunoassay test results	__________________________
How many ZIKV ELISAs did the study plan to conduct in each fetus?	__________________________
Would your study be able to provide the P/N ratio, titer, or other quantitative readout for ZIKV ELISAs to inform the measurement error models?	◯ Yes<br>◯ No

For peer review only

Ascertainment of fetal ZIKV infection - IFA

ZIKV IFA cut-off value for fetal infection ______________________

Was a positive control from the outbreak included for the ZIKV IFA for fetal infection?
◯ Yes
◯ No

Was a negative control included with every run of ZIKV IFA for fetal infection?
◯ Yes
◯ No

Definition of POSITIVE ZIKV IFA for fetal infection ______________________

Definition of NEGATIVE ZIKV IFA for fetal infection ______________________

Definition of INCONCLUSIVE ZIKV IFA for fetal infection ______________________

Source of definition for ZIKV IFA test results for fetal infection

Select all that apply
☐ Manufacturer
☐ Ministry of Health reference lab
☐ WHO (global or local office e.g: PAHO)
☐ Consensus/study researchers
☐ Other

Specify other source of definition for ZIKV IFA test results ______________________

Specify any additional inclusion or exclusion criteria for determining which samples to test for ZIKV using IFA ______________________

Will your study be able to provide the titer or optical density for ZIKV IFAs to inform the measurement error model?
◯ Yes
◯ No

For peer review only

Ascertainment of fetal ZIKV infection - MIA

ZIKV MIA cut-off value for fetal infection

____

Was a positive control from the outbreak included for the ZIKV MIAs for fetal infection?
- ◯ Yes
- ◯ No

Was a negative control included with every run of ZIKV MIAs for fetal infection?
- ◯ Yes
- ◯ No

Definition of POSITIVE ZIKV MIA for fetal infection

____

Definition of NEGATIVE ZIKV MIA for fetal infection

____

Definition of INCONCLUSIVE ZIKV MIA for fetal infection

____

Source of definition for MIA ZIKV test results for fetal infection

Select all that apply
- ☐ Manufacturer
- ☐ Ministry of Health reference lab
- ☐ WHO (global or local office e.g: PAHO)
- ☐ Consensus/study researchers
- ☐ Other

Specify other source of definition for ZIKV MIA test results

____

Specify any additional inclusion or exclusion criteria for determining which samples to test for ZIKV using MIA for fetal infection

____

Will your study be able to provide the titer for ZIKV MIAs to inform the measurement error model?
- ◯ Yes
- ◯ No

For peer review only

Ascertainment of fetal ZIKV infection - Molecular assay

Type of ZIKV molecular tests performed on fetus<br><br>Select all that apply	☐ conventional PCR<br>☐ rRT-PCR<br>☐ qRT-PCR<br>☐ Other NAAT
According to the study protocol, when were the ZIKV samples to be collected for molecular testing?<br><br>Select all that apply	☐ within 24 hours after birth<br>☐ within first week after birth<br>☐ Other
When were the ZIKV molecular tests (RT-PCR, other NAAT) conducted on fetus?	______________________________
Sample used for ZIKV RT-RCR/PCR in fetus<br><br>Select all that apply	☐ Serum/plasma<br>☐ Whole blood<br>☐ Urine<br>☐ Saliva<br>☐ Breast milk<br>☐ Umbilical cord tissue<br>☐ cerebrospinal fuid (CSF)<br>☐ chord blood<br>☐ Other
Specify other type of sample used for ZIKV RT-PCR/PCR on fetus	______________________________

For peer review only

ZIKV RT-PCR/PCR brand name used for testing fetus<br><br>Select all that apply	☐ In house technique<br>☐ Zika Virus RNA Qualitative Real-Time RT-PCR (Quest Diagnostics)<br>☐ RealStar Zika Virus RT-PCR Kit U.S. (altona Diagnostics GmbH)<br>☐ RealStar Zika Virus RT-PCR Kit 1.0 (altona Diagnostics GmbH)<br>☐ Aptima Zika Virus assay (Hologic Inc)<br>☐ Zika Virus Real-time RT-PCR (Viracor Eurofins)<br>☐ VERSANT Zika RNA 1.0 Assay (kPCR) (Siemens Healthcare Diagnostics Inc)<br>☐ xMAP MultiFLEX Zika RNA Assay (Luminex Corporation)<br>☐ Sentosa SA ZIKV RT-PCR Test (Vela Diagnostics Inc)<br>☐ Zika Virus Detection by RT-PCR (ARUP Laboratories)<br>☐ Abbott RealTime Zika (Abbott Molecular Inc)<br>☐ Zika ELITe MGB Kit U.S. (ELITechGroup Molecular Diagnostics)<br>☐ Gene-RADAR Zika Virus Test (Nanobiosym Diagnostics Inc)<br>☐ TaqPath Zika Virus Kit (Thermo Fisher Scientific)<br>☐ CII-ArboViroPlex rRT-PCR assay (Columbia University)<br>☐ Trioplex Real-Time RT-PCR Assay (CDC)<br>☐ Liferiver Zika Virus (ZIKV) Real Time RT-PCR Kit (Shanghai ZJ Bio-Tech Co Ltd)<br>☐ careGENE Zika Virus RT-PCR Kit (WELLS BIO Inc)<br>☐ Zika Virus - Single Check (Genekam Biotechnology AG)<br>☐ FTD Zika virus (Fast Track Diagnostics)<br>☐ VIASURE Zika Virus Real Time PCR Detection Kit (Certest Biotec)<br>☐ Zika Virus Real-TM (Sacace Biotechnologies)<br>☐ Logix Smart Zika Test Kit (Co-Diagnostics Inc)<br>☐ Genesig Kits for ZIKV (Primerdesign Ltd)<br>☐ Genesig Multiplex Kit for Dengue, Chikungunya and Zika Virus (Primerdesign Ltd)<br>☐ AccuPower ZIKV (DENV, CHIKV) Multiplex Real-Time RT-PCR Kit (Bioneer)<br>☐ VIASURE Zika, Dengue & Chikungunya Real Time PCR Detection Kit (Certest Biotec)<br>☐ FTD Zika/Dengue/Chik (Fast-Track Diagnostics)<br>☐ DiaPlexQ ZCD (ZIKV, CHIKV, DENV) Detection Kit (SolGent Co Ltd)<br>☐ TaqMan Zika Virus Triplex Kit (Thermo Fisher Scientific)<br>☐ GenoAmp Trioplex Real-Time RT-PCR Zika/Den/Chiku (Medical Innovation Ventures)<br>☐ Other
Describe procedure for in house ZIKV RT-PCR/PCR for fetal infection	__________________________________
Specify other ZIKV RT-PCR/PCR brand name	__________________________________
Did the study obtain a measure of ZIKV viral load for fetal infection?	◯ Yes<br>◯ No
ZIKV PCR/RT-PCR cut-off point for fetal infection	____________________________

For peer review only

Was a negative control included with every run of ZIKV RT-PCR/PCRs for fetal infection?	◯ Yes<br>◯ No
Definition of POSITIVE ZIKV RT-PCR/PCR for fetal infection	__________________________________
Definition of NEGATIVE ZIKV RT-PCR/PCR for fetal infection	__________________________________
Definition of INCONCLUSIVE ZIKV RT-PCR/PCR for fetal infection	__________________________________
Source of definition used to interpret ZIKV RT-PCR/PCR test results for fetal infection<br><br>Select all that apply	☐ Manufacturer<br>☐ Ministry of Health reference lab<br>☐ WHO (global or local office e.g: PAHO)<br>☐ US-CDC<br>☐ Consensus/study researchers<br>☐ Other
Specify other source of definition used to interpret ZIKV RT-PCR/PCR test results	___________________________
Where was the ZIKV RT-PCR/PCR test performed for samples from fetus?<br><br>Select all that apply	☐ Lab at the same health care facility where study is conducted<br>☐ Study-specific lab<br>☐ Ministry of Health reference lab<br>☐ Private external lab<br>☐ Other
Specify other lab where ZIKV RT-PCR/PCR test was performed for fetal samples	___________________________
Specify any additional inclusion or exclusion criteria for determining which samples from fetus to test for ZIKV using RT-PCR/PCR	__________________________________
How many ZIKV PCRs were conducted with with each fetus?	___________________________
Would your study be able to provide a Ct value for fetal ZIKV PCRs to inform the measurement error models?	◯ Yes<br>◯ No

For peer review only

Changes in diagnosis of fetal ZIKV infection over time

Which clinical criteria did you apply initially to diagnose fetal infection? (Clinical Definition 1) __________________________________

Did your clinical criteria for fetal infection change over time?
- ◯ No
- ◯ Yes - changed 1x
- ◯ Yes - changed more than 1x

If your clinical criteria changed over time, which criteria did you apply subsequently to assess fetal infection? (Clinical Definition 2) __________________________________

When did you begin using the revised clinical criteria for assessing fetal infection? (Clinical Definition 2) ___________________________

What was the third set of criteria you applied to assess fetal infection? (Clinical Definition 3) __________________________________

When did you begin using the third set of clinical criteria? (Clinical Definition 3) ___________________________

Which imaging criteria did you apply initially to diagnose fetal infection? (Clinical Definition 1) __________________________________

Did your imaging criteria for fetal infection change over time?
- ◯ No
- ◯ Yes - changed 1x
- ◯ Yes - changed more than 1x

If your imaging criteria changed over time, which criteria did you apply subsequently to assess fetal infection? (Clinical Definition 2) __________________________________

When did you begin using the revised imaging criteria for assessing fetal infection? (Clinical Definition 2) ___________________________

What was the third set of imaging criteria you applied to assess fetal infection? (Clinical Definition 3) __________________________________

When did you begin using the third set of imaging criteria? (Clinical Definition 3) ___________________________

What laboratory criteria was used initially to diagnose fetal ZIKV infection? (Laboratory Definition 1) __________________________________

Did your laboratory criteria for diagnosing fetal infection change over time?
- ◯ No
- ◯ Yes - changed 1x
- ◯ Yes - changed more than 1x

For peer review only

Which laboratory criteria did you apply subsequently? (Laboratory Definition 2) ______________________

When did you begin using the revised lab criteria for fetal infection? (Laboratory Definition 2) ______________________

What was the third set of laboratory criteria you applied for diagnosing fetal infection? (Laboratory Definition 3) ______________________

When did you begin using the third set of lab criteria for fetal infection? (Laboratory Definition 3) ______________________

Were fetal samples retested using the new laboratory criteria? ______________________

For peer review only

ZIKV testing algorithm & test interpretation - fetal ZIKV infection

Describe the testing algorithm for defining a confirmed ZIKV+ test in fetal testing	__________________________________
Describe the testing algorithm for defining a confirmed ZIKV- test in fetal testing	__________________________________
What tests were used to confirm ZIKV infection in fetal testing?<br><br>Select all that apply	☐ ZIKV IgM - 1 sample<br>☐ ZIKV IgM - paired samples (seroconversion or increased titers)<br>☐ ZIKV IgM (1 sample) + IgG<br>☐ ZIKV IgM (paired samples) + IgG<br>☐ RT-PCR or other NAAT<br>☐ PRNT > 10<br>☐ Other
Specify other test or combination of tests used to confirm ZIKV infection in fetal testing	__________________________________
Considering any relevant clinical and laboratory criteria, what was the study's definition of a CONFIRMED fetal ZIKV Infection	__________________________________
Considering any relevant clinical and laboratory criteria, what was the study's definition of a PROBABLE fetal ZIKV Infection	__________________________________
Considering any relevant clinical and laboratory criteria, what was the study's definition of a NEGATIVE/UNLIKELY fetal ZIKV Infection	__________________________________
Source of ZIKV test result categorization for fetal testing<br><br>Select all that apply	☐ Manufacturer<br>☐ Ministry of Health reference lab<br>☐ WHO (global or local office e.g: PAHO)<br>☐ Consensus/study researchers<br>☐ Other
Specify other source of ZIKV test result categorization for fetal testing	______________________________

For peer review only

Fetal Imaging & Ascertainment of Prenatal Microcephaly

Was fetal ultrasound performed?
◯ Yes
◯ No

How many fetal ultrasounds did the study plan to conduct for each pregnant woman?
____

When in pregnancy did the study plan to conduct the ultrasound(s)?
____
(List all trimesters or weeks of gestation the study had planned to administer ultrasound)

Fetal ultrasound machine model(s)
____

Where were fetal ultrasounds performed?

Select all that apply
☐ Same health care facility where study is taking place
☐ Study-specific site (not a health care facility)
☐ Private/external site

Training received by ultrasound technician
____

Quality control for ultrasound evaluation of abnormalities/head circumference
____

Criteria for considering a fetal ultrasound as "abnormal"
____

Was fetal MRI performed?
◯ Yes
◯ No

How many fetal MRIs did the study plan to conduct for each pregnant woman?
____

When in pregnancy did the study plan to conduct the fetal MRI(s)?
____
(List all trimesters or weeks of gestation the study had planned to administer MRIs)

Fetal MRI machine model(s)
____

Where were fetal MRIs performed?

Select all that apply
☐ Same health care facility where study is taking place
☐ Study-specific site (not a health care facility)
☐ Private/external site

For peer review only

Training received by MRI technician	__________________
Quality control for MRI evaluation of abnormalities/head circumference	__________________
Criteria for considering a fetal MRI as "abnormal"	__________________
When was prenatal/fetal microcephaly diagnosed?	◯ Postnatally diagnosed (after birth) congenital microcephaly<br>◯ Prenatally diagnosed (in utero) congenital microcephaly<br>◯ Study did not measure prenatal/fetal microcephaly
Gestational age (in weeks) at which study first assessed prenatal microcephaly	__________________
Reference standards used to calculate fetal head circumference Z score<br><br>Select all that apply	☐ Intergrowth<br>☐ Ministry of Health standards<br>☐ WHO or PAHO standard<br>☐ Local reference standard<br>☐ Consensus/study researchers<br>☐ Other
Specify other reference standards used to calculate fetal head circumference Z score	__________________
How was prenatal microcephaly diagnosed?<br><br>Select all that apply	☐ Presence vs absence<br>☐ By grades (normocephaly vs microcephaly vs severe microcephaly)<br>☐ By percentiles<br>☐ By Z-score<br>☐ By diagnostic certainty criteria<br>☐ Other
Specify other type of diagnosis of prenatal microcephaly	__________________
How was prenatal microcephaly defined?	◯ >2 SD below the mean<br>◯ < 3 SD below the mean<br>◯ 3 SD below the mean<br>◯ >3 SD below the mean<br>◯ below the 5th percentile<br>◯ below the 10th percentile<br>◯ Other
Specify other definition of prenatal microcephaly	__________________

For peer review only

How was prenatal microcephaly defined?	◯ Level 1A of diagnostic certainty<br>◯ Level 1B of diagnostic certainty<br>◯ Level 2 of diagnostic certainty<br>◯ Level 3A of diagnostic certainty<br>◯ Level 3B of diagnostic certainty<br>◯ Level 4 of diagnostic certainty<br>◯ Other
How else was prenatal microcephaly defined?	__________________________________

For peer review only

Ascertainment of infant ZIKV infection - Immunoassay

Was infant ZIKV infection measured?

- ◯ Yes
- ◯ No

Which criteria did you use to infer infant ZIKV status?

Select all that apply

- ☐ infant clinical criteria
- ☐ infant laboratory criteria
- ☐ infant imaging criteria
- ☐ maternal clinical criteria
- ☐ maternal laboratory criteria
- ☐ other criteria

In what other way was infant ZIKV infection assessed?

____

When was infant ZIKV infection measured?

Select all that apply

- ☐ Immediately after birth
- ☐ 1 month after birth
- ☐ 6 months after birth
- ☐ Other

At what other time was infant ZIKV infection measured?

____

Type of laboratory test performed to ascertain infant ZIKV infection

Select all that apply

- ☐ molecular diagnosis (e.g. RT-PCR, other NAAT)
- ☐ immunoassay (e.g. ELISA, IFA, PRNT, IHC, microneutralization)
- ☐ rapid diagnostic test (RDT)
- ☐ Other

Specify other test performed to ascertain infant ZIKV infection

____

Did you use different assays, different brands of the same assay, or different versions of the same brand of assay at different study sites to ascertain infant ZIKV status or over time within the study?

Select all that apply

- ☐ Yes, different versions of the same brand of assay over time
- ☐ Yes, different brands of the same type of assay over time
- ☐ Yes, different types of assays over time
- ☐ Yes, different versions of the same brand of assay at different sites
- ☐ Yes, different brands of the same type of assay at different sites
- ☐ Yes, different types of assays at different sites
- ☐ No, same tests applied throughout the project

Which different assay(s) did you use over time or across sites?

____

Which different brands of the assay did you use over time or across sites?

____

Which different versions of the brand of assay did you use over time or across sites?

____

For peer review only

ZIKV immunoassay analyte to ascertain infant infection<br><br>Select all that apply	☐ IgM<br>☐ IgG<br>☐ IgA<br>☐ neutralizing antibodies<br>☐ Other
Other ZIKV immunoassay analyte to ascertain infant infection	__________________
ZIKV immunoassay type to ascertain infant infection<br><br>Select all that apply	☐ ELISA<br>☐ IFA<br>☐ MIA<br>☐ PRNT<br>☐ Other
Specify other type(s) or ZIKV immunoassay(s) performed to ascertain infant infection	__________________

For peer review only

ZIKV immunoassay brand name (to ascertain infant infection)

Select all that apply

- ☐ In house-developed assay
- ☐ Zika MAC-ELISA (CDC)
- ☐ ZIKV Detect 2.0 IgM Capture ELISA Kit (InBios International Inc)
- ☐ Zika IgM ELISA (EUROIMMUN)
- ☐ Zika IgM + IgA ELISA (EUROIMMUN)
- ☐ Zika IgG ELISA (EUROIMMUN)
- ☐ ZIKV IgM IFA (EUROIMMUN)
- ☐ ZIKV IgG IFA (EUROIMMUN)
- ☐ Arbovirus Fever Mosaic 2 IgG + IgM IFA ZIKV-DENV-CHIKV (EUROIMMUN)
- ☐ ADVIA Centaur Zika Test (Siemens Healthcare Diagnostics)
- ☐ DPP Zika IgM Assay System (Chembio Diagnostic Systems Inc)
- ☐ DPP Zika IgM/IgG System (Chembio Diagnostic Systems Inc)
- ☐ DPP Zika/Chikungunya/Dengue IgM/IgG Combination Assay (Chembio Diagnostic Systems Inc)
- ☐ LIAISON XL Zika Capture IgM II assay (DiaSorin S.p.A.)
- ☐ STANDARD Q Zika IgM/IgG test (SD Biosensor)
- ☐ STANDARD E Zika IgM ELISA (SD Biosensor)
- ☐ STANDARD Q ZIKV/DENV/CHIKV Fast Quad (SD Biosenso[illegible]
- ☐ STANDARD Q ZIKV/DENV/CHIKV/YFV IgM Quad (SD Biosensor)
- ☐ Tell Me Fast Zika IgG/IgM Antibody Rapid Test (Biocan Diagnostics Inc)
- ☐ Tell Me Fast zika/dengue/chikungunya combination rapid tests (Biocan Diagnostics Inc)
- ☐ ZIKV-DENV-CHIKV IFA IgG (Vircell)
- ☐ ZIKV-DENV-CHIKV IFA IgM (Vircell)
- ☐ Human Anti-Zika ELISA Kits (Alpha Diagnostic International)
- ☐ anti-Zika virus IgM ELISA kit (µ-capture) (AbCam)
- ☐ anti-Zika virus IgG ELISA kit (AbCam)
- ☐ Zika Virus IgM ELISA Assay Kit (Eagle Biosciences)
- ☐ Zika Virus IgG ELISA Assay Kit (Eagle Biosciences)
- ☐ Human Anti-Zika Virus IgG ELISA Kit (R&D Systems)
- ☐ ZIKV IgG ELISA kit (DIA.PRO Diagnostic Bioprobes Srl)
- ☐ ZIKV IgM ELISA kit (DIA.PRO Diagnostic Bioprobes Srl)
- ☐ NovaLisa Zika Virus IgM µ-capture ELISA (NovaTec Immunodiagnostica GmbH)
- ☐ recomLine Tropical Fever IgG (MIKROGEN Diagnostik)
- ☐ recomLine Tropical Fever IgM (MIKROGEN Diagnostik)
- ☐ Other

Which other immunoassays were used to diagnose infant ZIKV infection?

____

What type of assay is the in house assay? (to ascertain infant ZIKV infection)

Select all that apply

- ☐ ELISA
- ☐ IFA
- ☐ MIA
- ☐ PRNT
- ☐ Other

What type of assay is the in house assay? (to ascertain infant ZIKV infection)

____

For peer review only

What is the analyte measured by the in house assay? (to ascertain infant ZIKV infection)<br><br>Select all that apply	☐ IgM<br>☐ IgG<br>☐ IgA
What is the antigenic target for the in house assay (to ascertain infant ZIKV infection)<br><br>Select all that apply	☐ NS1<br>☐ envelope protein<br>☐ NS5<br>☐ Other
What is the antigenic target for the in house assay (to ascertain infant ZIKV infection)	__________________________________
For the in house ZIKV immunoassay to ascertain infant infection, describe the testing procedure, including: incubation period, timing, type of cells, buffers, method to detect binding antibodies (e.g., optical density or secondary antibody detection), and methods to stop the enzymatic reaction	__________________________________
Sample used for ZIKV immunoassay (to ascertain infant infection)<br><br>Select all that apply	☐ Serum/plasma<br>☐ Whole blood<br>☐ cerebrospinal fuid (CSF)<br>☐ chord blood<br>☐ Other
Specify other sample used for ZIKV immunoassay (to ascertain infant infection)	____________________________
ZIKV ELISA tested in paired/subsequent samples	◯ Yes<br>◯ No
At what temperature was the ZIKV ELISA test stored? (degrees Celsius)	____________________________
At what temperature(s) was/were the ZIKV ELISA sample(s) stored? (degrees Celsius)	__________________________________<br>(Include sample type for each temperature)
In what volume(s) was/were the ZIKV ELISA sample aliquot(s) stored? (mL)	__________________________________<br>(Include sample type with each volume)
Did your study deviate from the directions provided in the ZIKV ELISA commercial kit?	◯ Yes<br>◯ No<br>◯ Study did not use a commercial ZIKV ELISA kit
Describe any deviations from the procedure recommended by the commercial ELISA kit, including: incubation period, timing, type of cells, buffers, method to detect bounding antibodies, and methods to stop the enzymatic reaction	__________________________________

For peer review only

Describe any additional conditions for ZIKV ELISA test administration for ascertining infant infection (e.g. paired samples within 21 days of fever onset; count with more than 2ul of serum from the febrile phase, etc.)	__________________________________
Where was the ZIKV ELISA test performed for ascertining infant infection?<br><br>Select all that apply	☐ Lab at the same health care facility where study is conducted<br>☐ Study-specific lab<br>☐ Ministry of Health reference lab<br>☐ Private external lab<br>☐ Other
Specify other lab where ZIKV ELISA was performed	__________________________

For peer review only

Ascertainment of infant ZIKV infection - ELISA

ZIKV ELISA cut-off point for ascertining infant infection ______________________

Was a positive control from the outbreak included for the ZIKV ELISAs for ascertining infant infection?
◯ Yes
◯ No

Was a negative control included with every run of ZIKV ELISAs for ascertining infant infection?
◯ Yes
◯ No

Definition of POSITIVE ZIKV ELISA for ascertining infant infection ______________________

Definition of NEGATIVE ZIKV ELISA for ascertining infant infection ______________________

Definition of INCONCLUSIVE ZIKV ELISA for ascertining infant infection ______________________

Source of definition used to interpret ZIKV immunoassay test results for ascertining infant infection

Select all that apply
☐ Manufacturer
☐ Ministry of Health reference lab
☐ WHO (global or local office e.g: PAHO)
☐ US-CDC
☐ Consensus/study researchers
☐ Other

Specify other source of definition used to interpret ZIKV immunoassay test results ______________________

How many ZIKV ELISAs did the study plan to conduct in each infant? ______________________

Would your study be able to provide the P/N ratio, titer, or other quantitative readout for ZIKV ELISAs to inform the measurement error models?
◯ Yes
◯ No

For peer review only

Ascertainment of infant ZIKV infection - IFA

ZIKV IFA cut-off value for ascertining infant infection ________________

Was a positive control from the outbreak included for the ZIKV IFA for ascertining infant infection?
- ◯ Yes
- ◯ No

Was a negative control included with every run of ZIKV IFA for ascertining infant infection?
- ◯ Yes
- ◯ No

Definition of POSITIVE ZIKV IFA for ascertining infant infection ________________

Definition of NEGATIVE ZIKV IFA for ascertining infant infection ________________

Definition of INCONCLUSIVE ZIKV IFA for ascertining infant infection ________________

Source of definition for ZIKV IFA test results for ascertining infant infection

Select all that apply
- ☐ Manufacturer
- ☐ Ministry of Health reference lab
- ☐ WHO (global or local office e.g: PAHO)
- ☐ Consensus/study researchers
- ☐ Other

Specify other source of definition for ZIKV IFA test results for ascertining infant infection ________________

Specify any additional inclusion or exclusion criteria for determining which samples to test for ZIKV using IFA for ascertining infant infection ________________

Will your study be able to provide the titer or optical density for ZIKV IFAs to inform the measurement error model?
- ◯ Yes
- ◯ No

For peer review only

Ascertainment of infant ZIKV infection - MIA

ZIKV MIA cut-off value for ascertining infant infection ______________________

Was a positive control from the outbreak included for the ZIKV MIAs for ascertining infant infection?
◯ Yes
◯ No

Was a negative control included with every run of ZIKV MIAs for ascertining infant infection?
◯ Yes
◯ No

Definition of POSITIVE ZIKV MIA for ascertining infant infection ______________________

Definition of NEGATIVE ZIKV MIA for ascertining infant infection ______________________

Definition of INCONCLUSIVE ZIKV MIA for ascertining infant infection ______________________

Source of definition for MIA ZIKV test results for ascertining infant infection

Select all that apply
☐ Manufacturer
☐ Ministry of Health reference lab
☐ WHO (global or local office e.g: PAHO)
☐ Consensus/study researchers
☐ Other

Specify other source of definition for ZIKV MIA test results ______________________

Specify any additional inclusion or exclusion criteria for determining which samples to test for ZIKV using MIA for ascertining infant infection ______________________

Will your study be able to provide the titer for ZIKV MIAs to inform the measurement error model?
◯ Yes
◯ No

For peer review only

Ascertainment of infant ZIKV infection - Immunoassay - PRNT

Did you use the CDC protocol for the PRNT for ascertining infant infection?	◯ Yes<br>◯ No
Describe the other protocol used for the PRNT	__________________________________
End-point PRNT titer for infants	◯ PRNT50<br>◯ PRNT90<br>◯ Other
Which other end-point did you use for the PRNT titer for infant infection?	__________________________________
Definition of a positive PRNT titer for infants	◯ PRNT>10<br>◯ Other
Other definition of a positive PRNT titer for infants	__________________________________
Source of the definition used to interpret ZIKV PRNT test results for infants<br><br>Select all that apply	☐ Manufacturer<br>☐ Ministry of Health reference lab<br>☐ WHO (global or local office e.g: PAHO)<br>☐ US-CDC<br>☐ Consensus/study researchers<br>☐ Other
Specify other source of definition used to interpret ZIKV PRNT test results	__________________________________
Definition of POSITIVE ZIKV PRNT for infants	__________________________________
Definition of NEGATIVE ZIKV PRNT for infants	__________________________________
Definition of INCONCLUSIVE PRNT (i.e. specific flavivirus could not be determined) for infants	__________________________________
Specify any additional inclusion or exclusion criteria for determining which infant samples to test for ZIKV using PRNT	__________________________________
Where was the ZIKV PRNT test performed for samples from infants?<br><br>Select all that apply	☐ Lab at the same health care facility where study is conducted<br>☐ Study-specific lab<br>☐ Ministry of Health reference lab<br>☐ Private external lab<br>☐ Other
Specify other lab where ZIKV PRNT test was performed for samples from infants	__________________________________

For peer review only

How many ZIKV PRNTs were conducted with samples from infants?	__________________________________
Will your study be able to provide the titer for ZIKV PRNTs from infants to inform the measurement error model?	◯ Yes<br>◯ No

For peer review only

Ascertainment of infant ZIKV infection - Molecular assay

Type of ZIKV molecular tests performed in infants<br><br>Select all that apply	☐ conventional PCR<br>☐ rRT-PCR<br>☐ qRT-PCR<br>☐ Other NAAT
According to the study protocol, when were the ZIKV samples to be collected for molecular testing?<br><br>Select all that apply	☐ within 24 hours after birth<br>☐ within first week after birth<br>☐ Other
When were the ZIKV molecular tests (RT-PCR, other NAAT) conducted in infants?	______________________________
Sample used for ZIKV RT-RCR/PCR in infants<br><br>Select all that apply	☐ Serum/plasma<br>☐ Whole blood<br>☐ Urine<br>☐ Saliva<br>☐ Breast milk<br>☐ Umbilical cord tissue<br>☐ cerebrospinal fuid (CSF)<br>☐ chord blood<br>☐ Other
Specify other type of sample used for ZIKV RT-PCR/PCR in infants	______________________________

For peer review only

ZIKV RT-PCR/PCR brand name used for testing infants<br><br>Select all that apply	☐ In house technique<br>☐ Zika Virus RNA Qualitative Real-Time RT-PCR (Quest Diagnostics)<br>☐ RealStar Zika Virus RT-PCR Kit U.S. (altona Diagnostics GmbH)<br>☐ RealStar Zika Virus RT-PCR Kit 1.0 (altona Diagnostics GmbH)<br>☐ Aptima Zika Virus assay (Hologic Inc)<br>☐ Zika Virus Real-time RT-PCR (Viracor Eurofins)<br>☐ VERSANT Zika RNA 1.0 Assay (kPCR) (Siemens Healthcare Diagnostics Inc)<br>☐ xMAP MultiFLEX Zika RNA Assay (Luminex Corporation)<br>☐ Sentosa SA ZIKV RT-PCR Test (Vela Diagnostics Inc)<br>☐ Zika Virus Detection by RT-PCR (ARUP Laboratories)<br>☐ Abbott RealTime Zika (Abbott Molecular Inc)<br>☐ Zika ELITe MGB Kit U.S. (ELITechGroup Molecular Diagnostics)<br>☐ Gene-RADAR Zika Virus Test (Nanobiosym Diagnostics Inc)<br>☐ TaqPath Zika Virus Kit (Thermo Fisher Scientific)<br>☐ CII-ArboViroPlex rRT-PCR assay (Columbia University)<br>☐ Trioplex Real-Time RT-PCR Assay (CDC)<br>☐ Liferiver Zika Virus (ZIKV) Real Time RT-PCR Kit (Shanghai ZJ Bio-Tech Co Ltd)<br>☐ careGENE Zika Virus RT-PCR Kit (WELLS BIO Inc)<br>☐ Zika Virus - Single Check (Genekam Biotechnology AG)<br>☐ FTD Zika virus (Fast Track Diagnostics)<br>☐ VIASURE Zika Virus Real Time PCR Detection Kit (Certest Biotec)<br>☐ Zika Virus Real-TM (Sacace Biotechnologies)<br>☐ Logix Smart Zika Test Kit (Co-Diagnostics Inc)<br>☐ Genesig Kits for ZIKV (Primerdesign Ltd)<br>☐ Genesig Multiplex Kit for Dengue, Chikungunya and Zika Virus (Primerdesign Ltd)<br>☐ AccuPower ZIKV (DENV, CHIKV) Multiplex Real-Time RT-PCR Kit (Bioneer)<br>☐ VIASURE Zika, Dengue & Chikungunya Real Time PCR Detection Kit (Certest Biotec)<br>☐ FTD Zika/Dengue/Chik (Fast-Track Diagnostics)<br>☐ DiaPlexQ ZCD (ZIKV, CHIKV, DENV) Detection Kit (SolGent Co Ltd)<br>☐ TaqMan Zika Virus Triplex Kit (Thermo Fisher Scientific)<br>☐ GenoAmp Trioplex Real-Time RT-PCR Zika/Den/Chiku (Medical Innovation Ventures)<br>☐ Other
Describe procedure for in house ZIKV RT-PCR/PCR for ascertining infant infection	__________________________________
Specify other ZIKV RT-PCR/PCR brand name	__________________________________
Did the study obtain a measure of ZIKV viral load in infants?	◯ Yes<br>◯ No
ZIKV PCR/RT-PCR cut-off point in infants	____________________________

For peer review only

Was a negative control included with every run of ZIKV RT-PCR/PCRs?	◯ Yes<br>◯ No
Definition of POSITIVE ZIKV RT-PCR/PCR in infants	__________________________________
Definition of NEGATIVE ZIKV RT-PCR/PCR in infants	__________________________________
Definition of INCONCLUSIVE ZIKV RT-PCR/PCR in infants	__________________________________
Source of definition used to interpret ZIKV RT-PCR/PCR test results<br><br>Select all that apply	☐ Manufacturer<br>☐ Ministry of Health reference lab<br>☐ WHO (global or local office e.g: PAHO)<br>☐ US-CDC<br>☐ Consensus/study researchers<br>☐ Other
Specify other source of definition used to interpret ZIKV RT-PCR/PCR test results	__________________________
Where was the ZIKV RT-PCR/PCR test performed for samples from infants?<br><br>Select all that apply	☐ Lab at the same health care facility where study is conducted<br>☐ Study-specific lab<br>☐ Ministry of Health reference lab<br>☐ Private external lab<br>☐ Other
Specify other lab where ZIKV RT-PCR/PCR test was performed for samples from infants	__________________________
Specify any additional inclusion or exclusion criteria for determining which samples from infants to test for ZIKV using RT-PCR/PCR	__________________________________
How many ZIKV PCRs were conducted with infants?	__________________________
Would your study be able to provide a Ct value for infant ZIKV PCRs to inform the measurement error models?	◯ Yes<br>◯ No

For peer review only

Changes in diagnosis of infant ZIKV infection over time

Which clinical criteria did you apply initially to diagnose infant infection? (Clinical Definition 1) ______________________

Did your clinical criteria for infant infection change over time?
- ◯ No
- ◯ Yes - changed 1x
- ◯ Yes - changed more than 1x

If your clinical criteria changed over time, which criteria did you apply subsequently to assess infant infection? (Clinical Definition 2) ______________________

When did you begin using the revised clinical criteria for assessing infant infection? (Clinical Definition 2) ______________________

What was the third set of criteria you applied to assess infant infection? (Clinical Definition 3) ______________________

When did you begin using the third set of clinical criteria? (Clinical Definition 3) ______________________

Which imaging criteria did you apply initially to diagnose infant infection? (Clinical Definition 1) ______________________

Did your imaging criteria for infant infection change over time?
- ◯ No
- ◯ Yes - changed 1x
- ◯ Yes - changed more than 1x

If your imaging criteria changed over time, which criteria did you apply subsequently to assess infant infection? (Clinical Definition 2) ______________________

When did you begin using the revised imaging criteria for assessing infant infection? (Clinical Definition 2) ______________________

What was the third set of imaging criteria you applied to assess infant infection? (Clinical Definition 3) ______________________

When did you begin using the third set of imaging criteria? (Clinical Definition 3) ______________________

What laboratory criteria was used initially to diagnose infant ZIKV infection? (Laboratory Definition 1) ______________________

Did your laboratory criteria for diagnosing infant infection change over time?
- ◯ No
- ◯ Yes - changed 1x
- ◯ Yes - changed more than 1x

For peer review only

Which laboratory criteria did you apply subsequently? (Laboratory Definition 2)	__________________________________
When did you begin using the revised lab criteria for infant infection? (Laboratory Definition 2)	___________________________
What was the third set of laboratory criteria you applied for diagnosing infant infection? (Laboratory Definition 3)	__________________________________
When did you begin using the third set of lab criteria for infant infection? (Laboratory Definition 3)	___________________________
Were infant samples retested using the new laboratory criteria?	__________________________________

For peer review only

ZIKV testing algorithm & test interpretation - infant ZIKV infection

Describe the testing algorithm for defining a confirmed ZIKV+ test in an infant
____

Describe the testing algorithm for defining a confirmed ZIKV- test in an infant
____

What tests were used to confirm ZIKV infection in an infant?

Select all that apply

- ☐ ZIKV IgM - 1 sample
- ☐ ZIKV IgM - paired samples (seroconversion or increased titers)
- ☐ ZIKV IgM (1 sample) + IgG
- ☐ ZIKV IgM (paired samples) + IgG
- ☐ RT-PCR or other NAAT
- ☐ PRNT > 10
- ☐ Other

Specify other test or combination of tests used to confirm ZIKV infection in an infant
____

Considering any relevant clinical and laboratory criteria, what was the study's definition of a CONFIRMED infant ZIKV Infection
____

Considering any relevant clinical and laboratory criteria, what was the study's definition of a PROBABLE infant ZIKV Infection
____

Considering any relevant clinical and laboratory criteria, what was the study's definition of a NEGATIVE/UNLIKELY infant ZIKV Infection
____

Source of ZIKV test result categorization

Select all that apply

- ☐ Manufacturer
- ☐ Ministry of Health reference lab
- ☐ WHO (global or local office e.g: PAHO)
- ☐ Consensus/study researchers
- ☐ Other

Specify other source of ZIKV test result categorization
____

For peer review only

ZIKV Diagnostics external quality assessment (EQA)

Did your study conduct ZIKV test-related external quality assessment (EQA)?
◯ Yes
◯ No

Where was the EQA conducted?
◯ Lab at the same health care facility where study is conducted
◯ Study-specific lab
◯ Ministry of Health reference lab
◯ Private external lab
◯ Other

Where else was the EQA conducted?
____

Which group conducted the EQA?
____

Which tests were evaluated in the EQA?

Select all that apply
☐ ZIKV ELISA
☐ ZIKV PCR/RT-PCR
☐ Other

Which other tests were evaluated in the EQA?
____

Which assay or assays was/were used as a comparator for the ELISA EQA?
____

Sensitivity of ZIKV ELISA test
____
(Provide as percentage)

Specificity of ZIKV ELISA test
____
(Provide as percentage)

PPV of the ZIKV ELISA test
____
(Provide as percentage)

ZIKV prevalence used to estimate the PPV
____

Sample (N) for ELISA EQA
____

How many ELISAs, either IgG or IgM, were included in the EQA?
____

Which assay or assays was/were used as a comparator for the PCR/RT-PCR EQA?
____

For peer review only

Sensitivity of ZIKV PCR/RT-PCR test

____
(Provide as percentage)

Specificity of ZIKV PCR/RT-PCR test

____
(Provide as percentage)

PPV of the ZIKV PCR/RT-PCR test

____
(Provide as percentage)

ZIKV prevalence used to estimate the PPV

____

How many molecular tests (PCR/RT-PCR) were included in the EQA?

____

Describe the procedure used to resolve inconsistencies in the EQA

____

Was a positive control from the outbreak included in the EQA?

◯ Yes
◯ No

Was a negative control from the outbreak included in the EQA?

◯ Yes
◯ No

Please describe any additional details of the EQA

____

List any publications related to the EQA described above

____

For peer review only

Perinatal Outcomes

How did the study measure gestational age at birth?	◯ last menstrual period (LMP)<br>◯ ultrasound<br>◯ LMP if ultrasound unavailable, otherwise ultrasound<br>◯ Other<br>◯ Study did not measure gestational age at birth
Describe other means of ascertaining gestational age at birth	__________________________________
Did the study include a newborn maturity assessment?<br><br>Select all that apply	☐ Ballard<br>☐ Dubowitz<br>☐ Capurro<br>☐ Other<br>☐ No, study did not include newborn maturity assessment
Specify other maturity assessment of newborn	____________________________
How did the study determine preterm birth?<br><br>Select all that apply	☐ Gestational age - from ultrasound<br>☐ Gestational age - from LMP<br>☐ Gestational age from LMP if ultrasound not available, otherwise from ultrasound<br>☐ Maturity assessment<br>☐ Other<br>☐ Study did not assess preterm birth
Specify other determination of preterm birth	____________________________
How did the study define preterm birth?	__________________________________<br>((e.g. extremely preterm (< 28 weeks); very preterm (28-32 weeks), moderate to late preterm (32-37 weeks)))
How did the study determine termination of pregnancy (TOP)/induced abortion?	◯ Self report<br>◯ Medical records<br>◯ Other<br>◯ Study did not measure TOP/induced abortion
Specify other means of determining termination of pregnancy/induced abortion	__________________________________
Provide wording of question related to TOP/induced abortion for self report or provide directions for extracting TOP-related information from medical chart	__________________________________
How did the study define miscarriage?	◯ Pregnancy loss < 20 weeks regardless of weight<br>◯ Pregnancy loss < 20 weeks or weight < 500 gr<br>◯ Involuntary loss < 20 weeks or voluntary loss between 10-20 weeks (Cuban definition)<br>◯ Other<br>◯ Study did not measure miscarriage

For peer review only

Specify other definition of miscarriage	__________________________
How did the study define fetal loss or fetal death?	◯ Any loss < 20 weeks regardless of weight<br>◯ Any loss < 22 weeks or weight < 500 gr<br>◯ Early (20-27 weeks) and late (>28 weeks) fetal loss regardless of weight<br>◯ Other<br>◯ Study did not measure fetal loss
Specify other definition of fetal loss or fetal death	__________________________
Did the study document placental insufficiency as an outcome?	◯ Yes<br>◯ No
Definition of placental insufficiency	__________________________
Did the study document intrauterine growth restriction (IGR) as an outcome?	◯ Yes<br>◯ No
Definition of intrauterine growth restriction	__________________________
Did the study document low birth weight (LBW) as an outcome?	◯ Yes<br>◯ No
How did the study define low birthweight?	______________________
How did the study define very low birthweight?	______________________
How did the study define extremely low birthweight?	______________________

For peer review only

For peer review only - http://bmjopen.bmj.com/site/about/guidelines.xhtml

Postnatal ZIKV-related imaging & microcephaly

Were postnatal cranial ultrasounds performed?
◯ Yes
◯ No

How many postnatal cranial ultrasounds did the study plan to conduct with each infant/child?
____

Age (months) when the study planned to conduct the postnatal cranial ultrasounds
____
(e.g., 1, 3, 6 months)

Postnatal cranial ultrasound machine models (list all that apply)
____
(Write "same as above" if ultrasound machine models are same as those described in prenatal ultrasound section.)

Place where postnatal cranial ultrasound(s) were performed

Select all that apply
☐ Same health care facility where study is taking place
☐ Study-specific site (not a health care facility)
☐ Private/external site
☐ Other

Other place that the postnatal cranial ultrasound(s) were performed
____

Training received by ultrasound technician performing postnatal cranial ultrasounds
____
(Write "same as above" if technician training same as those described in prenatal ultrasound section.)

Quality control procedures for ultrasound evaluation of abnormalities/head circumference
____
(Write "same as above" if quality control measures same as those described in prenatal ultrasound section.)

What other types of postnatal ultrasound did the study conduct?

Select all that apply
☐ Thoracic
☐ Abdominal
☐ Pelvic
☐ Osteo muscular
☐ Other
☐ No other types of postnatal ultrasound

Specify other type of additional postnatal ultrasound
____

Did the study perform postnatal brain/head/neck MRIs?
◯ Yes
◯ No

How many postnatal brain/head/neck MRIs did the study plan to conduct with each infant/child?
____

For peer review only

What other types of postnatal MRI did the study conduct?<br><br>Select all that apply	☐ Thoracic<br>☐ Abdominal<br>☐ Pelvic<br>☐ Osteo muscular<br>☐ Other<br>☐ No other types of postnatal MRI
Specify other type of additional postnatal MRI	__________________
Age (months) when the study planned to conduct any postnatal MRI	__________________<br>(e.g. 1, 3, 6, 9 months)
Describe any conditions for application of any postnatal MRI	__________________
Place where postnatal MRI was performed	◯ Same health care facility where study is taking place<br>◯ Study-specific site (not a health care facility)<br>◯ Private/external site
Training received by postnatal MRI technician	__________________<br>(Write "same as above" if technician training same as that described in prenatal MRI section.)
Quality control for evaluation of postnatal MRI	__________________<br>(Write "same as above" if quality control measures same as those described in prenatal MRI section.)
Was postnatal microcephaly an outcome in this study?	◯ Yes<br>◯ No
At what age(s) (in months) was postnatal microcephaly assessed?	__________________<br>(e.g., 1, 3, 6 months)
How was postnatal microcephaly diagnosed?<br><br>Select all that apply	☐ Presence vs absence<br>☐ By grades (normocephaly vs microcephaly vs severe microcephaly)<br>☐ By percentiles<br>☐ By Z-score<br>☐ By diagnostic certainty criteria<br>☐ Other
Specify other postnatal microcephaly diagnosis type	__________________

For peer review only

How did the study define postnatal microcephaly?	◯ >2 SD below the mean<br>◯ < 3 SD below the mean<br>◯ 3 SD below the mean<br>◯ >3 SD below the mean<br>◯ below the 5th percentile<br>◯ below the 10th percentile<br>◯ Other
Specify other definition of postnatal microcephaly	__________________________________
How did the study define postnatal microcephaly?	◯ Level 1 of diagnostic certainty<br>◯ Level 2A of diagnostic certainty<br>◯ Level 2B of diagnostic certainty<br>◯ Level 3A of diagnostic certainty<br>◯ Level 3B of diagnostic certainty<br>◯ Level 4 of diagnostic certainty<br>◯ Other
Specify other definition of postnatal microcephaly	____________________________
Did the study measure infant head circumference	◯ Yes<br>◯ No
Device used to measure infant head circumference<br><br>Select all that apply	☐ Flexible-non-stretchable measuring tape-marked by 0.1 cm increments<br>☐ Metal tape<br>☐ Paper tape<br>☐ Other<br>☐ Unknown
Specify other device used to measure infant head circumference	__________________________________
Reference standards used to calculate infant head circumference Z score<br><br>Select all that apply	☐ Intergrowth<br>☐ Ministry of Health standards<br>☐ WHO or PAHO standard<br>☐ Local reference standard<br>☐ Consensus/study researchers<br>☐ Other
Other reference standards used to calculate infant head circumference Z score	____________________________
Was epilepsy screening performed?	◯ Yes<br>◯ No
How did the study define epilepsy?	__________________________________
How many times did the study plan to screen the infant or child for epilepsy?	____________________________

For peer review only

List ages (in months) at which the study planned to screen for epilepsy	__________________
Was postnatal electroencephalography (EEG) performed?	◯ Yes<br>◯ No
How many EEGs did the study plan to conduct with each infant or child?	__________________
Were the EEGs reviewed for abnormalities by epileptologists who were blinded to the ZIKV status of the mother?	◯ Yes<br>◯ No

For peer review only

Ascertainment of congenital Zika syndrome (CZS)

Did the study measure congenital Zika syndrome (CZS)?
◯ Yes
◯ No

When did the study assess CZS?

Select all that apply
☐ Antenatal
☐ Peri/intrapartum
☐ Postpartum

When, during pregnancy, did the study assess antenatal CZS?

Select all that apply
☐ 1st trimester
☐ 2nd trimester
☐ 3rd trimester

Time of CZS assessment in hours after delivery for peri/intrapartum assessment
____
(Please list all times, separated by a comma)

Time of CZS assessment in days after delivery for postpartum diagnosis of CZS
____
(Please list all times, separated by a comma)

Which criteria were used to assess CZS?

Select all that apply
☐ maternal clinical
☐ maternal laboratory
☐ placental laboratory
☐ fetal clinical
☐ fetal laboratory
☐ fetal neuroimaging
☐ infant clinical
☐ infant laboratory
☐ infant neuroimaging
☐ developmental
☐ other

Which other criteria were used to assess CZS?
____

Infant or fetal neurologic alterations used to assess CZS

Select all that apply
☐ Epilepsy
☐ Seizures
☐ Dysphagia
☐ Hypertonia
☐ Hypotonia
☐ Hyperreflexia
☐ Hyporeflexia
☐ Other neurologic abnormality

Which other infant or fetal neurological criteria were used to assess CZS?
____

Infant or fetal structural abnormalities used to assess CZS

Select all that apply
☐ Cranial shape malformation
☐ Microcephaly
☐ Abnormalities of intracranial anatomy
☐ Musculoskeletal defects
☐ Eye defects
☐ Hearing (ENT) defects
☐ Other structural abnormality

For peer review only

Which other infant or fetal structural criteria were considered in assessing CZS?	__________________________
What type of microcephaly diagnosis was used to diagnose CZS?<br><br>Select all that apply	☐ Antenatal microcephaly<br>☐ Postnatal microcephaly<br>☐ Both antenatal and postnatal microcephaly<br>☐ Other
What other type of microcephaly diagnosis was used to diagnose CZS?	__________________________
Which intracranial abnormalities were considered in assessing CZS?<br><br>Select all that apply	☐ Diffuse, primarily subcortical calcifications<br>☐ Increased fluid spaces - ventricular and extra-axial<br>☐ Marked cortical thinning with abnormal gyral patterns<br>☐ Hypoplasia or absence of corpus callosum<br>☐ Anencephaly<br>☐ Holoprosencephaly<br>☐ Decreased myelination<br>☐ Cerebellar or cerebellar vermis hypoplasia<br>☐ Calcifications in basal ganglion/brainstem<br>☐ Other
What other intracranial abnormalities were considered in assessing CZS?	__________________________
Maternal clinical criteria used to assess CZS	◯ Fever only<br>◯ Rash only<br>◯ Fever + rash<br>◯ Fever or rash<br>◯ Fever + rash + joint pain<br>◯ Fever + rash + joint pain + conjunctivitis<br>◯ Other<br>◯ Fever + rash + other
Which other maternal clinical criterion or combination of criteria were used to assess CZS	__________________________
Fetal clinical criteria used to assess CZS	◯ Fever only<br>◯ Rash only<br>◯ Fever + rash<br>◯ Fever or rash<br>◯ Fever + rash + joint pain<br>◯ Fever + rash + joint pain + conjunctivitis<br>◯ Other<br>◯ Fever + rash + other
Which other fetal clinical criterion or combination of criteria were used to assess CZS	__________________________

For peer review only

Infant clinical criteria used to assess CZS	◯ Fever only<br>◯ Rash only<br>◯ Fever + rash<br>◯ Fever or rash<br>◯ Fever + rash + joint pain<br>◯ Fever + rash + joint pain + conjunctivitis<br>◯ Other<br>◯ Fever + rash + other
Which other infant clinical criterion or combination of criteria were used to assess CZS	__________________________________
Maternal laboratory diagnostics used to assess CZS<br><br>Select all that apply	☐ Molecular diagnosis (e.g. RT-PCR, other NAAT)<br>☐ Antibody-based immunoassay (e.g. ELISA, IFA, PRNT, microneutralization)<br>☐ Antigen-based immunoassay (e.g. ELISA, IFA)<br>☐ Rapid diagnostic test (RDT)<br>☐ Other
Which other maternal laboratory diagnostics were used to assess CZS?	___________________________
Placental laboratory diagnostics used to assess CZS<br><br>Select all that apply	☐ Molecular diagnosis (e.g. RT-PCR, other NAAT)<br>☐ Antibody-based immunoassay (e.g. ELISA, IFA, PRNT, microneutralization)<br>☐ Antigen-based immunoassay (e.g. ELISA, IFA)<br>☐ Rapid diagnostic test (RDT)<br>☐ Other
Which other placental laboratory diagnostics were used to assess CZS?	___________________________
Fetal laboratory diagnostics used to assess CZS<br><br>Select all that apply	☐ Molecular diagnosis (e.g. RT-PCR, other NAAT)<br>☐ Antibody-based immunoassay (e.g. ELISA, IFA, PRNT, microneutralization)<br>☐ Antigen-based immunoassay (e.g. ELISA, IFA)<br>☐ Rapid diagnostic test (RDT)<br>☐ Other
Which other fetal laboratory diagnostics were used to assess CZS?	___________________________
Infant laboratory diagnostics used to assess CZS<br><br>Select all that apply	☐ Molecular diagnosis (e.g. RT-PCR, other NAAT)<br>☐ Antibody-based immunoassay (e.g. ELISA, IFA, PRNT, microneutralization)<br>☐ Antigen-based immunoassay (e.g. ELISA, IFA)<br>☐ Rapid diagnostic test (RDT)<br>☐ Other
Which other infant laboratory diagnostics used to assess CZS?	___________________________
Fetal neuroimaging used to assess CZS<br><br>Select all that apply	☐ Ultrasound<br>☐ MRI<br>☐ CT scan<br>☐ Other

For peer review only

Specify other neuroimaging used to assess CZS in fetus	__________________________________
Infant neuroimaging used to assess CZS<br><br>Select all that apply	☐ Ultrasound<br>☐ MRI<br>☐ CT scan<br>☐ Other
Specify other neuroimaging used to assess CZS in infant	__________________________________
Which types of developmental delays or impairments were included in the CZS assessment?<br><br>Select all that apply	☐ motor<br>☐ cognitive<br>☐ speech<br>☐ other
Which other types of developmental delays or impairments were included in the CZS assessment?	__________________________________
Did your study apply the following criteria for CZS phenotype?<br><br>Severe microcephaly with partially collapsed skull Thin cerebral cortices with subcortical calcifications Macular scarring and focal pigmentary retinal mottling Congenital contractures Marked early hypertonia and symptoms of extrapyramidal involvement	◯ Yes<br>◯ No
Reference standard used to define CZS	◯ WHO guidelines 2015<br>◯ WHO guidelines 2016<br>◯ WHO guidelines 2017<br>◯ Current country guidelines (Ministry of Health)<br>◯ Study definition<br>◯ Other
Specify other reference standards used to define CZS	__________________________________
Specify study definition used to define CZS	__________________________________
Study's definition of a CONFIRMED case of CZS	__________________________________
Study's definition of a PROBABLE case of CZS	__________________________________
Study's definition of an UNLIKELY case of CZS	__________________________________

For peer review only

Genetic abnormalities

Did the study conduct prenatal genetic screening or testing?	◯ Yes<br>◯ No
Did the study conduct genetic testing on products of conception in cases of termination or fetal loss?	◯ Yes<br>◯ No
How was prenatal genetic screening conducted?<br><br>Select all that apply	☐ FTS (first trimester screening, standard or enhanced, maternal blood screen + ultrasound)<br>☐ only blood screening (maternal serum screening)<br>☐ only ultrasound findings (including Nuchal Translucency Ultrasound)<br>☐ cfDNA/NIPT (Non-invasive prenatal testing)<br>☐ Amniocentesis<br>☐ chorionic villus sampling<br>☐ Triple screen (with AFP)<br>☐ Quad screen (with AFP)<br>☐ other
What other type of genetic screening was conducted?	__________________________________
What type of prenatal genetic testing was conducted?<br><br>Select all that apply	☐ cfDNA/NIPT<br>☐ FISH<br>☐ karyotype<br>☐ chromosomal microarray<br>☐ single gene<br>☐ gene panel<br>☐ other
What other type of genetic testing was conducted?	__________________________________

For peer review only

Maternal and infant C reactive protein

Did the study collect a measure of maternal C-reactive protein	◯ Yes<br>◯ No
Was C-reactive protein in the mother reported as a binary or continuous measure?	◯ binary<br>◯ continuous
What was the cut off for the binary measure of C-reactive protein in the mother?	__________________________________
How many times did the study plan to measure C-reactive protein for each pregnant woman?	__________________________________
Did the study collect a measure of infant C-reactive protein?	◯ Yes<br>◯ No
Was C-reactive protein in the infant reported as a binary or continuous measure?	◯ binary<br>◯ continuous
What was the cut off for the binary C-reactive protein measure in the infant?	__________________________________
How many times did the study plan to measure C-reactive protein for each infant?	__________________________________
At what ages did the study plan to measure C-reactive protein for each infant?	__________________________________
How was C-reactive protein measured in the infant?	◯ Immunoturbidimetric<br>◯ Nephelometry

For peer review only

For peer review only - http://bmjopen.bmj.com/site/about/guidelines.xhtml

Ophthalmological exams

Did the study measure ocular abnormalities?	◯ Yes<br>◯ No
How did the study measure ocular abnormalities?<br><br>Select all that apply	☐ fundoscopic exam<br>☐ RetCam<br>☐ other
What other exam did the study use to measure ocular abnormalities?	__________________________________
Type of eye abnormalities measured in the study<br><br>Select all that apply	☐ Macular findings: Focal pigmentary mottling<br>☐ Macular findings: Chorioretinal atrophy<br>☐ Optic nerve findings: Optic nerve hypoplasia<br>☐ Optic nerve findings: Optic nerve cupping<br>☐ Optic nerve findings: Optic nerve atrophy<br>☐ Other retinal lesions: subretinal hemorrhage<br>☐ Hypopigmentation<br>☐ Vascular tortuosity<br>☐ Abnormal termination of the vessels<br>☐ Focal vascular dilation<br>☐ Congenital glaucoma<br>☐ Iris colobomas<br>☐ Microphthalmia<br>☐ Cataracts<br>☐ Subluxation of the lens<br>☐ Intraocular calcification<br>☐ Other
Specify other eye abnormalities measured by the study	__________________________
Were any of the following exams performed?<br><br>Select all that apply	☐ Cardiff acuity test<br>☐ Cardiff visual contrast test<br>☐ Teller test<br>☐ Other
Specify any other visual acuity tests performed by the study	__________________________
At what age(s) did the study plan to measure ophthalmological function?	__________________________
Reference standard used for evaluation of fundoscopic exam<br><br>Select all that apply	☐ Provided by manufacturer<br>☐ In-hospital standard<br>☐ Regional or state standard<br>☐ Federal standard<br>☐ WHO or local branch<br>☐ Other
Specify other basis for evaluation of fundoscopic exam	__________________________

For peer review only

Reference standard used for evaluation of RetCam exam<br><br>Select all that apply	☐ Provided by manufacturer<br>☐ In-hospital standard<br>☐ Regional or state standard<br>☐ Federal standard<br>☐ WHO or local branch<br>☐ Other
Specify other basis for evaluation of RetCam opthamological exam	__________________________

For peer review only

Auditory abnormalities

Did the study measure auditory abnormalities?	◯ Yes<br>◯ No
How did the study measure auditory abnormalities?<br><br>Select all that apply	☐ impedance audiometry<br>☐ otoacoustic emissions (OAEs)<br>☐ other
How else did the study measure auditory abnormalities?	__________________________________
Was impedance audiometry performed at birth or within the first 24-48 hours after birth?	◯ Yes<br>◯ No
Type of information used to assess auditory abnormalities	◯ Presence vs absence of alterations<br>◯ Severity of nerve damage<br>◯ Other
Specify other type of information used to assess auditory abnormalities	____
At what age(s) did the study plan to measure auditory function?	__________________________________
Did the study perform an Auditory Brainstem Response (ABR) test?	◯ Yes<br>◯ No
How many times did the study plan to perform ABR on each infant/child?	__________________________________
At what ages did the study perform ABR?	__________________________________
Specify any other auditory tests performed by study	__________________________________
Reference standard used for evaluation of impedance audiometry exam<br><br>Select all that apply	☐ Provided by manufacturer<br>☐ In-hospital standard<br>☐ Regional or state standard<br>☐ Federal standard<br>☐ WHO or local branch<br>☐ Other
Specify other reference standard used	____
Reference standard used for evaluation of OAE<br><br>Select all that apply	☐ Provided by manufacturer<br>☐ In-hospital standard<br>☐ Regional or state standard<br>☐ Federal standard<br>☐ WHO or local branch<br>☐ Other

For peer review only

Specify other reference standard used

For peer review only

Additional abnormalities

In addition to the ocular and auditory outcomes described previously, which other infant outcomes were assessed by the study?

Select all that apply

- ☐ Motor abnormalities (hypotonia, hypertonia, hyperreflexia, spasticity, clonus, extrapyramidal symptoms)
- ☐ Congenital contractures (arthrogryposis, uni or bilateral clubfoot)
- ☐ Craniofacial disproportion
- ☐ Seizures, epilepsy
- ☐ Postnatal intraventricular hemorrhage
- ☐ Other non-neurologic congenital abnormalities

How were congenital contractures defined?

____

Which other non-neurologic congenital abnormalities were assessed by the study?

____

For peer review only

Developmental screening tools

Study administered the Bayley Scales of Infant Development (BSID)?	◯ Yes<br>◯ No
Who administered the Bayleys?	◯ Pediatrician<br>◯ Neuropediatrician<br>◯ Other
Who else administered the Bayleys?	__________________________________
Describe the training of the person who administered the Bayleys	____
Was the person evaluating the Bayleys blinded to ZIKV status of the mother?	◯ Blinded<br>◯ Unblinded
How many times did the study plan to administer the Bayleys with each infant or child?	__________________________________
List ages (in months) at which the study planned to administer the Bayleys:	__________________________________
Did the study assess all 5 domains measured by the Bayleys? (i.e., receptive language, expressive language, fine motor, gross motor, and cognitive)	◯ Yes<br>◯ No
If all 5 domains were not assessed, which of the Bayleys scales were administered?<br><br>Select all that apply	☐ Receptive language<br>☐ Expressive language<br>☐ Fine motor<br>☐ Gross motor<br>☐ Cognitive
Study administered the Ages and Stages Questionnaire (ASQ)?	◯ Yes<br>◯ No
Was the person administering the ASQ blinded to the ZIKV status of the mother?	◯ Blinded<br>◯ Unblinded
How many times did the study plan to administer the ASQ with each infant or child?	__________________________________
List all ages (in months) at which the study planned to adminster the ASQ	__________________________________
Study administered the Warner Initial Developmental Evaluation of Adaptive and Functional Skills (WIDEA-FS)?	◯ Yes<br>◯ No
Was the person administering the WIDEA-FS blinded to the ZIKV status of the mother?	◯ Blinded<br>◯ Unblinded
How many times did the study plan to administer the WIDEA-FS with each child?	__________________________________

For peer review only

List all ages (in months) at which the study planned to adminster the WIDEA-FS	__________________
Study administered the Alberta Infant Motor Skills (AIMS) assessment?	◯ Yes<br>◯ No
Describe how the person evaluating the AIMS was trained	______________________
Who administered the AIMS?	◯ Pediatrician<br>◯ Neuropediatrician<br>◯ Other
Who else administered the AIMS?	__________________
Was the person administering the AIMS blinded to the ZIKV status of the mother?	◯ Blinded<br>◯ Unblinded
How many times did the study plan to administer the AIMS with each infant or child?	__________________
List all ages (in months) at which the study planned to adminster the AIMS	__________________
Study administered the Oxford Neurodevelopmental Assessment (Ox-NDA)?	◯ Yes<br>◯ No
Describe the training of the person administering the Ox-NDA	______________________
Was the person administering the Ox-NDA blinded to the ZIKV status of the mother?	◯ Blinded<br>◯ Unblinded
How many times did the study plan to administer the Ox-NDA with each infant or child?	__________________
List all ages (in months) at which the study planned to adminster the Ox-NDA	__________________
Study administered the Intergrowth-21st Neurodevelopmental Assessment (INTER-NDA)?	◯ Yes<br>◯ No
Describe the training of the person administering the INTER-NDA	______________________
Was the person administering the INTER-NDA blinded to the ZIKV status of the mother?	◯ Blinded<br>◯ Unblinded
List all ages (in months) at which the study planned to adminster the INTER-NDA	__________________

For peer review only

In addition to the instruments mentioned above, which of the following developmental screening tools did the project administer?<br><br>Select all that apply	☐ BDI (Batelle Developmental Inventory) 0-7 yrs<br>☐ BITSEA/ITSEA (Brief) Infant-Toddler Social and Emotional Assessment 1-3 yrs<br>☐ CDI (MacArthur-Bates Communication Development Inventory), 8-30 months<br>☐ DDST or DENVER II (Denver Developmental Screening Test) 0-6 yrs<br>☐ DECA (Devereaux Early Childhood Assessment) 1 months-6 years<br>☐ GMFCS (Gross Motor Function Classification System) 0-18 yrs<br>☐ Greenspan Social-Emotional Growth Chart 0-42 months<br>☐ HELP (Hawaii Early Learning Profile) 0-3 yrs<br>☐ HOME (Home Observation Measurement of the Environment) 0 and up<br>☐ MSEL (Mullen Scales of Early Learning) 0-7 yrs<br>☐ VABS (Vineland Adaptive Behavior Scales) 0 and up<br>☐ PDDST (Pervasive Developmental Disorders Screening Test) 1-4 yrs<br>☐ PEDI (Pediatric Evaluation of Disability Inventory) 6 months-7.5 yrs<br>☐ Other<br>☐ No additional infant development screening tools were administered by the project
Specify other infant developmental screening tools administered by project	__________________________________
Did the project administer any of the following development screening tools for toddlers and older?<br><br>Select all that apply	☐ BSRA (Bracken School Readiness Assessment) 2.6 years and up<br>☐ CBCL (Achenbach Child Behavior Checklist) 6-18 years<br>☐ SDQ (Strengths and Difficulties Questionnaire) 3-16 years<br>☐ Other<br>☐ No other toddler-and-above development screening tools were administered by the project than those mentioned previously
Specify other toddler developmental screening tools administered by project	__________________________________

For peer review only

Sociodemographic variables and maternal mental health

Did the study measure maternal ethnicity or minority status?
- ◯ Yes, self defined
- ◯ Yes, interviewer or study defined
- ◯ Yes, as reported in participant medical record
- ◯ No, not measured

Did the study measure household SES?
- ◯ Yes, household income (self reported)
- ◯ Yes, country-level measure of SES (self reported)
- ◯ Yes, country-level measure of SES (verified)
- ◯ Other
- ◯ No, not measured

Specify other measurement of household SES

____

Did the study measure maternal education?
- ◯ Yes
- ◯ No

Did the study measure maternal occupation?
- ◯ Yes
- ◯ No

Did the study measure maternal marital status?
- ◯ Yes
- ◯ No

List any maternal mental health scales that were administered by the study

Select all that apply
- ☐ BAI (Beck Anxiety Inventory)
- ☐ BDI (Beck Depression Inventory)
- ☐ BDI-II (Beck Depression Inventory II)
- ☐ CBS (Caregiver Burden Scale)
- ☐ CES-D (Center for Epidemiologic Studies Depression Scale)
- ☐ DASS-21 (Depression, Anxiety, and Stress Scale - Short Form)
- ☐ Patient Health Questionnaire-9 (PHQ-9)
- ☐ Patient Health Questionnaire-2 (PHQ-2)
- ☐ Edinburgh Postnatal Depression Scale (EPDS)
- ☐ MSPSS (Multidimensional Scale of Perceived Social Support)
- ☐ PSI (Parenting Stress Index)
- ☐ PSI-SF (Parenting Stress Index - Short Form)
- ☐ WHOQOL-BREF (WHO Quality of Life)
- ☐ Other
- ☐ No maternal mental health or parenting stress scales were administered by the project

Specify other maternal mental health screening tools administered by the study

____

For peer review only - http://bmjopen.bmj.com/site/about/guidelines.xhtml

Pre-pregnancy-related covariates

Did the study measure pre-pregnancy maternal exposure to clinically suspected arboviruses, such as dengue and chikungunya, with specific or proxy dates?	◯ Yes, self reported<br>◯ Yes, medical/clinical chart<br>◯ No, not measured
Did the study measure pre-pregnancy maternal exposure to workplace teratogens?	◯ Yes, self reported<br>◯ Yes, medical/clinical chart<br>◯ No, not measured
Did the study measure pre-pregnancy exposure to intimate partner violence?	◯ Yes, self reported<br>◯ Yes, medical/clinical chart<br>◯ No, not measured
Did the study measure number of previous live births (parity)?	◯ Yes, self reported<br>◯ Yes, medical/clinical chart<br>◯ No, not measured
Did the study measure the number of previous total pregnancies (gravidity)?	◯ Yes, self reported<br>◯ Yes, medical/clinical chart<br>◯ No, not measured
Did the study measure the number of previous pregnancies with any complications	◯ Yes, self reported<br>◯ Yes, medical/clinical chart<br>◯ No, not measured
Did the study measure the number of previous term pregnancies?	◯ Yes, self reported<br>◯ Yes, medical/clinical chart<br>◯ No, not measured
Did the study measure the number of previous preterm pregnancies?	◯ Yes, self reported<br>◯ Yes, medical/clinical chart<br>◯ No, not measured
Did the study measure the number of previous induced or spontaneous abortions?	◯ Yes, self reported<br>◯ Yes, medical/clinical chart<br>◯ No, not measured
Did the study measure the number of previous stillbirths?	◯ Yes, self reported<br>◯ Yes, medical/clinical chart<br>◯ No, not measured
Did the study measure the number of previous pregnancies with preterm labor?	◯ Yes, self reported<br>◯ Yes, medical/clinical chart<br>◯ No, not measured
Did the study measure the number of previous pregnancies with eclampsia?	◯ Yes, self reported<br>◯ Yes, medical/clinical chart<br>◯ No, not measured
Did the study measure the number of previous pregnancies with preeclampsia?	◯ Yes, self reported<br>◯ Yes, medical/clinical chart<br>◯ No, not measured
Did the study measure the number of previous pregnancies with gestational diabetes?	◯ Yes, self reported<br>◯ Yes, medical/clinical chart<br>◯ No, not measured

For peer review only

Did the study measure the number of previous pregnancies with birth defects?	◯ Yes, self reported<br>◯ Yes, medical/clinical chart<br>◯ No, not measured
Did the study measure history of pregnancies with fetus or infant affected by genetic disorders and/or congenital abnormalities?	◯ Yes, self reported<br>◯ Yes, medical/clinical chart<br>◯ No, not measured

For peer review only

Current pregnancy

Did the study measure complications related to the current pregnancy	◯ Yes, self reported<br>◯ Yes, medical/clinical chart<br>◯ No, not measured
Did the study measure gestational diabetes?	◯ Yes<br>◯ No
Did the study measure maternal body mass index (BMI)?	◯ Yes<br>◯ No

For peer review only

Ascertainment of maternal exposure to other infectious diseases

How did the study determine maternal HIV status?<br><br>Select all that apply	☐ Self report<br>☐ Medical records<br>☐ Diagnostic test<br>☐ Other<br>☐ Not measured
Specify other means of determining maternal HIV status	__________________________________
When was maternal HIV status determined?<br><br>Select all that apply	☐ 1st term<br>☐ 2nd term<br>☐ 3rd term<br>☐ whenever symptoms were present (per clinical indication)<br>☐ other
At what other time was maternal HIV status determined?	__________________________________
What diagnostic tests were applied to assess maternal HIV infection status?<br><br>Select all that apply	☐ serology<br>☐ RT-PCR<br>☐ other NAAT<br>☐ rapid diagnostic test (RDT)<br>☐ other
Which other diagnostic tests were applied to assess maternal HIV infection status?	__________________________________
How did the study determine maternal malaria infection (fever, placental parasitemia)?<br><br>Select all that apply	☐ Self report<br>☐ Medical records<br>☐ Diagnostic test<br>☐ Other<br>☐ Not measured
Specify other means of determining malaria infection	__________________________________
When was maternal malaria status determined?<br><br>Select all that apply	☐ 1st term<br>☐ 2nd term<br>☐ 3rd term<br>☐ whenever symptoms were present (per clinical indication)<br>☐ other
At what other time was maternal malaria status determined?	__________________________________
How did the study determine maternal parvovirus infection?<br><br>Select all that apply	☐ Self report<br>☐ Medical records<br>☐ Diagnostic test<br>☐ Other<br>☐ Not measured
Specify other means of determining parvovirus infection	__________________________________

For peer review only

When was maternal parvovirus status determined?<br><br>Select all that apply	☐ 1st term<br>☐ 2nd term<br>☐ 3rd term<br>☐ whenever symptoms were present (per clinical indication)<br>☐ other
At what other time was maternal parvovirus status determined?	__________________________________
How did the study determine maternal listeria infection (fever, placental parasitemia)?<br><br>Select all that apply	☐ Self report<br>☐ Medical records<br>☐ Diagnostic test<br>☐ Other<br>☐ Not measured
Specify other means of determining listeria infection	__________________________________
When was maternal listeria status determined?<br><br>Select all that apply	☐ 1st term<br>☐ 2nd term<br>☐ 3rd term<br>☐ whenever symptoms were present (per clinical indication)<br>☐ other
At what other time was maternal listeria status determined?	__________________________________
Other than HIV and malaria, which STORCH pathogens were measured during pregnancy?<br><br>Select all that apply	☐ Syphilis<br>☐ Toxoplasmosis<br>☐ Other (varicella, parovirus B19)<br>☐ Rubella<br>☐ CMV<br>☐ Herpes
What other pathogens were measured during pregnancy?	__________________________________
How did the study determine maternal syphilis infection status?<br><br>Select all that apply	☐ Self report<br>☐ Medical records<br>☐ Diagnostic test<br>☐ Other
Specify other means of determining maternal syphilis infection status	__________________________________
Which diagnostic test(s) were applied to assess maternal syphilis infection status?<br><br>Select all that apply	☐ serology<br>☐ RT-PCR<br>☐ immunohistology<br>☐ darkfield microscopy<br>☐ other
What other diagnostic tests were applied to assess maternal syphilis infection status?	__________________________________

For peer review only

Which serologic assay(s) was/were applied to assess maternal syphilis infection status?<br><br>Select all that apply	☐ TPPA (Treponema pallidum particle agglutination assay)<br>☐ TPHA (Treponema pallidum hemagglutination assay)<br>☐ Microhemagglutination assay (MHA-TP)<br>☐ EIA IgG<br>☐ EIA IgM<br>☐ IgM Immunoblot<br>☐ FTA-ABS (Fluorescent treponemal antibody absorption test)<br>☐ 19S-IgM-FTA-ABS<br>☐ Venereal Disease Research Laboratory test (VDRL)<br>☐ Rapid-Plasma-Reagin (RPR)<br>☐ Other
Which other serologic assay(s) was/were applied to assess maternal syphilis infection status?	__________________________________
When during pregnancy did the study plan to diagnose syphilis infection?<br><br>Select all that apply	☐ 1st term<br>☐ 2nd term<br>☐ 3rd term<br>☐ whenever symptoms were present (per clinical indication)<br>☐ other
At what other time during pregnancy did the study plan to diagnose syphilis infection?	__________________________________
How did the study determine maternal toxoplasmosis infection status?<br><br>Select all that apply	☐ Self report<br>☐ Medical records<br>☐ Diagnostic test<br>☐ Other
Specify other means of determining maternal toxoplasmosis infection status	___________________________
Which diagnostic test(s) were applied to assess maternal toxoplasmosis infection status?<br><br>Select all that apply	☐ serology<br>☐ RT-PCR<br>☐ immunohistology<br>☐ darkfield microscopy<br>☐ other
What other diagnostic tests were applied to assess maternal toxoplasmosis infection status?	__________________________________
Which serologic assay(s) was/were applied to assess maternal toxoplasmosis infection status?<br><br>Select all that apply	☐ IgM<br>☐ IgG<br>☐ IgA<br>☐ Other
Which other serologic assay(s) was/were applied to assess maternal toxoplasmosis infection status?	__________________________________

For peer review only

When during pregnancy did the study plan to diagnose toxoplasmosis infection?<br><br>Select all that apply	☐ 1st term<br>☐ 2nd term<br>☐ 3rd term<br>☐ whenever symptoms were present (per clinical indication)<br>☐ other
At what other time during pregnancy did the study plan to diagnose toxoplasmosis infection?	__________________________________
How did the study determine maternal rubella infection status?<br><br>Select all that apply	☐ Self report<br>☐ Medical records<br>☐ Diagnostic test<br>☐ Other
Specify other means of determining maternal rubella infection status	____________________________
Which diagnostic test(s) were applied to assess rubella infection status?<br><br>Select all that apply	☐ serology<br>☐ RT-PCR<br>☐ immunohistology<br>☐ darkfield microscopy<br>☐ other
What other diagnostic tests were applied to assess maternal rubella infection status?	__________________________________
Which serologic assay(s) was/were applied to assess maternal rubella infection status?<br><br>Select all that apply	☐ TPPA (Treponema pallidum particle agglutination assay)<br>☐ TPHA (Treponema pallidum hemagglutination assay)<br>☐ Microhemagglutination assay (MHA-TP)<br>☐ EIA IgG<br>☐ EIA IgM<br>☐ IgM Immunoblot<br>☐ FTA-ABS (Fluorescent treponemal antibody absorption test)<br>☐ 19S-IgM-FTA-ABS<br>☐ Venereal Disease Research Laboratory test (VDRL)<br>☐ Rapid-Plasma-Reagin (RPR)<br>☐ Other
Which other serologic assay(s) was/were applied to assess maternal rubella infection status?	__________________________________
When during pregnancy did the study plan to diagnose rubella infection?<br><br>Select all that apply	☐ 1st term<br>☐ 2nd term<br>☐ 3rd term<br>☐ whenever symptoms were present (per clinical indication)<br>☐ other
At what other time during pregnancy did the study plan to diagnose maternal rubella infection?	__________________________________

For peer review only

How did the study determine maternal CMV infection status?<br><br>Select all that apply	☐ Self report<br>☐ Medical records<br>☐ Diagnostic test<br>☐ Other
Specify other means of determining maternal CMV infection status	__________________________________
Which diagnostic test(s) were applied to assess maternal CMV infection status?<br><br>Select all that apply	☐ serology<br>☐ RT-PCR<br>☐ immunohistology<br>☐ darkfield microscopy<br>☐ other
What other diagnostic tests were applied to assess maternal CMV infection status?	__________________________________
Which serologic assay(s) was/were applied to assess maternal CMV infection status?<br><br>Select all that apply	☐ TPPA (Treponema pallidum particle agglutination assay)<br>☐ TPHA (Treponema pallidum hemagglutination assay)<br>☐ Microhemagglutination assay (MHA-TP)<br>☐ EIA IgG<br>☐ EIA IgM<br>☐ IgM Immunoblot<br>☐ FTA-ABS (Fluorescent treponemal antibody absorption test)<br>☐ 19S-IgM-FTA-ABS<br>☐ Venereal Disease Research Laboratory test (VDRL)<br>☐ Rapid-Plasma-Reagin (RPR)<br>☐ Other
Which other serologic assay(s) was/were applied to assess maternal CMV infection status?	__________________________________
Did the study conduct avidity assays for IgG positive CMV?	◯ Yes<br>◯ No
When during pregnancy did the study plan to diagnose maternal CMV infection?<br><br>Select all that apply	☐ 1st term<br>☐ 2nd term<br>☐ 3rd term<br>☐ whenever symptoms were present (per clinical indication)<br>☐ other
At what other time during pregnancy did the study plan to diagnose CMV infection?	__________________________________
How did the study determine maternal herpes infection status?<br><br>Select all that apply	☐ Self report<br>☐ Medical records<br>☐ Diagnostic test<br>☐ Other
Specify other means of determining maternal herpes infection status	__________________________________

For peer review only

Which diagnostic test(s) were applied to assess maternal herpes infection status?<br><br>Select all that apply	☐ serology<br>☐ RT-PCR<br>☐ immunohistology<br>☐ darkfield microscopy<br>☐ other
What other diagnostic tests were applied to assess maternal herpes infection status?	__________________________________
Which serologic assay(s) was/were applied to assess maternal herpes infection status?<br><br>Select all that apply	☐ TPPA (Treponema pallidum particle agglutination assay)<br>☐ TPHA (Treponema pallidum hemagglutination assay)<br>☐ Microhemagglutination assay (MHA-TP)<br>☐ EIA IgG<br>☐ EIA IgM<br>☐ IgM Immunoblot<br>☐ FTA-ABS (Fluorescent treponemal antibody absorption test)<br>☐ 19S-IgM-FTA-ABS<br>☐ Venereal Disease Research Laboratory test (VDRL)<br>☐ Rapid-Plasma-Reagin (RPR)<br>☐ Other
Which other serologic assay(s) was/were applied to assess maternal herpes infection status?	__________________________________
When during pregnancy did the study plan to diagnose herpes infection?<br><br>Select all that apply	☐ 1st term<br>☐ 2nd term<br>☐ 3rd term<br>☐ whenever symptoms were present (per clinical indication)<br>☐ other
At what other time during pregnancy did the study plan to diagnose herpes infection?	__________________________________

For peer review only

Additional pregnancy-level maternal exposures

How did the study determine maternal nutrition status?<br><br>Select all that apply	☐ Self report<br>☐ Medical records<br>☐ Empirical evaluation of nutrition status<br>☐ Other<br>☐ Not measured
Specify other means of determining maternal nutrition status	__________________________
How did the study determine maternal smoking during pregnancy?<br><br>Select all that apply	☐ Self report<br>☐ Medical records<br>☐ Other<br>☐ Not measured
Specify other means of determining maternal smoking during pregnancy	__________________________
How did the study determine maternal alcohol consumption?<br><br>Select all that apply	☐ Self report<br>☐ Medical records<br>☐ Other<br>☐ Not measured
Specify other means of determining maternal alcohol consumption	__________________________
How did the study determine maternal recreational drug use?<br><br>Select all that apply	☐ Self report<br>☐ Medical records<br>☐ Other<br>☐ Not measured
Specify other means of determining maternal recreational drug use	__________________________
How did the study determine prescription drug or other medication use during pregnancy?<br><br>Select all that apply	☐ Self report<br>☐ Medical records<br>☐ Other<br>☐ Not measured
Specify other means of determining maternal prescription drug or medication use	__________________________
How were vaccinations during pregnancy determined?<br><br>Select all that apply	☐ Self report<br>☐ Medical records<br>☐ Other<br>☐ Not measured
Specify other means of determining vaccinations during pregnancy	__________________________
How did the study determine exposure to workplace or environmental teratogens (glycol ethers, solvents, heavy metals, pesticides) during pregnancy?<br><br>Select all that apply	☐ Self report<br>☐ Medical records<br>☐ Other<br>☐ Not measured

For peer review only

Specify other means of determining exposure to workplace or environmental teratogens	__________________________
How was exposure to intimate partner violence during pregnancy determined?<br><br>Select all that apply	☐ Self report<br>☐ Medical records<br>☐ Other<br>☐ Not measured
Specify other means of determining exposure to intimate partner violence during pregnancy	__________________________

For peer review only

Other infant/child-level covariates

How was infant/child exposure to family violence determined?<br><br>Select all that apply	☐ Self report<br>☐ Medical records<br>☐ Other<br>☐ Not measured
Specify other means of determining infant/child exposure to family violence	__________________________________
Did the study measure family structure after the birth of the index child?	◯ Yes<br>◯ No
Do infants or children in the study receive any developmental interventions (e.g. physical, speech, motor therapy) provided by the study or by the health system?	◯ Yes<br>◯ No<br>◯ Not measured
Please describe the physical therapy/motor therapy interventions received by infants or children in the study	____
Please describe the speech/language therapy interventions received by infants or children in the study	____
Please describe the cognitive therapy interventions received by infants or children in the study	____
Please describe any other types of therapies or developmental interventions received by infants or children in the study	____

For peer review only

Appendix 3

ZIKV-IPD-MA Consortium participants

Pablo Aguilar Ticona, MD
Institute of Collective Health
Federal University of Bahia
Salvador, Brazil

Luiz Carlos Junior Alcantara, PhD
Flavivirus Reference Laboratory (LABFLA) from Oswaldo Cruz Institution (IOC)
Oswaldo Cruz Foundation (FIOCRUZ)
Rio de Janeiro, Brazil

Jackeline Alger, MD, PhD
Facultad de Ciencias Médicas
Universidad Nacional Autónoma de Honduras
Tegucigalpa, Honduras

Celia Alpuche Aranda, MD, PhD
CISEI Centro de Investigación Sobre Enfermedades Infecciosas
Institution Instituto Nacional de Salud Publica de México
Cuernavaca, México

Jonathan Altamirano, MS
Department of Pediatrics
Division of Infectious Diseases
Stanford University School of Medicine
Stanford, California, USA

Juan Arias, MD, PhD
Infectious Diseases
St Jude's Children's Research Hospital
Memphis, Tennessee, USA

Lumumba Arriaga-Nieto, MD
Instituto Mexicano del Seguro Social
Mexico City, Mexico
Melissa A. G. Avelino, PhD
Department of Surgery of Goiás Federal University
Goiânia, Goiás, Brazil
Angel Balmaseda, MD
Department of Virology
Sustainable Sciences Institute
Managua, Nicaragua

Azucena Bardají, MD, PhD
ISGlobal
Hospital Clínic-Universitat de Barcelona
Barcelona, Spain

Consorcio de Investigación Biomédica en Red de Epidemiología y Salud Pública (CIBERESP)
Madrid, Spain

Centro de Investigação em Saúde de Manhiça (CISM)
Maputo, Mozambique

Carlos Hernan Becerra Mojica, MD
Departamento de Obstetricia y Ginecologia
Universidad Industrial de Santander

For peer review only

Bucaramanga, Colombia

Mónica Benavides, MPH
Dirección de Vigilancia y Análisis del Riesgo en Salud Publica
Grupo de enfermedades Inmunoprevenibles
Instituto Nacional de Salud
Bogotá, Colombia

A.P. Bertozzi, MD, PhD
Department of Pediatrics
Faculty of Medicine of Jundiaí
São Paulo, Brazil

Karen Blackmon, PhD
Department of Psychiatry and Psychology
Mayo Clinic
Jacksonville, FL, USA

Victor Hugo Borja Aburto, MD
Instituto Mexicano del Seguro Social
Mexico City, Mexico

Patrícia Brasil, MD, PhD
Laboratório de Pesquisa Clínica em Doenças Febris Agudas
Instituto Nacional de Infectologia Evandro Chagas - FIOCRUZ
Rio de Janeiro, Brazil

William J Britt, MD
Department of Pediatrics
Heersink School of Medicine
University of Alabama
Birmingham, AL

Nathalie Broutet, MD, PhD
UNDP/UNFPA/UNICEF/WHO/World Bank Special Programme of Research, Development and Research Training in Human Reproduction
Department of Sexual and Reproductive Health and Research
World Health Organization
Geneva, Switzerland

Pierre Buekens, MD, PhD
School of Public Health and Tropical Medicine
Tulane University
Louisiana, USA

André Cabié, MD, PhD
Infectious Diseases and Tropical Medicine
Université des Antilles, CHU de Martinique, INSERM CIC 1424, INSERM PCCEI UMR 1058
Fort-de-France, Martinique, France

David Alejandro Cabrera-Gaytán, MD
Instituto Mexicano del Seguro Social
Mexico City, Mexico

Rodrigo Cachay Figueroa, MD

For peer review only

Instituto de Medicina Tropical Alexander von Humboldt
Universidad Peruana Cayetano Heredia
Lima, Perú

María Luisa Cafferata, MD
Mother and Children Health Research Department
Institute for Clinical Effectiveness and Health Policy
Buenos Aires, Argentina

Juan Ignacio Calcagno, MD
Department of Hospital Epidemiology
Maternidade Professor José Maria de Magalhães Netto
Salvador, Bahia, Brazil

Juan P. Calle, MD
Department of Pediatrics, Universidad del Quindío
Centro de Estudios en Infectología Pediátrica
Cali, Colombia

Mabel Carabali, MD, PhD
Département de Médecine Sociale et Préventive, École de Santé Publique,
Université de Montréal,
Montreal, Quebec, Canada.

Department of Epidemiology, Dalla Lana School of Public Health,
University of Toronto.
Toronto, Ontario, Canada.

Derrick WS Chan, MD
Paediatric Neurology, Paediatric Medicine
KK Women's and Children's Hospital
Singapore

Celia CD Christie, MBBS, DM Peds
Department of Child and Adolescent Health
University of the West Indies, Mona Campus
Kingston, Jamaica

Federico Costa, PhD
Institute of Collective Health
Federal University of Bahia
Salvador, Brazil

Antonio Jose Cunha, PhD
Departamento de Pediatria
Federal University of Rio de Janeiro
Rio de Janeiro, Brazil

Carlos Cure Cure, MD
BIOMELAB SAS
Barranquilla, Colombia

Johanna Antonia Adriana Damen, PhD
Julius Center for Health Sciences and Primary Care
University Medical Center Utrecht
Utrecht University

For peer review only

Utrecht, Netherlands

Marcela C. Daza, Médica Pediatra, MSc
Grupo de Investigación en Salud Materna y Perinatal
Dirección de Investigación en Salud Pública
Instituto Nacional de Salud
Bogotá, Colombia

Roberta L. DeBiasi, MD
Departments of Pediatrics and Microbiology, Immunology and Tropical Medicine
The George Washington University School of Medicine
Children's National Hospital
Washington, DC, USA

Thomas Debray, PhD
Julius Center for Health Sciences and Primary Care
University Medical Center Utrecht
Utrecht University
Utrecht, Netherlands

Valentijn M.T. de Jong, PhD
Julius Center for Health Sciences and Primary Care
University Medical Center Utrecht
Utrecht University
Utrecht, Netherlands

Data Analytics and Methods Task Force
European Medicines Agency
Amsterdam, Netherlands
Camille Delgado López, MPH
Puerto Rico Department of Health
San Juan, Puerto Rico

Leah deWilde, BS
Epidemiology Division
Virgin Islands Department of Health
Christiansted, St. Croix, Virgin Islands, USA

Alan Oliveira Duarte, MSc
Instituto Gonçalo Moniz
Fundação Oswaldo Cruz
Salvador, Bahia, Brazil

Geraldo Duarte, MD, PhD
Ribeirão Preto Medical School
University of São Paulo
São Paulo, Brazil

Valorie Eckert, MPH
California Birth Defects Monitoring Program
Genetic Disease Screening Division
California Department of Public Health
Richmond, CA USA

Esther M. Ellis, PhD
Territorial Epidemiologist

For peer review only

Virgin Islands Department of Health
Christiansted, St. Croix, Virgin Islands, USA

Andres Espinosa-Bode, MD
Division of Global Health Protection, Central America Region Office
Centers for Disease Control and Prevention
Atlanta, GA, USA

Cassia Fernanda Estofolete, MD, PhD
Department of Dermatologic, Infectious and Parasititc Diseases
Faculdade de Medicina de São José do Rio Preto
São José do Rio Preto, Brazil

Roberta Evans, MS
Windward Islands and Research Education Foundation
St. George, Grenada

Valéria Christina de Rezende Féres, PhD
Laboratório BIOTEC
Faculdade de Farmácia - Universidade Federal de Goiás
Setor Leste Universitário, Goiânia, Brasil

Fabíola Fiaccadori, PhD
Virology and Cell Culture Laboratory
Institute of Tropical Pathology and Public Health
Federal University of Goiás
Goiânia, Goiás, Brazil

Lester Fernando Figueroa Bolaños, MD
Departamento de Planificacion
Instituto de Nutricion de Centroamerica y Panama (INCAP)
Guatemala City, Guatemala

Olivier Fléchelles, MD, PhD
Pediatric and Neonatal Intensive Care Unit
University Hospital of Martinique
Martinique, France

Arnaud Fontanet, MD, DrPH
Global Health
Institut Pasteur
Paris, France

Victoria Fumadó, MD, PhD
Infectious and Imported Diseases Unit
Department of Pediatrics, Sant Joan de Déu University
Hospital Research Foundation
Barcelona, Spain

Anna L. Funk, PhD
Department of Pediatrics, Cumming School of Medicine
University of Calgary
Calgary, Alberta, Canada

Anna Gajewski, MPH
Sustainable Sciences Institute

For peer review only

Managua, Nicaragua

Rosa Margarita Gelvez Ramirez, MSc
Centro de Atención y Diagnóstico de Enfermedades Infecciosas-CDI
Santander, Colombia

Carlo Giaquinto, MD
Dipartimento di Salute della Donna e del Bambino
Università degli Studi di Padova
Italy

Luz Gibbons, MSc
Institute for Clinical Effectiveness and Health Policy
Buenos Aires, Argentina

Suzanne M. Gilboa, PhD
National Center on Birth Defects and Developmental Disabilities
Centers for Disease Control and Prevention
Atlanta, GA, USA

Maria Barbara Franco Gomes, MD
Maternal Infant Hospital
Goiania, Brazil

Anna Goncé, MD, PhD
Barcelona Centre for Maternal-Fetal and Neonatal Medicine (Hospital Clínic and Hospital Sant Joan de Déu)

Institut d'Investigacions Biomèdiques August Pi i Sunyer (IDIBAPS)
Universitat de Barcelona

Centre for Biomedical Research on Rare Diseases (CIBER-ER)
Barcelona, Spain

Cesar R Gonzalez-Bonilla, MD, PhD
Coordinación de Investigación en Salud
Instituto Mexicano del Seguro Social
Mexico City, Mexico

Eduardo Gotuzzo, MD
Instituto de Medicina Tropical Alexander Von Humboldt
Universidad Peruana Cayetano Heredia
Lima, Peru

Concepción Grajales-Muñiz, MD
Instituto Mexicano del Seguro Social
Mexico City, Mexico

Rebecca Grant, MSc, MPH
Epidemiology of Emerging Diseases Unit
Institut Pasteur
Paris, France

Elysse Grossi-Soyster, PhD(c)
Sarnow and Schnieder Labs
Microbiology & Immunology Department
Stanford University School of Medicine

For peer review only

Tahani Hamdan, MPH
Houston Health Department
City of Houston
Houston, TX, USA

Eva Harris, PhD
Division of Infectious Diseases and Vaccinology, School of Public Health
University of California, Berkeley
Berkeley, CA, USA

Cosme Harrison, MPH
Division of Epidemiology
Virgin Islands Department of Health
Saint Thomas/Virgin Islands, USA

Bruno Hoen, MD, PhD
Infectious Diseases and Tropical Medicine
University hospital of Guadeloupe
Pointe-à-Pitre, Guadeloupe, France

Cristina Hofer, PhD
Instituto de Puericultura e Pediatria Martagão Gesteira
Universidade Federal do Rio de Janeiro
Rio de Janeiro, Brazil

Natanael Holband, MD
Academic Pediatric Center Suriname
Academic Hospital Paramaribo
Paramaribo, Suriname

Ivonne Huerta, MD
CISEI Centro de Investigación Sobre Enfermedades Infecciosas
Institution Instituto Nacional de Salud Publica de México
Cuernavaca, México

Irene Inwani, MD
Department of Paediatrics and Child Health
Kenyatta National Teaching and Referral Hospital
Nairobi, Kenya

Thomas Jaenisch, MD, PhD
Heidelberg Institute for Global Health
Heidelberg University Hospital
Heidelberg, Germany

Center for Global Health
Colorado School of Public Health
University of Colorado
Denver, CO, USA

Esaú João, PhD
Department of Infectious Diseases
Hospital Federal dos Servidores do Estado
Rio de Janeiro, Brazil

For peer review only

Edna Kara, MD
UNDP/UNFPA/UNICEF/WHO/World Bank Special Programme of Research, Development and Research Training in Human Reproduction
Department of Sexual and Reproductive Health and Research
World Health Organization
Geneva, Switzerland

Salma Khuwaja, MD, DrPH
Houston Health Department
City of Houston
Houston, TX, USA

Caron Kim, MD
UNDP/UNFPA/UNICEF/WHO/World Bank Special Programme of Research, Development and Research Training in Human Reproduction
Department of Sexual and Reproductive Health and Research
World Health Organization
Geneva, Switzerland

Albert I. Ko, MD
Department of Epidemiology of Microbial Diseases
Yale School of Public Health
New Haven, CT, USA

Nancy F. Krebs, MD
Department of Pediatrics, Section of Nutrition
University of Colorado School of Medicine
Aurora, CO, USA

Angelle Desiree LaBeaud, MD
Department of Pediatrics
Division of Infectious Diseases
Stanford University School of Medicine
Stanford, California, USA

Heather Lake-Burger, MS, MPH
Division of Community Health Promotion
Florida Department of Health
Tallahassee, FL, USA

Ellen H. Lee, MD
Bureau of Communicable Disease
New York City Department of Health and Mental Hygiene
New York, USA

Vernon Lee, PhD
Saw Swee Hock School of Public Health
National University of Singapore
Singapore

Yee-Sin Leo, MD
Executive Director's Office
National Centre for Infectious Diseases
Singapore

For peer review only

Brooke Levis, PhD
Centre for Clinical Epidemiology
Lady Davis Institute of Medical Research
Jewish General Hospital
Montreal, Quebec

Centre for Prognosis Research, School of Medicine,
Keele University,
Staffordshire, United Kindom

Eduardo Lopez-Medina, MD
Centro de Estudios en Infectología Pediátrica —CEIP—
Department of Pediatrics, Universidad del Valle
Clínica Imbanaco, Grupo Quironsalud
Cali, Valle, Colombia

Anyela Lozano-Parra, MSc
Facultad de Salud
Universidad Industrial de Santander
Santander, Colombia

Elena Marbán-Castro, PhD
ISGlobal
Hospital Clínic-Universitat de Barcelona
Barcelona, Spain

Celina Maria Turchi Martelli, PhD
The Research Centre Aggeu Magalhães
Fundação Oswaldo Cruz (Fiocruz)
Recife, Brazil

Lauren Maxwell, PhD
Heidelberg Institute for Global Health
Heidelberg University Hospital
Heidelberg, Germany

Department of Sexual and Reproductive Health and Research
Human Reproduction Programme
World Health Organization

Clara Menéndez, MD, PhD
ISGlobal
Hospital Clínic-Universitat de Barcelona
Barcelona, Spain

Consorcio de Investigación Biomédica en Red de Epidemiología y Salud Pública (CIBERESP)
Madrid, Spain

Centro de Investigação em Saúde de Manhiça (CISM)
Maputo, Mozambique

Marcela Mercado Reyes, MSc
Public Health Research Division
Instituto Nacional de Salud - Colombia
Bogotá, Colombia

For peer review only

Conrado Milani Coutinho, MD, PhD
Departmento de Ginecologia e Obstetrícia
Hospital das Clínicas da Faculdade de Medicina de Ribeirão Preto da Universidade de São Paulo
São Paulo, Brazil

María Consuelo Miranda Montoya, MD
Facultad de Salud Carrera
Universidad Industrial de Santander
Santander, Colombia

Demócrito de Barros Miranda-Filho, PhD
Faculty of Medical Sciences
Federal University of Pernambuco
Recife, Brazil

Maria Elisabeth Moreira, PhD
Department of Neonatology
Fundação Oswaldo Cruz (Fiocruz)
Rio de Janeiro, Brazil

J. Glenn Morris, Jr., MD
Emerging Pathogens Institute
University of Florida
Gainesville, FL, USA

Sarah B. Mulkey, MD, PhD
Departments of Neurology and Pediatrics
The George Washington University School of Medicine
Prenatal Pediatrics Institute
Children's National Hospital
Washington, DC, USA

Johanna Munoz, PhD
Julius Center for Health Sciences and Primary Care
University Medical Center Utrecht
Utrecht University
Utrecht, Netherlands

José Esteban Muñoz-Medina, PhD
Department of Epidemiology
Instituto Mexicano del Seguro Social
Mexico City, Mexico

Peninah Munyua, PhD
Division of Global Health Protection
US Centers for Disease Control and Prevention
Nairobi, Kenya

Marisa Marcia Mussi-Pinhata, MD, PhD
Departamento de Pediatria
Faculdade de Medicina de Ribeirão Preto da Universidade de São Paulo
São Paulo, Brazil

Nivison Nery Jr., MSc
Instituto de Saúde Coletiva

For peer review only

Federal University of Bahia
Salvador, Brazil

M. Kariuki Njenga, PhD
Paul Allen Center for Global Health
Washington State University
Nairobi, Kenya

Mauricio L. Nogueira, MD, PhD
Department of Dermatologic, Infectious and Parasititc Diseases
Faculdade de Medicina de São José do Rio Preto
São José do Rio Preto, Brazil

Eric Osoro, MD
Department of Global Health
Washington State University, Kenya
Nairobi, Kenya

Miguel Parra-Saavedra, PhD
Department of Gynecology and Obstetrics
Universidad Simon Bolivar, CEDIUL
Barranquilla, Colombia

Saulo Passos, PhD
Department of Pediatrics
Faculty of Medicine of Jundiaí
São Paulo, Brazil

Bernadete Perez Coêlho MD, PhD
Department of Public Health of the Medical Sciences Center
Federal University of Pernambuco
Recife, Pernambuco, Brasil

Monika Piccardi RN, BSN, MS
Office for and People with Special Healthcare Needs
Maryland Department of Health
Baltimore, MD, USA

Léo Pomar, PhD
Department of Obstetrics and Gynecology
Centre Hospitalier de l'Ouest Guyanais
Saint-Laurent du Maroni, French Guiana

Materno-fetal and Obstetrics Research Unit
Department Woman-mother-child
Lausanne University Hospital, Switzerland

School of Health Sciences (HESAV)
University of Applied Sciences and Arts Western Switzerland
Delémont, Switzerland

Arnaldo Prata-Barbosa, PhD
Department of Pediatrics
D'Or Institute for Research & Education
Rio de Janeiro, Brazil

For peer review only

Ingrid Rabe, MBChB, MMed
Emerging Diseases and Zoonoses Unit
Global Infectious Hazards
Health Emergencies Programme
World Health Organization
Geneva, Switzerland

Mitermayer Reis, MD, PhD
Laboratório de Patologia e Biologia Molecular
Fundação Oswaldo Cruz (Fiocruz)
Salvador, Brazil

Hannah Rettler, MPH
Bureau of Epidemiology
Utah Department of Health
Salt Lake City, USA

Megan R. Reynolds, MPH
National Center on Birth Defects and Developmental Disabilities
Centers for Disease Control and Prevention
Atlanta, GA, USA

Ana Maria Rivera Casas, MD
Maternal-Fetal Medicine
FUCS University
Bogota, Colombia

Diana Patricia Rojas, MD, PhD
WHE/GIH/EZD
Zika and Chikungunya
World Health Organization
Geneva, Switzerland

Teresita Rojas-Mendoza, MD
Instituto Mexicano del Seguro Social
Mexico City, Mexico

Nicole Roth, MPH
National Center on Birth Defects and Developmental Disabilities
Centers for Disease Control and Prevention
Atlanta, GA, USA

Paola Mariela Saba Villarroel, PhD
Unité des Virus Émergents
UVE: Aix-Marseille University-IRD 190-INSERM 1207-IHU Méditerranée Infection
Marseille, France

Magda Sanz Cortes, MD, PhD
Department of Obstetrics and Gynecology
Fetal Surgery and Intervention
Baylor College of Medicine
Houston, TX, USA

Janet L. Sayers, MS
UNDP/UNFPA/UNICEF/WHO/World Bank Special Programme of Research, Development and Research Training in Human Reproduction

For peer review only

Department of Sexual and Reproductive Health and Research
Human Reproduction Programme
World Health Organization
Geneva, Switzerland

Deolinda Scalabrin, MD, PhD
Instituto Gonçalo Moniz
Fundação Oswaldo Cruz (Fiocruz)
Salvador, Bahia, Brasil

Lavinia Schuler-Faccini, MD, PhD
Genetics Department
Hospital de Clinicas de Porto Alegre
Universidade Federal do Rio Grande do Sul
Porto Alegre, Rio Grande do Sul, Brazil

Stacey Schultz-Cherry, PhD
Department of Infectious Diseases
St Jude Graduate School
St. Jude Children's Research Hospital
Memphis, Tennessee, USA

Kirstin Short, MPH
Houston Health Department
City of Houston
Houston, Texas, USA

Priya Shreedhar, MSc
Heidelberg Institute of Global Health
Heidelberg University
Heidelberg, Germany

Antônio Augusto Silva, PhD
Department Public Health
Federal University of Maranhão
Sao Luis, Maranhão, Brazil

Ronaldo Silva, PhD
UNDP/UNFPA/UNICEF/WHO/World Bank Special Programme of Research, Development and Research Training in Human Reproduction
Department of Sexual and Reproductive Health and Research
Human Reproduction Programme

Isadora Siqueira, MD, PhD
Researcher in Public Health
Instituto Gonçalo Moniz-Fiocruz
Salvador-BA, Brasil

Karen Sohan, MD
Consultant Obstetrician & Gynaecologist
Diagnostic (Mother & Baby) Ltd
Trinidad & Tobago

Carmen Soria-Segarra, MD PhD
SOSECALI C. Ltda
Universidad Católica Santiago de Guayaquil

For peer review only

Guayaquil, Ecuador

Antoni Soriano-Arandes, MD, PhD
Paediatric Infectious Diseases and Immunodeficiencies Unit
Hospital Universitari Vall d'Hebron
Barcelona, Catalonia, Spain

Patrícia da Silva Sousa, MD, PhD
Child Neurology and Clinical Neurophysiology
Universidade Federal do Maranhão - UFMA
Sao Luis, Maranhão, Brazil

Maria Benamor Teixeira, MD, PhD
Department of Infectious Diseases
Hospital Federal dos Servidores do Estado
Rio de Janeiro, Brazil

Claire Thorne, PhD
Great Ormond Street Institute of Child Health
University College London
London, UK

Soe Soe Thwin, PhD
UNDP/UNFPA/UNICEF/WHO/World Bank Special Programme of Research, Development and Research Training in Human Reproduction
Department of Sexual and Reproductive Health and Research
Human Reproduction Programme
World Health Organization
Geneva, Switzerland

Van T. Tong, MPH
National Center on Birth Defects and Developmental Disabilities
Centers for Disease Control and Prevention
Atlanta, GA, USA

Benoit Tressieres, MSc
Centre d'Investigation Clinique Antilles Guyane, Inserm CIC 1424
CHU de la Guadeloupe
Pointe-à-Pitre, France

Marília Dalva Turchi, PhD
Institute of Tropical Pathology and Public Health
Federal University of Goias
Goiânia, Brazil

Diana Valencia, MS
National Center on Birth Defects and Developmental Disabilities
Centers for Disease Control and Prevention
Atlanta, GA, USA

Miguel Valencia-Prado, MD
Puerto Rico Department of Health
San Juan, Puerto Rico

Alfonso Vallejos-Parás, PhD
Instituto Mexicano del Seguro Social

For peer review only

Mexico City, Mexico

Maria Van Kerkhove, PhD
WHO/WHE/GIH Unit head Emerging and Zoonotic Diseases
World Health Organization
Geneva, Switzerland

Luis Angel Villar, MD, PhD
Facultad de Salud Carrera
Universidad Industrial de Santander
Santander, Colombia

Carmen Viñuela Benéitez, MD
Department of Obstetrics and Gynecology.
Gregorio Marañón University Hospital School of Medicine
Complutense University.
Madrid, Spain

Manon Vouga, MD, PhD
Department Woman-Mother-Child
Lausanne University Hospital,
Lausanne, Switzerland

Randall Waechter, PhD
Department of Neuroscience, Physiology, & Behavioral Science
School of Medicine, St. George's University
St. George, Grenada

Yinghui Wei, PhD
Centre for Mathematical Sciences
School of Engineering, Computing and Mathematics
University of Plymouth
Plymouth, UK

Jamie Westcott, MS
Section of Pediatric Nutrition
University of Colorado School of Medicine
Aurora, CO, USA

Marc-Alain Widdowson, VetMB, MA, MSc
Division of Global Health Protection
Centers for Disease Control and Prevention
Nairobi, Kenya

George SH Yeo, MD
Division of Obstetrics and Gynecology
KK Women's and Children's Hospital
Singapore

Ricardo Arraes de Alencar Ximenes, MD, PhD
Department of Tropical Medicine
Federal University of Pernambuco
Recife, Brazil

For peer review only

For peer review only

BMJ Open

Heterogeneity of Zika Virus Exposure and Outcome Ascertainment Across Cohorts of Pregnant Women, their Infants, and their Children: A Metadata Survey

Journal:	*BMJ Open*
Manuscript ID	bmjopen-2022-064362.R1
Article Type:	Original research
Date Submitted by the Author:	24-Oct-2022
Complete List of Authors:	Carabali, Mabel; Universite de Montreal, Médecine Sociale et Préventive ; University of Toronto Dalla Lana School of Public Health, Epidemiology<br>Maxwell, Lauren; World Health Organization, Department of Sexual and Reproductive Health and Research; UniversitätsKlinikum Heidelberg<br>Levis, Brooke; Keele University, Centre for Prognosis Research, School of Primary, Community and Social Care<br>Shreedhar, Priya; UniversitätsKlinikum Heidelberg
**Primary Subject Heading**:	Infectious diseases
Secondary Subject Heading:	Epidemiology, Global health, Public health
Keywords:	Epidemiology < TROPICAL MEDICINE, Public health < INFECTIOUS DISEASES, INFECTIOUS DISEASES

SCHOLARONE™
Manuscripts

I, the Submitting Author has the right to grant and does grant on behalf of all authors of the Work (as defined in the below author licence), an exclusive licence and/or a non-exclusive licence for contributions from authors who are: i) UK Crown employees; ii) where BMJ has agreed a CC-BY licence shall apply, and/or iii) in accordance with the terms applicable for US Federal Government officers or employees acting as part of their official duties; on a worldwide, perpetual, irrevocable, royalty-free basis to BMJ Publishing Group Ltd ("BMJ") its licensees and where the relevant Journal is co-owned by BMJ to the co-owners of the Journal, to publish the Work in this journal and any other BMJ products and to exploit all rights, as set out in our licence.

The Submitting Author accepts and understands that any supply made under these terms is made by BMJ to the Submitting Author unless you are acting as an employee on behalf of your employer or a postgraduate student of an affiliated institution which is paying any applicable article publishing charge ("APC") for Open Access articles. Where the Submitting Author wishes to make the Work available on an Open Access basis (and intends to pay the relevant APC), the terms of reuse of such Open Access shall be governed by a Creative Commons licence – details of these licences and which Creative Commons licence will apply to this Work are set out in our licence referred to above.

Other than as permitted in any relevant BMJ Author's Self Archiving Policies, I confirm this Work has not been accepted for publication elsewhere, is not being considered for publication elsewhere and does not duplicate material already published. I confirm all authors consent to publication of this Work and authorise the granting of this licence.

For peer review only

Heterogeneity of Zika Virus Exposure and Outcome Ascertainment Across Cohorts of Pregnant Women, their Infants, and their Children: A Metadata Survey

Authors: Mabel Carabali[1, 2], Lauren Maxwell[3, 4], Brooke Levis[5, 6], Priya Shreedhar[4], on behalf of the ZIKV IPD consortium members.

Affiliations

1. Département de Médecine Sociale et Préventive, École de Santé Publique, Université de Montréal, Montreal, Quebec, Canada.
2. Department of Epidemiology, Dalla Lana School of Public Health, University of Toronto. Toronto, Ontario, Canada.
3. Development and Research Training in Human Reproduction, Department of Sexual and Reproductive Health and Research, World Health Organization, Geneve, Switzerland.
4. Heidelberger Institut für Global Health, Universitätsklinikum Heidelberg, Heidelberg, Germany.
5. Centre for Clinical Epidemiology, Lady Davis Institute for Medical Research, Montreal, Quebec, Canada.
6. Centre for Prognosis Research, School of Medicine, Keele University, Staffordshire, United Kingdom.

Corresponding author:

Dr. Mabel Carabali: mabel.carabali@utoronto.ca

Département de Médecine Sociale et Préventive, École de Santé Publique, Université de Montréal

7101 Avenue du Parc, Bureau/Office: 3123, Montréal (QC), H3N 1X9

Tel: 514 343 6111 Ext 44021

For peer review only - http://bmjopen.bmj.com/site/about/guidelines.xhtml

The ZIKV-IPD-MA Consortium: Pablo Aguilar Ticona, Luiz Carlos Junior Alcantara, Jackeline Alger, Celia Alpuche Aranda, Jonathan Altamirano, Juan Arias, Lumumba Arriaga-Nieto, Melissa A. G. Avelino, Angel Balmaseda, Azucena Bardají, Carlos Hernan Becerra Mojica, Mónica Benavides, A.P. Bertozzi, Karen Blackmon, Victor Hugo Borja Aburto, *Patrícia Brasil, William J Britt,* Nathalie Broutet, Pierre Buekens, André Cabié, David Alejandro Cabrera-Gaytán, Rodrigo Cachay Figueroa, María Luisa Cafferata, Juan Ignacio Calcagno, Juan P. Calle, Mabel Carabali, Derrick WS Chan, Celia CD Christie, Federico Costa, Antonio Jose Cunha, Carlos Cure, Johanna Antonia Adriana Damen, Marcela C. Daza, Roberta L. DeBiasi, Thomas Debray, Valentijn M.T. de Jong, Camille Delgado López, Leah deWilde, Alan Oliveira Duarte, Geraldo Duarte, Valorie Eckert, Esther M. Ellis, Andres Espinosa-Bode, Cassia Fernanda Estofolete, Roberta Evans, Valéria Christina de Rezende Féres, Fabíola Fiaccadori, Lester Fernando Figueroa Bolaños, Olivier Fléchelles, Arnaud Fontanet, Victoria Fumadó, Anna L. Funk, Anna Gajewski, Rosa Margarita Gelvez Ramirez, Carlo Giaquinto, Luz Gibbons, Suzanne M. Gilboa, Maria Barbara Franco Gomes, Anna Goncé, Cesar R Gonzalez-Bonilla, Eduardo Gotuzzo, Concepción Grajales-Muñiz, Rebecca Grant, Elysse Grossi-Soyster, Tahani Hamdan, Eva Harris, Cosme Harrison, Bruno Hoen, Cristina Hofer, Natanael Holband, Ivonne Huerta, Irene Inwani, Thomas Jaenisch, Esaú João, Edna Kara, Salma Khuwaja, Caron Kim, Albert I. Ko, Nancy F. Krebs, Angelle Desiree LaBeaud, Heather Lake-Burger, Ellen H. Lee, Vernon Lee, Yee-Sin Leo, Brooke Levis, Eduardo Lopez-Medina, Anyela Lozano-Parra, Elena Marbán-Castro, Celina Maria Turchi Martelli, Lauren Maxwell, Clara Menéndez, Marcela Mercado Reyes, Conrado Milani Coutinho, María Consuelo Miranda Montoya, Demócrito de Barros Miranda-Filho, Maria Elisabeth Moreira, J. Glenn Morris, Jr., Sarah B. Mulkey, Johanna Munoz, José Esteban Muñoz-Medina, Peninah Munyua, Marisa Marcia Mussi-Pinhata, Nivison Nery Jr., M. Kariuki Njenga, Mauricio L. Nogueira, Eric Osoro, Miguel Parra-Saavedra, Saulo Passos, Bernadete Perez Coêlho, Monika Piccardi, Léo Pomar, Arnaldo Prata-Barbosa, Ingrid Rabe, Mitermayer Reis, Hannah Rettler, Megan R. Reynolds, Ana Maria Rivera Casas, Diana Patricia Rojas, Teresita Rojas-Mendoza, Nicole Roth, Paola Mariela Saba Villarroel, Magda Sanz Cortes, Janet L. Sayers, Deolinda Scalabrin, Lavinia Schuler-Faccini, Stacey Schultz-Cherry, Kirstin Short, Priya Shreedhar, Antônio Augusto Silva, Ronaldo Silva, Isadora Siqueira, Karen Sohan, Carmen Soria-Segarra, Antoni Soriano-Arandes, Patrícia da Silva Sousa, Maria Benamor Teixeira, Claire Thorne, Soe Soe Thwin, Van T. Tong, Benoit Tressieres, Marília Dalva Turchi, Diana Valencia, Miguel Valencia-Prado, Alfonso Vallejos-Parás, Maria Van Kerkhove, Luis Angel Villar, Carmen Viñuela Benéitez, Manon Vouga, Randall Waechter, Yinghui Wei, Jamie Westcott, Marc-Alain Widdowson, George SH Yeo, Ricardo Arraes de Alencar Ximenes.
The full list of participants of the ZIKV-IPD-MA Consortium members is attached as supplementary material.

For peer review only - http://bmjopen.bmj.com/site/about/guidelines.xhtml

ABSTRACT (248 words)

Objectives: To support the Zika virus (ZIKV) Individual Participant Data (IPD) Consortium's efforts to harmonize and analyze IPD from ZIK-related prospective cohort studies and surveillance-based studies of pregnant women and their infants and children; we developed and disseminated a metadata survey among ZIKV-IPD meta-analysis (MA) study participants to identify and provide a comprehensive overview of study-level heterogeneity in exposure, outcome, and covariate ascertainment and definitions.

Setting: Cohort and surveillance studies that measured ZIKV infection during pregnancy or at birth and measured fetal, infant, or child outcomes were identified through a systematic search and consultations with ZIKV researchers and Ministries of Health from 20 countries or territories.

Participants: Fifty-four cohort or active surveillance studies shared de-identified data for the IPD-MA and completed the metadata survey, representing 33,061 women (11,020 with ZIKV) and 18,281 children.

Primary and secondary outcome measures: Study-level heterogeneity in exposure, outcome, and covariate ascertainment and definitions.

Results: Median study sample size was 268 (Interquartile range (IQR)=100, 698). Inclusion criteria, follow-up procedures, and exposure and outcome ascertainment were highly heterogenous, differing meaningfully across regions and multisite studies. Enrollment duration and follow-up for children after birth varied before and after the declaration of the Public Health Emergency of International Concern (PHEIC) and according to the type of funding received.

Conclusion: This work highlights the logistic and statistical challenges that must be addressed to account for the multiple sources of within- and between-study heterogeneity when conducting IPD-MAs of data collected in the research response to emergent pathogens like ZIKV.

For peer review only - http://bmjopen.bmj.com/site/about/guidelines.xhtml

For peer review only

Strengths and limitations

- We document the presence of cross- and within study heterogeneity across 54 different ZIKV-IPD-MA participant studies from 20 countries or territories. At the time of submission, our study included one of the largest samples of women (n=33,061) and ZIKV confirmed cases (n=11,020).
- Study and laboratory protocols changed substantially over time, as knowledge about ZIKV evolved, resulting in important heterogeneity across studies. The in-depth evaluation of the cross-study heterogeneity in exposure, outcome, and covariate ascertainment and definitions, elucidate logistic and methodological challenges inherent to conduct an IPD-MA of emergent pathogens.
- The level of heterogeneity across studies underlines the urgent need to develop statistical methods that account properly for the measurement error in both exposure and outcome ascertainment in the context of an IPD-MA of an emergent pathogen.
- This assessment could be used to appropriately separate measurement- versus clinically relevant sources of heterogeneity in the ZIKV IPD-MA.
- This appraisal can be used as a template for the transparent and comprehensive treatment of different sources of bias when harmonizing and analyzing IPD collected in the research response to other emerging pathogens.

For peer review only

INTRODUCTION

In 2016, the sharp surge in microcephaly incidence led to the declaration of Zika virus (ZIKV) as a public health emergency of international concern (PHEIC). Five years later, our understanding of the short and long-term effects of ZIKV exposure *in utero* is still incomplete[1-4]. ZIKV is principally transmitted by *Aedes* mosquitoes and can also be transmitted through unprotected sexual intercourse with a ZIKV-infected partner[5,6]. To date, 90 countries have reported autochthonous ZIKV transmission[7].

Diagnosis of ZIKV infection is challenging, expensive, and time consuming, especially in endemic areas where cross-reactivity and co-circulating arboviruses are important concerns[8-14]. Most ZIKV infections (75-85%) are asymptomatic.[1,5-7] If symptoms arise, the presence of conjunctivitis and rash distinguish ZIKV from other self-limited febrile arboviral infections (e.g., dengue virus (DENV), chikungunya (CHIKV))[6,15,16]. The confirmation of ZIKV includes ruling out the presence of DENV and other arboviruses via real-time or standard Reverse Transcription Polymerase Chain Reaction (RT-PCR) or plaque reduction neutralization test (PRNT)[9]. The performance of both immunological and molecular ZIKV tests differs according to the sensitivity and specificity of the assay, the characteristics of the sample (e.g., sample type, sample maintenance), the timing between symptom onset and sample collection and between sample collection and testing, and the implementation and adherence to laboratory quality control (QC) procedures[8-10,17].

ZIKV infections during pregnancy have been associated with severe outcomes including fetal loss and congenital Zika syndrome (CZS), a spectrum of clinical conditions including central nervous system-related congenital malformations (e.g., microcephaly) as well as ophthalmologic and musculoskeletal abnormalities[1,3]. American and Asian ZIKV strains have been causally related to fetal and postnatal onset microcephaly and CZS; recent nonhuman experimental studies describe higher than expected fetal pathogenicity among recent ZIKV African strains[18]. There is no specific treatment for ZIKV other than management of symptoms and no ZIKV vaccine has yet been approved for use[6,7,19,20]. At the country level, ZIKV-related healthcare needs and employment loss cost an estimated USD 2.3 billion per year and caused average yearly loss of over 44,000 disability-adjusted life years (DALYs) [21-24].

The ZIKV Individual Participant Data (IPD) Consortium was created in 2017 to bring together ZIKV-focused clinical and social science researchers and other stakeholders to conduct an IPD meta-analysis (IPD-MA)[2,25] of ZIKV-related cohorts and surveillance systems that measured ZIKV exposure during pregnancy or at birth. By harmonizing existing IPD across related studies, the consortium will leverage

For peer review only - http://bmjopen.bmj.com/site/about/guidelines.xhtml

existing data to develop and evaluate prognostic models to inform health care providers, pregnant women, and couples planning a pregnancy, and to improve prevention and control measures through identifying groups at the highest risk of adverse consequences during future outbreaks. In addition to priorities for improved diagnosis, management, and prevention of ZIKV infection described in the ZIKV Research and Development Roadmap[26], the ZIKV IPD Consortium's research priorities are to: 1) identify modifiers of the relation between ZIKV infection during pregnancy and adverse short and long term fetal, infant, and child outcomes, 2) assess the role of measurement error and other sources of bias in study estimates, and 3) assess the long-term effects of congenital exposure for children with and without clinical or laboratory signs of congenital infection at birth, with consistent definitions of CZS[2]. The ZIKV-IPD Consortium IPD-MA currently includes 65 cohorts and local surveillance sites from 24 countries and territories in the Americas, Asia, Europe, and Africa[2].

Several logistic and analytic challenges complicate the pooled analysis of retrospectively harmonized IPD from longitudinal studies in the ZIKV IPD-MA. Cross- and within-study assessment of the risk of adverse fetal, infant, and child ZIKV-related outcomes has been limited by: heterogeneity in study design, selection bias, differential ascertainment of important confounders or mediators, limitations in the ascertainment of exposure (e.g., timely diagnosis or incomplete follow-up) and outcomes (e.g., pregnancy losses or terminations), and lack of standardization in definitions across studies, which were primarily launched mostly during the acute phase of the ZIKV outbreak in 2015-2016[1-3,7,11]. Therefore, well characterized metadata are essential to appropriately account for study-level sources of heterogeneity in outcome or imputation models and to further improve the precision of subject-level inference in IPD-MA studies.

To support the ZIKV IPD Consortium's efforts to harmonize and analyze IPD from ZIKV- related prospective cohort studies and surveillance-based studies of pregnant women and their infants and children, we developed and disseminated a survey of study-level characteristics (i.e., metadata). Here we present the results from the ZIKV-IPD metadata survey to provide a comprehensive overview of study-level sources of heterogeneity related to IPD datasets that have or will be made available for the IPD-MA. The in-depth evaluation of cross-study heterogeneity in exposure, outcome, and covariate ascertainment and definitions will be used to inform and develop the analytic methods required to appropriately separate measurement- versus clinically derived sources of heterogeneity in the ZIKV IPD-MA. This comprehensive metadata review described the different sources of bias and heterogeneity in the analysis of ZIKV-related studies. It serves as a template for future work to harmonize and analyze IPD collected in the research response to emerging pathogens, where changes

For peer review only - http://bmjopen.bmj.com/site/about/guidelines.xhtml

to best practice, definitions, and study and laboratory protocols proscribe the use of traditional metadata extraction.

METHODS

Study selection

Studies eligible to participate in the IPD-MA and corresponding metadata survey were identified through a systematic search registered on PROSPERO (CRD42017068915) that was subsequently published elsewhere[2,25]. The systematic search, developed in consultation with an information scientist and initially conducted on July 8, 2018, applied MeSH and text terms to the Ovid (Medline and Embase) without date, geography, or language-related restrictions. Additional studies were identified through consultation with subject matter experts, Ministries of Health (MoH) and regional health authorities (WHO, PAHO), and through a monthly PubMed alert that applied the following text terms: Zika[Title] AND protocol[Title/Abstract] and Zika[Title] AND cohort[Title]. Longitudinal observational studies or local surveillance systems that (1) conducted laboratory testing for ZIKV during pregnancy or at birth; (2) measured fetal, infant, and/or child outcomes; and (3) included at least ten individuals (women or children) were eligible. Details of the systematic search to identify eligible studies, including the search strategy, PICO criteria, eligibility, and the screening and selection steps are presented in Appendix 1, Figure S1. Eligible studies were invited via e-mail to share data and to actively participate in the development and completion of the IPD-MA.

Metadata survey development and dissemination

To obtain key metadata information, specifically about procedures that could have potentially changed after studies' implementation, and to obtain information directly from investigators instead of relying on information included in published studies or protocols, we developed a comprehensive metadata survey of completed and ongoing ZIKV-related studies available in English, Spanish and Portuguese (Appendix 2). We programmed the survey using Research Electronic Data Capture (REDCap)[27] software and disseminated the survey to sites that agreed to share participant-level data for the IPD-MA. The metadata survey included 964 items related to study location, design, population, enrollment criteria, and sample size; maternal, placental, fetal, and infant ascertainment of ZIKV; ascertainment of microcephaly and CZS (clinical and laboratory criteria), other arboviruses, and STORCH pathogens; genetic testing, child development assessment, funding, and duration and timing of follow-up contacts. ZIKV IPD Consortium members formed expert panels that were designated as subject matter working groups (WGs) for outcomes, exposures, social sciences, metadata and harmonization. Each WG included clinicians with expertise in relevant areas (e.g., neonatology, neurology, psychology,

For peer review only - http://bmjopen.bmj.com/site/about/guidelines.xhtml

virology), epidemiologists, sociologists, and members of the metadata and harmonization team (i.e., consortium members who will work on the harmonization of the data as it becomes available). Using the ZIKV IPD-MA protocol as a guide, the WGs met and reviewed the established exposure and outcome-related components of the metadata survey to ensure its comprehensiveness, this is, that the draft survey included all potential exposures, outcomes, and likely confounders, and the methods for their ascertainment or measurement. The WGs also assessed the utility of survey in terms of public health and epidemiological relevance to potential end users such as other researchers, policy makers, and health care practitioners. The metadata survey was first circulated to ZIKV IPD Consortium members in June of 2020 and closed to new responses in March 2021. Investigators received two monthly reminders to complete the survey and were contacted individually to resolve study-level missingness and to address inconsistencies between survey responses and published study reports.

Data analysis

Overall design, recruitment, exposure, outcome, and covariate ascertainment variables included in the resultant metadata dataset were described using median and interquartile range (IQR) for continuous variables and proportions for categorical variables using RStudio Team (2020; R version 4·0·5). The metadata were assessed for cross- and within-study heterogeneity in recruitment, exposure, outcome and covariate ascertainment and definitions. ZIKV ascertainment practices were assessed by the type (e.g., serological, or molecular testing), timing of the ascertainment (e.g., antenatal, perinatal, and postnatal), and type or source of guidelines used. We conducted a *post hoc* qualitative bias assessment that describes potential issues with design and exposure and outcome ascertainment.

Patient and Public Involvement statement

This study assesses already collected data from ongoing or already finished ZIKV- research studies conducted during the ZIKV epidemic and PHEIC. Our unit of analysis are studies, not individuals. For this study, we did not involve patients in the development of research questions or outcome measurement. However, the ZIKV-IPD-MA consortium aims to leverage existing data to develop and evaluate prognostic models to inform pregnant women and couples planning a pregnancy, and to improve prevention and control measures through identifying groups at the highest risk of adverse consequences during future outbreaks. The consortium is in constant communication with Principal Investigators (PIs), Co-PIs, and site coordinators of the participant studies. Each participant study has a study-specific plan to disseminate their results. IPD-MA research results are disseminated among PIs, Co-PIs, and site coordinators as part of the already established different monthly WG meetings and yearly consortium meetings.

For peer review only - http://bmjopen.bmj.com/site/about/guidelines.xhtml

Role of the funding source

The systematic review and metadata survey were drafted and revised with funding support provided by the Wellcome Trust to the WHO Department of Sexual and Reproductive Health and Research Human Reproduction Program, grant numbers 206532/Z/17/Z and 216002/Z/19/Z, respectively. The funder of the study had no role in study design, data collection, data analysis, data interpretation, or writing of the report.

RESULTS

From 160 eligible publications identified through the systematic search and 145 studies identified through consultation, 63 cohort study or surveillance sites, representing 59 unique datasets agreed to share de-identified, participant-level data for the ZIKV IPD-MA (Figure 1A). The metadata survey was completed for 54 of these datasets (91·5%). Remaining studies (n=5) did not initiate or complete the metadata survey after nine months of regular follow-up. The four teams that reported a reason for non-participation, reported multiple considerations including inadequate time or resources to support participation (n=4), lack of consensus on participation from their team (n=1) and need for special permissions from MoH (n=1). The difference between the number of study sites and unique datasets is explained by the inclusion of studies using data from a single surveillance system or multisite studies for which the metadata (e.g., source population, ZIKV assays, and outcome ascertainment) did not vary across sites. For instance, the NIH/NIAID-funded International Prospective Observational Cohort Study of Zika in Infants and Pregnancy (ZIP) study[28] and the European Commission Horizon 2020-funded ZIKAlliance study[29,30] each standardized outcomes definitions and laboratory procedures across sites, but due to specific differences in the source population or follow-up procedures these studies are analyzed individually by study site here. In contrast, the United States Zika Pregnancy and Infant Registry (USZPR)[31,32] is an enhanced surveillance program including 50 states and other US territories with a shared standardized protocol contributing to a single metadata dataset. The Puerto Rico Zika Active Pregnancy Surveillance System (PR-ZAPSS) study[33], part of the overall US Zika registry program, has a slightly different protocol and is included as a separate dataset.

Recruitment

Table 1 presents basic characteristics of participating studies by geography. The 54 studies that completed the metadata survey included 33,061 women (non-pregnant, pregnant, and puerperal), 11,030 confirmed maternal ZIKV cases, and 18,281 infants and children from 20 countries or territories. Details including study location, authors, source population, enrollment characteristics, study design, recruitment and follow-up visits, sample size, and funding are presented in Appendix 1,

For peer review only - http://bmjopen.bmj.com/site/about/guidelines.xhtml

Table S1. Twenty-three studies (43%) were conducted in Brazil (Figure 1B). Thirty-seven studies (68%) recruited participants from healthcare facilities and three studies included travelers returning from endemic countries. Fifty studies enrolled pregnant and or puerperal women, three studies enrolled infants at birth and collected retrospective information on ZIKV symptoms during pregnancy, and one study included women of reproductive age regardless of pregnancy status. Figure 2 shows the duration of enrollment and infant and child follow-up by type of funding and Figure 3 shows the study size, enrollment start date and ZIKV positivity by study design. Duration of enrollment ranged from 1 to 60 months (median=18; IQR=11, 28 months) (Table 1). The most frequent study design was prospective cohort (83%). Surveillance studies (15%), with recruitment based on clinical or laboratory criteria (e.g., PCR or immunoassays), were by default case-only studies and often included larger sample sizes. Studies that limited enrollment to ZIKV positive women (32%) or had a higher percentage of ZIKV positive women often started enrollment before the declaration of the PHEIC in 2016 (Figure 3). Self-funded studies (n=7) and studies receiving local or national funding (n=12) generally initiated enrollment before the declaration of the PHEIC while studies receiving international funding mostly started enrolment months after (Figure 2).

Table 1. Summary table of study-level characteristics among participants of the ZIKV-IPD-MA metadata survey

		Study's Origin	
**Characteristic**	**Overall N = 54[1]**	**Brazil N = 23 (43%)**	**Other Countries N = 31(57%)**
**No. Pregnant women**			
Median (IQR)	268 (100, 698)	178 (98, 522)	436 (113, 1,108)
**No. ZIKV (+)**			
Median (IQR)	68 (18, 182)	66 (47, 150)	72 (16, 182)
**% ZIKV (+) per study**			
Median (IQR)	47 (3, 100)	68 (7, 100)	36 (2, 100)
**No. Children**			
Median (IQR)	154 (54, 487)	130 (54, 357)	220 (52, 616)
**Study design**			
Case cohort	1 (2%)	1 (4%)	0 (0%)
Cohort	45 (83%)	20 (87%)	25 (81%)
Surveillance	8 (15%)	2 (9%)	6 (19%)
**Source population**			
Community	6 (11%)	4 (17%)	2 (6.6%)
Community & healthcare facility	8 (15%)	6 (26%)	2 (6.6%)
Community + travelers	2 (4%)	0 (0%)	2 (6.6%)
Healthcare facility	37 (68%)	13 (57%)	24 (77%)
Travelers	1 (2%)	0 (0%)	1 (3.2%)
**Enrollment criteria**			
Children only	3 (5%)	3 (13%)	0 (0%)
Not pregnant	1 (2%)	1 (4%)	0 (0%)
Pregnant and/or puerperal women	50 (93%)	19 (83%)	31 (100%)
**Inclusion limited to ZIKV(+) pregnant women**	17 (32%)	6 (26%)	11 (37%)

For peer review only - http://bmjopen.bmj.com/site/about/guidelines.xhtml

Characteristic	Overall N = 54[1]	Study's Origin Brazil N = 23 (43%)	Other Countries N = 31(57%)
**Duration of enrollment (months)**			
Median (IQR)	18 (11, 28)	18 (11, 27)	18 (11, 27)
**Duration of follow-up (months)**			
Median (IQR)	24 (15, 29)	24 (12, 36)	24 (18, 24)
**Received funding**	47 (87%)	20 (87%)	27 (87%)
**Type of Funding Received**			
International funding	22 (41%)	4 (17%)	18 (58%)
Multiple sources	13 (24%)	10 (43%)	3 (9.7%)
National/local funding	12 (22%)	6 (26%)	6 (19%)
No/self-funding	7 (13%)	3 (13%)	4 (13%)
**Maternal ZIKV assessment**	51 (94%)	20 (87%)	31 (100%)
**Maternal immunoassay**	35/51 (69%)	13/20 (65%)	19/31 (61%)
**Maternal PCR**	48/51 (94%)	17/20 (85%)	31 (100%)
**Maternal PRNT**	12/51 (24%)	5/20 (25%)	7/31(23%)
**DENV testing**	35/51 (69%)	15/20 (75%)	20/31 (64%)
**CHIKV testing**	31/51 (61%)	14/20 (70%)	17/31 (61%)
**HIV testing**	44/52 (85%)	19/23(83%)	25/29 (86%)
**STORCH testing**	40/51 (78%)	19/23 (83%)	21/28 (75%)
**Fetal ZIKV assessment**	27/53 (51%)	10/22 (45%)	17/31 (55%)
**Infant ZIKV assessment**	41/53 (77%)	17/22 (77%)	24/31 (77%)
**Prenatal microcephaly assessment**	40 (74%)	16 (70%)	24 (77%)
**Pre & postnatal microcephaly assessment**	51 (94%)	23 (100%)	28 (90%)
**Fetal ultrasounds**	39/53 (74%)	15/23 (65%)	24/30 (80%)
**Postnatal ultrasound**	36/53 (68%)	13/23 (57%)	23/30 (77%)
**Fetal MRIs**	10/52 (19%)	3/23 (13%)	7/29 (24%)
**Postnatal MRI**	17/52 (33%)	8/23 (35%)	9/29 (31%)
**CZS assessment**	46 (85%)	22 (96%)	24 (77%)
**CZS identification criteria**			
Study definition	15/46 (33%)	7/22 (32%)	8/24 (33%)
WHO/CDC/MoH	31/46 (67%)	15/22 (68%)	16/24 (67%)
**Genetic screening**	9/53 (17%)	2/23 (8.7%)	7/30 (23%)
**Performed child developmental assessment**	40/53 (75%)	16/22 (73%)	24/31 (77%)
**Documented termination of pregnancy**	30 (55%)	9 (39%)	21(68%)

[1] Total number of studies, unless otherwise specified is (n= 54 for all studies; n=23 for Brazilian studies and n=31 for other countries). Interquartile Range (IQR); Confirmed ZIKV Positive (ZIKV (+); Polymerase Chain Reaction (PCR); plaque reduction neutralization test (PRNT); Dengue Virus (DENV); Chikungunya Virus (CHIKV); Human Immunodeficiency Virus (HIV); STORCH (Syphilis; Toxoplasmosis, Rubella, Cytomegalovirus and Herpes Testing); Magnetic Resonance Imaging (MRI); Congenital Zika Syndrome (CZS); World Health Organization (WHO); Center for Diseases Control (CDC); Ministry of Health (MoH).

Exposure ascertainment

Assessment of ZIKV infection in pregnant or puerperal women, and fetal and infant ascertainment is presented in Table 1 and Figures 4-5. Assessment of maternal ZIKV status (n=51) included laboratory confirmation alone in 38 studies (74%) and clinical definitions plus laboratory confirmation in 13 studies (25%). The median proportion of ZIKV-positive women per study was 47% (IQR=3, 100%). The laboratory assessment of ZIKV in pregnant women included PCR (94%), enzyme-linked immunosorbent assays (ELISA) for immunoglobulin M (IgM) or immunoglobulin G (IgG) (69%), an

For peer review only - http://bmjopen.bmj.com/site/about/guidelines.xhtml

PRNTs (24%). From the studies performing PCR, 71% reported cycle threshold (Ct) values and 73% reported QC procedures; 74% of studies conducting ELISAs, reported QC procedures. Four surveillance-based studies where the testing was done at local or national surveillance laboratories did not provide details of laboratory techniques or QC procedures. Twenty-seven studies ascertained fetal ZIKV exposure using a combination of maternal and fetal laboratory criteria (e.g., diverse testing of fetal tissues or umbilical cord) and used imaging as supporting ascertainment tool. Forty-one studies ascertained ZIKV in infants using PCRs (80%), ELISAs (61%) and PRNTs (12%). Maternal testing for DENV and CHIKV was conducted in 69% (N=35) and 61% (N=31) studies, respectively (Figure 4).

Outcome ascertainment

Antenatal and postnatal ascertainment of microcephaly was completed in 51 studies (94%). Criteria used to define microcephaly followed national or international standards (i.e., WHO, Intergrowth, or MoH definitions) in 42 studies (81%); 10 studies (19%) used their own study-specific criteria. Congenital ZIKV exposure or CZS was assessed in 46 studies (85%), from which 31 (67%) used international standard criteria and 15 (33%) study-specific definitions. Fetal ultrasounds were performed in 39 (74%) studies (range: 1 to 12 ultrasounds per pregnant woman) and 10 studies performed fetal MRIs. Postnatal cranial ultrasounds and brain MRIs were performed in 36 and 17 studies, respectively. Termination of the pregnancy was documented in 30 (55%) studies (Table 1). Duration of follow-up (median=24; IQR=15, 29 months), differed by the ZIKV status of pregnant women, microcephaly status of newborns, and funding source (Figure 2).

Infant and child development were assessed in 40 (75%) studies using several different international scales, some of which were adapted to local languages and contexts[34]. The most used scales were the Bayley Scales of Infant Development (BSID) (75%) and the Ages & Stages Questionnaires® Social-Emotional, Second Edition (ASQ®-SE-2) (52%). The age range for developmental assessment varied greatly (median age =24; IQR=18, 30, range = 3, 60 months). Two studies from the same research group reported applying the Warner Initial Developmental Evaluation of Adaptive and Functional Skills (WIDEA) and four the Alberta Infant Motor Scale (AIMS) to provide further information on developmental and motor delays not captured by the ASQ®-SE-2 and BSID[35] (Appendix 1, Table S2). Five studies reported planning to follow children beyond age five to assess developmental delays that relate to school performance, using the INTERGROWTH-21st Neurodevelopment Assessment (INTER-NDA) or the neuropsychological tests NEPSY®–II.

Covariate ascertainment

For peer review only - http://bmjopen.bmj.com/site/about/guidelines.xhtml

Genetic screening and testing were performed in nine and eight studies, respectively. Figure S2 (Appendix 1) presents the distribution of genetic testing, maternal STORCH and another pathogen ascertainment. Human Immunodeficiency Virus (HIV) testing was conducted in 44 (81%) studies. Fifty-one studies tested for STORCH pathogens, including syphilis (72%), toxoplasmosis (70%), rubella (59%), Cytomegalovirus (CMV) (57%), and herpes (37%). Four studies tested for malaria. Sociodemographic and behavioral factors measured by studies included education (83%), occupation (62%), marital status (77%), household socioeconomic status (71%), maternal exposure to workplace teratogens (33%), exposure to intimate partner violence (18%), recreational drug use/abuse (82%), smoking (82%), alcohol consumption (80%), and ethnicity (67%) (Appendix 1, Table S3). Covariate selection and ascertainment varied by study type (surveillance-based vs cohort), duration, timing related to declaration of PHEIC, location, and funding level and source.

Quality of data and risk of bias assessment

The metadata dataset included information obtained directly from investigators and substantiated by published reports to ensure reliability and quality of the information. The potential sources of bias identified across the participant studies relate to selection bias due to differential selection, as by the presence of symptoms or observable congenital malformations, into the study and follow-up, and measurement error due to exposure and outcome ascertainment which were differential by the presence of symptoms and by ZIKV assay results. Hence, the potential risk of both selection bias and measurement error was likely highest at the beginning of the epidemic, and surveillance-based studies were likely affected by under and overreporting at different stages of the epidemic. The metadata survey demonstrated that study protocols changed importantly over the course of study implementation to account for advances in the understanding of ZIKV presentation (e.g., the high percentage of asymptomatic cases), diagnostics (e.g., cross-reactivity with DENV), these changes increased the likelihood for potential misclassification, and for the ascertainment of long term outcomes (e.g., development of adverse developmental outcomes that were not observed at birth), and to accommodate funding availability, especially in the Americas region. The misclassification of ZIKV infection is foregrounded in our presentation of the diversity of methods, resources, definitions, and classifications applied in studies' ascertainment of maternal, fetal, and infant ZIKV infection across all study settings and designs (Table 1, Figure 4 and 5). Genetic testing, fetal ultrasounds, and MRIs used for outcome ascertainment is presented in Appendix 1, Table S4, and the type of maternal, fetal and infant ZIKV exposure assessment among ZIKV-IPD-MA study participants of the metadata survey are presented in Appendix 1, Tables S5a-S5c.

DISCUSSION

For peer review only - http://bmjopen.bmj.com/site/about/guidelines.xhtml

We conducted a comprehensive metadata survey to collect study-level data on exposure, outcome, and covariate ascertainment to ensure the appropriate harmonization and analysis of participant-level data from ZIKV IPD-MA contributing studies. We found a high level of cross- and within-study heterogeneity in both maternal ZIKV exposure and outcome ascertainment and the analysis of the evolving spectrum of congenital malformations and postnatal onset developmental delays that define CZS.

The range and distribution of study-level metadata have important public health and epidemiological implications related to the: 1) accurate communication of individual- and population-level risk, 2) reliable identification of prognostic factors and effect measure modifiers for ZIKV-related outcomes, and 3) urgent need to develop statistical methods that account properly for the measurement error in both exposure and outcome ascertainment in the context of an IPD-MA. Below we discuss the advantages of our approach to metadata collection, key study-level sources of heterogeneity, and the potential implications of our findings for the analysis of completed or ongoing IPD-MAs and the design of future research responses to emerging pathogens.

First, in the research response to emerging pathogens, stakeholder consultation is likely more effective than a traditional systematic review for identifying eligible studies and obtaining reliable metadata[36-40]. We initially tried to collect study-level metadata from publications or otherwise available protocols. However, we found important differences in design, definitions and ascertainment procedures between the available documentation and investigator self-reports. The conflicting or incomplete information across information sources, especially as related to exposure ascertainment and QC procedures, led us to develop the metadata survey.

Second, our metadata survey identified sources of heterogeneity across and within studies that are not commonly observed in IPD-MAs of well characterized pathogens or in chronic diseases[41-43]. Study and laboratory protocols changed substantially over time, as knowledge about ZIKV evolved, resulting in important heterogeneity across studies[5,9,15]. For example, diagnostic algorithms and testing guidelines evolved from using serology and RT-PCR to include PRNT testing over the course of the epidemic[5,9,14,15,44,45]. The high ZIKV infection attack rates (17-55%) reported during the epidemic across the Americas and the causal link with microcephaly [4,5,17,46-49], prompted a rapid rollout of cohorts of pregnant women. Due to the natural course of the epidemic and to study inclusion criteria, studies initiated around the epidemic peak or immediately after the declaration of PHEIC had higher ZIKV positive rates among pregnant women compared to studies initiated later in the epidemic. Enrollment

For peer review only - http://bmjopen.bmj.com/site/about/guidelines.xhtml

(criteria and duration), the follow-up for pregnant women and their children, and the planned methodological approaches varied by the type of funding. Thus, the natural course of the epidemic, changes to understanding of ZIKV etiology, and funding mechanisms[50] impacted the quality and amount of information collected, further contributing to cross-study heterogeneity.

Third, ZIKV diagnosis and confirmation is challenging[3,9-12,14,15,17,45]. Our findings indicate that the various criteria, definitions, assays, and testing algorithms used for the ZIKV diagnosis are key sources of heterogeneity in ZIKV infection ascertainment. The main challenges for the confirmation of ZIKV infection in our survey were the 1) lack of standardized or consistent guidelines and algorithms for ZIKV diagnosis; 2) ascertainment of ZIKV infection in settings that are endemic for other arboviruses using assays that do not adequately account for cross-reactivity; and 3) lack of ascertainment of fetal ZIKV infection. Most studies indicated that ZIKV infection among pregnant women was ascertained using both clinical and laboratory criteria. However, the range and scope of the clinical criteria was as diverse as the number of studies and the definition of laboratory confirmation included several combinations of immunoassays and molecular techniques, each one with different standards of procedures (SOPs), variable or unknown cutoff points, and different or unknown approaches for QC. The urgency and limited knowledge about ZIKV called for the simultaneous, asynchronous development of diagnostic tests, where researchers converged over time to a somewhat smaller number of more accurate assays or testing algorithms[8,9,14,15,26,31]. This lack of standardization can be seen as both detrimental, if at early stages of the epidemic the testing was based only on the presence of symptoms or if the methods used were less accurate[11,13-16,31], and beneficial because it led to innovation in test development[9,10,12,15,17,26].

Fourth, smaller studies that were launched earlier in the epidemic, which were often the less resourced, are potentially affected by an outcome and exposure ascertainment that is differential by the probability of infection, which in turn may influence the estimation of the long- and short-term risk of ZIKV effects. Surveillance-based studies include mostly symptomatic individuals who are able to seek and receive healthcare attention (e.g., diagnosis and standard care)[51]. Given the high proportion of asymptomatic ZIKV-infections[6,7,47,52], some surveillance-based studies are likely affected by underreporting[15,46,52-54]. Neither studies launched early in the epidemic nor surveillance sites included comparable data on women who were not infected with ZIKV during pregnancy. In addition, health seeking behavior for symptomatic individuals depends also on socioeconomic determinants of access to health care[46,53], potentially leading to differential outcome ascertainment, representing another source of bias[17,53,55]. For instance, in endemic areas, the presence of symptoms was related to antenatal healthcare practices (e.g., number of antenatal visits, ZIKV testing, and ultrasounds and medical

For peer review only - http://bmjopen.bmj.com/site/about/guidelines.xhtml

termination of the pregnancies) during the epidemic[46], leading to potential differential assessment of exposure and outcomes, including for miscarriages, induced and spontaneous fetal losses, and congenital malformations. Additionally, cultural sensitivity and restricted legal environments meant that only half of studies measured termination of pregnancy, which biases results differently across sites depending on abortion access[56]. Furthermore, a wide range of definitions of congenital Zika were implemented. Some studies used the WHO definition of CZS, others a modified version including isolated manifestations like ophthalmological abnormalities, and several the presence of microcephaly at birth to define CZS. Therefore, the few cohort studies with longer follow-up are valuable resources for understanding the longer-term consequences of congenital ZIKV infection among infants born without manifestations of congenital ZIKV, because studies with short or no follow-up for children would not have captured potential cognitive or developmental issues.[1,3,34,35]

These four issues pose both statistical and logistical challenges when harmonizing and analyzing data in the context of an IPD-MA of an emerging pathogen. The ZIKV-IPD Consortium recognized that existing methods for addressing the correlated sources of heterogeneity in the research response to ZIKV (e.g., dependency between timing of symptoms, test accuracy, and differential ascertainment for both exposure and outcome, etc.) are inadequate. In response to this gap, the Consortium launched a statistical WG to develop novel approaches to address heterogeneity and measurement error in the context of IPD-MAs of emerging pathogens. The statistical WG will use the findings from this metadata survey to guide the development of an analytic plan that accounts for the different sources of correlated measurement error. The statistical analysis plan will extend existing methods for measurement error and clinical risk prediction to address the specific challenges of an IPD-MA of an emerging pathogen. Methodological approaches for accounting for correlated sources of measurement error and the high levels of heterogeneity in the research response to emerging pathogens will be context- and pathogen-specific. Although specific recommendations or solutions to account for the diverse sources of heterogeneity are desirable, an in-depth proposal for the methods to address these statistical challenges is outside of the scope of this descriptive analysis.

Limitations and Strengths

The inclusion of ongoing studies presented important challenges in that key aspects of exposure and outcome ascertainment have continued to evolve as studies obtain additional funding to retest samples or to continue and expand follow-up[50]. In some cases, studies changed exposure ascertainment procedures or laboratory QC procedures and were not able to record when changes were made or what had been changed, which represents missing or misclassified metadata, further complicating the assessment of the accuracy of exposure ascertainment[2,9,17]. Although we conducted regular follow-ups

For peer review only - http://bmjopen.bmj.com/site/about/guidelines.xhtml

over a nine-month period to obtain the information, some researchers did not respond, reasonably because they were on the frontlines of the COVID-19 pandemic response. This impacted the timelines for participant- and metadata acquisition and review for most studies. Nonetheless, the multiple interactions required with the investigators enriched the analytical process, ensured the robustness and quality of the data, and strengthened the cross-consortium communication.

The identification of the above-mentioned sources of heterogeneity constitutes the main strength of this study. This is the first study to assemble a comprehensive reporting of study-level heterogeneity of ZIKV-related cohorts of pregnant women and their infants and children, which could only be gathered through investigators' self-report. The final metadata and participant-level data sets will represent the largest resource of longitudinal, participant-level data on maternal-infant/child dyads for many of the countries where research teams have contributed data for the IPD-MA. Ensuring that participant-level data are accompanied by well characterized metadata, coupled with engaging the teams that collected the data and external subject matter experts, will help the ZIKV IPD Consortium ensure the appropriate estimation of outcome and imputation models. This detailed study-level metadata will facilitate the way that IPD can be harmonized and analyzed, including the identification of study-related sources of heterogeneity and clinically related participant-level sources of heterogeneity[36,37,40]. Given the limited understanding of the distribution of adverse fetal, infant, and child outcomes related to congenital exposures, the meta- and participant-level dataset will represent a tremendous cross- and within-country resource for social, medical, infectious, and other exposures, that goes beyond ZIKV.

Conclusion

This analysis provides a comprehensive overview of study-level heterogeneity related to participant-level data available from ZIKV-related cohorts and surveillance systems of pregnant women and their infants and children. The challenges confronted and addressed here include: 1) the need to consult with researchers and local and international health authorities early in the outbreak to identify related studies; 2) the need to engage directly with investigators to understand and document heterogeneity in study design, definitions, and implementation, which are inconsistently or incompletely reported in the published literature and include information according to the existing knowledge of the problem at that given time; and 3) the need to consistently and regularly adjust research targets and timelines to account for continuing changes to study protocols, teams, agendas and funding availability, ensuring the quality of the studies. Our study will serve to inform ongoing and future collaborations that prospectively or retrospectively harmonize IPD and to drive the development of novel statistical methods that can address correlated sources of measurement error in IPD-MA of emerging pathogens.

For peer review only - http://bmjopen.bmj.com/site/about/guidelines.xhtml

Contributions

LM, MC, BL were responsible for the study conception and design. LM designed and conducted database searches to identify eligible studies. MC, LM, BL, and PS contributed to data extraction and coding for the metadata analysis. MC, LM, and BL contributed to the data analysis and interpretation. MC and LM wrote and BL, PS contributed to the first draft of the manuscript; ZIKV-IPD Consortium participants contributed to subsequent versions of the manuscript. All authors provided a critical review and approved the final manuscript. LM, MC, BL, PS are the guarantors; they have full access to all the data in the study and take responsibility for the integrity of the data and the accuracy of the data analyses.

Declaration of interests

All authors have completed the ICMJE uniform disclosure form and declare: no support from any organization for the submitted work; no financial relationships with any organizations that might have an interest in the submitted work in the previous three years for the main authors. No funder had any role in the design and conduct of this study; collection, management, analysis, and interpretation of the data; preparation, review, or approval of the manuscript; and decision to submit the manuscript for publication. Consortium members and study Principal Investigators (PIs) or collaborators contributing data to the ZIKV-IPD-MA members have provided declarations of potential or perceived conflict of interests in Appendix 3.

Acknowledgements

The authors would like to acknowledge the valuable contributions of all researchers, fieldworkers, and participants of the contributing studies of the ZIKV-IPD-MA.

Funding

This systematic review and summary of metadata was drafted and revised with funding support provided by the Wellcome Trust to the WHO Department of Reproductive Health and Research Human Reproduction Program, grant numbers 206532/Z/17/Z and 216002/Z/19/Z, respectively. Dr. Levis was supported by a Fonds de Recherche du Québec – Santé Postdoctoral Training Award. No other main authors reported funding for primary studies or for their work on the present study. Funding sources for each participating study in the ZIKV-IPD-MA are provided in the supplementary material. The funding source had no role in the drafting of the manuscript.

Data Sharing

For peer review only - http://bmjopen.bmj.com/site/about/guidelines.xhtml

Key metadata are presented in the tables in the manuscript and corresponding appendices. Requests for access to the full metadata dataset for the 54 contributing studies should be made to the corresponding author.

Ethics Statement

The IPD-MA protocol was deemed exempt from ethical review by the WHO Ethics Review Committee and the Emory University Institutional Review Board because the IPD-MA uses de-identified, participant-level data from studies that have received ethical approval for the same objectives as the original studies. In keeping with recommendations for the re-use of de-identified participant-level data in an IPD-MA, we conducted a qualitative study to assess source communities' perceptions regarding the reuse of their data for an IPD-MA and preferences for the communication of ZIKV-related risk in the presence of uncertainty in both the diagnosis and the probability of ZIKV-related adverse events, given a positive diagnosis.

Role of the funding source

The study funder had no role in study design, data collection, data analysis, data interpretation, or writing of the report. The corresponding author had full access to all data in the study and final responsibility for the decision to submit for publication.

Disclaimers

World Health Organization: The authors alone are responsible for the views expressed in this article and they do not necessarily represent the views, decisions, or policies of the institutions with which they are affiliated. United States Centers for Disease Control and Prevention: The findings and conclusions in this report are those of the authors and do not represent the official position of the Centers for Disease Control and Prevention (CDC).

For peer review only

REFERENCES

1. Martins MM, Alves da Cunha AJL, Robaina JR, Raymundo CE, Barbosa AP, Medronho RdA. Fetal, neonatal, and infant outcomes associated with maternal Zika virus infection during pregnancy: A systematic review and meta-analysis. *PLOS ONE* 2021; **16**(2): e0246643.
2. Zika Virus Individual Participant Data C. The Zika Virus Individual Participant Data Consortium: A Global Initiative to Estimate the Effects of Exposure to Zika Virus during Pregnancy on Adverse Fetal, Infant, and Child Health Outcomes. *Tropical medicine and infectious disease* 2020; **5**(4): 152.
3. Freitas DA, Souza-Santos R, Carvalho LMA, et al. Congenital Zika syndrome: A systematic review. *PLoS One* 2020; **15**(12): e0242367.
4. WHO. Emergency Committee on Zika virus and observed increase in neurological disorders and neonatal malformations. Geneva, Switzerland: WHO, 2016.
5. WHO. Zika virus infection: global update on epidemiology and potentially associated clinical manifestations. *Releve epidemiologique hebdomadaire* 2016; **91**(7): 73-81.
6. Alvarado MG, Schwartz DA. Zika Virus Infection in Pregnancy, Microcephaly, and Maternal and Fetal Health: What We Think, What We Know, and What We Think We Know. *Archives of pathology & laboratory medicine* 2016; **141**(1): 26-32.
7. World Health Organization (WHO). Zika epidemiology update. In: WHO, editor. Health Topics. July 2019 ed. Geneva, Switzerland; 2019. p. https://www.who.int/publications/m/item/zika-epidemiology-update.
8. Waggoner JJ, Gresh L, Vargas MJ, et al. Viremia and Clinical Presentation in Nicaraguan Patients Infected With Zika Virus, Chikungunya Virus, and Dengue Virus. *Clinical Infectious Diseases* 2016; **63**(12): 1584-90.
9. Waggoner JJ, Pinsky BA. Zika Virus: Diagnostics for an Emerging Pandemic Threat. *Journal of Clinical Microbiology* 2016; **54**(4): 860-7.
10. Fischer C, Pedroso C, Mendrone A, Jr., et al. External Quality Assessment for Zika Virus Molecular Diagnostic Testing, Brazil. *Emerging infectious diseases* 2018; **24**(5): 888-92.
11. Fritzell C, Rousset D, Adde A, Kazanji M, Van Kerkhove MD, Flamand C. Current challenges and implications for dengue, chikungunya and Zika seroprevalence studies worldwide: A scoping review. *PLOS Neglected Tropical Diseases* 2018; **12**(7): e0006533.
12. Voermans JJC, Pas SD, van der Linden A, et al. Whole-Blood Testing for Diagnosis of Acute Zika Virus Infections in Routine Diagnostic Setting. *Emerging infectious diseases* 2019; **25**(7): 1394-6.
13. Qiao L, Martelli CMT, Raja AI, et al. Epidemic preparedness: Prenatal Zika virus screening during the next epidemic. *BMJ Global Health* 2021; **6**(6): e005332.
14. Ximenes RAdA, Miranda-Filho DdB, Brickley EB, et al. Zika virus infection in pregnancy: Establishing a case definition for clinical research on pregnant women with rash in an active transmission setting. *PLOS Neglected Tropical Diseases* 2019; **13**(10): e0007763.
15. Pan American Health Organization (PAHO). Tool for the diagnosis and care of patients with suspected arboviral diseases. 1st Edition ed. Washington: PAHO; 2017.
16. Sanchez Clemente N, Brickley EB, Furquim de Almeida M, Witkin SS, Duarte Passos S, Group tJZC. Can Zika Virus Infection in High Risk Pregnant Women Be Differentiated on the Basis of Symptoms? *Viruses* 2020; **12**(11): 1263.
17. Simmons G, Stone M, Busch MP. Arbovirus Diagnostics: From Bad to Worse due to Expanding Dengue Virus Vaccination and Zika Virus Epidemics. *Clinical Infectious Diseases* 2017; **66**(8): 1181-3.
18. Aubry F, Jacobs S, Darmuzey M, et al. Recent African strains of Zika virus display higher transmissibility and fetal pathogenicity than Asian strains. *Nature Communications* 2021; **12**(1): 916.

For peer review only - http://bmjopen.bmj.com/site/about/guidelines.xhtml

19. Pierson TC, Graham BS. Zika Virus: Immunity and Vaccine Development. *Cell* 2016; **167**(3): 625-31.
20. Castanha PMS, Marques ETA. A Glimmer of Hope: Recent Updates and Future Challenges in Zika Vaccine Development. *Viruses* 2020; **12**(12): 1371.
21. Shewale JB, Ganduglia Cazaban CM, Waller DK, Mitchell LE, Langlois PH, Agopian AJ. Microcephaly inpatient hospitalization and potential Zika outbreak in Texas: A cost and predicted economic burden analysis. *Travel Medicine and Infectious Disease* 2019; **30**: 67-72.
22. Lee BY, Alfaro-Murillo JA, Parpia AS, et al. The potential economic burden of Zika in the continental United States. *PLOS Neglected Tropical Diseases* 2017; **11**(4): e0005531.
23. Thompson R, Martin Del Campo J, Constenla D. A review of the economic evidence of Aedes-borne arboviruses and Aedes-borne arboviral disease prevention and control strategies. *Expert Review of Vaccines* 2020; **19**(2): 143-62.
24. Puntasecca CJ, King CH, LaBeaud AD. Measuring the global burden of chikungunya and Zika viruses: A systematic review. *PLOS Neglected Tropical Diseases* 2021; **15**(3): e0009055.
25. Wilder-Smith A, Wei Y, Araújo TVBd, et al. Understanding the relation between Zika virus infection during pregnancy and adverse fetal, infant and child outcomes: a protocol for a systematic review and individual participant data meta-analysis of longitudinal studies of pregnant women and their infants and children. *BMJ Open* 2019; **9**(6): e026092.
26. World Health Organization (WHO). Zika Virus Research and Development (R&D) Roadmap. Geneva, Switzerland: World Health Organization, 2021.
27. Harris PA, Taylor R, Minor BL, et al. The REDCap consortium: Building an international community of software platform partners. *Journal of Biomedical Informatics* 2019; **95**: 103208.
28. Lebov JF, Arias JF, Balmaseda A, et al. International prospective observational cohort study of Zika in infants and pregnancy (ZIP study): study protocol. *BMC Pregnancy Childbirth* 2019; **19**(1): 282-.
29. Avelino-Silva VI, Mayaud P, Tami A, et al. Study protocol for the multicentre cohorts of Zika virus infection in pregnant women, infants, and acute clinical cases in Latin America and the Caribbean: the ZIKAlliance consortium. *BMC Infectious Diseases* 2019; **19**(1): 1081.
30. Ades AE, Brickley EB, Alexander N, et al. Zika virus infection in pregnancy: a protocol for the joint analysis of the prospective cohort studies of the ZIKAlliance, ZikaPLAN and ZIKAction consortia. *BMJ Open* 2020; **10**(12): e035307.
31. Reynolds MR, Jones AM, Petersen EE, et al. Vital Signs: Update on Zika Virus-Associated Birth Defects and Evaluation of All U.S. Infants with Congenital Zika Virus Exposure - U.S. Zika Pregnancy Registry, 2016. *MMWR Morb Mortal Wkly Rep* 2017; **66**(13): 366-73.
32. Center for Disease Control and Prevention (CDC). US Zika Pregnancy and Infant Registry. Zika and Pregnancy Home; Research and Tracking Atlanta, GA: Content source: National Center on Birth Defects and Developmental Disabilities, Centers for Disease Control and Prevention (CDC). 2016. p. https://www.cdc.gov/pregnancy/zika/research/registry.html.
33. Shapiro-Mendoza CK, Rice ME, Galang RR, et al. Pregnancy Outcomes After Maternal Zika Virus Infection During Pregnancy - U.S. Territories, January 1, 2016-April 25, 2017. *MMWR Morb Mortal Wkly Rep* 2017; **66**(23): 615-21.
34. K. Connery A, Berrios-Siervo G, Arroyave P, et al. Responding to the Zika Epidemic: Preparation of a Neurodevelopmental Testing Protocol to Evaluate Young Children in Rural Guatemala. *The American Journal of Tropical Medicine and Hygiene* 2019; **100**(2): 438-44.
35. Mulkey SB, Arroyave-Wessel M, Peyton C, et al. Neurodevelopmental Abnormalities in Children With In Utero Zika Virus Exposure Without Congenital Zika Syndrome. *JAMA Pediatrics* 2020; **174**(3): 269-76.
36. Volk CJ, Lucero Y, Barnas K. Why is Data Sharing in Collaborative Natural Resource Efforts so Hard and What can We Do to Improve it? *Environmental Management* 2014; **53**(5): 883-93.

For peer review only - http://bmjopen.bmj.com/site/about/guidelines.xhtml

37. Samuel S, König-Ries B. Understanding experiments and research practices for reproducibility: an exploratory study. *PeerJ* 2021; **9**: e11140.
38. Lancaster GA, McCray G, Kariger P, et al. Creation of the WHO Indicators of Infant and Young Child Development (IYCD): metadata synthesis across 10 countries. *BMJ Global Health* 2018; **3**(5): e000747.
39. Burkot TR, Farlow R, Min M, Espino E, Mnzava A, Russell TL. A global analysis of National Malaria Control Programme vector surveillance by elimination and control status in 2018. *Malaria Journal* 2019; **18**(1): 399.
40. Levis B, Hattle M, Riley RD. PRIME-IPD SERIES Part 2. Retrieving, checking, and harmonizing data are underappreciated challenges in individual participant data meta-analyses. *Journal of clinical epidemiology* 2021.
41. Salameh J-P, Bossuyt PM, McGrath TA, et al. Preferred reporting items for systematic review and meta-analysis of diagnostic test accuracy studies (PRISMA-DTA): explanation, elaboration, and checklist. *BMJ* 2020; **370**: m2632.
42. Riley RD, Lambert PC, Abo-Zaid G. Meta-analysis of individual participant data: rationale, conduct, and reporting. *BMJ* 2010; **340**: c221.
43. Harris PA, Taylor R, Thielke R, Payne J, Gonzalez N, Conde JG. Research electronic data capture (REDCap)—A metadata-driven methodology and workflow process for providing translational research informatics support. *Journal of Biomedical Informatics* 2009; **42**(2): 377-81.
44. Karwowski MP, Nelson JM, Staples JE, et al. Zika Virus Disease: A CDC Update for Pediatric Health Care Providers. *Pediatrics* 2016; **137**(5).
45. Ribeiro MRC, Khouri R, Sousa PS, et al. Plaque Reduction Neutralization Test (PRNT) in the Congenital Zika Syndrome: Positivity and Associations with Laboratory, Clinical, and Imaging Characteristics. *Viruses* 2020; **12**(11): 1244.
46. Lozier M, Adams L, Febo MF, et al. Incidence of Zika Virus Disease by Age and Sex — Puerto Rico, November 1, 2015–October 20, 2016. *Morbidity and Mortality Weekly Report* 2016; **65**(44): 1219-23.
47. Ellington SR, Devine O, Bertolli J, et al. EStimating the number of pregnant women infected with zika virus and expected infants with microcephaly following the zika virus outbreak in puerto rico, 2016. *JAMA Pediatrics* 2016; **170**(10): 940-5.
48. de Araújo TVB, Ximenes RAA, Miranda-Filho DB, et al. Association between microcephaly, Zika virus infection, and other risk factors in Brazil: final report of a case-control study. *Lancet Infect Dis* 2018; **18**(3): 328-36.
49. Brasil P, Pereira JP, Moreira ME, et al. Zika Virus Infection in Pregnant Women in Rio de Janeiro. *New England Journal of Medicine* 2016; **375**(24): 2321-34.
50. Koopmans M, de Lamballerie X, Jaenisch T, et al. Familiar barriers still unresolved—a perspective on the Zika virus outbreak research response. *The Lancet Infectious Diseases* 2019; **19**(2): e59-e62.
51. Gibbons CL, Mangen M-JJ, Plass D, et al. Measuring underreporting and under-ascertainment in infectious disease datasets: a comparison of methods. *BMC Public Health* 2014; **14**(1): 147.
52. Kucharski AJ, Funk S, Eggo RM, Mallet H-P, Edmunds WJ, Nilles EJ. Transmission Dynamics of Zika Virus in Island Populations: A Modelling Analysis of the 2013–14 French Polynesia Outbreak. *PLOS Neglected Tropical Diseases* 2016; **10**(5): e0004726.
53. Carabali M, Jaramillo-Ramirez GI, Rivera VA, Mina Possu N-J, Restrepo BN, Zinszer K. Assessing the reporting of Dengue, Chikungunya and Zika to the National Surveillance System in Colombia from 2014–2017: A Capture-recapture analysis accounting for misclassification of arboviral diagnostics. *PLOS Neglected Tropical Diseases* 2021; **15**(2): e0009014.

For peer review only - http://bmjopen.bmj.com/site/about/guidelines.xhtml

54. Hernández-Ávila JE, Palacio-Mejía LS, López-Gatell H, et al. Zika virus infection estimates, Mexico. *Bulletin of the World Health Organization* 2018; **96**(5): 306-13.
55. Oliveira Melo AS, Malinger G, Ximenes R, Szejnfeld PO, Alves Sampaio S, Bispo de Filippis AM. Zika virus intrauterine infection causes fetal brain abnormality and microcephaly: tip of the iceberg? *Ultrasound in Obstetrics & Gynecology* 2016; **47**(1): 6-7.
56. Carabali M, Austin N, King NB, Kaufman JS. The Zika epidemic and abortion in Latin America: a scoping review. *Global Health Research and Policy* 2018; **3**(1): 15.

Figure Legends:

Figure 1. Metadata Flowchart (1.A) and ZIKV-Metadata Countries & Territories Participants (1.B). *The difference between the number of study sites and unique datasets is explained by the inclusion of studies using data from a single surveillance system or multisite studies for which the metadata (e.g., source population, ZIKV assays, and outcome ascertainment) did not vary across sites.

Figure 2. Duration of Enrollment and Follow-up for Children by Type of Funding Among Participants of the ZIKV-IPD-MA Metadata Survey

Figure 3. ZIKV Positivity by Study's Start date, Sample Size, and Study Design Among Participants of the ZIKV-IPD-MA Metadata survey

Figure 4. Overview of ZIKV Ascertainment Among Participants of the ZIKV-IPD-MA Metadata Survey

Figure 5. ZIKV Ascertainment and Type of Laboratory Testing and Other Criteria Among Participants of the ZIKV-IPD-MA Metadata Survey

For peer review only - http://bmjopen.bmj.com/site/about/guidelines.xhtml

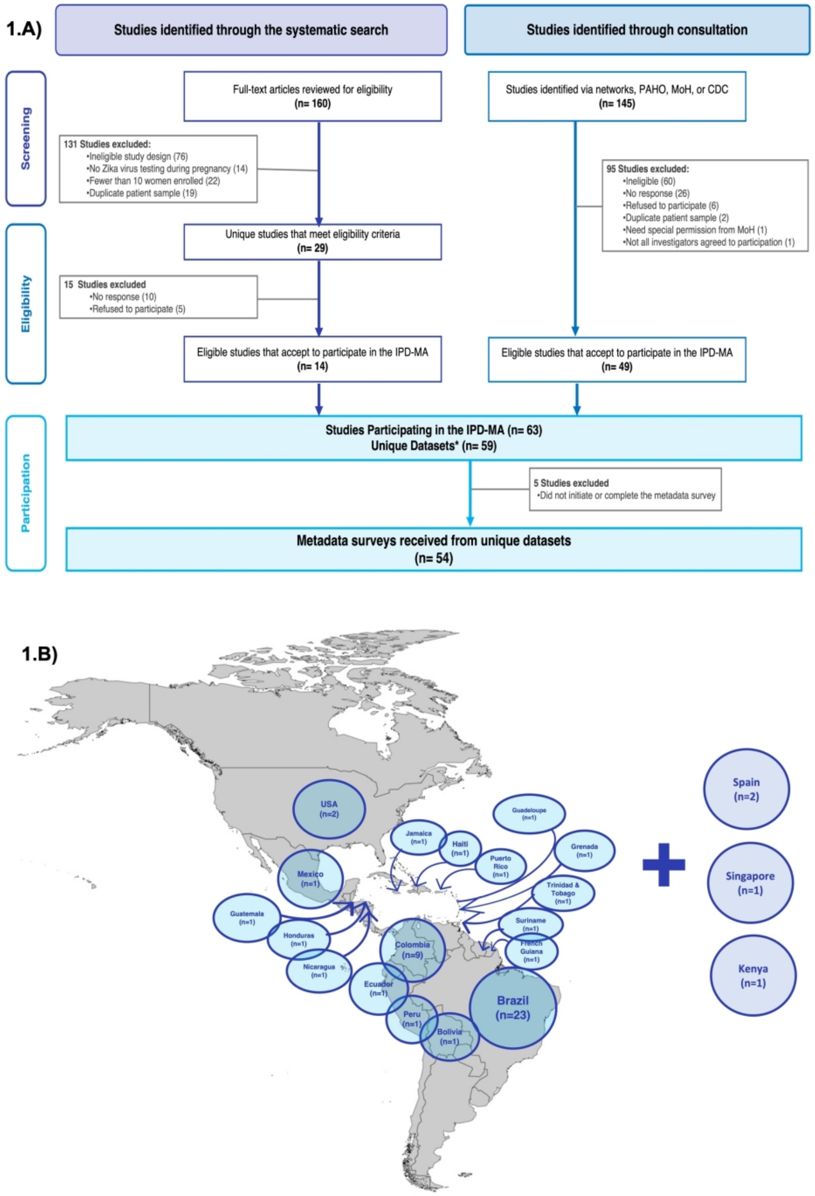

Figure 1. Metadata Flowchart (1.A) and ZIKV-Metadata Countries & Territories Participants (1.B). *The difference between the number of study sites and unique datasets is explained by the inclusion of studies using data from a single surveillance system or multisite studies for which the metadata (e.g., source population, ZIKV assays, and outcome ascertainment) did not vary across sites.

179x262mm (330 x 330 DPI)

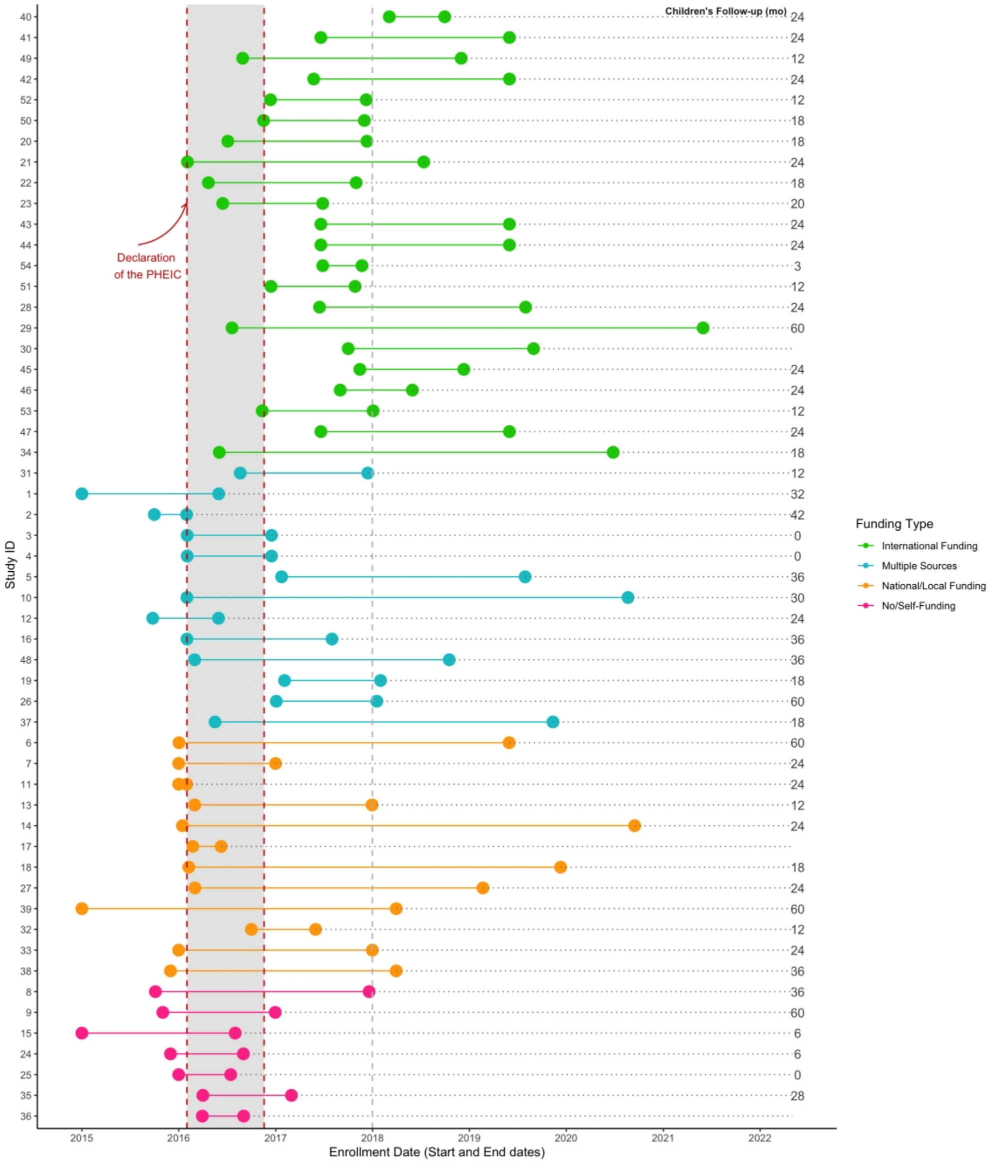

Figure 2. Duration of Enrollment and Follow-up for Children by Type of Funding Among Participants of the ZIKV-IPD-MA Metadata Survey

812x948mm (72 x 72 DPI)

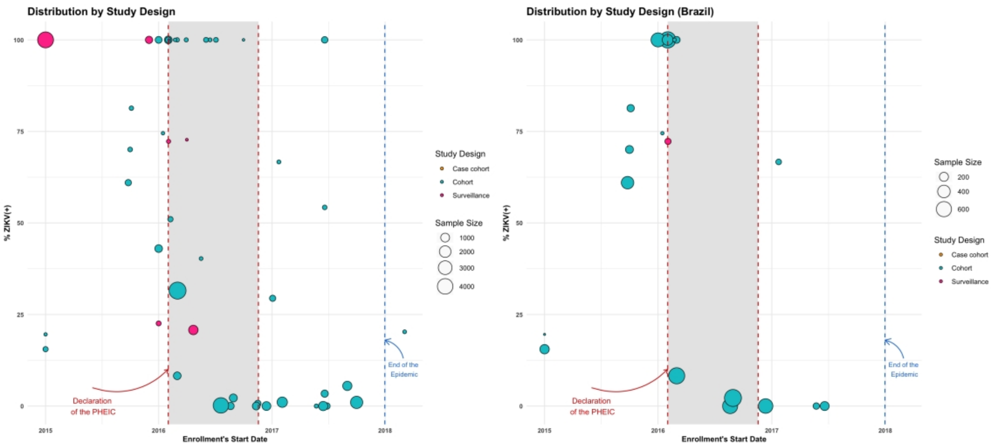

Figure 3. ZIKV Positivity by Study's Start date, Sample Size, and Study Design Among Participants of the ZIKV-IPD-MA Metadata survey

1219x541mm (72 x 72 DPI)

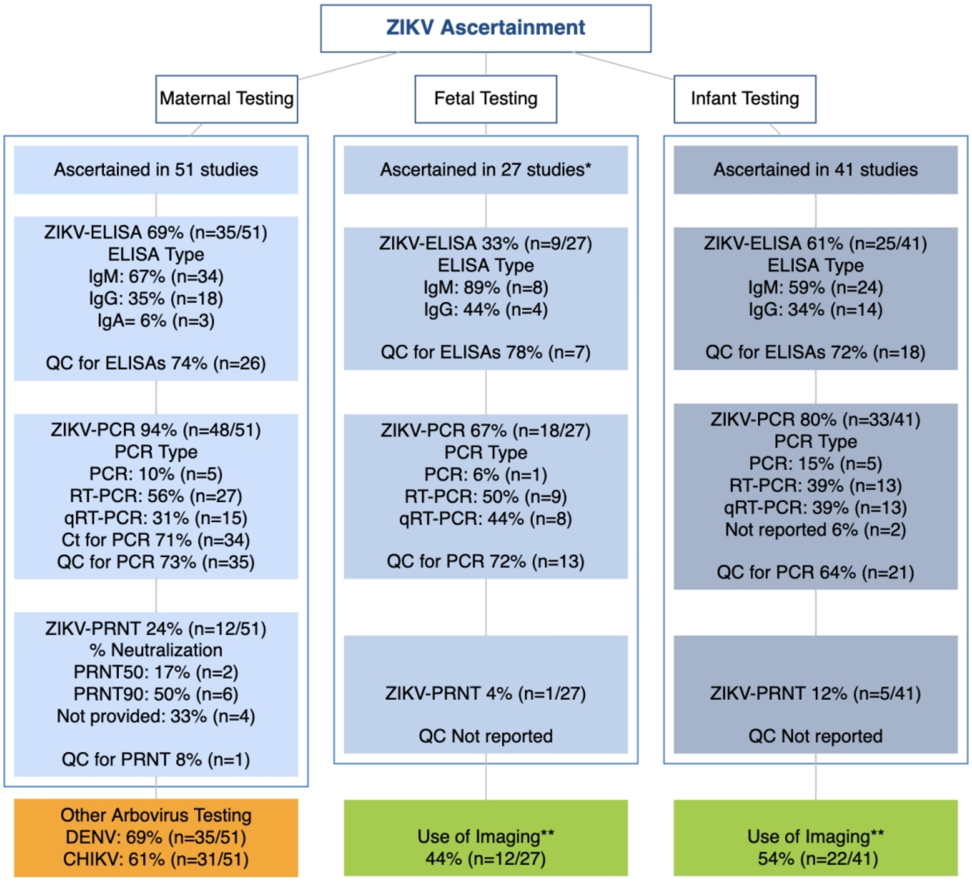

Figure 4. Overview of ZIKV Ascertainment Among Participants of the ZIKV-IPD-MA Metadata Survey

272x249mm (144 x 144 DPI)

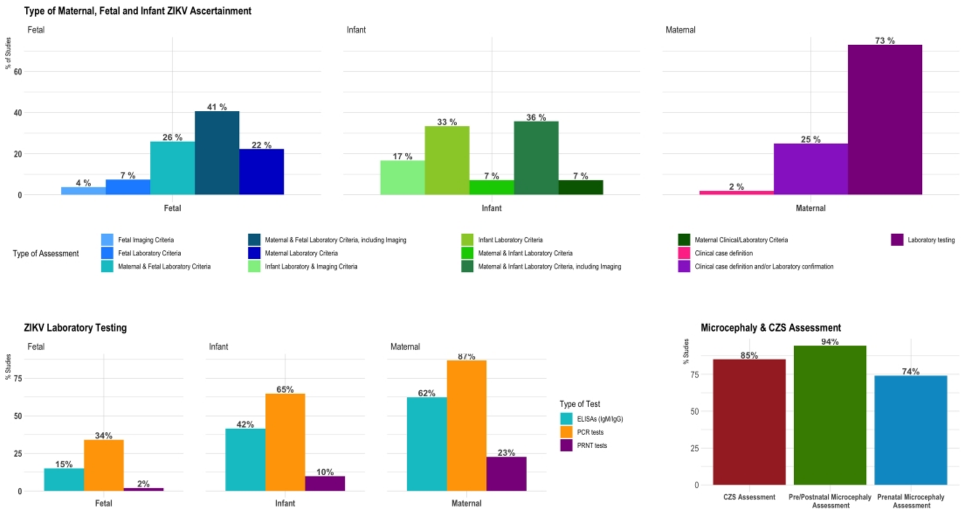

Figure 5. ZIKV Ascertainment and Type of Laboratory Testing and Other Criteria Among Participants of the ZIKV-IPD-MA Metadata Survey

1219x677mm (72 x 72 DPI)

Appendix 1.

Table of Contents

I. **Zika search strategy** 2

PICO CRITERIA: 2

Zika search, Medline (through Ovid): 2

Zika search, Embase (through Ovid): 2

II. **Screening, eligibility and selection of studies trhough systematic search** 4

Figure S.1. Flowchart for study participation trough systematic search. 4

III. **Supplementary tables with descriptive zikv-ipd-ma metada-related statisitcs and figures** 5

Table S1. Detailed description of ZIKV-IPD-MA studies participating in the metadata survey (Part 1). 5

Table S1. Detailed description of ZIKV-IPD-MA studies participating in the metadata survey (Part 2). 12

Table S2. Infant and child development assessment among ZIKV-IPD-MA study participants of the metadata survey... 17

Figure S2. STORCH and other additional testing among ZIKV-IPD-MA study participants of the metadata survey.... 17

Table S3. Sociodemographic and behavioral factors measured among ZIKV-IPD-MA study participants of the metadata survey. 18

Table S4. Genetic testing, fetal ultrasounds, and MRIs among ZIKV-IPD-MA study participants of the metadata survey 19

Table S5a. Type of maternal ZIKV assessment among ZIKV-IPD-MA study participants of the metadata survey. 20

Table S5b. Type of fetal ZIKV assessment among ZIKV-IPD-MA study participants of the metadata survey. 21

Table S5c. Type of infant ZIKV assessment among ZIKV-IPD-MA study participants of the metadata survey. 22

For peer review only

For peer review only - http://bmjopen.bmj.com/site/about/guidelines.xhtml

I. ZIKA SEARCH STRATEGY

PICO CRITERIA:

Population	Exposure	Comparator	Outcome (open)
Pregnant women and newborns	ZIKV infection during pregnancy	No ZIKV infection during pregnancy	Primary: microcephaly, miscarriage, fetal loss. Secondary: early/late fetal death, stillbirth, ocular abnormalities, hearing loss, neuroimaging abnormalities, etc.

Date: Started June 12, 2018 – Ended July 8, 2018

Zika search, Medline (through Ovid):

For peer

1. exp Zika Virus Infection/ or exp ZIKA VIRUS/
2. (zika or ZIKV).ti,ab,kf.
3. 1 or 2
4. exp Pregnancy/ or exp Maternal Exposure/ or exp "Embryonic and Fetal Development"/ or exp "Congenital, Hereditary, and Neonatal Diseases and Abnormalities"/ or exp Infant/ or exp Child/
5. (pregnan* or matern* or gestation* or perinatal* or birth* or congenital* or newborn* or fetal or fetus* or foetal or foetus* or neonat* or infan* or toddler* or child*).ti,ab,kf.
6. 4 or 5
7. 3 and 6
8. 7 not (exp Animals/ not exp Humans/)

☐	1	exp Zika Virus Infection/ or exp ZIKA VIRUS/	2407
☐	2	(zika or ZIKV).ti,ab,kf.	4672
☐	3	1 or 2	4784
☐	4	exp Pregnancy/ or exp Maternal Exposure/ or exp "Embryonic and Fetal Development"/ or exp "Congenital, Hereditary, and Neonatal Diseases and Abnormalities"/ or exp Infant/ or exp Child/	3661254
☐	5	(pregnan* or matern* or gestation* or perinatal* or birth* or congenital* or newborn* or fetal or fetus* or foetal or foetus* or neonat* or infan* or toddler* or child*).ti,ab,kf.	2574479
☐	6	4 or 5	4373079
☐	7	3 and 6	1947
☐	8	7 not (exp Animals/ not exp Humans/)	1857

Zika search, Embase (through Ovid):

1. exp Zika virus/ or exp Zika fever/
2. (zika or ZIKV).ti,ab,kw.
3. 1 or 2
4. exp pregnancy/ or exp pregnancy outcome/ or exp high risk pregnancy/ or exp pregnancy complication/ or exp maternal exposure/ or exp fetus/ or exp "functions of embryonic, fetal and placental structures"/ or exp Infant/ or exp infant disease/ or exp child/ or exp childhood disease/
5. (pregnan* or matern* or gestation* or perinatal* or birth* or congenital* or newborn* or fetal or fetus* or foetal or foetus* or neonat* or infan* or toddler* or child*).ti,ab,kw.
6. 4 or 5

For peer review only - http://bmjopen.bmj.com/site/about/guidelines.xhtml

7. 3 and 6
8. 7 not ((exp animal/ or exp nonhuman/) not exp human/)

# ▲	Searches	Results
1	exp Zika virus/ or exp Zika fever/	4757
2	(zika or ZIKV).ti,ab,kw.	5183
3	1 or 2	5788
4	exp pregnancy/ or exp pregnancy outcome/ or exp high risk pregnancy/ or exp pregnancy complication/ or exp maternal exposure/ or exp fetus/ or exp "functions of embryonic, fetal and placental structures"/ or exp Infant/ or exp infant disease/ or exp child/ or exp childhood disease/	4556497
5	(pregnan* or matern* or gestation* or perinatal* or birth* or congenital* or newborn* or fetal or fetus* or foetal or foetus* or neonat* or infan* or toddler* or child*).ti,ab,kw.	3374208
6	4 or 5	5355248
7	3 and 6	2675
8	7 not ((exp animal/ or exp nonhuman/) not exp human/)	2326

review only

II. SCREENING, ELIGIBILITY AND SELECTION OF STUDIES TRHOUGH SYSTEMATIC SEARCH

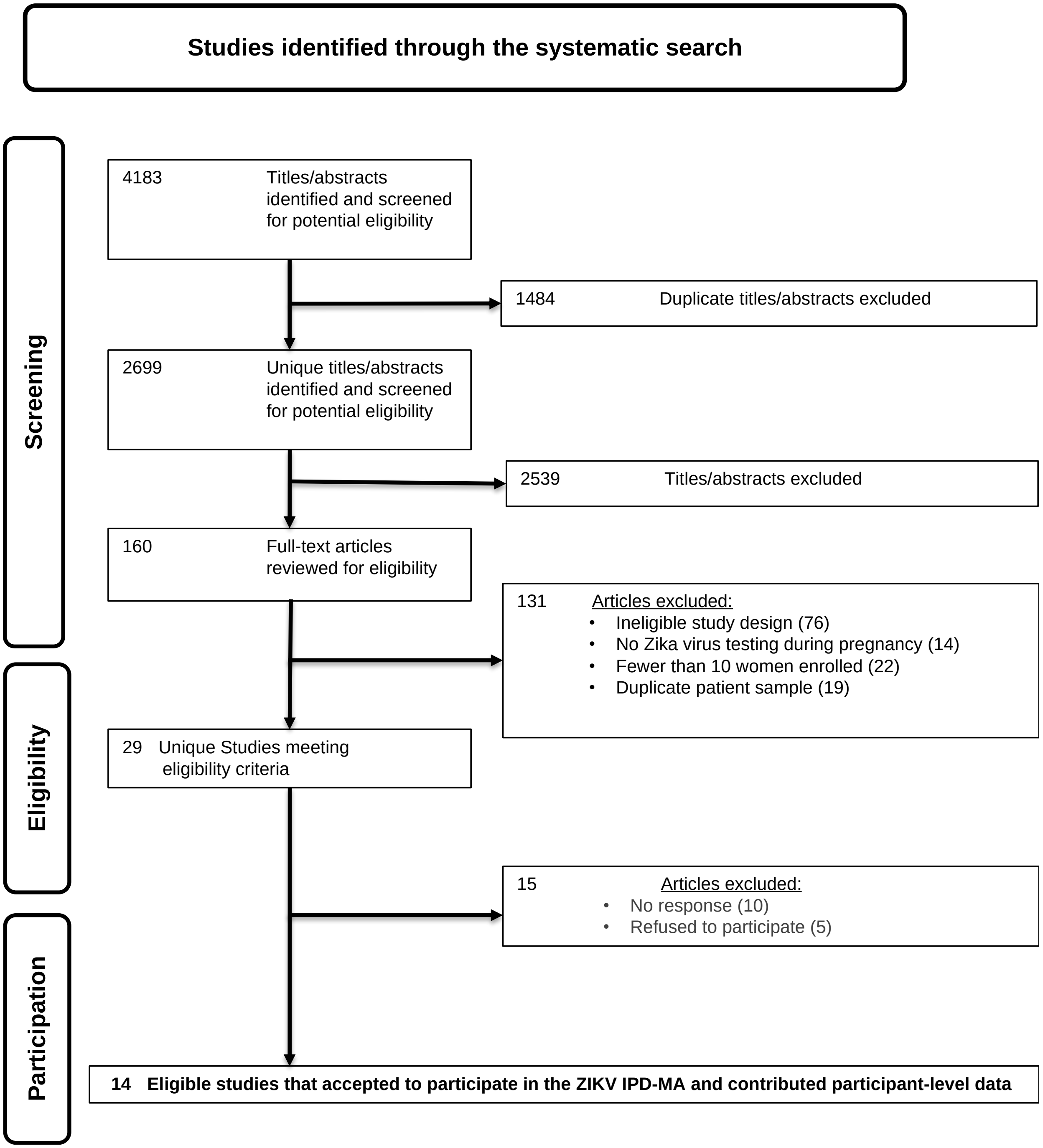

Figure S.1. Flowchart for study participation through systematic search.

For peer review only - http://bmjopen.bmj.com/site/about/guidelines.xhtml

III. SUPPLEMENTARY TABLES WITH DESCRIPTIVE ZIKV-IPD-MA METADA-RELATED STATISITCS AND FIGURES

Table S1. Detailed description of ZIKV-IPD-MA studies participating in the metadata survey (Part 1).

ID	Study Name	Country	Source population	Pregnancy status (Enrollment)	Enrollment and ZIKV status	Study Design	Planned follow-up pregnant women	Planned follow-up child	All Women	ZIKV (+) Women	Children	Funding
1	Bolivia_Roca_ZIKAlliance	Bolivia	Healthcare Facility	Pregnant	Unrelated to ZIKV	Cohort	9 visits conducted every month during pregnancy for both ZIKV+ and ZIKV- women	-	74	15	30	European Union
2	Brazil_Bahia_Ko_NIHNIAD	Brazil	Healthcare Facility	Pregnant	Unrelated to ZIKV	Cohort	9 visits in total for ZIKV+ and ZIKV- pregnant women, every 4 weeks during pregnancy, at delivery, and 6 weeks postpartum. Additional visits were also conducted for women with acute ZIKA-like symptoms.	4 visits at birth, 3, 6, and 12 months of age for infants of both ZIKV+ and ZIKV- pregnant women. The age window for the final visit could be extended up to 18 months.	600	0	509	NIH/NIAID and FIOCRUZ
3	Brazil_BahiaPaudaLima_Costa	Brazil	Community	Pregnant	Unrelated to ZIKV	Cohort	1 visit anytime during pregnancy for both ZIKV+ and ZIKV- women	1 visit anytime between 11-32 months of age for infants of both ZIKV+ and ZIKV- pregnant women	46	9	46	NIH/NIAID, Wellcome Trust, and Bahia State Research Support Foundation (FAPESB)
4	Brazil_BahiaSalvador_Costa	Brazil	Healthcare Facility	Delivered	Unrelated to ZIKV	Cohort	- (enrolled delivered women)	2 visits between 24-36 months of age and then between 30-42 months of age for infants of ZIKV+ and ZIKV- women	157	110	154	NIH/NIAID, Wellcome Trust, and Bahia State Research Support Foundation (FAPESB) ZIKA-FAPESB T.O. no PET0021/2016 (www.fapesb.ba.gov.br) and Coordination for the Improvement of Higher Education (CAPES) from Brazil (https://www.capes.gov.br/)
5	Brazil_BahiaSalvador_Siqueira_czs	Brazil	Healthcare Facility	Children only	Symptoms or presence of exposure	Surveillance	- (enrolled only infants)	One visit during the first week of life for infants of women with ZIKV symptoms during pregnancy	0	0	151	WHO, CNPq (Brazil), and Fapesb (Brazil)
6	Brazil_BahiaSalvador_Siqueira_zikvsymp	Brazil	Healthcare Facility	Pregnant and delivered	Symptoms or presence of exposure	Surveillance	-	One visit during the first week of life for infants of women with ZIKV symptoms during pregnancy	101	73	102	WHO, CNPq (Brazil), and Fapesb (Brazil)
7	Brazil_Brasil_ZIKAlliance	Brazil	Healthcare Facility	Pregnant	Unrelated to ZIKV	Cohort	9 visits conducted every month during pregnancy for both ZIKV+ and ZIKV- women	6 visits at week 4, and months 4, 9, 12, 18, and 24 (final age of folllow-up) for infants of both ZIKV+ and ZIKV- pregnant women	200	-	39	European Union

For peer review only - http://bmjopen.bmj.com/site/about/guidelines.xhtml

8	Brazil_Goias_Turchi	Brazil	Community & Healthcare Facility	Pregnant	Laboratory confirmation/ Symptoms or Presence of Exposure	Cohort	7 visits anytime during prenatal care and after birth for both ZIKV+ and ZIKV- women	6 visits at months 1, 3, 6, 12, 18 (final age of follow-up) for infants of both ZIKV+ and ZIKV- pregnant women	87	58	69	European Union and FAPEG - Fundacion de Amparo a Pesquisa do Estado de Goias
9	Brazil_Maranhao_Silva	Brazil	Healthcare Facility	Children only	Laboratory confirmation/ Symptoms or Presence of Exposure	Cohort	-	Two visits per year, at 6-month intervals, upto 3 years of age (originally planned upto 4 years of age) for infants of ZIKV+ pregnant women	0	0	110	CNPq (Brazilian Research Council), CAPES (Coordination for the Improvement of Higher Education Personnel, FAPEMA (Maranhao State Research Foundation) and Science and Technology Department, Ministry of Health, Brazil
10	Brazil_MatoGrosso_SchulerFaccini	Brazil	Community	Delivered	Laboratory confirmation/ Symptoms or Presence of Exposure	Case cohort	- (enrolled delivered women)	Visits at 6, 12, 18, 24 months and then once a year up to 5 years of age for infants of ZIKV+ pregnant women	50	50	47	National Institute of Population Medical Genetics (INaGeMP)
11	Brazil_Para_Oliveira	Brazil	Community & Healthcare Facility	Pregnant	Laboratory confirmation	Cohort	4 visits every month for ZIKV+ pregnant women. ZIKV- pregnant women not enrolled.	-	134	109	102	-
12	Brazil_Pernambuco_PerezCoelho	Brazil	Community & Healthcare Facility	Children only	Laboratory confirmation/ Symptoms or Presence of Exposure	Cohort	- (enrolled only infants)	-	0	0	130	-
13	Brazil_Pernambuco_Ximenes	Brazil	Community & Healthcare Facility	Pregnant	Laboratory confirmation/ Symptoms or Presence of Exposure	Cohort	3 visits during pregnancy and after a livebirth for ZIKV+ pregnant women. ZIKV- pregnant women not enrolled.	-	700	700	600	European Union, PAHO, NIH/NIAID, Wellcome Trust, CnPq; Capes; Decit; Facepe; Secretaria de Vigilancia em Saude - Ministerio da Saude - Brasil; ERAES; Medical Research Council.
14	Brazil_RdJ_Moreira_ZIP	Brazil	Healthcare Facility	Pregnant	Unrelated to ZIKV	Cohort	6 monthly visits throughout pregnancy for both ZIKV+ and ZIKV- pregnant women	4 visits at birth, 3, 6, and 12 months (final age of follow-up) for both infants of ZIKV+ and ZIKV- pregnant women	771	17	599	NIH/NIAID

For peer review only

15	Brazil_RibieroPrietro_Duarte	Brazil	Community & Healthcare Facility	Pregnant	Laboratory confirmation	Cohort	For ZIKV+ pregnant women, visits were conducted monthly, however the number of visits varied according to the gestational age of inclusion into the study. ZIKV- pregnant women not enrolled.	Visits at birth, and months 1, 3, 6, 12, 18, and 24 (final age of follow-up) for infants of ZIKV+ pregnant women	511	511	487	Mainly supported by Fundacio de Apoio ao Ensino Pesquisa e Assistencia do Hospital das Clinicas da Faculdade de Medicina de Ribeirio Preto da Universidade de Sao Paulo (FAEPA), Brasil, and received partial support from the CVE - Centro de Vigilancia Epidemiologica 'Prof. Alexandre Vranjac' da Secretaria da Saude do Estado de Sao Paulo (SES-SP).
16	Brazil_RiodeJaneiro_Brasil	Brazil	Community	Pregnant	Laboratory confirmation/ Symptoms or Presence of Exposure	Cohort	-	-	400	244	216	Brazilian Ministry of Health (DECIT), NIH and Fondation Merieux
17	Brazil_RiodeJaneiro_Cunha	Brazil	Healthcare Facility	Reproductive age	Laboratory confirmation/ Symptoms or Presence of Exposure	Cohort	8 visits, one per month during pregnancy, for both ZIKV+ and ZIKV- women	11 visits at months 1-6, 12, 18, 24, 30, 36 (final age of follow-up) for infants of ZIKV+ pregnant women. Infants of ZIKV- pregnant women were not followed.	118	118	54	FAPERJ
18	Brazil_RiodeJaneiro_Hofer	Brazil	Healthcare Facility	Pregnant	Laboratory confirmation/ Symptoms or Presence of Exposure	Cohort	1 visit every trimester for both ZIKV+ and ZIKV- women	12 visits at months 1-6, 8, 10, 12, 18, 24 for infants of both ZIKV+ and ZIKV- pregnant women	51	38	43	FAPERJ
19	Brazil_RiodeJaneiro_Joao	Brazil	Community	Pregnant	Laboratory confirmation	Cohort	6 prenatal visits for both ZIKV+ and ZIKV- pregnant women	1 visit at 6 months of age for infants of both ZIKV+ and ZIKV- pregnant women	219	34	221	No funding
20	Brazil_RiodeJaneiro_Moreira	Brazil	Healthcare Facility	Pregnant	Laboratory confirmation/ Symptoms or Presence of Exposure	Cohort	8 visits conducted every month for ZIKV+ pregnant women (also depends on time of ZIKV infection during pregnancy). 8 visits also conducted every month for ZIKV- pregnant women.	8 visits in total each at 3, 6, 9, 12, 18, 24, 30 and 36 months of age (excluding visit at birth). Further follow-up is planned from 36-60 months.	294	294	296	European Union, Wellcome Trust, CNPq and Faperj and PIP/Fiocruz (Brazilian governamental agencies)
21	Brazil_SaoPaulo_DuartePassos	Brazil	Healthcare Facility	Pregnant	Unrelated to ZIKV	Cohort	Unspecified number of visits* *According to medical judgement	Monthly up to 6 months of age; every 3 months up to 12 months of age; every 6 months up to 3 years of age	690	57	696	Brazilian Ministry of Health, London School of Hygiene and Tropical Medicine and FAPESP
22	Brazil_SaoPaulo_LacerdaNoguiera	Brazil	Healthcare Facility	Pregnant	Laboratory confirmation	Cohort	1 visit at enrollment and then 1 visit every trimester for ZIKV+ women. ZIKV- pregnant women not enrolled	-	54	54	54	FAPESP

For peer review only

23	Brazil_Segurado_ZIKAlliance	Brazil	Healthcare Facility	Pregnant	Unrelated to ZIKV	Cohort	9 visits conducted every month during pregnancy for both ZIKV+ and ZIKV- women	6 visits at week 4, and months 4, 9, 12, 18, and 24 (final age of follow-up) for infants of both ZIKV+ and ZIKV- pregnant women	106	0	48	European Union
24	Brazil_SP_Mussi_ZIP	Brazil	Community & Healthcare Facility	Pregnant	Unrelated to ZIKV	Cohort	On average 12 visits before delivery, 1 visit at delivery and 1 visit 6 weeks after delivery for both ZIKV+ and ZIKV- women	4 visits at birth, 3, 6, and 12 months (final age of follow-up) for both infants of ZIKV+ and ZIKV- pregnant women	554	0	418	NIH/NIAID
25	Colombia_Arias_ZIP	Colombia	Healthcare Facility	Pregnant	Unrelated to ZIKV	Cohort	9 visits at enrollment, monthly thereafter, and six weeks post partum for both ZIKV+ and ZIKV- women	4 visits at birth, 3-5 months, 6-11 months, 12-18 months for both infants of ZIKV+ and ZIKV- pregnant women	435	3	206	NIH/NIAID
26	Colombia_Becerra	Colombia	Healthcare Facility	Pregnant	Laboratory confirmation/ Symptoms or Presence of Exposure	Cohort	Monthly visits throughout pregnancy for both ZIKV+ and ZIKV- pregnant women	4 visits at months 6, 12, 18, 24 (final age of follow-up), for infants of both ZIKV+ and ZIKV- pregnant women	243	124	226	COLCIENCIAS Universidad Industrial De Santander Ascon Hospital Universitario De Santander
27	Colombia_Gilboa	Colombia	Healthcare Facility	Pregnant	Unrelated to ZIKV	Cohort	Both ZIKV+ and ZIKV- women had 14-16 visits if they were enrolled early in the first trimester and had a full-term pregnancy. If not, the visits were fewer. There was also one postpartum visit.	Some infants were only followed up until 6 months of age with visits at delivery, 10 days of age, and thereafter every two weeks. Other infants were followed up until 18 months with additional visits at 9, 12, and 18 months of age	1519	16	1239	US CDC, USAID, and Instituto Nacional de Salud (Colombia)
28	Colombia_Lopez Medina	Colombia	Community	Pregnant	Laboratory confirmation	Cohort	- (only followed up with infants)	Visits every 3-4 months until 18 months (final age of follow-up)	170	170	154	Thrasher Foundation
29	Colombia_Mattar	Colombia	Community	Delivered	Symptoms or presence of exposure	Surveillance	8 visits during every trimester for both ZIKV+ and ZIKV- women	4 visits at 6, 12, 18, 24 months (final age of follow-up) for infants of both ZIKV+ and ZIKV- pregnant women	11	11	11	WHO
30	Colombia_MercadoReyes	Colombia	Healthcare Facility	Pregnant	Laboratory confirmation/ Symptoms or Presence of Exposure	Surveillance	14 visits across pregnancy for both ZIKV+ and ZIKV- women	Study did not dictate number of follow-ups, but data that were available in medical records were abstracted up to 2 years of age for infants of both ZIKV+ and ZIKv- pregnant women	1218	253	1185	US CDC
31	Colombia_Mulkey	Colombia	Community + Travelers	Pregnant	Symptoms or presence of exposure	Cohort	2 visits if the ZIKV+ pregnant woman is enrolled prior to 32 weeks of gestation. ZIKV- pregnant women not enrolled	2 visits at 6 months and between 12-18 months for infants of ZIKV+ pregnant women	82	82	80	Ikaria Fund; Thrasher Research Fund; NIH

For peer review only - http://bmjopen.bmj.com/site/about/guidelines.xhtml

32	Colombia_SanzCortes	Colombia	Healthcare Facility	Pregnant	Laboratory confirmation/ Symptoms or Presence of Exposure	Cohort	An average of 5 monthly visits for ZIKV+ pregnant women. ZIKV- pregnant women not enrolled	An average of 5 monthly visits for infants of ZIKV+ pregnant women	17	17	17	No funding
33	Colombia_Villar_ZIKAlliance	Colombia	Healthcare Facility	Pregnant	Unrelated to ZIKV	Cohort	9 visits conducted every month during pregnancy for both ZIKV+ and ZIKV- women	6 visits at week 4, and months 4, 9, 12, 18, and 24 (final age of follow-up) for infants of both ZIKV+ and ZIKV- pregnant women	590	20	473	European Union
34	Ecuador_Soria_ZIKAlliance	Ecuador	Healthcare Facility	Pregnant	Unrelated to ZIKV	Cohort	9 visits conducted every month during pregnancy for both ZIKV+ and ZIKV- women	6 visits at week 4, and months 4, 9, 12, 18, and 24 (final age of folllow-up) for infants of both ZIKV+ and ZIKV- pregnant women	436	436	151	European Union
35	FrenchGuiana_Pomar	France	Healthcare Facility	Pregnant	Laboratory confirmation	Cohort	Monthly visits were conducted after inclusion for both ZIKV+ and ZIKV- pregnant women, capped at a maximum of 6 visits, as inclusion occured mainly after the results of the first serology performed during the first trimester	1 visit at 3 days post-partum for infants of both ZIKV+ and ZIKV- pregnant women	700	301	305	-
36	Grenada_LaBeaud	Grenada	Healthcare Facility	Pregnant and delivered	Symptoms or presence of exposure	Cohort	One prenatal and one postnatal visit for both ZIKV+ and ZIKV- women	Variable number of visits at variable ages upto a maximum of 30 months of age for both infants of ZIKV+ and ZIKV- pregnant women	384	113	388	NIH/NIAID, USAID, National Institutes of Health Eunice Kennedy Shriver, National Institute of Child Health and Human Development, Windward Islands Research and Education Foundation, Stanford Maternal Child Health Research Institute
37	Guadaloupe_Cabie	France	Healthcare Facility	Pregnant	Laboratory confirmation/ Symptoms or Presence of Exposure	Cohort	8 visits in total, one every month during pregnancy, for ZIKV+ women<br>4 visits in total, one every trimester and one at delivery, for ZIKV- women	5 visits at 2, 4, 9, 18, 24 (final age of follow-up) months for infants of both ZIKV+ and ZIKV- pregnant women	4959	1564	1180	French Ministry of Health
38	Guatemala_EspinosaBode_ZINC	Guatemala	Healthcare Facility	Pregnant	Unrelated to ZIKV	Cohort	15 visits at recruitment (if <20 weeks), 26, and 32 weeks, as well as at birth	2 visits at birth and at 3 months (final age of follow-up) for both infants of ZIKV+ and ZIKV- pregnant women	436	0	389	US CDC

For peer review only - http://bmjopen.bmj.com/site/about/guidelines.xhtml

39	Guatemala_Figueroa_ZIP	Guatemala	Community & Healthcare Facility	Pregnant	Unrelated to ZIKV	Cohort	10 monthly visits across pregnancy for both ZIKV+ and ZIKV- women	4 visits at birth, 3, 6, and 12 months (final age of follow-up) for both infants of ZIKV+ and ZIKV- pregnant women	998	0	722	NIH/NIAID
40	HaitiJamaica_ZIKAction	Haiti and Jamaica	Healthcare Facility	Pregnant	Unrelated to ZIKV	Cohort	4 visits at enrollment, 20, 28, and 36 weeks for both ZIKV+ and ZIKV- pregnant women. Extra visits were conducted if the woman was ill.	6 visits at 1, 4, 9, 12, 18, 24 (final age of follow-up) months for infants of both ZIKV+ and ZIKV- pregnant women	1147	-	-	European Union
41	Honduras_Alger	Honduras	Healthcare Facility	Pregnant	Unrelated to ZIKV	Cohort	1 visit upon enrollment for both ZIKV+ and ZIKV- pregnant women	1 visit for infants of both ZIKV+ and ZIKV- pregnant women	3995	7	645	US CDC and School of Public Health and Tropical Medicine, Tulane University
42	Kenya_Widdowson	Kenya	Healthcare Facility	Pregnant	Symptoms or presence of exposure	Cohort	10 monthly visits for ZIKV+ and ZIKV- pregnant women	-	2312	3	0	US CDC
43	Mexico_Alpuche_ZIKAlliance	Mexico	Healthcare Facility	Pregnant	Unrelated to ZIKV	Cohort	7 visits conducted every month during pregnancy for both ZIKV+ and ZIKV- women	6 visits at week 4, and months 4, 9, 12, 18, and 24 (final age of folllow-up) for infants of both ZIKV+ and ZIKV- pregnant women	-	-	-	European Union
44	Mexico_Borja_ZIKAlliance	Mexico	Healthcare Facility	Pregnant	Unrelated to ZIKV	Cohort	8 visits at 6-8, 10-13, 16-18, 22, 28, 32, 36, and 38-41 weeks for both ZIKV+ and ZIKV- pregnant women	4 visits at week 4, and months 4, 12, and 24 (final age of follow-up) for infants of both ZIKV+ and ZIKV- pregnant women	1144	63	657	European Union
45	Nicaragua_Balmaseda_ZIP	Nicaragua	Healthcare Facility	Pregnant	Unrelated to ZIKV	Cohort	On average 10 visits every month during pregnancy, 1 at delivery and 1 visit 6 weeks after delivery for both ZIKV+ and ZIKV- women	4 visits at birth, 3, 6, and 12 months (final age of follow-up) for both infants of ZIKV+ and ZIKV- pregnant women	722	0	-	NIH/NIAID
46	Peru_Gotuzzo_ZIKAlliance	Peru	Healthcare Facility	Pregnant	Unrelated to ZIKV	Cohort	9 visits conducted every month during pregnancy for both ZIKV+ and ZIKV- women	6 visits at week 4, and months 4, 9, 12, 18, and 24 (final age of folllow-up) for infants of both ZIKV+ and ZIKV- pregnant women	153	83	153	European Union
47	PuertoRico_ValenciaPrado_ZAPSS	Puerto Rico	Community & Healthcare Facility	Pregnant and delivered	Laboratory confirmation	Surveillance	-	-	4280	4280	4014	US CDC
48	Singapore_Chan	Singapore	Healthcare Facility	Pregnant	Laboratory confirmation/ Symptoms or Presence of Exposure	Cohort	7 visits every 4-6 weeks during pregnancy for ZIKV+ women. ZIKV- pregnant women were not enrolled.	-	15	15	18	Ministry of Health, Singapore KK Women's and Children's Hospital

For peer review only

49	Spain_Bardaji	Spain	Healthcare Facility	Pregnant	Laboratory confirmation/ Symptoms or Presence of Exposure	Surveil lance	Monthly follow-ups for ZIKV+ women throughout pregnancy. ZIKV- women were also followed monthly until a negative diganosis, after which they were referred to the closest healthcare centre for follow-up	7 visits at birth, and at months 1, 2, 6, 12, 18 and 24 (final age of follow up) for children of ZIKV+ pregnant women. Children of ZIKV- pregnant women were not followed	195	44	42	The Government of Spain, Fondo de Investigación en Salud (FIS), Instituto de Salud Carlos III.
50	Spain_Soriano	Spain	Travelers	Pregnant	Symptoms or presence of exposure	Cohort	At lesst one visit per trimester for ZIKV+ pregnant women. ZIKV- pregnant women not enrolled.	Visits scheduled at 1, 4, 9, 12, 18, 24 and 36 months of age for children of ZIKV+ pregnant women	186	186	213	European Union
51	Suriname_Juliana	Suriname	Healthcare Facility	Delivered	Laboratory confirmation/ Symptoms or Presence of Exposure	Surveil lance	2 visits for ZIKV+ women with one during pregnacy and another after. ZIKV- pregnant women were not enrolled	Visits at 3, 6, 9, and 12 months for infants with evidence of congential ZIKV syndrome. After this, children seen on an as-needed basis. Another complete assessment was conducted at 4 years of age	22	16	22	No funding
52	TrinidadTobago_ Sohan	Trinidad and Tobago	Healthcare Facility	Pregnant	Laboratory confirmation	Cohort	12 visits that occur every month until 26 weeks and then every 3 weeks until 36 weeks for ZIKV+ pregnant women. More visits can occur if abnormalities are detected<br>2 visits that occur during the trimester and then during the second or third trimester for ZIKV- pregnant women	-	100	100	0	-
53	USA_Mulkey	USA	Healthcare Facility	Pregnant	Symptoms or presence of exposure	Cohort	1 visit for ZIKV+ and ZIKV- pregnant women usually after 18 weeks of gestation	1-5 visits at birth and 3, 6, 12, and 18 months	72	29	42	Ikaria Fund; Thrasher Research Fund; NIH
54	USA_USZPIR	USA	Community + Travelers	Pregnant and delivered	Laboratory confirmation	Surveil lance	Medical records used for ZIKV+ pregnant women. Maternal data was collected at time of identification and then each trimester following. ZIKV- pregnant women not enrolled	Data on infants collected at birth and then at 2, 6, 12, 18, 24, 30, and 36 months	603	603	528	US CDC

ZIKV: Zika Virus; DENV: Dengue Virus; CHIKV: Chikungunya Virus; ZIKV(+): ZIKV positive individuals;

Note: Study Name convention: Country_City/PIName_StudyConsortium (if multisite)

For peer review only - http://bmjopen.bmj.com/site/about/guidelines.xhtml

Table S1. Detailed description of ZIKV-IPD-MA studies participating in the metadata survey (Part 2).

ID	Study Name	Maternal ZIKV assessment	Fetal ZIKV assessment	Infant ZIKV assessment	Maternal PCR	Maternal PRNT	Fetal PCR	Prenatal microcephaly definition	Postnatal microcephaly assessment	Identification of CZS malformations	CZS Definition	DENV testing	CHIKV Testing
1	Bolivia_Roca_ZIK Alliance	Laboratory testing	Maternal clinical/laboratory criteria, fetal laboratory criteria	Infant clinical/laboratory criteria	Yes	No	Yes	-	No*	Peri/intrapartum and postpartum	WHO	Yes	Yes
2	Brazil_Bahia_Ko_NIHNIAD	Laboratory testing	-	Infant clinical/laboratory, imaging, and maternal clinical/laboratory criteria	Yes	No	No	2 SD below the mean	Yes	Antenatal and peri/intrapartum and postpartum	WHO	Yes	Yes
3	Brazil_BahiaPaudaLima_Costa	Laboratory testing	-	-	No	No	No	-	Yes	Postpartum	Study definition	No	No
4	Brazil_BahiaSalvador_Costa	Laboratory testing	-	Infant clinical/laboratory, imaging and maternal clinical/laboratory criteria	No	Yes	No	-	Yes	Postpartum	Study definition	No	No
5	Brazil_BahiaSalvador_Siqueira_czs	-	Placental laboratory criteria	Infant clinical/laboratory, imaging and maternal clinical/laboratory criteria	No	No	No	2 SD below the mean	Yes	Postpartum	Country	No	No
6	Brazil_BahiaSalvador_Siqueira_zikvsymp	Clinical case definition and/or Laboratory confirmation	Placental laboratory criteria	Infant clinical/laboratory, imaging and maternal clinical/laboratory criteria	No	No	No	2 SD below the mean	Yes	Postpartum	Country	Yes	Yes
7	Brazil_Brasil_ZIK Alliance	Laboratory testing	-	Infant clinical/laboratory, imaging and maternal clinical/laboratory criteria	Yes	Yes	No	2 SD below the mean	No*	-	-	Yes	Yes
8	Brazil_Goias_Turchi	Laboratory testing	Fetal imaging criteria	Infant clinical/laboratory criteria	Yes	No	No	2 SD below the mean	Yes	Postpartum	Country	Yes	Yes
9	Brazil_Maranhao_Silva	Clinical case definition	-	Infant clinical/laboratory, imaging and maternal clinical/laboratory criteria	No	No	No	-	Yes	Postpartum	Study definition	No	No
10	Brazil_MatoGrosso_SchulerFaccini	Clinical case definition and/or Laboratory confirmation	-	-	Yes	No	No	-	Yes	Peri/intrapartum	Country	No	No

For peer review only - http://bmjopen.bmj.com/site/about/guidelines.xhtml
For peer review only

11	Brazil_Para_Oliveira	Laboratory testing	Maternal clinical/laboratory criteria and placental laboratory criteria	Maternal clinical/laboratory criteria	Yes	Yes	No	Below the 5th percentile	Yes	Peri/intrapartum and postpartum	Country	Yes	Yes
12	Brazil_Pernambuco_PerezCoelho	-	-	Infant clinical/laboratory and infant imaging criteria	No	No	No	-	Yes	Postpartum	Country	No	No
13	Brazil_Pernambuco_Ximenes	Laboratory testing	-	Infant clinical/laboratory, imaging and maternal clinical/laboratory criteria	Yes	Yes	No	-	Yes	Peri/intrapartum and postpartum	Study definition	Yes	Yes
14	Brazil_RdJ_Moreira_ZIP	Laboratory testing	Maternal clinical/laboratory, fetal imaging, laboratory and placental criteria	Infant clinical/laboratory, imaging and maternal clinical/laboratory criteria	Yes	No	Yes	2 SD below the mean	Yes	Antenatal and peri/intrapartum and postpartum	WHO	Yes	Yes
15	Brazil_RibieroPrietro_Duarte	Laboratory testing	Fetal imaging, laboratory and placental criteria	Infant clinical/laboratory criteria and maternal clinical/laboratory criteria	Yes	No	Yes	Below the 3rd centile	Yes	Postpartum	Country	No	No
16	Brazil_RiodeJaneiro_Brasil	Laboratory testing	Maternal clinical/laboratory, fetal imaging and laboratory criteria	Infant clinical/laboratory and infant imaging criteria	Yes	No	Yes	3 SD below the mean	Yes	Antenatal and postpartum	Study definition	Yes	Yes
17	Brazil_RiodeJaneiro_Cunha	Clinical case definition and/or Laboratory confirmation	Placental laboratory criteria	Infant clinical/laboratory, imaging and maternal clinical/laboratory criteria	Yes	No	No	2 SD below the mean	Yes	Peri/intrapartum	Country	Yes	Yes
18	Brazil_RiodeJaneiro_Hofer	Laboratory testing	-	Infant clinical/laboratory, imaging and maternal clinical/laboratory criteria	Yes	No	No	2 SD below the mean	Yes	Postpartum	Country	Yes	No
19	Brazil_RiodeJaneiro_Joao	Laboratory testing	-	-	Yes	Yes	No	-	Yes	Antenatal and peri/intrapartum and postpartum	Country	Yes	Yes
20	Brazil_RiodeJaneiro_Moreira	Clinical case definition and/or Laboratory confirmation	Maternal clinical/laboratory, fetal imaging, laboratory and placental criteria	Infant clinical/laboratory, imaging and maternal clinical/laboratory criteria	Yes	No	Yes	3 SD below the mean	Yes	Antenatal and peri/intrapartum and postpartum	WHO	Yes	Yes

For peer review only - http://bmjopen.bmj.com/site/about/guidelines.xhtml

21	Brazil_SaoPaulo_DuartePassos	Laboratory testing	-	Infant clinical/laboratory and infant imaging criteria	Yes	No	No	2 SD below the mean	Yes	Antenatal and postpartum	CDC/WHO /Country	Yes	Yes
22	Brazil_SaoPaulo_LacerdaNoguiera	Laboratory testing	-	Infant clinical/laboratory criteria	Yes	No	No	-	Yes	Antenatal and postpartum	Study definition	Yes	No
23	Brazil_Segurado_ZIKAlliance	Laboratory testing	Maternal clinical/laboratory, fetal imaging, laboratory and placental criteria	-	Yes	No	Yes	3 SD below the mean	Yes	Postpartum	Study definition	No	Yes
24	Brazil_SP_Mussi_ZIP	Laboratory testing	-	-	Yes	No	No	2 SD below the mean	Yes	Antenatal and peri/intrapartum and postpartum	WHO	Yes	Yes
25	Colombia_Arias_ZIP	Laboratory testing	Fetal laboratory criteria	Infant clinical/laboratory criteria	Yes	No	Yes	3 SD below the mean	Yes	-	-	Yes	Yes
26	Colombia_Becerra	Laboratory testing	Maternal clinical/laboratory, fetal imaging, laboratory and placental criteria	Infant clinical/laboratory criteria and maternal clinical/laboratory criteria	Yes	No	Yes	2 SD below the mean	Yes	Antenatal and peri/intrapartum and postpartum	WHO	Yes	No
27	Colombia_Gilboa	Laboratory testing	Fetal laboratory criteria	Infant clinical/laboratory criteria	Yes	No	Yes	Other	Yes	Antenatal and postpartum	CDC	Yes	Yes
28	Colombia_Lopez Medina	Laboratory testing	-	-	Yes	No	No	2 SD below the mean	Yes	-	-	No	No
29	Colombia_Mattar	Laboratory testing	-	-	Yes	No	No	2 SD below the mean	Yes	Antenatal and peri/intrapartum	Country	No	Yes
30	Colombia_MercadoReyes	Clinical case definition and/or Laboratory confirmation	Maternal clinical/laboratory, fetal imaging, laboratory and placental criteria	Infant clinical/laboratory, imaging and maternal clinical/laboratory criteria	Yes	No	No	3 SD below the mean	Yes	Antenatal and peri/intrapartum and postpartum	Study definition	Yes	Yes
31	Colombia_Mulkey	Clinical case definition and/or Laboratory confirmation	-	Maternal clinical/laboratory criteria	Yes	Yes	No	3 SD below the mean	Yes	Antenatal and postpartum	Study definition	Yes	No

For peer review only

32	Colombia_SanzCortes	Laboratory testing	Maternal clinical/laboratory, fetal imaging, laboratory and placental criteria	Infant clinical/laboratory, imaging and maternal clinical/laboratory criteria	Yes	No	Yes	3 SD below the mean	Yes	Antenatal and peri/intrapartum and postpartum	Country	No	No
33	Colombia_Villar_ZIKAlliance	Laboratory testing	-	-	Yes	No	No	-	Yes	Antenatal and peri/intrapartum and postpartum	WHO	Yes	Yes
34	Ecuador_Soria_ZIKAlliance	Laboratory testing	Fetal imaging, laboratory and placental criteria	Infant clinical/laboratory criteria	Yes	No	Yes	2 SD below the mean	Yes	Postpartum	Country	No	No
35	FrenchGuiana_Pomar	Laboratory testing	Fetal imaging, laboratory and placental criteria	Infant clinical/laboratory criteria	Yes	Yes	Yes	2 SD below the mean	No*	Antenatal and postpartum	Study definition	Yes	Yes
36	Grenada_LaBeaud	Laboratory testing	-	Infant clinical/laboratory criteria	Yes	No	No	-	Yes	Postpartum	WHO	Yes	Yes
37	Guadaloupe_Cabie	Laboratory testing	Placental laboratory criteria	Infant clinical/laboratory criteria and maternal clinical/laboratory criteria	Yes	No	No	2 SD below the mean	Yes	Antenatal and peri/intrapartum and postpartum	Study definition	No	No
38	Guatemala_EspinosaBode_ZINC	Clinical case definition and/or Laboratory confirmation	-	Infant clinical/laboratory criteria	Yes	No	No	2 SD below the mean	Yes	Postpartum	CDC	Yes	Yes
39	Guatemala_Figueroa_ZIP	Laboratory testing	Maternal clinical/laboratory criteria	Infant clinical/laboratory criteria	Yes	No	No	2 SD below the mean	Yes	-	-	Yes	No
40	HaitiJamaica_ZIKAction	Laboratory testing	-	Infant clinical/laboratory and infant imaging criteria	Yes	Yes	No	2 SD below the mean	No*	Antenatal and postpartum	Study definition	Yes	Yes
41	Honduras_Alger	Laboratory testing	-	-	Yes	No	No	-	Yes	-	-	Yes	-
42	Kenya_Widdowson	Clinical case definition and/or Laboratory confirmation	Maternal clinical/laboratory, fetal laboratory, and placental criteria	Infant clinical/laboratory criteria	Yes	Yes	Yes	-	No*	Postpartum	Intergrowth	Yes	Yes
43	Mexico_Alpuche_ZIKAlliance	Clinical case definition and/or Laboratory confirmation	-	-	Yes	No	No	-	Yes	Peri/intrapartum and postpartum	-	-	-
44	Mexico_Borja_ZIKAlliance	Laboratory testing	Fetal imaging, laboratory and	Infant clinical/laboratory and infant imaging criteria	Yes	No	Yes	2 SD below the mean	No*	Postpartum	Study definition	Yes	Yes

For peer review only - http://bmjopen.bmj.com/site/about/guidelines.xhtml

For peer review only

			placental criteria										
45	Nicaragua_Balmaseda_ZIP	Laboratory testing	-	-	Yes	No	-	-	-	-	-	-	-
46	Peru_Gotuzzo_ZIKAlliance	Laboratory testing	Fetal imaging, laboratory and placental criteria	Infant clinical/laboratory and infant imaging criteria	Yes	No	Yes	2 SD below the mean	Yes	Peri/intrapartum and postpartum	Country	No	Yes
47	PuertoRico_ValenciaPrado_ZAPSS	Laboratory testing	Placental laboratory criteria	Infant clinical/laboratory criteria	Yes	No	No	-	Yes	Antenatal and peri/intrapartum and postpartum	Study definition	Yes	Yes
48	Singapore_Chan	Laboratory testing	-	Infant clinical/laboratory criteria	Yes	No	No	2 SD below the mean	Yes	Antenatal and postpartum	Country	No	No
49	Spain_Bardaji	Laboratory testing	Maternal clinical/laboratory, fetal imaging, laboratory and placental criteria	Infant clinical/laboratory, imaging and maternal clinical/laboratory criteria	Yes	No	Yes	2 SD below the mean	Yes	Antenatal and peri/intrapartum and postpartum	WHO	Yes	Yes
50	Spain_Soriano	Clinical case definition and/or Laboratory confirmation	Maternal clinical/laboratory, fetal imaging, laboratory and placental criteria	Infant clinical/laboratory and infant imaging criteria	Yes	Yes	Yes	2 SD below the mean	Yes	Antenatal and peri/intrapartum and postpartum	Country	Yes	Yes
51	Suriname_Juliana	Clinical case definition and/or Laboratory confirmation	-	Infant clinical/laboratory, imaging and maternal clinical/laboratory criteria	Yes	No	No	-	Yes	Postpartum	WHO	No	No
52	TrinidadTobago_Sohan	Clinical case definition and/or Laboratory confirmation	-	-	Yes	No	No	Below the 5th percentile	Yes	-	-	Yes	Yes
53	USA_Mulkey	Clinical case definition and/or Laboratory confirmation	-	Maternal clinical/laboratory criteria	Yes	Yes	No	3 SD below the mean	Yes	Antenatal and peri/intrapartum and postpartum	Study definition	Yes	No
54	USA_USZPIR	Laboratory testing	Fetal laboratory and placental criteria	Infant clinical/laboratory criteria	Yes	Yes	Yes	3 SD below the mean	Yes	Antenatal and peri/intrapartum and postpartum	CDC	No	No

ZIKV: Zika Virus; DENV: Dengue Virus; CHIKV: Chikungunya Virus; ZIKV(+): ZIKV positive individuals;

For peer review only

For peer review only - http://bmjopen.bmj.com/site/about/guidelines.xhtml

Table S2. Infant and child development assessment among ZIKV-IPD-MA study participants of the metadata survey.

**Characteristic**	N = 40[1]
**Age of Developmental Assessment (Months)**	
**Median (IQR)**	24 (18, 30)
**Range**	3, 60
**Developmental assessment tool**	
**ASQ-SE Tool**	21 / 40 (52%)
**BSID Tool**	30 / 40 (75%)
**WIDEA-FS Tool**	2 / 39 (5.1%)
**AIMS Tool**	4 / 40 (10%)
**Other Assessments***	18 / 40 (45%)

[1] N= Total studies; Frequency n/N (row) (%);

Bayley Scales of Infant Development (BSID); Ages & Stages Questionnaires® Social-Emotional, Second Edition (ASQ®-SE-2); Warner Initial Developmental Evaluation of Adaptive and Functional Skills (WIDEA) and four the Alberta Infant Motor Scale (AIMS). Note: developmental assessment was only reported by 40 studies, so the denominator is 40 studies. *Includes INTER-NDA and NEPSY-II

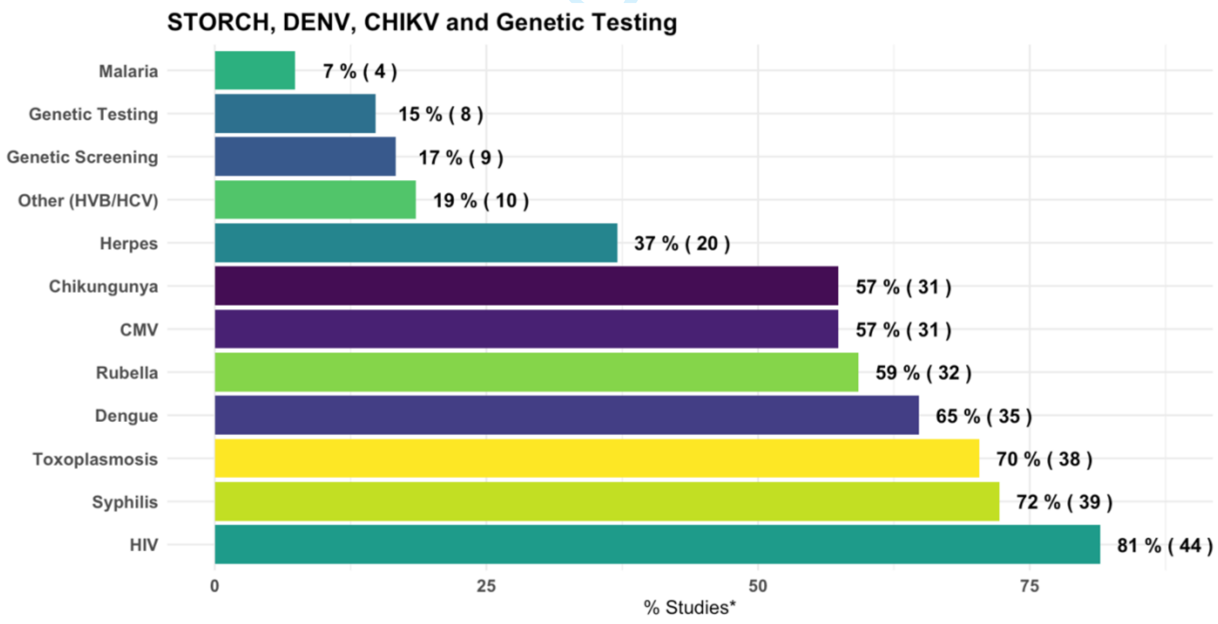

ZIKV: Zika Virus; DENV: Dengue Virus; CHIKV: Chikungunya Virus; HIV: Human Immunodeficiency Virus; CMV: Cytomegalovirus; HVB/HCV: Hepatitis B and Hepatitis C Virus.

Figure S2. STORCH and other additional testing among ZIKV-IPD-MA study participants of the metadata survey. The denominator is total number of studies (n=54)

Table S3. Sociodemographic and behavioral factors measured among ZIKV-IPD-MA study participants of the metadata survey.

**Characteristic**	**N = 52[1]**
**Education**	43 / 52 (83%)
**Occupation**	32 / 52 (62%)
**Marital status**	40 / 52 (77%)
**Nutrition status**	15 / 51 (29%)
**Maternal exposure to workplace or environmental occupational teratogens**	17 / 51 (33%)
**Pre-pregnancy exposure to IPV**	4 / 52 (7.7%)
**Maternal exposure to IPV**	9 / 51 (18%)
**Infant/child exposure to IPV**	7 / 52 (13%)
**Maternal exposure to vaccination**	33 / 51 (65%)
**Maternal prescription drug use**	40 / 51 (78%)
**Maternal recreational drug use**	42 / 51 (82%)
**Maternal ethnic or minority status**	35 / 52 (67%)
**Household SES**	37 / 52 (71%)
**Mental health scales**	4 / 51 (7.8%)
**Maternal smoking**	42 / 51 (82%)
**Maternal alcohol consumption**	41 / 51 (80%)

[1] N= Total studies; Frequency n/N (row) (%)

Note: There were Two studies that did not provide any information about the sociodemographic metadata.

For peer review only - http://bmjopen.bmj.com/site/about/guidelines.xhtml

Table S4. Genetic testing, fetal ultrasounds, and MRIs among ZIKV-IPD-MA study participants of the metadata survey

**Characteristic**	**Overall N = 53**[1]	**Other Countries N = 30**[1]	**Brazil N = 23**[1]
**Genetic Screening**	9 / 53 (17%)	7 / 30 (23%)	2 / 23 (8.7%)
**Genetic Testing**	8 / 52 (15%)	6 / 29 (21%)	2 / 23 (8.7%)
**Fetal Ultrasounds**	39 / 53 (74%)	24 / 30 (80%)	15 / 23 (65%)
**Fetal Ultrasound Timing**			
Monthly	7 / 39 (18%)	6 / 24 (25%)	1 / 15 (6.7%)
Once per Trimester	17 / 39 (44%)	6 / 24 (25%)	11 / 15 (73%)
2nd & 3rd Trimester	3 / 39 (7.7%)	3 / 24 (12%)	0 / 15 (0%)
Twice per Pregnancy	3 / 39 (7.7%)	2 / 24 (8.3%)	1 / 15 (6.7%)
Unspecified Timing	9 / 39 (23%)	7 / 24 (29%)	2 / 15 (13%)
**Number of Ultrasounds per Woman**			
At least 3	25 / 39 (64%)	13 / 24 (54%)	12 / 15 (80%)
4 to 6	5 / 39 (13%)	3 / 24 (12%)	2 / 15 (13%)
More than 7	3 / 39 (7.7%)	3 / 24 (12%)	0 / 15 (0%)
Unspecified Number	6 / 39 (15%)	5 / 24 (21%)	1 / 15 (6.7%)
**Postnatal Ultrasound**	36 / 53 (68%)	23 / 30 (77%)	13 / 23 (57%)
**Fetal MRIs**	10 / 52 (19%)	7 / 29 (24%)	3 / 23 (13%)
**Postnatal MRI**	17 / 52 (33%)	9 / 29 (31%)	8 / 23 (35%)

[1] Frequency n/(N row) (%)

For peer review only - http://bmjopen.bmj.com/site/about/guidelines.xhtml

Table S5a. Type of maternal ZIKV assessment among ZIKV-IPD-MA study participants of the metadata survey.

Characteristic	N = 51[1]	Characteristic	N = 51[1]
**Type of Maternal ZIKV Assessment**		**Performed PRNT**	12 / 51 (24%)
Clinical and Laboratory Criteria	13 / 51 (25%)	**PRNT %**	
Laboratory Criteria	38 / 51 (75%)	PRNT50	2 / 12 (17%)
**Performed PCR**	48 / 51 (94%)	PRNT90	6 / 12 (50%)
**PCR Type**		Unknown	4 / 12 (33%)
Conventional PCR	5 / 48 (10%)	**PRNT Cut-off Titers**	
qRT-PCR	15 / 48 (31%)	PRNT>20	5 / 12 (42%)
RT-PCR	27 / 48 (56%)	PRNT≥10	5 / 12 (42%)
Unknown	1 / 48 (2.1%)	Unknown	2 / 12 (17%)
**Performed QC for PCR**		**Performed DENV/ZIKV PRNT**	
No	5 / 48 (10%)	No	8 / 13 (62%)
Unknown	8 / 48 (17%)	Unknown	3 / 13 (23%)
Yes	35 / 48 (73%)	Yes	2 / 13 (15%)
**Performed Immunoassays**	35 / 51 (69%)	**Performed QC for PRNT**	
**Performed ELISA IgM**	34 / 51 (67%)	No	10 / 12 (83%)
**Performed ELISA IgG**	18 / 51 (35%)	Unknown	1 / 12 (8.3%)
**Performed QC for ELISAs**		Yes	1 / 12 (8.3%)
No	1 / 35 (2.9%)		
Unknown	8 / 35 (23%)		
Yes	26 / 35 (74%)		

[1] Frequency n/(N row) (%)

Table S5b. Type of fetal ZIKV assessment among ZIKV-IPD-MA study participants of the metadata survey.

**Characteristic**	**N = 27**[1]
**Type of Fetal ZIKV Assessment**	
Fetal Imaging Criteria	1 / 27 (3.7%)
Fetal Laboratory Criteria	2 / 27 (7.4%)
Maternal & Fetal Laboratory Criteria	7 / 27 (26%)
Maternal & Fetal Laboratory Criteria, including Imaging	11 / 27 (41%)
Maternal Laboratory Criteria	6 / 27 (22%)
**Performed PCR**	18 / 27 (67%)
**PCR Type**	
Conventional PCR	1 / 18 (5.6%)
qRT-PCR	8 / 18 (44%)
RT-PCR	9 / 18 (50%)
**Performed QC for PCR**	
No	3 / 18 (17%)
Unknown	2 / 18 (11%)
Yes	13 / 18 (72%)
**Performed ELISA IgM**	8 / 27 (30%)
**Performed ELISA IgG**	4 / 27 (15%)
**Performed QC for ELISAs**	
No	1 / 9 (11%)
Unknown	1 / 9 (11%)
Yes	7 / 9 (78%)
**Performed PRNT**	1 / 27 (3.7%)

[1] Frequency n/(N row) (%)

Table S5c. Type of infant ZIKV assessment among ZIKV-IPD-MA study participants of the metadata survey.

**Characteristic**	**N = 41**[1]
**Type of Infant ZIKV Assessment**	
Infant Laboratory & Imaging Criteria	7 / 41(17%)
Infant Laboratory Criteria	14 / 41 (34%)
Maternal & Infant Laboratory Criteria	3 / 41 (7%)
Maternal & Infant Laboratory Criteria, including Imaging	15 / 41 (37%)
Maternal Clinical/Laboratory Criteria	2 / 41 (5%)
**Performed PCR**	33 / 41 (80%)
**PCR Type**	
Conventional PCR	5 / 33 (15%)
qRT-PCR	13 / 33 (39%)
RT-PCR	13 / 33 (39%)
Unknown	2 / 33 (6.1%)
**Performed QC for PCR**	
No	5 / 33 (15%)
Unknown	7 / 33 (21%)
Yes	21 / 33 (64%)
**Performed ELISA IgM**	24 / 41 (59%)
**Performed ELISA IgG**	14 / 41 (34%)
**Performed QC for ELISAs**	
No	2 / 25 (8.0%)
Unknown	5 / 25 (20%)
Yes	18 / 25 (72%)
**Performed PRNT**	5 / 41 (12%)

[1] Frequency n/(N row) (%)

ZIKV IPD-MA Metadata survey

The metadata survey collects information from investigators about how their study or site measured ZIKV and DENV infection, how positive, negative, and indeterminate ZIKV tests were classified, and information on the measurement of confounders and outcomes of interest. These data will be used to appropriately account for study-level heterogeneity and measurement error in the statistical models developed for ZIKV IPD-MA Objectives 1-4.

The metadata survey may take up to one hour to complete and may require input from different members of your study team. Please contact Lauren Maxwell at maxwelll@who.int if you have questions while completing the metadata survey.

El mismo mensaje en español

La encuesta de metadatos recopila información de los investigadores sobre cómo su estudio o sitio midió la infección por ZIKV y DENV, cómo se clasificaron las pruebas de ZIKV positivas, negativas e indeterminadas, e información sobre la medición de factores de confusión y resultados de interés. Estos datos se utilizarán para explicar adecuadamente la heterogeneidad a nivel de estudio y el margin de error en los modelos estadísticos desarrollados para ZIKV IPD-MA Objetivos 1-4.

La encuesta de metadatos puede tardar hasta una hora en completarse y requerirá aportes de diferentes miembros de su equipo de investigacion. Por favor, póngase en contacto con Lauren Maxwell at maxwelll@who.int si tiene preguntas mientras completa la encuesta de metadatos.

A mesma mensagem em português

A pesquisa de metadados coleta informações de pesquisadores sobre como o estudo ou local mediu a infecção por ZIKV e DENV, como foram classificados os testes positivos, negativos e indeterminados de ZIKV e informações sobre como medir fatores de confusão e resultados de interesse. Esses dados serão utilizados para explicar adequadamente a heterogeneidade no nível do estudo e a margem de erro nos modelos estatísticos desenvolvidos para os objetivos 1-4 do ZIKV IPD-MA.

A pesquisa de metadados pode levar até uma hora para ser concluída e exigirá informações de diferentes membros da sua equipe de pesquisa. Entre em contato com Lauren Maxwell em maxwelll@who.int, se tiver dúvidas ao concluir a pesquisa de metadados.

Basic study information

Your first name ________________________

Your last name ________________________

Study name ______________________________

For peer review only

Study country	◯ Brazil<br>◯ Colombia<br>◯ Non-continental US<br>◯ Continental US<br>◯ Mexico<br>◯ Honduras<br>◯ Spain<br>◯ Thailand<br>◯ Nicaragua<br>◯ Philippines<br>◯ France<br>◯ Guyana<br>◯ Suriname<br>◯ Greece<br>◯ Grenada<br>◯ Italy<br>◯ UK<br>◯ Trinidad and Tobago<br>◯ Jamaica<br>◯ Singapore<br>◯ Kenya<br>◯ Other<br>◯ Multi-country study
For multi-country studies, specify the included countries	______________________
Specify other country	______________________
Study state	______________________<br>(Write N/A if you have a country-level study)
Study city	______________________<br>(Write N/A if you have a state- or country-level study)
Number of study sites	______________________
Provide city and state for each site, if relevant<br>Use a semi-colon to separate sites and a comma to separate the city and state<br><br>e.g. New York City, New York; San Francisco, California	______________________
Does your study agree, in principle, to share de-identified subject-level data for the ZIKV IPD Consortium IPD-MA?	◯ Yes<br>◯ No<br>◯ Considering
Study Type	◯ Cohort<br>◯ Active surveillance system<br>◯ Case cohort<br>◯ Other

For peer review only

Specify other study type	__________________
What is the source population for your study?<br><br>Select all that apply	☐ Hospital or health care center<br>☐ Community<br>☐ Travelers<br>☐ Other
What are the other source population(s) for your study?	__________________
Was your study funded by any of the following groups?<br><br>Select all that apply	☐ European Union<br>☐ GlOPID-R<br>☐ WHO<br>☐ PAHO<br>☐ Africa CDC<br>☐ E-CDC<br>☐ US-CDC<br>☐ Bill and Melinda Gates Foundation<br>☐ NIH/NIAID<br>☐ Wellcome Trust<br>☐ DfID<br>☐ USAID<br>☐ DFG<br>☐ Other<br>☐ Study did not receive funding from any of these groups
Which other group funded your study?	__________________
When did your study begin enrolling women?	__________________
When did your study finish enrolling women?	__________________<br>(Specify the date you estimate you will stop enrolling women if enrollment is ongoing)
How many women did your study PLAN to enroll?	__________________
How many women has your study ACTUALLY enrolled to date?	__________________
How many ZIKV+ pregnant women has your study enrolled to date?	__________________
How many infants or children did you study include?	__________________
For multi-site studies, have all sites applied the same laboratory and clinical protocols?	◯ Same laboratory protocol<br>◯ Same clinical protocol<br>◯ Same laboratory & clinical protocols<br>◯ Sites apply different laboratory and clinical protocols

For peer review only

Describe cross-site differences in laboratory protocols

Describe cross-site differences in clinical protocols

For peer review only

Enrollment criteria & recruitment methods

Describe the inclusion criteria for the cohort

____

Describe the exclusion criteria for the cohort

____

Describe the inclusion criteria for the cohort from which the case-cohort was sampled

____

Describe the exclusion criteria for the cohort from which the case-cohort was sampled

____

What was the case definition used to identify cases for the case-cohort study?

____

How were women sampled from the cohort to participate in the case-cohort study?

____

Which groups were recruited to the study?

- ○ pregnant women
- ○ recently delivered women with infants with evidence of ZIKV infection
- ○ recently delivered women, regardless of their infants' ZIKV status
- ○ women of reproductive age, regardless of their pregnancy status
- ○ other

Which other groups were recruited to the study?

____

How was the pregnancy identified?

Select all that apply

- ☐ Self-reported
- ☐ 1st obstetric visit (blood or urine test)
- ☐ Urine test (self-administered at home)
- ☐ Urine test (at health care facility)
- ☐ Blood test
- ☐ Ultrasound
- ☐ Varies at the participant level

How was gestational age determined?

Select all that apply

- ☐ LMP (last menstrual period)
- ☐ US (ultrasound)

Was ZIKV the original disease of interest for your cohort?

- ○ Yes
- ○ No, was originally a DENV study
- ○ No, was originally an influenza study
- ○ No, was formed for another purpose

What was the initial purpose of your cohort?

____

For peer review only

If your study wasn't originally designed to measure ZIKV, which symptoms were used to decide whether to test women for ZIKV?<br><br>Select all that apply	☐ Fever<br>☐ Rash<br>☐ Joint pain<br>☐ Conjunctivitis<br>☐ Rash OR joint pain OR conjunctivitis OR fever<br>☐ Other<br>☐ Symptoms not required for testing
What other symptoms were used to decide whether to test women for ZIKV?	__________________________________
How was enrollment of women or infants into the study related to their ZIKV status?<br><br>Select all that apply	☐ women's ZIKV assay<br>☐ women's ZIKV symptoms<br>☐ women's ZIKV assay OR ZIKV symptoms<br>☐ infant's ZIKV assay<br>☐ infant's ZIKV symptoms<br>☐ infant's ZIKV assay OR ZIKV symptoms<br>☐ women's presumed exposure (travel or sexual)<br>☐ other<br>☐ enrollment not related to ZIKV status of women or infants
In what other way was enrollment of women or infants into the study related to their ZIKV status?	__________________________________
How was study enrollment of women related to their ZIKV assay result or their infant's ZIKV assay result?	○ Study only enrolled ZIKV PCR pos (+) pregnant women<br>○ Study only enrolled ZIKV PCR pos (+) or probable pregnant women<br>○ Study only enrolled ZIKV PCR neg (-) pregnant women<br>○ Study only enrolled women with ZIKV PCR pos (+) infants, irregardless of women's ZIKV infection status<br>○ Study only enrolled women with ZIKV PCR pos (+) or ZIKV probable infants, irregardless of women's ZIKV infection status<br>○ Study only enrolled recently delivered ZIKV PCR pos (+) women with ZIKV PCR pos (+) or ZIKV probable infants<br>○ Other
Describe other ZIKV assay result-related inclusion criteria	__________________________________
What ZIKV-related symptoms did your study consider when enrolling women?	○ Fever only<br>○ Rash only<br>○ Fever + rash<br>○ Fever or rash<br>○ Fever + rash + joint pain<br>○ Fever + rash + joint pain + conjunctivitis<br>○ Other<br>○ Fever + rash + other
Describe other ZIKV symptom-related inclusion criteria	__________________________________

For peer review only

What ZIKV-related symptoms in infants did your study consider when enrolling infants and/or their mothers?

- ◯ Fever only
- ◯ Rash only
- ◯ Fever + rash
- ◯ Fever or rash
- ◯ Fever + rash + joint pain
- ◯ Fever + rash + joint pain + conjunctivitis
- ◯ Other
- ◯ Fever + rash + other

What other ZIKV-related symptoms in infants did your study consider when enrolling participants?

____

Describe any additional ZIKV-related inclusion criteria

____

Describe any additional non-ZIKV related inclusion criteria (e.g. HIV co-infected, residence in a certain neighborhood, etc).

____

List any study exclusion criteria

____

What was your mechanism for identifying potential participants for your study?

Select all that apply

- ☐ Referrals from active surveillance system
- ☐ Referrals from hospital or clinic
- ☐ Preexisting cohort
- ☐ Other

What was your mechanism for identifying potential participants for your study?

____

Were potential participants referred to your study using a surveillance definition?

- ◯ Yes
- ◯ No

What was the surveillance definition used to refer participants to the study or surveillance system?

____

Where did recruitment take place?

Select all that apply

- ☐ Primary healthcare facility
- ☐ Tertiary healthcare facility
- ☐ Any healthcare facility (not specified by the study)
- ☐ Community
- ☐ Other

Where else did recruitment take place?

____

When during pregnancy were women enrolled?

- ◯ Prior to pregnancy
- ◯ During 1st trimester only
- ◯ During 1st or 2nd trimester only
- ◯ Anytime during pregnancy
- ◯ First obstetric visit
- ◯ Other

For peer review only - http://bmjopen.bmj.com/site/about/guidelines.xhtml

At what other time were women enrolled?

____

Who recruited potential participants?

- ◯ Health care facility staff
- ◯ Project interviewer (not health care facility staff member)
- ◯ Community health worker
- ◯ Other

Which other group enrolled research participants?

____

For peer review only

Follow up schedules for pregnant women and infants

Was the number and timing of visits during pregnancy the same for ZIKV+ and ZIKV- pregnant women?	◯ Yes<br>◯ No<br>◯ Study only followed ZIKV + pregnant women<br>◯ Study did not follow any pregnant women
How many visits did the study plan to have with pregnant women?	__________________________________<br>(If there is a range for the number of visits, please indicate the upper limit of the range)
At what time during pregnancy was the visit/were the visits planned to occur?	__________________________________<br>(e.g. every trimester, every month, months 1, 6, 9, etc.)
How many visits did the study plan to have with ZIKV+ pregnant women?	__________________________________<br>(If there is a range for the number of visits, please indicate the upper limit of the range)
At what time during pregnancy was the visit/were the visits scheduled to occur with ZIKV+ pregnant women?	__________________________________<br>(e.g. every trimester, every month, months 1, 6, 9, etc.)
How many visits did the study plan to have with ZIKV- pregnant women?	__________________________________<br>(If there is a range for the number of visits, please indicate the upper limit of the range)
At what time during pregnancy was the visit/were the visits scheduled to occur with ZIKV- pregnant women?	__________________________________<br>(e.g. every trimester, every month, months 1, 6, 9, etc.)
Other than timing and duration of follow-up, describe any differences in the types of assessments conducted with ZIKV+ and ZIKV- pregnant women:	__________________________________
Did the study follow infants after birth?	◯ Yes<br>◯ No
Was the number and timing of follow-up visits the same for the infants/children of ZIKV+ and ZIKV- pregnant women?	◯ Yes<br>◯ No<br>◯ Study did not follow infants/children of ZIKV- pregnant women
How many visits did the study plan to have with infants/children?	__________________________________<br>(If there is a range for the number of visits, please indicate the upper limit of the range)
Up until what age (in months) did your study intend to follow infants/children?	__________________________________

For peer review only

At what ages was the visit/were the visits with infants/children scheduled to occur?

____
(Specify whether ages listed in months or years)

How many visits did the study plan to have with infants/children from ZIKV+ mothers?

____
(If there is a range for the number of visits, please indicate the upper limit of the range)

Up until what age (in months) did your study plan to follow infants/children of ZIKV+ mothers?

____

At what ages was the visit/were the visits with infants/children of ZIKV+ mothers scheduled to occur?

____
(Specify whether ages listed in months or years)

How many visits did the study plan to have with infants/children of ZIKV- mothers?

____
(If there is a range for the number of visits, please indicate the upper limit of the range)

Up until what age (in months) did your study plan to follow infants/children of ZIKV- mothers?

____

At what ages was the visit/were the visits with infants/children of ZIKV- mothers scheduled to occur?

____
(Specify whether ages listed in months or years)

Other than timing and duration of follow-up, describe any differences in the types of assessments conducted with infants/children of ZIKV+ and ZIKV- pregnant women:

____

For peer review only

ZIKV symptoms in pregnant women and infants

Which of the following were measured to determine symptomatic ZIKV infection in women?

Select all that apply

- ☐ Fever
- ☐ Rash
- ☐ Joint pain
- ☐ Conjunctivitis
- ☐ Rash OR joint pain OR conjunctivitis OR fever
- ☐ Other

What else was measured to determine symptomatic ZIKV infection in women?

____

How was fever measured in women?

Select all that apply

- ☐ Underarm
- ☐ Sublingual
- ☐ Otic
- ☐ Rectal
- ☐ Self reported or subjective (any site)

How did your study define fever in women? (degrees Celsius)

____
(Degrees Celsius)

How was fever measured in infants?

Select all that apply

- ☐ Underarm
- ☐ Sublingual
- ☐ Otic
- ☐ Rectal
- ☐ Self reported or subjective (any site)

How did your study define fever in infants? (degrees Celsius)

____
(Degrees Celsius)

For peer review only

Ascertainment of maternal ZIKV infection

What methods were used to assess maternal ZIKV status?<br><br>Select all that apply	☐ Clinical case definition<br>☐ Molecular diagnosis (e.g. RT-PCR, other NAAT)<br>☐ Immunoassay (e.g. ELISA, IFA, PRNT, microneutralization)<br>☐ Rapid diagnostic test (RDT)<br>☐ Other
What other method was used to assess maternal ZIKV status?	__________________________________
Did you use different assays, different brands of the same assay, or different versions of the same brand of assay at different study sites to ascertain maternal ZIKV status or over time within the study?<br><br>Select all that apply	☐ Yes, different versions of the same brand of assay over time<br>☐ Yes, different brands of the same type of assay over time<br>☐ Yes, different types of assays over time<br>☐ Yes, different versions of the same brand of assay at different sites<br>☐ Yes, different brands of the same type of assay at different sites<br>☐ Yes, different types of assays at different sites<br>☐ No, same tests applied throughout the project
Which different assay(s) did you use over time or across sites?	__________________________________
Which different brands of the assay did you use over time or across sites?	__________________________________
Which different versions of the brand of assay did you use over time or across sites?	__________________________________

For peer review only

Ascertainment of maternal ZIKV infection - Immunoassay

ZIKV immunoassay analyte<br><br>Select all that apply	☐ IgM<br>☐ IgG<br>☐ IgA<br>☐ neutralizing antibodies<br>☐ Other
Other ZIKV immunoassay analyte	__________________________________
ZIKV immunoassay type<br><br>Select all that apply	☐ ELISA<br>☐ IFA<br>☐ MIA<br>☐ PRNT<br>☐ Other
Specify other type(s) or ZIKV immunoassay(s) performed	__________________________________

For peer review only

ZIKV immunoassay brand name<br><br>Select all that apply	☐ In house-developed assay<br>☐ Zika MAC-ELISA (CDC)<br>☐ ZIKV Detect 2.0 IgM Capture ELISA Kit (InBios International Inc)<br>☐ Zika IgM ELISA (EUROIMMUN)<br>☐ Zika IgM + IgA ELISA (EUROIMMUN)<br>☐ Zika IgG ELISA (EUROIMMUN)<br>☐ ZIKV IgM IFA (EUROIMMUN)<br>☐ ZIKV IgG IFA (EUROIMMUN)<br>☐ Arbovirus Fever Mosaic 2 IgG + IgM IFA ZIKV-DENV-CHIKV (EUROIMMUN)<br>☐ ADVIA Centaur Zika Test (Siemens Healthcare Diagnostics)<br>☐ DPP Zika IgM Assay System (Chembio Diagnostic Systems Inc)<br>☐ DPP Zika IgM/IgG System (Chembio Diagnostic Systems Inc)<br>☐ DPP Zika/Chikungunya/Dengue IgM/IgG Combination Assay (Chembio Diagnostic Systems Inc)<br>☐ LIAISON XL Zika Capture IgM II assay (DiaSorin S.p.A.)<br>☐ STANDARD Q Zika IgM/IgG test (SD Biosensor)<br>☐ STANDARD E Zika IgM ELISA (SD Biosensor)<br>☐ STANDARD Q ZIKV/DENV/CHIKV Fast Quad (SD Biosensor<br>☐ STANDARD Q ZIKV/DENV/CHIKV/YFV IgM Quad (SD Biosensor)<br>☐ Tell Me Fast Zika IgG/IgM Antibody Rapid Test (Biocan Diagnostics Inc)<br>☐ Tell Me Fast zika/dengue/chikungunya combination rapid tests (Biocan Diagnostics Inc)<br>☐ ZIKV-DENV-CHIKV IFA IgG (Vircell)<br>☐ ZIKV-DENV-CHIKV IFA IgM (Vircell)<br>☐ Human Anti-Zika ELISA Kits (Alpha Diagnostic International)<br>☐ anti-Zika virus IgM ELISA kit (µ-capture) (AbCam)<br>☐ anti-Zika virus IgG ELISA kit (AbCam)<br>☐ Zika Virus IgM ELISA Assay Kit (Eagle Biosciences)<br>☐ Zika Virus IgG ELISA Assay Kit (Eagle Biosciences)<br>☐ Human Anti-Zika Virus IgG ELISA Kit (R&D Systems)<br>☐ ZIKV IgG ELISA kit (DIA.PRO Diagnostic Bioprobes Srl)<br>☐ ZIKV IgM ELISA kit (DIA.PRO Diagnostic Bioprobes Srl)<br>☐ NovaLisa Zika Virus IgM µ-capture ELISA (NovaTec Immunodiagnostica GmbH)<br>☐ recomLine Tropical Fever IgG (MIKROGEN Diagnostik)<br>☐ recomLine Tropical Fever IgM (MIKROGEN Diagnostik)<br>☐ Other
Which other immunoassays were used to diagnose maternal ZIKV infection?	__________________________________
What type of assay is the house assay?<br><br>Select all that apply	☐ ELISA<br>☐ IFA<br>☐ MIA<br>☐ PRNT<br>☐ Other
What other type of assay is the in house assay?	__________________________________

For peer review only

What is the analyte measured by the in house assay?<br><br>Select all that apply	☐ IgM<br>☐ IgG<br>☐ IgA
What is the antigenic target for the in house assay?<br><br>Select all that apply	☐ NS1<br>☐ envelope protein<br>☐ NS5<br>☐ Other
What is the antigenic target for the in house assay?	__________________________________
For the in house ZIKV immunoassay, describe the testing procedure, including: incubation period, timing, type of cells, buffers, method to detect binding antibodies (e.g., optical density or secondary antibody detection), and methods to stop the enzymatic reaction	__________________________________
Sample used for ZIKV immunoassay<br><br>Select all that apply	☐ Serum/plasma<br>☐ Whole blood<br>☐ Other
Specify other sample used for ZIKV immunoassay	____________________________
ZIKV ELISA tested in paired/subsequent samples	◯ Yes<br>◯ No
At what temperature was the ZIKV ELISA test stored? (degrees Celsius)	____________________________
At what temperature(s) was/were the ZIKV ELISA sample(s) stored? (degrees Celsius)	__________________________________<br>(Include sample type for each temperature)
In what volume(s) was/were the ZIKV ELISA sample aliquot(s) stored? (mL)	__________________________________<br>(Include sample type with each volume)
Did your study deviate from the directions provided in the ZIKV ELISA commercial kit?	◯ Yes<br>◯ No<br>◯ Study did not use a commercial ZIKV ELISA kit
Describe any deviations from the procedure recommended by the commercial ELISA kit, including: incubation period, timing, type of cells, buffers, method to detect bounding antibodies, and methods to stop the enzymatic reaction	__________________________________
Describe any additional conditions for ZIKV ELISA test administration (e.g. paired samples within 21 days of fever onset; count with more than 2ul of serum from the febrile phase, etc.)	__________________________________

For peer review only

Where was the ZIKV ELISA test performed?<br><br>Select all that apply	☐ Lab at the same health care facility where study is conducted<br>☐ Study-specific lab<br>☐ Ministry of Health reference lab<br>☐ Private external lab<br>☐ Other
Specify other lab where ZIKV ELISA was performed	__________________________________

For peer review only

Ascertainment of maternal ZIKV infection - ELISA

ZIKV ELISA cut-off point ______________________

Was a positive control from the outbreak included for the ZIKV ELISAs?
- ◯ Yes
- ◯ No

Was a negative control included with every run of ZIKV ELISAs?
- ◯ Yes
- ◯ No

Definition of POSITIVE ZIKV ELISA ______________________

Definition of NEGATIVE ZIKV ELISA ______________________

Definition of INCONCLUSIVE ZIKV ELISA ______________________

Source of definition used to interpret ZIKV ELISA test results

Select all that apply
- ☐ Manufacturer
- ☐ Ministry of Health reference lab
- ☐ WHO (global or local office e.g: PAHO)
- ☐ US-CDC
- ☐ Consensus/study researchers
- ☐ Other

Specify other source of definition used to interpret ZIKV ELISA test results ______________________

How many ZIKV ELISAs did the study plan to conduct in each pregnant woman? ______________________

Would your study be able to provide the P/N ratio, titer, or other quantitative readout for ZIKV ELISAs to inform the measurement error models?
- ◯ Yes
- ◯ No

For peer review only

Ascertainment of maternal ZIKV infection - IFA

ZIKV IFA cut-off value

____

Was a positive control from the outbreak included for the ZIKV IFA?

- ◯ Yes
- ◯ No

Was a negative control included with every run of the ZIKV IFA?

- ◯ Yes
- ◯ No

Definition of POSITIVE ZIKV IFA

____

Definition of NEGATIVE ZIKV IFA

____

Definition of INCONCLUSIVE ZIKV IFA

____

Source of definition used to interpret ZIKV IFA test results

Select all that apply

- ☐ Manufacturer
- ☐ Ministry of Health reference lab
- ☐ WHO (global or local office e.g: PAHO)
- ☐ Consensus/study researchers
- ☐ Other

Specify other source of definition for ZIKV IFA test results

____

Specify any additional inclusion or exclusion criteria for determining which samples to test for ZIKV using IFA

____

Will your study be able to provide the titer or optical density for ZIKV IFAs to inform the measurement error model?

- ◯ Yes
- ◯ No

For peer review only

Ascertainment of maternal ZIKV infection - MIA

ZIKV MIA cut-off value

____

Was a positive control from the outbreak included for the ZIKV MIAs?
◯ Yes
◯ No

Was a negative control included with every run of ZIKV MIAs?
◯ Yes
◯ No

Definition of POSITIVE ZIKV MIA

____

Definition of NEGATIVE ZIKV MIA

____

Definition of INCONCLUSIVE ZIKV MIA

____

Source of definition used to interpret ZIKV MIA test results

Select all that apply

☐ Manufacturer
☐ Ministry of Health reference lab
☐ WHO (global or local office e.g: PAHO)
☐ Consensus/study researchers
☐ Other

Specify other source of definition for ZIKV MIA test results

____

Specify any additional inclusion or exclusion criteria for determining which samples to test for ZIKV using MIA

____

Will your study be able to provide the titer for ZIKV MIAs to inform the measurement error model?
◯ Yes
◯ No

For peer review only

Ascertainment of maternal ZIKV infection - PRNT

Did you use the CDC protocol for the PRNT?	◯ Yes<br>◯ No
Describe the other protocol used for the PRNT	__________________________________
End-point PRNT titer	◯ PRNT50<br>◯ PRNT90<br>◯ Other
Which other end-point did you use for the PRNT titer?	____________________________
Definition of a positive PRNT titer	◯ PRNT>10<br>◯ Other
Specify other definition of a positive PRNT titer	____________________________
Source of definition used to interpret ZIKV PRNT test results<br><br>Select all that apply	☐ Manufacturer<br>☐ Ministry of Health reference lab<br>☐ WHO (global or local office e.g: PAHO)<br>☐ US-CDC<br>☐ Consensus/study researchers<br>☐ Other
Specify other source of definition used to interpret ZIKV PRNT test results	____________________________
Definition of POSITIVE ZIKV PRNT	__________________________________
Definition of NEGATIVE ZIKV PRNT	__________________________________
Definition of INCONCLUSIVE PRNT (i.e. specific flavivirus could not be determined)	__________________________________
Specify any additional inclusion or exclusion criteria for determining which samples to test for ZIKV using PRNT	__________________________________
Where was the ZIKV PRNT test performed?<br><br>Select all that apply	☐ Lab at the same health care facility where study is conducted<br>☐ Study-specific lab<br>☐ Ministry of Health reference lab<br>☐ Private external lab<br>☐ Other
Specify other lab where ZIKV PRNT test was performed	____________________________

For peer review only

How many ZIKV PRNTs were conducted with pregnant women?	________________________
Will your study be able to provide the titer for ZIKV PRNTs to inform the measurement error model?	◯ Yes<br>◯ No

For peer review only

Ascertainment of maternal ZIKV infection - Molecular assay

Type of ZIKV molecular tests performed<br><br>Select all that apply	☐ conventional PCR<br>☐ rRT-PCR<br>☐ qRT-PCR<br>☐ Other NAAT
According to the study protocol, when were the ZIKV samples to be collected for molecular testing?<br><br>Select all that apply	☐ 3 days after fever onset<br>☐ 5 days after fever onset<br>☐ 7 days after fever onset<br>☐ 10 days after fever onset<br>☐ 14 days after fever onset<br>☐ Other
When else were the ZIKV samples to be collected for molecular testing?	__________________________________
Sample used for ZIKV RT-PCR/PCR<br><br>Select all that apply	☐ Serum/plasma<br>☐ Whole blood<br>☐ Urine<br>☐ Saliva<br>☐ Breast milk<br>☐ Umbilical cord tissue<br>☐ Placental tissue<br>☐ Amniotic fluid<br>☐ Other
Specify other type of sample used for ZIKV RT-PCR/PCR	____________________________

For peer review only

ZIKV RT-PCR/PCR brand name<br><br>Select all that apply	☐ In house technique<br>☐ Zika Virus RNA Qualitative Real-Time RT-PCR (Quest Diagnostics)<br>☐ RealStar Zika Virus RT-PCR Kit U.S. (altona Diagnostics GmbH)<br>☐ RealStar Zika Virus RT-PCR Kit 1.0 (altona Diagnostics GmbH)<br>☐ Aptima Zika Virus assay (Hologic Inc)<br>☐ Zika Virus Real-time RT-PCR (Viracor Eurofins)<br>☐ VERSANT Zika RNA 1.0 Assay (kPCR) (Siemens Healthcare Diagnostics Inc)<br>☐ xMAP MultiFLEX Zika RNA Assay (Luminex Corporation)<br>☐ Sentosa SA ZIKV RT-PCR Test (Vela Diagnostics Inc)<br>☐ Zika Virus Detection by RT-PCR (ARUP Laboratories)<br>☐ Abbott RealTime Zika (Abbott Molecular Inc)<br>☐ Zika ELITe MGB Kit U.S. (ELITechGroup Molecular Diagnostics)<br>☐ Gene-RADAR Zika Virus Test (Nanobiosym Diagnostics Inc)<br>☐ TaqPath Zika Virus Kit (Thermo Fisher Scientific)<br>☐ CII-ArboViroPlex rRT-PCR assay (Columbia University)<br>☐ Trioplex Real-Time RT-PCR Assay (CDC)<br>☐ Liferiver Zika Virus (ZIKV) Real Time RT-PCR Kit (Shanghai ZJ Bio-Tech Co Ltd)<br>☐ careGENE Zika Virus RT-PCR Kit (WELLS BIO Inc)<br>☐ Zika Virus - Single Check (Genekam Biotechnology AG)<br>☐ FTD Zika virus (Fast Track Diagnostics)<br>☐ VIASURE Zika Virus Real Time PCR Detection Kit (Certest Biotec)<br>☐ Zika Virus Real-TM (Sacace Biotechnologies)<br>☐ Logix Smart Zika Test Kit (Co-Diagnostics Inc)<br>☐ Genesig Kits for ZIKV (Primerdesign Ltd)<br>☐ Genesig Multiplex Kit for Dengue, Chikungunya and Zika Virus (Primerdesign Ltd)<br>☐ AccuPower ZIKV (DENV, CHIKV) Multiplex Real-Time RT-PCR Kit (Bioneer)<br>☐ VIASURE Zika, Dengue & Chikungunya Real Time PCR Detection Kit (Certest Biotec)<br>☐ FTD Zika/Dengue/Chik (Fast-Track Diagnostics)<br>☐ DiaPlexQ ZCD (ZIKV, CHIKV, DENV) Detection Kit (SolGent Co Ltd)<br>☐ TaqMan Zika Virus Triplex Kit (Thermo Fisher Scientific)<br>☐ GenoAmp Trioplex Real-Time RT-PCR Zika/Den/Chiku (Medical Innovation Ventures)<br>☐ Other
Describe procedure for in house ZIKV RT-PCR/PCR	______________________________
Specify other ZIKV RT-PCR/PCR brand name	______________________________
Did the study obtain a measure of ZIKV viral load?	◯ Yes<br>◯ No
ZIKV PCR/RT-PCR cut-off point	__________________________

For peer review only

Was a negative control included with every run of ZIKV RT-PCR/PCRs?	◯ Yes<br>◯ No
Definition of POSITIVE ZIKV RT-PCR/PCR	__________________________
Definition of NEGATIVE ZIKV RT-PCR/PCR	__________________________
Definition of INCONCLUSIVE ZIKV RT-PCR/PCR	__________________________
Source of definition used to interpret ZIKV RT-PCR/PCR test results<br><br>Select all that apply	☐ Manufacturer<br>☐ Ministry of Health reference lab<br>☐ WHO (global or local office e.g: PAHO)<br>☐ US-CDC<br>☐ Consensus/study researchers<br>☐ Other
Specify other source of definition used to interpret ZIKV RT-PCR/PCR test results	_____________________
Where was the ZIKV RT-PCR/PCR test performed?<br><br>Select all that apply	☐ Lab at the same health care facility where study is conducted<br>☐ Study-specific lab<br>☐ Ministry of Health reference lab<br>☐ Private external lab<br>☐ Other
Specify other lab where ZIKV RT-PCR/PCR test was performed	_____________________
Specify any additional inclusion or exclusion criteria for determining which samples to test for ZIKV using RT-PCR/PCR	__________________________
How many ZIKV PCRs were conducted with pregnant women?	_____________________
Would your study be able to provide a Ct value for ZIKV PCRs to inform the measurement error models?	◯ Yes<br>◯ No

For peer review only

ZIKV testing algorithm & test interpretation - Maternal ZIKV infection

Describe the testing algorithm for defining a confirmed ZIKV+ test in pregnant women

____

Describe the testing algorithm for defining a confirmed ZIKV- test in pregnant women

____

What tests were used to confirm ZIKV infection in pregnant women?

Select all that apply

- ☐ ZIKV IgM - 1 sample
- ☐ ZIKV IgM - paired samples (seroconversion or increased titers)
- ☐ ZIKV IgM (1 sample) + IgG
- ☐ ZIKV IgM (paired samples) + IgG
- ☐ RT-PCR or other NAAT
- ☐ PRNT > 10
- ☐ Other

Specify other test or combination of tests used to confirm ZIKV infection in pregnant women

____

Considering any relevant clinical and laboratory criteria, what was the study's definition of a CONFIRMED maternal ZIKV Infection

____

Considering any relevant clinical and laboratory criteria, what was the study's definition of a PROBABLE maternal ZIKV Infection

____

Considering any relevant clinical and laboratory criteria, what was the study's definition of a NEGATIVE/UNLIKELY maternal ZIKV Infection

____

Source of ZIKV test result categorization

Select all that apply

- ☐ Manufacturer
- ☐ Ministry of Health reference lab
- ☐ WHO (global or local office e.g: PAHO)
- ☐ Consensus/study researchers
- ☐ Other

Specify other source of ZIKV test result categorization

____

For peer review only - http://bmjopen.bmj.com/site/about/guidelines.xhtml

Changes in maternal ZIKV assessment over time

Which criteria did you use to infer maternal ZIKV status or fetal exposure to ZIKV?<br><br>Select all that apply	☐ Maternal clinical criteria<br>☐ Maternal laboratory criteria<br>☐ Infant clinical criteria<br>☐ Infant laboratory criteria
Which clinical criteria did you apply initially to diagnose maternal infection? (Clinical Definition 1)	__________________________________
Did your clinical criteria for diagnosing maternal infection change over time?	◯ No<br>◯ Yes - changed 1x<br>◯ Yes - changed more than 1x
If your clinical criteria changed over time, which criteria did you apply subsequently? (Clinical Definition 2)	__________________________________
When did you begin using the revised criteria? (Clinical Definition 2)	___________________________
What was the third set of criteria you applied? (Clinical Definition 3)	__________________________________
When did you begin using the third set of clinical criteria? (Clinical Definition 3)	___________________________
What laboratory criteria was used initially to diagnose maternal ZIKV infection? (Laboratory Definition 1)	__________________________________
Did your laboratory criteria for diagnosing maternal infection change over time?	◯ No<br>◯ Yes - changed 1x<br>◯ Yes - changed more than 1x
Which laboratory criteria did you apply subsequently? (Laboratory Definition 2)	__________________________________
When did you begin using the revised lab criteria? (Laboratory Definition 2)	___________________________
What was the third set of laboratory criteria you applied? (Laboratory Definition 3)	__________________________________
When did you begin using the third set of lab criteria? (Laboratory Definition 3)	___________________________
Were maternal samples retested using the new laboratory criteria?	◯ Yes<br>◯ No
If testing was performed for more than one arbovirus, including ZIKV, were they assessed simultaneously (i.e. multiplex testing) or separately?	◯ Yes, arboviruses tested simultaneously (multiplex)<br>◯ No, arboviruses tested separately

For peer review only

Ascertainment of maternal DENV infection

Did the study assess pregnant women's DENV status?	◯ Yes<br>◯ No
What methods were used to assess maternal DENV status?<br><br>Select all that apply	☐ Clinical case definition<br>☐ Molecular diagnosis (e.g. RT-PCR, other NAAT)<br>☐ Immunoassay (e.g. ELISA, IFA, PRNT, microneutralization)<br>☐ Rapid diagnostic test (RDT)<br>☐ Other
What other method was used to assess maternal DENV status?	__________________________________
Were ZIKV and DENV assessed using separate tests?	◯ DENV assessed separately from ZIKV<br>◯ DENV and ZIKV assessed using multiplex testing
At any time, did your study screen for DENV and only test DENV (-) participants for ZIKV?	◯ Yes, at some point study screened for DENV first, only tested DENV negative participants for ZIKV<br>◯ No, study always tested for DENV and ZIKV simultaneously
If yes, when did your study BEGIN only administering ZIKV tests to DENV- participants?	___________________________
If yes, when did your study STOP only administering ZIKV tests to DENV- participants?	___________________________

For peer review only

Ascertainment of maternal DENV infection - Immunoassay

DENV immunoassay analyte<br><br>Select all that apply	☐ IgM<br>☐ IgG<br>☐ IgA<br>☐ neutralizing antibodies<br>☐ Other
Other DENV immunoassay analyte	______________________
DENV immunoassay type<br><br>Select all that apply	☐ ELISA<br>☐ IFA<br>☐ MIA<br>☐ PRNT<br>☐ Other
Specify other type(s) or DENV immunoassay(s) performed	______________________
DENV immunoassay brand name<br><br>Select all that apply	☐ In house technique<br>☐ MAC-ELISA<br>☐ DENV Detect ELISA IgM/IgG (InBios International, United States)<br>☐ Dengue DxSelect IgM/IgG (Focus Diagnostics, USA)<br>☐ PATHOZYME-DENGUE IgM/IgG (Omega Diagnostics, UK)<br>☐ Dengue IgM/IgG Capture ELISA (Panbio Diagnostics, Australia)<br>☐ Dengue IgM/IgG Capture ELISA (Standard Diagnostics, South Korea)<br>☐ Platelia Dengue IgM/IgG ELISA" (Bio-Rad, France)<br>☐ SD Bioline Dengue IgM/IgG (Alere, United States)<br>☐ Arbovirus Fever Mosaic 2 IgG + IgM IFA ZIKV-DENV-CHIKV (EUROIMMUN)<br>☐ recomLine Tropical Fever IgG (MIKROGEN Diagnostik)<br>☐ recomLine Tropical Fever IgM (MIKROGEN Diagnostik)<br>☐ DPP Zika/Chikungunya/Dengue IgM/IgG Combination Assay (Chembio Diagnostic Systems Inc)<br>☐ STANDARD Q ZIKV/DENV/CHIKV Fast Quad (SD Biosenso<br>☐ STANDARD Q ZIKV/DENV/CHIKV/YFV IgM Quad (SD Biosensor)<br>☐ Tell Me Fast zika/dengue/chikungunya combination rapid tests (Biocan Diagnostics Inc)<br>☐ Other
Which other immunoassays were used to diagnose maternal DENV infection?	______________________
What type of assay is the in house assay?<br><br>Select all that apply	☐ ELISA<br>☐ IFA<br>☐ MIA<br>☐ PRNT<br>☐ Other
What type of assay is the in house assay?	______________________

For peer review only

What is the analyte measured by the in house assay?<br><br>Select all that apply	☐ IgM<br>☐ IgG<br>☐ IgA
What is the antigenic target for the in house assay<br><br>Select all that apply	☐ NS1<br>☐ envelope protein<br>☐ NS5<br>☐ Other
What is the antigenic target for the in house assay	__________________________________
For the in house DENV immunoassay, describe the testing procedure, including: incubation period, timing, type of cells, buffers, method to detect binding antibodies (e.g., optical density or secondary antibody detection), and methods to stop the enzymatic reaction	__________________________________
Sample used for DENV immunoassay<br><br>Select all that apply	☐ Serum/plasma<br>☐ Whole blood<br>☐ Urine<br>☐ Saliva<br>☐ Breast milk<br>☐ Umbilical cord tissue<br>☐ Placental tissue<br>☐ Amniotic fluid<br>☐ Other
Specify other type of sample used for DENV immunoassay	____________________________

For peer review only

Ascertainment of maternal DENV infection - ELISA

DENV ELISA cut-off value

____

Was a positive control from the outbreak included for the DENV ELISAs?

◯ Yes
◯ No

Was a negative control included with every run of DENV ELISAs?

◯ Yes
◯ No

Definition of POSITIVE DENV ELISA

____

Definition of NEGATIVE DENV ELISA

____

Definition of INCONCLUSIVE DENV ELISA

____

Source of definition used to interpret DENV ELISA test results

Select all that apply

☐ Manufacturer
☐ Ministry of Health reference lab
☐ WHO (global or local office e.g: PAHO)
☐ Consensus/study researchers
☐ Other

Specify other source of definition for DENV ELISA test results

____

Specify any additional inclusion or exclusion criteria for determining which samples to test for DENV using ELISA

____

Would your study be able to provide the P/N ratio, titer, or other quantitative readout for DENV ELISAs to inform the measurement error models?

◯ Yes
◯ No

For peer review only

Ascertainment of maternal DENV infection - IFA

DENV IFA cut-off value

____

Was a positive control from the outbreak included for the DENV IFA?

◯ Yes
◯ No

Was a negative control included with every run of DENV IFA?

◯ Yes
◯ No

Definition of POSITIVE DENV IFA

____

Definition of NEGATIVE DENV IFA

____

Definition of INCONCLUSIVE DENV IFA

____

Source of definition used to interpret DENV IFA test results

Select all that apply

☐ Manufacturer
☐ Ministry of Health reference lab
☐ WHO (global or local office e.g: PAHO)
☐ Consensus/study researchers
☐ Other

Specify other source of definition for DENV IFA test results

____

Specify any additional inclusion or exclusion criteria for determining which samples to test for DENV using IFA

____

Will your study be able to provide the titer or optical density for DENV IFAs to inform the measurement error model?

◯ Yes
◯ No

For peer review only

Ascertainment of maternal DENV infection - MIA

DENV MIA cut-off value ______________________

Was a positive control from the outbreak included for the DENV MIAs?
◯ Yes
◯ No

Was a negative control included with every run of DENV MIAs?
◯ Yes
◯ No

Definition of POSITIVE DENV MIA ______________________

Definition of NEGATIVE DENV MIA ______________________

Definition of INCONCLUSIVE DENV MIA ______________________

Source of definition used to interpret DENV MIA test results

Select all that apply

☐ Manufacturer
☐ Ministry of Health reference lab
☐ WHO (global or local office e.g: PAHO)
☐ Consensus/study researchers
☐ Other

Specify other source of definition for DENV MIA test results ______________________

Specify any additional inclusion or exclusion criteria for determining which samples to test for DENV using MIA ______________________

Will your study be able to provide the titer for DENV MIAs to inform the measurement error model?
◯ Yes
◯ No

For peer review only

Ascertainment of maternal DENV infection - Immunoassay - PRNT

Did you use the CDC protocol for the DENV PRNT?
◯ Yes
◯ No

Describe the other protocol used for the DENV PRNT?
____

Did your study simultaneously perform PRNT for ZIKV and DENV?
◯ Yes
◯ No

End-point PRNT titer
◯ PRNT50
◯ PRNT90
◯ Other

Which other end-point did you use for the PRNT titer?
____

Definition of a positive PRNT titer
◯ PRNT>10
◯ Other

Other definition of a positive PRNT titer
____

Definition of POSITIVE DENV PRNT
____

Definition of NEGATIVE DENV PRNT
____

Definition of INCONCLUSIVE DENV PRNT
____

Source of definition used to interpret DENV PRNT test results

Select all that apply
☐ Manufacturer
☐ Ministry of Health reference lab
☐ WHO (global or local office e.g: PAHO)
☐ US-CDC
☐ Consensus/study researchers
☐ Other

Specify other source of definition used to interpret DENV PRNT test results
____

Specify any additional inclusion or exclusion criteria for determining which samples to test for DENV using PRNT
____

Where was the DENV PRNT test performed?

Select all that apply
☐ Lab at the same health care facility where study is conducted
☐ Study-specific lab
☐ Ministry of Health reference lab
☐ Private external lab
☐ Other

For peer review only

Specify other lab where DENV PRNT test was performed	__________________________
Will your study be able to provide the titer for DENV PRNTs to inform the measurement error model?	◯ Yes<br>◯ No
Can your study provide the full differences between the ZIKV and DENV titers or information about the magnitude of the difference? (e.g. is the difference 4 fold or greater? Is the difference 1 or less than 1?)	◯ Yes<br>◯ No<br>◯ Unsure

For peer review only

Ascertainment of maternal DENV infection - Molecular assay

Type of DENV PCR/RT-PCR performed<br><br>Select all that apply	☐ PCR (conventional or standard)<br>☐ qRT-PCR<br>☐ rRT-PCR<br>☐ No DENV PCR performed
DENV PCR/RT-PCR brand name<br><br>Select all that apply	☐ In house procedure<br>☐ CDC DENV-1-4 rRT-PCR Multiplex and Trioplex rRT-PCR Assays (CDC-USA)<br>☐ CDC DENV-1-4 Real-Time RT-PCR Multiplex Assay (CDC-USA)<br>☐ CDC Trioplex rRT-PCR assay (CDC-USA)<br>☐ Dengue Fever Virus & Chikungunya Virus Real Time RT-PCR Kit (Creative Biogene)<br>☐ Dengue Virus General-type Real Time RT-PCR Kit (Creative Biogene)<br>☐ RealStar Dengue RT-PCR (Altona Diagnostics-Hamburg,Germany)<br>☐ GenoAmp Trioplex Real-Time RT-PCR Zika/Den/Chiku (Medical Innovation Ventures)<br>☐ GenoAmp Real-Time RT-PCR Dengue (Serotyping of Dengue 1-Dengue 4) (Medical Innovation Ventures)<br>☐ RT-PCR kit (RealArt; artus/Qiagen-Germany)<br>☐ SimplexaTMdengue RT-PCR assay (Focus Diagnostics-Cypress, CA)<br>☐ Dengue virus general type real-time RT-PCR kit LiferiverTM (Shanghai ZJ Bio-Tech Co-China)<br>☐ Geno-Sen's dengue 1-4 real-time RT-PCR kit (Genome Diagnostics Pvt-New Delhi, India)<br>☐ CII-ArboViroPlex rRT-PCR assay (Columbia University)<br>☐ AccuPower ZIKV (DENV, CHIKV) Multiplex Real-Time RT-PCR kit (Bioneer)<br>☐ VIASURE Zika, Dengue & Chikungunya Real Time PCR Detection Kit (Certest Biotec)<br>☐ FTD Zika/Dengue/Chik (Fast-Track Diagnostics)<br>☐ DiaPlexQ ZCD (ZIKV, CHIKV, DENV) Detection Kit (SolGent Co Ltd)<br>☐ TaqMan Zika Virus Triplex Kit (Thermo Fisher Scientific)<br>☐ Genesig Multiplex Kit for Dengue, Chikungunya and Zika Virus (Primerdesign Ltd)<br>☐ Other
Describe procedure for in house DENV PCR/RT-PCR	__________________________________
Specify other DENV PCR/RT-PCR used	__________________________________
Sample used for DENV PCR/RT-PCR<br><br>Select all that apply	☐ Serum/plasma<br>☐ Whole blood<br>☐ Urine<br>☐ Saliva<br>☐ Breast milk<br>☐ Umbilical cord tissue<br>☐ Placental tissue<br>☐ Amniotic fluid<br>☐ Other

For peer review only

Specify other type of sample used for DENV PCR/RT-PCR	__________________
Did the study obtain a measure of DENV viral load?	◯ Yes<br>◯ No
DENV PCR/RT-PCR cut-off point	__________________
Was a negative control included with every run of DENV PCR/RT-PCRs?	◯ Yes<br>◯ No
Definition of POSITIVE DENV PCR/RT-PCR	__________________
Definition of NEGATIVE DENV PCR/RT-PCR	__________________
Definition of INCONCLUSIVE DENV PCR/RT-PCR	__________________
Source of definition used to interpret DENV PCR/RT-PCR test results<br><br>Select all that apply	☐ Manufacturer<br>☐ Ministry of Health reference lab<br>☐ WHO (global or local office e.g: PAHO)<br>☐ Consensus/study researchers<br>☐ Other
Specify other source of definition for DENV PCR/RT-PCR test results	__________________
Specify any additional inclusion or exclusion criteria for determining which samples to test for DENV using PCR/RT-PCR	__________________
Would your study be able to provide a Ct value for DENV PCR/RT-PCRs to inform the measurement error models?	◯ Yes<br>◯ No

For peer review only

DENV testing algorithm & qualitative definitions

What assay was used to confirm maternal DENV infection?

- ◯ NAAT (PCR) only
- ◯ NS-1 only
- ◯ IgM only
- ◯ IgG increase in paired samples
- ◯ IgM seroconversion in paired samples
- ◯ PRNT + any serologic test (IgM, IgG, or both)
- ◯ PCR or any NAAT + any serologic test (IgM, IgG or both)
- ◯ Other combination of assays

Specify other combination of assays used to confirm maternal DENV infection

____

Considering any relevant clinical and laboratory criteria, what was the study's definition of a CONFIRMED maternal DENV infection

____

Considering any relevant clinical and laboratory criteria, what was the study's definition of a PROBABLE maternal DENV infection

____

Considering any relevant clinical and laboratory criteria, what was the study's definition of a NEGATIVE/UNLIKELY maternal DENV infection?

____

For peer review only

Ascertainment of maternal CHIKV infection

Did the study conduct chikv-specific laboratory tests?	◯ Yes, chikv assessed separately from ZIKV<br>◯ Yes, chikv assessed using multiplex testing<br>◯ No, chikv not assessed
What methods were used to assess maternal CHIKV status?<br><br>Select all that apply	☐ Clinical case definition<br>☐ Molecular diagnosis (e.g. RT-PCR, other NAAT)<br>☐ Immunoassay (e.g. ELISA, IFA, PRNT, microneutralization)<br>☐ Rapid diagnostic test (RDT)<br>☐ Other
What other method was used to assess maternal CHIKV status?	__________________________________
CHIKV immunoassay analyte<br><br>Select all that apply	☐ IgM<br>☐ IgG<br>☐ IgA<br>☐ neutralizing antibodies<br>☐ Other
Other CHIKV immunoassay analyte	___________________________
CHIKV immunoassay type<br><br>Select all that apply	☐ ELISA<br>☐ IFA<br>☐ MIA<br>☐ PRNT<br>☐ Other
Specify other type(s) or CHIKV immunoassay(s) performed	___________________________

For peer review only

CHIKV immunoassay brand name<br><br>Select all that apply	☐ In house technique<br>☐ MAC-ELISA<br>☐ CHIK IgM micro-capture ELISA (IBL International -Germany)<br>☐ RecombiLISA CHIK IgM Test (CTK Biotech-USA/China)<br>☐ CHIKV IgM µ-capture ELISA (Genway-Germany)<br>☐ Anti-CHIKV IgM human ELISA kit (Abcam-Germany)<br>☐ CHIKa IgM ELISA (SD Diagnostics South Korea)<br>☐ Anti-CHIKV ELISA (IgM) (Euroimmun-Germany)<br>☐ CHIKjj Detect MAC-ELISA (Inbios-USA)<br>☐ anti-chikungunya virus ELISA (IgM) kit (Euroimmun US-Mountain Lakes, NJ)<br>☐ Chikungunya IgM µ-capture ELISA (IBL International-Hamburg, Germany)<br>☐ Chikungunya IgG Capture ELISA (IBL International-Hamburg, Germany)<br>☐ Anti-Chikungunya Virus ELISA IgG test (Euroimmun-Lübeck, Germany)<br>☐ Arbovirus Fever Mosaic 2 IgG + IgM IFA ZIKV-DENV-CHIKV (EUROIMMUN)<br>☐ recomLine Tropical Fever IgG (MIKROGEN Diagnostik)<br>☐ recomLine Tropical Fever IgM (MIKROGEN Diagnostik)<br>☐ DPP Zika/Chikungunya/Dengue IgM/IgG Combination Assay (Chembio Diagnostic Systems Inc)<br>☐ STANDARD Q ZIKV/DENV/CHIKV Fast Quad (SD Biosensor<br>☐ STANDARD Q ZIKV/DENV/CHIKV/YFV IgM Quad (SD Biosensor)<br>☐ Tell Me Fast zika/dengue/chikungunya combination rapid tests (Biocan Diagnostics Inc<br>☐ Other
Which other immunoassays were used to diagnose maternal CHIKV infection?	__________________________________
For in house CHIKV immunoassay, describe the testing procedure, including: incubation period, timing, type of cells, buffers, method to detect binding antibodies, and methods to stop the enzymatic reaction	__________________________________
Sample used for CHIKV immunoassay<br><br>Select all that apply	☐ Serum/plasma<br>☐ Whole blood<br>☐ Urine<br>☐ Saliva<br>☐ Breast milk<br>☐ Umbilical cord tissue<br>☐ Placental tissue<br>☐ Amniotic fluid<br>☐ Other
Specify other type of sample used in CHIKV immunoassay	__________________________________

For peer review only

Ascertainment of maternal CHIKV infection - ELISA

CHIKV ELISA cut-off value

____

Was a positive control from the outbreak included for the CHIKV ELISAs?
- ◯ Yes
- ◯ No

Was a negative control included with every run of CHIKV ELISAs?
- ◯ Yes
- ◯ No

Definition of POSITIVE CHIKV ELISA

____

Definition of NEGATIVE CHIKV ELISA

____

Definition of INCONCLUSIVE CHIKV ELISA

____

Source of definition used to interpret CHIKV ELISA test results

Select all that apply
- ☐ Manufacturer
- ☐ Ministry of Health reference lab
- ☐ WHO (global or local office e.g: PAHO)
- ☐ Consensus/study researchers
- ☐ Other

Specify other source of definition for CHIKV ELISA test results

____

Specify any additional inclusion or exclusion criteria for determining which samples to test for CHIKV using ELISA

____

Would your study be able to provide the P/N ratio, titer, or other quantitative readout for CHIKV ELISAs to inform the measurement error models?
- ◯ Yes
- ◯ No

For peer review only

Ascertainment of maternal CHIKV infection - IFA

CHIKV IFA cut-off value

____

Was a positive control from the outbreak included for the CHIKV IFA?

◯ Yes
◯ No

Was a negative control included with every run of CHIKV IFA?

◯ Yes
◯ No

Definition of POSITIVE CHIKV IFA

____

Definition of NEGATIVE CHIKV IFA

____

Definition of INCONCLUSIVE CHIKV IFA

____

Source of definition used to interpret CHIKV IFA test results

Select all that apply

☐ Manufacturer
☐ Ministry of Health reference lab
☐ WHO (global or local office e.g: PAHO)
☐ Consensus/study researchers
☐ Other

Specify other source of definition for CHIKV IFA test results

____

Specify any additional inclusion or exclusion criteria for determining which samples to test for CHIKV using IFA

____

Will your study be able to provide the optical density or titer for CHIKV IFAs to inform the measurement error model?

◯ Yes
◯ No

For peer review only

Ascertainment of maternal CHIKV infection - MIA

CHIKV MIA cut-off value	__________________________________
Was a positive control from the outbreak included for the CHIKV MIAs?	◯ Yes<br>◯ No
Was a negative control included with every run of CHIKV MIAs?	◯ Yes<br>◯ No
Definition of POSITIVE CHIKV MIA	__________________________________
Definition of NEGATIVE CHIKV MIA	__________________________________
Definition of INCONCLUSIVE CHIKV MIA	__________________________________
Source of definition used to interpret CHIKV MIA test results<br><br>Select all that apply	☐ Manufacturer<br>☐ Ministry of Health reference lab<br>☐ WHO (global or local office e.g: PAHO)<br>☐ Consensus/study researchers<br>☐ Other
Specify other source of definition for CHIKV MIA test results	__________________________________
Specify any additional inclusion or exclusion criteria for determining which samples to test for CHIKV using MIA	__________________________________
Will your study be able to provide the titer for CHIKV MIAs to inform the measurement error model?	◯ Yes<br>◯ No

For peer review only

Ascertainment of maternal CHIKV infection - Molecular assay

Type of CHIKV PCR/RT-PCR performed<br><br>Select all that apply	☐ PCR (standard or conventional)<br>☐ qRT-PCR<br>☐ rRT-PCR<br>☐ No CHIKV PCR performed
CHIKV PCR/RT-PCR brand name<br><br>Select all that apply	☐ In house procedure<br>☐ CHIKV CDC qRT-PCR (CDC-USA)<br>☐ CDC Trioplex rRT-PCR Assay (CDC-USA)<br>☐ RealStar Chikungunya RT-PCR Kit (Altona Diagnostics-Germany)<br>☐ Genesig Chikungunya Non structural protein 2 standard (nsp2) RT-PCR kit (Primerdesign-UK)<br>☐ CII-ArboViroPlex rRT-PCR assay (Columbia University)<br>☐ AccuPower ZIKV (DENV, CHIKV) Multiplex Real-Time RT-PCR kit (Bioneer)<br>☐ VIASURE Zika, Dengue & Chikungunya Real Time PCR Detection Kit (Certest Biotec)<br>☐ FTD Zika/Dengue/Chik (Fast-Track Diagnostics)<br>☐ DiaPlexQ ZCD (ZIKV, CHIKV, DENV) Detection Kit (SolGent Co Ltd)<br>☐ TaqMan Zika Virus Triplex Kit (Thermo Fisher Scientific)<br>☐ Dengue Fever Virus & Chikungunya Virus Real Time RT-PCR Kit (Creative Biogene)<br>☐ GenoAmp Trioplex Real-Time RT-PCR Zika/Den/Chiku (Medical Innovation Ventures)<br>☐ Genesig Multiplex Kit for Dengue, Chikungunya and Zika Virus (Primerdesign Ltd)<br>☐ Other
Describe procedure for in house PCR/RT-PCR	__________________________________
Specify other CHIKV PCR/RT-PCR brand used	__________________________________
Sample used for CHIKV PCR/RT-PCR<br><br>Select all that apply	☐ Serum/plasma<br>☐ Whole blood<br>☐ Urine<br>☐ Saliva<br>☐ Breast milk<br>☐ Umbilical cord tissue<br>☐ Placental tissue<br>☐ Amniotic fluid<br>☐ Other
Specify other type of sample used for CHIKV PCR/RT-PCR	__________________________________
Did the study obtain a measure of CHIKV viral load?	◯ Yes<br>◯ No
CHIKV PCR/RT-PCR cut-off point	__________________________________
Was a negative control included with every run of CHIKV PCR/RT-PCRs?	◯ Yes<br>◯ No

For peer review only

Definition of POSITIVE CHIKV PCR/RT-PCR	__________________
Definition of NEGATIVE CHIKV PCR/RT-PCR	__________________
Definition of INCONCLUSIVE CHIKV PCR/RT-PCR	__________________
Source of the definition used to interpret CHIKV PCR/RT-PCR test results<br><br>Select all that apply	☐ Manufacturer<br>☐ Ministry of Health reference lab<br>☐ WHO (global or local office e.g: PAHO)<br>☐ US-CDC<br>☐ Consensus/study researchers<br>☐ Other
Specify other source of the definition used to interpret CHIKV PCR/RT-PCR test results	__________________
Specify any additional inclusion or exclusion criteria for determining which samples to test for CHIKV using PCR/RT-PCR	__________________
Where was the CHIKV PCR/RT-PCR test performed?<br><br>Select all that apply	☐ Lab at the same health care facility where study is conducted<br>☐ Study-specific lab<br>☐ Ministry of Health reference lab<br>☐ Private external lab<br>☐ Other
Specify other lab where the CHIKV PCR/RT-PCR test was performed	__________________

For peer review only

CHIKV testing algorithm & qualitative definitions

What tests were used to confirm CHIKV infection in pregnant women?<br><br>Select all that apply	☐ CHIKV IgM - 1 sample<br>☐ CHIKV IgM - paired samples (seroconversion or increased titers)<br>☐ CHIKV IgM (1 sample) + IgG<br>☐ CHIKV IgM (paired samples) + IgG<br>☐ RT-PCR or other NAAT<br>☐ PRNT > 10<br>☐ Other
Specify other test or combination of tests used to confirm CHIKV infection	__________________________________
Considering any relevant clinical and laboratory criteria, what was the study's definition of a CONFIRMED maternal CHIKV infection	__________________________________
Considering any relevant clinical and laboratory criteria, what was the study's definition of a PROBABLE maternal CHIKV infection	__________________________________
Considering any relevant clinical and laboratory criteria, what was the study's definition of a NEGATIVE maternal CHIKV infection	__________________________________

For peer review only

Ascertainment of maternal infection with other flavi/alphaviruses

Does the study capture information on maternal history of any other flavivirus/alphavirus infections?	◯ Yes, self-reported<br>◯ Yes, lab assessment<br>◯ Yes, both self-reported and lab assessment<br>◯ No
Does the study capture information on maternal history of yellow fever or Japanese encephalitis vaccination?	◯ Yes<br>◯ No
Was testing performed for other current maternal arbovirus infections? If so, which ones?<br><br>Select all that apply	☐ DENV<br>☐ CHIKV<br>☐ Japanese/Venezuelan encephalitis<br>☐ Mayaro virus<br>☐ West Nile virus<br>☐ Other arboviruses<br>☐ No, study did not test for concurrent arbovirus infections
Which other current maternal arboviral infections did the study test for?	__________________________

For peer review only

Ascertainment of placental or fetal ZIKV infection

Was placental ZIKV infection measured?

◯ Yes
◯ No

Was fetal ZIKV infection measured?

◯ Yes
◯ No

Which criteria did you use to infer fetal ZIKV status?

Select all that apply

☐ fetal clinical criteria
☐ fetal laboratory criteria
☐ fetal imaging criteria
☐ maternal clinical criteria
☐ maternal laboratory criteria
☐ placental clinical criteria
☐ placental laboratory criteria
☐ other criteria

In what other way was fetal ZIKV infection assessed?

____

Which laboratory tests were used to assess fetal ZIKV status?

Select all that apply

☐ molecular diagnosis (e.g. RT-PCR, other NAAT)
☐ immunoassay (e.g. ELISA, IFA, PRNT, IHC, microneutralization)
☐ rapid diagnostic test (RDT)
☐ Other

Which other laboratory tests were used to assess fetal ZIKV status?

____

Were the assays or the clinical criteria used to assess fetal ZIKV infection the same as the assays or clinical criteria used to assess placental ZIKV infection?

◯ Yes
◯ No

Please describe the differences between the clinical criteria or assays used to assess fetal and placental ZIKV infection

____

When was fetal ZIKV infection measured using laboratory criteria?

Select all that apply

☐ 1st trimester
☐ 2nd trimester
☐ 3rd trimester
☐ First antenatal visit
☐ Following confirmation of maternal ZIKV infection, irregardless of trimester
☐ Other

Type of sample(s) obtained to ascertain fetal ZIKV infection

Select all that apply

☐ Amniotic fluid
☐ Placental tissue (Including umbilical cord and fetal membranes)
☐ Fetal tissue
☐ Other

What other type(s) of samples were obtained to ascertain fetal ZIKV infection?

____

For peer review only

At what other time was fetal ZIKV infection measured?	______________________________
Where was the fetal sample analyzed?<br><br>Select all that apply	☐ Lab at the same health care facility where study is conducted<br>☐ Study-specific lab<br>☐ Ministry of Health reference lab<br>☐ Private external lab<br>☐ Other
Specify other lab where the fetal sample was analyzed	_________________________
Did you use different assays, different brands of the same assay, or different versions of the same brand of assay at different study sites to ascertain fetal ZIKV status or over time within the study?<br><br>Select all that apply	☐ Yes, different versions of the same brand of assay over time<br>☐ Yes, different brands of the same type of assay over time<br>☐ Yes, different types of assays over time<br>☐ Yes, different versions of the same brand of assay at different sites<br>☐ Yes, different brands of the same type of assay at different sites<br>☐ Yes, different types of assays at different sites<br>☐ No, same tests applied throughout the project
Which different assay(s) did you use over time or across sites?	______________________________
Which different brands of the assay did you use over time or across sites?	______________________________
Which different versions of the brand of assay did you use over time or across sites?	______________________________

For peer review only

Ascertainment of fetal ZIKV infection - Immunoassay

ZIKV immunoassay analyte to ascertain fetal infection<br><br>Select all that apply	☐ IgM<br>☐ IgG<br>☐ IgA<br>☐ neutralizing antibodies<br>☐ Other
Other ZIKV immunoassay analyte to ascertain fetal infection	__________________________________
ZIKV immunoassay type to ascertain fetal infection<br><br>Select all that apply	☐ ELISA<br>☐ IFA<br>☐ MIA<br>☐ PRNT<br>☐ Other
Specify other type(s) or ZIKV immunoassay(s) performed to ascertain fetal infection	__________________________________

For peer review only

ZIKV immunoassay brand name<br><br>Select all that apply	☐ In house-developed assay<br>☐ Zika MAC-ELISA (CDC)<br>☐ ZIKV Detect 2.0 IgM Capture ELISA Kit (InBios International Inc)<br>☐ Zika IgM ELISA (EUROIMMUN)<br>☐ Zika IgM + IgA ELISA (EUROIMMUN)<br>☐ Zika IgG ELISA (EUROIMMUN)<br>☐ ZIKV IgM IFA (EUROIMMUN)<br>☐ ZIKV IgG IFA (EUROIMMUN)<br>☐ Arbovirus Fever Mosaic 2 IgG + IgM IFA ZIKV-DENV-CHIKV (EUROIMMUN)<br>☐ ADVIA Centaur Zika Test (Siemens Healthcare Diagnostics)<br>☐ DPP Zika IgM Assay System (Chembio Diagnostic Systems Inc)<br>☐ DPP Zika IgM/IgG System (Chembio Diagnostic Systems Inc)<br>☐ DPP Zika/Chikungunya/Dengue IgM/IgG Combination Assay (Chembio Diagnostic Systems Inc)<br>☐ LIAISON XL Zika Capture IgM II assay (DiaSorin S.p.A.)<br>☐ STANDARD Q Zika IgM/IgG test (SD Biosensor)<br>☐ STANDARD E Zika IgM ELISA (SD Biosensor)<br>☐ STANDARD Q ZIKV/DENV/CHIKV Fast Quad (SD Biosenso<br>☐ STANDARD Q ZIKV/DENV/CHIKV/YFV IgM Quad (SD Biosensor)<br>☐ Tell Me Fast Zika IgG/IgM Antibody Rapid Test (Biocan Diagnostics Inc)<br>☐ Tell Me Fast zika/dengue/chikungunya combination rapid tests (Biocan Diagnostics Inc)<br>☐ ZIKV-DENV-CHIKV IFA IgG (Vircell)<br>☐ ZIKV-DENV-CHIKV IFA IgM (Vircell)<br>☐ Human Anti-Zika ELISA Kits (Alpha Diagnostic International)<br>☐ anti-Zika virus IgM ELISA kit (µ-capture) (AbCam)<br>☐ anti-Zika virus IgG ELISA kit (AbCam)<br>☐ Zika Virus IgM ELISA Assay Kit (Eagle Biosciences)<br>☐ Zika Virus IgG ELISA Assay Kit (Eagle Biosciences)<br>☐ Human Anti-Zika Virus IgG ELISA Kit (R&D Systems)<br>☐ ZIKV IgG ELISA kit (DIA.PRO Diagnostic Bioprobes Srl)<br>☐ ZIKV IgM ELISA kit (DIA.PRO Diagnostic Bioprobes Srl)<br>☐ NovaLisa Zika Virus IgM µ-capture ELISA (NovaTec Immunodiagnostica GmbH)<br>☐ recomLine Tropical Fever IgG (MIKROGEN Diagnostik)<br>☐ recomLine Tropical Fever IgM (MIKROGEN Diagnostik)<br>☐ Other
Which other immunoassays were used to diagnose fetal ZIKV infection?	__________________________________
What type of assay is the in house assay (to ascertain fetal infection)?<br><br>Select all that apply	☐ ELISA<br>☐ IFA<br>☐ MIA<br>☐ PRNT<br>☐ Other
What type of assay is the in house assay (to ascertain fetal infection)?	__________________________________

For peer review only

What is the analyte measured by the in house assay (to ascertain fetal infection)?<br><br>Select all that apply	☐ IgM<br>☐ IgG<br>☐ IgA
What is the antigenic target for the in house assay (to ascertain fetal infection)?<br><br>Select all that apply	☐ NS1<br>☐ envelope protein<br>☐ NS5<br>☐ Other
What is the antigenic target for the in house assay (to ascertain fetal infection)?	__________________________________
For the in house ZIKV immunoassay to ascertain fetal infection, describe the testing procedure, including: incubation period, timing, type of cells, buffers, method to detect binding antibodies (e.g., optical density or secondary antibody detection), and methods to stop the enzymatic reaction	__________________________________
Sample used for ZIKV immunoassay (to ascertain fetal infection)<br><br>Select all that apply	☐ Serum/plasma<br>☐ Whole blood<br>☐ cerebrospinal fuid (CSF)<br>☐ chord blood<br>☐ Other
Specify other sample used for ZIKV immunoassay (to ascertain fetal infection)	____________________________
ZIKV ELISA tested in paired/subsequent samples for ascertaining fetal infection	◯ Yes<br>◯ No
At what temperature was the ZIKV ELISA test stored for fetal infection? (degrees Celsius)	____________________________
At what temperature(s) was/were the ZIKV ELISA sample(s) stored for fetal infection? (degrees Celsius)	__________________________________<br>(Include sample type for each temperature)
In what volume(s) was/were the ZIKV ELISA sample aliquot(s) stored for fetal infection? (mL)	__________________________________<br>(Include sample type with each volume)
Did your study deviate from the directions provided in the ZIKV ELISA commercial kit for fetal infection?	◯ Yes<br>◯ No<br>◯ Study did not use a commercial ZIKV ELISA kit
Describe any deviations from the procedure recommended by the commercial ELISA kit, including: incubation period, timing, type of cells, buffers, method to detect bounding antibodies, and methods to stop the enzymatic reaction	__________________________________

For peer review only

Describe any additional conditions for ZIKV ELISA test administration for ascertaining fetal infection (e.g. paired samples within 21 days of fever onset; count with more than 2ul of serum from the febrile phase, etc.)	__________________________________
Where was the ZIKV ELISA test performed?<br><br>Select all that apply	☐ Lab at the same health care facility where study is conducted<br>☐ Study-specific lab<br>☐ Ministry of Health reference lab<br>☐ Private external lab<br>☐ Other
Specify other lab where ZIKV ELISA was performed for fetal infection	____________________________

For peer review only

Ascertainment of fetal ZIKV infection - ELISA

ZIKV ELISA cut-off point for fetal infection	__________________________________
Was a positive control from the outbreak included for the ZIKV ELISAs for fetal infection?	◯ Yes<br>◯ No
Was a negative control included with every run of ZIKV ELISAs for fetal infection?	◯ Yes<br>◯ No
Definition of POSITIVE ZIKV ELISA for fetal infection	__________________________________
Definition of NEGATIVE ZIKV ELISA for fetal infection	__________________________________
Definition of INCONCLUSIVE ZIKV ELISA for fetal infection	__________________________________
Source of definition used to interpret ZIKV immunoassay test results<br><br>Select all that apply	☐ Manufacturer<br>☐ Ministry of Health reference lab<br>☐ WHO (global or local office e.g: PAHO)<br>☐ US-CDC<br>☐ Consensus/study researchers<br>☐ Other
Specify other source of definition used to interpret ZIKV immunoassay test results	__________________________________
How many ZIKV ELISAs did the study plan to conduct in each fetus?	__________________________________
Would your study be able to provide the P/N ratio, titer, or other quantitative readout for ZIKV ELISAs to inform the measurement error models?	◯ Yes<br>◯ No

For peer review only

Ascertainment of fetal ZIKV infection - IFA

ZIKV IFA cut-off value for fetal infection

____

Was a positive control from the outbreak included for the ZIKV IFA for fetal infection?

◯ Yes
◯ No

Was a negative control included with every run of ZIKV IFA for fetal infection?

◯ Yes
◯ No

Definition of POSITIVE ZIKV IFA for fetal infection

____

Definition of NEGATIVE ZIKV IFA for fetal infection

____

Definition of INCONCLUSIVE ZIKV IFA for fetal infection

____

Source of definition for ZIKV IFA test results for fetal infection

Select all that apply

☐ Manufacturer
☐ Ministry of Health reference lab
☐ WHO (global or local office e.g: PAHO)
☐ Consensus/study researchers
☐ Other

Specify other source of definition for ZIKV IFA test results

____

Specify any additional inclusion or exclusion criteria for determining which samples to test for ZIKV using IFA

____

Will your study be able to provide the titer or optical density for ZIKV IFAs to inform the measurement error model?

◯ Yes
◯ No

For peer review only

Ascertainment of fetal ZIKV infection - MIA

ZIKV MIA cut-off value for fetal infection ________________

Was a positive control from the outbreak included for the ZIKV MIAs for fetal infection?
- ◯ Yes
- ◯ No

Was a negative control included with every run of ZIKV MIAs for fetal infection?
- ◯ Yes
- ◯ No

Definition of POSITIVE ZIKV MIA for fetal infection ________________

Definition of NEGATIVE ZIKV MIA for fetal infection ________________

Definition of INCONCLUSIVE ZIKV MIA for fetal infection ________________

Source of definition for MIA ZIKV test results for fetal infection

Select all that apply
- ☐ Manufacturer
- ☐ Ministry of Health reference lab
- ☐ WHO (global or local office e.g: PAHO)
- ☐ Consensus/study researchers
- ☐ Other

Specify other source of definition for ZIKV MIA test results ________________

Specify any additional inclusion or exclusion criteria for determining which samples to test for ZIKV using MIA for fetal infection ________________

Will your study be able to provide the titer for ZIKV MIAs to inform the measurement error model?
- ◯ Yes
- ◯ No

For peer review only

Ascertainment of fetal ZIKV infection - Molecular assay

Type of ZIKV molecular tests performed on fetus<br><br>Select all that apply	☐ conventional PCR<br>☐ rRT-PCR<br>☐ qRT-PCR<br>☐ Other NAAT
According to the study protocol, when were the ZIKV samples to be collected for molecular testing?<br><br>Select all that apply	☐ within 24 hours after birth<br>☐ within first week after birth<br>☐ Other
When were the ZIKV molecular tests (RT-PCR, other NAAT) conducted on fetus?	__________________________________
Sample used for ZIKV RT-RCR/PCR in fetus<br><br>Select all that apply	☐ Serum/plasma<br>☐ Whole blood<br>☐ Urine<br>☐ Saliva<br>☐ Breast milk<br>☐ Umbilical cord tissue<br>☐ cerebrospinal fuid (CSF)<br>☐ chord blood<br>☐ Other
Specify other type of sample used for ZIKV RT-PCR/PCR on fetus	____________________________

For peer review only

ZIKV RT-PCR/PCR brand name used for testing fetus<br><br>Select all that apply	☐ In house technique<br>☐ Zika Virus RNA Qualitative Real-Time RT-PCR (Quest Diagnostics)<br>☐ RealStar Zika Virus RT-PCR Kit U.S. (altona Diagnostics GmbH)<br>☐ RealStar Zika Virus RT-PCR Kit 1.0 (altona Diagnostics GmbH)<br>☐ Aptima Zika Virus assay (Hologic Inc)<br>☐ Zika Virus Real-time RT-PCR (Viracor Eurofins)<br>☐ VERSANT Zika RNA 1.0 Assay (kPCR) (Siemens Healthcare Diagnostics Inc)<br>☐ xMAP MultiFLEX Zika RNA Assay (Luminex Corporation)<br>☐ Sentosa SA ZIKV RT-PCR Test (Vela Diagnostics Inc)<br>☐ Zika Virus Detection by RT-PCR (ARUP Laboratories)<br>☐ Abbott RealTime Zika (Abbott Molecular Inc)<br>☐ Zika ELITe MGB Kit U.S. (ELITechGroup Molecular Diagnostics)<br>☐ Gene-RADAR Zika Virus Test (Nanobiosym Diagnostics Inc)<br>☐ TaqPath Zika Virus Kit (Thermo Fisher Scientific)<br>☐ CII-ArboViroPlex rRT-PCR assay (Columbia University)<br>☐ Trioplex Real-Time RT-PCR Assay (CDC)<br>☐ Liferiver Zika Virus (ZIKV) Real Time RT-PCR Kit (Shanghai ZJ Bio-Tech Co Ltd)<br>☐ careGENE Zika Virus RT-PCR Kit (WELLS BIO Inc)<br>☐ Zika Virus - Single Check (Genekam Biotechnology AG)<br>☐ FTD Zika virus (Fast Track Diagnostics)<br>☐ VIASURE Zika Virus Real Time PCR Detection Kit (Certest Biotec)<br>☐ Zika Virus Real-TM (Sacace Biotechnologies)<br>☐ Logix Smart Zika Test Kit (Co-Diagnostics Inc)<br>☐ Genesig Kits for ZIKV (Primerdesign Ltd)<br>☐ Genesig Multiplex Kit for Dengue, Chikungunya and Zika Virus (Primerdesign Ltd)<br>☐ AccuPower ZIKV (DENV, CHIKV) Multiplex Real-Time RT-PCR Kit (Bioneer)<br>☐ VIASURE Zika, Dengue & Chikungunya Real Time PCR Detection Kit (Certest Biotec)<br>☐ FTD Zika/Dengue/Chik (Fast-Track Diagnostics)<br>☐ DiaPlexQ ZCD (ZIKV, CHIKV, DENV) Detection Kit (SolGent Co Ltd)<br>☐ TaqMan Zika Virus Triplex Kit (Thermo Fisher Scientific)<br>☐ GenoAmp Trioplex Real-Time RT-PCR Zika/Den/Chiku (Medical Innovation Ventures)<br>☐ Other
Describe procedure for in house ZIKV RT-PCR/PCR for fetal infection	__________________________________
Specify other ZIKV RT-PCR/PCR brand name	__________________________________
Did the study obtain a measure of ZIKV viral load for fetal infection?	○ Yes<br>○ No
ZIKV PCR/RT-PCR cut-off point for fetal infection	____________________________

For peer review only

Was a negative control included with every run of ZIKV RT-PCR/PCRs for fetal infection?	◯ Yes<br>◯ No
Definition of POSITIVE ZIKV RT-PCR/PCR for fetal infection	__________________________________
Definition of NEGATIVE ZIKV RT-PCR/PCR for fetal infection	__________________________________
Definition of INCONCLUSIVE ZIKV RT-PCR/PCR for fetal infection	__________________________________
Source of definition used to interpret ZIKV RT-PCR/PCR test results for fetal infection<br><br>Select all that apply	☐ Manufacturer<br>☐ Ministry of Health reference lab<br>☐ WHO (global or local office e.g: PAHO)<br>☐ US-CDC<br>☐ Consensus/study researchers<br>☐ Other
Specify other source of definition used to interpret ZIKV RT-PCR/PCR test results	___________________________
Where was the ZIKV RT-PCR/PCR test performed for samples from fetus?<br><br>Select all that apply	☐ Lab at the same health care facility where study is conducted<br>☐ Study-specific lab<br>☐ Ministry of Health reference lab<br>☐ Private external lab<br>☐ Other
Specify other lab where ZIKV RT-PCR/PCR test was performed for fetal samples	___________________________
Specify any additional inclusion or exclusion criteria for determining which samples from fetus to test for ZIKV using RT-PCR/PCR	__________________________________
How many ZIKV PCRs were conducted with with each fetus?	___________________________
Would your study be able to provide a Ct value for fetal ZIKV PCRs to inform the measurement error models?	◯ Yes<br>◯ No

For peer review only

Changes in diagnosis of fetal ZIKV infection over time

Which clinical criteria did you apply initially to diagnose fetal infection? (Clinical Definition 1) __________________________________

Did your clinical criteria for fetal infection change over time?
- ◯ No
- ◯ Yes - changed 1x
- ◯ Yes - changed more than 1x

If your clinical criteria changed over time, which criteria did you apply subsequently to assess fetal infection? (Clinical Definition 2) __________________________________

When did you begin using the revised clinical criteria for assessing fetal infection? (Clinical Definition 2) ____________________________

What was the third set of criteria you applied to assess fetal infection? (Clinical Definition 3) __________________________________

When did you begin using the third set of clinical criteria? (Clinical Definition 3) ____________________________

Which imaging criteria did you apply initially to diagnose fetal infection? (Clinical Definition 1) __________________________________

Did your imaging criteria for fetal infection change over time?
- ◯ No
- ◯ Yes - changed 1x
- ◯ Yes - changed more than 1x

If your imaging criteria changed over time, which criteria did you apply subsequently to assess fetal infection? (Clinical Definition 2) __________________________________

When did you begin using the revised imaging criteria for assessing fetal infection? (Clinical Definition 2) ____________________________

What was the third set of imaging criteria you applied to assess fetal infection? (Clinical Definition 3) __________________________________

When did you begin using the third set of imaging criteria? (Clinical Definition 3) ____________________________

What laboratory criteria was used initially to diagnose fetal ZIKV infection? (Laboratory Definition 1) __________________________________

Did your laboratory criteria for diagnosing fetal infection change over time?
- ◯ No
- ◯ Yes - changed 1x
- ◯ Yes - changed more than 1x

For peer review only

Which laboratory criteria did you apply subsequently? (Laboratory Definition 2) __________________________________

When did you begin using the revised lab criteria for fetal infection? (Laboratory Definition 2) ____________________________

What was the third set of laboratory criteria you applied for diagnosing fetal infection? (Laboratory Definition 3) __________________________________

When did you begin using the third set of lab criteria for fetal infection? (Laboratory Definition 3) ____________________________

Were fetal samples retested using the new laboratory criteria? __________________________________

For peer review only

ZIKV testing algorithm & test interpretation - fetal ZIKV infection

Describe the testing algorithm for defining a confirmed ZIKV+ test in fetal testing

____

Describe the testing algorithm for defining a confirmed ZIKV- test in fetal testing

____

What tests were used to confirm ZIKV infection in fetal testing?

Select all that apply

- ☐ ZIKV IgM - 1 sample
- ☐ ZIKV IgM - paired samples (seroconversion or increased titers)
- ☐ ZIKV IgM (1 sample) + IgG
- ☐ ZIKV IgM (paired samples) + IgG
- ☐ RT-PCR or other NAAT
- ☐ PRNT > 10
- ☐ Other

Specify other test or combination of tests used to confirm ZIKV infection in fetal testing

____

Considering any relevant clinical and laboratory criteria, what was the study's definition of a CONFIRMED fetal ZIKV Infection

____

Considering any relevant clinical and laboratory criteria, what was the study's definition of a PROBABLE fetal ZIKV Infection

____

Considering any relevant clinical and laboratory criteria, what was the study's definition of a NEGATIVE/UNLIKELY fetal ZIKV Infection

____

Source of ZIKV test result categorization for fetal testing

Select all that apply

- ☐ Manufacturer
- ☐ Ministry of Health reference lab
- ☐ WHO (global or local office e.g: PAHO)
- ☐ Consensus/study researchers
- ☐ Other

Specify other source of ZIKV test result categorization for fetal testing

____

For peer review only

Fetal Imaging & Ascertainment of Prenatal Microcephaly

Was fetal ultrasound performed?
- ◯ Yes
- ◯ No

How many fetal ultrasounds did the study plan to conduct for each pregnant woman? ______________________

When in pregnancy did the study plan to conduct the ultrasound(s)? ______________________
(List all trimesters or weeks of gestation the study had planned to administer ultrasound)

Fetal ultrasound machine model(s) ______________________

Where were fetal ultrasounds performed?

Select all that apply
- ☐ Same health care facility where study is taking place
- ☐ Study-specific site (not a health care facility)
- ☐ Private/external site

Training received by ultrasound technician ______________________

Quality control for ultrasound evaluation of abnormalities/head circumference ______________________

Criteria for considering a fetal ultrasound as "abnormal" ______________________

Was fetal MRI performed?
- ◯ Yes
- ◯ No

How many fetal MRIs did the study plan to conduct for each pregnant woman? ______________________

When in pregnancy did the study plan to conduct the fetal MRI(s)? ______________________
(List all trimesters or weeks of gestation the study had planned to administer MRIs)

Fetal MRI machine model(s) ______________________

Where were fetal MRIs performed?

Select all that apply
- ☐ Same health care facility where study is taking place
- ☐ Study-specific site (not a health care facility)
- ☐ Private/external site

For peer review only

Training received by MRI technician	__________________
Quality control for MRI evaluation of abnormalities/head circumference	__________________
Criteria for considering a fetal MRI as "abnormal"	__________________
When was prenatal/fetal microcephaly diagnosed?	◯ Postnatally diagnosed (after birth) congenital microcephaly<br>◯ Prenatally diagnosed (in utero) congenital microcephaly<br>◯ Study did not measure prenatal/fetal microcephaly
Gestational age (in weeks) at which study first assessed prenatal microcephaly	__________________
Reference standards used to calculate fetal head circumference Z score<br><br>Select all that apply	☐ Intergrowth<br>☐ Ministry of Health standards<br>☐ WHO or PAHO standard<br>☐ Local reference standard<br>☐ Consensus/study researchers<br>☐ Other
Specify other reference standards used to calculate fetal head circumference Z score	__________________
How was prenatal microcephaly diagnosed?<br><br>Select all that apply	☐ Presence vs absence<br>☐ By grades (normocephaly vs microcephaly vs severe microcephaly)<br>☐ By percentiles<br>☐ By Z-score<br>☐ By diagnostic certainty criteria<br>☐ Other
Specify other type of diagnosis of prenatal microcephaly	__________________
How was prenatal microcephaly defined?	◯ >2 SD below the mean<br>◯ < 3 SD below the mean<br>◯ 3 SD below the mean<br>◯ >3 SD below the mean<br>◯ below the 5th percentile<br>◯ below the 10th percentile<br>◯ Other
Specify other definition of prenatal microcephaly	__________________

For peer review only

How was prenatal microcephaly defined?

◯ Level 1A of diagnostic certainty
◯ Level 1B of diagnostic certainty
◯ Level 2 of diagnostic certainty
◯ Level 3A of diagnostic certainty
◯ Level 3B of diagnostic certainty
◯ Level 4 of diagnostic certainty
◯ Other

How else was prenatal microcephaly defined?

____

For peer review only

Ascertainment of infant ZIKV infection - Immunoassay

Was infant ZIKV infection measured?
- ◯ Yes
- ◯ No

Which criteria did you use to infer infant ZIKV status?

Select all that apply
- ☐ infant clinical criteria
- ☐ infant laboratory criteria
- ☐ infant imaging criteria
- ☐ maternal clinical criteria
- ☐ maternal laboratory criteria
- ☐ other criteria

In what other way was infant ZIKV infection assessed?

____

When was infant ZIKV infection measured?

Select all that apply
- ☐ Immediately after birth
- ☐ 1 month after birth
- ☐ 6 months after birth
- ☐ Other

At what other time was infant ZIKV infection measured?

____

Type of laboratory test performed to ascertain infant ZIKV infection

Select all that apply
- ☐ molecular diagnosis (e.g. RT-PCR, other NAAT)
- ☐ immunoassay (e.g. ELISA, IFA, PRNT, IHC, microneutralization)
- ☐ rapid diagnostic test (RDT)
- ☐ Other

Specify other test performed to ascertain infant ZIKV infection

____

Did you use different assays, different brands of the same assay, or different versions of the same brand of assay at different study sites to ascertain infant ZIKV status or over time within the study?

Select all that apply
- ☐ Yes, different versions of the same brand of assay over time
- ☐ Yes, different brands of the same type of assay over time
- ☐ Yes, different types of assays over time
- ☐ Yes, different versions of the same brand of assay at different sites
- ☐ Yes, different brands of the same type of assay at different sites
- ☐ Yes, different types of assays at different sites
- ☐ No, same tests applied throughout the project

Which different assay(s) did you use over time or across sites?

____

Which different brands of the assay did you use over time or across sites?

____

Which different versions of the brand of assay did you use over time or across sites?

____

For peer review only

ZIKV immunoassay analyte to ascertain infant infection<br><br>Select all that apply	☐ IgM<br>☐ IgG<br>☐ IgA<br>☐ neutralizing antibodies<br>☐ Other
Other ZIKV immunoassay analyte to ascertain infant infection	________________
ZIKV immunoassay type to ascertain infant infection<br><br>Select all that apply	☐ ELISA<br>☐ IFA<br>☐ MIA<br>☐ PRNT<br>☐ Other
Specify other type(s) or ZIKV immunoassay(s) performed to ascertain infant infection	________________

For peer review only

ZIKV immunoassay brand name (to ascertain infant infection)<br><br>Select all that apply	☐ In house-developed assay<br>☐ Zika MAC-ELISA (CDC)<br>☐ ZIKV Detect 2.0 IgM Capture ELISA Kit (InBios International Inc)<br>☐ Zika IgM ELISA (EUROIMMUN)<br>☐ Zika IgM + IgA ELISA (EUROIMMUN)<br>☐ Zika IgG ELISA (EUROIMMUN)<br>☐ ZIKV IgM IFA (EUROIMMUN)<br>☐ ZIKV IgG IFA (EUROIMMUN)<br>☐ Arbovirus Fever Mosaic 2 IgG + IgM IFA ZIKV-DENV-CHIKV (EUROIMMUN)<br>☐ ADVIA Centaur Zika Test (Siemens Healthcare Diagnostics)<br>☐ DPP Zika IgM Assay System (Chembio Diagnostic Systems Inc)<br>☐ DPP Zika IgM/IgG System (Chembio Diagnostic Systems Inc)<br>☐ DPP Zika/Chikungunya/Dengue IgM/IgG Combination Assay (Chembio Diagnostic Systems Inc)<br>☐ LIAISON XL Zika Capture IgM II assay (DiaSorin S.p.A.)<br>☐ STANDARD Q Zika IgM/IgG test (SD Biosensor)<br>☐ STANDARD E Zika IgM ELISA (SD Biosensor)<br>☐ STANDARD Q ZIKV/DENV/CHIKV Fast Quad (SD Biosenso<br>☐ STANDARD Q ZIKV/DENV/CHIKV/YFV IgM Quad (SD Biosensor)<br>☐ Tell Me Fast Zika IgG/IgM Antibody Rapid Test (Biocan Diagnostics Inc)<br>☐ Tell Me Fast zika/dengue/chikungunya combination rapid tests (Biocan Diagnostics Inc)<br>☐ ZIKV-DENV-CHIKV IFA IgG (Vircell)<br>☐ ZIKV-DENV-CHIKV IFA IgM (Vircell)<br>☐ Human Anti-Zika ELISA Kits (Alpha Diagnostic International)<br>☐ anti-Zika virus IgM ELISA kit (μ-capture) (AbCam)<br>☐ anti-Zika virus IgG ELISA kit (AbCam)<br>☐ Zika Virus IgM ELISA Assay Kit (Eagle Biosciences)<br>☐ Zika Virus IgG ELISA Assay Kit (Eagle Biosciences)<br>☐ Human Anti-Zika Virus IgG ELISA Kit (R&D Systems)<br>☐ ZIKV IgG ELISA kit (DIA.PRO Diagnostic Bioprobes Srl)<br>☐ ZIKV IgM ELISA kit (DIA.PRO Diagnostic Bioprobes Srl)<br>☐ NovaLisa Zika Virus IgM μ-capture ELISA (NovaTec Immunodiagnostica GmbH)<br>☐ recomLine Tropical Fever IgG (MIKROGEN Diagnostik)<br>☐ recomLine Tropical Fever IgM (MIKROGEN Diagnostik)<br>☐ Other
Which other immunoassays were used to diagnose infant ZIKV infection?	__________________________________
What type of assay is the in house assay? (to ascertain infant ZIKV infection)<br><br>Select all that apply	☐ ELISA<br>☐ IFA<br>☐ MIA<br>☐ PRNT<br>☐ Other
What type of assay is the in house assay? (to ascertain infant ZIKV infection)	__________________________________

For peer review only - http://bmjopen.bmj.com/site/about/guidelines.xhtml

What is the analyte measured by the in house assay? (to ascertain infant ZIKV infection)<br><br>Select all that apply	☐ IgM<br>☐ IgG<br>☐ IgA
What is the antigenic target for the in house assay (to ascertain infant ZIKV infection)<br><br>Select all that apply	☐ NS1<br>☐ envelope protein<br>☐ NS5<br>☐ Other
What is the antigenic target for the in house assay (to ascertain infant ZIKV infection)	__________________________________
For the in house ZIKV immunoassay to ascertain infant infection, describe the testing procedure, including: incubation period, timing, type of cells, buffers, method to detect binding antibodies (e.g., optical density or secondary antibody detection), and methods to stop the enzymatic reaction	__________________________________
Sample used for ZIKV immunoassay (to ascertain infant infection)<br><br>Select all that apply	☐ Serum/plasma<br>☐ Whole blood<br>☐ cerebrospinal fuid (CSF)<br>☐ chord blood<br>☐ Other
Specify other sample used for ZIKV immunoassay (to ascertain infant infection)	____________________________
ZIKV ELISA tested in paired/subsequent samples	◯ Yes<br>◯ No
At what temperature was the ZIKV ELISA test stored? (degrees Celsius)	____________________________
At what temperature(s) was/were the ZIKV ELISA sample(s) stored? (degrees Celsius)	__________________________________<br>(Include sample type for each temperature)
In what volume(s) was/were the ZIKV ELISA sample aliquot(s) stored? (mL)	__________________________________<br>(Include sample type with each volume)
Did your study deviate from the directions provided in the ZIKV ELISA commercial kit?	◯ Yes<br>◯ No<br>◯ Study did not use a commercial ZIKV ELISA kit
Describe any deviations from the procedure recommended by the commercial ELISA kit, including: incubation period, timing, type of cells, buffers, method to detect bounding antibodies, and methods to stop the enzymatic reaction	__________________________________

For peer review only

Describe any additional conditions for ZIKV ELISA test administration for ascertining infant infection (e.g. paired samples within 21 days of fever onset; count with more than 2ul of serum from the febrile phase, etc.)	__________________________________
Where was the ZIKV ELISA test performed for ascertining infant infection?<br><br>Select all that apply	☐ Lab at the same health care facility where study is conducted<br>☐ Study-specific lab<br>☐ Ministry of Health reference lab<br>☐ Private external lab<br>☐ Other
Specify other lab where ZIKV ELISA was performed	__________________________________

For peer review only

Ascertainment of infant ZIKV infection - ELISA

ZIKV ELISA cut-off point for ascertining infant infection
____

Was a positive control from the outbreak included for the ZIKV ELISAs for ascertining infant infection?
◯ Yes
◯ No

Was a negative control included with every run of ZIKV ELISAs for ascertining infant infection?
◯ Yes
◯ No

Definition of POSITIVE ZIKV ELISA for ascertining infant infection
____

Definition of NEGATIVE ZIKV ELISA for ascertining infant infection
____

Definition of INCONCLUSIVE ZIKV ELISA for ascertining infant infection
____

Source of definition used to interpret ZIKV immunoassay test results for ascertining infant infection

Select all that apply

☐ Manufacturer
☐ Ministry of Health reference lab
☐ WHO (global or local office e.g: PAHO)
☐ US-CDC
☐ Consensus/study researchers
☐ Other

Specify other source of definition used to interpret ZIKV immunoassay test results
____

How many ZIKV ELISAs did the study plan to conduct in each infant?
____

Would your study be able to provide the P/N ratio, titer, or other quantitative readout for ZIKV ELISAs to inform the measurement error models?
◯ Yes
◯ No

For peer review only

Ascertainment of infant ZIKV infection - IFA

ZIKV IFA cut-off value for ascertining infant infection ______________________

Was a positive control from the outbreak included for the ZIKV IFA for ascertining infant infection?
◯ Yes
◯ No

Was a negative control included with every run of ZIKV IFA for ascertining infant infection?
◯ Yes
◯ No

Definition of POSITIVE ZIKV IFA for ascertining infant infection ______________________

Definition of NEGATIVE ZIKV IFA for ascertining infant infection ______________________

Definition of INCONCLUSIVE ZIKV IFA for ascertining infant infection ______________________

Source of definition for ZIKV IFA test results for ascertining infant infection

Select all that apply
☐ Manufacturer
☐ Ministry of Health reference lab
☐ WHO (global or local office e.g: PAHO)
☐ Consensus/study researchers
☐ Other

Specify other source of definition for ZIKV IFA test results for ascertining infant infection ______________________

Specify any additional inclusion or exclusion criteria for determining which samples to test for ZIKV using IFA for ascertining infant infection ______________________

Will your study be able to provide the titer or optical density for ZIKV IFAs to inform the measurement error model?
◯ Yes
◯ No

For peer review only

Ascertainment of infant ZIKV infection - MIA

ZIKV MIA cut-off value for ascertining infant infection	__________________
Was a positive control from the outbreak included for the ZIKV MIAs for ascertining infant infection?	◯ Yes<br>◯ No
Was a negative control included with every run of ZIKV MIAs for ascertining infant infection?	◯ Yes<br>◯ No
Definition of POSITIVE ZIKV MIA for ascertining infant infection	__________________
Definition of NEGATIVE ZIKV MIA for ascertining infant infection	__________________
Definition of INCONCLUSIVE ZIKV MIA for ascertining infant infection	__________________
Source of definition for MIA ZIKV test results for ascertining infant infection<br><br>Select all that apply	☐ Manufacturer<br>☐ Ministry of Health reference lab<br>☐ WHO (global or local office e.g: PAHO)<br>☐ Consensus/study researchers<br>☐ Other
Specify other source of definition for ZIKV MIA test results	__________________
Specify any additional inclusion or exclusion criteria for determining which samples to test for ZIKV using MIA for ascertining infant infection	__________________
Will your study be able to provide the titer for ZIKV MIAs to inform the measurement error model?	◯ Yes<br>◯ No

For peer review only

Ascertainment of infant ZIKV infection - Immunoassay - PRNT

Did you use the CDC protocol for the PRNT for ascertining infant infection?
- ◯ Yes
- ◯ No

Describe the other protocol used for the PRNT

____

End-point PRNT titer for infants
- ◯ PRNT50
- ◯ PRNT90
- ◯ Other

Which other end-point did you use for the PRNT titer for infant infection?

____

Definition of a positive PRNT titer for infants
- ◯ PRNT>10
- ◯ Other

Other definition of a positive PRNT titer for infants

____

Source of the definition used to interpret ZIKV PRNT test results for infants

Select all that apply
- ☐ Manufacturer
- ☐ Ministry of Health reference lab
- ☐ WHO (global or local office e.g: PAHO)
- ☐ US-CDC
- ☐ Consensus/study researchers
- ☐ Other

Specify other source of definition used to interpret ZIKV PRNT test results

____

Definition of POSITIVE ZIKV PRNT for infants

____

Definition of NEGATIVE ZIKV PRNT for infants

____

Definition of INCONCLUSIVE PRNT (i.e. specific flavivirus could not be determined) for infants

____

Specify any additional inclusion or exclusion criteria for determining which infant samples to test for ZIKV using PRNT

____

Where was the ZIKV PRNT test performed for samples from infants?

Select all that apply
- ☐ Lab at the same health care facility where study is conducted
- ☐ Study-specific lab
- ☐ Ministry of Health reference lab
- ☐ Private external lab
- ☐ Other

Specify other lab where ZIKV PRNT test was performed for samples from infants

____

For peer review only

How many ZIKV PRNTs were conducted with samples from infants? ______________________________

Will your study be able to provide the titer for ZIKV PRNTs from infants to inform the measurement error model?
◯ Yes
◯ No

For peer review only

Ascertainment of infant ZIKV infection - Molecular assay

Type of ZIKV molecular tests performed in infants<br><br>Select all that apply	☐ conventional PCR<br>☐ rRT-PCR<br>☐ qRT-PCR<br>☐ Other NAAT
According to the study protocol, when were the ZIKV samples to be collected for molecular testing?<br><br>Select all that apply	☐ within 24 hours after birth<br>☐ within first week after birth<br>☐ Other
When were the ZIKV molecular tests (RT-PCR, other NAAT) conducted in infants?	______________________________
Sample used for ZIKV RT-RCR/PCR in infants<br><br>Select all that apply	☐ Serum/plasma<br>☐ Whole blood<br>☐ Urine<br>☐ Saliva<br>☐ Breast milk<br>☐ Umbilical cord tissue<br>☐ cerebrospinal fuid (CSF)<br>☐ chord blood<br>☐ Other
Specify other type of sample used for ZIKV RT-PCR/PCR in infants	________________________

For peer review only

ZIKV RT-PCR/PCR brand name used for testing infants<br><br>Select all that apply	☐ In house technique<br>☐ Zika Virus RNA Qualitative Real-Time RT-PCR (Quest Diagnostics)<br>☐ RealStar Zika Virus RT-PCR Kit U.S. (altona Diagnostics GmbH)<br>☐ RealStar Zika Virus RT-PCR Kit 1.0 (altona Diagnostics GmbH)<br>☐ Aptima Zika Virus assay (Hologic Inc)<br>☐ Zika Virus Real-time RT-PCR (Viracor Eurofins)<br>☐ VERSANT Zika RNA 1.0 Assay (kPCR) (Siemens Healthcare Diagnostics Inc)<br>☐ xMAP MultiFLEX Zika RNA Assay (Luminex Corporation)<br>☐ Sentosa SA ZIKV RT-PCR Test (Vela Diagnostics Inc)<br>☐ Zika Virus Detection by RT-PCR (ARUP Laboratories)<br>☐ Abbott RealTime Zika (Abbott Molecular Inc)<br>☐ Zika ELITe MGB Kit U.S. (ELITechGroup Molecular Diagnostics)<br>☐ Gene-RADAR Zika Virus Test (Nanobiosym Diagnostics Inc)<br>☐ TaqPath Zika Virus Kit (Thermo Fisher Scientific)<br>☐ CII-ArboViroPlex rRT-PCR assay (Columbia University)<br>☐ Trioplex Real-Time RT-PCR Assay (CDC)<br>☐ Liferiver Zika Virus (ZIKV) Real Time RT-PCR Kit (Shanghai ZJ Bio-Tech Co Ltd)<br>☐ careGENE Zika Virus RT-PCR Kit (WELLS BIO Inc)<br>☐ Zika Virus - Single Check (Genekam Biotechnology AG)<br>☐ FTD Zika virus (Fast Track Diagnostics)<br>☐ VIASURE Zika Virus Real Time PCR Detection Kit (Certest Biotec)<br>☐ Zika Virus Real-TM (Sacace Biotechnologies)<br>☐ Logix Smart Zika Test Kit (Co-Diagnostics Inc)<br>☐ Genesig Kits for ZIKV (Primerdesign Ltd)<br>☐ Genesig Multiplex Kit for Dengue, Chikungunya and Zika Virus (Primerdesign Ltd)<br>☐ AccuPower ZIKV (DENV, CHIKV) Multiplex Real-Time RT-PCR Kit (Bioneer)<br>☐ VIASURE Zika, Dengue & Chikungunya Real Time PCR Detection Kit (Certest Biotec)<br>☐ FTD Zika/Dengue/Chik (Fast-Track Diagnostics)<br>☐ DiaPlexQ ZCD (ZIKV, CHIKV, DENV) Detection Kit (SolGent Co Ltd)<br>☐ TaqMan Zika Virus Triplex Kit (Thermo Fisher Scientific)<br>☐ GenoAmp Trioplex Real-Time RT-PCR Zika/Den/Chiku (Medical Innovation Ventures)<br>☐ Other
Describe procedure for in house ZIKV RT-PCR/PCR for ascertining infant infection	__________________________________
Specify other ZIKV RT-PCR/PCR brand name	__________________________________
Did the study obtain a measure of ZIKV viral load in infants?	◯ Yes<br>◯ No
ZIKV PCR/RT-PCR cut-off point in infants	____________________________

For peer review only

Was a negative control included with every run of ZIKV RT-PCR/PCRs?	◯ Yes<br>◯ No
Definition of POSITIVE ZIKV RT-PCR/PCR in infants	__________________________________
Definition of NEGATIVE ZIKV RT-PCR/PCR in infants	__________________________________
Definition of INCONCLUSIVE ZIKV RT-PCR/PCR in infants	__________________________________
Source of definition used to interpret ZIKV RT-PCR/PCR test results<br><br>Select all that apply	☐ Manufacturer<br>☐ Ministry of Health reference lab<br>☐ WHO (global or local office e.g: PAHO)<br>☐ US-CDC<br>☐ Consensus/study researchers<br>☐ Other
Specify other source of definition used to interpret ZIKV RT-PCR/PCR test results	____________________________
Where was the ZIKV RT-PCR/PCR test performed for samples from infants?<br><br>Select all that apply	☐ Lab at the same health care facility where study is conducted<br>☐ Study-specific lab<br>☐ Ministry of Health reference lab<br>☐ Private external lab<br>☐ Other
Specify other lab where ZIKV RT-PCR/PCR test was performed for samples from infants	____________________________
Specify any additional inclusion or exclusion criteria for determining which samples from infants to test for ZIKV using RT-PCR/PCR	__________________________________
How many ZIKV PCRs were conducted with infants?	____________________________
Would your study be able to provide a Ct value for infant ZIKV PCRs to inform the measurement error models?	◯ Yes<br>◯ No

For peer review only

Changes in diagnosis of infant ZIKV infection over time

Which clinical criteria did you apply initially to diagnose infant infection? (Clinical Definition 1)

____

Did your clinical criteria for infant infection change over time?

◯ No
◯ Yes - changed 1x
◯ Yes - changed more than 1x

If your clinical criteria changed over time, which criteria did you apply subsequently to assess infant infection? (Clinical Definition 2)

____

When did you begin using the revised clinical criteria for assessing infant infection? (Clinical Definition 2)

____

What was the third set of criteria you applied to assess infant infection? (Clinical Definition 3)

____

When did you begin using the third set of clinical criteria? (Clinical Definition 3)

____

Which imaging criteria did you apply initially to diagnose infant infection? (Clinical Definition 1)

____

Did your imaging criteria for infant infection change over time?

◯ No
◯ Yes - changed 1x
◯ Yes - changed more than 1x

If your imaging criteria changed over time, which criteria did you apply subsequently to assess infant infection? (Clinical Definition 2)

____

When did you begin using the revised imaging criteria for assessing infant infection? (Clinical Definition 2)

____

What was the third set of imaging criteria you applied to assess infant infection? (Clinical Definition 3)

____

When did you begin using the third set of imaging criteria? (Clinical Definition 3)

____

What laboratory criteria was used initially to diagnose infant ZIKV infection? (Laboratory Definition 1)

____

Did your laboratory criteria for diagnosing infant infection change over time?

◯ No
◯ Yes - changed 1x
◯ Yes - changed more than 1x

For peer review only

Which laboratory criteria did you apply subsequently? (Laboratory Definition 2)	__________________________________
When did you begin using the revised lab criteria for infant infection? (Laboratory Definition 2)	__________________________
What was the third set of laboratory criteria you applied for diagnosing infant infection? (Laboratory Definition 3)	__________________________________
When did you begin using the third set of lab criteria for infant infection? (Laboratory Definition 3)	__________________________
Were infant samples retested using the new laboratory criteria?	__________________________________

For peer review only

ZIKV testing algorithm & test interpretation - infant ZIKV infection

Describe the testing algorithm for defining a confirmed ZIKV+ test in an infant

____

Describe the testing algorithm for defining a confirmed ZIKV- test in an infant

____

What tests were used to confirm ZIKV infection in an infant?

Select all that apply

- ☐ ZIKV IgM - 1 sample
- ☐ ZIKV IgM - paired samples (seroconversion or increased titers)
- ☐ ZIKV IgM (1 sample) + IgG
- ☐ ZIKV IgM (paired samples) + IgG
- ☐ RT-PCR or other NAAT
- ☐ PRNT > 10
- ☐ Other

Specify other test or combination of tests used to confirm ZIKV infection in an infant

____

Considering any relevant clinical and laboratory criteria, what was the study's definition of a CONFIRMED infant ZIKV Infection

____

Considering any relevant clinical and laboratory criteria, what was the study's definition of a PROBABLE infant ZIKV Infection

____

Considering any relevant clinical and laboratory criteria, what was the study's definition of a NEGATIVE/UNLIKELY infant ZIKV Infection

____

Source of ZIKV test result categorization

Select all that apply

- ☐ Manufacturer
- ☐ Ministry of Health reference lab
- ☐ WHO (global or local office e.g: PAHO)
- ☐ Consensus/study researchers
- ☐ Other

Specify other source of ZIKV test result categorization

____

For peer review only

ZIKV Diagnostics external quality assessment (EQA)

Did your study conduct ZIKV test-related external quality assessment (EQA)?	◯ Yes<br>◯ No
Where was the EQA conducted?	◯ Lab at the same health care facility where study is conducted<br>◯ Study-specific lab<br>◯ Ministry of Health reference lab<br>◯ Private external lab<br>◯ Other
Where else was the EQA conducted?	__________________________
Which group conducted the EQA?	__________________________
Which tests were evaluated in the EQA?<br><br>Select all that apply	☐ ZIKV ELISA<br>☐ ZIKV PCR/RT-PCR<br>☐ Other
Which other tests were evaluated in the EQA?	__________________________________
Which assay or assays was/were used as a comparator for the ELISA EQA?	__________________________________
Sensitivity of ZIKV ELISA test	__________________________<br>(Provide as percentage)
Specificity of ZIKV ELISA test	__________________________<br>(Provide as percentage)
PPV of the ZIKV ELISA test	__________________________<br>(Provide as percentage)
ZIKV prevalence used to estimate the PPV	__________________________
Sample (N) for ELISA EQA	__________________________
How many ELISAs, either IgG or IgM, were included in the EQA?	__________________________
Which assay or assays was/were used as a comparator for the PCR/RT-PCR EQA?	__________________________________

For peer review only

Sensitivity of ZIKV PCR/RT-PCR test	______________ (Provide as percentage)
Specificity of ZIKV PCR/RT-PCR test	______________ (Provide as percentage)
PPV of the ZIKV PCR/RT-PCR test	______________ (Provide as percentage)
ZIKV prevalence used to estimate the PPV	______________
How many molecular tests (PCR/RT-PCR) were included in the EQA?	______________
Describe the procedure used to resolve inconsistencies in the EQA	______________
Was a positive control from the outbreak included in the EQA?	◯ Yes<br>◯ No
Was a negative control from the outbreak included in the EQA?	◯ Yes<br>◯ No
Please describe any additional details of the EQA	______________
List any publications related to the EQA described above	______________

For peer review only

Perinatal Outcomes

How did the study measure gestational age at birth?

◯ last menstrual period (LMP)
◯ ultrasound
◯ LMP if ultrasound unavailable, otherwise ultrasound
◯ Other
◯ Study did not measure gestational age at birth

Describe other means of ascertaining gestational age at birth

____

Did the study include a newborn maturity assessment?

Select all that apply

☐ Ballard
☐ Dubowitz
☐ Capurro
☐ Other
☐ No, study did not include newborn maturity assessment

Specify other maturity assessment of newborn

____

How did the study determine preterm birth?

Select all that apply

☐ Gestational age - from ultrasound
☐ Gestational age - from LMP
☐ Gestational age from LMP if ultrasound not available, otherwise from ultrasound
☐ Maturity assessment
☐ Other
☐ Study did not assess preterm birth

Specify other determination of preterm birth

____

How did the study define preterm birth?

____
((e.g. extremely preterm (< 28 weeks); very preterm (28-32 weeks), moderate to late preterm (32-37 weeks)))

How did the study determine termination of pregnancy (TOP)/induced abortion?

◯ Self report
◯ Medical records
◯ Other
◯ Study did not measure TOP/induced abortion

Specify other means of determining termination of pregnancy/induced abortion

____

Provide wording of question related to TOP/induced abortion for self report or provide directions for extracting TOP-related information from medical chart

____

How did the study define miscarriage?

◯ Pregnancy loss < 20 weeks regardless of weight
◯ Pregnancy loss < 20 weeks or weight < 500 gr
◯ Involuntary loss < 20 weeks or voluntary loss between 10-20 weeks (Cuban definition)
◯ Other
◯ Study did not measure miscarriage

For peer review only

Specify other definition of miscarriage	__________________
How did the study define fetal loss or fetal death?	◯ Any loss < 20 weeks regardless of weight<br>◯ Any loss < 22 weeks or weight < 500 gr<br>◯ Early (20-27 weeks) and late (>28 weeks) fetal loss regardless of weight<br>◯ Other<br>◯ Study did not measure fetal loss
Specify other definition of fetal loss or fetal death	__________________
Did the study document placental insufficiency as an outcome?	◯ Yes<br>◯ No
Definition of placental insufficiency	__________________
Did the study document intrauterine growth restriction (IGR) as an outcome?	◯ Yes<br>◯ No
Definition of intrauterine growth restriction	__________________
Did the study document low birth weight (LBW) as an outcome?	◯ Yes<br>◯ No
How did the study define low birthweight?	__________________
How did the study define very low birthweight?	__________________
How did the study define extremely low birthweight?	__________________

For peer review only

Postnatal ZIKV-related imaging & microcephaly

Were postnatal cranial ultrasounds performed?
◯ Yes
◯ No

How many postnatal cranial ultrasounds did the study plan to conduct with each infant/child?
____

Age (months) when the study planned to conduct the postnatal cranial ultrasounds
____
(e.g., 1, 3, 6 months)

Postnatal cranial ultrasound machine models (list all that apply)
____
(Write "same as above" if ultrasound machine models are same as those described in prenatal ultrasound section.)

Place where postnatal cranial ultrasound(s) were performed

Select all that apply
☐ Same health care facility where study is taking place
☐ Study-specific site (not a health care facility)
☐ Private/external site
☐ Other

Other place that the postnatal cranial ultrasound(s) were performed
____

Training received by ultrasound technician performing postnatal cranial ultrasounds
____
(Write "same as above" if technician training same as those described in prenatal ultrasound section.)

Quality control procedures for ultrasound evaluation of abnormalities/head circumference
____
(Write "same as above" if quality control measures same as those described in prenatal ultrasound section.)

What other types of postnatal ultrasound did the study conduct?

Select all that apply
☐ Thoracic
☐ Abdominal
☐ Pelvic
☐ Osteo muscular
☐ Other
☐ No other types of postnatal ultrasound

Specify other type of additional postnatal ultrasound
____

Did the study perform postnatal brain/head/neck MRIs?
◯ Yes
◯ No

How many postnatal brain/head/neck MRIs did the study plan to conduct with each infant/child?
____

For peer review only

What other types of postnatal MRI did the study conduct?<br><br>Select all that apply	☐ Thoracic<br>☐ Abdominal<br>☐ Pelvic<br>☐ Osteo muscular<br>☐ Other<br>☐ No other types of postnatal MRI
Specify other type of additional postnatal MRI	__________________________________
Age (months) when the study planned to conduct any postnatal MRI	__________________________________<br>(e.g. 1, 3, 6, 9 months)
Describe any conditions for application of any postnatal MRI	__________________________________
Place where postnatal MRI was performed	◯ Same health care facility where study is taking place<br>◯ Study-specific site (not a health care facility)<br>◯ Private/external site
Training received by postnatal MRI technician	__________________________________<br>(Write "same as above" if technician training same as that described in prenatal MRI section.)
Quality control for evaluation of postnatal MRI	__________________________________<br>(Write "same as above" if quality control measures same as those described in prenatal MRI section.)
Was postnatal microcephaly an outcome in this study?	◯ Yes<br>◯ No
At what age(s) (in months) was postnatal microcephaly assessed?	__________________________________<br>(e.g., 1, 3, 6 months)
How was postnatal microcephaly diagnosed?<br><br>Select all that apply	☐ Presence vs absence<br>☐ By grades (normocephaly vs microcephaly vs severe microcephaly)<br>☐ By percentiles<br>☐ By Z-score<br>☐ By diagnostic certainty criteria<br>☐ Other
Specify other postnatal microcephaly diagnosis type	__________________________________

For peer review only

How did the study define postnatal microcephaly?	◯ >2 SD below the mean<br>◯ < 3 SD below the mean<br>◯ 3 SD below the mean<br>◯ >3 SD below the mean<br>◯ below the 5th percentile<br>◯ below the 10th percentile<br>◯ Other
Specify other definition of postnatal microcephaly	__________________________________
How did the study define postnatal microcephaly?	◯ Level 1 of diagnostic certainty<br>◯ Level 2A of diagnostic certainty<br>◯ Level 2B of diagnostic certainty<br>◯ Level 3A of diagnostic certainty<br>◯ Level 3B of diagnostic certainty<br>◯ Level 4 of diagnostic certainty<br>◯ Other
Specify other definition of postnatal microcephaly	____________________________
Did the study measure infant head circumference	◯ Yes<br>◯ No
Device used to measure infant head circumference<br><br>Select all that apply	☐ Flexible-non-stretchable measuring tape-marked by 0.1 cm increments<br>☐ Metal tape<br>☐ Paper tape<br>☐ Other<br>☐ Unknown
Specify other device used to measure infant head circumference	__________________________________
Reference standards used to calculate infant head circumference Z score<br><br>Select all that apply	☐ Intergrowth<br>☐ Ministry of Health standards<br>☐ WHO or PAHO standard<br>☐ Local reference standard<br>☐ Consensus/study researchers<br>☐ Other
Other reference standards used to calculate infant head circumference Z score	____________________________
Was epilepsy screening performed?	◯ Yes<br>◯ No
How did the study define epilepsy?	__________________________________
How many times did the study plan to screen the infant or child for epilepsy?	____________________________

For peer review only

List ages (in months) at which the study planned to screen for epilepsy	__________________________________
Was postnatal electroencephalography (EEG) performed?	◯ Yes<br>◯ No
How many EEGs did the study plan to conduct with each infant or child?	__________________________________
Were the EEGs reviewed for abnormalities by epileptologists who were blinded to the ZIKV status of the mother?	◯ Yes<br>◯ No

For peer review only

Ascertainment of congenital Zika syndrome (CZS)

Did the study measure congenital Zika syndrome (CZS)?
◯ Yes
◯ No

When did the study assess CZS?

Select all that apply
☐ Antenatal
☐ Peri/intrapartum
☐ Postpartum

When, during pregnancy, did the study assess antenatal CZS?

Select all that apply
☐ 1st trimester
☐ 2nd trimester
☐ 3rd trimester

Time of CZS assessment in hours after delivery for peri/intrapartum assessment
____
(Please list all times, separated by a comma)

Time of CZS assessment in days after delivery for postpartum diagnosis of CZS
____
(Please list all times, separated by a comma)

Which criteria were used to assess CZS?

Select all that apply
☐ maternal clinical
☐ maternal laboratory
☐ placental laboratory
☐ fetal clinical
☐ fetal laboratory
☐ fetal neuroimaging
☐ infant clinical
☐ infant laboratory
☐ infant neuroimaging
☐ developmental
☐ other

Which other criteria were used to assess CZS?
____

Infant or fetal neurologic alterations used to assess CZS

Select all that apply
☐ Epilepsy
☐ Seizures
☐ Dysphagia
☐ Hypertonia
☐ Hypotonia
☐ Hyperreflexia
☐ Hyporeflexia
☐ Other neurologic abnormality

Which other infant or fetal neurological criteria were used to assess CZS?
____

Infant or fetal structural abnormalities used to assess CZS

Select all that apply
☐ Cranial shape malformation
☐ Microcephaly
☐ Abnormalities of intracranial anatomy
☐ Musculoskeletal defects
☐ Eye defects
☐ Hearing (ENT) defects
☐ Other structural abnormality

For peer review only

Which other infant or fetal structural criteria were considered in assessing CZS?

____

What type of microcephaly diagnosis was used to diagnose CZS?

Select all that apply

- ☐ Antenatal microcephaly
- ☐ Postnatal microcephaly
- ☐ Both antenatal and postnatal microcephaly
- ☐ Other

What other type of microcephaly diagnosis was used to diagnose CZS?

____

Which intracranial abnormalities were considered in assessing CZS?

Select all that apply

- ☐ Diffuse, primarily subcortical calcifications
- ☐ Increased fluid spaces - ventricular and extra-axial
- ☐ Marked cortical thinning with abnormal gyral patterns
- ☐ Hypoplasia or absence of corpus callosum
- ☐ Anencephaly
- ☐ Holoprosencephaly
- ☐ Decreased myelination
- ☐ Cerebellar or cerebellar vermis hypoplasia
- ☐ Calcifications in basal ganglion/brainstem
- ☐ Other

What other intracranial abnormalities were considered in assessing CZS?

____

Maternal clinical criteria used to assess CZS

- ○ Fever only
- ○ Rash only
- ○ Fever + rash
- ○ Fever or rash
- ○ Fever + rash + joint pain
- ○ Fever + rash + joint pain + conjunctivitis
- ○ Other
- ○ Fever + rash + other

Which other maternal clinical criterion or combination of criteria were used to assess CZS

____

Fetal clinical criteria used to assess CZS

- ○ Fever only
- ○ Rash only
- ○ Fever + rash
- ○ Fever or rash
- ○ Fever + rash + joint pain
- ○ Fever + rash + joint pain + conjunctivitis
- ○ Other
- ○ Fever + rash + other

Which other fetal clinical criterion or combination of criteria were used to assess CZS

____

For peer review only
For peer review only - http://bmjopen.bmj.com/site/about/guidelines.xhtml

Infant clinical criteria used to assess CZS	◯ Fever only<br>◯ Rash only<br>◯ Fever + rash<br>◯ Fever or rash<br>◯ Fever + rash + joint pain<br>◯ Fever + rash + joint pain + conjunctivitis<br>◯ Other<br>◯ Fever + rash + other
Which other infant clinical criterion or combination of criteria were used to assess CZS	__________________________________
Maternal laboratory diagnostics used to assess CZS<br><br>Select all that apply	☐ Molecular diagnosis (e.g. RT-PCR, other NAAT)<br>☐ Antibody-based immunoassay (e.g. ELISA, IFA, PRNT, microneutralization)<br>☐ Antigen-based immunoassay (e.g. ELISA, IFA)<br>☐ Rapid diagnostic test (RDT)<br>☐ Other
Which other maternal laboratory diagnostics were used to assess CZS?	____________________________
Placental laboratory diagnostics used to assess CZS<br><br>Select all that apply	☐ Molecular diagnosis (e.g. RT-PCR, other NAAT)<br>☐ Antibody-based immunoassay (e.g. ELISA, IFA, PRNT, microneutralization)<br>☐ Antigen-based immunoassay (e.g. ELISA, IFA)<br>☐ Rapid diagnostic test (RDT)<br>☐ Other
Which other placental laboratory diagnostics were used to assess CZS?	____________________________
Fetal laboratory diagnostics used to assess CZS<br><br>Select all that apply	☐ Molecular diagnosis (e.g. RT-PCR, other NAAT)<br>☐ Antibody-based immunoassay (e.g. ELISA, IFA, PRNT, microneutralization)<br>☐ Antigen-based immunoassay (e.g. ELISA, IFA)<br>☐ Rapid diagnostic test (RDT)<br>☐ Other
Which other fetal laboratory diagnostics were used to assess CZS?	____________________________
Infant laboratory diagnostics used to assess CZS<br><br>Select all that apply	☐ Molecular diagnosis (e.g. RT-PCR, other NAAT)<br>☐ Antibody-based immunoassay (e.g. ELISA, IFA, PRNT, microneutralization)<br>☐ Antigen-based immunoassay (e.g. ELISA, IFA)<br>☐ Rapid diagnostic test (RDT)<br>☐ Other
Which other infant laboratory diagnostics used to assess CZS?	____________________________
Fetal neuroimaging used to assess CZS<br><br>Select all that apply	☐ Ultrasound<br>☐ MRI<br>☐ CT scan<br>☐ Other

For peer review only

Specify other neuroimaging used to assess CZS in fetus	__________________________________
Infant neuroimaging used to assess CZS<br><br>Select all that apply	☐ Ultrasound<br>☐ MRI<br>☐ CT scan<br>☐ Other
Specify other neuroimaging used to assess CZS in infant	__________________________________
Which types of developmental delays or impairments were included in the CZS assessment?<br><br>Select all that apply	☐ motor<br>☐ cognitive<br>☐ speech<br>☐ other
Which other types of developmental delays or impairments were included in the CZS assessment?	__________________________________
Did your study apply the following criteria for CZS phenotype?<br><br>Severe microcephaly with partially collapsed skull Thin cerebral cortices with subcortical calcifications Macular scarring and focal pigmentary retinal mottling Congenital contractures Marked early hypertonia and symptoms of extrapyramidal involvement	◯ Yes<br>◯ No
Reference standard used to define CZS	◯ WHO guidelines 2015<br>◯ WHO guidelines 2016<br>◯ WHO guidelines 2017<br>◯ Current country guidelines (Ministry of Health)<br>◯ Study definition<br>◯ Other
Specify other reference standards used to define CZS	__________________________________
Specify study definition used to define CZS	__________________________________
Study's definition of a CONFIRMED case of CZS	__________________________________
Study's definition of a PROBABLE case of CZS	__________________________________
Study's definition of an UNLIKELY case of CZS	__________________________________

For peer review only

Genetic abnormalities

Did the study conduct prenatal genetic screening or testing?	◯ Yes<br>◯ No
Did the study conduct genetic testing on products of conception in cases of termination or fetal loss?	◯ Yes<br>◯ No
How was prenatal genetic screening conducted?<br><br>Select all that apply	☐ FTS (first trimester screening, standard or enhanced, maternal blood screen + ultrasound)<br>☐ only blood screening (maternal serum screening)<br>☐ only ultrasound findings (including Nuchal Translucency Ultrasound)<br>☐ cfDNA/NIPT (Non-invasive prenatal testing)<br>☐ Amniocentesis<br>☐ chorionic villus sampling<br>☐ Triple screen (with AFP)<br>☐ Quad screen (with AFP)<br>☐ other
What other type of genetic screening was conducted?	__________________________________
What type of prenatal genetic testing was conducted?<br><br>Select all that apply	☐ cfDNA/NIPT<br>☐ FISH<br>☐ karyotype<br>☐ chromosomal microarray<br>☐ single gene<br>☐ gene panel<br>☐ other
What other type of genetic testing was conducted?	__________________________________

For peer review only

Maternal and infant C reactive protein

Did the study collect a measure of maternal C-reactive protein	◯ Yes<br>◯ No
Was C-reactive protein in the mother reported as a binary or continuous measure?	◯ binary<br>◯ continuous
What was the cut off for the binary measure of C-reactive protein in the mother?	__________________________________
How many times did the study plan to measure C-reactive protein for each pregnant woman?	____
Did the study collect a measure of infant C-reactive protein?	◯ Yes<br>◯ No
Was C-reactive protein in the infant reported as a binary or continuous measure?	◯ binary<br>◯ continuous
What was the cut off for the binary C-reactive protein measure in the infant?	__________________________________
How many times did the study plan to measure C-reactive protein for each infant?	____
At what ages did the study plan to measure C-reactive protein for each infant?	____
How was C-reactive protein measured in the infant?	◯ Immunoturbidimetric<br>◯ Nephelometry

For peer review only

Ophthalmological exams

Did the study measure ocular abnormalities?	◯ Yes<br>◯ No
How did the study measure ocular abnormalities?<br><br>Select all that apply	☐ fundoscopic exam<br>☐ RetCam<br>☐ other
What other exam did the study use to measure ocular abnormalities?	__________________________________
Type of eye abnormalities measured in the study<br><br>Select all that apply	☐ Macular findings: Focal pigmentary mottling<br>☐ Macular findings: Chorioretinal atrophy<br>☐ Optic nerve findings: Optic nerve hypoplasia<br>☐ Optic nerve findings: Optic nerve cupping<br>☐ Optic nerve findings: Optic nerve atrophy<br>☐ Other retinal lesions: subretinal hemorrhage<br>☐ Hypopigmentation<br>☐ Vascular tortuosity<br>☐ Abnormal termination of the vessels<br>☐ Focal vascular dilation<br>☐ Congenital glaucoma<br>☐ Iris colobomas<br>☐ Microphthalmia<br>☐ Cataracts<br>☐ Subluxation of the lens<br>☐ Intraocular calcification<br>☐ Other
Specify other eye abnormalities measured by the study	__________________________________
Were any of the following exams performed?<br><br>Select all that apply	☐ Cardiff acuity test<br>☐ Cardiff visual contrast test<br>☐ Teller test<br>☐ Other
Specify any other visual acuity tests performed by the study	__________________________________
At what age(s) did the study plan to measure ophthalmological function?	__________________________________
Reference standard used for evaluation of fundoscopic exam<br><br>Select all that apply	☐ Provided by manufacturer<br>☐ In-hospital standard<br>☐ Regional or state standard<br>☐ Federal standard<br>☐ WHO or local branch<br>☐ Other
Specify other basis for evaluation of fundoscopic exam	__________________________________

For peer review only

Reference standard used for evaluation of RetCam exam<br><br>Select all that apply	☐ Provided by manufacturer<br>☐ In-hospital standard<br>☐ Regional or state standard<br>☐ Federal standard<br>☐ WHO or local branch<br>☐ Other
Specify other basis for evaluation of RetCam opthamological exam	__________________________

For peer review only

Auditory abnormalities

Did the study measure auditory abnormalities?	◯ Yes<br>◯ No
How did the study measure auditory abnormalities?<br><br>Select all that apply	☐ impedance audiometry<br>☐ otoacoustic emissions (OAEs)<br>☐ other
How else did the study measure auditory abnormalities?	__________________________________
Was impedance audiometry performed at birth or within the first 24-48 hours after birth?	◯ Yes<br>◯ No
Type of information used to assess auditory abnormalities	◯ Presence vs absence of alterations<br>◯ Severity of nerve damage<br>◯ Other
Specify other type of information used to assess auditory abnormalities	____
At what age(s) did the study plan to measure auditory function?	__________________________________
Did the study perform an Auditory Brainstem Response (ABR) test?	◯ Yes<br>◯ No
How many times did the study plan to perform ABR on each infant/child?	__________________________________
At what ages did the study perform ABR?	__________________________________
Specify any other auditory tests performed by study	__________________________________
Reference standard used for evaluation of impedance audiometry exam<br><br>Select all that apply	☐ Provided by manufacturer<br>☐ In-hospital standard<br>☐ Regional or state standard<br>☐ Federal standard<br>☐ WHO or local branch<br>☐ Other
Specify other reference standard used	____
Reference standard used for evaluation of OAE<br><br>Select all that apply	☐ Provided by manufacturer<br>☐ In-hospital standard<br>☐ Regional or state standard<br>☐ Federal standard<br>☐ WHO or local branch<br>☐ Other

For peer review only

Specify other reference standard used

For peer review only

Additional abnormalities

In addition to the ocular and auditory outcomes described previously, which other infant outcomes were assessed by the study?<br><br>Select all that apply	☐ Motor abnormalities (hypotonia, hypertonia, hyperreflexia, spasticity, clonus, extrapyramidal symptoms)<br>☐ Congenital contractures (arthrogryposis, uni or bilateral clubfoot)<br>☐ Craniofacial disproportion<br>☐ Seizures, epilepsy<br>☐ Postnatal intraventricular hemorrhage<br>☐ Other non-neurologic congenital abnormalities
How were congenital contractures defined?	__________________________________
Which other non-neurologic congenital abnormalities were assessed by the study?	__________________________________

For peer review only

Developmental screening tools

Study administered the Bayley Scales of Infant Development (BSID)?	◯ Yes<br>◯ No
Who administered the Bayleys?	◯ Pediatrician<br>◯ Neuropediatrician<br>◯ Other
Who else administered the Bayleys?	__________________________________
Describe the training of the person who administered the Bayleys	____
Was the person evaluating the Bayleys blinded to ZIKV status of the mother?	◯ Blinded<br>◯ Unblinded
How many times did the study plan to administer the Bayleys with each infant or child?	__________________________________
List ages (in months) at which the study planned to administer the Bayleys:	__________________________________
Did the study assess all 5 domains measured by the Bayleys? (i.e., receptive language, expressive language, fine motor, gross motor, and cognitive)	◯ Yes<br>◯ No
If all 5 domains were not assessed, which of the Bayleys scales were administered?<br><br>Select all that apply	☐ Receptive language<br>☐ Expressive language<br>☐ Fine motor<br>☐ Gross motor<br>☐ Cognitive
Study administered the Ages and Stages Questionnaire (ASQ)?	◯ Yes<br>◯ No
Was the person administering the ASQ blinded to the ZIKV status of the mother?	◯ Blinded<br>◯ Unblinded
How many times did the study plan to administer the ASQ with each infant or child?	__________________________________
List all ages (in months) at which the study planned to adminster the ASQ	__________________________________
Study administered the Warner Initial Developmental Evaluation of Adaptive and Functional Skills (WIDEA-FS)?	◯ Yes<br>◯ No
Was the person administering the WIDEA-FS blinded to the ZIKV status of the mother?	◯ Blinded<br>◯ Unblinded
How many times did the study plan to administer the WIDEA-FS with each child?	__________________________________

For peer review only

List all ages (in months) at which the study planned to adminster the WIDEA-FS	__________________
Study administered the Alberta Infant Motor Skills (AIMS) assessment?	◯ Yes<br>◯ No
Describe how the person evaluating the AIMS was trained	__________________
Who administered the AIMS?	◯ Pediatrician<br>◯ Neuropediatrician<br>◯ Other
Who else administered the AIMS?	__________________
Was the person administering the AIMS blinded to the ZIKV status of the mother?	◯ Blinded<br>◯ Unblinded
How many times did the study plan to administer the AIMS with each infant or child?	__________________
List all ages (in months) at which the study planned to adminster the AIMS	__________________
Study administered the Oxford Neurodevelopmental Assessment (Ox-NDA)?	◯ Yes<br>◯ No
Describe the training of the person administering the Ox-NDA	__________________
Was the person administering the Ox-NDA blinded to the ZIKV status of the mother?	◯ Blinded<br>◯ Unblinded
How many times did the study plan to administer the Ox-NDA with each infant or child?	__________________
List all ages (in months) at which the study planned to adminster the Ox-NDA	__________________
Study administered the Intergrowth-21st Neurodevelopmental Assessment (INTER-NDA)?	◯ Yes<br>◯ No
Describe the training of the person administering the INTER-NDA	__________________
Was the person administering the INTER-NDA blinded to the ZIKV status of the mother?	◯ Blinded<br>◯ Unblinded
List all ages (in months) at which the study planned to adminster the INTER-NDA	__________________

For peer review only

In addition to the instruments mentioned above, which of the following developmental screening tools did the project administer?<br><br>Select all that apply	☐ BDI (Batelle Developmental Inventory) 0-7 yrs<br>☐ BITSEA/ITSEA (Brief) Infant-Toddler Social and Emotional Assessment 1-3 yrs<br>☐ CDI (MacArthur-Bates Communication Development Inventory), 8-30 months<br>☐ DDST or DENVER II (Denver Developmental Screening Test) 0-6 yrs<br>☐ DECA (Devereaux Early Childhood Assessment) 1 months-6 years<br>☐ GMFCS (Gross Motor Function Classification System) 0-18 yrs<br>☐ Greenspan Social-Emotional Growth Chart 0-42 months<br>☐ HELP (Hawaii Early Learning Profile) 0-3 yrs<br>☐ HOME (Home Observation Measurement of the Environment) 0 and up<br>☐ MSEL (Mullen Scales of Early Learning) 0-7 yrs<br>☐ VABS (Vineland Adaptive Behavior Scales) 0 and up<br>☐ PDDST (Pervasive Developmental Disorders Screening Test) 1-4 yrs<br>☐ PEDI (Pediatric Evaluation of Disability Inventory) 6 months-7.5 yrs<br>☐ Other<br>☐ No additional infant development screening tools were administered by the project
Specify other infant developmental screening tools administered by project	__________________________________
Did the project administer any of the following development screening tools for toddlers and older?<br><br>Select all that apply	☐ BSRA (Bracken School Readiness Assessment) 2.6 years and up<br>☐ CBCL (Achenbach Child Behavior Checklist) 6-18 years<br>☐ SDQ (Strengths and Difficulties Questionnaire) 3-16 years<br>☐ Other<br>☐ No other toddler-and-above development screening tools were administered by the project than those mentioned previously
Specify other toddler developmental screening tools administered by project	__________________________________

For peer review only

Sociodemographic variables and maternal mental health

Did the study measure maternal ethnicity or minority status?	◯ Yes, self defined<br>◯ Yes, interviewer or study defined<br>◯ Yes, as reported in participant medical record<br>◯ No, not measured
Did the study measure household SES?	◯ Yes, household income (self reported)<br>◯ Yes, country-level measure of SES (self reported)<br>◯ Yes, country-level measure of SES (verified)<br>◯ Other<br>◯ No, not measured
Specify other measurement of household SES	__________________________________
Did the study measure maternal education?	◯ Yes<br>◯ No
Did the study measure maternal occupation?	◯ Yes<br>◯ No
Did the study measure maternal marital status?	◯ Yes<br>◯ No
List any maternal mental health scales that were administered by the study<br><br>Select all that apply	☐ BAI (Beck Anxiety Inventory)<br>☐ BDI (Beck Depression Inventory)<br>☐ BDI-II (Beck Depression Inventory II)<br>☐ CBS (Caregiver Burden Scale)<br>☐ CES-D (Center for Epidemiologic Studies Depression Scale)<br>☐ DASS-21 (Depression, Anxiety, and Stress Scale - Short Form)<br>☐ Patient Health Questionnaire-9 (PHQ-9)<br>☐ Patient Health Questionnaire-2 (PHQ-2)<br>☐ Edinburgh Postnatal Depression Scale (EPDS)<br>☐ MSPSS (Multidimensional Scale of Perceived Social Support)<br>☐ PSI (Parenting Stress Index)<br>☐ PSI-SF (Parenting Stress Index - Short Form)<br>☐ WHOQOL-BREF (WHO Quality of Life)<br>☐ Other<br>☐ No maternal mental health or parenting stress scales were administered by the project
Specify other maternal mental health screening tools administered by the study	__________________________________

For peer review only

Pre-pregnancy-related covariates	
Did the study measure pre-pregnancy maternal exposure to clinically suspected arboviruses, such as dengue and chikungunya, with specific or proxy dates?	◯ Yes, self reported<br>◯ Yes, medical/clinical chart<br>◯ No, not measured
Did the study measure pre-pregnancy maternal exposure to workplace teratogens?	◯ Yes, self reported<br>◯ Yes, medical/clinical chart<br>◯ No, not measured
Did the study measure pre-pregnancy exposure to intimate partner violence?	◯ Yes, self reported<br>◯ Yes, medical/clinical chart<br>◯ No, not measured
Did the study measure number of previous live births (parity)?	◯ Yes, self reported<br>◯ Yes, medical/clinical chart<br>◯ No, not measured
Did the study measure the number of previous total pregnancies (gravidity)?	◯ Yes, self reported<br>◯ Yes, medical/clinical chart<br>◯ No, not measured
Did the study measure the number of previous pregnancies with any complications	◯ Yes, self reported<br>◯ Yes, medical/clinical chart<br>◯ No, not measured
Did the study measure the number of previous term pregnancies?	◯ Yes, self reported<br>◯ Yes, medical/clinical chart<br>◯ No, not measured
Did the study measure the number of previous preterm pregnancies?	◯ Yes, self reported<br>◯ Yes, medical/clinical chart<br>◯ No, not measured
Did the study measure the number of previous induced or spontaneous abortions?	◯ Yes, self reported<br>◯ Yes, medical/clinical chart<br>◯ No, not measured
Did the study measure the number of previous stillbirths?	◯ Yes, self reported<br>◯ Yes, medical/clinical chart<br>◯ No, not measured
Did the study measure the number of previous pregnancies with preterm labor?	◯ Yes, self reported<br>◯ Yes, medical/clinical chart<br>◯ No, not measured
Did the study measure the number of previous pregnancies with eclampsia?	◯ Yes, self reported<br>◯ Yes, medical/clinical chart<br>◯ No, not measured
Did the study measure the number of previous pregnancies with preeclampsia?	◯ Yes, self reported<br>◯ Yes, medical/clinical chart<br>◯ No, not measured
Did the study measure the number of previous pregnancies with gestational diabetes?	◯ Yes, self reported<br>◯ Yes, medical/clinical chart<br>◯ No, not measured

For peer review only

Did the study measure the number of previous pregnancies with birth defects?	◯ Yes, self reported<br>◯ Yes, medical/clinical chart<br>◯ No, not measured
Did the study measure history of pregnancies with fetus or infant affected by genetic disorders and/or congenital abnormalities?	◯ Yes, self reported<br>◯ Yes, medical/clinical chart<br>◯ No, not measured

For peer review only

Current pregnancy

Did the study measure complications related to the current pregnancy	◯ Yes, self reported<br>◯ Yes, medical/clinical chart<br>◯ No, not measured
Did the study measure gestational diabetes?	◯ Yes<br>◯ No
Did the study measure maternal body mass index (BMI)?	◯ Yes<br>◯ No

For peer review only

Ascertainment of maternal exposure to other infectious diseases

How did the study determine maternal HIV status?<br><br>Select all that apply	☐ Self report<br>☐ Medical records<br>☐ Diagnostic test<br>☐ Other<br>☐ Not measured
Specify other means of determining maternal HIV status	__________________________________
When was maternal HIV status determined?<br><br>Select all that apply	☐ 1st term<br>☐ 2nd term<br>☐ 3rd term<br>☐ whenever symptoms were present (per clinical indication)<br>☐ other
At what other time was maternal HIV status determined?	__________________________________
What diagnostic tests were applied to assess maternal HIV infection status?<br><br>Select all that apply	☐ serology<br>☐ RT-PCR<br>☐ other NAAT<br>☐ rapid diagnostic test (RDT)<br>☐ other
Which other diagnostic tests were applied to assess maternal HIV infection status?	__________________________________
How did the study determine maternal malaria infection (fever, placental parasitemia)?<br><br>Select all that apply	☐ Self report<br>☐ Medical records<br>☐ Diagnostic test<br>☐ Other<br>☐ Not measured
Specify other means of determining malaria infection	__________________________________
When was maternal malaria status determined?<br><br>Select all that apply	☐ 1st term<br>☐ 2nd term<br>☐ 3rd term<br>☐ whenever symptoms were present (per clinical indication)<br>☐ other
At what other time was maternal malaria status determined?	__________________________________
How did the study determine maternal parvovirus infection?<br><br>Select all that apply	☐ Self report<br>☐ Medical records<br>☐ Diagnostic test<br>☐ Other<br>☐ Not measured
Specify other means of determining parvovirus infection	__________________________________

For peer review only

When was maternal parvovirus status determined?<br><br>Select all that apply	☐ 1st term<br>☐ 2nd term<br>☐ 3rd term<br>☐ whenever symptoms were present (per clinical indication)<br>☐ other
At what other time was maternal parvovirus status determined?	__________________________
How did the study determine maternal listeria infection (fever, placental parasitemia)?<br><br>Select all that apply	☐ Self report<br>☐ Medical records<br>☐ Diagnostic test<br>☐ Other<br>☐ Not measured
Specify other means of determining listeria infection	__________________________
When was maternal listeria status determined?<br><br>Select all that apply	☐ 1st term<br>☐ 2nd term<br>☐ 3rd term<br>☐ whenever symptoms were present (per clinical indication)<br>☐ other
At what other time was maternal listeria status determined?	__________________________
Other than HIV and malaria, which STORCH pathogens were measured during pregnancy?<br><br>Select all that apply	☐ Syphilis<br>☐ Toxoplasmosis<br>☐ Other (varicella, parovirus B19)<br>☐ Rubella<br>☐ CMV<br>☐ Herpes
What other pathogens were measured during pregnancy?	__________________________
How did the study determine maternal syphilis infection status?<br><br>Select all that apply	☐ Self report<br>☐ Medical records<br>☐ Diagnostic test<br>☐ Other
Specify other means of determining maternal syphilis infection status	__________________________
Which diagnostic test(s) were applied to assess maternal syphilis infection status?<br><br>Select all that apply	☐ serology<br>☐ RT-PCR<br>☐ immunohistology<br>☐ darkfield microscopy<br>☐ other
What other diagnostic tests were applied to assess maternal syphilis infection status?	__________________________

For peer review only

Which serologic assay(s) was/were applied to assess maternal syphilis infection status?<br><br>Select all that apply	☐ TPPA (Treponema pallidum particle agglutination assay)<br>☐ TPHA (Treponema pallidum hemagglutination assay)<br>☐ Microhemagglutination assay (MHA-TP)<br>☐ EIA IgG<br>☐ EIA IgM<br>☐ IgM Immunoblot<br>☐ FTA-ABS (Fluorescent treponemal antibody absorption test)<br>☐ 19S-IgM-FTA-ABS<br>☐ Venereal Disease Research Laboratory test (VDRL)<br>☐ Rapid-Plasma-Reagin (RPR)<br>☐ Other
Which other serologic assay(s) was/were applied to assess maternal syphilis infection status?	__________________________________
When during pregnancy did the study plan to diagnose syphilis infection?<br><br>Select all that apply	☐ 1st term<br>☐ 2nd term<br>☐ 3rd term<br>☐ whenever symptoms were present (per clinical indication)<br>☐ other
At what other time during pregnancy did the study plan to diagnose syphilis infection?	__________________________________
How did the study determine maternal toxoplasmosis infection status?<br><br>Select all that apply	☐ Self report<br>☐ Medical records<br>☐ Diagnostic test<br>☐ Other
Specify other means of determining maternal toxoplasmosis infection status	__________________________________
Which diagnostic test(s) were applied to assess maternal toxoplasmosis infection status?<br><br>Select all that apply	☐ serology<br>☐ RT-PCR<br>☐ immunohistology<br>☐ darkfield microscopy<br>☐ other
What other diagnostic tests were applied to assess maternal toxoplasmosis infection status?	__________________________________
Which serologic assay(s) was/were applied to assess maternal toxoplasmosis infection status?<br><br>Select all that apply	☐ IgM<br>☐ IgG<br>☐ IgA<br>☐ Other
Which other serologic assay(s) was/were applied to assess maternal toxoplasmosis infection status?	__________________________________

For peer review only

When during pregnancy did the study plan to diagnose toxoplasmosis infection?<br><br>Select all that apply	☐ 1st term<br>☐ 2nd term<br>☐ 3rd term<br>☐ whenever symptoms were present (per clinical indication)<br>☐ other
At what other time during pregnancy did the study plan to diagnose toxoplasmosis infection?	__________________________________
How did the study determine maternal rubella infection status?<br><br>Select all that apply	☐ Self report<br>☐ Medical records<br>☐ Diagnostic test<br>☐ Other
Specify other means of determining maternal rubella infection status	____________________________
Which diagnostic test(s) were applied to assess rubella infection status?<br><br>Select all that apply	☐ serology<br>☐ RT-PCR<br>☐ immunohistology<br>☐ darkfield microscopy<br>☐ other
What other diagnostic tests were applied to assess maternal rubella infection status?	__________________________________
Which serologic assay(s) was/were applied to assess maternal rubella infection status?<br><br>Select all that apply	☐ TPPA (Treponema pallidum particle agglutination assay)<br>☐ TPHA (Treponema pallidum hemagglutination assay)<br>☐ Microhemagglutination assay (MHA-TP)<br>☐ EIA IgG<br>☐ EIA IgM<br>☐ IgM Immunoblot<br>☐ FTA-ABS (Fluorescent treponemal antibody absorption test)<br>☐ 19S-IgM-FTA-ABS<br>☐ Venereal Disease Research Laboratory test (VDRL)<br>☐ Rapid-Plasma-Reagin (RPR)<br>☐ Other
Which other serologic assay(s) was/were applied to assess maternal rubella infection status?	__________________________________
When during pregnancy did the study plan to diagnose rubella infection?<br><br>Select all that apply	☐ 1st term<br>☐ 2nd term<br>☐ 3rd term<br>☐ whenever symptoms were present (per clinical indication)<br>☐ other
At what other time during pregnancy did the study plan to diagnose maternal rubella infection?	__________________________________

For peer review only

How did the study determine maternal CMV infection status?<br><br>Select all that apply	☐ Self report<br>☐ Medical records<br>☐ Diagnostic test<br>☐ Other
Specify other means of determining maternal CMV infection status	__________________________
Which diagnostic test(s) were applied to assess maternal CMV infection status?<br><br>Select all that apply	☐ serology<br>☐ RT-PCR<br>☐ immunohistology<br>☐ darkfield microscopy<br>☐ other
What other diagnostic tests were applied to assess maternal CMV infection status?	__________________________
Which serologic assay(s) was/were applied to assess maternal CMV infection status?<br><br>Select all that apply	☐ TPPA (Treponema pallidum particle agglutination assay)<br>☐ TPHA (Treponema pallidum hemagglutination assay)<br>☐ Microhemagglutination assay (MHA-TP)<br>☐ EIA IgG<br>☐ EIA IgM<br>☐ IgM Immunoblot<br>☐ FTA-ABS (Fluorescent treponemal antibody absorption test)<br>☐ 19S-IgM-FTA-ABS<br>☐ Venereal Disease Research Laboratory test (VDRL)<br>☐ Rapid-Plasma-Reagin (RPR)<br>☐ Other
Which other serologic assay(s) was/were applied to assess maternal CMV infection status?	__________________________
Did the study conduct avidity assays for IgG positive CMV?	◯ Yes<br>◯ No
When during pregnancy did the study plan to diagnose maternal CMV infection?<br><br>Select all that apply	☐ 1st term<br>☐ 2nd term<br>☐ 3rd term<br>☐ whenever symptoms were present (per clinical indication)<br>☐ other
At what other time during pregnancy did the study plan to diagnose CMV infection?	__________________________
How did the study determine maternal herpes infection status?<br><br>Select all that apply	☐ Self report<br>☐ Medical records<br>☐ Diagnostic test<br>☐ Other
Specify other means of determining maternal herpes infection status	__________________________

For peer review only

Which diagnostic test(s) were applied to assess maternal herpes infection status?<br><br>Select all that apply	☐ serology<br>☐ RT-PCR<br>☐ immunohistology<br>☐ darkfield microscopy<br>☐ other
What other diagnostic tests were applied to assess maternal herpes infection status?	__________________________________
Which serologic assay(s) was/were applied to assess maternal herpes infection status?<br><br>Select all that apply	☐ TPPA (Treponema pallidum particle agglutination assay)<br>☐ TPHA (Treponema pallidum hemagglutination assay)<br>☐ Microhemagglutination assay (MHA-TP)<br>☐ EIA IgG<br>☐ EIA IgM<br>☐ IgM Immunoblot<br>☐ FTA-ABS (Fluorescent treponemal antibody absorption test)<br>☐ 19S-IgM-FTA-ABS<br>☐ Venereal Disease Research Laboratory test (VDRL)<br>☐ Rapid-Plasma-Reagin (RPR)<br>☐ Other
Which other serologic assay(s) was/were applied to assess maternal herpes infection status?	__________________________________
When during pregnancy did the study plan to diagnose herpes infection?<br><br>Select all that apply	☐ 1st term<br>☐ 2nd term<br>☐ 3rd term<br>☐ whenever symptoms were present (per clinical indication)<br>☐ other
At what other time during pregnancy did the study plan to diagnose herpes infection?	__________________________________

For peer review only

Additional pregnancy-level maternal exposures

How did the study determine maternal nutrition status?<br><br>Select all that apply	☐ Self report<br>☐ Medical records<br>☐ Empirical evaluation of nutrition status<br>☐ Other<br>☐ Not measured
Specify other means of determining maternal nutrition status	__________________
How did the study determine maternal smoking during pregnancy?<br><br>Select all that apply	☐ Self report<br>☐ Medical records<br>☐ Other<br>☐ Not measured
Specify other means of determining maternal smoking during pregnancy	__________________
How did the study determine maternal alcohol consumption?<br><br>Select all that apply	☐ Self report<br>☐ Medical records<br>☐ Other<br>☐ Not measured
Specify other means of determining maternal alcohol consumption	__________________
How did the study determine maternal recreational drug use?<br><br>Select all that apply	☐ Self report<br>☐ Medical records<br>☐ Other<br>☐ Not measured
Specify other means of determining maternal recreational drug use	__________________
How did the study determine prescription drug or other medication use during pregnancy?<br><br>Select all that apply	☐ Self report<br>☐ Medical records<br>☐ Other<br>☐ Not measured
Specify other means of determining maternal prescription drug or medication use	__________________
How were vaccinations during pregnancy determined?<br><br>Select all that apply	☐ Self report<br>☐ Medical records<br>☐ Other<br>☐ Not measured
Specify other means of determining vaccinations during pregnancy	__________________
How did the study determine exposure to workplace or environmental teratogens (glycol ethers, solvents, heavy metals, pesticides) during pregnancy?<br><br>Select all that apply	☐ Self report<br>☐ Medical records<br>☐ Other<br>☐ Not measured

For peer review only

Specify other means of determining exposure to workplace or environmental teratogens	__________________________
How was exposure to intimate partner violence during pregnancy determined?<br><br>Select all that apply	☐ Self report<br>☐ Medical records<br>☐ Other<br>☐ Not measured
Specify other means of determining exposure to intimate partner violence during pregnancy	__________________________

For peer review only

Other infant/child-level covariates

How was infant/child exposure to family violence determined?<br><br>Select all that apply	☐ Self report<br>☐ Medical records<br>☐ Other<br>☐ Not measured
Specify other means of determining infant/child exposure to family violence	__________________________
Did the study measure family structure after the birth of the index child?	◯ Yes<br>◯ No
Do infants or children in the study receive any developmental interventions (e.g. physical, speech, motor therapy) provided by the study or by the health system?	◯ Yes<br>◯ No<br>◯ Not measured
Please describe the physical therapy/motor therapy interventions received by infants or children in the study	__________________________
Please describe the speech/language therapy interventions received by infants or children in the study	__________________________
Please describe the cognitive therapy interventions received by infants or children in the study	__________________________
Please describe any other types of therapies or developmental interventions received by infants or children in the study	__________________________

For peer review only

Appendix 3

ZIKV-IPD-MA Consortium participants

Pablo Aguilar Ticona, MD
Institute of Collective Health
Federal University of Bahia
Salvador, Brazil

Luiz Carlos Junior Alcantara, PhD
Flavivirus Reference Laboratory (LABFLA) from Oswaldo Cruz Institution (IOC)
Oswaldo Cruz Foundation (FIOCRUZ)
Rio de Janeiro, Brazil

Jackeline Alger, MD, PhD
Facultad de Ciencias Médicas
Universidad Nacional Autónoma de Honduras
Tegucigalpa, Honduras

Celia Alpuche Aranda, MD, PhD
CISEI Centro de Investigación Sobre Enfermedades Infecciosas
Institution Instituto Nacional de Salud Publica de México
Cuernavaca, México

Jonathan Altamirano, MS
Department of Pediatrics
Division of Infectious Diseases
Stanford University School of Medicine
Stanford, California, USA

Juan Arias, MD, PhD
Infectious Diseases
St Jude's Children's Research Hospital
Memphis, Tennessee, USA

Lumumba Arriaga-Nieto, MD
Instituto Mexicano del Seguro Social
Mexico City, Mexico
Melissa A. G. Avelino, PhD
Department of Surgery of Goiás Federal University
Goiânia, Goiás, Brazil
Angel Balmaseda, MD
Department of Virology
Sustainable Sciences Institute
Managua, Nicaragua

Azucena Bardají, MD, PhD
ISGlobal
Hospital Clínic-Universitat de Barcelona
Barcelona, Spain

Consorcio de Investigación Biomédica en Red de Epidemiología y Salud Pública (CIBERESP)
Madrid, Spain

Centro de Investigação em Saúde de Manhiça (CISM)
Maputo, Mozambique

Carlos Hernan Becerra Mojica, MD
Departamento de Obstetricia y Ginecologia
Universidad Industrial de Santander

For peer review only

Bucaramanga, Colombia

Mónica Benavides, MPH
Dirección de Vigilancia y Análisis del Riesgo en Salud Publica
Grupo de enfermedades Inmunoprevenibles
Instituto Nacional de Salud
Bogotá, Colombia

A.P. Bertozzi, MD, PhD
Department of Pediatrics
Faculty of Medicine of Jundiaí
São Paulo, Brazil

Karen Blackmon, PhD
Department of Psychiatry and Psychology
Mayo Clinic
Jacksonville, FL, USA

Victor Hugo Borja Aburto, MD
Instituto Mexicano del Seguro Social
Mexico City, Mexico

Patrícia Brasil, MD, PhD
Laboratório de Pesquisa Clínica em Doenças Febris Agudas
Instituto Nacional de Infectologia Evandro Chagas - FIOCRUZ
Rio de Janeiro, Brazil

William J Britt, MD
Department of Pediatrics
Heersink School of Medicine
University of Alabama
Birmingham, AL

Nathalie Broutet, MD, PhD
UNDP/UNFPA/UNICEF/WHO/World Bank Special Programme of Research, Development and Research Training in Human Reproduction
Department of Sexual and Reproductive Health and Research
World Health Organization
Geneva, Switzerland

Pierre Buekens, MD, PhD
School of Public Health and Tropical Medicine
Tulane University
Louisiana, USA

André Cabié, MD, PhD
Infectious Diseases and Tropical Medicine
Université des Antilles, CHU de Martinique, INSERM CIC 1424, INSERM PCCEI UMR 1058
Fort-de-France, Martinique, France

David Alejandro Cabrera-Gaytán, MD
Instituto Mexicano del Seguro Social
Mexico City, Mexico

Rodrigo Cachay Figueroa, MD

For peer review only

Instituto de Medicina Tropical Alexander von Humboldt
Universidad Peruana Cayetano Heredia
Lima, Perú

María Luisa Cafferata, MD
Mother and Children Health Research Department
Institute for Clinical Effectiveness and Health Policy
Buenos Aires, Argentina

Juan Ignacio Calcagno, MD
Department of Hospital Epidemiology
Maternidade Professor José Maria de Magalhães Netto
Salvador, Bahia, Brazil

Juan P. Calle, MD
Department of Pediatrics, Universidad del Quindío
Centro de Estudios en Infectología Pediátrica
Cali, Colombia

Mabel Carabali, MD, PhD
Département de Médecine Sociale et Préventive, École de Santé Publique,
Université de Montréal,
Montreal, Quebec, Canada.

Department of Epidemiology, Dalla Lana School of Public Health,
University of Toronto.
Toronto, Ontario, Canada.

Derrick WS Chan, MD
Paediatric Neurology, Paediatric Medicine
KK Women's and Children's Hospital
Singapore

Celia CD Christie, MBBS, DM Peds
Department of Child and Adolescent Health
University of the West Indies, Mona Campus
Kingston, Jamaica

Federico Costa, PhD
Institute of Collective Health
Federal University of Bahia
Salvador, Brazil

Antonio Jose Cunha, PhD
Departamento de Pediatria
Federal University of Rio de Janeiro
Rio de Janeiro, Brazil

Carlos Cure Cure, MD
BIOMELAB SAS
Barranquilla, Colombia

Johanna Antonia Adriana Damen, PhD
Julius Center for Health Sciences and Primary Care
University Medical Center Utrecht
Utrecht University

For peer review only

Utrecht, Netherlands

Marcela C. Daza, Médica Pediatra, MSc
Grupo de Investigación en Salud Materna y Perinatal
Dirección de Investigación en Salud Pública
Instituto Nacional de Salud
Bogotá, Colombia

Roberta L. DeBiasi, MD
Departments of Pediatrics and Microbiology, Immunology and Tropical Medicine
The George Washington University School of Medicine
Children's National Hospital
Washington, DC, USA

Thomas Debray, PhD
Julius Center for Health Sciences and Primary Care
University Medical Center Utrecht
Utrecht University
Utrecht, Netherlands

Valentijn M.T. de Jong, PhD
Julius Center for Health Sciences and Primary Care
University Medical Center Utrecht
Utrecht University
Utrecht, Netherlands

Data Analytics and Methods Task Force
European Medicines Agency
Amsterdam, Netherlands
Camille Delgado López, MPH
Puerto Rico Department of Health
San Juan, Puerto Rico

Leah deWilde, BS
Epidemiology Division
Virgin Islands Department of Health
Christiansted, St. Croix, Virgin Islands, USA

Alan Oliveira Duarte, MSc
Instituto Gonçalo Moniz
Fundação Oswaldo Cruz
Salvador, Bahia, Brazil

Geraldo Duarte, MD, PhD
Ribeirão Preto Medical School
University of São Paulo
São Paulo, Brazil

Valorie Eckert, MPH
California Birth Defects Monitoring Program
Genetic Disease Screening Division
California Department of Public Health
Richmond, CA USA

Esther M. Ellis, PhD
Territorial Epidemiologist

For peer review only

Virgin Islands Department of Health
Christiansted, St. Croix, Virgin Islands, USA

Andres Espinosa-Bode, MD
Division of Global Health Protection, Central America Region Office
Centers for Disease Control and Prevention
Atlanta, GA, USA

Cassia Fernanda Estofolete, MD, PhD
Department of Dermatologic, Infectious and Parasititc Diseases
Faculdade de Medicina de São José do Rio Preto
São José do Rio Preto, Brazil

Roberta Evans, MS
Windward Islands and Research Education Foundation
St. George, Grenada

Valéria Christina de Rezende Féres, PhD
Laboratório BIOTEC
Faculdade de Farmácia - Universidade Federal de Goiás
Setor Leste Universitário, Goiânia, Brasil

Fabíola Fiaccadori, PhD
Virology and Cell Culture Laboratory
Institute of Tropical Pathology and Public Health
Federal University of Goiás
Goiânia, Goiás, Brazil

Lester Fernando Figueroa Bolaños, MD
Departamento de Planificacion
Instituto de Nutricion de Centroamerica y Panama (INCAP)
Guatemala City, Guatemala

Olivier Fléchelles, MD, PhD
Pediatric and Neonatal Intensive Care Unit
University Hospital of Martinique
Martinique, France

Arnaud Fontanet, MD, DrPH
Global Health
Institut Pasteur
Paris, France

Victoria Fumadó, MD, PhD
Infectious and Imported Diseases Unit
Department of Pediatrics, Sant Joan de Déu University
Hospital Research Foundation
Barcelona, Spain

Anna L. Funk, PhD
Department of Pediatrics, Cumming School of Medicine
University of Calgary
Calgary, Alberta, Canada

Anna Gajewski, MPH
Sustainable Sciences Institute

For peer review only

Managua, Nicaragua

Rosa Margarita Gelvez Ramirez, MSc
Centro de Atención y Diagnóstico de Enfermedades Infecciosas-CDI
Santander, Colombia

Carlo Giaquinto, MD
Dipartimento di Salute della Donna e del Bambino
Università degli Studi di Padova
Italy

Luz Gibbons, MSc
Institute for Clinical Effectiveness and Health Policy
Buenos Aires, Argentina

Suzanne M. Gilboa, PhD
National Center on Birth Defects and Developmental Disabilities
Centers for Disease Control and Prevention
Atlanta, GA, USA

Maria Barbara Franco Gomes, MD
Maternal Infant Hospital
Goiania, Brazil

Anna Goncé, MD, PhD
Barcelona Centre for Maternal-Fetal and Neonatal Medicine (Hospital Clínic and Hospital Sant Joan de Déu)

Institut d'Investigacions Biomèdiques August Pi i Sunyer (IDIBAPS)
Universitat de Barcelona

Centre for Biomedical Research on Rare Diseases (CIBER-ER)
Barcelona, Spain

Cesar R Gonzalez-Bonilla, MD, PhD
Coordinación de Investigación en Salud
Instituto Mexicano del Seguro Social
Mexico City, Mexico

Eduardo Gotuzzo, MD
Instituto de Medicina Tropical Alexander Von Humboldt
Universidad Peruana Cayetano Heredia
Lima, Peru

Concepción Grajales-Muñiz, MD
Instituto Mexicano del Seguro Social
Mexico City, Mexico

Rebecca Grant, MSc, MPH
Epidemiology of Emerging Diseases Unit
Institut Pasteur
Paris, France

Elysse Grossi-Soyster, PhD(c)
Sarnow and Schnieder Labs
Microbiology & Immunology Department
Stanford University School of Medicine

For peer review only

Tahani Hamdan, MPH
Houston Health Department
City of Houston
Houston, TX, USA

Eva Harris, PhD
Division of Infectious Diseases and Vaccinology, School of Public Health
University of California, Berkeley
Berkeley, CA, USA

Cosme Harrison, MPH
Division of Epidemiology
Virgin Islands Department of Health
Saint Thomas/Virgin Islands, USA

Bruno Hoen, MD, PhD
Infectious Diseases and Tropical Medicine
University hospital of Guadeloupe
Pointe-à-Pitre, Guadeloupe, France

Cristina Hofer, PhD
Instituto de Puericultura e Pediatria Martagão Gesteira
Universidade Federal do Rio de Janeiro
Rio de Janeiro, Brazil

Natanael Holband, MD
Academic Pediatric Center Suriname
Academic Hospital Paramaribo
Paramaribo, Suriname

Ivonne Huerta, MD
CISEI Centro de Investigación Sobre Enfermedades Infecciosas
Institution Instituto Nacional de Salud Publica de México
Cuernavaca, México

Irene Inwani, MD
Department of Paediatrics and Child Health
Kenyatta National Teaching and Referral Hospital
Nairobi, Kenya

Thomas Jaenisch, MD, PhD
Heidelberg Institute for Global Health
Heidelberg University Hospital
Heidelberg, Germany

Center for Global Health
Colorado School of Public Health
University of Colorado
Denver, CO, USA

Esaú João, PhD
Department of Infectious Diseases
Hospital Federal dos Servidores do Estado
Rio de Janeiro, Brazil

For peer review only

Edna Kara, MD
UNDP/UNFPA/UNICEF/WHO/World Bank Special Programme of Research, Development and Research Training in Human Reproduction
Department of Sexual and Reproductive Health and Research
World Health Organization
Geneva, Switzerland

Salma Khuwaja, MD, DrPH
Houston Health Department
City of Houston
Houston, TX, USA

Caron Kim, MD
UNDP/UNFPA/UNICEF/WHO/World Bank Special Programme of Research, Development and Research Training in Human Reproduction
Department of Sexual and Reproductive Health and Research
World Health Organization
Geneva, Switzerland

Albert I. Ko, MD
Department of Epidemiology of Microbial Diseases
Yale School of Public Health
New Haven, CT, USA

Nancy F. Krebs, MD
Department of Pediatrics, Section of Nutrition
University of Colorado School of Medicine
Aurora, CO, USA

Angelle Desiree LaBeaud, MD
Department of Pediatrics
Division of Infectious Diseases
Stanford University School of Medicine
Stanford, California, USA

Heather Lake-Burger, MS, MPH
Division of Community Health Promotion
Florida Department of Health
Tallahassee, FL, USA

Ellen H. Lee, MD
Bureau of Communicable Disease
New York City Department of Health and Mental Hygiene
New York, USA

Vernon Lee, PhD
Saw Swee Hock School of Public Health
National University of Singapore
Singapore

Yee-Sin Leo, MD
Executive Director's Office
National Centre for Infectious Diseases
Singapore

For peer review only

Brooke Levis, PhD
Centre for Clinical Epidemiology
Lady Davis Institute of Medical Research
Jewish General Hospital
Montreal, Quebec

Centre for Prognosis Research, School of Medicine,
Keele University,
Staffordshire, United Kindom

Eduardo Lopez-Medina, MD
Centro de Estudios en Infectología Pediátrica —CEIP—
Department of Pediatrics, Universidad del Valle
Clínica Imbanaco, Grupo Quironsalud
Cali, Valle, Colombia

Anyela Lozano-Parra, MSc
Facultad de Salud
Universidad Industrial de Santander
Santander, Colombia

Elena Marbán-Castro, PhD
ISGlobal
Hospital Clínic-Universitat de Barcelona
Barcelona, Spain

Celina Maria Turchi Martelli, PhD
The Research Centre Aggeu Magalhães
Fundação Oswaldo Cruz (Fiocruz)
Recife, Brazil

Lauren Maxwell, PhD
Heidelberg Institute for Global Health
Heidelberg University Hospital
Heidelberg, Germany

Department of Sexual and Reproductive Health and Research
Human Reproduction Programme
World Health Organization

Clara Menéndez, MD, PhD
ISGlobal
Hospital Clínic-Universitat de Barcelona
Barcelona, Spain

Consorcio de Investigación Biomédica en Red de Epidemiología y Salud Pública (CIBERESP)
Madrid, Spain

Centro de Investigação em Saúde de Manhiça (CISM)
Maputo, Mozambique

Marcela Mercado Reyes, MSc
Public Health Research Division
Instituto Nacional de Salud - Colombia
Bogotá, Colombia

For peer review only

Conrado Milani Coutinho, MD, PhD
Departmento de Ginecologia e Obstetrícia
Hospital das Clínicas da Faculdade de Medicina de Ribeirão Preto da Universidade de São Paulo
São Paulo, Brazil

María Consuelo Miranda Montoya, MD
Facultad de Salud Carrera
Universidad Industrial de Santander
Santander, Colombia

Demócrito de Barros Miranda-Filho, PhD
Faculty of Medical Sciences
Federal University of Pernambuco
Recife, Brazil

Maria Elisabeth Moreira, PhD
Department of Neonatology
Fundação Oswaldo Cruz (Fiocruz)
Rio de Janeiro, Brazil

J. Glenn Morris, Jr., MD
Emerging Pathogens Institute
University of Florida
Gainesville, FL, USA

Sarah B. Mulkey, MD, PhD
Departments of Neurology and Pediatrics
The George Washington University School of Medicine
Prenatal Pediatrics Institute
Children's National Hospital
Washington, DC, USA

Johanna Munoz, PhD
Julius Center for Health Sciences and Primary Care
University Medical Center Utrecht
Utrecht University
Utrecht, Netherlands

José Esteban Muñoz-Medina, PhD
Department of Epidemiology
Instituto Mexicano del Seguro Social
Mexico City, Mexico

Peninah Munyua, PhD
Division of Global Health Protection
US Centers for Disease Control and Prevention
Nairobi, Kenya

Marisa Marcia Mussi-Pinhata, MD, PhD
Departamento de Pediatria
Faculdade de Medicina de Ribeirão Preto da Universidade de São Paulo
São Paulo, Brazil

Nivison Nery Jr., MSc
Instituto de Saúde Coletiva

For peer review only

Federal University of Bahia
Salvador, Brazil

M. Kariuki Njenga, PhD
Paul Allen Center for Global Health
Washington State University
Nairobi, Kenya

Mauricio L. Nogueira, MD, PhD
Department of Dermatologic, Infectious and Parasititc Diseases
Faculdade de Medicina de São José do Rio Preto
São José do Rio Preto, Brazil

Eric Osoro, MD
Department of Global Health
Washington State University, Kenya
Nairobi, Kenya

Miguel Parra-Saavedra, PhD
Department of Gynecology and Obstetrics
Universidad Simon Bolivar, CEDIUL
Barranquilla, Colombia

Saulo Passos, PhD
Department of Pediatrics
Faculty of Medicine of Jundiaí
São Paulo, Brazil

Bernadete Perez Coêlho MD, PhD
Department of Public Health of the Medical Sciences Center
Federal University of Pernambuco
Recife, Pernambuco, Brasil

Monika Piccardi RN, BSN, MS
Office for and People with Special Healthcare Needs
Maryland Department of Health
Baltimore, MD, USA

Léo Pomar, PhD
Department of Obstetrics and Gynecology
Centre Hospitalier de l'Ouest Guyanais
Saint-Laurent du Maroni, French Guiana

Materno-fetal and Obstetrics Research Unit
Department Woman-mother-child
Lausanne University Hospital, Switzerland

School of Health Sciences (HESAV)
University of Applied Sciences and Arts Western Switzerland
Delémont, Switzerland

Arnaldo Prata-Barbosa, PhD
Department of Pediatrics
D'Or Institute for Research & Education
Rio de Janeiro, Brazil

For peer review only

Ingrid Rabe, MBChB, MMed
Emerging Diseases and Zoonoses Unit
Global Infectious Hazards
Health Emergencies Programme
World Health Organization
Geneva, Switzerland

Mitermayer Reis, MD, PhD
Laboratório de Patologia e Biologia Molecular
Fundação Oswaldo Cruz (Fiocruz)
Salvador, Brazil

Hannah Rettler, MPH
Bureau of Epidemiology
Utah Department of Health
Salt Lake City, USA

Megan R. Reynolds, MPH
National Center on Birth Defects and Developmental Disabilities
Centers for Disease Control and Prevention
Atlanta, GA, USA

Ana Maria Rivera Casas, MD
Maternal-Fetal Medicine
FUCS University
Bogota, Colombia

Diana Patricia Rojas, MD, PhD
WHE/GIH/EZD
Zika and Chikungunya
World Health Organization
Geneva, Switzerland

Teresita Rojas-Mendoza, MD
Instituto Mexicano del Seguro Social
Mexico City, Mexico

Nicole Roth, MPH
National Center on Birth Defects and Developmental Disabilities
Centers for Disease Control and Prevention
Atlanta, GA, USA

Paola Mariela Saba Villarroel, PhD
Unité des Virus Émergents
UVE: Aix-Marseille University-IRD 190-INSERM 1207-IHU Méditerranée Infection
Marseille, France

Magda Sanz Cortes, MD, PhD
Department of Obstetrics and Gynecology
Fetal Surgery and Intervention
Baylor College of Medicine
Houston, TX, USA

Janet L. Sayers, MS
UNDP/UNFPA/UNICEF/WHO/World Bank Special Programme of Research, Development and Research Training in Human Reproduction

For peer review only

Department of Sexual and Reproductive Health and Research
Human Reproduction Programme
World Health Organization
Geneva, Switzerland

Deolinda Scalabrin, MD, PhD
Instituto Gonçalo Moniz
Fundação Oswaldo Cruz (Fiocruz)
Salvador, Bahia, Brasil

Lavinia Schuler-Faccini, MD, PhD
Genetics Department
Hospital de Clinicas de Porto Alegre
Universidade Federal do Rio Grande do Sul
Porto Alegre, Rio Grande do Sul, Brazil

Stacey Schultz-Cherry, PhD
Department of Infectious Diseases
St Jude Graduate School
St. Jude Children's Research Hospital
Memphis, Tennessee, USA

Kirstin Short, MPH
Houston Health Department
City of Houston
Houston, Texas, USA

Priya Shreedhar, MSc
Heidelberg Institute of Global Health
Heidelberg University
Heidelberg, Germany

Antônio Augusto Silva, PhD
Department Public Health
Federal University of Maranhão
Sao Luis, Maranhão, Brazil

Ronaldo Silva, PhD
UNDP/UNFPA/UNICEF/WHO/World Bank Special Programme of Research, Development and Research Training in Human Reproduction
Department of Sexual and Reproductive Health and Research
Human Reproduction Programme

Isadora Siqueira, MD, PhD
Researcher in Public Health
Instituto Gonçalo Moniz-Fiocruz
Salvador-BA, Brasil

Karen Sohan, MD
Consultant Obstetrician & Gynaecologist
Diagnostic (Mother & Baby) Ltd
Trinidad & Tobago

Carmen Soria-Segarra, MD PhD
SOSECALI C. Ltda
Universidad Católica Santiago de Guayaquil

For peer review only

Guayaquil, Ecuador

Antoni Soriano-Arandes, MD, PhD
Paediatric Infectious Diseases and Immunodeficiencies Unit
Hospital Universitari Vall d'Hebron
Barcelona, Catalonia, Spain

Patrícia da Silva Sousa, MD, PhD
Child Neurology and Clinical Neurophysiology
Universidade Federal do Maranhão - UFMA
Sao Luis, Maranhão, Brazil

Maria Benamor Teixeira, MD, PhD
Department of Infectious Diseases
Hospital Federal dos Servidores do Estado
Rio de Janeiro, Brazil

Claire Thorne, PhD
Great Ormond Street Institute of Child Health
University College London
London, UK

Soe Soe Thwin, PhD
UNDP/UNFPA/UNICEF/WHO/World Bank Special Programme of Research, Development and Research Training in Human Reproduction
Department of Sexual and Reproductive Health and Research
Human Reproduction Programme
World Health Organization
Geneva, Switzerland

Van T. Tong, MPH
National Center on Birth Defects and Developmental Disabilities
Centers for Disease Control and Prevention
Atlanta, GA, USA

Benoit Tressieres, MSc
Centre d'Investigation Clinique Antilles Guyane, Inserm CIC 1424
CHU de la Guadeloupe
Pointe-à-Pitre, France

Marília Dalva Turchi, PhD
Institute of Tropical Pathology and Public Health
Federal University of Goias
Goiânia, Brazil

Diana Valencia, MS
National Center on Birth Defects and Developmental Disabilities
Centers for Disease Control and Prevention
Atlanta, GA, USA

Miguel Valencia-Prado, MD
Puerto Rico Department of Health
San Juan, Puerto Rico

Alfonso Vallejos-Parás, PhD
Instituto Mexicano del Seguro Social

For peer review only

Mexico City, Mexico

Maria Van Kerkhove, PhD
WHO/WHE/GIH Unit head Emerging and Zoonotic Diseases
World Health Organization
Geneva, Switzerland

Luis Angel Villar, MD, PhD
Facultad de Salud Carrera
Universidad Industrial de Santander
Santander, Colombia

Carmen Viñuela Benéitez, MD
Department of Obstetrics and Gynecology.
Gregorio Marañón University Hospital School of Medicine
Complutense University.
Madrid, Spain

Manon Vouga, MD, PhD
Department Woman-Mother-Child
Lausanne University Hospital,
Lausanne, Switzerland

Randall Waechter, PhD
Department of Neuroscience, Physiology, & Behavioral Science
School of Medicine, St. George's University
St. George, Grenada

Yinghui Wei, PhD
Centre for Mathematical Sciences
School of Engineering, Computing and Mathematics
University of Plymouth
Plymouth, UK

Jamie Westcott, MS
Section of Pediatric Nutrition
University of Colorado School of Medicine
Aurora, CO, USA

Marc-Alain Widdowson, VetMB, MA, MSc
Division of Global Health Protection
Centers for Disease Control and Prevention
Nairobi, Kenya

George SH Yeo, MD
Division of Obstetrics and Gynecology
KK Women's and Children's Hospital
Singapore

Ricardo Arraes de Alencar Ximenes, MD, PhD
Department of Tropical Medicine
Federal University of Pernambuco
Recife, Brazil

For peer review only

For peer review only
